# Evolution of Spatially Coexpressed Families of Type-2 Vomeronasal Receptors in Rodents

**DOI:** 10.1093/gbe/evu283

**Published:** 2014-12-23

**Authors:** Simona Francia, Lucia Silvotti, Filippo Ghirardi, François Catzeflis, Riccardo Percudani, Roberto Tirindelli

**Affiliations:** ^1^Department of Neuroscience, University of Parma, Italy; ^2^Laboratoire de Paleontologie, Institut des Sciences de l’Evolution, UMR 5554 Centre National de la Recherche Scientifique, Université de Montpellier 2, France; ^3^Department of Life Sciences, University of Parma, Italy

**Keywords:** vomeronasal, pheromones, chemosensory, evolution, phylogeny, rodents

## Abstract

The vomeronasal organ (VNO) is an olfactory structure for the detection of pheromones. VNO neurons express three groups of unrelated G-protein-coupled receptors. Type-2 vomeronasal receptors (V2Rs) are specifically localized in the basal neurons of the VNO and are believed to sense protein pheromones eliciting specific reproductive behaviors. In murine species, V2Rs are organized into four families. Family-ABD V2Rs are expressed monogenically and coexpress with family-C V2Rs of either subfamily C1 (V2RC1) or subfamily C2 (V2RC2), according to a coordinate temporal diagram. Neurons expressing the phylogenetically ancient V2RC1 coexpress family-BD V2Rs or a specific group of subfamily-A V2Rs (V2RA8-10), whereas a second neuronal subset (V2RC2-positive) coexpresses a recently expanded group of five subfamily-A V2Rs (V2RA1-5) along with vomeronasal-specific Major Histocompatibility Complex molecules (H2-Mv). Through database mining and Sanger sequencing, we have analyzed the onset, diversification, and expansion of the V2R-families throughout the phylogeny of Rodentia. Our results suggest that the separation of V2RC1 and V2RC2 occurred in a Cricetidae ancestor in coincidence with the evolution of the H2-Mv genes; this phylogenetic event did not correspond with the origin of the coexpressing V2RA1-5 genes, which dates back to an ancestral myomorphan lineage. Interestingly, the evolution of receptors within the V2RA1-5 group may be implicated in the origin and diversification of some of the V2R putative cognate ligands, the exocrine secreting peptides. The establishment of V2RC2, which probably reflects the complex expansion and diversification of family-A V2Rs, generated receptors that have probably acquired a more subtle functional specificity.

## Introduction

The accessory olfactory organ or vomeronasal organ (VNO) of Jacobson is a sensory structure dedicated to the detection of pheromones that are molecules secreted or excreted by conspecifics (Tirindelli et al. 2009).

The VNO first originated in a tetrapod ancestor and led to the appearance of a rudimentary structure in amphibians that became highly organized in many animal orders such as Squamata, Didelphimorphia, Rodentia, and in primates (Prosimians and New World Monkeys). In contrast, VNO is absent in birds, bats, Old World Monkeys, Apes, and humans (Dennis et al. 2004; Grus et al. 2005; Smith et al, 2005; Shi and Zhang 2007; Zhao et al. 2011; Syed et al. 2013). In Didelphimorpha, Lagomorpha, and Rodentia, the VNO presents two distinct neuronal layers (apical and basal) characterized by the expression of different G-protein subunits and receptors (Young and Trask 2007). Apical and basal neurons project to two separate regions (anterior and posterior) of the accessory olfactory bulb. From here, the projections of the apical and basal neurons remain segregated in the amygdala and hypothalamus (Yoon et al. 2005; Martinez-Marcos 2009).

The molecular organization of the rodent vomeronasal neuroepithelium in apical and basal neurons is based on the specific expression pattern of two transduction molecules, namely G-protein Gα_i2_ and Gα_o_, and two distinct groups of putative pheromone receptors namely type-1 vomeronasal receptors (V1Rs) (Dulac and Axel 1995) and type-2 vomeronasal receptors (V2Rs) (Herrada and Dulac 1997; Matsunami and Buck 1997; Ryba and Tirindelli 1997). The vomeronasal neurons also express formyl-peptide receptors (FPRs) which are known to sense antimicrobial peptides rather than pheromonal cues (Liberles et al. 2009; Riviere et al. 2009). Gα_i2_ is expressed in the apical neurons and colocalizes with V1Rs, whereas Gα_o_ coexpresses with V2Rs in the basal neurons (Dulac and Axel 1995; Herrada and Dulac 1997; Matsunami and Buck 1997; Ryba and Tirindelli 1997). FPRs are expressed in both apical and basal region of rodent VNO (Liberles et al. 2009; Riviere et al. 2009). In addition to V1Rs, V2Rs, and FPRs, the basal neurons of the VNO specifically express molecules of the nonclassical class I genes of the Major Histocompatibility Complex termed H2-Mv. The nine vomeronasal-specific H2-Mv genes are differentially expressed in subsets of basal neurons (Ishii et al. 2003; Loconto et al. 2003). Although not indispensable to generate physiological responses in the VNO neurons, H2-Mv molecules are required to obtain a supersensitive detection of pheromones (Leinders-Zufall et al. 2014).

V2Rs differ from V1Rs for the presence of introns and for the long N-terminal extracellular region that reflects the chemical properties of their ligands. In fact, the sequence analysis of V2Rs reveals that the highest intraspecies and interspecies variability is located in the extracellular N-terminal domain (Yang et al. 2005), which is believed to bind pheromones. Whereas the airborne pheromones are detected by V1Rs (Boschat et al. 2002), evidence indicates that substances such as major urinary proteins, gland secreting peptides (ESPs) and MHC peptides are candidates as V2R ligands (Loconto et al. 2003; Kimoto et al. 2005; Ishii and Mombaerts 2008; Leinders-Zufall et al. 2009; Papes et al. 2010; Ferrero et al. 2013; Sturm et al. 2013). However, only one member of the large family of exocrine secreting peptides (ESPs), namely the lacrimal peptide ESP1, has been unequivocally demonstrated to bind to a specific V2R subtype, eliciting behavioral effects in female mouse (Haga et al. 2010; Abe and Touhara 2014).

From the evolutionary point of view, V2R genes appeared in fish and amphibians (Shi and Zhang 2007; Young and Trask 2007; Grus and Zhang 2009; Ji et al. 2009; Francia et al. 2014). A striking variation of V2R genes occurred in terricolous species as intact genes have only been reported in Squamata (lizard and snake), Didelphimorpha, (opossum), and Rodentia and Lagomorpha (rabbit). No functional V2Rs have been identified in Carnivora (dog), Artiodactyla (cow), or primates (macaque, chimpanzee, gorilla, and human) with the exception of prosimians (loris, lemur, and tarsier) (Dong et al. 2012; Hohenbrink et al. 2012; Yang et al. 2005; Shi and Zhang 2007; Young and Trask 2007; Ishii and Mombaerts 2011). In mouse and rat, V2Rs are classified into four families, A–D (Yang et al. 2005). Receptors of family A typically represent the majority of the V2Rs (95% in the mouse) and show a strong lineage specificity so that orthologs can be exclusively found in closely related species, in which they tend to form small but independent clades (Grus and Zhang 2008). In mouse and rat, family A further expanded originating two distinct groups, namely subfamily A1-6 and subfamily A7-10 (Silvotti et al. 2011). Family C is the most ancient among V2R families and it is typically represented by one or two genes in each species with the exception of mouse and rat (Rodriguez 2004; Shi and Zhang 2007; Silvotti et al. 2011; Brykczynska et al. 2013). Intact family-C genes are found in prosimians whereas Old World Monkeys, Apes, and humans possess only pseudogenes (Hohenbrink et al. 2013). In mouse and rat, family-C V2Rs expanded, originating two distinct subfamilies, namely C1 and C2. In the VNO, family-ABD V2Rs are expressed monogenically (Rodriguez et al. 1999), although the basal neurons show a multigenic expression of V2Rs. In fact, family-ABD V2Rs are coexpressed with family-C V2Rs according to a specific pattern (Martini et al. 2001; Silvotti et al. 2007, 2011). In the rat and mouse, the expansion of family C and family A defined two populations of basal neurons. One population expresses subfamily A8-10, family-BD, and subfamily-C1 V2Rs, whereas the other population expresses combinations of subfamily A1-6 and subfamily-C2 V2Rs (Silvotti et al. 2011).

In this study, we analyzed some evolutionary features of V2Rs in Rodentia. First, we characterized the phylogenetic tree of these receptors starting from the most basal species. Second, we identified when the expansion of family A and family C V2Rs occurred. Third, we traced the evolutionary history of the V2R coexpressing H2-Mv genes and of the V2R putative protein ligands, ESPs. Finally, we sought a correlate between the diversification and expansion of family-C genes with their potential functions. To perform this evolutionary analysis, we partially reconstructed the V2R sequences in various rodent species either employing the currently available databases or by PCR amplification and Sanger sequencing of genomic DNA obtained from tissue specimens.

## Materials and Methods

### Species Abbreviations

MURIDAE: *Mus musculus* (ms), *Rattus norvegicus* (rt), *Meriones unguiculatus* (mr). CRICETIDAE: *Cricetulus griseus* (cr), *Mesocricetus auratus* (mcr), *Peromyscus maniculatus* (pr), *Microtus ochrogaster* (mic). SPALACIDAE: *Spalax leucodon* (sp), *Nannospalax galili* (ns). DIPODIDAE: *Jaculus jaculus* (jc). ANOMALURIDAE: *Anomalurus* sp. (an). PEDETIDAE: *Pedetes capensis* (pd). HETEROMYIDAE: *Dipodomys ordii* (dp). CAVIIDAE: *Cavia porcellus* (cv). BATHYERGIDAE: *Heterocephalus glaber* (ht). SCIURIDAE: *Ictidomys tridecemlineatus* (sq), *Sciurus vulgaris* (scv). LAGOMORPHA: *Oryctolagus cuniculus* (rb).

### Genomic Amplification

Tissues of different rodent species used for amplification were obtained from purchased animals (T-RT1, T-RT2), roadkill animals (T-RT3), or from the tissue library of the University of Montpellier, France (Michaux et al. 2001) (table 1).

**Table 1:** Distribution of V2R Genes in Rodent Species

Family	Species	Name	Source^a^	A1-5	A6	A8-9	E	B	D	C1	C2	H2Mv^b^
Muridae	*Mu. musculus*	Mouse^b^	nr	66	1	33	1	9	4	1	6	9
	*R. norvegicus*	Rat^b^	nr	38	5	22	–	5	4	1	3	7
	*M. unguiculatus*	Gerbil	T-RT1	11 (1)	–	5 (3)				1		
Cricetidae	*C. griseus*	Chinese hamster	nr; WGS	16 (10)	2	16 (9)	6 (3)	(1)	2 (1)	1	1 (2)	7
	*Mes. auratus*	Golden hamster	T-RT2; WGS	5 (3)	3	10					1	
	*P. maniculatus*	Prairie deer mouse	nr; WGS	33 (17)	–	33 (12)	6 (1)	–	6 (1)	1	1	5
	*Mi. ochrogaster*	Prairie vole	nr; WGS	7 (5)	1	1	1	–	1	1 (1)	1	2
Spalacidae	*Sp. leucodon*	Lesser mole rat	T-1022	1 (13)	(3)	–				1	–	–
	*N. galili*	Upper Galilee Mountains blind mole rat	nr; WGS	(6)	(1)	–	(1)	(1)	(1)	1	–	–
Dipodidae	*J. jaculus*	Lesser Egyptian jerboa	T-0552; nr; WGS	28 (7)	–	–				2	–	–
Anomaluridae	*Anomalurus* sp	Scaly-tailed squirrel	T-1787	–	(3)	3 (3)				1	–	
Pedetidae	*Ped. capensis*	South Africa springhare	T-3278							1	–	
Heteromyidae	*D. ordii*	Kangaroo rat	WGS	–	–	65 (28)	(1)	–	1	2	–	–
Caviidae	*Ca. porcellus*	Guinea pig	En; WGS	–	(1)	26 (45)	8 (1)	2 (1)	2	1	–	–
Chinchillidae	*Ch. lanigera*	Long-tailed chinchilla^b^	nr	–	–	21					–	
Octodontidae	*O. degus*	Degu^b^	nr	–	2	47				1	–	
Bathyergidae	*H. glaber*	Naked mole rat	nr; WGS	–	(1)	10 (26)	–	–	1	1	–	
Sciuridae	*I. tridecemlineatus*	Thirteen-lined ground squirrel	En; WGS	–	(2)	–	1	1	1	1	–	–
	*S. vulgaris*	European red squirrel	T-RT3	–	1 (6)	–		1				–

NOTE.—^a^Family-ABDE (exon 3), family-C, H2-MV intact and pseudogenized (in brackets) sequences are identified by BLAST search on GenBank whole genome shotgun sequence (WGS), NCBI nonredundant sequence (nr) and Ensembl sequence (En) databases or obtained by molecular cloning of genomic DNA extracted from tissue samples (T- prefix). Cells with no values refer to not-searched sequences. The symbol “–” refers to searched but not identified sequences. Mouse and rat sequences are from Young and Trask (2007).

^b^Pseudogenes are not indicated.

Genomic DNA was isolated by digestion of tissues with proteinase-K and SDS followed by phenol extraction (Sambrook et al. 1989). To amplify family-A, family-C V2Rs, and H2-Mv genes, degenerate primers were designed within conserved regions (supplementary table S1, Supplementary Material online).

PCR conditions were as follows: an initial denaturation step of 5′ at 95 °C, followed by 40 cycles of 30″ at 95 °C, the annealing of 45″ at 55 °C–60 °C, the extension of 1′ at 72 °C, and a final step of 5′ at 72 °C. A second amplification of 20–35 cycles was occasionally required. The band of interest was excised from the agarose gel and purified using the Qiagen gel extraction kit. Normally, the results of four separate amplifications were pooled for cloning. Purified products were cloned using the pGEM-T Easy Vector System (Promega) or Topo TA cloning kit (Invitrogen).

For each species, a minimum of 50 colonies were sequenced for family-A and family C analysis.

### RT-PCR

RNA was extracted from fresh tissues of C57BL mice and purified using Trizol reagent (Invitrogen Milano, Italy). About 2 μg of total RNA served as template for oligo-dT primed first strand cDNA synthesis with Im-Prom-II Reverse Transcriptase (Promega, Milano, Italy). PCR was performed in Mastercycler Personal (Eppendorf, Milano, Italy) using AmpliBiotherm DNA polymerase, 3 mM MgCl2, 0.2 mM for each dNTPs, and 200 pmol forward/reverse target-specific oligonucleotide primers. Cycling parameters consisted of an initial denaturation step (95 °C, 2 min) followed by 35 cycles, each of these included a denaturation (95 °C, 30 s), a primer annealing (50 °C, 30 s), and an extension (72 °C, 30 s) step. Reaction was completed by a final extension step at 72 °C for 5 min. Semi-quantitative analysis of RNA expression was performed on agarose gel after electrophoresis using the NIS-Elements Advanced Research software (Nikon, Firenze, Italy).

PCR primer pairs were designed to amplify family-C V2Rs (supplementary table S1, Supplementary Material online). The expected amplified sequences encompass exon 3 and exon 4 and corresponded to the C-terminal region of the extracellular domain of these receptors.

### In Situ Hybridization

The sequence encoding the family-B and the family-A6 probes of *S. vulgaris* was obtained by RT-PCR from VNO cDNA with primers shown in supplementary table S1, Supplementary Material online.

PCR products were cloned into pGEM-T Easy Vector and were subjected to sequence analysis. Specific antisense cRNA probes were obtained by using digoxigenin-labeled NTPs (Roche) starting from 2 μg of linearized DNA template. The reaction products were precipitated and resuspended in 200 μl of hybridization buffer and used at the working dilution of 1:1,000. Experiments were performed as previously described (Schaeren-Wiemers and Gerfin-Moser 1993), except that hybridization and washing procedures were performed at 63 °C unless otherwise indicated.

### Bioinformatics

Primers for V2R and H2-Mv amplification were assessed for their theoretical capacity to match with genomic sequences obtained from the current databases of rodents. Imperfect matches with degenerated primers were determined using the Fuzznuc program of the Emboss package (supplementary tables S2 and S3, Supplementary Material online). The position of mismatches along the primer sequence was determined by parsing the Fuzznuc output with a Perl script based on the IUPAC module of the Bioperl package (Ver. 1.006901) (supplementary figs. S1 and S2, Supplementary Material online).

The search for genes encoding V2Rs was conducted using vertebrate genomic sequences available in the Ensembl (http://www.ensembl.org/, last accessed January 8, 2015) and Genbank (http://www.ncbi.nlm.nih.gov/genbank/, last accessed January 8, 2015) databases. An initial tblastn search with the mouse V2R sequences was performed to identify and retrieve the genomic contigs containing V2R genes in the various species. Next, complete coding sequences and pseudogenes were determined using Genewise (Birney et al. 2004) through homology comparisons with a Hidden Markov Model (HMM) of V2R proteins. The V2R HMM was constructed using the Hammer package (Eddy 1998) with an alignment of ten manually curated full-length V2R sequences from mouse and rat. The full-length protein sequences extracted with this procedure are reported in the supplementary information. Protein sequence alignments were carried out with ClustalW 2.1 (Thompson et al. 2002). DNA sequence alignments were based on protein alignments using the Transalign program (Bininda-Emonds 2005). Phylogenetic analysis was performed with the neighbor-joining (NJ) algorithm implemented in ClustalW, and the maximum-likelihood method (Felsenstein 1981) implemented in the PHYML (Guindon et al. 2009) and RAxML ver. 7.7.8 (Izquierdo-Carrasco et al. 2011) programs. The SH-LIKE algorithm and GTRCAT substitution models were used for PHYML and RAxML analysis, respectively, while the Kimura model was used for the NJ analysis. The validity of the Kimura model for V2R phylogeny was tested by estimating the transition/transversion ratio of mouse V2R sequences (*T*_s_/*T*_v_ = 1.72) with the codeml program of the Paml package (Yang 1997). Trees were visualized and annotated with the FigTree program (http://tree.bio.ed.ac.uk/software/figtree/, last accessed January 8, 2015). The exon-3 and exon-5 sequences obtained by PCR amplifications were clustered using the cd-hit program (Fu et al. 2012). Sequences with <1% nucleotide differences were considered to be the same sequence, which could originate from alleles of the same locus or from PCR errors (Dehara et al. 2012). Sequences obtained from molecular cloning were trimmed by excluding the primer sequences. Multiple alignment encompassing database and PCR sequences were trimmed at the same length before phylogenetic analysis. Trees reconstructed with the NJ methods shown in the figures were consistent with the ML trees. The MHC tree was reconstructed with the ML method based on an untrimmed alignment. The detection of possible pseudogenes in the amplified exon-3 sequences was based on the identification of frame-shift or nonsense mutations. The putative intact sequences were compared with the closest mouse homologues with the codeml program to verify that the ratio of synonymous and nonsynonymous substitutions (*d*N/*d*S) was <1.

#### Immunohistochemistry

For immunohistochemistry, 2-month old FVB mice were deeply anesthetized with pentobarbital and transcardially perfused at room temperature with a solution containing 10% saturated picric acid, 2% paraformaldehyde in PBS for 5′ followed by a 50 ml of PBS. Tissues were then dissected, decalcified in EDTA 0.5 M pH 8.0 for 48–72 h at 4 °C, and cryo-protected in 30% sucrose at 4 °C overnight. Subsequently, tissues were included in OCT embedding solution (CellPath, UK) and frozen in liquid nitrogen cooled pentane. Cryostat-cut sections (20 μm) were treated with 0.5% sodium dodecyl-sulfate for 10 min, washed in PBS, prior to incubation with the primary antibody. For Vmn2r1/ChAT double staining, sections were incubated with an anti-Vmn2r1 antibody (1:100) (Silvotti et al. 2007) and an anti-ChAT antibody (1:25, developed in goat) (Ogura et al. 2011) in PBS solution containing 1% albumin, 0.3% Triton X-100 (blocking buffer) for 48 h at 4 °C. After washes, sections were first incubated with an Alexa488 conjugated anti-goat antibody for 2 h. After washes, sections were preincubated with blocking buffer containing 10% goat serum for 1 h before incubation with an Alexa568 conjugated anti-rabbit antibody. For Vmn2r1/IP3R3 double staining, sections were first incubated with both the anti-Vmn2r1 antibody and an anti-IP3R3 antibody (1:100, developed in mouse) (Elsaesser et al. 2005) for 48 h at 4 °C. Staining was visualized with an Alexa488 conjugated anti-rabbit antibody (Vmn2r1) and an Alexa568 conjugated anti-mouse antibody (IP3R3). For preabsorption controls, 5 µg of each anti-V2R antibody were incubated with 10 µg of the polypeptide against which the antibody was raised. Anti-IP3R3 was purchased from BD Transduction Laboratories, anti-ChAT from Millipore and the Alexa-conjugated antibodies from Molecular Probes- Life Technologies. Fluorescent images were obtained using a Zeiss fluorescent microscope.

All experiments were carried out on rodents and involved only the painless suppression of animals. The experiments comply with the Principles of Animal Care (publication no. 85-23, revised 1985) of the National Institutes of Health and with the current law of the European Union and Italy. The present project was approved by the Ethical Committee of the University of Parma: approval ID: 17/14, March 27, 2014.

### Accessions

Accession numbers of the sequences used in this study are reported in supplementary file S1, Supplementary Material online when not indicated in figures.

### Gene Nomenclature

The nomenclature of V2Rs was that proposed by Young and Trask (2007).

## Results and Discussion

The four gene families (ABCD) in which rodent V2Rs are classified (Yang et al. 2005; Shi and Zhang 2007; Young and Trask 2007) show different evolutionary histories. Family-C genes were found in the shark genome (*Callorhinchus milii*) (Grus and Zhang 2009), whereas our analysis revealed that family-B V2Rs were already established after the separation of Squamata, in a lineage leading to Monotremata (platypus). Family D could not be clearly identified in platypus by our analysis, but it was detected in the opossum genome (Didelphimorpha) (Young and Trask 2007) (supplementary fig. S3, Supplementary Material online). Our phylogenetic data also suggest that family-A V2Rs (V2RA) is the most recently derived family. A large gene group of V2RA was found in the armadillo (Cingulata) genome, suggesting that V2RA was established before the rodent separation from Laurasiatheria (supplementary fig. S3, Supplementary Material online).

**Fig. 1.—:**
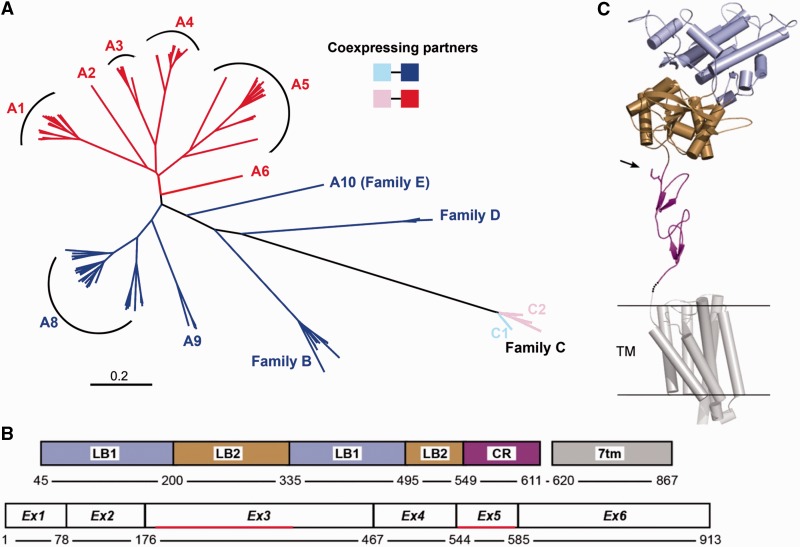
Structure and classification of V2Rs in the mouse. (*A*) Unrooted NJ phylogenetic tree based on multiple alignment of full-length V2R sequences of the mouse. Family-E V2R corresponds to the former subfamily A10. The combinatorial coexpression between subfamilies C1 and C2 and family-ABDE V2Rs is indicated by colors in the tree. (*B*) Domain and exon boundaries in the primary structure of V2Rs are indicated referring to the amino acid sequence of Vmn2r1. The red segments represent the regions of exon 3 and exon 5 considered for PCR amplification of family A and family C, respectively. (*C*) Predicted 3D structure of V2Rs. The structural model of mouse Vmn2r1 was constructed with the Phyre program using the coordinates of the extracellular region of the metabotropic glutamate receptor (PDB: 2E4W) and the transmembrane region of squid rhodopsin (PDB: 2ZIY) as templates. Portions corresponding to the ligand-binding 1 (LB1), ligand-binding 2 (LB2), cysteine-rich (CR), and transmembrane (7tm) domains as shown in (*B*) are represented in different colors. Arrow points to the amino acid position in the CR domain used to differentiate between subfamily C1 and C2.

In the mouse where a striking expansion and diversification of V2Rs occurred, V2RA reportedly includes nine subfamilies split into two groups, namely subfamily A1-6 and subfamily A8-10 (with subfamily A7 exclusively present in rat) (fig. 1*A*) (Yang et al. 2005). Subfamily A10 represents the basal branch of V2RA (Silvotti et al. 2011). While V2RA-subfamilies show an average identity >40% among each other, the mouse A10 gene shows an average identity of <40% with other V2RA subfamilies, which is comparable with that of the closest external V2R branch (family B) (supplementary table S4, Supplementary Material online). Moreover, in contrast to all the other V2RA-subfamilies, orthologues of A10 were already present in progenitors of mammal basal species such as elephant and tenrec (Afrotheria) (supplementary fig. S3*A*, Supplementary Material online). Thus, in this study, we classified this subfamily as a separate branch, which we have named *Family E*. Furthermore, as all family-E and family-D sequences reported in the available mammalian databases are incomplete, we reconstructed these genes in mouse and rabbit in order to establish that these V2R families included putatively functional genes (supplementary file S2, Supplementary Material online).

Because we recently proposed that in Muridae (mouse and rat), V2RA, and family-C V2Rs (V2RC) underwent a specific expansion originating the coexpressing subfamily A1-6 and subfamily C2 (Silvotti et al. 2011), the first question we asked was when this phylogenetic event occurred throughout the evolution of Rodentia.

### Expansion of family-A V2Rs in Rodentia

**Fig. 2.—:**
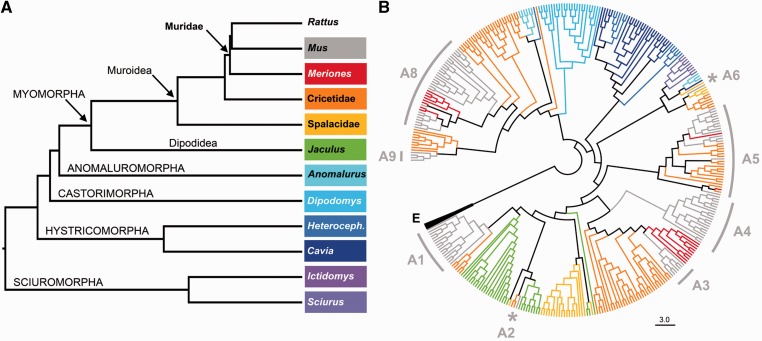
Evolutionary history of family-A V2Rs in rodents. (*A*) The phylogenetic tree of Rodentia highlighting the five main suborders (Myomorpha, Anomaluromorpha, Castorimorpha, Hystricomorpha, and Sciuromorpha), the Muroidea and Dipodidea clades, the Muridae family and representative genera in which this order is divided (Hedges, et al. 2006; Blanga-Kanfi et al. 2009). (*B*) Radial dendrogram showing the evolutionary origin of the A-subfamily. The NJ phylogenetic tree is based on a multiple alignment of family-A DNA sequences (exon 3) from representative species of Rodentia. The mouse V2RA subfamilies are indicated. The tree is rooted using family E (E) as an outgroup. Branches are stained according to species classification as shown in panel *A*. Only representative sequences for each species are included in the tree. The complete repertoire of the analyzed sequences with branch lengths, tip labels and bootstrap supports are shown in supplementary fig. S5, Supplementary Material online.

To characterize the V2R lineages and identify the origin and expansion of subfamily A1-6, we analyzed the V2R sequences in representative species of Rodentia (fig. 2*A* and table 1). Due to their complex gene structure (six exons) (fig. 1*B*), the complete reconstruction of all rodent V2R genes is difficult to obtain. Thus, to build a correct phylogenetic tree, we asked whether single exon sequences could be representative of the full-length genes. To answer this question, we first aligned the DNA sequences of all mouse intact V2RA and then we created independent phylogenetic trees with each exon. Each tree was compared with that obtained with the alignment of the full-length V2RA sequences. Our results indicate that the phylogenetic tree reconstructed from exon 3, encompassing most of the ligand binding domain of V2Rs (fig. 1*B* and 1*C*), shows a very similar topology with that obtained by aligning the full-length V2RA sequences (supplementary fig. S4, Supplementary Material online).

On these grounds, we approached the problem in two ways. In species with a draft genomic coverage (table 1), the exon 3 of V2RA genes was identified and reconstructed based on blastn similarity with V2R sequences of mouse and rat. The novel sequences obtained from each species were used as queries to conduct further blastn analysis.

For rodent species without a genomic coverage but important for our analysis, PCR experiments with specific primer pairs (see Methods and supplementary tables S2 and S3, Supplementary Material online) were performed to amplify the exon 3 of family A genes from tissue-extracted genomic DNA (table 1 and supplementary table S1, Supplementary Material online). To increase the number of novel V2RA sequences that could be obtained by this approach, the genomic DNA of each species was amplified at different annealing temperatures and the resulting amplicons were pooled and subcloned for sequencing into two different vectors. Using this molecular strategy, we were able to identify 20–25 novel sequences for each species (with the exception of *Anomalurus* sp. and *S. vulgaris*) which were considered to be sufficient for the aim of this study (table 1). To infer the evolutionary history of rodent V2RA, we performed a phylogenetic analysis of the sequences obtained by blastn search and cloning approach. Although we tentatively distinguished between putatively functional genes and pseudogenes on the basis of exon-3 sequences, all sequences were included in the analysis, as pseudogenes provide information on the origin of the V2RA-subfamilies. The consensus topology was verified and supported by maximum likelihood and NJ algorithm. The V2R phylogenetic tree obtained from this analysis was compared with that of the most commonly accredited evolutionary tree of Rodentia (fig. 2, supplementary fig. S5, and file S3, Supplementary Material online) (Hedges et al. 2006; Blanga-Kanfi et al. 2009).

The first observation from our analysis suggests that rodent V2RA were established from few ancestral families. This is evident in *I. tridecemlineatus and S. vulgaris* (Sciuromorpha), which represent the basal branch of Rodentia. These two distantly related squirrel species revealed a limited number of V2RA, highly pseudogenized, and all clustering with subfamily A6 (table 1, fig. 2*B*, and supplementary fig. S5, Supplementary Material online). Hence, it is most likely that the ancestor of rodent A1-5 subfamilies was established after the split of Rodentia from Lagomorpha (Huchon et al. 2002; Hedges et al. 2006).

A second observation shows that rodent V2RA tend to form private clades in distantly related species or semiprivate clades (with few orthologues) in closely related species as also reported for other lineages (Shi and Zhang 2007; Grus and Zhang 2008). The occurrence of gene duplication following species separation is a condition that makes it difficult to define orthologous relationships (Sonnhammer and Koonin 2002). Moreover, the subfamily classification is also complicated by the presence of several pseudogenes (as in Spalacidae) (fig. 2*B*). Thus, the inclusion of sequences into a specific mouse subfamily was based on their monophyletic grouping in the tree. In *Anomalurus* sp. (Anomaluromorpha), we found no evidence of V2Rs clusterizing with subfamily A1-5 V2Rs (V2RA1-5). In this species, regardless the use of specific additional primers (supplementary table S1, Supplementary Material online), we obtained only a limited number of distinct sequences (fig. 2*B* and supplementary fig. S5, Supplementary Material online). This is indicative of a small V2R repertoire in this species although it remains possible that *Anomalurus*, as previously discussed, formed phylogenetically distant V2RA sequences which could not be amplified with our primers.

From our analysis*, J. jaculus* (Dipodidae), *Sp. leucodon*, and *N. galili* (Spalacidae) all have receptors in the A1-5 group and thus they are candidates to represent the species most distantly related to mouse in which this clade appeared (table 1). In these rodent families, given their crucial evolutionary position, sequences of exon 3 were obtained from both the molecular and bioinformatic approach (supplementary fig. S6, Supplementary Material online and table 1). Moreover, the analysis extended to mouse/rat closely related species such as *M. unguiculatus*, C. *griseus, Mes. auratus, P. maniculatus*, and *Mi. ochrogaster* confirmed that all these rodent species have orthologues of V2RA1-5 (fig. 2*B*, supplementary fig. S5, Supplementary Material online, and table 1).

### Phylogenetic Origin of C-Subfamilies

**Fig. 3.—:**
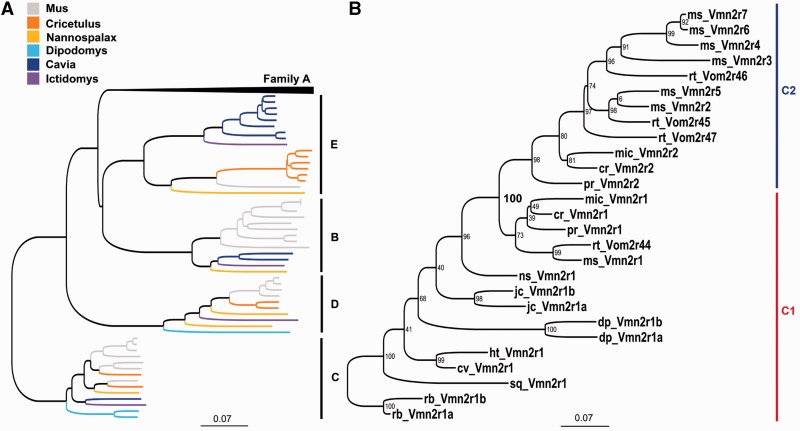
Phylogeny of family-BCDE V2Rs in rodents. (*A*) Rooted NJ phylogenetic tree based on multiple DNA sequence alignments of full-length family-BCDE V2Rs. DNA sequences are reconstructed from the available genome databases. Family-A branches are collapsed. The tree is rooted with rabbit family-C V2Rs as outgroups. Bootstrap value of the subfamily C1 and C2 separation node is shown in bold. Scale bar: mean number of base substitutions per site. (*B*) Maximum likelihood phylogenetic tree based on the multiple amino acid alignment of family-C V2Rs reconstructed from the available databases of rodents. For species abbreviations refer to Methods. Bootstrap values are shown. Scale bar: mean number of amino acid substitutions per site.

Family-C represents the basal V2R branch in rodents (fig. 3*A*). In mouse and rat, V2RC genes underwent a process of duplication and inversion starting from a single gene that is present in most rodent and nonrodent species (Silvotti et al. 2011). Since, in Muridae, all inverted/duplicated family-C genes cluster in subfamily C2, we previously proposed that the establishment of this subfamily might coincide with this genetic event (Silvotti et al. 2011). Indeed, the analysis of the family-C locus in *C. griseus* (Cricetidae) supports this finding as this species has the same genomic organization as rat and mouse (Silvotti et al. 2011). Our analysis extended to *J. jaculus* (Dipodidae), suggested that duplication/inversion did also occur; here, however, the duplicated/inverted family-C gene clustered with the subfamily-C1 group (fig. 3*B*). This indicates that either the progenitor of the Dipodidae species independently duplicated and inverted the family-C gene or that the establishment of subfamily C2 occurred later in rodent evolution.

**Fig. 4.—:**
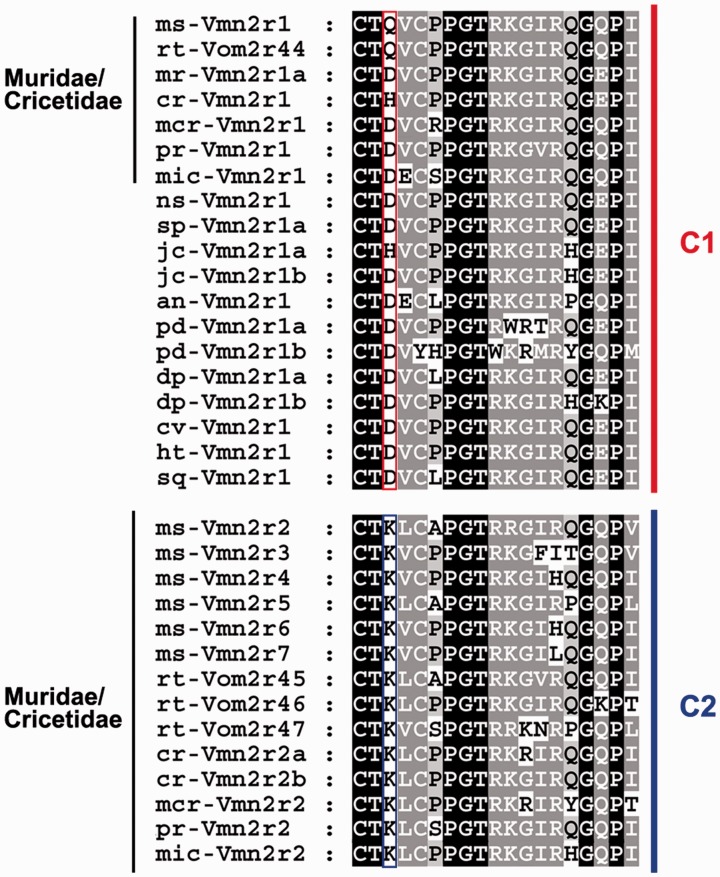
Multiple alignment of family-C V2Rs in rodents. The amino acid substitution (Q/H/D → K) in exon 5 that differentiates subfamily-C1 from subfamily-C2 V2Rs is encased by a red and blue rectangle, respectively. The position of this residue in the 3D structure of the protein is indicated in figure 1*C* with an arrow. For species abbreviations refer to Methods.

Thus, we supposed that the origin of subfamily C2 took place in ancestral lineages postdating the separation between Dipodidea and Muroidea (fig. 2*A*). To date this phylogenetic event, we considered the family of Spalacidae (*Sp. leucodon* and *N. galili*), which represents the basal branch of the muroid lineage. Our reconstruction of the complete V2RC gene in *N. galili* by blastn search against the whole genome shotgun sequence (WGS) and NCBI nonredundant sequence (nr) databases using mouse sequences as queries (Fang et al. 2014), indicates the presence of a single V2RC gene, split into two contigs (gi|605751882, gi|605715922), clustering with subfamily C1 (supplementary file S4, Supplementary Material online). To confirm that subfamily-C2 did not include spalacid sequences, we amplified the genomic DNA of *Sp. leucodon* with primers specific for the exon 5 of rodent V2RC genes (supplementary table S1, Supplementary Material online). The choice of exon 5 as a template for PCR amplification was the result of the sequence analysis of V2RC in all rodent and nonrodent species with a draft genome. In exon 5, we identified a single amino acid substitution that was likely to be a feature to discriminate subfamily-C1 V2Rs (V2RC1) from subfamily-C2 V2Rs (V2RC2). All V2RC2 genes so far identified invariably presented a lysine at position 552 in place of a glutamine, histidine, or aspartate (K 552 → Q/H/D, referred to mouse Vmn2r1) which are distinctive residues of all V2RC1 genes (fig. 4 and supplementary fig. S7, Supplementary Material online). The substitution is located in the cysteine-rich (CR) domain (fig. 1*C*) that is thought to play a role in transmitting the ligand-induced conformational change to the G-protein and in the receptor oligomerization process (Muto et al. 2007). The phylogeny as well as the sequence analysis of exon 5 in *Sp. leucodon* and *N. galili* confirm that Spalacidae did not evolve V2RC2 genes (fig. 4 and supplementary fig. S8, Supplementary Material online). Given the loss of the V2R repertoire and probably of the vomeronasal functions in these species, we cannot exclude that the spalacid progenitor inherited (and then completely lost) the duplicated/inverted V2RC1 gene which was observed in *Jaculus*.

### Phylogeny of H2-Mv in Rodents

**Fig. 5.—:**
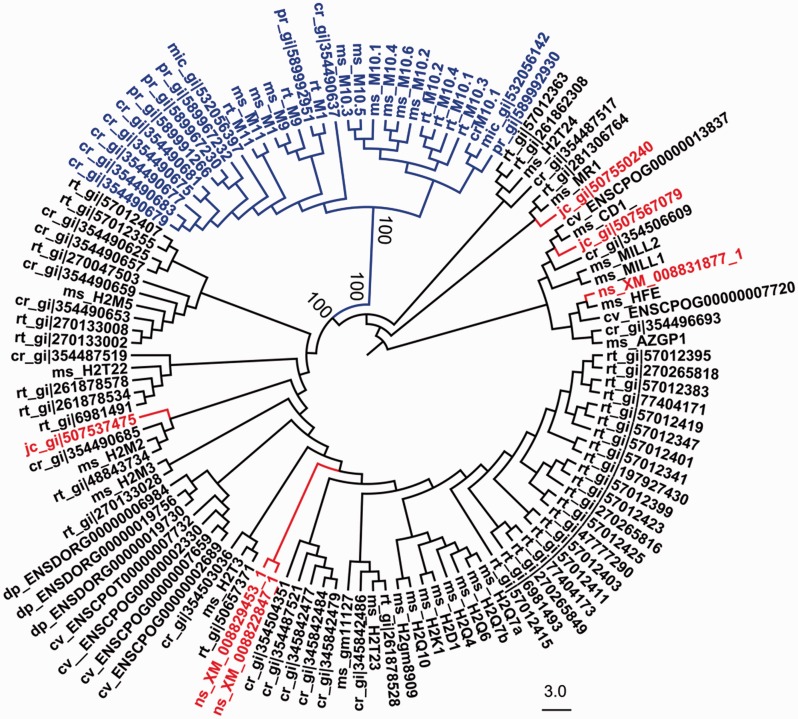
Phylogenetic reconstruction of H2-Mv molecules in rodent species. NJ phylogenetic tree based on protein alignment of Mhc, obtained from BLAST search with H2-Mv protein sequences against the available rodentia sequence databases (Ensembl and nr databases). H2-Mv branches are represented in blue whereas protein hits obtained from *J. jaculus* and *N. galili* databases are shown in red. Accession numbers are given for each sequence except for mouse (ms) and rat (rt). For species abbreviations refer to Methods. Bootstrap values are shown. Scale bar: mean number of amino acid substitutions per site.

In rat and mouse, the coexpression of V2RC2 and V2RA1-5 in a specific subset of VNO neurons is also related to the expression of nonclassical major histocompatility complex molecules (H2-Mv) (Silvotti et al. 2007; Ishii and Mombaerts 2011). These proteins are specifically expressed in the VNO and are phylogenetically distinct from classical major histocompatility complex (Mhc) molecules (Ishii et al. 2003; Loconto et al. 2003). Furthermore, H2-Mv are reportedly involved in pheromone detection (Leinders-Zufall et al. 2014). Thus, we asked whether the phylogenetic origin of H2-Mv was correlated with that of the coexpressing V2RA1-5 or V2RC2 that, respectively, date before and after the separation of Dipodidea and Muroidea. To establish this, we first searched for H2-Mv sequences in the rodent databases to generate a phylogenetic tree. Our tblastn analysis using rat and mouse queries against the WGS and nr databases of *C. griseus*, *P. maniculatus*, and *Mi. ochrogaster* revealed that orthologues of the mouse H2-Mv genes were already present in Cricetidae (*C. griseus*). An iterated tblastn search in the genomes of Spalacidae (*N. galili*), Dipodidae (*J. jaculus),* Heteromyidae (*D. ordii*), and Caviidae (*Ca. porcellus*) failed to identify H2-Mv sequences (fig. 5 and table 1). To further exclude the presence of H2-Mv genes in Spalacidae, which represent the basal branch of the Muroidea clade, we used a PCR approach to analyze the genome of *Sp. leucodon*. Degenerate primer pairs (H256 and H257) were designed based on the exon-2 sequence of H2-Mv annotated in the nr sequence databases of *Mu. musculus, R. norvegicus*, *and C. griseus* (supplementary tables S1 and S3, Supplementary Material online). As expected, these primers successfully amplified H2-Mv sequences in these species but they were unable to generate amplicons in all the other rodent species, including *Sp. leucodon* and *J. jaculus* (supplementary fig. S9, Supplementary Material online). We also designed less degenerate primers (H255) that matched most conserved H2-Mv regions of exon 4 but that were also predicted to match sequences of some Mhc type-I subclasses different from H2-Mv (supplementary tables S1 and S3, Supplementary Material online). Control experiments indicated that our primers indeed amplified both mouse H2-Mv and Mhc sequences. As expected, primers H255 generated amplicons in *Sp. leucodon* (and in all of the other rodent species) that were cloned and sequenced (supplementary fig. S9, Supplementary Material online). The analysis of 86 clones yielded 16 different sequences, all encoding Mhc, phylogenetically distinct from H2-Mv (supplementary fig. S10 and file S5, Supplementary Material online). Thus, our data strongly support the hypothesis that H2-Mv coevolved with V2RC2 genes in an ancestor of the Cricetidae species.

### Differential Expression Pattern of Subfamily-C1 and -C2 V2Rs in Extra-Vomeronasal Tissues

One important issue is understanding why family C has diversified and expanded to establish the phylogenetically recent subfamily C2 in Muridae and Cricetidae species. Although V2RC are typically defined as vomeronasal receptors, they are not phylogenetically linked to the origin of the VNO. Fish, that did not develop this organ, express V2RC genes in the olfactory epithelium (MOE) (DeMaria et al. 2013). Interestingly, V2RC genes are exclusively expressed in the MOE of amphibians although they have a functional VNO (Syed et al. 2013). In phylogenetically more recent species such as mouse and rat, a V2RC1 gene, but not any other V2R, was detected in the olfactory-related sensory cells of the Grueneberg ganglion (Roppolo et al. 2006; Fleischer et al. 2007). All these observations suggest that V2RC may subserve to broader chemosensory functions, possibly common to many species (Mamasuew, Hofmann, Breer et al. 2011, Mamasuew, Hofmann, Kretzschmann, et al. 2011; Liu et al. 2012; Hanke et al. 2013).

**Fig. 6.—:**
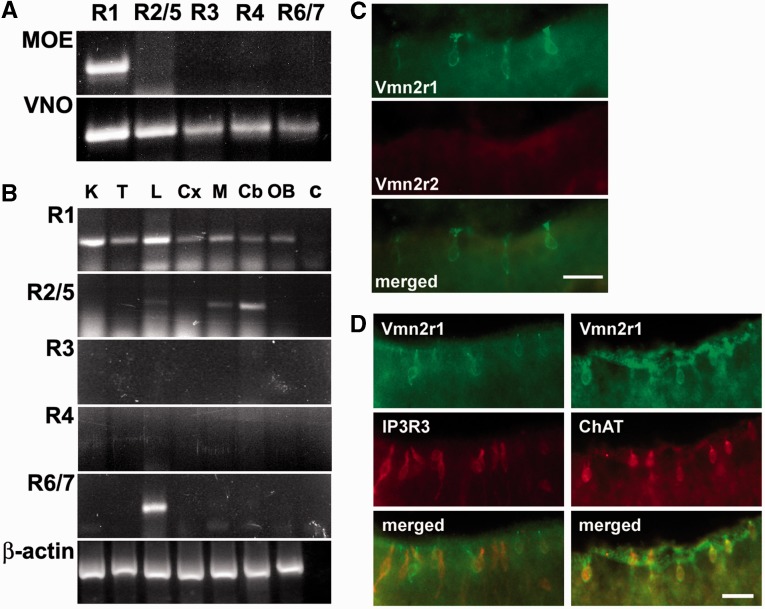
Tissue distribution and expression of family-C V2Rs in the mouse. (*A*) RT-PCR with mouse family-C specific primers in the VNO and MOE and (*B*) in extra-vomeronasal tissues. MOE, main olfactory epithelium; K, kidney; T, testis; L, lung; Cx, cortex, M, midbrain; Cb, cerebellum; OB, olfactory bulb; C, negative control. Vmn2r1 (R1 in the panel) clusters with subfamily C1 V2Rs whereas Vmn2r2/5 (R2/5), Vmn2r3 (R3), Vmn2r4 (R4) and Vmn2r6/7 (R6/7) with subfamily C2 V2Rs. The coding sequence of β-actin was used as a control of amplification. (*C*) Double label immunohistochemistry on tissue sections of the olfactory epithelium with antibody against Vmn2r1 (subfamily C1) and Vmn2r2 (subfamily C2). Scale bar, 20 µm. (*D*) Immunohistochemical localization of Vmn2r1 in the olfactory epithelium. Double label immunohistochemistry was performed with an antibody against Vmn2r1 in combination with antibodies against IP3R3 or ChAT. Scale bar, 20 µm.

On these grounds, we hypothesized that subfamily-C2 establishment and expansion was related to a functional requirement of vomeronasal specificity for V2RC. Thus, taking into account the mouse as a model, we analyzed V2RC expression in different tissues. To achieve this purpose, we reverse-transcribed mouse RNA from different tissues and amplified the cDNA with primers specific for each family-C gene (Vmn2r1-7). Vmn2r1 (subfamily C1) amplicons were identified in all tissues we have tested including MOE (figs. 6*A* and 2*B*). In contrast, Vmn2r2 (subfamily C2) expression was only revealed in cerebellum, subcortical regions, and lung (fig. 6*B*) whereas Vmn2r6/7 (subfamily C2) expression was exclusively detected in lung. No expression of the Vmn2r3 (subfamily C2) gene was identified in any of the tested tissues (fig. 6*B*). All PCR products were subcloned for sequence confirmation. We also performed PCR reactions with different cDNAs and primer pairs that matched family-ABDE V2R sequences. No PCR products of the predicted molecular weight were detected in all tested tissues.

Since V2RC amplicons were detected in different tissues, we assayed them for protein expression by immunohistochemistry using antibodies against family-C V2Rs. By staining tissue sections with an antibody raised against Vmn2r1, we found a specific labeling of a cellular subset located in the upper part of MOE lining above the sustentacular cell layer. No staining of these cells was revealed with any other subfamily-C2 antibodies (Vmn2r2-7) (fig. 6*C* and supplementary fig. S11, Supplementary Material online). Similar but isolated Vmn2r1 positive cells were also identified in trachea but no other tissues showed Vmn2r1 immunoreactivity (supplementary fig. S12*A*, Supplementary Material online).

Both olfactory and tracheal Vmn2r1-positive cells appeared equipped with microvilli, therefore we asked whether they corresponded to the microvillous cells previously described by different authors (Elsaesser et al. 2005; Lin et al. 2008; Krasteva et al. 2011). A first subset of olfactory microvillous cells was shown to express molecules of the phosphatidyl-inositol triphosphate pathway such as PLC-beta-2, type III inositol 1,4,5-triphosphate receptor (IP3R-3) and the transient receptor potential channel C6 (TRPC6) (Elsaesser et al. 2005). These cells respond to specific odorants and are probably involved in the processes of neural regeneration of the MOE (Montani et al. 2006; Hegg et al. 2010; Jia et al. 2013). To prove the correlation between this microvillous cellular subset and our Vmn2r1 positive cells, we performed double label immunohistochemistry with an antibody raised against IP3R-3. IP3R-3 staining revealed that 90% of the Vmn2r1 positive cells did not show IP3R-3 immunoreactivity at detectable levels (fig. 6*D*). Thus, the microvillous cells described by Elsaesser et al. (2005) did not express the vomeronasal receptor Vmn2r1. A second subtype of microvillous cells, identified in the olfactory and tracheal epithelium, was reported to express the transient receptor potential channel M5 (TRPM5) (Lin et al. 2008; Krasteva et al. 2011). These cells are cholinergic as they also express the signature markers of choline acetyltransferase (ChAT) and the vesicular acetylcholine transporter (VAchT) (Ogura et al. 2011). The olfactory TRPM5/ChAT/VAchT-expressing microvillous cells are believed to respond to xenobiotic chemicals or to thermal stimuli, releasing acetylcholine to modulate activities of the olfactory sensory neurons (Ogura et al. 2011). In contrast, in trachea, microvillous cells expressing ChAT are solitary chemosensory cells that also express bitter receptors and sense bitter compounds via a cholinergic pathway initiating an aversive reflex (Krasteva et al. 2011). For this reason, we assessed whether these two cellular subsets may also express Vmn2r1. Double label immunohistochemistry with anti-Vmn2r1 and anti-ChAT antibodies clearly indicated that these molecules indeed colocalized (fig. 6*D* and supplementary fig. S12*B*, Supplementary Material online). Thus, V2RC1 expression in the olfactory and tracheal epithelium is associated with a specific subset of excitable nonneuronal cells that responds to sensory stimuli different from those typically pheromonal (Ogura et al. 2011). In contrast, all V2RC2 appeared to be exclusively expressed in sensory neurons of the VNO. Thus, the expansion of V2RA1-5 genes in muroid species probably required a more subtle functional specificity for the coexpressing V2RC.

## Conclusions

In this study, we have analyzed the evolutionary history of V2Rs in rodent species supported by the analysis of their expression pattern. We first observed that the last rodent common ancestor exhibited a very small repertoire of V2Rs, probably restricted to one or few receptors for each ABCDE-family (table 1). This minimal repertoire remained almost unmodified in the extant species of Sciuridae (basal branch of Rodentia) such as *I. tridecemlineatus* and *S. vulgaris* (table 1). Recently, a correlation was proposed between the reduction in the number of V2R expressing neurons in the VNO and the occurrence of sexual dimorphism in mammalian species (Suarez et al. 2011). Our observations do not support this hypothesis, as we found a similarly limited V2R repertoire in both the dimorphic species of *I. tridecemlineatus* (ground squirrel) and the monomorphic species of *S*. *vulgaris* (tree squirrel) (Shulte-Hostedde 2007; Mateju and Kratochvil 2013). In this latter species, we also observed a striking reduction of the V2R expressing neuronal layer by in situ hybridization and immunohistochemical studies on the VNO (supplementary fig. S13, Supplementary Material online). In addition, as inferred from the annotation in the nr databases, rodent species which are reportedly dimorphic such as the *Octodon degus* and *Chinchilla lanigera* (Hystricomorpha) (Lammers et al. 2001; Shulte-Hostedde 2007; Suarez and Mpodozis 2009) have evolved a consistent repertoire of V2Rs (table 1 and supplementary file S1, Supplementary Material online). Thus, in contrast to V1Rs, where a correlation exists between the size of this receptor group and adaptive situations (Wang et al. 2010), the functional significance of the expansion or loss of V2Rs in mammalian species is not clearly established. Yet, it is noteworthy that the majority of the identified V2RA sequences (exon 3) of *Sp. leucodon* and the whole V2R repertoire (with the exclusion of V2RC) of *N. galili* is characterized by pseudogenes (table 1). Because the Spalacidae family only includes fossorial species, it is possible that the constrained sociosexual behavior in the subterranean environment resulted in the loss of some if not all the vomeronasal functions (Smith et al. 2007). In line with this hypothesis, a strong pseudogenization of V2RA genes is also evident in *H. glaber*, another burrowing rodent of the Hystricomorpha suborder (table 1).

**Fig. 7.—:**
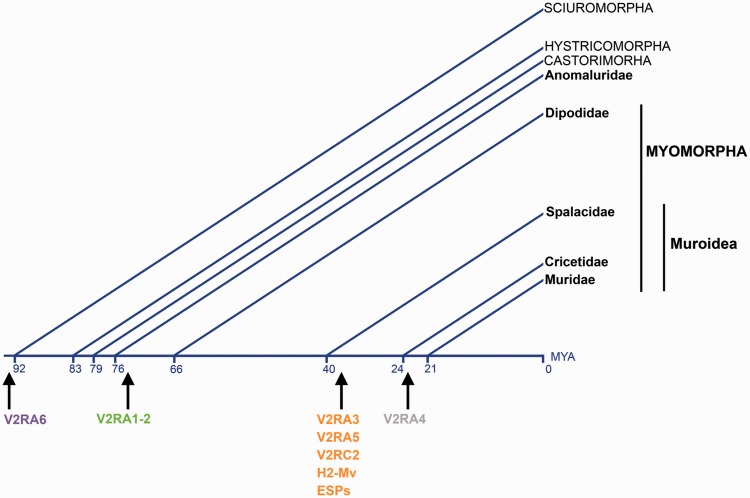
Origins of the pheromone-related genetic components in the basal VNO neurons. Divergence times, in MYA, are based on Hedges et al. (2006).

Our study also elucidates that the phylogenetically recent V2RA1-5 group was established in myomorphan species, most likely from an ancestral receptor of the subfamily A6, after the split of Anomaluridae (Anomaluromorpha) and Dipodidae, an event that approximately occurred about 76 million years ago (MYA) (fig. 7) (Hedges et al. 2006). In contrast, the origin of the coexpressing (in rat and mouse) V2RC2 and H2-Mv occurred in a common ancestor of Cricetidae and Muridae, then after the appearance of the V2RA1-5 genes (fig. 7) (Hedges et al. 2006). This finding may have interesting implications on the regulatory mechanisms underlying V2R gene expression that are believed to occur according to a temporal succession in the developing VNO neurons. Since in mouse, a single V2RA1-5 gene is expressed first and drives the expression of a V2RC2 gene (Ishii and Mombaerts 2011), it is conceivable that, in *J. jaculus* (Dipodidea), that possesses V2RA1-5 but not V2RC2 genes, different regulatory mechanisms (genes encoding transcription factors, for example) have evolved to address the coexpression of subfamily A1-5 with the extant subfamily-C1 genes.

The obligatory coexpression of V2RC with non-V2RC genes in the VNO also predicts a common evolutionary history for these two groups of receptors. Indeed, we generally observed that the incidence of pseudogenization in non-V2RC genes highly correlates with that of V2RC genes in most mammalian species with the exception of *N. galili* (Spalacidae) whose V2R repertoire, according to our analysis, is limited to one intact subfamily-C1 gene (supplementary fig. S3, Supplementary Material online). The presence of a single V2R gene, as observed in Spalacidae, could also be explained with the loss of the VNO basal layer in this species and the expression of V2RC1 in tissues different from the VNO, as reported in this and previous studies (Fleischer et al. 2007; DeMaria et al. 2013; Syed et al. 2013).

Our phylogenetic analysis within the A1-5 group shows that subfamily A1-2 V2Rs (V2RA1-2) are first found in Dipodidae (*J. jaculus*) and Spalacidae (*Sp. leucodon* and *N. galili*), whereas subfamily-A3 and subfamily-A5 V2Rs (V2RA1-2 and V2RA5) are detected in Cricetidae (*C. griseus, Mes. auratus, P. maniculatus*, and *Mi. ochrogaster*). Finally, subfamily A4 probably evolved in murid species (rat and mouse) (supplementary fig. S5 and table S5, Supplementary Material online). As largely reported, V2Rs are supposed to primarily respond to peptide or protein pheromones (Chamero et al. 2007; Leinders-Zufall et al. 2014). However, the only ligand which was unequivocally demonstrated to bind to a specific V2R is a member of the ESP family, namely ESP1 (Kimoto et al. 2005, 2007). The mouse receptor for ESP1, V2Rp5 (Vmn2r116) clusterizes with V2RA3 (Haga et al. 2010; Abe and Touhara 2014) (supplementary fig. S5, Supplementary Material online). Interestingly, our analysis shows that V2RA3 and ESPs are probably evolutionary correlated, both being established in Cricetidae but absent in Spalacidae species (supplementary table S5, fig. S14, Supplementary Material online, and fig. 7).

In conclusion, data presented here contribute to clarify the evolutionary history of V2Rs in the Rodentia order starting from the most basal species, focusing on the diversification and expansion processes underlying these receptors in the superfamily of Muroidea which includes the most speciose families in the animal kingdom. Moreover, functional correlations have been highlighted between the expansion of these receptors and their expression in the VNO and in other tissues. This evolutionary analysis will also provide useful information for the search and identification of protein ligands for V2Rs.

## Supplementary Material

Supplementary figures S1–S14, tables S1–S5, and files S1–S5 are available at *Genome Biology and Evolution* online (http://www.gbe.oxfordjournals.org/).

Supplementary Data
