# Supplementary material for: Evolution of Spatially Coexpressed Families of Type-2 Vomeronasal Receptors in Rodents
Source: Genome Biol Evol. 2014 Dec 23;7(1):272–85. doi: 10.1093/gbe/evu283 (PMC4316634; doi:10.1093/gbe/evu283)
Supplement: Supplementary Data [file supp_7_1_272__index.html]

Evolution of Spatially Coexpressed Families of Type-2 Vomeronasal Receptors in Rodents — Supplementary Data 

# Evolution of Spatially Coexpressed Families of Type-2 Vomeronasal Receptors in Rodents

## Supplementary Data

files

**Files in this Data Supplement:**

- Supplementary Data - pdf file
- Supplementary Data - pdf file
- Supplementary Data - pdf file
- Supplementary Data - pdf file
- Supplementary Data - pdf file
- Supplementary Data - pdf file
- Supplementary Data - pdf file
