# Supplementary material for: Evolution of Spatially Coexpressed Families of Type-2 Vomeronasal Receptors in Rodents
Source: Genome Biol Evol. 2014 Dec 23;7(1):272–85. doi: 10.1093/gbe/evu283 (PMC4316634; doi:10.1093/gbe/evu283)
Supplement: Supplementary Data [file supp_evu283_Supplementary_Figures.pdf]

Supplementary Figure S1

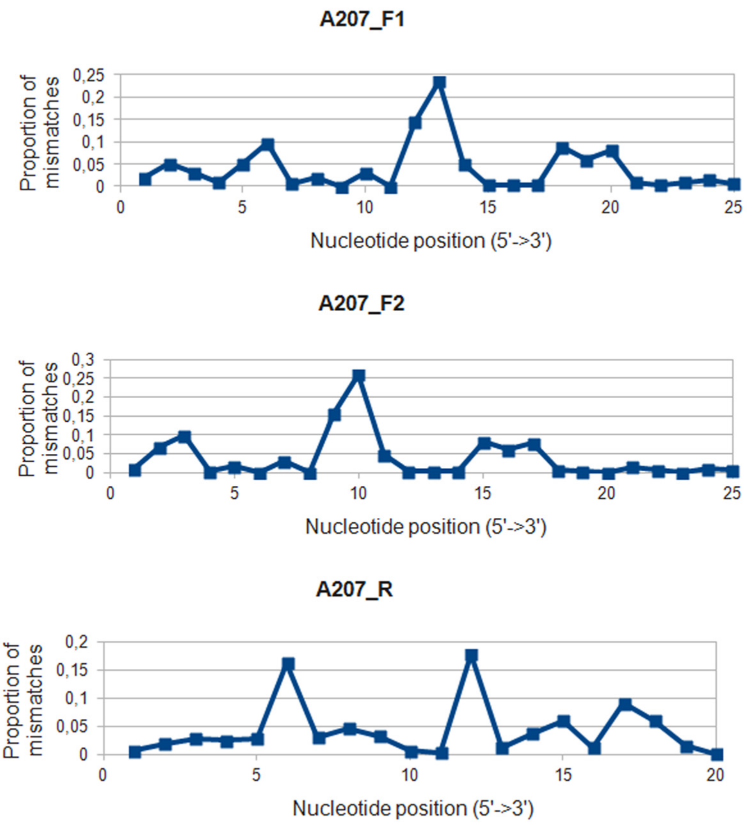

Supplementary Figure S1. Mismatch profiles of the family-A primers. The proportion and position of mismatches are determined by matching primers A207 to rodent V2RA sequences identified from the genome assembly.

Supplementary Figure S2

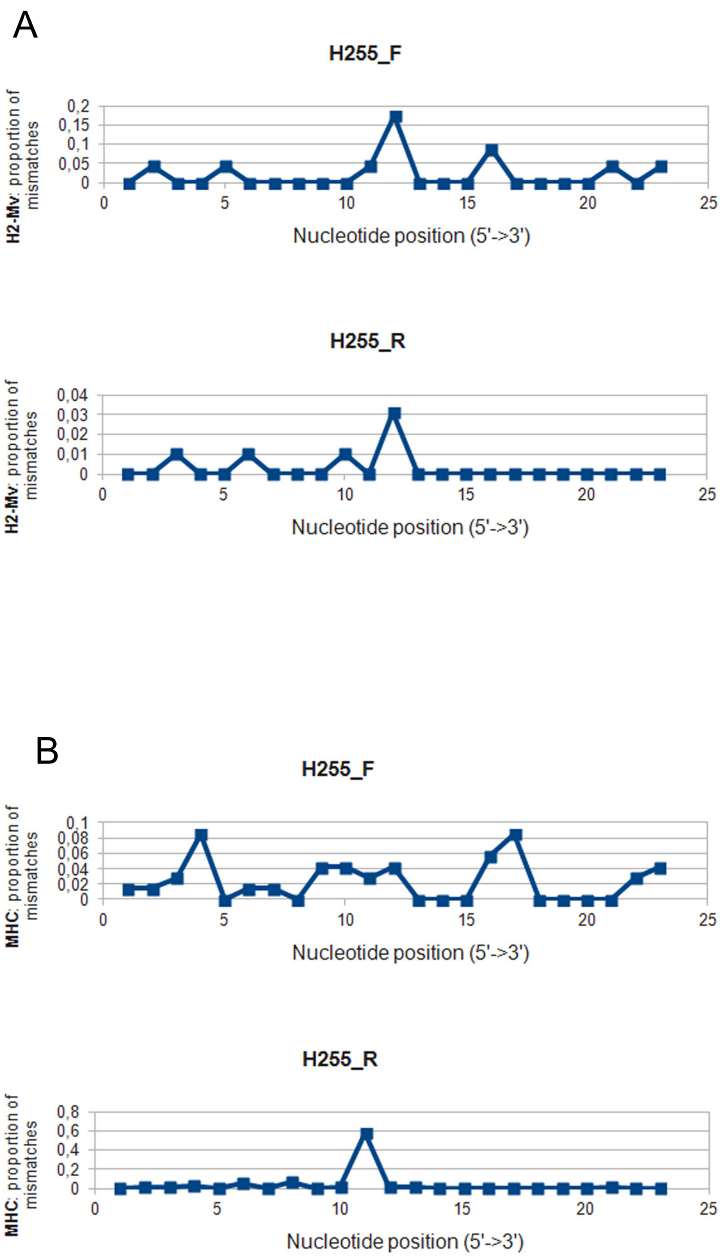

Supplementary Figure S2. Mismatch profiles of the H2-Mv primers. (A) The proportion and position of mismatches are determined by matching primers H255 to mouse, rat and C. griseus H2-Mv genes identified from the genome assembly. (B) The proportion and position of mismatches are determined by matching primers H255 to mouse, rat and C. griseus Mhc genes (except H2-Mv genes) identified from the draft genome assembly by blastn search with mouse H2-Mv sequences as queries.

Supplementary Figure S3

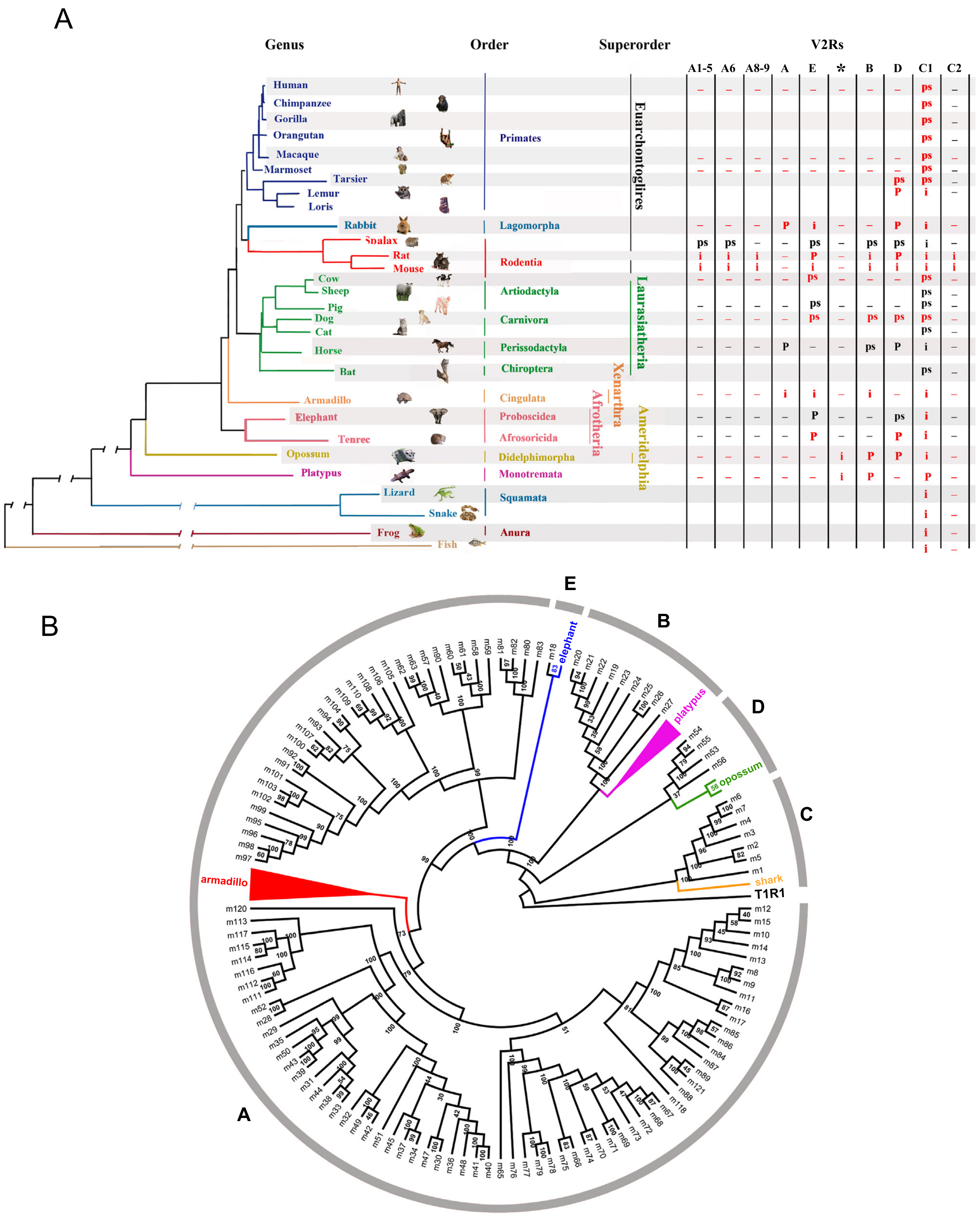

Supplementary Figure S3. Rodent V2R families (ABCDE) in tetrapods and fish. (A) Shown are familiy-ABCDE V2R intact genes (i) or partially reconstructed genes (P) and pseudogenes (ps) identified by blastn and tblatn searches in WGS databases (black characters), annotated in nr databases or reported in literature (red characters) (Brykczynska, et al., 2013; Dong, et al., 2012; Grus, et al., 2007; Grus and Zhang, 2009; Hohenbrink, et al., 2013; Shi and Zhang, 2007; Syed, et al., 2013; Yang, et al., 2005; Young and Trask, 2007). The '—' symbol indicates that sequences are not detected in our blastn and tblastn analysis on the genome drafts; empty cases indicate that search is not performed. Asterisk indicates independent V2R subfamilies external to the family-E branch. The tree is adapted from Francia and colleagues (Francia, et al., 2014). (B) Neighbor-joining tree based on protein multiple alignments of mouse V2Rs (black branches) and V2Rs of other tetrapod and fish species (colored branches) traces the phylogenetic origin of the rodent ABCDE-V2R families. The mouse sweet taste receptor T1R1 (Tas1r1) is used as an outgroup. Bootstrap support values as well as V2R families are indicated.

Supplementary Figure S4

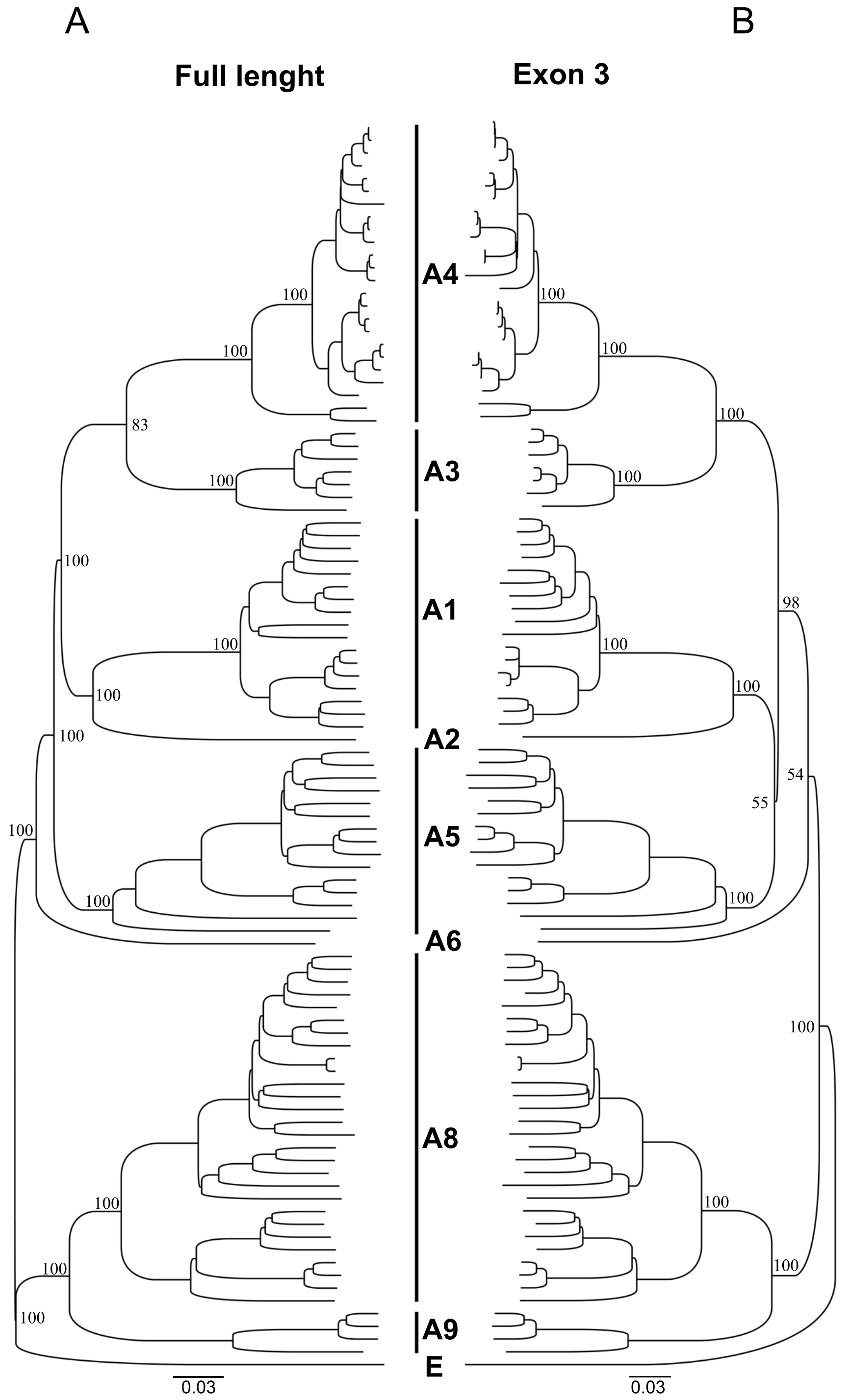

Supplementary Figure S4. Phylogenetic reconstruction of mouse family-A V2R genes. Comparison of Neighbor-joining phylogenetic trees based on a multiple alignment of (A) full-length and (B) exon-3 V2R DNA sequences showing similar topology and bootstrap values. Trees are rooted using family E as an outgroup. Branch length is proportional to the genetic distance as indicated by the scale bar.

Supplementary Figure S5

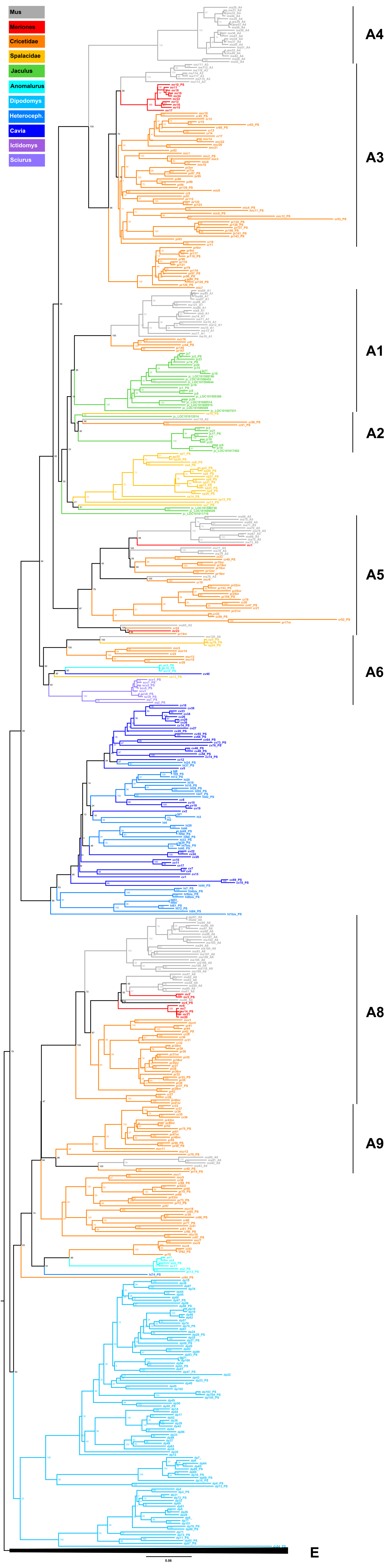

Supplementary Figure S5. Neighbor-joining phylogram of family-A V2Rs in rodents. The tree is based on a multiple alignment of all V2RA DNA sequences (exon-3) that are identified in representative species of Rodentia as shown in Fig. 2 and Table 1. Pseudogenized sequences (PS) are also included in the analysis. The inclusion of sequences into a specific mouse subfamily was based on their monophyletic grouping in the tree. The tree is rooted with family E as an outgroup. Bootstrap support values and V2RA-subfamilies are indicated. For species abbreviations refer to Methods.

Supplementary Figure S6

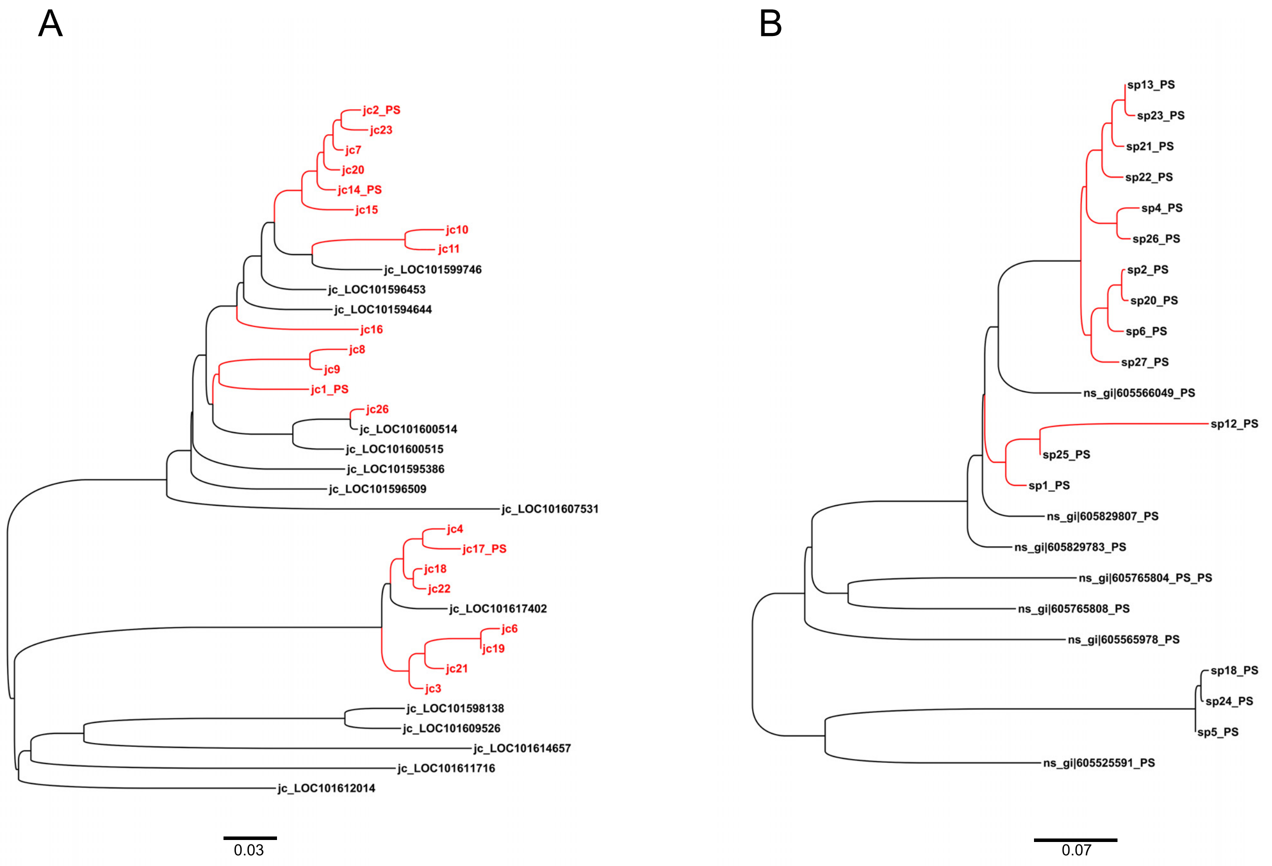

Supplementary Figure S6. V2RA repertoire in Jaculus jaculus and in spalacid species. (A) The neighbor-joining unrooted phylogenetic tree based on multiple alignment of S. leucodon and N. galili of family-A V2R DNA sequences obtained from the nr database (black) and molecular cloning (red). (B) Neighbor-joining phylogenetic unrooted tree of family-A V2R based on multiple alignment of J. jaculus DNA sequences obtained from the nr database (black) and molecular cloning (red). Scale bar: mean number of base substitutions per site.

Supplementary Figure S7

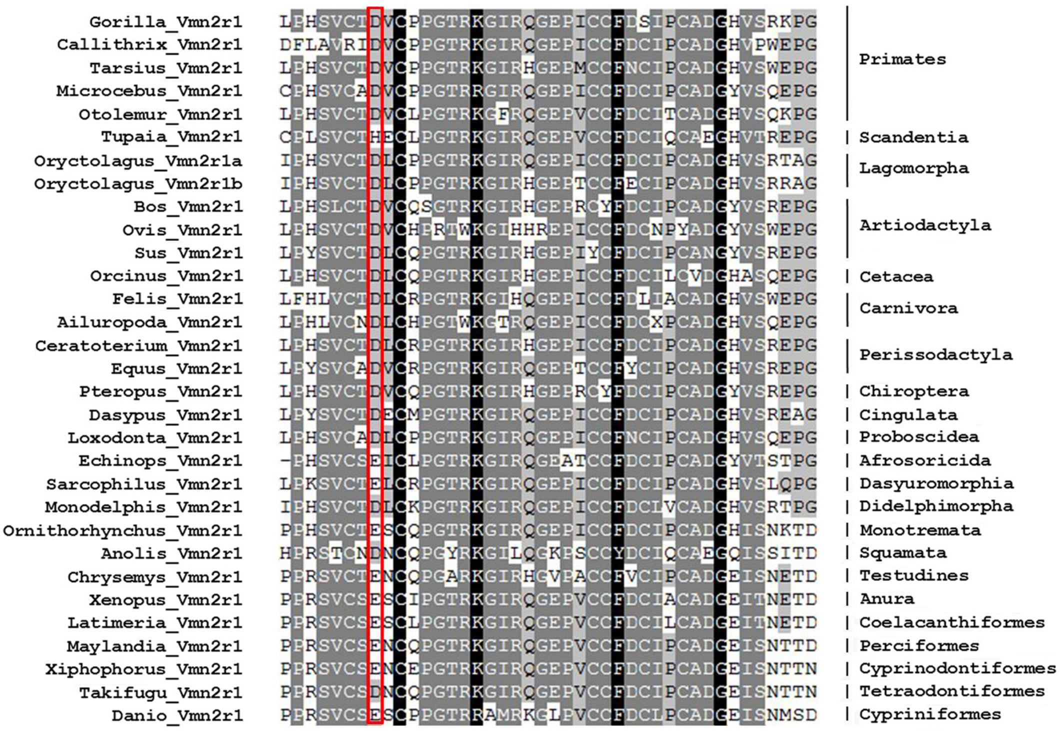

Supplementary Figure S7. Amino acid alignment of the exon-5 of family-C V2Rs in non-rodent species. Sequences are obtained by blast searches. The amino acid position in exon 5 that differentiates subfamily-C1 from subfamily-C2 V2Rs is encased by a red rectangle. The absence of sequences with the Q/H/D → K substitution (see Figure 4) in non-rodent species is verified by PHI-blast analysis (W-N-T-E-S-S-x(8)-[QDH]).

Supplementary Figure S8

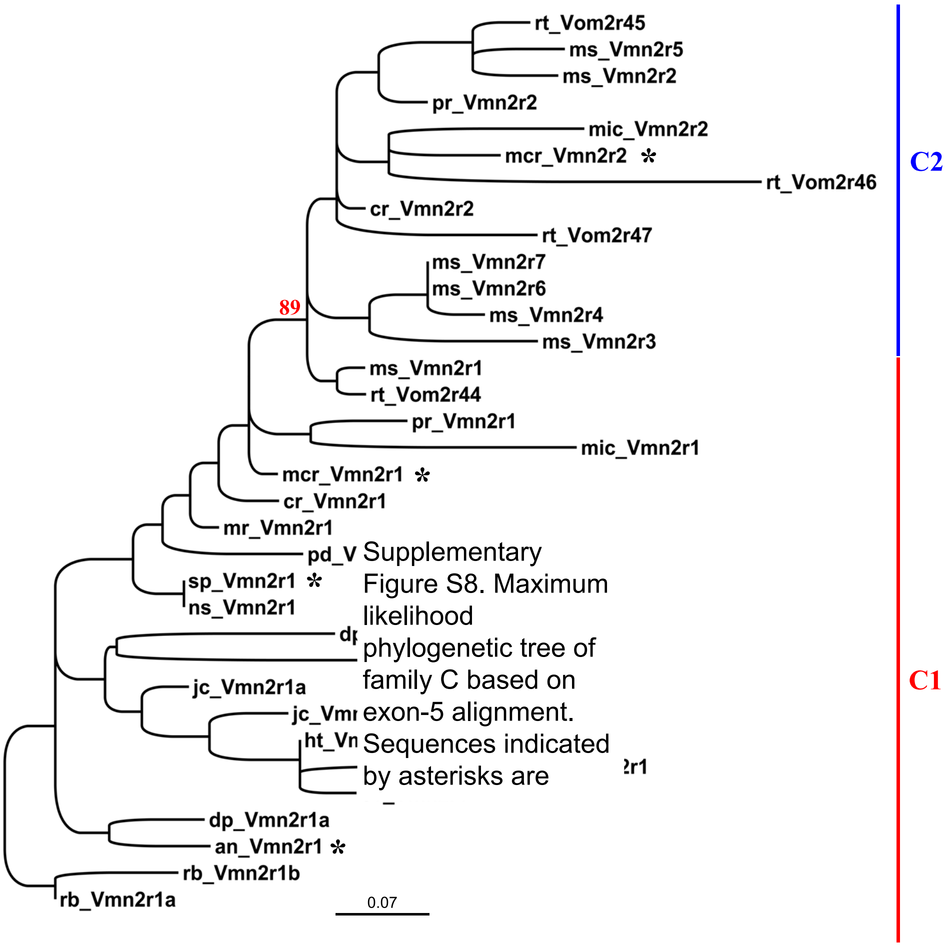

Supplementary Figure S8. Maximum likelihood phylogenetic tree of family C based on exon-5 alignment. Sequences indicated by asterisks are obtained by amplifying and cloning the genomic DNA of rodent species. The Oryctolagus cuniculus (rabbit, rb) sequences are used as outgroups. Bootstrap values (in percentage) are shown at each node. For species abbreviations refer to methods.

Supplementary Figure S9

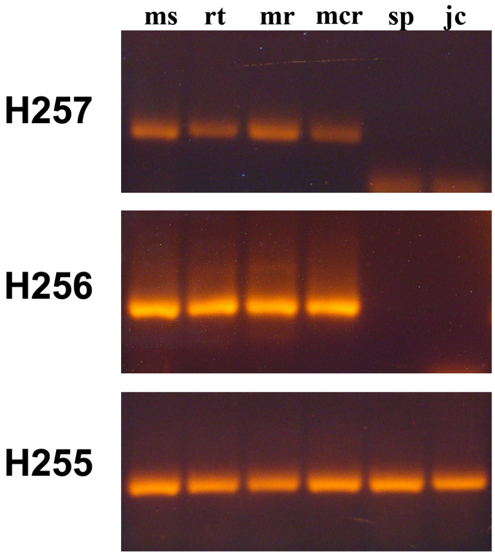

Supplementary Figure S9. PCR amplification of the genomic DNA of mouse (ms), R. norvegicus (rt) , M. uniculatus (mr), M. auratus (mcr), S. leucodon (sp) and J. jaculus (jc) with primers specific for H2-Mv (H256 and H257) and H2-Mv/Mhc sequences (H255).

Supplementary Figure S3

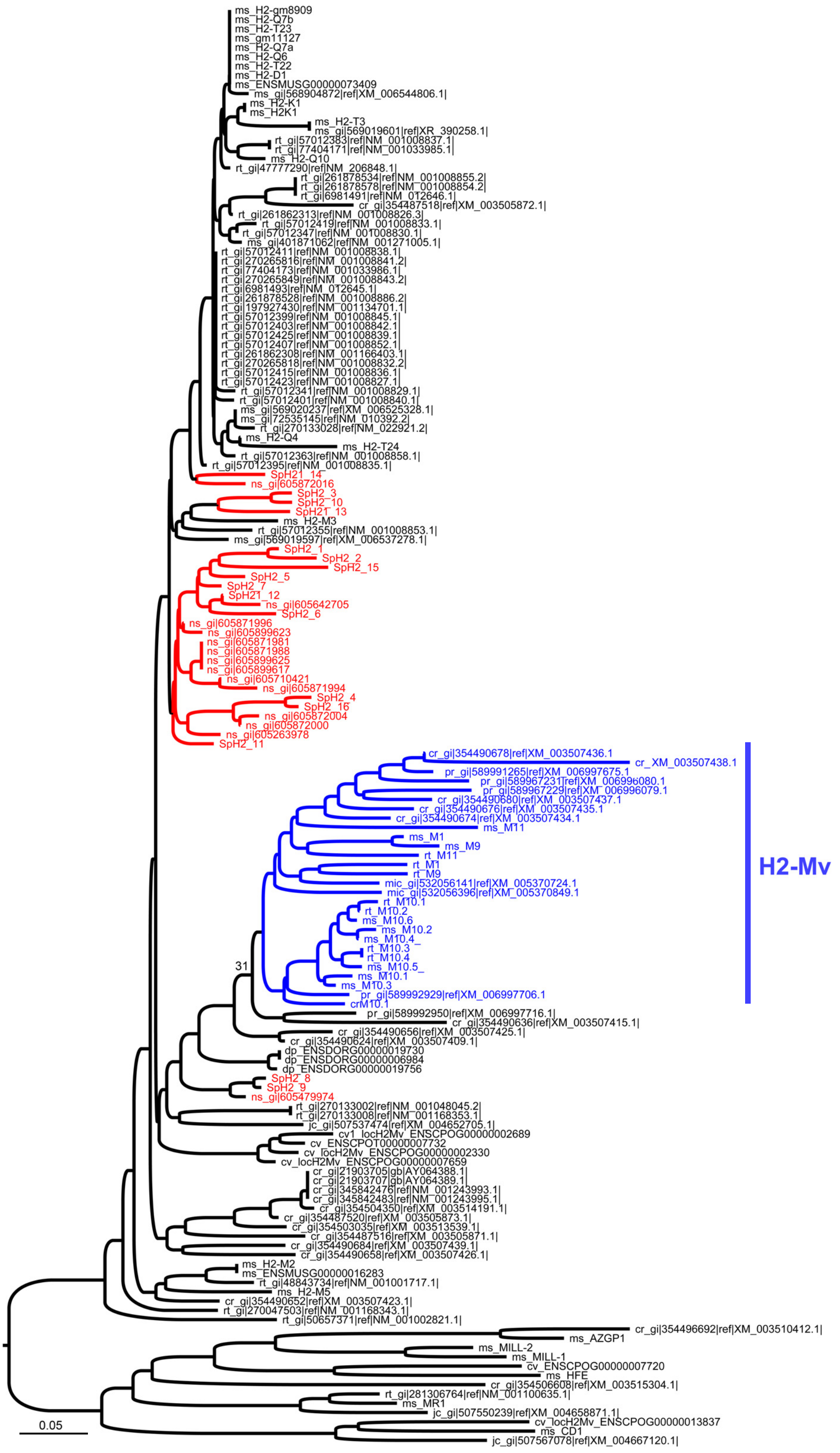

Supplementary Figure S10. Phylogenetic reconstruction of H2-Mv molecules in rodent species. Neighbor-joining phylogenetic tree based on multiple alignments of Mhc DNA sequences (exon 4) retrieved by blastn search from the WGS and nr databases of C. porcellus, D. ordii, J. jaculus, N. galili, M. ochrogaster, P. maniculatus, , C. griseus, R. norvegicus and M. musculus with mouse M1 and M10 genes as queries. The sequences of S. leucodon are obtained by PCR amplification of genomic DNA with H255 primers. The tree is rooted using mouse CD1 as an outgroup. H2-Mv sequences are represented in blue whereas spalacid Mhc sequences in red. For species abbreviations refer to methods. The Bootstrap value is shown for the H2-Mv node.

Supplementary Figure S11

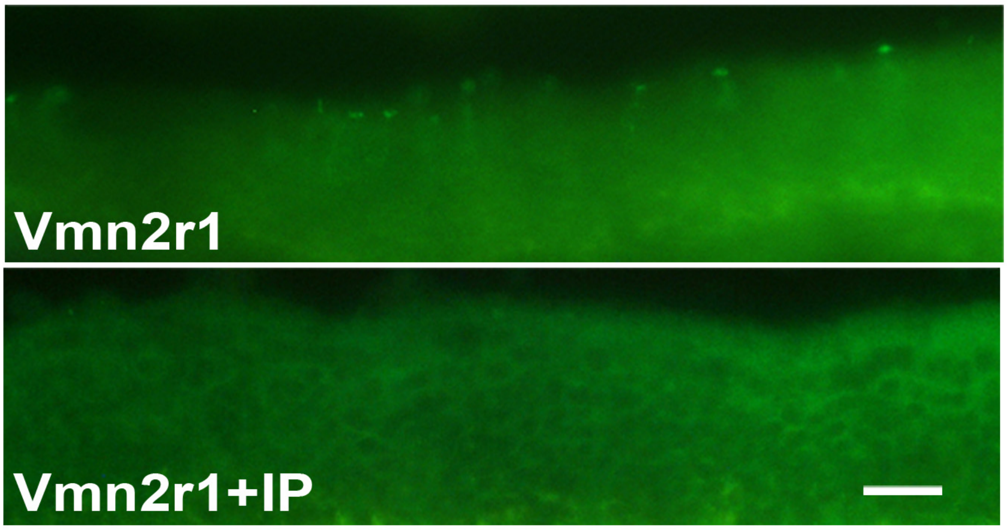

Supplementary Figure S11. Antibody control. Sections of mouse olfactory epithelium are stained with an antibody against Vmn2r1 preincubated with the immunogenic peptide to which it is raised (IP). Scale bar, 20 µm.

Supplementary Figure S12

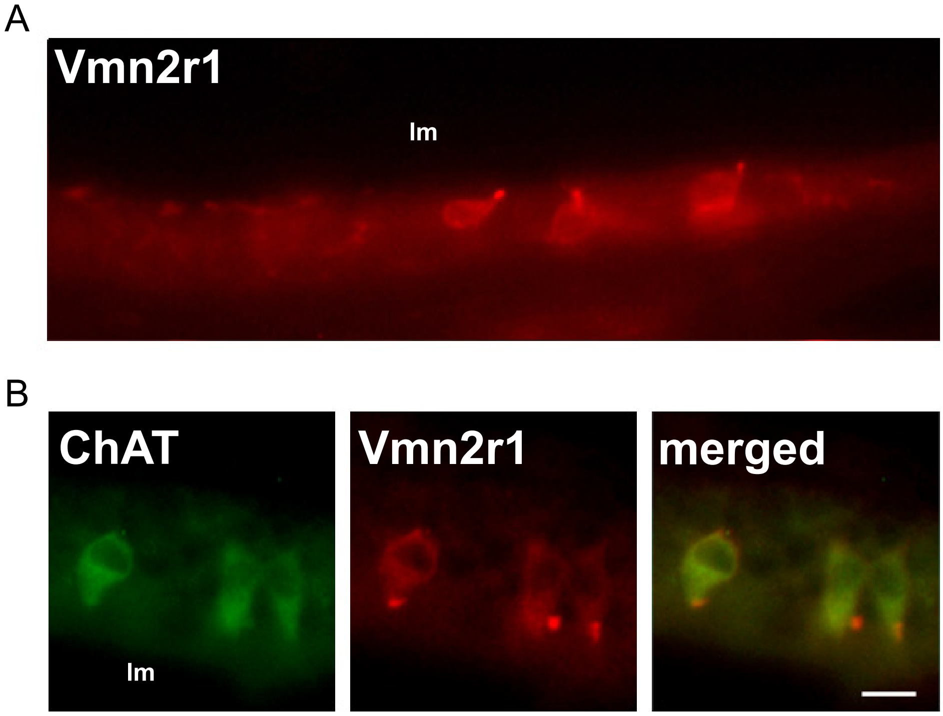

Supplementary Figure S12. Vmn2r1 positive cells in trachea. (A) Sections of trachea stained with anti-Vmn2r1 antibody show immunopositive isolated cells in the respiratory epithelium. These cells are morphological similar to those observed in the olfactory epithelium. Lm, tracheal lumen. Scale bar, 10 μm. (B) Double label immunohistochemistry is performed with an antibody against Vmn2r1 and ChAT in tracheal sections. Scale bar, 20 μm.

Supplementary Figure S13

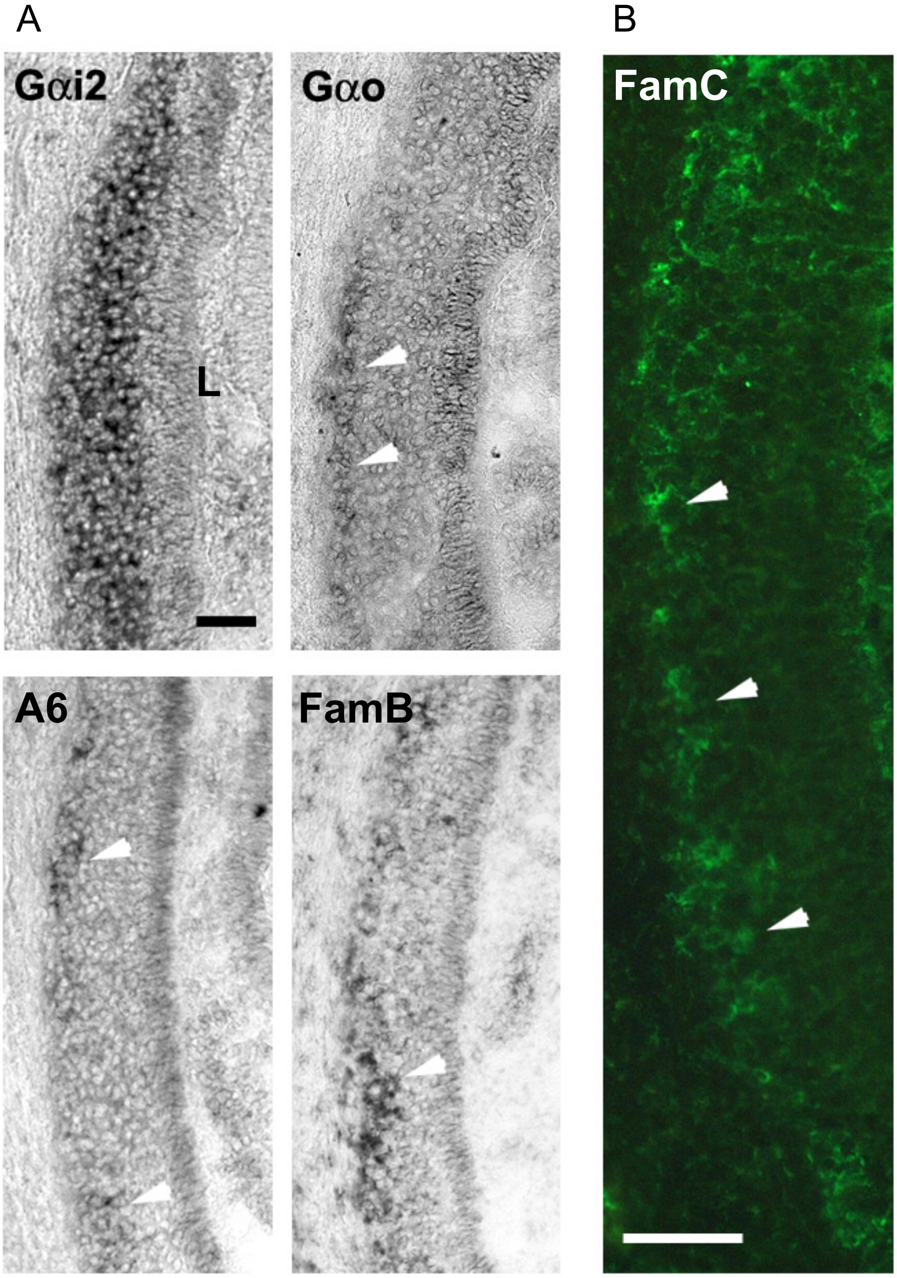

Supplementary Figure S13. V2R expression in Sciurus vulgaris. (A) In-situ hybridization with digoxigenin labeled antisense probes coding for genes expressed in the apical (G-protein α-subunit, Gαi2) and basal (G-protein α-subunit, Gαo; scvV2RA6; scvfamily-B V2R) neurons of the VNO. Scale bar, 50 µm. (B) Immunolocalization of the basal neurons expressing the family-C V2R. Staining is performed using an antibody raised against mouse family-C V2Rs (FamC) (Martini, et al., 2001). Scale bar, 50 µm.

Supplementary Figure S14

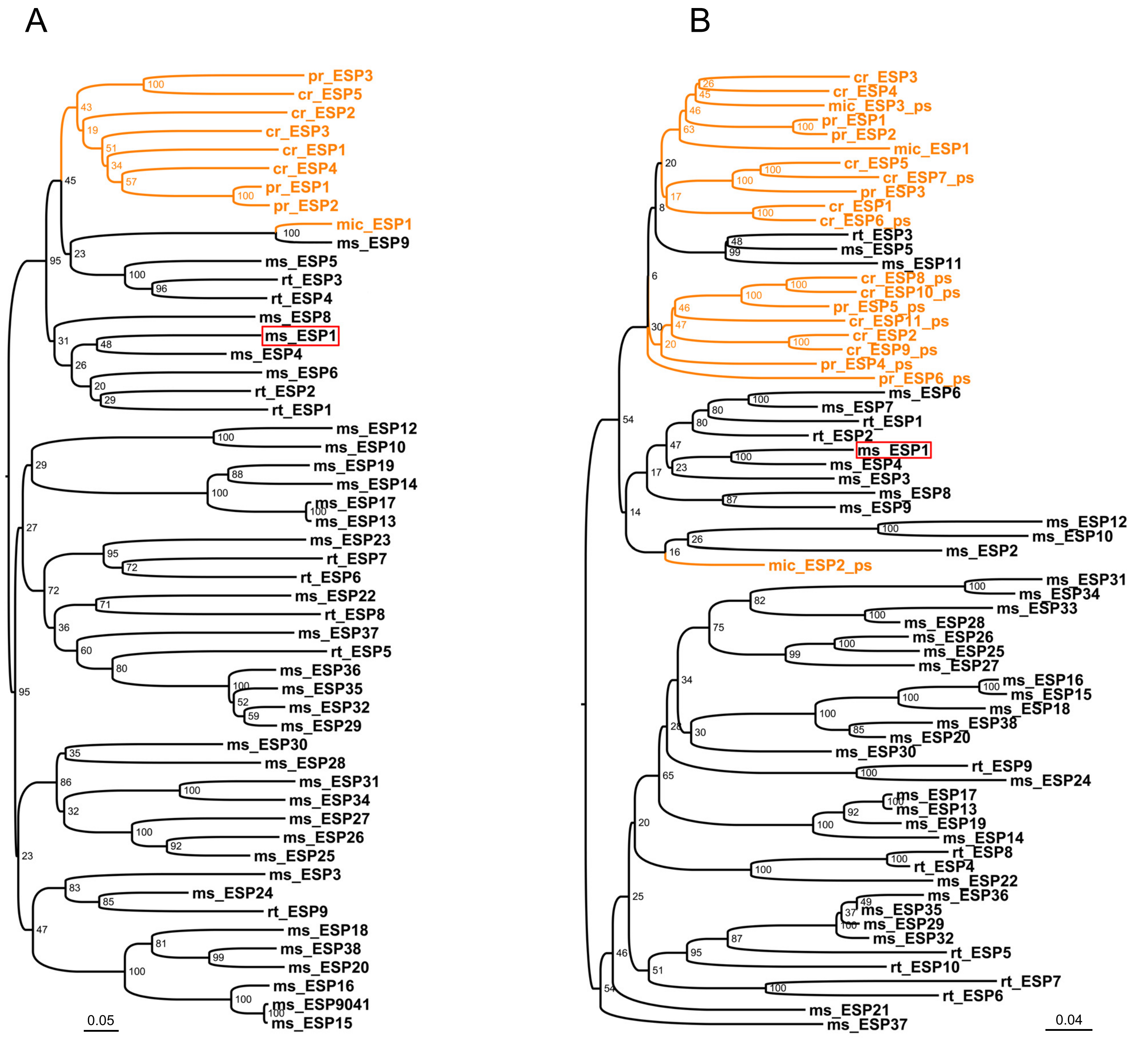

Supplementary Figure S14. Evolutionary history of ESPs in rodentia. (A) Neighbor-joining phylogenetic tree based on multiple amino acid sequence alignment of ESPs, translated and partially reconstructed from DNA sequences obtained by tblastn and blastn searches with ESP DNA-sequences (exon 2) of rat and mouse against the available rodent WGS and nr databases (for more details refer to Suppl. Table S5). Cricetidae branches are colored in orange. Mouse ESP1 is encased in red. For species abbreviations refer to methods. Scale bar: mean number of amino acid substitutions per site. (B) Neighbor-joining phylogenetic tree based on multiple DNA-sequence alignment of ESPs, obtained by blastn search with ESP DNA-sequences (exon 2) of rat and mouse against the available rodent WGS databases. Pseudogenized sequences are included. The Cricetidae branches are colored in orange. Mouse ESP1 is encased in red. Bootstrap values are shown. For species abbreviations refer to methods. Scale bar: mean number of base substitutions per site.
