# Supplementary material for: Evolution of Spatially Coexpressed Families of Type-2 Vomeronasal Receptors in Rodents
Source: Genome Biol Evol. 2014 Dec 23;7(1):272–85. doi: 10.1093/gbe/evu283 (PMC4316634; doi:10.1093/gbe/evu283)
Supplement: Supplementary Data [file supp_evu283_Supplementary_file_S1.pdf]

Accessions: NCBI/Ensembl

Ornithorynchus anatinus (platypus)
Type-2 vomeronasal receptors
ENSOANG00000010029, gi|345310514

Dasypus novemcinctus **(armadillo)**
Type-2 vomeronasal receptors
gi|488527039, gi|488527045, gi|488527043, gi|488527041, gi|488580751, gi|488574987, gi|488583513, gi|488574989, gi|488569487, gi|488585153, gi|488579950, gi|488581765, gi|488588474, ENSDNOG00000019348.

Loxodonta africana **(african elephant)**
Type-2 vomeronasal receptors
gi|344289015

Felis catus **(cat)**
Type-2 vomeronasal receptors
gi|410971138

Suus scrofa **(pig)**
Type-2 vomeronasal receptors
gi|350580569

Ovis aries **(sheep)**
Type-2 vomeronasal receptors
gi|426219376

Equus caballus **(horse)**
Type-2 vomeronasal receptors
gi|149729885

Spermophilus tridecemlineatus **(ground squirrel)**
Type-2 vomeronasal receptors
gi|532093238

Sciurus vulgaris **(Eurasian red squirrel)**
Type-2 vomeronasal receptors
KJ847412.1, KJ847413.1, KJ847414.1, KJ847415.1, KJ847416.1, KJ847417.1, KJ847418.1

Octodon degus **(degu)**
Type-2 vomeronasal receptors
gi|507715372, gi|507715370, gi|507715301, gi|507713644, gi|507713522, gi|507713520, gi|507712744, gi|507712740, gi|507712736, gi|507712648, gi|507712644, gi|507711764, gi|507711760, gi|507711756, gi|507711752, gi|507711748, gi|507711744, gi|507711740, gi|507711736, gi|507711732, gi|507711728, gi|507711724, gi|507711716, gi|507711712, gi|507711708, gi|507711704, gi|507711700, gi|507711696, gi|507711692, gi|507710825, gi|507710821, gi|507710615, gi|507710599, gi|507710473, gi|507703127, gi|507703123, gi|507703119, gi|507703115, gi|507703107, gi|507703103, gi|507703099, gi|507703095, gi|507703088, gi|507703084, gi|507703080, gi|507703076, gi|507703072, gi|507685909, gi|507682767, gi|507652285, gi|507652277, gi|507636974

Chinchilla lanigera
Type-2 vomeronasal receptors
gi|533147610, gi|533193893, gi|533193891, gi|533193889, gi|533193969, gi|533195396, gi|533194021, gi|533193919, gi|533193917, gi|533193959, gi|533193957, gi|533205205, gi|533193967, gi|533205203, gi|533205201,

gi|533205199, gi|533204759, gi|533186303, gi|533194029, gi|533186301, gi|533193961, gi|533194013, gi|533193543, gi|533193955, gi|533204767.

***Heterocephalus glaber* (naked mole rat)**
Type-2 vomeronasal receptors
XM_004834499.1

***Cavia porcellus* (guinea pig)**
Type-2 vomeronasal receptors
ENSCPOG00000026120, ENSCPOG00000027304, ENSCPOG00000024688, ENSCPOG00000027528, ENSCPOG00000023032, ENSCPOG00000026403, ENSCPOG00000020354, ENSCPOG00000027402, ENSCPOG00000020639, ENSCPOG00000026305, ENSCPOG00000020266, ENSCPOG00000024706, ENSCPOG00000023021, ENSCPOG00000020635, ENSCPOG00000019824, ENSCPOG00000023880, ENSCPOG00000023273, ENSCPOG00000024100, ENSCPOG00000025728, ENSCPOG00000026642, ENSCPOG00000022627, ENSCPOG00000020200, ENSCPOG00000025441.

***Anomalurus* sp**
Type-2 vomeronasal receptors
KJ847403.1, KJ847404.1, KJ847405.1, KJ847406.1, KJ847407.1, KJ847408.1, KJ847409.1, KJ847410.1, KJ847411.1

***Spalax leucodon* (lesser mole rat)**
Type-2 vomeronasal receptors
KJ847354.1, KJ847349.1, KJ847339.1, KJ847358.1, KJ847357.1, KJ847356.1, KJ847355.1, KJ847353.1, KJ847352.1, KJ847351.1, KJ847350.1, KJ847348.1, KJ847347.1, KJ847346.1, KJ847345.1, KJ847344.1, KJ847343.1, KJ847342.1, KJ847341.1, KJ847340.1, KJ847338.1

***Jaculus jaculus* (lesser Egyptian jerboa)**
Type-2 vomeronasal receptors
KJ847359.1, KJ847360.1, KJ847361.1, KJ847362.1, KJ847363.1, KJ847364.1, KJ847365.1, KJ847366.1, KJ847367.1, KJ847368.1, KJ847369.1, KJ847370.1, KJ847371.1, KJ847372.1, KJ847373.1, KJ847374.1, KJ847375.1, KJ847376.1, KJ847377.1, KJ847378.1, KJ847379.1, KJ847380.1, KJ847381.1, KJ847382.1, gi|507541613, gi|507567714, gi|507564994, gi|507567712, gi|507570849, gi|507576186, gi|507578857, gi|507578744, gi|507579144, gi|507578989, gi|507570053, gi|507579093, gi|507578756, gi|507570899, gi|507536246, gi|507536248

***Peromyscus maniculatus* (Prairie deer mouse)**
Type-2 vomeronasal receptors
gi|589960468, gi|589992913, gi|589960474, gi|589995422, gi|589960472, gi|589995180, gi|589968385, gi|589995432, gi|589995059, gi|589996311, gi|589969228, gi|589956449, gi|589956644, gi|589956445, gi|589956443, gi|589956455, gi|589968509, gi|589956447, gi|589956453, gi|589968191, gi|589995346, gi|589968193, gi|589994568, gi|589994302, gi|589966519, gi|589963547, gi|589963541, gi|589963499, gi|589963501, gi|589994711, gi|589995846, gi|589965776, gi|589996236, gi|589995871, gi|589994713, gi|589967591, gi|589965780, gi|589967593, gi|589965778, gi|589960384, gi|589960381, gi|589960470, gi|589995320, gi|589969685, gi|589968389, gi|589983819, gi|589951481, gi|589951479, gi|589995057, gi|589968467, gi|589994748, gi|589964108, gi|589985723, gi|589968507, gi|589957055, gi|589995330, gi|589968573, gi|589995475, gi|589968571, gi|589956453, gi|589969578, gi|589969580, gi|589969576, gi|589996626, gi|589968787, gi|589957248, gi|589918945, gi|589918947
H2-mv
gi|589992930, gi|589992951, gi|589991266, gi|589967232, gi|589967230

***Microtus ochrogaster* (Prairie vole)**
Type-2 vomeronasal receptors
gi|532007317, gi|532059123, gi|532058253, gi|532036537, gi|531999162, gi|531999453, gi|531999451

H2-mv

gi|532056142, gi|532056397

***Cricetulus griseus* (Chinese hamster)**

Type-2 vomeronasal receptors

gi|354507417, gi|354507282, gi|354506798, gi|354504430, gi|354505818, gi|354508049, gi|354499749, gi|354504998, gi|354499743, gi|354506551, gi|354500264, gi|354504996, gi|354500266, gi|354494033, gi|354487592, gi|354495110, gi|354495114, gi|354494039, gi|354504500, gi|354500978, gi|354487594, gi|354507044, gi|354488240, gi|354475502, gi|354488244, gi|354506796, gi|354495108, gi|354487596, gi|354487590, gi|354499512, gi|354502766, gi|354500097, gi|354507107, gi|354500099.

H2mv

gi|354490637, gi|354490675,gi|354490681, gi|354490677, gi|354490679, gi|354490683, gi|354490639

***Meriones unguiculatus* (Mongolian gerbil)**

Type-2 vomeronasal receptors

KJ847383.1, KJ847384.1, KJ847385.1, KJ847386.1, KJ847387.1, KJ847388.1, KJ847389.1, KJ847390.1, KJ847391.1, KJ847392.1, KJ847393.1, KJ847394.1, KJ847395.1, KJ847396.1, KJ847397.1, KJ847398.1, KJ847399.1, KJ847400.1, KJ847401.1, KJ847402.1

***Microcebus murinus* (mouse lemur)**

Type-2 vomeronasal receptors

gi|434858693, gi|434858658

***Callithrix jacchus* (marmoset)**

Type-2 vomeronasal receptors

gi|390476195
