# Supplementary material for: Evolution of Spatially Coexpressed Families of Type-2 Vomeronasal Receptors in Rodents
Source: Genome Biol Evol. 2014 Dec 23;7(1):272–85. doi: 10.1093/gbe/evu283 (PMC4316634; doi:10.1093/gbe/evu283)
Supplement: Supplementary Data [file supp_evu283_Supplementary_file_S2.pdf]

Family-E and family- D V2Rs: full length sequences

>Vmn2r18 **[Mus musculus],** mouse vomeronasal type 2 receptor (V2R), Family E, complete sequence
ATGTACTTTACTTTTAATTGTCTTTTCCTGCTCCTGCAATCTTTATTTACACCAGTGGAGATGACTCGCAAGAGCTGTTA
TGAGTTCTGGAATCCCAGCACCTATATGAAGGCAGACATAATCCTAGGTGCATTCATCCCCATTTTCATCTTACTAGAAG
GTCCCAAAGAAATCCAGCAATTTATTTGGATCCAGCCAGATATAGTACTGGAAAACATCCTACTACAATGGAAAAACTAT
CAATATTTGATGACCTTATACTTTGCCATTGATGAAATCAATAAGGACACCCACCTGCTTCCCAATGTTACCTTGGGTTT
CCACATCTATAATGCCTTTAATTTTCACCCAAGAACTTTGGAGGGACCTCTAATGTGGCTGTCTGGAAGGAATGAGTTTA
TCCCTAACTATAAATGCAAAACTCAATACAAAGCTCTTGGAATAATTTCAGGAACTAGGAGTGAATATTCTGCTGGTATT
GGATCTCTTTTGGAGCGCTACAAAATTCCACAAGTAAGTTTTGCTTTGCTTCTTCAGGTCAACTATGGACCTTTTGATTC
CATGCTCAGTGACAAAGATACATTCCCATCCCTTTATCAGATGTCTCCTAAGGACACAGCTCTAACACAAGGGGTGATCT
CTGTATTGCTGCACTTTGGCTGGAAGTGGGTGGGGGTCATTGTGTCTGATGATCTGAAGGGAATGGAGTTCCTCTCTCAC
CTGAAAGCAGAGATGGTATCAGAAGACATCTGTGTGGCTTTTACGAAAAGCCTCAAAACAAATTGGAAGGTACAGTATAC
ATCTGGTGTTGGATTGGCAAATTTGAACCACCATCCACAAGTAAATGTGAACATACTCTATGGTGATATAGATGACTTGC
TGTATTTTTGTTTGGAAAATAAACTTCTGTCAAGCAGAAGGAAGGTGTGGATCATGGCAAAGCTCCATTTAGTATACTTA
GAGTCTGTATTCTTTAAGAGGAAGGATTTGATAAACTTTTTCACAGGAAGCTTACTATTTTCCAAGAAGAGAAATATCCC
TGGTTTCAAGAACTTTCTCGAATCACTCACACCCTCCTACTACCCAGGAGAATTTTACTTCTATAAATTCTGGATTGACA
AGTTTGATTGCGCACCTCCTGCTTTGCTATGTGGAAGGAATAAACCCTGTCCACTAAATATTACTCTGAAGAATAAAGAA
GGAGAAAATGATATAATGATCCCTTCTGAGGCCAGCTATTCCATATGGAATACAGTGTATGCAGTGGCCCATGCCATTCA
TAATATGTTTTTGAGAAAAACAGAAATAGGATCTCATGAAGACAAAAATCATGACAGGTTTCTTCCTTGGCAGCTGCATC
CATTTCTGAGGAAAATCCAATTTACCAACACTGCTGGGGACCCAATAAGTTTTGATGAGCATAAGAATCATATGGAAATA
TATGAAATACAAAATTTCATGAAATATACTAAGCAGCATTCATATCTAGTTAAAGTCGGAGAGTTTGTCTCCAAGAGTCC
ACAAGATCAAAGCTTATTCATCAATGAATCTCTGATTAAATGGCCAAACAATTTTAATCAGACTCCTCAGTCTGTATGTA
CCCAGAGCTGTGGTCCAGGGTTCTGGAAAATTCTACAAGAAGAAAGGCCTGTCTGCTGTTTTTCTTGTGTGCCTTGTCCA
GAGAAGCACATTTCCAACCTAACAGATCAGCAGAAGTGTATTGCTTGCCTGATTCAAGAATACCCAAACCCTGAGAGAAA
CCACTGTCTGCCTAAATCAGCGACCTTCCTGTCCTTTGAGGATCCTCTGGGCATGTCTCTGGCCTGCATGGCTCTGGGCT
TCTCTGTTAGCACAGCTGCAGTTTTGGGGATCTTTCTCAAGTATCAAGACTCACCCATCATTAAGGCCAATAACCTTACT
CTCAGTTACATCCTGCTGATCTCCCTCCTCCTCTGCTTCCTATGCTCCTTTCTCTTCATTGGCCACCCAAACACAGTCTC
CTGCATACTGCAACAAATAACATTTGCACTTGTGTTCACATTGGCTCTTTCCACTGTTTTGGCCAAAACCATAACTGTAA
TTCTGGCTTTTAGGGCCTTGAAACCAGGAAGAACAATGAGAAGGCTGTTTGTTTTAGGCATCTACAATGCTGTTATCCCC
TTCTGTGTCCTGATCCAACTTATTACCTCTGGCATCTGGCTGGCAACCTCACCACCCTATATTGACACAGATTCATACTC
TGAACATGCTCATATCATCATTTTATGCAACAATGGCTCAGTTACTGCATTCTATTGCATGCTGGTATACTTAGGGACAC
TGGCACTAGGGAGCTTCACTGTGGCTTTCCTGGCAAGGAACCTTCCTGACACATTCAATGAAGCCAAGTTCCTGACATTC
AGTATGGTGGTGTTCTGCAGTGTCTGGGTCACCTTTGTTCCCGTTTACCAGAGCACCAAGGCCAAGGCCATGGTGGCTGT
GGAGGTCTTCTCCATCTTGGCCTCCAGTGCAGGGTTGCTAGCATGTATTTTTTTCCCTAAGTGCTACATTATTCTAAGAG
AACCTGATAAGAGAAACTTGAAATGTTTTAAAAACAAAACACGTATAAAAAAATGA
>Vmn2r53 **[Mus musculus],** mouse vomeronasal type 2 receptor (V2R), Family D, complete sequence
ATGTGGCTGTGTACTCTCCTTTCTGTTCTCTTTGGATGTCTCTCTGGGTCTGCCCTTTCTGGACCAAGAGTCTGGATAAT
TCCAAGGCAGAGTCCTCGCTTTGACCGTCCTGGAGATGTAATTGTGGGTGGCAGCTTCTCTATCTTTCACTTTTCTGATG
GTACCCTGTCTAATTTCACTGCCCCACCACCTGTGCTGAAGACTTCAAGTGCCTCCATGTGGAGCTACCGGGTAGCCCAG
AGTTTCGTCTTTGCCATTGAGGAGATTAATAGGAGTGCTCACTTGTTGCCCAATTTGACACTGGGCTTCTCCATTCGAAA
CTCTGGAGACTCAGTGCATGGAGCGCTCTATGAGACAATGGGCTTTCTCACAGGGCAGGAGGAGCCCATCCCCAACTACA
CATGCCAGCATGGCTCTCCTCAGGCTGCCTTGGTTGGGGACACACGGTCTTCCCTGTCTGTCTCCATGGCCAGACTTCTG
GGGCTGTACAAGTTTCCCCAGGTCAGTTACTCATCTTCACTACCCAGTCTGAGTGACAAGATCCAGTTTCCATCCTTCAT
TCGGACCCTGACTAGTGACCTAACATGCTCCCATGCAGTTACCCAGCTGGTAATTCACTTTCAATGGTCCTGGGTGATCA
TTCTTGCCCATGATGATGACTTTGGGCAGCAGGCCAGCTCTCTGGCCACACAGATGTTGACCCCAGCTGGTGTGTGCATT
GAGTATCACCTTCACGTCCCTTCCCATCAGTCCTTTGGGAAGATTGAAGAGACTGTCCAGAAGATGCAGAAATGTACAGC
CAGAGTTGTTTTGGTTTTCTTAAGCAATTCAAATTTCCAGCTCATCCTACATGGCTTACTGGCTGTCCCTGTCTCAGGAC
AGGTGTGGGTCAGCATGGACACTCTGCACATGGCACTTGCCCTGACCATACCAGGCATTTCCCAGGTGTTGCAAGGCACA
TTTGGCCTTCTGTATCACAGCAGCAGGGCTATTGGCTTCCCTGAGTTCCTTGCTCACCTGCGCCCCAGCCAGACCCCAGA
AGACATGTTTATAAAGAAGTTCTGGGAGTTCACCTTTGATTGTACATGGCCCTACCAAAACAGCACAGTGACAGAGGGTG
TCCAGTTCTGCACAGGAAATGAGAGTCTGAAAAACAAGCCACATCCCTTTCCAGAAGTGAGTAGAATTGATGCTGCTTAC
ACAGCTGTCTACAGCATTGCCCATGCTCTGCACAATATGCTAGCCTGTGAGCACCAGGAGAGGAAAGGTACAAACTCTCA
CAACTTCCATTCCTGGCAGGTAAGAGAGACATGTCTGCTTCATAACCTCAGAAATGTGTACTTCAAGACTTCTGATGGAA
ATAAGATTATGTTTGATGCCAATGGAGATTTGGTGACACAATTTGACATTTTCCAAGGGAAGAAGACTCCTGCAGGTGTA
TTCCACTTGGTGCATGTAGGCTTGATAGACCCTCAAGCCTCTTCAGGAAACAAAATGATGGTCCAGTTGAAGCAGGATCT
CCAAGTGAGTTCTCTAAATGCAGAGATAACTGTTGTGCCCACCTCCATCTGCAGTGAAAGCTGCCTTCCAGGGTTCAGCC
AGGTACCCCGACTGGGAGCCCCCCACTGCTGTTTTGATTGCAGTCCATGCCCTGAGGGACAAAATGGTGTCGTTGTGTCT
CCCATAGACATGAAGAGATGCCTTCTGTGCCCCAAGGAGCAGTACTCGAGCCACACCAGAGACCATTGCCTGCCCAGGAC
AGAGATCTTCTTGGCCTTTGAGGAACCACTGGGATTCATATTGGCTTTGGTAGCACTCTTGCTGGCTGGTCTGGCTGTCC

TGGTTCTTGGAGTGTTCCTGAAGCACCGGGATACACCTGTGGTCAGGGCCAACAACAGAACTCTCAGCTACTTCCTCCTG
ATCTCTCTTTCTTTGTGTGCCCTGTGTGCCTTGCTCTTCCTTGGGAGACCCACTGTTACTACCTGCCTCCTCCGTCAGAC
CATCTTTGCTGTGGTATTCACAGTGGCTGTCTCCTCCGTTCTGGCTAAGACTCTAACAGTGGTCTTGGCCTTCCGAGTCA
CCAGGCCACGAAGCAGGATCCAGGTATGCCTGAGCTCTGGTGCTTCTACCTCAGTGGTCCTCATTGCTTCCTTAATACAG
GTTGTTCTTTGTGGGATATGGTTGATTACCTCTCCACCATTCCCAGACAGGGATATGATCTCAGAGCCCCGGCACACTGT
CATCCAATGCCATGAGGGTTCTGGAGCCAACTTCTTCTGTGTGCTGGGTTTCTTGGGTTTCCTGGCAGGGGGTACCTTCT
CTGTGGCTTTTCTAGCCAGGGGCCTTCCAGATGTCTTCAATGAGACCAAGTTCCTCACCTTCAGCATGCTGCTCTTCTGC
AGTGTCTGGACAGCCTTCCTTCCCCTGTACCATAGTGCCCGGGGAAAGTCCACTGTGGCTGTAGAGATCTTTTCCATCCT
GGCCTCCACTGCTGGGCTTCTGGGTGGCATCTTCATCCCCAAGTGCTACATCATCTTGCTGAAACCTGAGAAGAACACCC
CTGCCTGGTTAAGGCAAGGCCACCAGGTACAGCACAATAGGCAGTGTGAGATG
>Vmn2r55 **[Mus musculus],** mouse vomeronasal type 2 receptor (V2R), Family D, complete sequence
ATGTGGCTGTGTGCTTTCATTTTTGCTTTCTTTGTCTGTCTCTCTGGGTCTGACCTTTCTGGACCAAGAGCCTGGATATT
GCCAAGACAGAGCCCTCATTTTGACCGTCCTGGAGATGTAATTGTTGGGGGTAGCTTCTCCATCTTTCACTTCTCTGATG
GTACACCCTCTGATTTCACTGCCCCACCAACTGGGCTGCAGACTTCAAGTGTCTCCATGTGGGGCTACCGGGTGGCCCAG
AGTTTCGTCTTTGCCATTGAGGAGATTAATAGGAGTGCTCACTTGTTGCCCAATTTGACACTGGGCTTCTCCATTCGAAA
CTCTGGAGACTCAGTGCATGGAGCCCTCTATGAGACAATGGGCTTTCTCACAGGGCAGGAGGAGCCCATCCCCAACTACA
CATGCCAGCATGGCTCTCCTCAGGCTGCCTTGGTTGGGGACACACGGTCTTCCCTGTCTGTCTCCATGGCCAGACTTCTG
GGGCTGTACAAGTTTTCCCAGGTCAGTTACTCATCTTCACTACCCAGTCTGAGTGACAAGATCCAGTTTCCATCTTTTCT
TCGGACCCTGACTAGTGACATTACATCTTCCCATGCAGTTACCCAGCTGATAATTCACTTTCAATGGTCCTGGGTGATCA
TTCTTGCCCAAGATGATGACTTTGGGCAGCAGGCCAGCTCTCTGGCCACTCAGCAGCTGAGTCCAGCTGGGGTTTGCATA
GAGTATCACCTCCACGTCCCTTCCCATCAGTCCTTGGGGAAGATTGAAGAGACTGTCCAGAAGATGCAGAAATGTACATC
CAAAGTTGTTCTGGTTTTCTTAAGCAATTCTAATTTCCAGCTCATCCTACATGGCTTACTGGGTGTCCCTGTCTCAGGAC
AGGTGTGGGTCAGCAAAGGCACTCTGCACATGGCACTTGCCCTGACCATACCAGGCATTTCCCAGGTGTTGCAAGGCACA
TTTGGCCTTCTGTATCACAGCAGCAGGGCTATCGGCTTCCCTGAGTTCCTTGCTCACCTGCGCCCCAGCCAGACCCCAGA
AGACATGTTTATAAAGAAGTTCTGGGAGTTCACCTTTGATTGTACGTGGCCCTACCAGAACAGCACAGTGACAGAGGGTG
TCCAGTTCTGCACAGGGAATGAGAGTCTGAAAAACAAGCCACATCCATTTCCAGAAGTGAGTAAAATTGATGCTGCTTAC
ACAGCTGTCTACAGCATTGCCCATGCTCTGCACAATATGCTAGCCTGTGAGCACCAGGAGAGGAAAGGTACAAATTCTCA
CAACTTCCATTCCTGGCAGTCAACAATATTTCCCTTTCTTTCTCAGCTGCTCCATGCCCTCAAAAAGGTACACTTCAAGA
CTCTGGATGGAATCAAGATTATGTTTGATGCCAATGGAGATTTGGTGACAAAATTTGATATTTTCCAAGGGCAGAAGACT
CCTGCAGGTGTATTCCACTTGGTGCATGTAGGCATGATAGACCCTCAAGTCTCTTCTGGGAACAAAATGATGGTTCAGTT
GAAGGAGGATCTCCAAGTGCCCTCCTCCATCTGCAGTGAAAGCTGCCTTCCAGGGTTCAGCCAGGTACCCCGACTGGGAG
CCCCCCACTGCTGTTTTGATTGCAGTCCATGCCCTGAGGGACAAAATGGTGTCTATGTGTCTCCCACAGACATGAAGAGA
TGTCTTCTGTGCCCCAAGGAGCAGTACTCGAGCCACACCAGAGACCATTGCCTGCCCAGGACAGAGATCTTCCTGGCCTT
TGAGGAACCACTGGGATTCATATTGGCTTTGGTAGCACTCTTGCTGGCTGGTCTGGCTGTCCTGGTTCTTGGAGTGTTCC
TGAAGCACCGGGATACACCTGTGGTTAGGGCCAACAACAGAACTCTGAGCTACTTCCTCCTGATCTCTCTTTCCCTGTGT
GCCCTGTGTGCCTTGCTCTTCCTTGGGAGACCCACTGTCACCACCTGCCTCCTCCGTCAGACCACCTTTGCTGTGGTGTT
CACAGTGGCTGTCTCCTCTGTTCTGGCCAAGACTCTCACAGTAGTCTTGGCATTCAAAGTCACCAGACCACGAAGCAGGA
TCCAGATATGCCTGAGCCCTGGTACTTCAACCTTAGTGGTCCTCATTGCTTCCTTAATACAGGTTGTTCTCTGTGGGGTG
TGGTTGGCCACCTTCCCACCATTCCCAGACAAGGATATGCTCTCAGAGCCCCAGCACATTGTCATCCAATGCCATGATGG
TTCTGGTGCCACCTTCTTCTGTGTGCTGGGCTACTTGGGTTTCCTGGCAGGGGGTACCTTCTCTGTGGCTTTTCTGGCTA
GAGACTTACCAGATGTCTTCAATGAGACCAAGTTCCTCACCTTCAGCATGTTGCTCTTCTGTAGTGTCTGGACAGCCTTC
CTTCCCCTGTACTACAGTGCCCGGGGAAAGTCCACCGTGGCTGTAGAGATCTTCTCCATCCTGGCCTCTACCGCTGGACT
TCTTGGTGGCATCTTTATTCCGAAGTGCTACATCATCTTACTGAAACCTGAGAAGAACACTCCTTCCTGGCTAAAGCAAG
GCCATCATATA
>Vmn2r56 **[Mus musculus],** mouse vomeronasal type 2 receptor (V2R), Family D, complete sequence
ATGTGGCTGTGTACTCTCCTTCCTGTTCTCTTTGGCTGTCCCTCTGGCTCTGCCCTTTCTGGAGCAAGAGCCTGGCTGTT
GCCAAGGCAGAGCCCTCATTTTGATCGTCCTGGAGATGTAATCGTGGGGGGCAGCTTCTCCATCTTTCGCTTCTCTAATG
GTACCCTATCTAATTTCACTGCCCCGCCAGCTGGGCTGCAGACTTCAAGCGTTTCCATGTGGGGCTACCGGGTGGCCCAG
AGTTTCGTCTTTGCCATTGAGGAGATTAATAGGAGTACTCACCTGTTGCCTAATTTGACACTGGGCTTCTCTATTCGAAA
CTCTGGGGACTCAGCGCATGGAGCCCTCTATGAAACAATGGGCTTTCTCACAGGGCAGGAGGAGCCCATCCCCAACTACA
CATGCCAGCATGGCTCTCCTCAGGCTGCTTTGGTTGGGGACACACGGTCATCCCTGTCTGTCTCCATGGCCAGACTTCTG
GGGCTGTACAAGTTTCCCCAGGTCAGTTACTCATCTTCACTACCCAGCCTGAGTGACAAGATCCAGTTTCCATCCTTCAT
TCGGACCCTGACTAGTGACCTCACATCCTCCCATGCAGTTACCCAGCTGATAATTCACTTCCAGTGGTCCTGGGTGATCA
TTCTTGCCCATGATGATGACTTTGGGCAGCAGGCCAGCTCTCTGGCCAGTCAGGTGCTAACCCCAGCTGGGGTTTGCATT
GAATTCTACCTCTATGTCCCTTCCCATCAGTCCTTGGGGAAGATTGAAGAGACTGTCCGGAAGATGCAGAAATGTACAGC
CAGAGTTGTTCTGGTTTTTCTAAGCAATTATAATTTCCAGCTCATCTTGTATGGCTTGCTAGCTGTCCCTGTCTCAGGAC
AGGTGTGGGTCAGCAAAGACACTCTGCACATGGCACTTGCCCTGACCATACCAGGCATTTCCCAGGTGTTGCAAGGCACA
TTTGGCCTTCTGTATCACAGCAGCAGGGCTATTGGCTTCCCTGAGTTCCTTGCTCACCTGCGCCCCAGCCAGACCCCAGA
AGACATGTTTATAAAGAAGTTCTGGGAGTTCACCTTTGATTGTATGTGGCCCTACCAGAACATCACAGTGACAGAGGGTG
TCCAGTTCTGCACAGGGAATGAGAGTCTGAAAAACAAGCCACATCCATTTCCAGAAGTGAGTAAAATTGATGCTGCTTAC

ACAGCTGTCTACAGCATTGCTCATGCCCTACATGATATGATATCCTATGAGCACCAGGATGGGAAAGGTACAAACTCCCA
GGACTTCCAGCCCTGGCAGTCTTCAGTATTTCCTTTTTTCCCCCCTCAGCTGCTTCATGTCCTCAGAAAGGTGCACTTCA
AGACTCCCGATGGAAGTGAGATTATGTTTGATGCCAATGGAGATTTGGTGACAAAATTTGACATTTTTCAAGGGCAGAAG
ACCCCCGATGGTGTATTTCACTTGGTCTGTGTTGGCTTGATAGACCCTCAAGCCTCTTCAGGGAACAAAATGATGGTCCA
GTTGAAGGAGGATCTCCAAGTGCCCACCTCTGTCTGCAGTGAAAGCTGCCTTCCAGGGTTCAGCCAAGTGCCCAGACTGG
GAGCCCCCCACTGCTGTTTTGATTGCAGCCCATGCCCTGAGGGACAAAATGGTGTCTATGTGTCTCCCACAGACATGAAG
AGATGTCTTCTGTGCCCCAAGGAGCAGTACTCGAGCCACACCAGAGACCATTGCCTGCCCAGGACAGAGATCTTCCTGGC
CTTTGAGGAACCACTGGGATTCATATTGGCTTTGGTAGCACTCTTGCTGGCTGGTCTGGCTGTCCTGGTTCTTGGAGTGT
TCCTGAAGCACCGGGATACACCTGTGGTTAGGGCCAACAACAGAACTCTGAGCTACTTCCTCCTGATCTCTCTTTCCCTG
TGTGCCCTGTGTGCCTTGCTCTTCCTTGGGAGACCCACTGTCACCACCTGCCTCCTCCGTCAGACCACCTTTGCTGTGGT
GTTCACAGTGGCTGTCTCCTCTGTTCTGGCCAAGACTCTCACAGTAGTCTTGGCATTCAAAGTCACCAGACCACGAAGCA
GGATCCAGATATGCCTGAGCCCTGGTACTTCAACCTTAGTGGTCCTCATTGCTTCCTTAATACAGGTTGTTCTCTGTGGG
GTGTGGTTGGCCACCTTCCCACCATTCCCAGACAAGGATATGCTCTCAGAGCCCCAGCACATTGTCATCCAATGCCATGA
TGGTTCTGGTGCCACCTTCTTCTGTGTGCTGGGCTACTTGGGTTTCCTGGCAGGGGGTACCTTCTCTGTGGCTTTTCTGG
CTAGAGACTTACCAGATGTCTTCAATGAGACCAAGTTCCTCACCTTCAGCATGTTGCTCTTCTGCAGTGTCTGGACAGCC
TTCCTTCCCCTGTACTACAGTGCCCGGGGAAAGTCCACCGTGGCTGTAGAGATCTTCTCCATCCTGGCCTCTACCGCTGG
ACTTCTGGGTGGCATCTTTATTCCCAAGTGCTGCATCATCTTACTGAAACCTGAGAAGAACACTTCTTCCTGGCTCAGGC
AAGGCCATCATGTA
>rb14 **[Oryctolagus cuniculus]**, rabbit vomeronasal type 2 receptor (V2R), Family E, complete sequence
ATGTTGTCTGTGAGCCTTCTGTGCCTACTCCTGCATACTATACACACTTTACAGGGCATGACTCACAGGGAATGCCATCA
TTACCTTAAGCCAAGTATCAACCAAGACGGAGACCTGATCTTGGGTGGCTTTTTCCCTCTCTACTATCCAGAAACAGAAA
CTGAAAAGGTCCAGATATCTTTTTTACATCGTCCCCAGTATAAAACTGTTGTTTCGTGGTGGCTGTGGAAGAACTACCAA
TACGTGCTGGCCTTCCGCTTTGCCATCCAGGAGATCAATAAGGACCCCCAGCTACTCCCCAACCTGACCCTGGGTTTCAA
ATTCTTCAATGCCTTTGCCAGTGAACAGTATACCTTATTGAGCTTAGTGTATTGGTTGGCTGGGATGAATCTGGCTTTTC
CTAACTACAGCTGCCATAAACAAGGAAGACATGTTGCCATCATTGCTGGAACCACATCAGCATTGTCCATTGAATTTGGA
ACACTTTTGGAGCTCTACAAAACCCCACAGCTCACCTATGGTCCTTTTGATCCCATGCTAAATGATAAGGACCAGTTTCC
CTCACTCTATCAGATGGCCACCAGTGACAGCTCTTTGGGCCATGGAATGATCTCCTTGTTGCTGCATTTTGGCTGGATGT
GGGTGGCTCTCTTTGTGTCTGATGACATGAAGGGAGAGCAGTTCCTTCAGTACTTTGAAGCACAGATGCTCAAGAAAAGT
GTCTGTGTGGCCTTTAGAGTGAAGCTCCCTGTCACAAAGAAGTTGTATGGGGATAGTGATCTCACTTTCATGAGTAGCAT
CAGAGCTTCATCTGCAAATGTGCATATACTCTATGGTGATGTAAGAGGTCTCATCATTGTGGATATTTCAGTGAGGTCCT
TTTTAACCATGGGGAAGGTGTGGATCTTGACATCAAAGTGGGATCTTGTTATGTCTGAGACAAACCACATGTTGCACTCT
CTACATGGAGGCTTCTCATTTTCACCCCACAAGGAAGAAATCCCTGGCCTCAAACATTTTGTCAAGATGGCAAACCCTTC
CCACTACCCAGAAGACTTTTACTTTAGTAAATTATGGCTTTTCCATCTGGACTGCTCACTTGCAGGGTCATTTTGTGGAC
GTATTGGAACCTGCTCACGAAATACTTCCTTAGAGTTTCTTCCAGGACATATTGACCTGTTGACCATATCTGACTCCAGC
TATTTAGTCTACAATGCTGTCTACACAGTGGCTCATGTCCTCCATAAAATGCTTTTGGAGAAAGTAGAAATGGGATCTCC
AGGAGAAGCAGCCCAGCCCATGATTCTTCCCTGGCAGCTCCATCCATTTCTTAGGAAAATCCAATTTACAAACAGCGCTG
GAGAAGATGTATCCTTCCATGAAACAAGAAACCACATGGTGCATTACTATATCCAGAACGTTGTGAATTTTCCTAGTGGT
CTGCGTTTGCTTATTAAAATTGGAGAGTTTTCCTCCAAGAATCCACATGAACAAAGCTTACTTATCAATGAAGAGATGAT
AGAATGGCCTGTTGCATTCAAAGTGACTCCTCGATCTGTGTGTAGCCAGAGCTGTGGTCCTGGATTCAGGAAAATGTCAC
AGGAAGGAAGACCTATCTGCTGCTATACTTGTGTTTTGTGTCCCGAGAGAGAAATTGCCAATCAGACAGATGCAAAGCAG
TGCATGCAGTGTTCAGACGATGAGTACCCAAACCAGGAAAGAAATCGCTGCCTCCCTAAACTGGTGACCTTCTTAGCCTT
TGAAGAATCCCTGGGGATGGCTCTGGCCTGCATGGCTCTGTGCTTCTCTGTCCTCACAGCTGCGGTCCTCTGGGTCTTTG
TGAAGCACCGAGACACTCCCATTGTCAAGGCCAATAACAGGACCCTCAGCTACATCCTGCTTATCACCCTCCTCCTGTGC
TTCCTCTGCTCCTTACTCTTCCTTGGCCGTCCCAACACAGCCACCTGCCTCCTGCAACAAATAGCATTTGGCCTTGTGTT
CACAGTGGCTCTTTCCACTGTGCTGGCCAAGACCATCACTGTGATTCTGGCCTTCAAGGTTACAAAACCTGGAAGAACAA
TGAGGAGATTGCTGGTATCAGGCGTATATAACTCTGTTATTCCCATCTGCTCCTTGATCCAACTGGCTCTCTGTGGCATC
TGGTTGGGAACCTACCCTCCTTTTATTGAGTTAGATACACACTCTGAGCTTGACCACATCATCCTGGTGTGCAACAAGGG
CTCGGCCACTGCCTTCTACTGTGTCCTGGGATACCTGGGCTCCTTGGCCCTGGTGAGCTTCACTGTAGCTTTCCTAGCCA
GGAATCTGCCTGACACCTTCAATGAAGCCAAGTTCCTGACATTCAGCATGCTGGTGTTCTGCAGTGTCTGGGTGACCTTC
CTGCCTGTCTACCACAGCATCAAGGGGAAGGTCATGGTGGCCGTGGAGGTCTTCTCTATCTTGGCCTCCAGTGCAGGGCT
CCTAGGCTGCATCTTTTTTCCCAAGTGCTATGTTATTCTTATTAGACCTGAGAAGAACACTTTGAAAGGCTTAAAGAAAA
AAGCAAGTTCTAAAGGATTCTAA
