# Supplementary material for: Evolution of Spatially Coexpressed Families of Type-2 Vomeronasal Receptors in Rodents
Source: Genome Biol Evol. 2014 Dec 23;7(1):272–85. doi: 10.1093/gbe/evu283 (PMC4316634; doi:10.1093/gbe/evu283)
Supplement: Supplementary Data [file supp_evu283_Supplementary_file_S3.pdf]

Rodent V2RA sequences included in the tree of Suppl. Fig. 5S

```
>ms18_A10 .
GGGGGTCATTGTGTCTGATGATCTGAAGGGAATGGAGTTCCTCTCTCACCTGAAAGCAGAGATGGTATCAGAAGAC
ATCTGTGTGGCTTTTACGAAAAGCCTCAAAACAAATTGGAAGGTACAGTATACATCTGGTGTTGGATTGGCAAATT
TGAACCACCATCCACAAGTAAATGTGAACATACTCTATGGTGATATAGATGACTTGCTGTATTTTTGTTTGGAAAA
TAAACTTCTGTCAAGCAGAAGGAAGGTGTGGATCATGGCAAAGCTCCATTTAGTATACTTAGAGTCTGTATTCTTT
AAGAGGAAGGATTTGATAAACTTTTTCACAGGAAGCTTACTATTTTCCAAGAAGAGAAATATCCCTGGTTTCAAGA
ACTTTCTCGAATCACTCACACCCTCCTACTACCCAGGAGAATTTTACTTCTATAAATTCTGGATTGACAAGTTTGA
TTGCGCACCTCCTGCTTTGCTATGTGGAAGGAATAAACCCTGTCCACTAAATATTACTCTGAAGAATAAAGAAGGA
GAAAATGATATAATGATCCCTTCTGAGGCCAGCTATTCCATATGG
>ms80_A9 .
GGGACTGATCATCACAGATGACCACAGAGGTGCTCAGTTTCTATCAGACTTTAGAAAGGAGCTAGACAAAACTAGA
ATCTGCATAGCTTTCGTGCAAACAGTATTATATCTGGAGGAAACCCTACTTCATTTGCTATCCCAAAATCTTATCC
ACTTTCTAGAATCATCAACAGCAGATGTGATTGTAATTTATGGACCCACTTCTATTCTATTAACTTTAATAGAAAG
CACATATAGAAAATACAACATGAAAAAAATTTGGATTATGAATTCAAAATGGTTTTGTCCAAACTTGGAACTATAT
AATATGATAGAATTATCCCATGGGGCTCTCATTTTCTCCCCCCATTATGAGGAGATTACTGGTTTCAAAAAGTTTA
TGCAAGAAGCCACCCCAAACAAGTACCCAGAAGATATTTTTCTTCATTTATTGTGGTACTGGCATTTCAACTGCTC
ATTTTTGCATTCTGAATGTAAAATATTTGAAAACTGTCTGCAGAATGCCTCTTTGGAATTGTTGCCAGGGAACATT
TTTACAATGACCATGACTGAAGAGAGTTACAATGTGTAC
>ms81_A9 .
GGGACTCCTCATCCCAGATGACCAGAGAGGTGCTCAGTTTCTATCAGACTTTAGAAAGGAGCTAGACAAAACTAAA
ATCTGCATAGCTTTTGTGAAAACAGTATTATATCTGGGGGAAACCCTACTTCGTTTGATATCACAAGATCATATCC
ACATTATAGAATCATCAACAGCAGATGTGATTGTAATTTATGGACCCACTTCTATTCTATTAACTTTAATAGAAAA
TACATATAGAACGTACAACAAGAAAAAAATTTGGGTTATGAGTACAAAATGGTTTTGCCCAAATTTGGAACTATAT
AATATGTTAGAATTAACCCATGGGGCTCTGATTTTTTCACCTCATTATAAGGAGATTGTTGGTTTCAAAAAGTTTA
TGCTAGAAGCCACCCCCATCAAGTACCCAGAAGATATTTTTCTTCATTTATTGTGGTACTGGCATTTCAACTGCTC
ATTTTTGCATTCTGAATGTAAAATATTTGAAAACTGTCTGCAGAATGCCTCTTTGGAATTGTTGCCAGGAAATATT
TTTACAATGACCATGACTGAAGAGAGTTACAATGTGTAT
>ms82_A9 .
GGGACTCCTCATCCCAGCTGACCAGAGAGGTGCTCAGTTTTTATCAGACTTTAGAAAGGAGCTAGACAAAACTAGA
ATCTGCATAGCTTTTGTGCAAACAGTATTATATCTGGGGGAAACCCTACTTCGTTTGCTATCACAAGATCATATCC
ACATTCTAGAATCATCGACCGCAGACGTGATTGTAATTTATGGACCCACTTCTATTCTATTAACCTTAATAGAAAG
CACATATAGAAAGTACAACATGAAAAAAATTTGGGTTATGAATACAAAATGGTTTTGCCCCAATATAGAACTATAT
AATATGTTAGAATTATCCCATGGGGCTCTCATTTTTTCACCCCATTATGAGGAGATTGCTGGTTTCAAAAAGTTTA
TGCAAGAAGCCACCCCCATCAAGTACCCAGAAGATATTTTTCTTCATATATTGTGGTACTGGTATTTCAAATGCTC
ATTTTTGCATTTTGAATGTAAAATATTTAAAAACTGTCTGCAGAATGCCTCTTTGGAATTGTTGCCAGGGAATATT
TTTACAATGACCATGACTGAAGAGAGTTACTATGTGTAT
>ms83_A9 .
GGGACTGCTCAGCACAGATGACCACAGAGGTGCTCAGTTTCTATCAGACTTGAGAAAGGTGCTGGAAAATAATACA
GTCTGCATAGCTTTTATGGAAACAGTATCATTGTGGGGGGAATCCCTAAACTCTTTGCAAACCCATTATCAGATGC
ATATTCTAGAGTCATCAGCAAATGTGATTGTAATTTATGGATCTATTGCTTTCATATTAACTGTAATAAAAAATAG
ACATAGTAAGTACATAACGAAAAAAGTTTGGGTCATGAACTCAAAATGGGTTGGCCAAAAATTTGAACAATACACC
ATGTTAGAATTATCTCATGGGGCTCTCAGTTTTTCACACCATCATGGGGAAATTTTTGGTTTCACTAATTTTGTGC
ATGAAGCCACTCCTTTCAAGTACCCAGAAGACATTTTTCTTCATGTCTTATGGAACAAGTATTTCAATTGCTCACT
TTTGCAATCTGATTGTAAAATCATTGAAAAATGTCTGCCCAATTCCTCCTTGGAATTGTTACCAGGGAATATTTTT
GAAATGATCATGACTGAAGAGAGTTACAATGTGTAC
>ms57_A8 .
GGGTCTGATTGTCCCAAATGACCCCAGAGGGACTCAGATTCTGTCAGACTTTAGAGAAGTTATGGAAAGTAATAGG
ATCTGCTTAGCCTTTGTGAAAATGATTCCTGGCACCTGGAATTCATATTCTGATGCAATATGGAAAAATATGGAGA
AGATTCAGGAATCATCAGCAAATGTGATTATTATTTATGGGGACACTGTTTCTTTACAAGGTTTAATGCGACACAT
TGCACAACTGTTAGTGACATGGAAAGTCTGGGTCTTGAACTCTCAATGGGATATTGACTACTATTCTGATTATTTT
ATGATAGAATCCTTTCATGGGAGTCTCATTTTTTTCACACCATCATGAAGAGATGGTTGAGTTTGTGAATTTTGTTC
AAACAGTTAATCCCTACACATACCCAGAAGATGCTTATCTTCCTAAATTTTGGGTTTTTTTCTTCAATTGCTCTTT
TTCTGAGTTTGATTGCCAACTTTTAGAGAACTGCCAACCCAATGCATCTTTAGACTTACTCCCCAGACACATTTTT
GACCCAGCAATGAGTGAAGAGAGCTACAATATATAC
>ms58_A8 .
TGGTCTGATCATACCAGATGATCACAGAGGGACTCAGATACTATCAGACTTAAGAGAAAATATGGAAAACCATGGT
ATCTGCATAGCCTTTTTGAAAATTATCTCTAGTGCATGGAATGCATTTTCCAATGAACTATGGAAAAATATGTATA
AGATTGAGGAATCATCAGCAAATGTCATAGTTATTTATGGAGACATTATTTCTGTACAAGGTTTAATGCGACATAT
TGCACAACTGTTAGTGACATGGAAAGTCTGGGTCTTGAGCTCTTCATGGGATGTTGATACCCACTCTGATTATTTT
```

ATGGTAGAGTCATTTCATGGGAGTCTAGTTTTTTCACACCACCATGACGAGATGGTTGAGTTTATGAATTTTGTCC
AAACAGTTAATCCCTACAAATACCCAGAGGACAACTATCTTCCTAAGTTTTGGCATTTGTTTTTCAAGTGCTCATT
TTCTGAATTTAATTGTCAACTTTTGGAGAACTGCCAACCCAATGCTTCTTTGGATTTACTTCCAAGACACCATTTT
GACCCAGCCATGAATGAAGAAGGCTACAATATTTAC
>ms59_A8 .
TGGTCTGATCCTCCCAGATGACCACAGAGGGACTCAGATACTATTAGACTTGAGAGAAAATATGGAGAATCATGGC
ATCTGCATAGCCTTTCTGAAAATGATCTCTGGCACATGGAATGCATATTCCAATGAACAATGGAAAAATATGGAGA
AGATTGAGGAATCATCCGCAAATGTCATAGTTATTTATGGAGACATTACTTCTGTACAAGGTTTAATGCGACATAT
TGCACAACTGTTAGTGACATGGAAAGTCTGGGTTTTGAACTCTTCATGGGATGTTGATACCCACTCTGATTATTTT
ATGGTAGAGTCATTTCATGGGAGTTTAGTTTTTTCACACCACCGTGAAGAGATGGTTGAGTTTATGAATTTTGTCC
AAACAGTTAATCCCTACAAATACCCAGAGGACACCTATCTTCCTAAGTTTTGGCATTTGTTTTTCAAGTGCTCATT
TTCTGAATTTGATTGTCAACTTTTGGAGAACTGCCAACCCAATGCTTCTTTGGATTTACTTCCAAGACACCAATTT
GAGCCAGTCATGGGTGAAGAAGGCTACCATATATAT
>ms60_A8 .
TGGTCTGATCCTCCCAGATGACCACAGAGGGACTCAGATTCTTTCAGACTTAAGAGAAAATATGGAGAGTCATGGT
ATCTGCATAGCCTTTTTGAAAATGATCTCTGGCACATGGAATGCATTTTCCAATGCATTATGGAAAAGTATGGAGA
AGATTGAGGAATCATCAGCAAATGTCATAGTTATTTATGGAGACATTATTTCTGTACAAGGTTTAATGCTACATAT
TACACAACTGTTAGTGACATGGAAAGTCTGGGTCTTGAACTCTTCATGGGATGTTGATAGCCACTCTGATTATTTT
ATGGTAGAGTCATTTCATGGGAGTCTTATTTTCTCATACCACCATGAAGAGATGGTTGAGTTTATGAATTTTGTCC
AAACAGTTAATCCTTACAAATACCCAGAGGACAACTATCTTCCTAAGTTTTGGCATTTGTTTTTCAAGTGCTCATT
TTCTAAATTTGATTGTCAGCTTTTGGAGAACTGCCAACCCAATGCTTCTTTGGATTTACTTCCAAGACACCTTTTT
GACCCAGCCATGAGTGAGGAAGGCTACAATATATAC
>ms61_A8 .
TGGTCTGATCCTCCCAGATGATCACAGAGGGATTCAGATTCTATCAGACTTGAGAGAAAATATGGAGAGTCATAGG
ATCTGCATAGCCTTTTTGAAAATGATCTCTGGCACATGGAATGCATTTTCCAATGCATTATGGAAAAATATGGAGA
CAATTGAGGAATCATCAGCCAATGTCATACTTATTTATGGAGACATTATTTCTGTACAAGGTTTAATGCGACATAT
TGCACAACTGTTAGTGACATGGAAAGTCTGGGTCTTGACCTCTTCATGGGATGTTGATACACACTCTGATTATTTT
ATGGTAGAGTCATTTCATGGGAGTCTCATTTTTTCACACCACCATGAAGACATGGTTGAGTTTATGAAGTTTGTCC
AAACAGTTAATCCCTACAAATACCCAGAGGACAATTATCTTCCTAAGTTTTGGCATTTGTTTTTCAAGTGCTCATT
TTCCAAATTTGATTGTCAACTTTTGGAGAACTGCCAACCCAATGCTTCTTTGGATTTACTTCCCAGACACCTTTTT
GACCCAGCCATGAGTGAGGAAGGCTACAATATATAC
>ms62_A8 .
GGGTCTGATTGTCCCAAATGACCCCAGAGGGACTCAGATTCTATCAGAGTTTAGAGATGTTATGGAAAGTAATAGG
ATCTGCTTAGCCTTTGTGAAAATGATTCCTGGCACCTGGAATTCATATTCTGATTCAATATGGAAAAATATGGAGA
AGATTCAGGAATCATCAGCAAATGTGATTGTTATTTATGGGGACATTGTTTCTTTACAAGGTTTAATGCGACACAT
TGCACAACTGTTAGTGACATGGAAAGTCTGGGTCTTGAACTCTCAATGGGATATTGACTACTATTCTGATTATTTT
ATGATAGAATCCTTTCATGGGAGTCTCATTTTTTCACACCATCATGAAGAGATGGTTGAGTTTGTGAATTTTGTTC
AAACAGTGAATCCCTACACATACCCTGAAGATGATTATCTTCCTAAGTTTTGGGTTTTTTTCTTCAAGTGCTCTTT
TTCTGAGTTTGATTGCCAACTTTTGGAGAACTGCCAACCCAATGCATCTTTGGACTTACTGCCCAGACACATTTTT
GACCCAGCCATGAGTGAAGAGAGCTACAACCTATAC
>ms63_A8 .
GGGTCTGATTGTCCCAAATGACCCCAGAGGGACTCAGATTCTATCAGAGTTTAGAGACGTTATGGAAAGTAATAGG
ATCTGTTTAGCCTTTGTGAAAATGATTCCTGGCACCTGGAATTCATATTCTGATGCAATATGGAAAAATATGGAGA
AGATTCAGGAATCATCAGCAAATGTGATTGTTATTTGTGGAGACATTGTTTCTTTACAAGGTTTAATGCGACACAT
TGCACAACTGTTAGTGACATGGAAAGTCTGGGTCTTGAACTCTCAATGGGATATTGACTACTATTCTGATTATTTT
ATGATAGAATCCTTTCATGGGAGTCTCATTTTTTCACACCATCATGAAGAGATGGTTGAGTTTGTGAATTTTGTTC
AAACAGTGAATCCCTACACATACCCAGAAGATGCTTATCTTCCTAAATTTTGGGTTATTTTCTTCAATTGCTCTTT
TTCTGAATTTGATTGCCAACTTTTGGAGAACTGCCAACCCAATGCATCTTTGGACTTACTTCCCAGACACATTTTT
GACCCAACCATGAGTGAAGAGAGCTACAACATATAC
>ms90_A8 .
TGGTCTGATTATCCCAGATGACCACAAAGGGACACAGATTTTGTCTGACTTGAGACAAGTTATGGAGAGAAATAAC
ATTTGCATAGCCTCTGTGCAAATGATCCCTGGCATCTGGAATTCATTTTCAAATGCATTATGGAAAAGTCTGGTCC
AGACTAAGGAATCATCAGCAAATGTGACAGTTGTTTGTGGTGACATTGTTTCTCTACAAGGTTTAATGCGACATAT
TGCACAGTTGTTAGTGACTTGGAAAGTCTGGGTCTTAAACTCTCAATGGGATGTTGACACCCATTCTGATTATTTC
ATGTTAGAGTCATTACATGGAAGTTTCATTTTTTCACACCACCATGAAGAGATGGCTGAATTTACAAATTTTATTA
GAACAGTTAACCCCTACAAATACCCAGAAGACAATTATCTTCCTAAGTTTTGGTATTTATTCTTCAAGTGCTCATT
TTCTGAGTTTGATTGCCAACTTTTGGAAAACTGCCAACCCAATGCTTCTTTGGAGTTACTGCCAAGATACCTTTTT
GACCCAGTCATGAGTGAAGAGAGCTACAATATATAT
>ms91_A8 .
TGGTCTCATCATCCCAGATGACCACAAAGGAAATAAAATTCTATCAGACTTTAGGGAGGAGATGGAGAGAAAGGGA
ATCTGTATAGCTTTTGTAAAAATGATCCCAGAAACATGGAATTTATATTTTGCCAAATTCTGGGAAAATATGGATG
AGACAAATGTAATAATTATTTATGGTGACACTGATTCTCTAGCAAGTCTAATGAGAAATATTGGGCAAAGGTTATT

GACATGGAATGTCTGGGTCATGAACATTGAACATCATGTTATAGACACAGCTGATTATTTCATGTTAGACTTATTC
CATGGAAGCCTAATTTTTAAGCACCATTATAGGGAGAATTTTGAATTTACCAAATTTATTCAAACAGTTAATCCCA
ATAAATACCCAGAAGACATTTATCTTCCTAAGCTGTGGTATTTCTTCTTCAAGTGCTCATTTGCTGGCATTAATTG
TCATGTTTTGGCCAACTGTCAAACCAATGCCTCTTTGGATATTTTACCTAGTCACATATTTGATGTGGCCATGAAT
GATGAGAGCAGAAATATTTAC
>ms92_A8 .
TGGTCTCATCATCCCAGATGACCACAAAGGAAATAAAATTCTATCAGATTTTAGGGAGGAGATGGAGAGAAAGGGA
ATCTGTATAGCTTTTGTAAAAATGATCCCAGAAACATGGAATTTATATTTTGCCAAATTCTGGGAAAATATGGATG
AGACAAATGTAATAATTATTTATGGTGACACTGATTCTCTAGCAAGTCTAATGAGAAATATTGGGCAAAGGTTATT
GACATGGAATGTCTGGGTCATGAACATTGAACATCATGTTATAGACACAGCTGATTATTTCATGTTAGACTTATTC
CATGGAAGCCTAATTTTTAAGCACCATTATAGGGAGAATTTTGAATTTACCAAATTTATTCAAACAGTTAATCCCA
ATAAATACCCAGAAGACATTTATCTTCCTAAGCTGTGGTATTTCTTCTTCAAGTGCTCATTTGCTGGCATTAATTG
TCATGTTTTGGCCAACTGTCAAACCAATGCCTCTTTGGATATTTTACCTAGTCACATATTTGATGTGGCCATGAAT
GATGAGAGCAGAAATATTTAC
>ms94_A8 .
TGGTCTCATTCTCCCCGATAACCACAAAGGAAATAAAATTCTATCAGATTTTAGGGAGGAGATGGAGAGAAACAGA
ATCTGCATAGCTTTTGTAAAAATGATCCCAGCCACATGGACTGCATATTTTACAAGCTTCTGGGAAAATATGGAAG
AGACAAATGTAATAATTATTTATGGTGACATTGATTCTCTAGAAGGTCTAATGAGAAATATTGGGCAAAGGATATT
GACATGGATTGTCTGGGTCATGAACATTGAACATACTATTACTTACGATAATGATTATTTCATGTTAGACTCATTC
CATGGAAGCCTAATTTTTAGGCACAATTATAGAGAGAATTTTGAATTTACCAAATTTATTCAAACAGTTAATCCTA
ATAAATACCCTGAAGACATTTATCTACCTAAGTTGTGGAATTTCTTCTTCAAGTGCTCATTTTCTGACACTAATTG
TCATGTTTTGGACAAGTGTCAAACCAATGCTTCTTTGGATCTATTACCTAGACACATATTTGATGTTGTCATGAGT
GAAGAGAGCACAAATATTTAC
>ms93_A8 .
TGGTCTCATCCTCCCAGATGACCACAAGGGAAATAAAATTCTATCAGATTTTAGGGAGGAGATGGAGAGGAAAAGA
ATCTGCATGGCTTTTGTAAAAATGATCCCAGCCACATGGACTTCATACTTTGCCAAATTCTGGAAAAATATGGATG
AGACAAATGTAATAATTATTTATGGTGACATTGATTCTCTAGAAGGTCTAATGAGAAATATTGGGCAAAGGTTATT
GACATGGATTGTCTGGGTCATGAACACTGAACACCATGTTCTTGAAATTTCTGATTATTTCATGTTAGACTCATTC
CATGGAAGCTTAATTTTTAAGCACCATTACAGGGAGAATTCTGATTTTACTAAATTTATTCAAACAGTTAATCCTA
AAAACTACCCAGAAGACATTTATCTTCCTAAGTTGTGGTATTACTTCTTCAAATGCTCATTTTCTGATATTAATTG
TCATGTTTTAGACAATTGTCAAACTAATGCTTCTTTGGATGTATTACCTAGTCACATATTTGATGTGGCTATGACT
GAAGAGAGCACAAGTATTTAT
>ms95_A8 .
TGGTCTCATCCTCCCCGATGACCACAAAGGTAATAAAATTCTATCAGATTTTAGGGAGGAGATGGAGAGAAAAGAT
ATCTGCCTAGCTTTTGTAAAAATGACCTCAGAAACATGGACTTCATATTTTTATAAATTCTGGGAAAATATCGATG
AGACAAATGTAACAATTATTTATGGTGACATTGATTCACTAGAAGGTGTAATGAGGAATATTGAGCAAAGGTTATT
GACATGGAATATATGGATCATGAACATTGAACATCATGTTATTGACAGAGCTGACTATTTCATGTTAGACTCATTC
CATGGAAGCCTAATTTTTAAGCACCATTATAGGGAGAATTTTGAGTTTACCAAATTTATTCAAAGAGTTAATCCCA
ATAAATACCCAGAAGATATTTATCTTCCTAAGCTGTGGTATTTGTTCTTCAAGTGCTCATTTTCTGCCACTAATTG
TCATGTTTTGGAAAACTGTCAAACCAATGCTTCTTTGGATGTATTCCCTCGTCACATATTTGATGTGGCAATGAAT
GCAGAGAGCACAAGTATTTAC
>ms96_A8 .
TGGTCTCATCCTTCCCAATGACCACAAAGGAAATAAAATTCTATCAGATTTTAGGGAGGAGATGGAGAGAAATGGC
ATCTGCCTAGCTTTTGTAAAAATGACCCCAGAAACATGGACTTCATATGTTGCCAAATTCTGGGAAAATATGGATG
AGACTAATGTAACAATTATTTATGGTGAGATTGATTCACTGGAAGGTATAATGAGGAATATTGAGCAAAGGTTATT
GACATGGAATGTGTGGATCATGAACATTGAACATCATGTTATTGACAGAGCAGATTATTTCATGTTAGACTCATTC
CATGGGAGCCTCATTTTTAAGCACAATTATAGAGAGAATATTGAATTTACCAAATTTATTCAAACAGTTAATCCCA
ATAAATACCCAGAAGACATTTATCTTCCTAAGCTGTGGTATTTGTTTTTCAAGTGTTCATTTTCTGACACTAATTG
TCATGTGTTGGAAAACTGTCAAACCAATGCTACCTTGGATATATTCCCTCGTCACATATTTGATATGGTCATGAAT
GCAGAGAGCACAATTATTTAC
>ms97_A8 .
TGGTCTCATCCTTCCCGATGACCACAAAGGAAATAAAATTCTATCAGATTTTAGGGAGGAGATGGAGAGAAATGGC
ATCTGCCTAGCTTTTGTAAAAATGACCTCAGAAACATGGACTTCATATGTTGCCAAATTCTGGGAAAATATGCATG
AGACTAATGTAACAATTATTTATGGTGAGAATGATTCATTGGAAGGTATAATTAGGAATATTGAGCAAAGGTTATT
GACATGGAATGTCTGGATCATGAACATTGAACATCATGTTATTGACAGAGCTGATTATTTCATGTTAGACTCATTC
CATGGGAGCCTCATTTTTAAGCACAATTATAGAGAGAATTTTGAATTTACCAAATTTATTCAAACAGTTAATCCCA
ATAAATACCCAGAAGATATTTATCTTCCTAAGCTGTGGTATTTGTTCTTCAAGTGCTCATTTTCTGACACTAATTG
TCATGTGTTGGAAAACTGTCAAACCAATGCTTCTTTGGATGTATTCCCTCGTCACATATTTGATGTGGCCATGAAT
GCAGAAAGCACAATTATTTAC
>ms98_A8 .
TGGTCTAATCCTCCCCAATGACCACAAAGGAAATAAAATACTATCAGATTTTAGGGAGGAGATGGAGAGAAATGGC
ATCTGCCTAGCTTTTGTAAAAATGACCTCAGACACATGGACTTCATATGTTGCCAAATTCTGGGAAAATATGGATG

AAACAAATGTAACAATTATTTATGGTGACATTGATTCACTAGAAGGTATAATGAGGAATATTGAGCAAAGGTTATT
GACATGGAATATATGGATCATGAACATTGAACATCATGTTATTGACAGAGCTGATTATTTCATATTAGACTCATTC
CATGGGAGCCTCATTTTTAAGCACTCTTATAGAGAGAATTTTGAATTCACCAAATTTATTCAAACAGTTAATCCCA
ATAAATACCCAGAAGACGTTTATCTTCCTAAGCTGTGGTATTTGTTCTTCAAGTGCTCATTTTCTGACACTAATTG
TCATGTGTTGGGAAACTGTCAAACCAATGCTTCTTTGGAAGTATTCCCTAGTCACATATTTGATGTGGCCATGAAT
GCCGAGAGCACAAGTATTTAC
>ms99_A8 .
TGGCCTCATCCTCCCTGATGACCACAAAGGAAATATAATTCTATCAGATTTTAGTGAGGAGATGGAGAGAAAAGGC
ATCTGCCTAGCTTTTGTAAAAATGACCCCCGCCACATGGACTTCATATTTTGCCAAATTCTGGGAAAATATGGATG
AGACAAATGTAACAATTATTTATGGTGACATTGATTCGCTAGAAGGTGTAATGAGGAATATTGAGCAAAGGTTATT
GACATGGAATGTCTGGATCATGAACATTGAACATCATATTATTGACATAGCTGATTATTTCATGTTAGACCCATTC
CATGGAAGCCTAATTTTTAAGCACAATTATAGAGAGAATTTTGAATTTACCAAATTTATTCAAACAGTTAATCCCA
ATAAATACCCAGAAGACATTTATCTTCCTAAGCTGTGGCATTTGTTCTTCAAGTGCTCATTTTCTGACATTAATTG
TCATGTGTTGGAAAACTGTCAAACCAATGCTTCTTTGGATGTATTCCCTCGTCACATATTTGATGGGTCTATGAGT
GATGAGAGTAGAAGTATTTAC
>ms100_A8 .
TGGTCTCATTCTTCCTGATGACCACAAAGGAAATAAAGTGCTATCAGATTTTAAACAGGAGATGGAGAGAAGAAGA
ATCTGCATAGCTTTTGTAAAAATGATTCCAGCCACATGGACTTCATATTTTACCAAATTCTGGCAAAATATGGAAG
AGACAAATGTAATAATTATTTATGGTGACATTGATTCTCTAGAAGGTCTAGTGAGAAATATTGGGCAAAGGTTATT
GACATGGAATGTTTGGGTCATGAACGTTGAATACAATATTAAATTAATTACTGATTATTTCTTGTTAGACTCATTT
CATGGAAGCCTTATTTTTAAGCACAACTTTAGAGAGAATTTTGAGTTTACCAAGTTTATTCAAACAGTCAATCCTA
GTAAATACCCAAAAGACATTTATCTTCCTAAGTTGTGGTATTTGTTCTTCAAGTGCTCATTTGCTGATGTTAATTG
TCATGTGTTGGACAACTGTCAAAGCAATGCTTCTTTAGATATATTACCTAGTCACATATTTGATGTGGCCATGAGT
GAAGAGAGCACAAGTATTTAC
>ms101_A8 .
TGGTCTCATACTCCCTGATGACCACAAAGGAAATAAGATTGTATCAGATTTTAGGGACGAGATGGAGAGAAAAGGC
ATCTGCCTAGCTTTTGTAAAAATGATCCCAGGCACATGGACTTCACATTTTGCCAAATTCTGGGAACATATGGATG
AGACAAATGTAACAATTGTTTATGGTGATGTTGATTCGCTAGAAGGTGTAATGAGGAATATTGAACAAAGGTTATT
GACACGGAATGTCTGGAACATGAACATTGAACACCATGTTATTGACAGAGCTGACTATTTCATGTTAGACTCATTC
CATGGAAGCCTAATTTTTAAGCACAATTACAAAGAGAATTTTGATTTTACCAAATTTATTCAAACAGTTAATCCTA
ATAAATACCCAGAAGACATTTATCTTCCTAAGCTGTGGTATTTATTTTTCAAGTGCTCATTTGCTGACATTAATTG
TCAAGTTTTGAACAACTGTCAAACCAATGCTTCTTTCGATATATTACCTCGACACATATTTGATGTGGTCATGAAT
GCAGAGAGCACAAGTATTTAC
>ms102_A8 .
TGGTCTCATACTCCCTGATGACCACAAAGGAAATAAGATTGTATCAGATTTTAGGGAGGAGATGGAGAGAAAAGGC
ATCTGCCTAGCTTTTGTAAAAATGATCCCAGCCACATGGACTTCACATTTTGCCAAATTCTGGGAACATATGGATG
AGACAAATGTAACAATTGTTTATGGTGATGTTGATTCACTAGAAGGTGTAATAAGGAATATTGAACAAAGGTTATT
GACACAGAATGTCTGGATCATGAACATTGAACATCATGTTATTGACAGAGCTGACTATTTCATGTTAGACACATTC
CATGGAAGCCTAATTTTTAAGCACAGTTATAGAGAGAATTTTGAGTTTACCAAATTTATTAAAACAGTTAATCCTA
AAAAATACCCAGAAGACATTTATCTTCCTAAGCTGTGGCATTTATTTTTCAAGTGCTCATTTGCTGACATTAATTG
CAATGTTTTGGACAACTGCCAAACCAATGCTTCCTTGGATGTATTCCCTCGTCACATATTTGATGTGGCCATGAAT
GAAGAGAGCTCTAGTATTTAC
>ms103_A8 .
TGGTCTCATACTCCCTGATGACCACAAAGGAAATAATATTGTATCAGATTTTCGGGAGGAGATGGAGAGAAAAGGC
ATCTGCCTAGCTTTTGTAAAAATGATCCCAGCCACATGGACTTCACATTTTGCCAAATTCTGGGAACATATGGATG
AGACAAATGTAACAATTGTTTATGGAGATGTTGATTCCCTAGAAGGTATAATGAGGAATATTGAACAAAGGTTATT
GACACAGAATGTCTGGATCATGAACATTGAACATCATGTTATTGACAGAGCTGACTATTTCATGTTAGACTCATTC
CATGGAAGCCTAATTTTTAAGCACAATTACAAAGAGAATTTTGAGTTTACCAAATTTATTCAAACAGTTAATCCTA
ATAAATACCCAGAAGATATTTATCTTCCTAAGCTGTGGCATTTGTTTTTCAAGTGCTCATTTGCTGACATTAATTG
TAATGTTTTGGAAAACTGTCAAACCAATGCTTCTTTGGATGTATTCCCTCGTCACATATTTGATTTATCCATGAAT
GCAGAGAGCACAAGTATTTAC
>ms104_A8 .
TGGCCTCATCATCCCCGATAACCACAAAGGAAATAAAATTCTATCAGAATTTAGGGAGGAGATGGAGAGAAACAAA
ATCTGCATAGCCTTTTTAAAAATGATCCCAGCCACATGGACCTCATATTTTACTAGATTCTGGGAAAATATGGATG
AGACAAATGTAATAATTATTTATGGTGACATTGATTCTTTAGAAATTCTTATGCAAATTATTGGGCAAAGGTTATT
GACATGGAACATCTGGATCATGAACAATGAACCCCATATTATTGAACTTAGTGATTATTTCATGTTAGACTCATTC
CATGGAAGTTTCATTTTTAGACACAATTATAGAGACAACATTGAGTTTACCAAATTTATTCAAAGAGTTAATCCAT
ATAAATACCCTGAAGACATTTATCTTCCTAAGTTGTGGAATTTCTTCTTCAAGTGTTCATTTTCTGACATTAATTG
TCATGTTTTAGACAACTGTAAAACCAATGCTTCTTTGGATCTATTACCCAGTTTCATATTTGATGTTGTCATGAGT
GAAGAGAGCACAAACATTTAC
>ms105_A8 .

TGGTCTCATTCTTCCAGATGACCACAAAGGAAATAAAATTCTATCAGATTTCAAAAATGAGATGGAGAGAAAAGGA
ATCTGCATAGCTTTTGTGAAAATGATCCCTGCCACCTGGTCTTCACATTTAAACCAATTCTGGAAAAATATAGATG
AAACAAATGTCATAATTATTTATGGTGACATTGATTCTCTAGAAGGTCTAATGAGAAATATTAGGCAAAGGTTATT
GACAGGGAAAGTCTGGGTCATGAACATTGAACCTCATTTTACTGACTATGCTGATTATTTCTTCTTAGACTCATTT
CATGGGAGCCTTATCTTTACATACCATTATTCAGAAAGATTTGAGTTTACAAATTTTATTCAAACAGTTAATCCCT
ACAAATATCCAGAAGATATTTATCTTCCTAAGTTGTGGCATTTGTTCTTCAAGTGTTCATTTTCTGATAATGGTTG
TCAACTTTTGAACAACTGTCAGTTCAATGCTTCTTTGGATGCATTGCCTCGTCACATATTTGATGAGTCCATGAGT
GATGAGAGTACCAGCATATAC
>ms106_A8 .
TGGTCTCATCCTTCCAGATGACCACAAAGGGAATAAAATTCTACCAGAAATCAAAGAAGACATGCAGAGAAAAGGA
GTCTGTATAGCTTTTGTTAAAATGATCCCTGCCACCTGGACTTCACATTTTAGCAAATTCTGGGAAAATATGGATG
AGGTAAATGTCATAATTATATATGGTGACATTGATTCTCTAGAAATTCTAATGAGAAATATTGGACAAAGGTTATT
GACATTGAAAGTCTGGGTCTTGAACATTGAACCACATGTTACTGACTATGCTGATTATTTCTTGTTAGACTCATTC
CATGGGAGCTTAATTTTTACTCACCATTACAGAAAGAATTTTGAGTTCACCAATTTCATTCAAACAGTTAATCCTT
ACAAATACCCAGAAGATATTTATCTTCCTAAGTTGTGGCATTTGTTCTTCAAGTGCTCATTTTCTGATATTGATTG
TCAACTATTGGCTAACTGTCAAGCCAATGCTTCTTTGGATGTATTGCCTTGTCATATATTTGACATAGCGATTAGT
GAAGAGAGCAAGACTATCTAT
>ms107_A8 .
TGGTCTAATTCTCCCAGATGACCACAAAGGAAATAAAATACTATCAGATTTTAGAAAGGAGATGGAGAGAAAAAGA
ATCTGTACGGCTTTTGTAAAAATGATTCCTGCCACATGGACTTCATCTTTTGTCAAATTCTGGGAAAATATGGATG
ACACCAACATAATAATTATTTATGGTGACATTGATTCTCTAGAAGGTCTAATGCGAAATATTGGGCAAAGGTTATT
GACATGGCATGTCTGGGTCATGAACATTGAACCCCATATTATTGAATATGATAATTATTTCATGTTAGATTCATTC
CATGGAAGTTTAATTTTTAAGCACAATTATAGAGAGAATTTTGAGTTTACCAAATTTATTCGAACAGTTAATCCTA
AAAAATACCCAGAAGACATTTATCTCCCTAAGATGTGGTATTTGTTCTTCATGTGCTCATTTTCTGATATTAATTG
TCAAGTTTTGGACAGCTGTCAAACAAATGCTTCTTTGGATATGTTACCTAGTCAGATATTTGATGTGGTCATGAGT
GAAGAGAGCACAAGTATTTAC
>ms108_A8 .
TGGTCTCATCCTTCCAGATAACCACAAAGGAAATAAAATTTTATCAGAGTTCAGAGAAGATATGGAGAGAAAAGGA
GTCTGCATAGCTTTTGTGAAAATGATCCCTATGACATGGAATGCATATTATAACAAATTCTGGGAAAATATGGATG
AGACAAATGTTATAATTATTTATGGTGACATTGATTCTCTCACAGGTCTAATGCGAAATATTGGGCAAAGATTGTT
GACAGCAAAAGTCTGGATCATGAACACTGAACCTCATATTACTGACTATGCTGACTATTTCATGCTTGACTCATTC
CATGGTAGCTTAATTTTTACACACAATTATAGAAAGAGTTTTAAGATTTCTAATTTTATTCAAACAGTTAATCCTT
ACAAATACCCTGAAGACATTTACCTTCCTAAGTTATGGCATTTGTTCTTCAAATGCTCATTTTCTGATGTTGATTG
TCGACTTTTGGCTAACTGTCAACCCAATGCTTCTCTGGATGTGTTACCTAGTCATATATTGGACATGGTAATTAGT
GAAGAAAGCAATAATATCTAT
>ms109_A8 .
TGGTCTCATACTCCCAGATGATCACAAAGGAAATATAATTTTATCTGAGTTCAGAGAAGATATGGATAGAAAAGGC
ATCTGCATAGCTTTTGTGAAAATGATCCCTGACACGTGGGCTTCATATATTAACAAATTCTGGGAAAATATGGATG
AGACAAATGTCATAATTATTTATGGTGACATTGATTCTCTCATAGGTCTAATGAGAAATATTGGTGAAAGATTGTT
GACAGGGAAAGTCTGGGTCATGAACATTGAACCTTATATTACTGACTATGCTGATTATTTCATGTTTGACTCATTC
CATGGTAGCCTAATTTTTACTTACCATTATAGAGAAAGTTTTGAGCTTACTAAGTTTATTCAAACAGTTAATCCTT
ACAAATACCCAGAAGACATTTATCTTCCTAAGTTATGGCATTTGTTCTTCAAGTGCTCATTTTCTGATATTAATTG
TCAACTGTTGGCTGACTGTCACTCCAATGCTTCTCTGGATATGTTACCTAGTCATATATTGGACATGACAATCAGT
GAAGAAAGCAACAATATCTAT
>ms110_A8 .
TGGTCTCATCCTCCCAGATGACCACAAAGGGTATATAATTTTATCAGAACTCAGAGAAGATATGGAGAGAAAAGGA
GTCTGCATAGCTTTTGTGAAAATGATCCTTCTCACATTGACTTCATATTATAACAAATTCTGGGAAAATATGGATA
AGACAAATGTCATAATTATTTACGGTGATGTTGATTCACTCACAGGTCTAATGAGAAATATTGGGCAAAGATTGTT
GACAGCAAAAGTCTGGGTCATGAATATTGAACCTCATATTACTGACTATGCTGACTATTTCATGTTTGACTCTTTC
CATGGTAGCCTAATTTTTACCCACAAGTATAGAGAGAGCTTTGATCTTACTACATTTATTCAAACAGTTAATCCTT
ACAAATACCCTGAAGACATTTACCTTCCTAAGTTATGGCATTTGTTCTTCAAGTGCTCATTTTCTGATATTGATTG
TCTACTATTGGCTAACTGTCAATCCAATGCTTCTCTGGATGTGTTACCTAGTCATATATTGGACATGACAATCAGT
GAAGAAAGCAATAATATCTAT
>ms120_A6 .
GGGGCTGATCATCACAGAGAATGAAAAGGGTATTAAATTTCTGTCAGACTTGAAAAGAGAGATGAAGAAAAACAGT
GTGTGTGCAGCCTTTGTACAAATGCTCAGTCTCAAATACCGGTACAACTGGAATGATCTGCTCAGCACTGATCCTG
CAATCATGGGGGAAATGGTAAAGGTGTTCATTGTATTTGCTGATATTGACTCTACTCTAGTTGTGATATTTAAAGC
ATCAGGACAAGTAGACCCGTGGAGGGTGTGGGTCACAACCTCACAGTGGGATATTGCTAGCAGTGTAAGACATTTC
ATCCTGGACTCATTCCATGGAACCCTCATTTTTTCACAACATCATCCTGAAATTTCTGCTTTTAAAGATTTTATAC
AGACTGTAAACCCATCCAAATATCCAGATGATATCTTTCTTTCACATATATGGAAAATGTATTTGAATTGTCCATT
TTCACGGGTTTACTGTAAAACACTGAAAACTTGTTCATCCAATGGATCCTTGGCATTGTTACCTTGGCACCGATTT
GACATGGACATGAGTGAAGAGAGTTATCACATATAT

>ms65_A5 .
GGGACTGGTCATTTCAGATGATGAACTAGGCATTGAGTTTCTCTTTAAGTTGAGAGGAGAGATGCACAAACACAAA
GTCTGTATAGCCTTTGTGAATATGATAATACAAAATATACAACTATATCAGAAAAGAGCTGAAAAGTATTATAACC
AGATCTTGACGTCATCAGCAAAAGTTATTATCATTTATGGTGACTCGGACATTCTAACTGTACACTTTAGACTGTG
GCAACATTTAGGTATTAAGAGACTCTGGATTACCACCTCACATTGGGATGAGAACACAAGTAAAGGAGACTTACTT
CTTTCCTCCTTTAATGGGATTTTCATTTTTTCACATCCCCATTCTAAGATTCCTGGTTTTAAGAAATTCATCCAGA
CAGTGCACCCTTCCAACTACAGTAAGGATACTTCCCTTGCTAGGTTGTGGTGGCTGTATTTCAACTGTTCATTGCC
ATCTCATTGTAAGACACTGAAAAATTGTTCAACCAAAATCCTACTAGAATGGTTATCCAGGCACCAGTTTGAAGTG
TCAATGAGCGAGCCAAGTTACAACCTATAC
>ms66_A5 .
AGGAGCGATTGTTACCAATGATGACCATGGAATTCAATTCCTTTCTGAATTAAGAGGAGAAATGCAAAAACACATT
GTCTGTTTATCAGTTGCAATTATTATCCAAACTGAGAAGATCATGGCCCTTAAAGAGTTTCATACGCATTACAACC
AAATTATAATGTCATCAGCAAAAGTTGTGATAGTTTATGGAGACAAAGACTCTCCTATACATTTTGCCCTCATTGT
GTGGAAATCTAAAGGCATTTGGAGAATCTGGGTTAGTGTCTCACAATTTGATATGATCACAATTATAGGAGATTTC
TTGCTATACTCCTCCATAGGGACTTTCATTTTTTCACACCAGCAACCTGAAATACCTGGTTTTGAGCAATTTATCC
AAACAGTACACCCTTCAAACTACAGTAGTGAACATTCCCTTGCCAAACTATGGTGGACGTATTTTAGATGTTCTTT
GCCACCATCTAATTGTAAGAAGCTGAAGAATTGTCCAACCACAACTGTATTTAAATGGTTATTCATGACACCACTT
GGAATGGCCATGAGTAATACATGCTATAATTTATAT
>ms67_A5 .
AGGAGCGATTGTTACAACTGATGACCATGGAATTCAATTCCTTTCTGAATTGAGAGGAGAAATGCAAAAACACATT
GTCTGTTTATCGTTTGAGATTACTATCCTAACTGAGCATAGCATGGCCCGTAAAGAGTTTCAAAGGTATTTTAACA
GAATTGTAATGTCATCAGCAAAAGTTGTGATAGTGTATGGAGACTACACCTCTCCCATAGATCTTGTCCTCCATTT
GTGTAAATCCAAAGGCATTTTTAGAATCTGGGTTAGTGTGTCACAATTTGATATGATCACAAAACTAGGAGATTTC
ATGCTATATTCCTCCTCTGGGACATTCATTTTTTCACACCAGAAACCTGAACTATCTGGTTTTCAACAATTTATCC
AAACAGTACACCCTTCAAATTACAGTAGTGAATTTTCCTTTGCCAAACTATGGTGGACATATTTTAGGTGTTCTTT
GCCACCATCTAATTGTAAGAAGCTGAAGAATTGTCCAACCAAAATTGTATTTAAATGGTTATTCATGACACCACTT
GGAATGGCCATGAGTGATACATGCTATAACTTATAC
>ms68_A5 .
AGGAGCGATGATTACAAATGATGACCATGGAATTCAATTTCTTTCTAAATTGAGAGGAGAAATGCAAAAACACATT
GTCTGTTTATCGTTTGTGATTACTATCCTAACTGAGCATAGCATGGCCCATAAAGAGTTTCAAAGGTATTTTAACA
GAATTGTAATGTCATCAGCAAAAGTTGTGATAGTGTATGGAGACTACACCTCTCCCATAGATCTTGTCCTCCATTT
GTGTAAATCCAAAGGCATTTTTAGAATCTGGGTTAGTGTGTCACAATTTGATATGATCACATATCTAGGAGATTTC
ATGCTATATTCCTCCTCTGGGACATTCATTTTTTCACACCAGAAACCTGAACTATCTGGTTTTCAACAATTTATCC
AAACAGTACACCCTTCAAATTACAGTAGTGAATTTTCCTTTGCCAAACTATGGTGGACATATTTTAGGTGTTCTTT
GCCACCATTTAATTGTAAGAAGCTGAAGAATTGTCCAACCAAAATTGTATTTAAATGGTTATTCATGACACCACTT
GGAATGGCCATGAGTGATACATGCTATAACTTATAC
>ms69_A5 .
AGGAGTGATTGTTACAAGTGATGACCAAGGAATACAATTCCTTTCTGAATTGAAAGGAGAAATGCAAAAAAACATT
GTTTGTTTATCAGTTGCTATTATTATCCAAACTGAGATGTCCATGGCTCTTAAAGAGTTAAGTATGAATTATAAAC
ATATCTCAATATCATCAGCAAAAGTTGTGATAGTTTATGGAGACAAATTTTCACCCATAAATTATGCCCTCACATT
GTGGATATCTCAAGGCATTTTAAGAATCTGGGTTAGTGTGTCACAATTTGATATGATCACAATTCTAGGAGATTTC
TTGCTATACTCCTCTACTGGTACTTTTATTTTTTCACACCAGCAACCTGAGATACCTGGTTTTGAAAAATTTATCC
AAACAGTATACCCTTCAAACTACAGTAGTGAATTTTCCTTTGCCAAACTATGGTGGACATATTTTAGATGTTCATT
GCCACCATCGAATTGTAAGAAGCTGAAGAATTGTCCAACCAAAACTATATTTACATGGTTATTCAGGACACCATTT
GGAATGGCCATGAGTGATACATGCTATAACTCATAC
>ms70_A5 .
AGGAGTAATCATTACAAATGATGACCATGGAATTCAATTCCTTTCTGAATTGAGAGGAGAAATGCAAAACAACATT
GTCTGTTTATCAGTTGTTATTACTATCAAAACTCAGAAGCTCGTGGCCCTTAAAGAGTTACATATGAATTATAAAC
AAATCTTAATGTCATCAGCAAAAGTTGTGATAGTTTATGGATACAAAGACTCTCCCATAATCTATGCCCTCATTTC
ATGGAAATCTCATGGCATTTTTAGAATCTGGGTTAGTGTGTCACAATTTGATATGATCACGATTCGAGGAGATTTC
TTGCTCTACTCCTCCACTGGGACTTTCATTTTCTCACACCAGAAACCTGAAATATCCGGCTTTGAACAATTTATAC
AAACAGTACACCCTTCAAATTACAGTAGTGAATTTTCCTTTGCCAAACTCTGGTGGACATATTTTAGATGTTCTTT
GCCACCTTCTGATTGTAAGAAACTAAAGAATTGTCCCACCAAAACTGTGTTTAAATGGTTATTCATGACACCACTT
GGAATGGCCATGAGTGATACATGCTATAACTTATAC
>ms71_A5 .
AGGAGTAATCATTACAAATGATGACCACGGAATACAATTCCTTTCTGAATTGAAAGGAGATATGCAAAAAAGCATT
GTTTGCTTATCAGTTGCTATTATTATCCAAACTGAGGTGTACATGGCCCTTAAAGAGTTACATATGAACTATAAAA
AAATCTTAATGTCATCAGCAAAAGTTGTGATAGTTTATGGAGACAAAGACTCTCCCATAAAGTATGTTCTCACAGC
ATGGAAATCTCAAGGCATTTTTAGAATCTGGGTTAGTGCATCACAATTTGATATGATCACGATACTAGGAGATTTC
TTGCTATACTCCTACACTGGGACTTTTATTTTTTCACACCAGCAACCTGAAATACCTGGTTTTGAAAAATTTATCC
AAACAGTACACCCTTCAAACTACAGTAGTGAATTTTCCTTTGCCAAACTGTGGTGGACATATTTTAGATGTTCTTT

GCCACCATCTAATTGTAAGAAGCTGAAGAATTGTCCAACCAAAAGTGTATTTGCATGGTTATTCAGGACACCACTT
GGAATGGCCATGAGTGATACATGCTATAACTCATAC
>ms72_A5 .
AGGAGCGATGATTACAAATGATGACCATGGAATTCAATTCCTTTCTAAATTGAGAGGAGAAATGCAAAAACACATG
GTCTGTTTATCATTTGTGTTTGCTATCCTAACTGAGCATAGCATGGTCCGTAAAGAGTTTCATAAGAATTTTAATT
TAATTGTAAGGTCATCAGCAAAAGTTGTGATAGTTTATGGAGACTATGCCTCTCCCATAGATCTTGTCCTCCATTG
GTTTAAATCAAAAGGCCTTTTTAGAATCTGGGTTAGTGTGTCACAATTTGATATAATCACAAATCTAGGAGATTTC
ATGCTATATTCCTCCTCTGGGACATTCATTTTTTCACACCAGAAACCCGAAATATCTGGTTTCCAAAAATTTATCA
AAACAGTATACCCTTCAAATTACAGTAGTGAATTTTCTTTTGCCAAACTATGGTGGACATATTTTAGATGTTCTTT
GCCACCATCTAATTGTAAAACGCTGAAGAATTGTCCCACCAAAACTGTATTTAAATGGTTATTCAGGACACCACTT
GGAATGGCCATGAGTGATACATGCTATAACTCATAC
>ms73_A5 .
AGGAGTGATCATTACAAATGATAACCATGGAATTCAATTCCTTTCTGAATTGAGAGGAGAAACGCAAAAACAAATT
GTATGTTTATCAGTTGTGATTATTATCCAAACTGATGATATCACGGCATATAAAGAGTATCATATGAATTATAATA
AAATTGTAATGTCTTCAGCAAAAGTTGTGATAGTTTATGGAGACCACAACTCGCCCATAAAGTTTGTCCTATTTTT
GTGGAAATCTCAAGGCATTTTTAGAATTTGGGTTAGTGTGTCACAATTTGACATGATCACACCTCTAGGAGATTTC
ATGCTATACTCCGCCTCTGGGACATTAATTTTTTCACACCAGCAATCTGAACTGTCTGGTTTTGAAAAATTTATCA
AAACAGTAAAACCTTCAAACTACAGTAGTGAATTTTCTTTTGCTAAACTATGGTGGACATATTTTAGATGTTCTTC
TTTGCCACCATCTAATTGTAAGAAACTGAAGAATTGTCCCACCAAAACTGTATTTAGATGGTTATTCATGACACCA
CTTGGAATGGCCATGAGTGATACCTGTTATAACTTATAC
>ms74_A5 .
AGGAGTGATCGTTACAAATGATGACCATGGAATTCAATTCCTTTCTGAATTTAGAGGAGAAATGCAAAACAAGATT
GTCTGTTTATCATTTGCTATTATTATCAAAACTGGAAAGTTCCTGGCTGTTACAGAGTTACATATGAATTATAAGC
AAATCTTAATGTCATCAGCCAAAGTTGTGATAGTTTATGGATACAGAGACTCGCCCATAATCTATGCCTTCATTGT
GTGGAAATCTCAAGACCTTTTCAGAATCTGGGTTAGCGTATCACAACTTGATATGATCACATTTCTAGGAGATTTC
TTGCTATATGCGTCCACTGGGACTTTCATTTTTTCACACCAGAAACCCGAAATATCTGGCTTTGAACAATTTATCC
AAAGAATACACCCTTCAAACTACAGTAGTGAATTTTCCTTAGCTAAACAATGGTGGACATATTTTAGATGTTCTTT
GCCACCATCTAATTGTAAGAAGCTCAAGAATTGTTCCACCAAAACTGTATTTAAATGGTTATTCATGACACCACTT
GGATTAGCCATGAGTGATACATGCTATAACATATAC
>ms75_A5 .
AGGAGCAATAGTTACAAATGATGACCATGGAATTCAATTCCTTTCTGAATTGAGAGGAGAAATGCAAAAACACATT
GTCTGTTTATCAGTTGCGATTATTATCCAAACTGAGAAGTTCATGGCCCTTAAAGAGTTTCGTATGAATTATAACA
AAATTGCAATGTCATCAGCAACAGTTGTAATAGTTTATGGAGACAAAGACTCTCCCATACAGTTTACCCTCATTAT
GTGGAAATCTGAAGGCATTTGGAGAATCTGGGTTAGTGTGTCACAATTTGATATGATCACAGTTATAGGAGATTTT
TTACTATACTCCTCCACGGGGAGTTTCATTTTTTCACACCAGCAATCTGAAATATCTGGTTTTGAAAAATTCATCC
AAACAGTACACCCTTCAAACTACAGTAGTGAATTTTCCCTTGCCAAACTGTGGTGGACATATTTTACATGTTCTTT
GCCACCGTCTAATTGTAAGAAGCTAAAGAATTGTCCAATCAAAACTGTATTTAAATGGTTATTCATGACACCTATT
GGAATGTCCATGAGTGATATATCCTATAACTTATAC
>ms76_A5 .
GGGACTGATGATTTCAGATGACGGTCTAGGACTTCAATTTGCCTCTGAATTGAGAGAAGAAATGCAAAGATATGAC
ATCTGTTTAGCCTTTGTGACTATTATCACATATAACACAAAATTATTCTTGACAATGACTGACACGTATTACAATC
AGATCATGATGTCTTTAGCAAAAGCTGTTATCATTTTTGGAGACAAAGATTCTCTTCTACAAGTGATTTTTAGACT
GTGGCAGTTTTTAGACATTCGAAGAATCTGGGTTACTACCTCACAGTGGGATATCATTACAAGTAATGGAGAATTC
CTCTTCAACTCCTTTCATGGTACCCTCAGCTTTTCACACCATTATTCTGAGATAGCTGGTTTTAAAGAATTTATAC
AGACAGCGCACCCTTTAAACTACAGTAACACTATTTCTCTTGCTAAATTATGGTGGTTGAATTTTAATTGTTTTTT
GTCATCATCTAATTGTAAGAGTCTCAAGACATGTTCAAGAAAAATGTTGCTGAAATGGTTATCCAGAAACCACTTT
GAAATGTCCATGAGTGACACAAGTTATAACCTATAC
>ms77_A5 .
AGGAGTCATCATTACAGATGATGACCATGGAATTCAGTTCCTTTCTGAACTGAGAACAGGAATGGAAAGAAACACT
GTCTGCTTAGCATTTGTCACTTCTATCACTTATGATAAGATGTTATACCTTAAAATGACTCATAAATATTATCACC
AAATCATAATGTCATCAGCAAAAGTTGTCGTCGTCTATGGGGATAAAGAATCTCCTCTACAACTGAACTTCATGCT
TTGGAAATCTATAAACATTCAGAGACTCTGGGTCAGTGTGTCACAATTTGATATGATCACAATGATGGGAGATTTC
ATGCTTAATTCCATTCATGGGACTCTTATTTTTTCACATCAACAGTCTGAGATGTCTGGTTTTAAACACTTTATTC
AGAGAGTGAACCCTTCTAACTACAGTAATGATATTTCTTTGGCTAAACTGTGGTGGACTTACTTTAAGTGTTCATT
GCCACCACCTGATTGTAAAACACTGAAGAATTGTCCAACCAAAACCCTATTTCAATGGTTTTTTGAGCCACCTCTT
GGAATGTCTATGAGTGAAACATGTTACAACTTATAC
>ms78_A5 .
AGGAGTCATCATTACAGATGATGACCATGGAATTCAGTTCCTTTCTGAACTGAGAACAGGAATGGAAAGTAACACT
GTCTGCTTAGCATTTGTAACTATTATCACATACAATAGGATGTTATACCTTAAAATGTATCATAAATATTATCACC
AAATCACAATGTCATCAGCAAAAGTTGTCATTGTCTATGGAGATAAAGAATCTCCTCTACAATTTAACTTCATACT
ATGGAAATCTGAAAACATTCAGAGACTTTGGGTCAGTGTGTCACAATTTGATATGATCACAGTGATAAGGGATTTC
ATGCTTAACTCCTTGCATGGAACTCTTATTTTTTCACATCAGCAGTCTGAGATATCTGGTTTTAAACAATTTATGC

AGACAGTGCACCCATCTAACTACAGTAATGACATTTCTTTGGCTAAAATGTGGTGGACATACTTTAAGTGTTCTTT
GAAACCACCTGATTGTAAAACACTGAAGAATTGTCCAACCAAAACCCTATTTAAATGGTTTTTTGTACCACCTCTT
GGAATGTCTATGAGTGAAACATGTTACAACTTATAC
>ms79_A5 .
AGGAGTCATCATTACAGATGATGACCATGGAATTCAGTTCCTTTCTGAATTGAGAGCAGGAATGGAAAGTAACACT
GTCTGCTTAGCATTTGTAACTATTATCACATACAATAGGAAGTTATACCTTAAAATGTATCATAAATATTATCACC
AAATCACAATGTCATCAGCAAAAGTTGTCATTGTCTATGGAGATAAAGAATCTCCTCTACAGTTTAACTTCATACT
ATGGAAATCTGAAAACATTCAGAGACTTTGGGTCAGTGTGTCACAATTTGATATAATCACAATGATAGGTGAATTC
ATGCTTAACTCTGTGCATGGAACTCTCATTTTTTCACATCAGCAGTCTGAGATGTCTGGTTTTAAACAATTCATGC
AGACAGTGCACCCATCTAACTACAGTAATGACATTTCATTGGCTAAACTGTGGTGGACTTACTTTAAGTGTTCTTT
GCCACCACCTGATTGTAAAACACTGAAGAATTGTCCAACCAAAACCCTACTTAAAAGGTTTTTTGTGCCACCTCTT
GGAATGTCTATGAGTGAAACATGTTACAACTTATAC
>ms28_A4 .
CGGCCTTATCATCCCTGATGATGAAGATGGCTATCAGTTTCTGTCACAGTTGAAAAAAGAGAGCAAAGGCAAGGAA
ATTTGCTTTGCCTTTGTGAATATGATAGGAATCTCTGATATTTTGACCTATGCCACAACTGAAATGAACTACAACC
AAATTATGATGTCATCCACAAATGTTATTATTATTTATGGGGAAACAAACAATTTTATTGAATTGAGCTTCAGAAT
ATGGGAATCTACAGTTATACAGAGAATATGGGTCACCACAATACAACTGAATTTCCCTACCAGTAAGAAAGACTTA
AATCATGGCACATTCTATGGGACTTTTACTTTTCTACCCCACCATGCTGAGATTTCTGGCTATAAAAAGTTTGTAC
AGACATTGTTCCATCTCAAAAGCACAGATTTAAACCTAGAAATGCAAGAGTGGAAGTACTTTAACTGTGAAGATTC
AGCATGTAACTGTAAAATACTGATGAATTTTTCATCAAATGCTTCATTGGATTGGCTAATAGAACAGAAGTTTGAC
ATAACCTTTAGTGATGGTAGCCAAAACATATAT
>ms29_A4 .
TGGCCTTGTCATTCCAGATGACGATCAAGGAAACCAATTTCTTTTAGAGTTGAAGAAACAGAGTGAAAACAAGGAA
ATTTGCTTTGCCTTTGTGAAAATGATCTCTGTTGATTATGTTTATTTAGAACCAGAAATTGAAATGTACTACAAAC
AAATTGTGATGTCATCCTCAAATGTTATTATCATTTATGAGGAAAAAGACAATTTCTTTGATTTGATCTTCAGAAT
GTGGGAACCTCCAGTTTTACAGAGAATATGGATCACCACAAAACAATGGAATTGCCCTACCAGTAAGAGAGACATA
ACTCATGGCACATTCTATGGATCACTTACTTTTCTACCCCACCATGGTGAGATTTCTGGCTTTAAAAATTTTGTAC
AGACATGGTTCAATCTCAGTAACAAAGATTTATATCTAGTAATGCCAGAGTGGAAATATTTTAAATATGAAGGCTC
AGCATCTAACTGTAAAATACTGAAGAACAATTCATCTGATGCCTCATTTGATTGGCTAATGGAACAGAAGTTTGAC
ATGACCTTTAGTGAGAATAGTCATAACATATAC
>ms30_A4 .
TGGCCTTGTCATCCCAGATGACGATCAAGGAAACCAATTTCTTTTAGAGTTGAAGAAACAGAGTGAAAACAAGGAA
ATTTGCTTTGCCTTTGTGAAAATGATCTCTGTTGATGATACTTCATTTCCACATAAAACTGAAATGGACTACAACC
AAATTGTGATGTCATCCACAAATGTTATTATCATTTATGGAGAAACACGCAATTTCATTTATTTGATCTTCAGAAT
GTGGGAACCTCCCATTTTACAGAGAATATGGATCACCACAAAACAATTGAATTTCCCTACCAGGAAGACAGACATA
AGTCATGGCACATTCTATGGATCACTTACTTTTCTACCCCACCATGGTGAGATTTCTGGCTTTAAAAAGTTTGTAC
AGACATGGTTCCATGTCAGAAACACAGATTTATATTTAGTAATGCCAGAGTGGAACTATTTTAACTATGTAAGCTC
AGCATCCAATTGTAAAATACTGAAGAACAATTCATCTGATGCCTCATTTGATTGGCTAATGGAACAGAAGTTTGAC
ATGACCTTTAGTGAGAATAGTCATAACATATAC
>ms31_A4 .
TGGCCTTGTCATCCCAGATGACGATCAAGGAAACCAATTTCTTTTAGAGTTGAAAAAACAGAGTGAAAACAAGGAA
ATTTGCTTTGCCTTTGTGAAAATGATCTCTGTTGATGACATTTCATTTGAACACAAAACTGAAATGTACTACAAAC
AAATTGTGATGTCATCCTCAAATGTTATCATCATTTATGAGGAAACAATCAATTTCATTGATTTGATCTTCAGAAT
GTGGGAACCTCCAGTTTTACAGAGAATATGGATCACCACAAAACAATGGAATTTCCCTACCAGTAAGAGAGACATA
ACTCATGGCACATTCTATGGATCACTTACTTTTCTACCCCACCATGGTGGGATTTCTGGCTTTAAAGATTTTGTAC
AGACATGGTTCCATCTCAGAAGCAAAGATTTATATCTAGTAATGCCAGAGTGGAAATACTTTAAATATGAATCCTC
AGCATCTAAGTGTAAAATACTGAAGAACAATTCATCTGATGCCTCATTTGATTGGCTAATGGAACAGAAGTTTGAC
ATGGCCTTTAGTGAGAGTAGTCATAACATATAC
>ms32_A4 .
TGGCCTTGTCATCCCAGATGACGATCAAGGAAACCAATTTCTTTTAGAGTTGAAGAAACAGAGTGAAAACAAAGAA
ATTTGCTTTGCCTTTGTGAAAATGATATCTGTTGATGAAGTTTCATTTCCACAAAAAACTGAAATATACTACAAAC
AAATTGTGAAGTCATTAACAAATGTTATTATCATTTATGGAGAAACATATAATTTCATTGATTTGATCTTCAGAAT
GTGGGAACCTCCCATTTTACAGAGAATATGGATCACCACAAAACAATTGAATTTCCCTACCAGTAAGACAGACATA
AGTCATGACACATTCTATGGATCACTTACTTTTCTACCCCACCATGGTGAGATTTCTGGCTTTAAAAATTTTGTAC
AGACATGGTTCCATCTCAGAAACACAGATTTATATCTAGTAATGCCAGAGTGGAAATATATTAACTCTGAAGACTC
AGCATCTAATTGTAAAATACTGAAGAACAGTTCATCTGATGCCTCATTTGATTGGCTAATGGAACAGAAGCTTGAC
ATGGCCTTTAGTGATAATAGTCATAACATATAT
>ms33_A4 .
TGGCCTTGTCATCCCAGATGACGATCAAGGAAACCAATTTCTTTTAGAGTTGAAAAAACAGAGGGAAAACAAGGAA
ATTTGCTTTGCCTTTGTGAAAATGATCTCTGTTGATGACATTTCATTTGAACACAAAACTGAAATGTACTACAAAC
AAATTGTGATGTCATCCTCAAATGTTATCATCATTTATGAGGAAACAATCGATTTCATTGATTTGATCTTCAGAAT
GTGGGAACCTCCAGTTTTACAGAGAATATGGATCACCACAAAACAATGGAATTTCCCTACCAGTAAGAGAGATATA

ACTCATGGCACATTCTATGGATCACTTACTTTTCTACCCCACCATGGTGGGATTTCTGGCTTTAAAGATTTTGTAC
AGACATGGTTCCATCTCAGAAGCAAAGATTTATATCTAGTAATGCCAGAGTGGAAATACTTTAAATATGAATCCTC
AGCATCTAAGTGTAAAATACTGAAGAACAATTCATCTGATGCCTCATTTGATTGGCTAATGGAACAGAAGTTTGAC
ATGGCCTTTAGTGAGAGTAGTCATAACATATAT
>ms34_A4 .
TGGCCTTGTCATTCCAGATGATGACCAAGGAAACCAATTTCTTTTAGAGTTGAAGAAACAGAGTGAAAACAAGGAA
ATTTGCTTTGCCTTTGTGAAAATGATCTCTGTTGATGATGTTTCATTTCCACAAAATACTGAAATGTACTACAACC
AAATTGTGATGTCATCCACAAATGTTATTATCATTTATGGAGAAACATACAATTTCATTGATTTGATCTTCAGAAT
GTGGGAACCTCCCATTTTACAGAGAATATGGATCACCACAAAACAATTGAATTTCCCTACCAGGAAGACAGACATA
AGTCATGGCACATTCTATGGATCACTTACTTTTCTACCCCACCATGGCGTGATTTCTGGTTTTAAAAATTTTGTAC
AGACATGGTTCCATCTCAGAAACACAGATTTATATCTAGTAATGCAAGACTGGAAATACTTTAACTATGAAGACTC
AGCATCTACCTGTAAAATACTGAAGAACAATTCATCTAATGCCTCATTTGATTGGCTAATGGAACAGAAGTTTGAC
ATGACCTTTAGTGAGAATAGTCATAACATATAC
>ms35_A4 .
TGGCCTTGTCATCCCAGATGATGATCAAGGAAACCAATTTTTTTTAGAGTTGAAAAAACAGTGTGAAAACAAGGAA
ATTTGCTTTGCCTTTGTGAAAATGATCTCTGTTGATGACATTTCATTAGAAGAAAAAACTGAAATGTACTACAAAC
AAATTGTGATGTCATCCTCAAATGTTATCATCATTTATGAGGAAACAATCAATTTCATTGATTTGATCTTCAGAAT
GTGGGAACCTCCAGTTTTACAGAGAATATGGATCACCACAAAACAATGGAATTTCCCTACCAGTAAGAGAGACATA
ACGCATGACACATTCTATGGATCACTTACTTTTCTACCCCACCATGGTGGGATTTCTGGCTTTAAAAATTTTGTGC
AGACTTGGTTCCATCTCAGAAGCAAAGATTTATATCTAGTAATGCCAGAGTGGAAATACTTTAAATATGAATCCTC
AGCATCTAAGTGTAAAATACTGAAGAGCAAATCATCCAATGCCTCATTTGATTGGCTAATGGAACAGAAGTTTGAC
ATGGCCTTTAGTGAGAGTAGTCATAACATATAC
>ms36_A4 .
TGGCCTTGTCATCCCAGATGATGATCAAGGAAACCAATTTCTTTTAGAGTTGAAGAAACAGAGTGAAAACAAGGAA
ATTTGCTTTGCCTTTGTGAAAATGATCTCTGTTGATGATGTTTCATTTCCACAAAATACTGAAATGTACTACAACC
AAATTGTGATGTCATCCACAAATGTTATTATCATTTATGGAGAAACATACAATTTCATTGATTTGATCTTCAGAAT
GTGGGAACCTCCCATTTTACAGAGAATATGGATCACCACAAAACAATTGAATTTCCCTACCAGGAAAAAAGACATA
AGTCATGGCACATTCTATGGATCACTTACTTTTCTACCCCACCATGGTGAGATTTCTGGCTTTAAAAATTTTGTAC
AGACATGGTTCCATTTCAGAAACACAGATTTATATCTAGTAATGCCAGAGTGGAAATATTTTAACTATGAAGACTC
AGCATCTAACTGTAAAATACTGAAGAACAATTCATCTGATGCCTCATTTGATTGGCTAATGGAACAGAAGTTTGAC
ATGACCTTTAGTGAGAGTAGTCATAACATATAC
>ms37_A4 .
TGGCCTTGTCATTCCAGATGATGACCAAGGAAACCAATTTCTTTTAGAGTTGAAGAAACAGAGTGAAAACAAGGAA
ATTTGCTTTGCCTTTGTGAAAATGATCTCTGTTGATGATGTTTCATTTCCACAAAATACTGAAATGTACTACAACC
AAATTGTGATGTCATCCACAAATGTTATTATCATTTATGGAGAAACATACAATTTCATTGATTTGATCTTCAGAAT
GTGGGAACCTCCCATTTTACAGAGAATATGGATCACCACAAAACAATTGAATTTCCCTACCAGGAAAAAAGACATA
AGTCATGGCACATTCTATGGATCACTTACTTTTCTACCCCACCATGGTGTGATTTCTGGTTTTAAAAATTTTGTAC
AGACATGGTTCCATCTCAGAAACACAGATTTATATCTAGTAATGCAAGAGTGGAAATACTTTAACTATGAAGACTC
AGCATCTACCTGTAAAATACTGAAGAACAATTCATCTAATGCCTCATTTGATTGGCTAATGGAACAGAAGTTTGAC
ATGACCTTTAGTGAGAATAGTCATAACATATAC
>ms39_A4 .
TGGCCTTCTCATCCCAGATGATGATCAAGGAAACCAATTTCTTTTAGAGTTGAAAAAACAGAGTGGAAACAAGGAA
ATTTGCTTTGCCTTTGTGAAAATGATCTCTGTTGATGACATTTTATTAGAACAAAAAACTGAAATGTACTACCAAC
AAATTGTGATGTCATCCTCAAATGTTATCATCATTTATGAGGAAACAATCAATTTCATTGATTTGATCTTCAGAAT
GTGGGAACCTCCAGTTTTACGGAGAATATGGATCACCACAAAGCAATGGAATTTCCCTACCAGTAAGAGAGACATA
ACTCATGGCACATTCTATGGATCACTTACTTTCCTACCCCACCATGGTGGGATTTCTGGCTTTAAAAATTTTGTAC
AGACTTGGTTCCATCTCAGAAGCAAAGATTTATATCTAGTAATGCCAGAGTGGAAATACTTTAAATATGAATCCTT
AGCATCTAACTGTAAAATGCTGAAGAGCAATTCATCCAATGCCTCATTTGATTGGCTAATGGAACAGAAATTTGAC
ATGGCCTTTAGTGAGAGTAGTCATAACATATAC
>ms45_A4 .
TGGCCTTGTCATCCCAGATGATGACCAAGGAAACCAATTTCTTTTAGAGTTGAAGAAACAGAGTGAAAACAAGGAA
ATTTGCTTTGCCTTTGTGAAGATGATCTCTGTTGATGATGTTTCATTTCCACAAAATATTGAAATGTACTACAACC
AAATTGTGATGTCATCCACAAATGTTATTATCATTTATGGAGAAACATACAATTTCATTGATTTGATCTTCAGAAT
GTGGGAACCTCCCATTTTACAGAGAATATGGATCACCACAAAACAATTGAATTTCCCTACCAGGAAGACAGACATA
AGTCATGGCACATTCTATGGATCACTTACTTTTCTACCCCACCATGGTGTGATTTCTGGTTTTAAAAATTTTGTAC
AGACATGGTTCCATCTCAGAAACACAGATTTATATCTAGTAATGCAAGAGTGGAAATACTTTAACTATGAAGACTC
AGCATCTACCTGTAAAATAAAGAAGAACAATTCATCTAATGCCTCATTTGATTGGCTAATGGAACAGAAATTTGAC
ATGACCTTTAGTGAGAATAGTCATAACATATAC
>ms44_A4 .
TGGCCTTGTCATCCCAGATGATGATCAAGGAAACCAATTTCTTTTAGAGTTGAAAAAACAGAGGGAAAACAAGGAA
ATTTGCTTTGCCTTTGTGAAAATGATCTCTGTTGATGACATTTCATTTGAACACAAAACTGAAATGTACTACAAAC
AAATTGTGATGTCATCCTCAAATGTTATCATCATTTATGAGGAAACAATCGATTTCATTGATTTGATCTTCAGAAT

GTGGGAACCTCCAGTTTTACAGAGAATATGGATCACCACAAAACAATGGAATTTCCCTACCAGTAAGAGAGACATA
ACTCATGGCACTTTCTATGGATCACTTACTTTTCTACCCCACCATGGTGGGATTTCTGGCTTTAAAGATTTTGTAC
AGACATGGTTCCATCTCAAAAGCAAAGATTTATATCTAGTAATGCCAGAGTGGAAATACTTTAAATATGAATCCTC
AGCATCTAAGTGTAAAATACTGAAGAACAATTCATCTGATGCCTCATTTGATTGGCTAATGGAACAGAAGTTTGAC
ATGGCCTTTAGTGAGAGTAGTCATAACATATAC
>ms43_A4 .
TGGCCTTCTCATCCCAGATGATGATCAAGGAAACCAATTTCTTTTAGAGTTGAAAAAACAGAGTGGAAACAAGAAA
ATTTGCTTTGCCTTTGTGAAAATGATCTCTGTTGATGACATTTTATTAGAACAAAAAACTGAAATGTACTACCAAC
AAATTGTGATGTCATCCTCAAATGTTATCATCATTTATGAGGAAACAATCAATTTCATTGATTTGATCTTCAGAAT
GTGGGAACCTCCAGTTTTACGGAGAATATGGATCACCACAAAGCAATGGAATTTCCCTACCAGTAAGAGAGACATA
ACTCATGGCACATTCTATGGATCACTTACTTTCCTACCCCACCGTGGTGGGATTTCTGGCTTTAAAAATTTTGTAC
AGACTTGGTTCCATCTCAGAAGCAAAGATTTATATCTAGTAATGCCAGAGTGGAAATACTTTAAATATGAATCCTT
AGCATCTAACTGTAAAATGCTGAAGAGCAATTCATCCAATGCCTCATTTGATTGGCTAATGGAACAGAAATTTGAC
ATGGCCTTTAGTGAGAGTAGTCATAACATATAC
>ms42_A4 .
TGGCCTTGTCATCCCAGATGATGATCAAGGAAACCAATTTCTTTTAGAGTTGAAGAAACAGAGTGAAAACAAAGAA
ATTTGCTTTGCCTTTGTGAAAATGATCTCTGTTGATGAAGTTTCATTTCCACAAAAAACTGAAATAAACTACAAAC
AAATTGTGAAGTCACTAACAAATGTTATTATCATTTATGGAGAAACATATAATTTCATTGATTTGATCTTCAGAAT
GTGGGAACCTCCCATTTTACAGAGAATATGGATCACCACAAAACAATTGAATTTCCCTACCAGTAAGACAGACATA
AGTCATGACACATTCTATGGATCACTTACTTTTCTACCCCACCATGGTGAGATTTCTGGCTTTAAAAATTTTGTAC
AGACATGGTTCCATCTCAGAAACACAGATTTATGTCTAGTAATGCCAGAGTGGAAATATATTAACTCTGAAGACTC
AGCATCTAATTGTAAAATACTTAAGAACAGTTCATCTGATGCCTCATTTGATTGGCTAATGGAAGAGAAGCTTGAC
ATGGCCTTTAGTGAGAATAGTCATAACATATAT
>ms41_A4 .
TGGCCTTGTCATCCCAGATGATGATCAAGGAAACCAATTTCTTTTAGAGTTGAAGAAACAGAGTGAAAACAAGGAA
ATTTGCTTTGCCTTTGTGAAAATGATCTCTGTTGATGATGTTTCATTTCCACAAAATACTGAAATGTACTACAACC
AAATTGTGATGTCATCCACAAATGTTATTATCATTTATGGAGAAACATACAATTTCATTGATTTGATCTTCAGAAT
GTGGGAACCTCCCATTTTACAGAGAATATGGATCACCACAAAACAATTGAATTTCCCTACCAGGAAAAAAGACATA
AGTCATGGCACATTCTATGGATCACTTACTTTTCTACCCCACCATGGTGAGATTTCTGGCTTTAAAAATTTTGTAC
AGACATGGTTCCATCTCAGAAACACAGATTTATATCTACTAATGCAAGAGTGGAAATATTTTAACTATGTAAGCTC
AGCATCCAATTGTAAAATACTGAAGAACAATTCATCTGATGCCTCATTTAATTGGCTAATGGAACAGAAGTTTGAC
ATGACCTTTAGTGAGAATAGTCATAACATATAC
>ms49_A4 .
TGGCCTTGTCATCCCAGATGACGATCAAGGAAACCAATTTCTTTTAGAGTTGAAGAAACAGAGTGAAAACAAAGAA
ATTTGCTTTGCCTTTGTGAAAATGATCTCTGTTGATGAAGTTTCATTTCCACAAAAAACTGAAATATACTACAAAC
AAATTGTGAAGTCATTAACAAATGTTATTATCATTTATGGAGAAACATATAATTTCATTGATTTGATCTTCAGAAT
GTGGGAACCTCCCATTTTACAGAGAATATGGATCACCACAAAACAATTGAATTTCCCTACCAGTAAGACAGACATA
AGTCATGACACATTCTATGGATCACTTACTTTTCTACCCCACCATGGTGAGATTTCTGGCTTTAAAAATTTTGTAC
AGACATGGTTCCATCTCAGAAACACAGATTTATATCTAGTAATGCCAGAGTGGAAATATATTAACTCTGAAGACTC
AGCATCTAATTGTAAAATACTGAAGAACAGTTCATCTGATGCCTCATTTGATTGGCTAATGGAAGAGAAGCTTGAC
ATGGCCTTTAGTGATAATAGTCATAACATATAT
>ms50_A4 .
TGGCCTTCTCATCCCAGATGATGATCAAGGAAACCAATTTCTTTTAGAGTTGAAAAAACAGAGTGGAAACAAGGAA
ATTTGCTTTGCCTTTGTGAAAATGATCTCTGTTGATGACATTTTATTAGAACAAAAAACTGAAATGTACTACCAAC
AAATTGTGATGTCATCCTCAAATGTTATCATCATTTATGAGGAAACAATCAATTTCATTGATTTGATCTTCAGAAT
GTGGGAACCTCCAGTTTTACAGAGAATATGGATCACCACAAAGCAATGGAATTTCCCTACCAGTAAGAGAGACATA
ACTCATGGCACATTCTATGGATCACTTACTTTCCTACCCCACCATGGTGGGATTTCTGGCTTTAAAAATTTTGTAC
AGACTTGGTTCCATCTCAGAAGCAAAGATTTATATCTAGTAATGCCAGAGTGGAAATACTTTAAATATGAATCCTC
AGCATCTAACTGTAAAATGCTGAAGAGCAATTCATCCAATGCCTCATTTGATTGGCTAATGGAACAGAAATTTGAC
ATGGCCTTTAGTGAGAGTAGTCATAACATATAC
>ms51_A4 .
TGGCCTTGTCATTCCAGATGACGATCAAGGAAACCAATTTCTTTTAGAGTTGAAGAAACAGAGTGAAAACAAGGAA
ATTTGCTTTGCGTTTGTGAAAATGATCTCTGTTGATGACGTTTCACTTCCACATACAACTGAAATGTACTACAGCC
AAATTGTGATGTCATTAACAAATATTATTATCATTTATGGAGAAACATACAATTTCATTGACTTGATCTTCAGAAT
GTTGGACCCTCCCACTTTACAGAGAATATGGATCACCACAATAGAATGGAATTTCCCTACCAGTAATACAGACATA
AATCATGGCACATTCTATGGATCACTTACTTTTCTACCCCACCATGGTGAGATTTCTGGCTTTAAAAATTTTGTAC
AGACATGGTTCCATGTCAGAAACACAGATTTATATCTACTAATGCAAGAGTGGAAATATTTTAACTATGAAGACTC
AGCATCTAACTGTAAAATACTGAAGAACAATTCATCTGATGCCTCATTTGATTGGCTAATGGAACAGAAGTTTGAC
ATGACCTTTAGTGAGAGTAGTCATAACATATAC
>ms52_A4 .
CGGCCTTATCATCTCAGATGATGATGAAGGTTATCAGTTTCTGTCACAGTTGAAAAAAGAGAGCAAAGGCAAGGAA
ATTTGCTTTGCCTTTGTGAAAATGATAGGAATCTCTCACATTTTCACATATGCCTCAACTGAAATGGACTACAACC

```
AAATTATGATGTCATCCACAAATGTTATTATTATTTATGGGGAAACAAGCAATTTTATTGAATTGAACTTCAGAAT
ATGGGAATCTACAGTTATACAGAGAATATGGGTCACCACAGTACAACTGAATTTCCCTACCAGTAAGAAAGACTTA
ACTCATGGCCCATTCTATGGGACTTTTACTTTTCTACCCCACCATGGTGAGATTTCTGGCTATAAAAATTTCCTAC
AAACATTGTTCCATCTCAAAAGCACAGATGCATTTCTAGTAATGCCAGAGTGGAAATATTTTAACTGTGAAGACTC
AGCATGTAACTGTAAAATACTGATGAATTATTCAGCAAATTCTTCATTGGATTGGCTAATGGGACAGAAGTTTGAG
ATAACTTTTAGTGATGATAGCCAAAACATATAC
>ms111_A3 .
AGGGTTGGTCATCTCAGACAGTGATCAAGGTATCCAATTTCTCTCCTATTTGAGAAGAGAGATGGAAAAATACACA
GTGTGCTTTGCCTTTGTCAACATGATTCCAGTCAACATGAATTTATACATGTCAAGAGCTGAAGTGTATTACAACC
AAATCATGACATCATCCACAAATGTTGTCATCATTTATGGTGACACCGACAGTACATTAGCTGTGAGCTTTAGAAT
GTGGGAATCCCTAGGTATACAGAGACTATGGATCACCACCTCACAGTGGGATGTCAGTCCTAGAATGAAAGACTTT
ACATTTGGTAACAAATATGGGACTTTTGATTTTCAACAACACAATAGTGAGATTTCTGGTTTCAAAAATTTTGTGC
AGACATTGAACTCTGTCAAATACCCAGATGACTATCTGGTAGAGCTGGAATGGATGCACTTTAACTGTGAGGTCTC
AGCATCTAAATGTAAGACACTGAAGAACTGCTCATCCAATCACTCATTGGAATGGTTAATGGTACATACTTTTGAC
ATGGCTTTTATTGAAAAGAGTTATTACATATAC
>ms112_A3 .
AGGGTTGGTCATCTCAGACAGTGATCAAGGTATCCAATTTCTCTCCTATTTGAGAAGAGAGATGGAAAAATATACA
GTGTGCTTTGCCTTTGTCAACATGATTCCAGTCAACATGAATTTATATATGTCAAGAGCTGAAGTGTATTACAACC
AAATCATGACATCATCCACAAATGTTGTCATCATTTATGGTGACACCGACAGTACATTAGCTGTGAGCTTTAGAAT
GTGGGAATCCCTAGGTATACAGAGACTATGGATCACCACCTCACAGTGGGATGTCAGTCCTAGTATGAAAGACTTT
ACATTTGGTAACAAATATGGGACTTTTGATTTTCAACAACACAATAGTGAGATTTCTGGTTTTAAAAATTTTGTGC
AGACATTGAACTCAGTCAAATGCCCAGATGAATATCTGGTAGAGCTGGAATGGATGCACTTTAACTGTGAGGTCTC
AGCATCTAAATGTAAGCCACTGAAGAACTGCTCATCCAGTTACTCATTTGATTGGTTAATGGAACATACTTTTGAC
ATGGCGTTTATTGAAAAGAGTTATTACATATAC
>ms113_A3 .
AGGGTTGGCCATCTCAGACAATGATAAAGGTATCCAATTTCTTTCCTATTTGAGAAGAGAGATGGAAAAAAATACA
GTCTGCTTTGCTTTTGTCAGCATGATTCCAGTCAATATGCATTTATACATGACAAGAACTGAAGTGTATTACAATC
AAATCATGACATCATCTACAAATGTTGTTATCATATATGGTGACACAGACAGTACTTTAGCTGTGAGTTTTAGAAT
GTGGGAATCTCTAGATATAAAGAGAATATGGGTCACCACCTCACAGTGGGCTATTACTACTGGTAAGAAAGACTTC
ACATTTAATAACTTGTATGGGACATTTGCTTTTGGACACCACCATGGTGAGATTTCTGGTTTTAAAAACTTTGTCC
AGACATTGAACCTGTTAAAATACTCAGATGAATATCTGGTAAAGCTGGAATGGATGTACTTTAACTGTGAAGTCTC
AGCACCTAAATGTAAGACACTGAAGAACTGCTCTTCCAATCACCCATTGGAATGGTTAATGGTACGTACTTTTGAC
ATGGCCTTTACTGAAGGGAGTTATGCCATATAC
>ms114_A3 .
AGGGCTGGCCATCTCAGACAATGATCAAGGTATCCAATTTCTCTCCTATTTGAGAAGAGTCATGGAAAAAAATGCA
GTCTGCTTTGCCTTTGTCAACATGATTCCAATGAGCATGAATTTATACATGTCAAGAGCTGAAGTGTATTACAACC
AAATCATGACATCATCCACAAATGTTGTCATCATTTATGGTGACACAGACAGTACCTTAGCTGTGAGCTTTAGAAT
GTGGGAATCTCTAGGAATACAGAGACTATGGATCACCACCTCACAGTGGGATGTCAGTCCTAGTATGAAAGACTTT
ACATTTGGTAACGAATATGGGACTTTTGCTTTTGGACAACACCATAGTGAGATTTCTGGGTTTAAACATTTTGTCC
AGACATTGAACTCTGTCAAATGCCCAGATGAATATCTGGTAAAGCTGGAATGGATGCACTTTAACTGTGAGGTCTC
AGCCTCTAAATGTAAGACACTGAAGAACTGCTCATCCAATCACTCATTGGAATGGTTAATGGTACATACTTTTGAT
ATGGCTTTTATTGAAGGGAGTTATGACATATAC
>ms115_A3 .
AGGGTTGGTCATCTCAGACAATGATCAAGGTATCCAATTTCTCTCCTATTTGAAAACAGAGATGGAAAAAAATACA
ATCTGCTTTGCTTTTGTCAACATGATTCCAATGAGCATGAATTTGTACATGTCAAAAGCTGAAGTGTATTACAACC
AAATCATGACATCATCCACAAATGTTGTCATCATTTATGGTGACACAGACAGTACTTTAGCTGTAAGCTTTAGAAT
GTGGGAATCCCTAGGTATACAGAGACTATGGATCACTACCTCACAGTGGAATGTCAGTCCTGGTATGAAAGACTTT
ACATTTGGTAATGAATATGGGACTTTTGCTTTTGGACAACACCATAGTGAGATATCTGGGTTTAAACATTTTGTCC
AGACATTGAACTCTGTCAAATGCCCAGATGAGTATCTGGTAAAGCTGGAATGGATGCACTTTAACTGTGAGGTCTC
AGCCTCTAAATGTAAGACACTGAAGAACTGCTCATCCAATCACTCATTGGAATGGTTAATGGTACATACTTTTGAC
ATGGCCTTTATTGAAGGGAGTTATGACATATAC
>ms116_A3 .
AGGGTTGGTCATCTCAGACAGTGATCAAGGTATTCAATTTCTCTCCTATTTGAGAAGAGAGATGGAAAAATATACA
CTCTGCTTTGCCTTTGTCAACATGATTCCATTAAACATTAATTTATACATGTCAAGAGCTGAAGTGTATTACAACC
AAATCATGACATCATCCACAAATGTTGTCATCATTTATGGTGATACAGACAGTACCTTAGCTGTGAGCTTTAGAAT
GTGGGAATCCCTAGGTATACAGAGACTATGGATCACCACCTCACAGTGGGATGTCAGTCCTAGTATGAAAGACTTT
ACATTTGGTAACAAATATGGGACTTTTGCCTTTGAACAACACCATAGTGAGATTTCTGGGTTTAAACATTTTGTCC
AGACATTGAACTCTGTCAAATGCCCAGATGAATATCTGGTAAAGCTGGAATGGATGCACTTTAACTGCGAGGTCTC
AGCCTCTAAATGTAAGACACTGAAGAACTGCTCATCCAATCACTCATTGAAATGGTTAATGGTACATACTTTTGAC
ATGGCCTTTATTGAAGAGAGTTATTACATATAC
>ms117_A3 .
```

AGGGTTGGTCATCTCAGACAATGATGAAGATATCCAATTTCTCTCCTATTTGAGAAGAGAGATGGAAAAAAATACA
GTCTGCTTTGCCTTTGTCAACATGATTCCAATGAGCATGAATTTATACATGTCAAGAGCTGAAGTGTATTACAACC
AAATCATGACATCATCCACAAATGTTGTCATCATTTATGGTGACACAGACAGTACCTTAGCTGTGAGCTTTAGAAT
GTGGGAATCTCTAGGAATACAGAGACTATGGATCACCACCTCACAGTGGGATGTCAGTCCTAGTATGAAAGACTTT
ACATTTGGTAACGAATATGGGACTTTTGCTTTTGGACAACACCATAGTGAGATTTCTGGGTTTAAACATTTTGTCC
AGACATTGAACTCTGTCAAATGCCCAGATGAATATCTGGTAAAGCTGGAATGGATGCACTTTAACTGTGAGGTCTC
AGCCTCTAAATGTAAGACACTGAAGAACTGCTCATCCAATCACTCATTGGAATGGTTAATGGTACATACTTTTGAT
ATGGCTTTTATTGAAGGGAGTTATGACATATAC
>ms118_A2 .
GACTAGTCATCCAAGATGATGACCAAGGTATTCAATTTCTCTTAGATATAAAAGAAGAAATGCAAAGAAATGGAGT
CTGTTTAGCCTTTGTGAATGTGATTCCACAGAACATGCATTTATTTACAACAAGGGCTGAGAAATATTACAATCAA
ATAATTACATCTTCAGCAAATGTTGTTATACTTTATGGAGAACTGAATACTGCTTTAGAAGTGAGCTTTAAACATT
GGAAATATTTAGGCACACAGAAAATCTGGTTCACCACCTCACAATGGGATGCCATCACAAGGGAGAAAGACTTCAG
CCTTACTTCATTTTATGGGACTCTCACTATTTCACAATATCATGGAGATGTTTCAACTTTAAATAATTTTTTTCAG
GGAGCGAATCTTTCTAAGGACACACATTATTTTTCTTCTGAAAGACTTGGGTGGATGTACTTTAACTGTTCAATAT
TAAAATCTAACTGTAAAACACCAAACCAGTGCACATCCAGCAACTTATTGGAATGGTTACCACGGCACAGTTTTGA
CATGGCCATGAATGATGAGAGTTACAATATATAC
>ms84_A1 .
AGGACTGGTCATCTCAGATGATGACCAGGGTGTTCAGCTTCATTCAGATTTAAGAGAAGAAAGCCAAAGGCACGGT
ATTTGTTTAGCTTTTGTGAATGTGATCCCAGAAACCATGAAGATATACATGACAAGGGGTAATATGTATGATAAAC
AAATTATGACATCTTCAGCAAAGGTTGTTATCATTTATGGTGAAATGAACTCTACACTAGAAATTAGCTTTAGAAG
ATGGGCATATTTAGGTGCTCGGAGGATCTGGATCACAACCTCACAATGGGATGTTATCACAAATAAAAAAGATTTC
AGCCTTGATCTCTTCCATGGTACTGTCACTTTTGCACACCACAAAGGTTGGATTGCTAAATTTAAGAATTTTATGC
AAACAATGAACACTTCCAAATACCCAATAAACATTTCTCAGTCAGTACTGAGGTGGAATTATTTTAATTGTTCAGT
CTCTAAGAACAGCATTAAAATGGATCATTTCACATGCGAAAACCCATTAGAATTGACAGCACTGCACAACTATGAC
ATGGCCCTGAGTGATGAAGGTTACAATTTGTAT
>ms85_A1 .
AGGACTGGTCATCTCAGATGATGACCAGGGTGTTCAGCTTCATTCAGATTTAAGAGAAGAAAGCCAAAGGCATGGT
ATTTGTTTAGCTTTTGTGAATGTGATCCCAGAAACCATGAAGATATACATGACAAGGGGTAATATGTATGATAAAC
AAATTATGACATCTTCAGCAAAGGTTGTTATCATTTATGGTGAAATGAACTCTACACTAGAAATTAGCTTTAGAAG
ATGGGTATATTTAGGTGCTCAGAGGATCTGGATCACAACCTCACAATGGGATGTTATCACAAATAAAAAAGATTTC
AGCCTTGATCTCTTCCATGGTACTGTCACTTTTGCACACCACAAAGGTTGGATTGCTAAATTTAAGAATTTTATGC
AAACAATGAACACTTCCAAATACCCAATAAGCATTTCTGAGTCAATACTGAGGTGGAATTATCTTAATTGTTCAGT
CTCTAAGAACAGCATTAAAATGGATCATTTCACATGCAAAAACCCATTAGAATTGACAGCACTGCACAACTATGAT
ATGGCCCTGAGTGATGAAGGTTACAATTTGTAT
>ms86_A1 .
AGGACTGGTCATCTCAGATGATGACCAGGGTATTCAGCTTCATTCAGATTTAAGAGAAGAAAGCCAAAGGCATGGT
ATTTGTTTAGCTTTTGTGAATGTGATCCCAGAAACCATGAAGATATACATGACAAGGGGTAATATGTATGATAAAC
AAATTATGACATCTTCAGCAAAGGTTGTTATCATTTATGGTGAAATGAACTCTACACTAGAAATTAGCTTTAGAAG
ATGGGTATATTTAGGTGCTCGGAGGATCTGGATCACAACCTCACAATGGGATGTTATCACAAATAAAAAAGATTTC
AGCCTTGATCTCTTCCATGGTACTGTCACTTTTGCACACCACAAAGGTTGGATTGCTAAATTTAAGAATTTTATGC
AAACAATGAACACTTCCAAATACCCAATAAACATTTCTCAGTCAATACTGAGGTGGAATTATTTTAATTGTTCAGT
CTCTAAGAACAGCATTAAAATGGATCATTTCACATGCAAAAACCCATTAGAATTGACAGCACTGCACAACTATGAC
ATGGCCCTGAGTGATGAAGGTTACAATTTGTAT
>ms87_A1 .
AGGACTGGTCATTTCAGATGATGACCAGGGTATTCAGCTTCATTCAGATTTAAGAGAAGAAAGCCAAAGGCACGGT
ATTTGTTTAGCTTTTGTGAATGTGATCCCAGAAACCATGAAGATATACATGACAAGGGGTAATATGTATGATAAAC
AAATTATGACATCTTCAGCAAAGGTTGTTATCATTTATGGTGAAATGAACTCTACACTAGAAATTAGCTTTAGAAG
ATGGGTATATTTAGGTGCTCGGAGGATCTGGATCACAACCTCACAATGGGATGTTATCACAAATAAAAAAGATTTC
AGCCTTGATCTCTTCCATGGTACTGTCACTTTTGCACACCACAAAGGTTGGATTGCTAAATTTAAGAATTTTATGC
AAACAATGAACACTTCCAAATACCCAATAAACATTTCTCAGTCAGTACTGAGGTGGAATTATTTTAATTGTTCAGT
CTCTAAGAACAGCATTAAAATGGATCATTTCACATGCAAAAACCCATTGGAATGGACAGCACTGCACAACTATGAC
ATGGCCCTGAGTGATGAAGGTTACAATTTGTAT
>ms88_A1 .
AGGACTGGTCATCTCAGATGATGACAAGGGTATTCAGTTTCTCTCAGATTTAAGAGAAGAAAGCCAAAGGCATGGG
ATCTGTTTAGCTTTTGTTAATATGATCCCAGAAAACATGCAGATATACATGACAAGGGCTACAATATATGATAAAC
AAATTATGACGTCTTTAGCAAAAGTTGTTATCATTTATGGTGAAATGAACTCTACACTAGAAGTAAGCTTTAGAAG
ATGGGAAAATTTAGGTGCTCGGAGAATCTGGATCACAACCTCACAATGGGATGTCATCACAAATAAAAAAGAATTC
ACCCTTAATCTCTTCCATGGGACTATTACTTTTGCACACCGCAGATTTGAGATTCCTAAATTTAAAAAATTTATGC
AAACAATGAACACTGCCAAATACCCAGTAGATATTTCTCATACTATATTGGAGTGGAATTATTTTAATTGTTCAAT
CTCTAAGAACAGCAGTAAAATGGATCATATTACATTCAACAACACATTGGAATGGACAGCACTGCACAACTATGAT
ATGGTGATGAGTGATGAAGGTTACAATTTGTAT

>ms89_A1 .
AGGACTGGTCATCTCAGATGATGACCAGGGTATTCAGTTTCTCTCAGATTTAAGAGAAGAAAGCCAAAGGCATGGG
ATCTGTTTAGCTTTTGTTAATATGATCCCAGAAAACATGCAGATATACATGACAAGGGCTACAATATATGATAAAC
AAATTATGACATCTTCAGCAAAGGTTGTTATCATTTATGGTGAAATGAACTCTACTCTAGAAGTAAGCTTCAGAAG
ATGGGAAGATTTAGGTGCTCGGAGAATCTGGATCACAACCTCACAATGGGATATCATATTAAATAAAAAAGAATTC
ACTCTTAATCTCTTCCATGGCCCTATCACTTTTGCACACCACAAAGTTGAGATTCCTAAATTAAGGAATTTTATGC
AAACAATGAACACTGCCAAATACCCAGTAGATATTTCTCATACTATACTGGAGTGGAATTATTTTAATTGTTCAAT
CTCTAAGAACAGCAGTAAAATGGATCTTTTTACATCCAACAACACATTGGAATGGACAGCACTGCACAACTATGAT
ATGGCCATGAGTGATGAAGGTTACAATTTGTAT
>ms8_A1 .
AGGACTGGTCATTTCAGATGATGACCAGGGTATTCAGTTTCTATCAGACTTGAGAGAAGAAATGCAAAGACATGGA
ATCTGTTTAGCTTTTGTTAATATGATCCCAGAAAACATGCAGATATACATGACAAGGGCTACGATATATGACAAAC
AAATTATGGAATCAACAGCAAAGGTTGTTATCATTTATGGTGAAATGAACTCTACCCTAGAAGTCAGCTTTAGAAG
GTGGGAAGATTTAGGTGTAAGGAGAATCTGGATCACAACATCACAATGGGACGTTATCACAAATAAAAATGATTTT
AGCCTTGATTTCTTCCATGGGACTGTTACTTTTGAACACCACCACAGTGAGATTGCTAAATTTAAGAATTTTATGA
AAACAATGAACACTGACAAATACCCAGTAAACATTTCTCAGTCTATAGTGGGGTGGAATTACTTTAATTGTTCAAC
CTCAATGAACAGCTTTAGCAAAATGGATCATCTTACATTCAACAACACATTGGAATGGACAGCACTGCACAATTTT
GACATGGTCCTGAGTGAAGAAGGCTACAATTTGTAT
>ms9_A1 .
AGGACTGGTCATCTCAGATGATGACCAGGGTATTCAGTTTCTATCAGACTTGAGAGAAGAAATGCAAAGACATGGA
ATCTGTTTAGCTTTTGTTAATATGATCCCAAAAGACATGCAGATATACATGACAAGGGCTACGATACATGATAAAC
AAATTATGGAATCAACAGCAAAGGTTGTTATCATTTATGGTGAAATGAACTCTACCCTAGAAGTCAGCTTTAGAAG
GTGGGAAGATTTAGGCGCAAGGAGAATCTGGATCACAACCTCACAATGGGACGTTATCACAAATAAAAATGATTTT
AGCCTTGATTTCTTTCACGGGACTGTTACTTTTGAACACCACCACAGTGAGATTGCTAAATTTAAGAATTTTATGA
AAACAATGAACACTGACAAATACCCAGTAAACATTTCTCAGTCTATAGGGGGGTGGAATTACTTTAATTGTTCAAC
CTCAATGAACAGCTATAGCAAAATGGATCATCTTACATTCAACAACACATCGGAATGGACAGCACTGCACAATTTT
GACATAGTCCTGAGTGAGGAAGGCTACAATTTGTAT
>ms10_A1 .
AGGAATGGTCATCTCAGATGATGACCAGAGTATTCAGTTTCTATCAGACATGAGAGAAGAAATGCAAAGACATGGA
ATCTGTTTAGCTTTTGTTAATATGATCCCAGAAGACATGCAGTTATATATGACAAGGGCTACAATATATGATAAAC
AAATTATGGAATCAACAGCAAAGGTTGTTATGATTTATGGTGAAATGAACTCTACCTTAGAAGTTAGCTTTAGAAG
GTGGGAAGATTTAAGTATAAGGAGAATCTGGATCACAACCTCACAATGGGACGTTATCACAAATAAAAATGATTTC
AGCCTTGATTTCTTCCAAGGGACTGTCACTTTTGCACACCATGTAGGTGAAATTGCTAACTTTAGGAATTTCTTGC
AAACAATGAACAGTGAAAAATACACAGTAAACATTTCTGAGTCTAGACTGGGGTGGAATTATTTTAATTGTTCCAT
CTCTAAGAACAGCAATAAAAAGGATCATTTTACATTCAACAACACATTGGAATGGACAACACTGCACAAATATGAC
ATGGTCCTAAGTGAGGAAGGCTACAATTTGTAT
>ms11_A1 .
AGGAATGGTCATCTCAGATGATGACCAAGGTATTCAGTTTCTATCAGACTTGAGAGAAGAAATGCAAAGACATGGA
ATCTGTTTAGCTTTTGTTAATATGATCCCAGAAAACATGCAGATATACATGACAAGGGCTAAGATATATGATAAAC
AAATTATGGAATCAACAGCAAAGGTTGCTATCATTTATGGTGAAATGAACTCTTCCCTAGAAGTCAGCTTTAGAAG
GTGGGAAGATTTAGGTGTAAGGAGAATCTGGATCACAACCTCACAATGGGATGTTATCACAAATAAAAATGATTTT
AGCCTTGATTTCTTTCACGGGACTGTTACTTTTGAACACCACCACAGTGAGATTGCTAAATTTAAGAATTTTATGC
AAACAATAAATACTGACAAATACCCAGTAAATATTTCTGAGTCTATACTGGGGTGGAATTACTTCAACTGTTCAAC
CTCAATGAACAGCTATAGCAAAATGGATCATCTTACATTCAACAACACATCGGAATGGACAGCACTGCACAAATAT
GACATGGCCCTGAGTGAGGAAGGTTACAATCTGTAT
>ms12_A1 .
AGGATTGGTCATCTCAGATGATGACCAGTGTATTCAGTTTCTATCAGACTTGAGAGAAGAAATGCAAGGACATGGA
ATCTGTTTAGCTTTTGTTAATATGATCCCAGAAGACATGCAGTTATATATGACAAGGGCTGAGATATATGACAAAC
AAATTATGGAATCAACAGCAAAGGTTGTTATCATTTATGGTGAAATGAACTCTACCCTAGAAGTCAGCTTTAGAAG
GTGGGAAGATAAAGGTGTAAGGAGAATCTGGATCGCAACCTTGCAATGGGACGTTATCACAAATAAAAAAGATTTC
ACCCTCGATTTCTTCCAAGGGACTGTCACTTTTGCACACCATGTAGGTGATATTGCTAACTTTAGGAATTTCTTGC
AAACAATGAACAATGAAAAATACCCAATAAACATTTCTGAGTCTATACTGCAGTGGAATTCTTTTAATTGTTCCTT
CTCTAAGAACAGCAATAAAAAGGATCATTTTACATTCAACAACACATTGGAATGGACAGCACTGCACATATATAAC
ATGGTTCTGAGTGAGGAAGGTTACAATCTGTAT
>ms13_A1 .
AGGACTGGTCATCTCAGATGATGACCAAGGTATTCAGTTTCTATCAGATATGAGAGAAGAAATGCAAAGAGATGGA
ATCTGTTTAGCTTTTGTTAATATGATCCCAGGAAACATGCAATTATATATGACAAGGGCTAAGATATATGATAAAC
AAATTATGGAATCAACAGCAAAGGTTGTTATCATTTATGGTGAAATGAACTCTACCCTAGAAGTCGGCTTTAGAAG
GTGGATACATTTAGGTGTAAGGAAAATCTGGATCACAACCTCACAATGGGATGTTGTCACAAATAAAAAAGATTTC
AGTTTTAATTTCTTTCATGGGACTGTTACTTTTGCACACCACATAGGTGAACTTGTTAAATTTAGGAATTTCTTGC
AAACAATGAACAATGAAAAATACCCAGTAAACATTTCTGAGACTAGACTGGGGTGGAATTCTTTTAATTGCTCCAT

CTCTAAGAACAGCAATAAAAAGGATCATTTTACATTCAACAACACATTAGAATGGACAGCAAGGAACAATTTTGAC
ATGGTCCTGAGTGAGGAAGGTTACAATCTGTAT
>ms14_A1 .
AGGACTGGTCATCTCAGATGATGACCAAGGTATTCAGTTTCTATCAGACTTGAGAGAAGAAATGCAAAGACATGGA
ATCTGTTTAGCTTTTGTTAATATGATCCCAGAAGACATGCAGTTATACATGACAAGGGCTAAGATATATGATAAAC
AAATTATGGAATCAACAGCAAAGGTTGTTATCATTTATGGTGAAATGAACTCTACCCTAGAAGTCAGCTTTAGAAG
GTGGGAAGATTTAGGCGCAAGGAGAATCTGGATCACAACCTCACAATGGGATGTTATCACAAATAAAAATGATTTT
AGCCTTGATTTCTTTCACGGGACTGTCACTTTTGAATACCACCACAGTGAGATTGCTAAATTTAAGAATTTTATGA
AAACAATGAACACTGACAAATATCCAGTAAACATTTCTGAGTCTATACTGCGGTGGAATTACTTTAATTGTTCAAC
CTCAATGAACAGCTATAGCAAAATGGGTCATTTTACATTCAACAACACATTGGAATGGACAGCACTGCACAATTTT
GACATGGCCCTGAGTGAGGAAGGCTACAATTTGTAT
>ms15_A1 .
AGGCCTGGTTATCTCAGATGATGACCAGAGTATTCAGTTTCTATCAGACTTGAGAGAAGAAATGCAAAGACATGGA
ATCTGTTTAGCTTTTGTTAATATGATCCCAGAAGACATGCAGTTATATATGACAAGGGCTAAGATATATGATGAAG
AAATTATGACATCAACAGCAAAGGTTGTTATCATTTATGGTGAAATGAACTCTACCCTACAAGTCAGCTTTAGAAG
GTGGGAAGATTTAGGTGTAAGGAGAATCTGGATCACAACCTCACAATGGGATGTTATCACAAATAAAAATGATTTT
AGCCTTGATTTCTTTCACGGAACTGTCACTTTTGCACACCATGTAGGTAAAATTGCTAAATTTAGGAATTTCTTGC
AAACAATGAACAGTGACAAATACCCAGTAAACATTTCTAAGTCTATACTGGGGTGGAATTATTTTAATTGTTCTGT
CTCTAAGAAAGGCAATAAAAAGGATCATTTTACATTCAACAACACATTGGAATGGACAGCACTGCACAATTTTGAC
ATAGTCCTGAGTGAGGAAGGCTACAATTTGTAT
>ms16_A1 .
AGGCCTGGTTATCTCAGATGATGAACAGGGTATTCAATTTCTCTCAGGCTTAAGAGAAGAAATGCAAACATATGCA
ATCTGTTTAGCTTTTGTTAATATGATTCCGGAAACCATGCAGATATACATGACAAGGGCTGATATATATGATAAAC
AAATTATAGAATCAACAGCAAAGGTTGTTATCATTTATGGTGAAATGAACTCTACTCTAGAAGTCAGCTTTAGAAG
ATGGGGATATTTAGGTGCTCGGAGAATCTGGATCACAACCTCACAATGGGATGTCATCACAAATGAAAAAGATTTT
AGTCTTGATTTCTTCCATGGGACTATCACATTTGCCCACCACAATGGCAGAATTGCTAAATTTAACAATTTTTTGC
AAACAATGAACACTTCCAAATACCCAATAGATGTTACTCAGACCATGCAGGATTGGAATTATTTTAATTGTTCAAT
CTTTAAGAACAGTGTTAGGAAAACGGGACAGTTTATATTCAACAACACATTGGAATGGACAACACAACACAAAATT
GACATGGTCCTGAGTGAAGAAGGTCACAATTTATAT
>ms17_A1 .
AGGACTTGTCATCTCAGATGATGATCAGAGTATTCAGTTTCTCTCAGACTTGAGAGAAGCAATGCAAAGACATGGA
ATCTGTTTAGCTTTTGTGAATATGATCCCAGAAAACATGCAGATATATATGACAAGGGGTAAGATATATGACAAAC
AAATTATGACATCATCAGCCAAGGTTGTTATCATTTATGGTGAAATGAACTCTACTCTAGAAGTCAGCTTTAGAAG
ATGGGCATATTTATGTGCAAGGAGAATCTGGATCACAACCTCACAATGGGATGTCATCACAAATAAAAGAGATTTC
AGCTTTGATTTCTTCCATGGGACTGTCACTTTTGCACACCACAATGGTGAAATTGTTATATTTAGGAATTTTTTGC
AAACAGTGAACACTTCCAAGTACCCATTAGACATTTCTCAGACTATGCAGGAGTGGAATTATTTTAATTGTTCAAT
CTCTAAGAACAGCAACAGCAAAATGGATCATTTTACATTCAACAACACATTGGAATGGACAGCACTGCAAAAATTA
AACATGGTCCTGAGTGAAGAAGGCTACAATTTGTAT
>ms121_A1 .
AGGACTGGTCATCTCAGATGATGACCAGGGTATTCAGTTTCTCTCAGATTTAAGAGAAGAGAGCCAAAGGCATGAG
ATCTGTTTAGCTTTTGTTAATATGATCCCAGAAAACATGCAGATATACATGACAAGAGCTACAATATATGATAAAC
AAATTATGACATCTTCAGCAAATGTTGTTATCATTTATGGTGAAATGAACTCTACTCTAGAAGTAAGCTTTAGAAG
ATGGGAAGAGTTAGGTGCTTGGAGAATCTGGATCACAACCTCACAGTGGGATGTCATCAAAAATAAAAAAGAATTC
ACCTTTAATCTCTTCCATGGCACTATCACTTTTGCACACCACAAAGTTGAGATTCCTAAATTTAAAAAATTTATGC
AAACAATGAACACTGCCAAATACCCAGTAGATATTTCTCATACTATATTGGAGTGGAATTATTTTAATTGTTCAAT
CTCTAAGAACAGCAGTAAAATGGATCATATGACATTCAACAACACATTGGAATGGACAGCACTGCACAACTATGAT
ATGGCCATGAGTGATGAAGGTTACAATTTGTAT
>mic1 .
TGGACTGGCCATCTCAGACAATGATCAGGGTATTCAATTTCTCTCACACTTGAGGAGAGAGATGGAAAAAAATACA
GTTTGCTTTGCCTTTGTGAATATGATTCCAGTCGAAATGCGTTTATTCCAGTCAAGAGCTGAAGTTTATTATAACC
AAATCACGACATCATCCACAAATGTGGTTATCATTTATGGTGACACAGACAGTACTCTAGCTGTGAGCTTTAGAAG
GTGGCAATCTCTAGGTATACAAAGAATATGGGTCACCACCTCACAGTGGGATGTCATCACAAGTAAGAGAGACTTT
CAACTTAATTCATCCCATGGTACACTAGCTTTTGCACACCACCATGCTGATATTTCTAGCTTTAAAATTTTTGTCC
AGACATTGAACTCTCTCAAATACACAGATGAATACCTGGCAAGTCTGGAGTGGATGAACTTGAACTGTGAACTCTC
AGATTCTAACTGTAAGACCTTGAGAAATTGTTTATCCAATGCCTCACTGCAATGGCTAAAGGTACAGACTTTAGAC
ATGGCCTTTAGTGATGACAGTTATGACATATA
>mic2_PS .
TGGCCTGGCTATCTCAGACAATGATCAGGGTACTCAATTTCTCACACGCTTGAGAGGAGAGATGGAAAAACTTACA
GTCTGCTTTGCCTTTGTGAGTATGATTCCAGTCAAGATGGATTTATTCTTGTCAAGAGCTGAGTGTATTATAACCA
AATAGTGACATCATCCACAAATGTAGTTATCATTTATGGTGACCCAGAAAGTGCTCTAGCTGTGGGCTTTAGAAGG
TGGGAATCTCTAGGTTTACAGATAATATGGGTCACCTCTTGGGATGGCACTACAAGAAAGAATGACTTTCCATTTA
ATATATTCAATGGAAAAATTACTTTTGCATACCACCATGCTGAGATATCTAGTTTTAAAACTTTTGTCCAGACATT

```
GAACCCTCTCAAATACACAGATGAATTCCTGGCCAGGCTGGAATGGATGAACTTGAAATGCAATGTCTCAGCTTCT
ATGTGTAGGACCCTGCAGAATTGCTTATCCAATGCCTCATTGCAATGGTTAAAGGTACAGACTTTTGACATGGCCT
TTAGTGATGAGAGTTATGACATATA
>mic3 .
TGGACTGGCCATCTCAGACAATGATCAGGGTACTCAATTTCTCACACAATTAAGAGGAGAGATGGAAAAAATTACA
GTCTGCTTTGCCTTTGTGAGTGTGATCCCAGTCAAGATGGATTTATTCTTGCAAAGAGCTGAAGTATATTATAACC
AAATAGTGACATCATCCACAAATGTAGTTATCATTTACGGTGACCCAGACAGTATTCTAGCTGTGGGCTTTAGAAG
ATGGGAATCTCTAGGTTTACAGAGAATATGGGTCACCACCTCACAGTGGGATGGCACTACAAGTAAGCATGACTAC
CCACTTAATTCATTCAATTGGAAAATAACATTTGCATACAAGCATTCTGAGATTTCTAGTTTTAAAACTTTTGTAC
AGACACTGAACCCTCTCAAATACACTGATGAATTCCTGGCTAAGCTGGAGTGGATGAGCATGAACTGCAAAGTCTC
AGCTTCTATGTTTAAGACCCTAAAAAGTTGCTTATCCAATGCCTCATTGCAATTGCTAAAAGCACAGACCTTTGGC
ATGGCCTTTAGTGATGAGAGTTATGACATATA
>mic4_PS .
TGGGCTGCACATCTCAAACAATGAAGACTGTGCTCAATTTCTCTCAAAATTGAGAAAACACATGGAAAATATACAG
TCCGCTATGACTTGTGAATATGATCGCAGTCAACTTTACTTGTCAAGATCTGAAGTATATTATAACCAAATAATGA
CATCACCCACAATTTATTTTTCATTTATGGTGACACAGACAATACTCACACTGTGTGCTTTAGAATGTGGGAATCA
CTAGGTACATAAAGAATATGGGTCACCACCTCACAGTAGGTTGAATGTCTCTATAAGAAATAGAAACTTCACACTT
GACTCATACCATTGGGATGTGAACTTTTACAAATCTGCCTGTTGAGATTTATGCTTTTTAAATTTTGACCTTACTT
TGGACCAGTTCCTAAAATTCAGATGAAAACGTGACAAGACTAGAGGATGAGGTTTAGATGTATCAGCTTCTAGTGT
AGACACTGAAAAACTTCTCATTCAATGCCTCATTGGAATGGCTAATGGTACAGACTTTTGACATGGCCTTTAGTAA
GAGCACATATGATGTATA
>mic5 .
GGGGCTGGTCATCTCAGAAGAGAAAGATGTTCAGTTTCTCTCAGAAATAGTACGAGAGATGGAAAGAAACAGTGTC
TGTGTAGCCTTCACTGAAATGATCCCAGACAACCATTTTTTGATGAAAGAAGTTGATGTCTATGATAGCCAGATTA
TGATGAACTCAGCAAAAATTATTATTATTTATGGTGACACTGACTCAGGTTTAGGTGTCTTCTTCAGAAGATGGGA
ACATGTTCTCCCATGGAGAATCTGGGTCACCACTTCACAATGGGAAGTCACTACTTCTATGCAACATTTCATTCTT
GACTCATTCCATGGGACTCTTATCTTTTCACAACACCATGGTGAGATTTCCACTTTTAAAGATTTTGTAAAGACAG
TCACCCCTTCCAAATACCCAGAAGATATTTTTCTTGCAAGATTGTGGGAGAAATATTTTAATTGTGCACTCTCTGA
TACTTCCTGTAAACCAGTAGAAAATTGCTCATCTAGGGGTTCCTTGGAATGGTTACCTTGGTACCACTTTGACACT
GCCATGAGTGATGGCAGCTACCATGTATA
>mic6_PS .
ACAAATGTAGTTATTATTTATGGTGACACAGACAGTACTTTGGCTATGTGCTTTCGAATGTTGGAATTACTAGGTA
AAAAGAGCTATGGGCCACCACCTCACCACCTCACAGTGGGATATTACTACATGTAAAAGAGACTTCATACTTGGCT
AATCCATTGGGATGCTAGCTTTTGCACACCACTATGCTGAGAGTTCTTGTTTTAAATTGTGTTCAGACATTGACCC
ATCTCAAATATTCAGATGAATACCTGACAAGTCTAGGGGATGAATTTAGAAGGCTCAGTTTCTAAGTGTAAGACAC
TGAAAAACTGCTCATTCAATTCTTCATTGGAATATCTAATGGTACAGCCTTTTGAC
>mic7 .
TGGTCTGGCACTCTCAGATAATGACCAAGGTAACCAATTTCTCTCAGATCTGAGAGGAGAAATGGAACCCAAAAGA
GTTTGCTTAGCCTTTGTGATCATGATCCCAGGCAACACACATTTATACATCTCAAGAGCTGAAGTGTATAACAACC
AAATCATGACATCATCCACAAATGTTATCATTTATGGTGACACAGATAGTACTCTAGCTGTGTGTTTTATAATGTG
GAAATTGCAGGGTATACAGAAACTATGGTTCACCACTTCACAGTGGCATGTCATTACAAGTAAGAGAGACTTCACC
CTTGACTCATTCCATGGGACCCTTACTTTTGTACACCACCATGCTGAGATTTCTGGTTTTAAAAATTATATTCAGA
TATTTAACCCTTCAAAATACACAGACAGTTTTTTGGCAAGACTGGGGTGGATGTACTTTAGCTGTGATGCCTTAAC
GTCTAGGTGTAAGAAACTGGAGAACTGCTCATTGAATGCCTCACTGGAATGGTTAATGCTACAGAGGTTTGACATG
GCCTTGAGTGATGACAGTTATGATGTATA
>mic8 .
TGGACTGGCCATCTCAGACAATGATCAGGGTACTCAATTTCTCACACAATTAAGAGGAGAGATGGAAAAAATTACA
ATCTGCTTTGCCTTTGTGAGTGTGATCCCAGTCAATATGCATTTATTCTTGACAAGAGTTGAAGTTTATTATAACC
AAATAGTGACATCATCCCCAAATGTAGTTGTCATTTATGGTGACCCAGAGAGTTCTCTAGCTGTGGCCTTTAGAAG
GTGGAAATCTCTAGGTTTACAGAGAATATGGGTCACCACCTCACAGTGGGATGGCACTACAACTAAGAATGACTTC
CCACTTAATTCATTCAATGGAAAAATAACTTTTGCACACCATCATGCTGAGGTTTCTCATTTTAAAACATTTGTCC
AGACATTGAACCCTCTCAAATACACAGACGAATTCCTGGCCAGGCTGGAGTGGATGAACTTGAACTGCAAAGTCTC
AGGTTCTATGTGTAAGACCCTGAAGAACTGCTTATCCAATGCCTCATTGCAATGGCTAAAGGCTCAGACTTTTGAC
ATGACCTTTAGTGATGAGAGTTCTGACATATA
>mic9 .
TGGACTGACCATCTCAGACAATGATCAGGGTATCCAAATTCTCTCATATTTGAGAACAGAAATGGAAAAAAATATA
CTCTGTTTTGCCTTTGTTAACATAATTCCGCTCAACATTCATTTGTTCATAAGAAGAGCTAAAGTGTATTACAACC
AAATCTTGATGTCATCCTCAAATGTTATTATAATTTATGGTGAAACAGATAGTACTCTATCTTTAAACTTTAGAAT
ATGGACATCTCAAGGTATACAGAAAATATGGGTCACCACCTCACAGTTAGAAGTCACTACAAGTATGAGTGACTTC
TTGCCTGAATTGTTCCATGGGACTCTAGCATTTGCACACCACCATGCTGAGATTTCTGGTTTTAGAAATTTTATCC
AGACAGTGAACCCTCTCAAATACTCAGATAAATACCTCGCAGGGCTGGAATGGATGAATTTTAACTGTGGAATCTC
```

TGCTTCTATGTGTAAGACACTGAGGAACTGCTCATCCAACAGCTCATTGGAATCACAGACATTCGACATGGCTTTT
AGTGATGACAGCTATGACATATA
>mic10 .
TGGACTGGCCATCTCAGACAATGATCAGGGTACTCAATTTCTCACACAATTAAGAGGAGAGATGGAAAAAATTACA
GTCTGCTTTGCCTTTGTGAGTGTGATCCCAGTCAAGATGGATTTATTCTTGTCAAGAGTTGAAGTTTATTATAACC
AAATAGTGACATCATCCACAAATGTGGTTATCATTTATGGTGACCCAGAGAGTTCTCTAGCTATGGCCTTTAGAAG
GTGGCAATCTCTAGGTTTACAGAGAATATGGGTCACCACCTCACAGTGGGATGGCACTACAACTAAGAATGACTTC
CAACTTGATTCATTCAATGGGAAAATAACTTTTGCACACCATCATGCTGAGATTTCTAATTTTAAAACATTTGTCC
AGACATTGAACCCTCTCAAATACACAGACGAATTTCTGGCCAGGCTGGAGTGGATGAACTTGAACTGCAAAGTCTC
AGGTTCTATGTGTAAGACCCTGAAGTATTGCTTATCTAATGCCTCATTGCAATGGCTAAAGGCTCAGACTTTTGAC
ATGGCCTTTAGTGATGAGAGTTATGACATATA
>mic11_PS .
TGGGGGCCCATCTCAGACAATGAAGATGGTGCTCAATTTCTCTCATAATTGAAAAAAAAGTTATATTAAATATACA
GTCTGTTTTGACTTTATAAATATGATCACAGTCAACATGCAATTATACTTGTCAAGACCTGAAAGATATGATAACG
AAATCATGATGTCATCCACAATTTACTTTTCATTCATGGTCACACAGACAGTTCTCTATCCGTGTGCTGTAGAATG
TGAGAATCACTAGGTATACAAAGAATATGGGTCCCACCTCACAGAAGATTGATGTCTCTATAAGAAATAGAGACTT
CACAACTGACTCAACCCTTGGGGTGTTAACTGTAGCAAACCTACCTGCTGAGATTTATGCTTTTTAAATTTTGACC
TTACTTGGCCCAATCTCAAATACTCAGATGAATACCTGGCAAACTAGGTAGATGAAGTTCAGAAGTATCAGCTTCT
AAGTGTCAGACACTGAAGAAGTTCTCACTCAACTCCTCATTGGAATGACTAATGGTACAGACTTTTGACATGGCCT
TTAGTGA
>mic12_PS .
CTGAAGTGTATTATAACCAAAACATGACGTTGACTTGCTCCACAAATGTTGTGATCATTTATTTTGACACAGGCAG
TACTCTAGTTATGAGTTTTAGGATGTGAGAATCACTAGAAATACAGAGAATTTGGGTCACCACCTCACAGGGGGCT
GTCCCTACAAGGAAGTGTGACTGTGCACTTGACTCTAGATTTTAAACAACACAATGCTGAGATTTCTAATTATAAA
AACTTTTTTCTAGATATTGAACTCCCTCAAATACTCAGATGACTACTTTGCAAAGCTAGAGTGGATAAAATTCAAC
TGTGAAATCTCAACTTCTAAGGGTAAGACACAGTACTGCTCATCCAAATTTTCAATGGAATGGCTAACAGTATAGA
CTTCTGAAATGGCTTTTA
>mcr1 .
GGGACTGGTTCTCATAGATGACCATAATGGGGCTGCGATTCTCTCAGACCTGAGAGGAGAGATGGACAGGAACCGT
GTGTGTGTATCATTTGTAGAAATGATCCCAGACAATGTGGTTTCCTTTTACTTTAATTCTTTAACGATTTTCCAGA
TCATGATATCAACTGCAAATGTGGTTATCATTTATGGTGACATTGAATCTTTAGGTGGTATAATCTTACAGATAGG
GGAATTTTTAATGAATTGGAAAGTCTGGGTCCTGAGGTCACAATGGGATCTAAGTGTTGTTACTCGTTATTTCATG
TTGGATTCATTACACGGAAGTCTCATTTTTTCACATCATCATGTTGAATTTTCTGAATTTAGGAAGTTTATCCAGA
CATATAACCCTTCCAAATACCCAGATGACCATTATCTAGGTCTTGCTTGGAATACACACTTCAATTGCTCTTTTTC
TGGACCTGATTGTAAAATTCTGGGTAACTGCCTACCCAATGTTTCCTTGGAAATGTTGCCTAGAAATGCCTGGGAA
ATGGACATGACTGAAGAGAGTTACAATGTATAC
>mcr3 .
TGGTCTGGTCCTCCCAGATAACCATAAAGGGACAAATTTTCTATCAGATTTAAGAGACAAGTTTGAGAGTAGTGGA
GTATGCATGGCATTTGTAGAGATGATCCCAGGCACCTGGACTTCATTTTCCAAAAATTTTTTTAAAACTCTAGGGA
AGATACAAGAATCATTGGCCAATGTAATCATTATTTATGGTGATACAGATTCTCTACAAGGTTTAATGCGAAATCT
AGGGCAACAGTATTTGACACAGAGAGTCTGGGTTATGAACTCTCAGTGGGATGCTACACACCATGCTGAATATTTC
ATGCTAGACACTTTCCATGGGAGTCTCACTTTTGAACACCAACATGAGGAAATGATTGAGTTCACAAATTTTATTA
GAGGAGTTAATCCATATAAATACCCAGAAGACAATTACCTTCCTAAATTGTGGCATTTGTTCTTTAAATGTCAATT
TTCTAAGCTCAATTGTCAACTTTTAGAGATCTGCCAACCCAATGCTTCCTTGGAATTCTTGCCTAGACACATTTTT
AACACAGCCATGAATGAAGAGAGTTATAATATATAC
>mcr4 .
TGGACTACTCCTTCCTGATGACCACAGAGGGACAAATATTTTGTCAGATTTAAGAGAAAAGATGAAGATGAATAAT
GTATGCTTAGCTTTTGTTGAAATGATCCCAAGCACCTGGACTTCATTTTCCAACAAACTTTGGAAAAATCTGGGGA
AGTTACAAGAATCACAGGCCAATGTAATCATTATTTATGGTGACACTGATTCTCTACAAGGTTTAATGCGAAATCT
AGGGCAACAGTATTTGACCCAGAAAGTCTGGGTTATGAATTCTCGATGGGATACTACAAACCATGGTGATTATTTC
ATGATAGACTCTTTCCATGGGAGTCTCATTTTTGAACACCACCATGAGGAGATGATTGAATTTACCAAATTTATTA
GAAGAATTAATCCTTATAATTACCCAGAAGACAATTACCTTCCTAAACTATGGTTTTTGTTCTTTAAATGTCAATT
TTCTGAGGTTGATTGCCAACTTTTAGAGATCTGCCAACCCAATGCTTCCTTAGAATTCTTGCCTAAACACATTTTT
AACACAGCCATGAGTGAAGAGAGTTACAATATGTAC
>mcr5 .
AGGACTGGTTCTCATAGATGACCAAAATGGGGCTGAGGTTCTCTCAGATCTGCGAGGAGAGATGGAAAGGAACCAC
GTTTGTGTAGCTTTTGTAGACATGATTGCAGACAATGTGGCTTCCTTTAACAAGTACTTTTTAATTCATTGGAAAG
TCTGGGTCATGAGGTCACAATGGGATATAAATATTGATTCTCTCTCTTTCATGTTGGATTCATTACACGGGAGTCT
CATTTTTGCACATCATCATGTTGAGATTTCTGATTTTAGGAAGTTTATCCAGACATACAACCCTTCCAAATACCCA
GATGACCATTATCTTGCTCTTGTTTGGCATAAACACTTCAATTGTTCCTTTTCTGGGCCAGATTGTAAATTTCTGG
GTAACTGTCAACCCAATGTTTCCTTGGAAATGTTGCCTAGATATTCCTGGGTAGCGGACATGAGTGAAGAGACTTA
CAATGTATTC

>mcr6 .
GGGACTGATCATATCAGATGATGACCTAGGACTTCAATTTGCCTTTGAATTGAGAAAAGAAGTACAAAAACATGAC
ATCTGTTTAGCCTTTGTTACTATTATCACATATGACATAAAACTATTCATTAAGAGGGCTAGTACATATTATAATC
AGATCATGATGTCTTCAGCAAAAGCTGTTATAATTTATGGAGACAAAGACTCTCCTCTACAAGTGATCCTAAGACT
ATGGCAGTTTGTAGACATTCGGAGAATCTGGGTCACTACCTCACAATGGGATATGATTACAAGTAATGGAAAATTC
CTCTTCAACTCCTTCCATGGAACTCTCAGTTTTTCCCATCATTATTCTGAAATAGCTGGCTTTAAAAAATTTATAC
AGACAGTTCACCCTTCAAATTACAGTAATAGTATTTCTCTTGCTAAATTATGGTGGATATACTTTAAATGTTCTTT
ATTATCATCTAATTGCAAGAAACTGAAGAAATGTTCAGAAGATATGCTACTGGATTGGTTATCCAGGAATCAGTTT
GAAATGTCCATGAGTGACACTAGTTACAAACTATAT
>mcr7 .
GGGGTTGATTGTGTCTGAAGGAGAAAAAGGCGTTGAGATCATCTCAGACTTGGGGGCAGAAATGGACAGAAACAGA
ATATGTTTAGATTTTGTAGAAATGCTCCCAGTCAGCGAAGTATCCTATGATGCAAGCAGACAATTGAAACCTACAC
GGCTTCTGAAATCATCAGCAAATGTGATCGTTGCCTATGGTGACAGTGACTTTCTGAGAGGTTTCCTGTTTTATAT
AAAACATACTTTAGTTACCACGAAAGTCTGGATCATGAACTCAGAATGGGATATTCTCCTCCATTCAAAGCTTCTA
ATTTTGCAGTCATTCCATGGGAGTCTCATTTTCACACATCACCACAAAGAAATCTCTGGTTATGGGAATTTTATCC
AATCAGTTCACCCTTCTAAATACCCAGAAGATTTTTACCTTACGAGGTTCTGGTTCTACTTTTTTAACTGCTCATT
TGCTGACGATGATTGTAATACACTGGAGAACTGTGCGCCTAACGTTTCCTTGCGTGACTCACCTAAGAACTCTTTT
GATACGGTCATGACTGAGTACCCTTATAACACATAC
>mcr8 .
GGGGTTGATTGTGTCTGAAGGACAAAAAGGTGTTGAGATCATCGCAGACTTGGGGGCAGAAATGGACAAAAACAGA
ATATGTGTAGAATTTGTAGAAATGCTCCCAGTCAGCAAAGTATCCTTTTTAGCAAACATACAGTTGAATCCTACAC
GGCTTCTGAAGTCATCAGCAAATGTGATCATTGCCTATGGTGACAGTGACTTTCTGAGAGGTTTACTGTTTTATTT
AAAACATAGTTTAGTAACCATGAAAGTCTGGATCATGAACTCAGAATGGGATATTTTCCTCCATTCAAAACATTTC
ATTTTGCAGTCATTCCATGGGAGTCTCATTTTCACACATCACCACAAGGAAATCTCTGGTTTCAGAAATTTTATCC
AATCAGTTCACCCTTCCAAATACCCAGAAGATTTTTACCTTACGAGGTTCTGGTTCTACTTTTTTAACTGCTCATT
TGCTGATGATGACTGTAATACACTGGAGAACTGTGCGCCTAACGTTTCCTTGAATGAATCACCTAAAAACTCTTTT
GATACGGTCATGACTGAGTACCCTTACAACACATAC
>mcr9 .
GGGGTTGATTGTGTCTGAAGGAGAAAAAGGTGTTGAGATCATCTCAGACTTGGGGGCAGAAATGAACAGAAACAGA
ATATGTGTAGATTTTGTAGAAATGCTCCCAGTCAGTGAAGTATCCTTTTTAGCAATCAGACAATTGAAACCTACAC
GGCTTCTGAAGTCATCAGCAAATGTAATCATTGCCTATGGTGACAGTGACTTTCTGAGAGGTTTCCTGTTTTATTT
AAAACATACTTTAGTTACCACGAAAGTCTGGATCATGAACTCAGAATGGGATATTTTACTCCATTCAAGGCTTTTC
ATTTTGCAGTCATTCCATGGCAGTCTCATTTTCACACATCGCCACAAAGAAATCTCTGGTTATGGGAATTTTATCC
AATCAGTTCACCCTTCTAAATACCCAGAAGATTTTTACCTTGCAAGATTCTGGTTCTACTTTTTTAACTGCTCTTA
TGCTGATGATGACTGTAATACATTGGAGAACTGTGCACCTAATGTTTCCTTGTATGAATCACCTAAGAACTCTTTT
GATACTGTCATGACTGAATATGCTTACAACACATAC
>mcr10 .
AGGGATGGTCATCTCAGACAATGATCAGGGCACCCAATTTCTCTCATATTTAAGAAGAAAGTTGGAAAAAAATACA
ATCTGCTTTGCCTTTGTAAATATGATTCCAATCAACATGCAGCTATACATGTCAAGAGCTGAAGTGTATTATAACC
GAATCAAGACATCATCCACAAAAGTTGTTATCATTTATGGTGACACAGACAGTACTCTTGCTGTGAGCTTTCGAAT
GTGGGAGTCCAGAGGTTTACAGAGAATATGGGTCATCACCTCACAGTGGGCTGTGACTACAAGCAAAAGAGACTTC
ATGCTTGACTCATCACAAATGACTCTAGCTTTTGCACACCATCATGGTAAGGCTACTGGTTTTAAAAATTTTGTCC
AGACAATGAACCCTCTCAAGTACACAGATGAATACCTTGCAAGGCTGGAGTGGATGAACTTTAACTGTGAAGTCTT
CACATCCAACTGTCAGACACTAAAGAACTACTCATTGAATGTGACTAAGGAATGGCTACTGGTGAGGACTTTTGAC
ATGGCCTTTACTGATGACAGTTATGACATATAC
>mcr11 .
AGGACTGTTCATTGCAGATGACCACAAAGGCTCCCAGATTCTATCAGACTTGAGAAGAGAGATGGACAAAAATGGA
GTCTGCATAGCATTTGTGGAAATGATCCTAGTCAGCAGGGGATCGATTCTGCCCAGACCCTGGAAAAATCAGGTGC
AGATACTGGAATCATCAGCAAATGTGATTATTATTTATGGGGACACTGATTCTCTGTTGAGCTTAATAGTAAATAT
TAAGCAGAAGTTAGTCACATGGAAAGTCTGGATCCTGACCTCACACTGGGATGCTTCCAAATTTGATGATTATTTC
ATGTTAGATTCCTTGCATGGGGCTCTTATTTTTTCACACCAATATGGTGAGATTGCTAATTTTACAGATTTTATCC
AGACAGCCAACCCTTCCAAATACCCAGAAGACATTTACCTTCATGTATTGTGGCAAATGTTCTTCAACTGCTCATT
TTCAAGGAAGAAATGTAAAATATTGGGTAACTGTCTGCCCAATGCCTCCTTGGAATTCTTGCCTGGGAATATATTT
GACATGACCATGAGTGAAGAGAGTTACCATGTGTAC
>mcr12 .
GGGACTCCTTATTGCAGATGACCACAAAGCTACTCAAATTCTATCCAACCTAAGAAAAGATATGGATAAAAAGGGA
ATATGTATGGCATTTGTGGAAATGATTCACATCAGCAGGGGCTCATTTCTGACAAACTCCTGGAAAAATCAGCTGC
AGATTATGGGATCATCAGCAAATGTGATTATCATTTATGGAGACACTGATTCTTTATTGAGTTTAATAGCAATTGT
AAATAGGGGGTTCCTTATGTGGAAAGTCTGGGTTCTAAACTCAAAATGGGATGTTTCCACTCTTGATAAATATTTC
CTCTTAGATTTATTGCATGGGACTTTAATCTTTTCACACCATCATGAGGAGATTGTAAATTTTACAGATTTTATTC
AAGCAGCCAGCCCTTCCAAATTCCCGGAAGACATTTATCTTCACCTATTGTGGCAAATGTTCTTCAACTGCTCATT

TTCAGGGAAGTATTGCAAAATTGTGGATAACTGTCAGCGCAATGCCTCTTTGGCATTCTTGCCTGGAAACATATTT
GACATGGCCATAAGTGAAGAGAGTTACAATGTGTAC
>mcr13 .
GGGCCTGGTTATCTCAGAAGAAGAAAAGGGTGTTCAGTTTGTCTCAGAACTGGTACCAGAGATGGACAGAAATGGA
CTCTGTGTAGCCTTCATTGATATGATTCCAATCATGCATAAGTATAACCTTGTTAGCCCAAGAGAATATTATGCTG
ATATTATGATGGGCTCAGCAAAAATTATTGTCATTTATGGTGTCACAGACTCTGCTTTAGGTGTGGTTTTCAGCAT
TTGGAAATACTTACTCCCATGGAGAATCTGGGTCACTACTTCACAATGGGAAGTCTTTACTACTATGAAACATTTT
ATTCTTGACTCATTTCATGGGAGTCTCATTTTTTCACAACACCATGGTGAGGTTTCTACTTTTAAAGATTTTGTAA
AAACATTCACCCCTTCCAAATACCCAGAAGACATTTTCCTTGCAAGATTGTGGAAGATTTATTTTAATTGTACAAT
GTCTAAGGATTCTTGTAAAGCAATAGAAAATTGTTCATCAAGAGGTTCATTGGCATGGTTACCTTGGTACCACTTT
GACAAGGCCATGAGTATTGGAAGTTACCATGTATAT
>mcr14 .
GGGGCTGGTCATCTCAGAAGAAGATAAAGTTGTTCAGTTTCTCTCAGAATTGATACCAGAGATGGAAAGAAACAGA
GTCTGTGTAGCCTTCAAGGCAATGGTCCCAATAAACTATTTGGTCCTCATGAGAGACTCTGGTGTCTACTATAGGC
AGATTGTGATGAACTCAGCAAAAATTATTATCATATATGGTGACACGGACTCATCTTTATGCGTCCTCTTCAGTAG
ATGGGAATATGTATTTCCATGGAGAATCTGGGTCTCCACTTCACAATGGGAAGCCGTTACTGCTATGAGACTTTTC
ATTCTTGACTCATTCCATGGGACTCTCATCTTTTCACAACACCATGGTGAGATTTCTACTTTTAAAGATTTTGTAA
AAACAGTCACCCCTTCCAAATACCCAGAAGACATTTTCCTTGCAAGACTGTGGAAGATTTATTTTAATTGTGCACT
CTCTGACATTTCCTGTAAAGTAGAAGAAAACTGCTCATCTAGTGGATCATTGGAATGGTTACCTTGGTACCACTAT
GATACTGCCATGAGTGATGGAAGTTACCATGTATAT
>mcr15 .
GGGGCTGATCATCTCAGAAGATGAAAAAGGTGTTCATTTTGTCTCAGAACTGATACTAGAGTTGGAAAAAAACAGT
GTCTGTGTAGCGTTCATGGAAATGCTCCCAGTCATGAATGTACATTACATTGTTGAACCTAATATCATTCAAAATA
GGATTGAGGCAAGCACAGCAAAAGTAATTATCTGCTATGGTGACACAGAGTCTTCTTTAGGAATACTCTTCAGAAT
ATGGGAACATATACTCCCATGGAGAATCTGGGTAACCACTTCACAATGGGAAGTCTTTACTGCTATGAAACATTTA
TTTCTTGATTCATTTCATGGAACTCTAATTTTTTCAAAACACCATGGTGAGATTTCTACTTTTAAAGATTTTGTAA
AGACAGTCACCCCTTCCAAATACCCAGAAGACATTTTCCTTGCAAGATTGTGGGAGATTTATTTTAATTGTCCACT
TTCACATGCTTCCTGTAAATCAGTAGAAAATTGTTCATATAAAGGTTCCTTGGAATGGTTACCTTGGTACTACTTT
GAAAAGGCCATGAGTGATGGCAGTTACCATGTATAC
>mcr16 .
AGGGTTGGTTATCTCAGATGATGACCAAGGAATCCAGTTCCTCTCAGACTTGAGAGCAGAAATGCAAAGGAAAGGA
ATTTGTTTAGCTTTTGTGAATGTGATCCCAGATAGCATGCAGTTATACATGGCAGGAGCTGTAATATATGACAACC
AAATCATGACATCATCAGCAAAGGTTGTTGTCATTTATGGTGAAATGAACTCTACCCTAGAAGTCAGCTTCAGAAG
ATGGGCATATTTAGGTGTACAGAGAATCTGGGTCACCACCTCACAATGGGATGTCATCACAAGTAAGAGAGATTTC
AGCCTTGACCTCTTCCATGGGACTGTCACTTTTATACACCACCATGCTAAGGTTCCTAAATTTAGGAATTTTGTGC
GAACACTGAACACTTCCAAATATCCAGTAGACATTTCTCAGATGAGAACAAAGTGGAATTTTTTTAATTGTTCAGT
CTCTGAAACCAATTATAGCTCAATGAATCATGGTTCAATCAACACTACATTGGAATGGTTATCACAACACAAATTT
GACATGGTCTTGAGTGAAGAAGGTTACAATTTATAC
>mcr18 .
GGGGCTGGTCCTCATAGATAACTATAAAGGAAATAAGATTCTATCAGACATGAGTGGAGAGATGTACAGGAATAGA
GTGTGCTTAGCATTTGTAAAAATGGTGCAAGAGAACTTGTTTTGTTTTGGCTATTCATCCCTAGAGACTGTCATGG
TGATTAAGAAGTCATCTGCAAATGTTGTTATCATTTATGAACACAGGGAATCTTTACATTGCTTAATGCTATGTGG
ATTGCATATTGTAAAAACATGGAAAGTCTGGGTCATGAATTCAAAGTTTGATATGAGTATCACTTCTGAGCCTTTT
ATTTTTGATCCATTTCATGGGACTCTCCTTTTTACACATCACCATGATGAGATTTCTGACTTTAAAACTTTTATTC
AGGCATATACCCCTTCTAAATATCCAGATGACTATTATCTTAATATTATATGGAATGGTTGGTTCAATTGCTCTTT
ATCTCTACCTTATTGTAAAGTATCTGGTAACTGTCTACCGAATGCTTCATTGGAATTGATTCCTTCAAATACATGG
GAAATGGACATGACTGAAGCAAGTTACAATATATAT
>mcr19 .
TGGGTTGGTCCTCACAGATGACCACAAGGCATCTCAGATTCTATCTGATTTGAGAGGAGAAATGGACAGAAATAAA
GTGTGCATAGCTTTTGCAAAAATGATTCCAGACACTCAGTTTCTCACTCTCAACTCTCTCATGAAAACTCGTGTGC
AGATTCTGGAATCATTGACAAATGTGATTATCATTTATGGTGACTATGATTCCCTAAAATTTTTAATATTTACCTT
ATCACATACTTTACTGACAATGAAAGTCTGGGTCTTGAAATCACGACTACATGCTCCTTCCTATTCTCATTCACAT
GAGTTCTTGGTTGACTCATTCCATGCAGGTCTCATTTTTTCACACCATCATGCAAAAATTCCTGGTTTTAAACATT
TCATGCAGACAGTTAATCCTTTCAAATACCCAGAAGACAATTTCCTTGCTATTTTGTGGCATCAAAACTTCAATTG
TTCTTTTTCTCAACATGATTGTAAAATTGTGGGTATCTGTCTACCCAATGCTTCCTTGGAACAGTTGCCTAAGAAA
AATTGGGAAATGGGCATGACTGAAGAGAGTTATAATATATAT
>mcr20 .
AGGGCTAGTCATCTCTGACAATGATCAGGGCACCCAATTTCTCTCACACTTGAGAAGAGTGTTGGAAAAACATACA
GTTTGCCTTGCCTTTGTAAATGTGATTCCAATCAGCATGCAGTTATACATGTCAAGAGCTGAAATATATTATAACC
AAATTAAGACGTCATCCGCAAATGTTGTTATCATTTATGGTGATTCAGACAGTACTCTTGCTGTGAGCTTTCGATT
GTGGAAATCCCAAGGTTTACAGAGAATATGGGTCATCACCTCACAGTGGGATGTGACAACTAGTAAAAGAGACTTT
GTATTTCATACAGCCCAAATGACTCTAGCTTTTGCTCAGCATCATGGTGATATTTCTGGTTTTAAAAAATTTGTTA

AGAAAGCGAACCCTATCAAAAACAAAGATGAATACCTTGCAAGGATGGAATGGATGAACTTTAATTGTGAAGTTTC
TACATCCAACTGTAAGACACTGAAGAAATACTCATGGAATACCTCCTTGGAATGGCTAGTGGTACGGACTTCTGAC
ATGGCCTTTAGTGATGGCAGTTACGACATATAC
>mcr21 .
GGGGCTGGTCATCTCTGACAATGATCATGGCATTCAATTTCTCTCATATTTGAGAAGAGAGTTGGAAAAATACATA
GTCTGCCTTGCCTTTGTAAGTATGATTCCAATCAACATGCAGTTATACATGTCAAGAGCTGAAGCATATTATAACC
AAATCAAGACATCATCGGCAAATGTTGTTATCATTTACGGTGACTATGAGAGTACTCTTGTTGTGAGCTTTCGAAT
GTGGGAATCCCAAGGTTTACAGAGAATATGGGTTATCACCTCACAGTGGGATGTAACTGCAAGTAAAACAAACTTC
ACGGTTCATACAGCCCAAATGATTCTAGCTTTTGCTCACCATCATGGCGATATTTCTGGTTTTAAAAGTTTTGTCC
AGACAATGAACCCAATCAAAAACACAAATGAATACCTTGCAAGGATGGAATGGATGAACTTTAACTGTGAAGTTTC
TACATCCAACTGTAAGACACTGAAGAAATACTCATGGAATACCTCGATGGAATGGCTATTGGCACAGACTTTTGAC
ATGACCTTTAGTGATGGCTGTTATGACATATAT
>cr1 .
GGGGATCATTGTGGCTGATGACCTAAAAGGAAAGGAGTTCCTCTCTGACCTGACAGCAGAGCTTGCTTCAGAAGAT
ATATGTGTGGCTTTTACAGAAAAAATACCAACTTATATGAAAGTGGAGTTACTGTTTGGTGGTGGATTGGTGAATT
TGAGCCAACATACAAAAGTAAATGTGCATGTGTTCTATGGGGATATAGATGATGTCCTGTTTTTCTACATACTAAA
TGAAATTAGGTCAAATAGAAAGAAGATGTGGATCATGGCAAAGCCGCACTTTGTTTACTTACAGTCTGTATTTTAT
AAGTGGATTGGTTTGATGTCCTTTTTTGAAGGTAGCTTCTCATTTTCAAAGAACAGAAATATCCCTGGCTTCAAGC
ACTTTATCCAGGCACTTACACCCTCACAGTACCCAGGTGATATTTACTTCCATAAATTCTGGCTGGACAATTTTCA
CTGTTCACCTTCTCCTTTGCTATGTGGAGGTCAAGAACTCTGCCCACTGAATATATCGCTAAAGAATAAACAGGAA
ATACAGGTTTTGATGATCACTTCTGAGCCCAGCGTTTCCATATGG
>cr2 .
GGGAATCATTGTAGCTGATGACCTAAAAGGAAAGGAGTTCCTCTCTGACATGACAGCAGAGCTTGCTTCAAAAGAC
ATCTGTGTGGCTTTTACAGAAAAAATACCAACTTATATGAAATTGGAGTTACTGTTTGGGGGTGGATTGGTGAATT
TGAGCCAACATACAAAAGTAAATGTGCATGTATTCTATGGGGATATAGATGATGTCCTGTTTTTCTACATACTAAA
TGAAATTAGGTCAAATAGAAAGAAGATGTGGATCATGGCAAAGCCGCACTTTGTTTACTTACAGTCTGTATTTTAT
AAGTGGATTGGTTTGATGTCCTTTTTTGAAGGTAGCTTCTCATTTTCAAAGAAGAGAAATATCCCTGGCTTCAAGC
ACTTTATCGAGGCACTTACACCCTCACAGTACCCAAGTGATATTTACTTCCATAAATTCTGGCTGGACAATTTTCA
CTGTTCACCTTCTCCTTTGCTATGTGGAGTTCAAGAACTCTGCCCACTGAATATATCGCTAAAGAATAAACAGGAA
ATACAGGTTGTGATGATCACTTCTGAGCCCAGCGTTTCCATATGG
>cr3 .
GGGGCTCATTGTGGCTGATGACCTAAATGGAAAGGAATTCCTCTCTGACCTGACAGCAGAGCTTGCTTCAGAAGAT
ATCTGTGTGGCTTTTACAGAAAAAATACCAACTTTTAAAAAAAATGGATTTTCTGTTTTGGGTTGGGGTGGTGAAT
TTGAGCCAACATACAAAAGTAAATTGCATGTGTTTTATGGGGATATAGATGATGTCCTGATTTTCTACATACTAAA
CGAAATTAAGTCAAAGACAAAGAAGATGTGGATCATGGCAAAGCCCAACTTTGTTTACTTACAATCTGTATTTTAT
AAGTGGATTGGTTTGATGTCCTTTTTTGAAGGTAGATACTCATTGTCAAAGAAGAGAAATATCCCTGGCTTCAAGC
ACTTTGTCCAGGCACTTACTCCCTCACATTACCCAAGTAGTATTTACTTCCATAAATTCTGGCTGGACAATTTTCA
CTGTTCACCTTCTCCTTTGCTATGTGGAGGTCAAGAACTCTGCCCACTGAATATATCGCTAAAGAATAAACAGGAA
ATAAAGGTTGTGATGATAACTTCTGAGCCCAGCGTTTCCATATTG
>cr4 .
GGGAATCATTGTAGCTGATGACCTAAAAGGAAAGGAGTTCCTCTCTGACATGACAGCAGAGCTTGCTTCAGAAGAT
ATCTGTGTGGCTTTTACAGAAAAAATACCAACTTTTAAAAAAATGGATTTTCTGTTTGGGGTTGGGGTGGTGAATT
TGAGCCAACATACAAAAGTAAATGTGTATGTGTTTTATGGGGATATAGATGATGTCCTGATTTTCTACATACTAAA
CGAAATTAAGTCAAAGAGAAAGAAGATGTGGATCATGGCAAAGCCCAACTTTGTTTATTTACAATCTGTATTTTAT
AAGTGGATTGGTTTGATGCCCTTTTTTGAAGGTAGCTTCTCATTTTCAAAGAAGAGAAATATCCCTGGCTTCAAGC
ACTTTATCGAGGCACTTACACCCTCACAGTACCCAAGTGATATTTACTTCCATAAATTCTGGCTGGACAATTTTCA
CTGCTCACCTTCTCCTTTGCTATGTGGAAGTAAAGAACTCTGCCCACTGAATATATCGCTAAAGAATAAACAGGAA
ATAAAGGTTGTGATGATAACTTCTGAGCCCAGCGTTTCCATATGG
>cr5 .
GGGGCTCATTGTGGCTGATGACATACATGGAAGGGAGTTCCTCTCTGACCTGACAGCAGAGCTTGCTTCAGAAGAC
ATCTGTGTGGCTTTTACAGAAACAATACCAACTTATATGAAAGTGGATTTTCTCTTTGAGGTAGGGATGGTGGATT
TGAGCCAACATACAAAAGTAAATGTGTATGTGTTTTATGGCGATATAGATGATGTAACGATTTTCTACATAGAAAA
TGAAATTATATCAAAGAGAAAGAAGATGTGGATCATGGCAAAGCCCAACTTTGTTTACTTACAATCTGTATTTTAT
AAGTGGATTGGTTTGATGTCCTTTTTTGAAGGTAGCTTCTCATTTTCAAAGAAGAGAAATATCCCTGGCTTCAAGC
ACTTTGTCCAGGCACTTACTCCCTCACATTACCCAAGTAGTATTTACTTCCATAAATTCTGGCTGGACAATTTTCA
CTGTTCACCTTCTCCTTTGCTATGTGGAGTTCAAGAACTCTGCCCACTGAATATATCGCTAAAGAATAAACAGGAA
ATACAGGTTGTGATGATCACTTCTGAGCCCAGCGTTTCCATATGG
>cr6 .
GGGGCTCATTGTGGCTGATGACCTAAACGGAAAGGAGTTCCTCTCTGACCTGACAGCAGAGCTTGCTTCAGAAGAT
ATCTGTGTGGCTTTTACAGAAAGAATACCAACTTATATGAAAGTGGATTTTTACTTTGGGGTTGGGTTGGTGAATT
TGAGCCAACATACAAAAGTAAATGTGCATGTGTTCTACGGGGATATAGATGATGTCCTGATTTTCTATATACAAAA
TGAAATTATATCAAAGAGAAAGAAGATGTGGATCATGGCAAAGCCCCGCATTGTTTACTTACAATCTGTATTTTAT

AAGTGGATTGGTTTGATGTCCTTTTTTGAAGGTAGCTTCTCATTTTCAAAGAACAGAAATATCCCTGGCTTCAAGC
ACTTTATCCAGGCACTTACACCCTCACAGTACCCAAGTGATATTTACTTCCATAAATTCTGGCTGGACAATTTTCA
CTGTTCACCTTCTCCTTTGCTATGTGGAGTTCAAGAACTCTGCCCACTGAATATATCGCTAAAGAATAAACAGGAA
ATACAGGTTGTGATGATCACTTCTGAGCCCAGCGTTTCCATATGG
>cr8 .
AGGTCTTGTCATCTCAGATGATGACCAGGGAATCCAGTTTCTCTCAGACTTGAGAGAAGAGATGCAAAGAAAGGGA
ATCTGTTTAGCTTTTGTGAATATGATCCCAGATAGCATACAGTTATACACGAAAAGGGCTGTAATATATGACAACC
AAATCATGACATCATCAGCAAAGGTTGTTGCCATTTATGGTGAAATGAACTCTACCCTAGAAGTCAGCTTCAGAAG
ATGGGCATATTTAGGTGTACAGAGGATCTGGGTCACTACCTCACAATGGGATGTCATCACAAGTAACAAAGATTTT
AGCCTTGACCCTTTTCATGGGACCATCACTTATATGCATCACCATGGTAAGGTTTCTAAATTTATGAATTTTATGA
GAACATTGAATACTTCCAAATATCCAGTAGATATTTCTCAGATGAAAATGAAGTGGAATTTTTTTAATTGTTCAGT
CTCTGACACCAACTATAGCTCAATGAATCATTATCTATATAACACTACATTGGAATGGTTGTCACAACACAAATTT
GACATGGTCTGGAGTGAAGAAGGCTACAATTTATAC
>cr9 .
TGGGCTGGCCATCTCAGACAATGATGAGGGCATACAATTTCTCTCTTATTTAAGAAGAGAGATGGAAAAAAGTATA
CTTTGCTTTGCCTTTGTGAACATGATCCCAGTCGAATTGCATTTATACATATCAAGAGCTGAAGTGTATTATAAAG
AAATAGTGACCTCATCCACAAATGTTGTTATCATTTATGGTGACACAGACAGTACTCTAGCTGTGAGCTTTAGAAT
GTGGGAATCTCAGGGTATACAGAGAATATGGGTCACCACCTCACAGTGGGATGCCACTACAAATAAGAAAGACTTC
ACACTTGGCTTACAACATGGGACTATTGCTTTTGCACACCACCATGCTGAGATTTCTGGTTTTAAAACTTTTGTTC
AGACATTGAACCCTGTCAAATATTCAAATGAATACCTGGCTAAGCTGGAGTGGATGAAATTTAACTGTGAAGCCTC
AACTTCTATGTGTAAGACACTGAAAAACTGCTCATCCAATGCCTCATTGGAAGCTCTAATGATAGAAACTGTTGAC
ATGGCCTTTGGTGATGAGAGTTATGGCATATAC
>cr10 .
TGGGCTCGCCATCTCAGACAATGATCAGGGTACACAATTTCATGCATATTTGAGAAGAGAAATGGAAAAAAACACA
ATCTGCTTTGCCTTTGTGATTATCATCCCAGAAAAGATGCAGCTATTCATGTCAAGATCTGAAATGTATTATAACC
AAATCATGACATCATCCACAAATGTTGTTATCCTTTATGGTGATACAGACAATACTCTACTTGTGAGCATAAAAAT
GTGGGAATCTCTCGGCATACATAAAATATGGATTACTACCTCAAAGTTGGATGTAACCACAAGTGAGAAAGATTTC
ACACTTAACAATGCTCATGGGATTCTGTCTTTTGCACATCACCATGCTGAGATTTATGGTTTTAAAAAATTTGTTC
AGACATTGAACCCTTTCCAATACTCAGAGGATGTCCTGGCAAGGCTGGAGTGGATGAACATTAACTGTGAAGTCTC
AGCTTCTAAGTGTAAGACCCTGAAGAACTGTTCATCCAGTGCCTCTTTGGAATGGCTAATGGTAGAAACAGTTGAC
ATGGCCTTTAGTGATAGCATTTATGATGTAATC
>cr11 .
TGGGCTCGCCATCTCAGACAATGATCAGGGTACACAATTTCATGCATATTTGAGAAGAGAAATGGAAAAAAACACA
ATCTGCTTTGCCTTTGTGATTATCATCCCAGAAAAGATGCAGCTATTCATGTCAAGAGCTGAAATGTATTATAACC
AAATCATGACATCATCCACAAATGTTGCTATCCTTTATGGTGATACAGACAGTACTCTCCTTGTGAGCATAAAAAT
GTGGGAATCACTCGGCATACATAAAATATGGATTACTACCTCACACTTGGATGTAACCACAAGTGAGAAAGACCTC
AGACTTGATAATGCTTATGGGATTCTATCTTTTGACCATCACCATGCTGAGATTTATGGTTTTAAAAATTTTGTTC
AGACATTGAACCCTCACCAATACTCAGAGGAAATCCTGCCAAGGCTGGAGTGGATGAACATTAACTGTGAAGTCTC
AGCTTCTAAGTGTAAGACCCTGGAGAACTGCTCGTCCAATGCCTCTTTGGAATGGCTAATGGTAGAGACTGTTGAC
ATGGCCTTTAGTGATAGCACTTATCACGTATAC
>cr12 .
AGGGCTGGTCATCTCAGACAACGATCAGGGCACCCAATTTCTCTCATATTTGAGAAGAGAGATGGAAAAAAGTACA
GTCTGTCTTGCCTTTGTCAATAAGATTCCAATCAACAAGCAGTTATACATGTCAAGAGCTGAATTGTATTATAACC
AAATAGAGACATCATCCGCAAATGTCGTTATCATTTATGGTGACCCAGACAGCACTCTTGCTGTGAGCTTTCAAAT
GTGGCAATCCCGAGGTTTACAGAGATTATGGGTCATCAATTCACACTGGGATGTGATTACAAGTAAAAGAGACTTC
ATGCTTGACTCATCCCATATGACTCTAGCCTTTGCCCACCATCATGGTGAGATTTCTGGCTTTAAAAATTTTTGCC
AGCCAATAAACTCTCTCAAGTACACAGATGAATACCTTGCAATGATGGAATGGATGAACTTTAACTGTGACATCTC
CACATCCAACTGTAAGACACTGAGGAACTACTCATCAAATTTGTCAGTGGAATGGCTAGTGGCACAGACTTTTGAC
ATGGCCTTTAGTGATGGCCTTTATGACATATAC
>cr13 .
AGGGCTGGTCATCTCAGATGATGATCAGGGTACCCAATTTCTCTCATATTTGAGAAGAGAGTTGGAAAAGAATACA
GTCTGCTTTGCCTTTGTAAATATGATCCCAATTAACATGCAGTTATACATATCAAAAGCTGAATTGTATTATAACC
AAATCAAGGAATCATCCACAAATGTTGTTATCATTTATGGAGACTCATACAGTACTCTTGCTGTGAGCTTTCAATT
TTTGGCATCCCGAGGTTTACAGAGAATATGGGTCATCACCTCACAGTGGGATACGACTACAAGTAAAAGAGACTTC
ATGCTTGACTCATCACATTTGACTCTAGCTTTTGCACACCATCATGGTGAGATTTCTGGGTTTAAAAATTTTGTCC
AGACAATGAATCCTATCAAATACACAGAAGAATACCTTGCAAGGCTAGAGTGGATGAACTTTAACTGTGACGCTTC
CATATCTAACTGTAAAACACTGATGAATGACTCATTGAATAACTCCATGGAACGGCTAGTGGTACGGACTTTTGAC
ATGTCCTTTAGTGATGGCATTTATGACATCTAT
>cr14 .
AGGCTTGGTCATCTCAGACGATGATCAGGGTACCCAATTTCTCTCATATTTGAGAAGAGAGTTGGAAAAAAATACA
GTCTGCTTTGCCTTTGTAAATATGATTCCAATCAACATGCAGTTATACATGTCAAGAGCTGAATTGTATTATAACC
AAATCAAGGAATCATCCACAAATGTTGTTATCATTTATGGAGACTCAGACAGTACTCTTGCTGTGAGCTTTCAATT

GTGGGCATCCCAGGGTTTACAGAGAATATGGGTCATCACCTCACAGTGGGATGTGACTACAAGTAAAAGAGACTTC
ATGCTTGACTCATCACATATAACTCTAGCCTTTGCACACCATCATGGTGAAATATCCGGGTTTAAAAATTTTGTCC
AGAAAATGAATTCTATCAAATACACAGAAGAATACCTTGCAAGGCTGGAGTGGATGAACTTTAAATGTGAAGCTTC
CACATCTAACTGTAAAGCACTGATGAACGACTCATTGAATAACTCCATGGAAAGGCTAGTGGTACAGACTTTTGAC
ATGTCCTTTAGTGATGGTATTTATGACATCTAC
>cr15 .
AGGGCTGGTCATCTCAGACAATGATCAGGGCACCCAATTTCTCTCATATTTGAGAAGAGAGTTGCAAAAAAGTACA
GTCTGTTTTGCCTTTGTAAATATGATTCCAATCAACATGCAGTTATACATGTCAAGAGCTGAATTGTATTATAGCC
AAATCAAGGCATCATCCACAAATGTTGTGATTATTTATGGTGACTCAGACAGTACTTTTGCTATGAGCTTTCAAAT
GTGGGAATCCCAAGGTTTACAGAGACTATGGGTCATCACTTCCCAGTGGGATGTGACTACAAGTAAAAGAGACTTT
ATGCTTGACTCATCACATATGACTCTAG
>cr17 .
AGGCCTGGTCATCTCTGACAATGATCAGGGTAAGGAATTTCTCTCATATTTAAGAAGAGAGTTGGAAAACAATACA
ATCTGTTTGGCCTTTGTAAATATGATTCCAATCAACACACAGTTGTACATGTCAAGAGCTGAAACTTATTATAACC
AAATTGACACATCATCCACGAATGTTGTTATCATTTATGGTGACACAGACAGTACTCTTTCTGTGAGCTTTCAAAT
GTGGGAATCCAGAGGTTTACAGAGAATATGGGTCATCACCTCACAGTGGGATGTGACTATTAGTAAAAGAGAATTC
ATTCATGACTCATCCCATATGACTCTAGTTTTTGCACAATATCATGGTGAGATTTCTGGCTTTAAAAATTTTGTCC
AGAGAATGAACCCTCTGGAATACACAGATGAATACCTTGCAAGGCTGGAGTGGATGAATTTTAACTGTGACGTCTC
CATGTCCAATTGTAATACACTGAAGAACTGCTCTTTGAATGACGCTATGGAATGGGTATTGGTACGGACTTCTGAC
ATGGACTTTAGTGATGACGATTACAACATATAC
>cr18 .
TGGACTGATCATTTCAGATGATGATCTAGGACTTCAATTCTCCTCTGAATTGAGAAAAGAGATGCAAAGACATGAC
ATCTGTTTAGCCTTTGTGACTATTATCACATATGACATAAAATTATTCTTTAAAAGAGTTAGTATGTATTATAATC
AGATCATGATGTCTTCAGCCAAAGTTGTTATAATTTATGGAGACAAAGATTCTCTTCTACAAATGAACCTAAGACT
ATGGCAGTTTGTAGACATTCGGAGAATCTGGGTCACTACTTCTCAATGGGATATGATTACAAGTAAAGGAGAATTC
CTCTTCAACTCCTTCCATGGGACTCTCAGCTTTTCACATCATTATTCTGAGATAGCTGGCTTTAAAAAATTTATGC
AGACAGTACACCCTTCAAACTACACTAATAGTATTTCTCTTGCTAAATTATGGTGGATATATTTTAATTGTTCTTT
GTCATCAACTAATTGCAAGAAACTGAAGAAATGTTCAGGAAAAATGCTACTGGAATGGTTATCCAGGTACCAGTTT
GAAATGTCCATGAGTGACACTAGTTACAAACTATAT
>cr19 .
TGGTGCAATCATTTCAGATGATGACCTAGGCCTTCAATTTCTCTCAGAATTGAGAAAAGAGATGCAAAGAAATAGT
GTTTGCTTAGCCTTTGTGCATATTATTATGGAAGATAAAATATTATTCCAGAAAAATGTAAATATCTATTATAATG
AAATCACAACATCATCAGCCAAAGTTGTTATCATTTACGGAGACAAAGACTCTCATCTGCAGCTCAACCTTAAACT
ATACAGATTAGTAAATGTTCAGAGGATATGGGTCACTACCTCACAATGGAACTTGATCACATATAATGAGAGATTA
CTTCTTAATTCCTTCTATGGGACCTTTACTTTTTTATTTCACTTTTCTGAATTACCTGGTTTTACAACCTTTATCA
AGCAAACAGATCCTTCCAAGTATAAAAACTTATTGAACCTTTCTGCTTTATGGTGGGTGTATTTTAATTGTTCCCC
AAATTCATTTAATTCTATGAATCTTAATAATTGCTCAAGCGAAAAACGGTATCATTGGTTATTCAAACATCATTTG
GAACTGTCTGTGGGTGGTACAGGTTATGCCCTATAC
>cr20 .
TGGAGCAATCATCTCAGATGATGACCTAGGACTTCAGTTTCTCTTTGAATTGAGGAGAGAGACACATAAAAACAGT
GTATGCTTGGCCTTTGTACACATTATTGTGGAAGATAAAATATTATTCCAGAAAAATGCAAATATCTTTTATAATG
AGATCATAACATCATCAGCAAGAGTTGTTATCATTTATGGAGACAAAGACTCTCATCTTCAGCTCAACCTTAGACT
CTACAGATTAGTAAATGTTCAGAGGATCTGGGTCACTACCTCAGAGTGGGACTTAATCAAACATAATGAGAGATTC
CTTCTTGATTCCTTCTTTGGGACCTTTACTTTTTTGCCTCATTTTTCTGAATTATCTGGTTTTACAACATTTATTG
AGACAATAGATCCTTCCAAGTATACTAATCCATTTGCTCTTGCTAAATTATGGTGGATTTATTTTAATTGTTCCAC
AAATTCATTTAATTTAATGAAAATTAAGAATTCCTCAACAGACAAACTATATATATGGTTACTCAAGCACAAGTTT
GAAATGTCTGTGGGTGGTACAGGTTATGTCCTATAC
>cr21 .
TGGAGCCAGCATCTCAGATGATGACCTAGGACTTCAGTTTCTCTTTGAATTGAGGAGAGAGACACAAAGAAACAGT
GTATGCTTGGCCTTTGTACACATTATTGTGGAAGATAAAATATTATTCCAGAAAAATGTAAATACCTTTTATAATG
AGATCATAACATCATCAGCAAAAGTTGTTATCATTTATGGAGACAAAGACTCTCATCTTCAGCGCAACCTTAGACT
CTACAGATTAGTAAATGTTCAGAGGATCTGGGTCACTACCTCACAGTGGGATATGATCACACACGATGACAGGTTA
CTTTTTAATTCCTTCTATGGAACCTTTACTTTTTTACTTCATTTTTCTGAATTATCTGGTTTTACAGCATTTATCC
TAGCACCAGATCCTTCCAAGTATATTAGTACATGGATTCTTTCTGAATTGTGGTGGATTTATTTTAATTGTTCCCC
AAATTCATTTAATTCAATGAAAATTAAGAATTACTCAAAAGGAAAACTATATAATTGGTTATTCATGCAAAAGATG
GAAATGTCTGTGAGCAGTACTGGTTATGTCCTATAC
>cr22 .
GGGAACAATCATTTCGGATGATGACTTAGGACTTCAATTTCTCTTGGAATTGAGAAAAGAAATGCAAAGAAACAGT
GTGTGTTTATCCTTTGTGAGTATCATTACAAATGATATTACAGTATTCCTGAAAAATATAAATATATATTATAGCC
AGATCATGATGTCATCAGCAAAAGTTGTTATCATTTATGGAGATAAAGACTCTTACCTACAAGTGAACCTTAGAAT
ATGGAATTCAATGAAAATTAAGAGAATTTGGGTCACTACTGCCCAGTGGGATATGATCATACCTGAAGGAAAATTC
CTCCTTAACTCCTTCTATGGGACTCTCACTTTTATAAATCACTATTCTGAATTAACTGGTTTTAAAACATTTATCC

AGACAACACACCCTACCAACTACAGTAATGATATCTCTCTTGCTAGATTGTGGTGGATGTATTTTAATTGTTCTTT
GTCATCATTTAATTGCAAGAATCTGAAGAATTGTTCAACCAAAACAATATTAAACTGGTTATCCAAGAACCATTCT
GAAATTTCTTTGAGTGGTACAAGTTATGACCTACAT
>cr23 .
GGGAGTGATCATTCCAGATGAAGTCCATGGACATGAATTTCTTTCTGAATTGAGAGAGGAGATGCAAAATAAAATT
GTCTGTATAGCATTTGTGAGCATTATCACAAATGATGCTATATCATATACTAAAATGGCTGATACATATTATAACC
AGATCATGATGTCATCAGCAAAAGTTGTGATTGTTTATGGAGACAAAGACTCTCTAGTATGTCACTTTATACTATG
GAACTATGTGGAAATTCGGAGAATCTGGGTCAGTATGTCCCAATTGGATAGTATCACATTTATGGGAGATTTTATG
CTTAACCCGTTCTATGGGACTTTCATTTTCTCACATAAGAATCCTGAGATGTCTGGTTTTAAACAATTTATTCAGA
CAGTGCATCCTTCAAACTATAGTAATGAAATTTCACTTGCTAAACTATGGTGGATTTATTTTGAATGTTCTTTCTC
ATCATCTAATTGCACAAAACTTAAAGATTGTTCTACCAAAACTCTGTTGAAATGGTTCTATAGGCACCAGTTTGGA
ATGGCCATGAGTGATACAAGTTATAATTTATAC
>cr24 .
GGGACTGGTAATTTCAGATGATGACCCAGGTATTCAATTTCTTTCTGAATTGAAAGGAGAGATGCACAAACACAGA
GTCTGTCTAGCCTTTGTGAATATCATCACAGAAAATATACAATTACATCAGAAAACAGCTGAAAAGTACTATAACC
AGATCATGTTATCATCAGCAAAAGTTATTATCATTTATGGAGACACAGACTCCACTCTATCTGTATACTTTAGAAT
ATGGCAACATCTAGACATTCAGAGGCTCTGGATAACCACCTCACAGTGGGATATGAACACAAGTAAGGGAGATTTC
CTCCAAAGTCCCTTCCATGGGACTCTCATTTTATCACATCACCATTCTGAGATTTCTGGTTTTAAAAAATTCATAC
AGACAATTCACCCTTCCAACTACAATAAAGACACTTCTATTGCTAGGTTATGGTGGATGTATTTTAACTGTTCATT
GTCATCACATTGTAAGACACTGAAAAATTGTTCAACCAAAATCCTACTAGAATGGCTATCCAGGCACAATTTTGAA
GTGTCCATGAGAGAGACAAGTTACAACCTAGAC
>cr25 .
GGGGCTGGTGATCTCAGATGAAGAAAAGGGTGTTCACTTTGTCTCAGAATTGATACCAGAGATGGAAAGAAACAGA
GTCTGTGCTGCCTTCATAAAAATGATCCCAATCAGCCATTTATTCTCTATGTCATTCATTGACATGCACCATAGCC
CGGTTGTGATGAACTCAGCAAAAGTCATCATCATCTATGGTGACACTGACTCTTCTTTAGGTGTGTTCTTTAGAAG
ATGGGAATATGTATTTCCATGGAGAATCTGGATCACCACTTCACAATGGGAAGTCACTACTGCTATGAGACATTTC
ATTCTTGACTCATTCCATGGAACTCTCATCTTTTCACAACACCATGGTGAGATTTCTGCTTTTAAGGACTTTGAAA
AGACAGTTACCCCTTCCAAATACCCAGAAGACATTTTCCTTGCAAGACTGTGGGAGATGTATTTTGATTGTGCAAT
CTCTAATACTTCCTGTAAAGCATTAGAAAATTGTTCATCTAGAGGTTCCTTGGAATGGCTACCTTGGTACCACTTT
GACACCACCATGAGTGATGGCAGTTATCATGTGTAC
>cr26 .
GGGGCTGGTCATCTCAGAAGATGAAAAAGGCGTTCAGTTTGTCTCAGAACTGATACCAGAGATGGAAAAAAACAGA
GTCTGTGTAGCCTTCATGGAAATGATCCCAGTCATGCATACACATTACATTGTTCACCCTGATATAATTCAAAACA
AGATTGCTATCAACACAGCAAAAGTTATTATCTGTTATGGTGACACTGAGACTTCTTTAGGAATACTCTTCATAAT
ATGGGAACATGTACTCCCATGGAGAATCTGGGTCACCACTTCACAATGGGAAGTTATTACTGCTATGAAACATTTC
ATTCTTGACTCATTTCATGGGACTCTCATTTTTTCAAAACACCATGGTGAGATTTCTACTTTTAAAGATTTTGTAA
AGAAAGTCACCCCTTCCCAATACCCGGAAGACATTTTCCTTGCAAGACTGTGGGAGATTTATTTTAATTGTGCACT
TTCTCATTCTTCATGTACATCAGTAGAAAATTGTTCATCTAGAGGTTCCTTGGAATTGTTACCTTGGTACCACTTT
GACAAAGCCATGAGTGATAGCAGTTATCATGTATAC
>cr27 .
AGGACTGCTCCTCACAGATGACCACAGAGGAATGCAGATTTTATCTGACTTGAAAGAAGAGATGAAGAAAAATAGA
GTCTGTGTGGCTTTTGTGGAATTGATTTCAGGTACCTGGAATTCACTTTCCCACAAATTCTGGAAAAGTCTGGGAA
AGATTGAAGACTCATCTGCAAATGTGATTATCATTTTTGGAGACACTGATTCTCTTCAAGGTTTAATGAAAAATAT
AGGGCAGAAATTAACAACATGGAAAGTCTGGATTATGAACTCACAATGGGATGTTACCAACCACAATGATTATTTT
ATGTTAGATTCTTTCCATGGGAGTCTCACTTTTTCACACCATTATGAGGAGATGGTGGATTTTACAAATTTTATAC
AAACGGTTCACCCTTCCAAATACCCAGAAGATACATATCTTCCTAAATTGTGGCATTTGTTTTTCAAATGCCCATT
TTCTAAAATTAATTGTAAATTGCTGGATAATTGTCAACCCAATGCTTCCTTGGATTTATTGCCCAAACAAAATTTT
GACACAGCTATGAGCGAAGAGAGTTATTATATATAT
>cr28_ .
AGGACTGCTCCTCACAGATGACCACAGAGGAATGCAGATTTTATCTGACTTGAAAGAAGAGATGAAGAAAAATAGA
GTCTGTGTGGCTTTTGTGGAATTGATTTCAGGTACCTGGAATTCACTTTCCCACAAATTCTGGAAAAGTCTGGGAA
AGATTGAAGACTCATCTGCAAATGTGATTATCATTTTTGGAGACACTGATTCTCTTCAAGGTTTAATGAAAAATAT
AGGGCAGAAATTAACAACATGGAAAGTCTGGATTATGAACTCACAATGGGATGTTACCAACCACAATGATTATTTT
ATGTTAGATTCTTTCCATGGGAGTCTCACTTTTTCACACCATTATGAGGAGATGGTGGATTTTACAAATTTTATAC
AAACGGTTCACCCTTCCAAATACCCAGAAGATACATATCTTCCTAAATTGTGGCATTTGTTTTTCAAATGCCCATT
TTCTCAAACTGATTGTAAATTGTTGGAGAACTGTCAACCAAATGCTTCCTTGGATTTATTGCCTAAGCAAAATTTT
GACACAACCATGAGTGAAGAGAGTTACTATATATAT
>cr29 .
TGGTCTGCTTCTTCCAGATGACCACAGAGGGACTGAGTTTCTGTCAGACTTCAGAAAAAATATGGAGAAAAATACA
GTCTGTATAGCTTTTGTTGAAATGATCTCAAGCACATGGACTTCATTTTCCTACAAATTCTGGGCAAATTTGGGGA
AGATACAGGAATCATTGGCCAATGTCATCATCATTTATGGTGACAGTGATTCCCTACAAGGTTTAATGCTGAATCT
AGGAAAACACTTTTTGACATGGAAAGTCTGGGTCATGAACTCTCAATGGGATGTTACCAATCATGCTGATTATTTC

TTGGTAGATTCATTCCATGGAAGTCTCATTTTTTCACTTCAACATGAGCAAATGGCTGAATTTCCTAATTTTATCA
AAACAGTTAACCCATTCAATTACCCAGAAGACATTTACCTTCCTAAATTATGGGCTTTGTTCTTCCAATGCTCATT
TTCAGAGCTTGATTGTCAACTTGTGGAGAGATGCCAACCAAATGCTTCCTTGGAATTACTGCCTAGACACATTATT
GGTATGACAATGAGGGAAGAGAGTTATAATGTATAC
>cr30 .
TGGTCTGCTTCTTCCAGATGACCACAGAGGGACTGAGTTTCTATCAGATTTAAGAAAAAATATGGAGAAAAATACA
GTCTGTATAGCTTTTGTGGAAATGATCTCAAGCACATGGACTTCATTTTCCTACAAATTCTGGGCAAATTTGGGGA
AGATCCAGGAATCATTAGCCAATATCATCGTCATTTATGGTGACAGCGATTCCCTACAAGGTTTAATGCTGAATCT
AGGAAAACACTTTTTGACATGGAAAGTTTGGGTCATGAACTCTCAATGGGATGTTACCAACCATGCTGATTATTTC
CTGGTAGATTCATTCCATGGAAGTCTCATTTTTAAACATCAACATGAGCAGATGGCTGAATTTTCAAGATTTATCA
AAACAGTTAACCCATTCAATTACCCAGAAGACATTTACCTTCCTAAGTTATGGTTTTTGTTCTTCAAATGCTCATT
TTCAGAGCTTGATTGTCAACTTTTGGAGAGATGCCAAGCAAATGCTTCCTTGGAATTTCTTCCTAGACACATTTTT
GACATGGCAATGAGTGAAGAGAGTTATAATATATAC
>cr31 .
AGGTCTGCTTCTTCCAGATGACCACAGAGGAACTGAGTTTCTGTCAGATTTAAGAAAAAATATGGAGAAAAATAGT
GTCTGTATAGCTTTTGTGGAAATGATCTCAAGCACATGGACTTCATTTTCCCACAAATTCTGGGCACATATGGGGA
AAATACAGGAATCATTGGTGAATATCATCATTATTTTTGGTGACAGTGATTCACTACAAGGTTTGATGCTAAATCT
GGGGAACCAGTTTTTGACATGGAAAGTCTGGATCATGAACTCTCAATGGGATGTTACCAACCACGGTGATTATTTC
TTGCTAGATTCATTCCATGGAAGTCTCATTTTCTCACTTCAACATGAACAGTTGGTTGAATTTCCAAATTTTATCA
AAACAGTAAATCCTTTCAACTACCCAGAAGACATTTACCTTCCAAAGGTATGGTTTTTGTTCTTCAAATGCACATT
CTCAGAGATTGATTGTCAACTTTTGAAAACATGCCAACAAAATGCTTCCTTGGAATCCCTGCCTAGACACATTTTT
GACATGGCAATGAGTGAAGAGAGTTATAATATATAC
>cr32 .
TGGTCTGCTTCTTCCAGATGACCACAGAGGGACTGAGTTTCTGTCAGATTTAAGAAAAAATATGGAGAAAAATAGA
GTTTGCATAGCTTTTGTGGAAATGATCACAAGCACATGGACTTCATTTACCTACAAATTCTGGCCAAATTTTGGAA
AGATCCAAGAATCATTGGCCAATATCATCATCATTTATGGTGATAATGAGTCACTACAAGGATTAATGGTAAATAT
AGGAAAACAGTCTTTGACGAGGAAAGTCTGGGTCATGAACTCTCAATGGGATGCTACCAACCATGCCGATTATTTC
TTGCTAGATTCATTCCATGGAAGTCTCATTTTTTCACATCAATATGAAGAGATTGTTGGATTTCCAAATTTTATCA
AAACAGTTAATCCTTTCAAATACCCAGAAGACATTTACCTTCCTAAGTTATGGTTTTTGTTCTTCAAATGCTCATT
TTCAGAGCTTGATTGTCAACTTTCAGAAAAATGCCAACTCAATGCTTCCTTGGAATTTCTGCCTAGACACATTTTT
GACATGGCAATGAGTGAAGAAAGTTATAATATATAC
>cr33 .
TGGACTTTTTATGATAGATGATGACAAAGGTGCCCAGACTCTGTCAGATTTGAGAAGTGACCTGGAGAAAAATGGA
GTCTGCATAGCATTTGTGGAAATGATCCTGGTCACCAGGGGTTCGTTTCTCACCACATCCTGGAAAAATCAGGTGC
AGATCCTGGAATCAACGGCCAATGTGATTATTATTTATGGTGACTCTGATTCTCTACTAAGCTTGATTGTAAATAT
TAAACAGAAGTTATTTACATGGAAAGTCTGGGTCATGAACTCACAATGGGATGCTGCTAAATTTGATAACTATTTC
CTGTTAGATGTATTGCATGGTTCCCTTATTTTTTCACACCATAATGAGGAGATTATTAATTTCACAGATTTTATTA
AGACAGTCAATCCTTCCAAATACCCAGAAGACATTTATCTTCATGTACTGTGGCATTCATTCTTCAATTGCTCATT
TTTGAGGGAAGATTGTAAAATTGTGGATAACTGTCTGCCTAATGCCTCCTTGGAGTTCTTGCCAGGGAATATATTT
GACATGGCTATGAGTGAAGAGAGTTACAATGTGTAC
>cr34 .
GGGACTATTCATCATAGATGATGACAAAGGTGCCCAGATTCTGTCAGATTTGAAAGGCGGCCTGGAGAAGAACGGA
GTCTGTATAGCTTTTGTGGAAATGATCCTGGTCAGCAGGGGTTCATTTCTCACCACTTCCTGGAAAAATCAGGTGC
ATATCCTGGAATCAACGGCAAATGTGATTATTATTTATGGGGACACCGATTCTCTATTGAGCTTAATAGTAAATCT
AAAGCAGACGTTACTTATGTGGAAAGTCTGGGTCCTGAACTCACAATGGGATGCTGCTAAATTTGATAACTATTTC
CTGTTAGATGCATTGCATGGAGCGCTTATTTTTTCACACCATAATGAGGAGATTATTAATTTCACAGATTTTATTA
AGACAGCCAATCCTTCCAAATACCCAGAAGACATTTATCTTCATATACTGTGGCATTCATTCTTCAATTGCTCACT
TTTGAGGAAAGATTGTAAAATTGTGGATAACTGTCTGCCTAATGCCTCCTTGGAGTTCTTGCCAGGGAATATATTT
GACATGGCTATGAGTGAAGAGAGCTACAATGTGTAC
>cr35 .
CGGACTGTTCATCATGGATGATGACAAAGGTGCCCAGATTCTGTCAGATTTGAGAAGCGAGCTGGAGAAAATTGGA
GTCTGCCTAGCATTTGTGGAAATGACCCTGGTCAGCAGGGGTTCTTTTCTCACCACATCCTGGAAAAATCAGGTGC
AGATCCTGGAATCCTCAGCCAATGTGATTATTATTTATGGGGACACCGATTCTCTATTGAGCTTAATAGTAAATCT
AAAGCAGAAGTTACTTATGTGGAAAGTCTGGGTCCTGAACTCACAATGGGATGCTTCTAAATTTGATAACTATTTG
CTGTTAGATGCATTGCATGGAGCCCTTATTTTTTCACACCAGAATGAGGAGATTGTGAATTTCACAGATTTTATTA
AGACAGCCAATCCTTCCAAATACCCAGAAGACATTTATCTTCATGTATTGTGGCATTCATTCTTCAATTGCTCATT
TTTGAGGGAAGATTGCAAAATTGTGGATAACTGTCTGCCTAATGCCTCCTTGGAGTTCTTGCCAGGGAATATATTT
GACATGGCTATGAGTGAAGAGAGTTACAATGTGTAC
>cr36 .
CGGACTGTTCATCATGGATGATGACAAAGGTGCCCAGATTCTGTCAGATTTGAGAAGCGAGCTGGAGAAAATTGGA
GTCTGCATAGCATTTGTGGAAATGACCCTGGTCAGCAGGGGTTCTTTTCTCACCACATCCTGGAAAAATCAGGTGC
AGATCCTGAAATCCTCAGCCAATGTGATTATTATTTATGGGGACACCGATTCTCTATTGAGCTTAATAGTAAATCT

AAAGCAGAAGTTACTTATGTGGAAAGTCTGGGTCCTGAACTCACACTGGGACTATTCCAGCTTTGATAACTATTTG
CTGTTGGATGCATTGCATGGAGCCCTTATTTTTTCACACCATAATGAGAAGATTATCAATTTCACAAACTTTATAC
AGACAGCCAATCCTTCCAAATACCCAGAAGACACTTATCTTCATGTATTGTGGCATTCATTCTTCAATTGCTCATT
TTTGAGGAGAGATTGTAAAATTGCGGATAACTGTCTGCCGAATGCCTCCTTGGAGTTCTTGCCAGGGAATATATTT
GACATGGCCACAAGTGAAGAGAGTTACAATGTGTAC
>cr37 .
TGGGCTTTTTATTATAGATGATGACAAAGGTGCCCAGATTCTGTCAGATTTGAAAGGTGACCTGGAGAAAAATGGA
GTCTGCATAGCATTTGTGGAAATGATCCTGGTCACCAGGGGTTCGTTTCTCACCACATCCTGGAAAAATCAGGTGC
AGATCCTGGAATCAACGGCCAATGTGATTATTATTTATGGTGACTCTGATTCCCTACTGAGCTTAATTGTAAATAT
TAAGCAGAAGTTATTTACATGGAAAGTCTGGGTCCTGAACTCGCACTGGGATGTTGCTAAATTTGATAACTATTTC
CTGCTAGATGCATTGCATGGTTCCCTTATTTTTTCACACCATAAAGAGGAGATTATTAATTTCACAGATTTTATTA
AGACAGCCAATCCTTTCAAATACCCAGAAGACATTTATCTTCATGTACTGTGGCATTCATTCTTCAATTGCTCGTT
TTTGAGGAAAGATTGTAAAATTGTGGATAACTGTCTGCCTAATGCCTCCTTGGAGTTCTTGCCAGGGAATATATTT
GACATGGCTATGAGTGAAGAAAGTTACAATGTGTAC
>cr38 .
GGGACTGGTTCTCACAGATGATCACAAAGGGGCTGAGAGTCTCTCAGAATTGAGAGGGGAGATGGACAGCAACCAA
GTGTGCATAGCTTTTGTAGAAATGATTGCAGAGAGTGTGGATTCCTTTTACTTTACTTCTATGATAAGTATCCAGA
AGATCATAAAGTCAACTGCAAATGTGGTTATCATTTACGGTGACACTGAATCTTTAACTGGAATATTCCTATATAT
GGGGTACTTTTTGATGATTTGGAAAGTCTTGGTCATGAAGTCACAATGGGATCCAAGTATTGTTAGTTACTTTTTT
ATGCTTGATTCATTAGACGGGAGTCTCATTTTTACACATCATCATGATGAGATTCCTGAATTTACAAAGTTTATAC
AGACATACAACCCTTCCAAATACCCTGAAGACCATTATCTTGGTGTTTTTTGGGATAGACATTTCAATTGTCATTT
TTCTGGACTTGATTGTAAATTATCGGGTAACTGTAAACCCAATATTTCATTGGAAATGTTGCCTAGAGATATTTGG
GACATGGACATGACTGAAGAGAGTTACAATATATAC
>cr39 .
GGGGTTGATCCTCATAGATAACCACAAAGGTATTCAGATCCTATCAGACTTTAGAGAAGAGTTTCAGAGGAATAAA
ATATGTGTAGCATTCATAAAAATGATGTCAGAAAATATGATTTACTCAGACTATGTAGATGAAGCCACTCTTCGGC
TGATCCTGATGTCATCTGCAAATGTGGTTATCTTTTATGATGATACTGAAAGTGCTCATGGCATATTGCTACATCC
AATGTATTTGTTAAAAACATGGAAAGTCTGGGTCATGAATTCACAAATGGATATTGGTAGCATTGTAGATCCTTTT
GTGTTTGATTCATTTCATGGAACCATCTATTTTGTTCACCATCATGATGAGATCTCTGATTTTAGAAAGTTTATGC
AGACATACACCCCTTCTAAATACCCAGAAGATCATTATCTTGCACGTTTCTGGAATTTATACTTCAACTGCTCTTT
TTCTCTACCTGATTGTAAAATATCAGATAACTGCCTACCCAATGCTTCCTTGGAATTGTTACCTTCAAATATGTGG
AACATGGACATGACTGAAGAGGCTTACAATATGTAT
>cr40 .
GGGGTTGATCCTCATAGATAACCACAAAGGTATTCAGATTCTATCAGACTTTAGAGAAGAGTTTCACAGGAATAAA
ATATGTATAGCTTTCATAAAATTGATTTCAGAAAATATGATTTACTCAGACTATGTAGATGAAGCCACTCTTCGGC
TGATCCTGATGTCATCTGCTAATGTGGTTATCTTTTATGATGATAGTGAAAGTGCTCATGGTGTATTTCTATATCC
AATGTATGTTTTAAAAACATGGAAAATCTGGGTCATGACTTCACAAATGGATTTTGGTGGCATTGAGGATCCTGTT
GTGTTTGATTCATTTCATGGAACCATCTATTTTGTTCACCATCATGATGAGATTTCTGATTTTAGAAAGTTTATGC
AGACATACACCCCTTCTAAATACCCAGAAGATCATTATCTTGCTCTTTTATGGAGTATATACTTCAACTGCTCTTT
TTCCCTTCCTGACTGTAAAATATTGGGTAACTGCCTACCCAATGCTTCTTTGGAATTGTTACCTTCAAAAATGTGG
AACATGGAAATGACTGAAGAGGCTTACAATATATAT
>cr41 .
GGGCTTGTTCCTCGTAGACAACCAAAAAGGAGCTCATATTCTATCATATTTGACAGAGGAGTTTTCCAGGAATCGT
GTTTGTGTGGCTTTTGTGAAAATGATGCCTGAAAACCTACTTTCCTTTCACAGTGAACACAAAGGAAATATTGAGC
TGATGAGGTCAACATCTGTAAATGTAGTTTTCATCTATGATGACGCGGAATCTTTACATTGTTTAATGTTATATTC
TACATATATATTTTATACATGGAAAGTCTGGGTCATGAATTCACAATGTGATGTATCTAACAACGATGATGCTTTC
ATTTTTGATTTACTTCATGGGTCTCTATTTTATGCACACCACCATGATGAGATTTCTGATTTTAAAAAGTTCGTCC
AGACATATAGCCCTTCCAAATACCCAGAAGACCATCATCTTTTGTGTTTTTGGCATAAATTCTTCAATTGCTCTTT
ATCTCTCCCTGATGGTAAAATATCAGGTAACTGTTTACCCAATGCTTCTTTGGAATTGTTGCCTTCAAATCTCTGG
GAAATGGACATGACTGAAGAGACTTATAATATATAT
>cr43 .
GGGGTTGATTGTGTCTGAAGGACAAAAAGGTGTTGAGATAATCTCAGACTTGGGGGCAGAAATGGACAAAAACAGA
ATATGTGTAGATTTTGTGGAAATGCTCCCAGTCAGTGAAGTATCCTATTTAGCAAGCAGACAATTGATTCCTACAC
GGCTTCTGAAGTCATCAGCAAACGTGATCATTGCCTATGGTGACAGTGACTTTCTGAGAGGTTTCCTGTTTTATTT
AAAACACAGTTTAGTTACCATGAAAGTCTGGATCATGAACTCAGAATGGGATGTTTTCCTCCATTCAAAGCATTTC
ATTTTGCAGTCATTCCATGGGAGCCTCATTTTCACACATCACCACAAAGAAATCTCTGGTTTCAGAAACTTTATCC
AAACAGTTCACCCTTCCAAATACCCAGAAGATTTTTACCTTACACGGTTCTGGTTCTACTTTTTTAACTGCTCATT
TGCTGACGATGACTGTAATACACTGGAGAACTGTGCGCCTAATGTTTCCTTGCTTGAATCACCTAAGGATTCTTTT
GATATGGTCATGAGTGAGTACGCTTATAACACATAC
>cr44_PS .
AGGTCTTGTCATGTCAGATGATGATCAAGGAATTCAGTTTCTATCAGACTTGAGAGAAGAGATACAGATAAATGGA
GCCTGTTTAGCTTTTGTGAATGTGATCCCAGATAACATTCAGTTATATATGGAAAGGGCTATAATAAATGACAACC

AAATCATGACATCATCAGCAAAAGTTGCTATCATTTATGGTGAAATGAACTCTACCCTAGAAGTCAGCTTCAGAAG
ATGGACATATTTAGGTGTACAGAGAATCTGGGTCACTACCTCACAATGGGATGTCATCACAAGTAAGAGAGATTTT
AGACTTGACCCCTTTCATGAGACCATCACTTTTGCACACCACCATGATAAGGTTTCTAAATTTAGAAATTTTATGC
AAACAATGAACACTTCCAAATATCCAGTAGGTATTTCTCAGATGAGAACAAAGTGGAATTATTTTAATTGTTCAGT
CTCTAAGACCAACTATAGTTCAATGAATCATTGTCTATATAACACCACATTGGAATAGTTGTCACAACACAAGTTT
GACATGGACCTGAGTGAAGAAGGCTATAACTTATAC
>cr45_PS .
AGGGCTGGTCATCTCAGACAATGATCAGGGCACCCAATTTCTCTCAGATTTGAGAAGAGAGTTGGAAAAAAACACC
ATCTGTTTTGCCTTTGTAAATATGATTCCAATCAGCATGCAGCTGTACATGTCAAGAGCTGAAGTATATTATAACC
AAATCATGACATCATCCACAAAAGTTGTTATCATCTATGGTGACTCAGACAGTACTCTTGCTGTCAGCTTTCGAAT
GTGGGAATCCCGAGGCGTACAGAGAATATGGGTCATCACCTCACAGTGGGATGTGACTACAAGTAAAAGAGACTTC
ATGCTTGATTCATCACGTATGACTCTAGCTTTTGCACACCATCATGGTGAGATTTCTGGCTTCAAAAATTTTGTTC
AGACAATGAACCCTCTCAAGTACACAGATGAATATCTTGCAAGGCTGGAGTGGATGAACTTTAACTGTGAAGTCTC
CACATCCAACTGTAAGACACTGAAGAACTACTCATTGAATGCATCTATGGAATGGCTAGTGGTACAGACTTTTGAG
ATGGCCTTTAATGGTGGCCTTTATAACATCTAA
>cr46_PS .
GGGATTGGTCCTCATAGACGACCATAAAGGAACTCAGATTTTATAAGACTTGAGAGAAGAGTTGCATAGGAATAGA
ATTTGTGTAGCTTTTGTGACAATGATGTCAGAAAATATGATTTACTTGGCTGATGAACACAAAGTATTTGAGCTGA
TTGTGTTGTCATCTGCAAATGTGGTTGTTGTTTATGATAACACTGAATCTTTTCAGATCACTTTTTTCTGTATAAC
ATATTGGTTAAAATGGAAAATCTGGGTCCTAAATTCCCATTTGGGTATCTCTAGCAGTTATATTCCTTTCATTTTT
TATTTCATGGGACTCTCTTTTTTGCACAAAACCATGATGAGATTTCTGATTTTAGAAAATTTATCCAGACTTACAC
CCTTTCTAAATACCCAGAAGACCATTATCTTGCTCTGTTTTTGGAATACACACTTCGGTTGCTCTTTATCTGTACC
TGACTGTAAAATATCGGGTAACTATCTACCCAGTGCTTCATTGGAATTAGTTCCTTCAAATCAATGGGAAACGGAC
ATGTTTGATGAGAGTTACAATATATAC
>cr47_PS .
TGGAGCAATCATCTCAGATGATGACCTAGGACTGCAGTTTCTCTTTGAATTGAGGAGAGAGACACATAAAAACAGT
GTATGCTTGGCCTTTGTACACATTATTGTGGAAGATAAAATACTATTCCAGAAAAATGTAAATATATTTTATAATG
AGGTCATAACATCATCAGCAAAAGTTGTTATCATTTATGGAGACAAAGAATCTCATCTGCAGCTCAACTTTAGACT
CTACAGATTAGTTAATGTTCAGAGGATCTGGGTCACTACCTCAGAGTGGGACTTGATCACACATAATGAGATATTC
CTTCTTAATTCCTTCTATGGCACCTTTTCTTTTTTACCTCATTTTTCTGAATTATCTGGTTTTACAACATTTATCA
AGACAATAGATCCTTCCAAGTATACTAATCCATTTGCTTTTGGTACATTATGGTGGATTTATTTTAATTGTTCCCC
AAATTCATTTGATTTAATGAAAATTAAGAATTACTCAAAAGAACAACTATATATATGGTTACTCAAGCACAAGTTT
GAAATGTCTGTGGGTGGTACAGGTTATGTCCTATAC
>cr48_PS .
GGGACTGCTCATCACAAGTGACCACACAGGTGCTCAGATTCTATCAGACATGAGAAGGGAGCTCGACAGGAATGGA
GTCTGCATAGCTTTTGTGGAAACAATACCAATTTTGGGAGCGTCAGGATATTACAATTCCGTTCATACTGTGGTGC
ATATTCAGGAATCATCATCAAATGTGATTGTAATTTATGGAGACATTACTTCTTTATTAACTGTAATGGTAAATAA
ATGGGGAAAGTTCTTATCTAGGAAGGTTTGGGTCATGAACTCAAAATGGGGTGGTAGCAGGTTGCATGAATATACC
ATGTTAGATTCATTCCATGGGAGTTTCATTTTTTCTCCCCATCACGGGGAGATTTTGGGTTTCCCAAAATTCATGC
AGGAAGCCACCCCTATAAAATACCCAGAAGACATTTATCTTCATGTATTTTGGAACATATATTTCAATTGCTCACT
TTTGCCTTCTAATTGTGAAATCTTTGAAAACTGTCTGCCCAATGCCTCTTTGGAATTGTTGCCAGGGAACATTTTT
GACATGGCCATGAGTGAAGAGAGTTACAATGTAAC
>cr49_PS .
GGGAGTGATCATTTCAAATGAAGACAGTGGAACTGAATTTCTTTCTGAATTAAGAGAGGAGATGCAAAGGAATATT
GTCTGTTTAGCATTTGTGAGTATTATCACAATTGATGCCATATCATATTTTAAAATGGCCGATAATTATTATAACC
AGATCATGATGTCATCAGCAGAAGTTGTGATTGTTTATGGAGACAAAGATACTCAGCTATAATGGAACTTTATACT
ATGGAATTATGTAGAAATTCGGAGAATCTGGGTCAGTGTGTCACAACTGGATAGGATCACAGTTATGGGTGATTTT
TTCCTTAACTCCTTCTATGGGACTTTTATTTTTTTCACATAAGTATACTCAGATGTCTGGTTTTAAACAATTTTTT
CAAACAGTGCATCCTTCAAACTATAATGATGAAATTTCACTTGGTAAATTATGGTGGATTTATTTTGATTGTTCTT
TGTCTTCATCTAATTGTACGAAACTTAAGGATTGTTCTACCAAAACTCTATTGAAATGGTTATACAGGAACCAGTT
TGGAATGGCCATGAGTGATATAAGTTTTACCTTGTAC
>cr50_PS .
GATGGTCATGACAGATGATGAAAAGGGTATTGAATTCCTATCAGATATGAGGAAAGAGATACAAATTAACAGTCTG
TTTAGATTTGGGAACATGATTCTGGGAATCATATTTATACAGTTATATAAGAAACAAGGATATATGTATCATTATG
TAGTTGTGATAACATCAGCAATATTGTTATCCTATATGATGACATTAATTCCCTTCCAGCTTTAAACTTTCTAATT
TGGAAAACTTTAGGCACATAGAAAACCAGCGTCACCATCTCACAATTGGATATTATCACAAATAATAGCAAAGTCA
TCTCTGATCCATTCTGTGGGTCTTTTTCACACAGCCATTGTAAACTTTGTGGATTTAAATCTTTTATTCAGACAGT
GAAGCCTTGCAGATACCAAGATCATATTTCATTTGGAATACTGGGGTGACTGAATTTCAATTGTGTTGCCTCTAAA
TCTTCCTATGACACACTGAGAAATTATAATCCAACAACTCATTGGGATGGTGAAGTGGTCATAGTTTTTGGTGTAG
TCATGAATATGCAAGTTACAATACATAC
>cr51_PS .

GAATCTGGTCACAACAGATAATGATAAAAAGCATTAAATTCCTGGCAGAGATAGGAAAAGAGAAGCAAATTATCAG
AGCCTCTTTAGCCTTTATGAATGTGATTCTAAGACTCACATTTATAGGGTTACACAAGAAACTGGAATATATGCAT
TATTATCTAGGTATGATGTCATCAGCAAGAGTTGTTATCATATATGATGACACTAATTCTCTTCTAGCTTTGAACT
TCTAATTTTGAAAAATGCAGACACACAGAAAACCTGGGTCACCAACTCACAATTGGATGATATTACAAATTATAAA
ATATTATCTCTGACTCAGTCTGTGGGACTTTCATTTATAGACTTAAAACTCTTACTCATACCTTGAAGCTTTGTAA
ATATCCAGAACATATATATGCCCTTTGGAACACTGGGTGGCTGGATTTTAATTGTTTAGTCTCTAATTCTTCCTAT
AACACACTGAGAAATTGTACATCCAACAATTCATTGGAATGGTTAAGTGGTCACAGTTTTGATATGGCCATGAGTA
ATTCAAGTTACAAC
>cr52_PS .
TGGAGCAATCATCTCAGATGATGACCTCGGTTTCTCTTTGAATTGAGGAGAGAGACACATAAAAACAGTGTATGCT
TGGCCTTTGTACACATTATTGTGGAACATAAAATATTATTCCAGAAAAGCGTAAATGTATTTTATAATGAGATCAT
AACATCATCAGCAAGAGTTGTTATCATTTATGGAGACAAAGACTCTCATTTGCAGCTCAACCTTAGACTCTACAGA
TTAGTAAATGTTCAGAGGATCTGGATCAACAGAAAAACTATACTCTTGGGTATTCAAGCACAAGTTATAAATGTCT
GTGGGTGGTACAGGCTATGTCCTATACAATACTGTGCATGCTGTGGCCCATGCACTCCATGAGATGCTGCTACAAG
AAGTGGATACATGGCCAAAGTATTCTGGGGAAAGGTTGGAATTTGACTCTTGGCAGGTAATAAGTATTCCAAATGA
ATGCAATGTAGTGACAAAATATACTTTTCCCAATAGATAATGTTGTTTCTATGTAAGAACACCAGGATCATTTCTA
GCCTACACAGAATTTATATATAGG
>cr53_PS .
ACAATTACTGGGTCCAGGAAGAAATAAAAAAAAGAAATTAATGATTTCCTAGAATTGAATGAGAATGTGGAGACAA
CATATCCAAACCTATGGGACACTTTGAAAGCAGTGCTAAGAATGGAGACATCACATCCAAACCTATGGGACACTTT
GAAAGCAGTGCTAAGAACAAAGTTCATATGTTGTTATCATTTATGGTGACTCAGACAGTACTCTTGCTATGAGCTT
TCAAATGTGGGAATCCCGAGGTTTACAGAGATTATGGGTCATCACCTCACAGTGGGAGGGGACTGTATGACTCTAG
CATTTGCACACCATCATGGTGAGATTTCTGGCTTTAAAAATTTTGTCCAGACAATGAATCCTATAAAATACACAGA
TGAGTACCTTGCAACGATGGATTGGATGAGCTTCAACTGTGCAGCCTCCACATCCAACTATAAGACACTAATGAAT
AACTCCATGGAATG
>cr59_PS .
GGGAACAATCATTTCGGATGATGACTTAGGACTTCAATTTCTCTTGGAATTGAGAAAAGAAATGCAAAGAAACACT
GTGTGTTTATCCATTGTGAGTATCATTACAAACGATCCTACAGTATTCCTGAAAAATATAAATATATATTATAGCC
AGATCATGATGTCATCAGCAAAAGTTGTTATCATTTATGGAGATAAAGACTCTTACCTACAAGTGAACCTTAGAAT
ATGGAATTCAATGAACATTCAGAGGATTTGGGTCACTACTGCCCAGTGGGATATGATCATACATGAAGGAAAATTA
TGAACCTTAGAATATGGAATTCAATGAAAATTAAGAGAATTTGGGTCACTACTGCCCAGTGGGATATGATCATACC
TGAAGGAAAATTCCTCCTTAACTCCTTCTATGGGACTCTCACTTTTATACATCACTATTCTGAATTAACTGGCTTT
AAAACATTTATCCAGACAACACACCCTACCAACTACAGTAATGATATCTCTCTTGCTAGATTGTGGTGGATGTATT
TTAATTGTTCTTTGTCATCATTTAATTGCAAGAATCTGAAGAATTGTTCAACCAAAACAATATTAAACTGGTTATC
CAAGAACCATTCTGAAATTTCTTTGAGTGGTACAAGTTATGACCTACAT
>cr60_PS .
AGGGTTGGTCATCTCAGACAACGATCATGCTAGCCACTTTTTATCGTATGTGAGAAGAGCATGTGAAAAAAATACA
GTCTGCCTTGCCTTTGTTAATATGATTCCAATCAACATGCAGTTATACATGTCCAGAGCTTAATTGTATTATAACC
GAATCAAGGTATCATCCACAAAAGTTGTTATCATTTATGGTGACTCAGACACTACTTTTGCTGCGAGCTTTCAAAT
GTGGAAATCCCGAGGTTTACAGAAAATATGGGTCATCACCTCACAGTGGGATGTGACTACAAATAAAAGAGACTTC
ATGCTTGACTCATCACATATGACTCTAGCTTTTACACAACATCATGGTGAGATTTCTGGCTTTAAAAATTTTGTCC
AGACAATGAACCCTCTCAAGTATACAGATGAATACCTTGCAAGGCTGGAGTGGATGAACTTTAAATGTGAAGTCTC
CACATACAACTGTAAGACATTGAGGAACTACTCATCAGATGTCTCAATGGAATGGCTAGTGATTCGGAC
>cr61_PS .
AGGCTTGATCCTCATAGACAACCAAAAAGGAATTCAGATTCTATCAGACTTGAGAGAAGAGTTGCACAGGAATAGA
GTTTGTGTGGCTTTTGTGAAAATGATGTCAGAAAACCTAATATATTTTGACAGTAAAAACAAAGAAAATTTTGAGC
TGATGATGTCAATATCTGTAAATGTAGTTATCATCTATGATGACAAAGAATCTTTACATTGCATAATGGTATATTT
AATATATGGGTTTTATACATGTAAAGTCTGGGTCATGAATTCACAATGTGATTTATCTCAAGTTAATGACCCACTC
ATTTTTGATTCACTTCATGGGACTCTATTTTATGAACACCACCATGATGAGATTTCTGATTTTAAAAAGTTTGTCC
AGATATATAGCCCTTCCAAATATCCAGAAGACCATTATCTTTCTTTGTTTTGGAATAAATTCTTCAATTGCTCTTT
TTCTCTGATTGTAAAATATCAGGTAACTGTCTACCCAATGCTTCTTTGGAATTGTTGCCTTCAAATCAATGGGAAA
TGGACATGACTGAAGAGAGTTATAATATATAT
>cr62_PS .
GGGGTTGATTGTGTCTGAAGGACAAAAAGGTGTTGAGATAATCTCAGACTTGGGGGCAGAAATGGACAAAAACAGA
ATATGTGTAGATTTTGTGGAAATGCTCCCAGTCAGTGAAGTATCCTATTTAGCAAGCAGACAACTGATTCCTACAC
GGCTTCTGAAGTCATCAGCAAATGTGATCATCGCCTATGGTGACAGTGACTTTCTGAGAGGTTTCCTGTTTTATTT
AAAACACAGTTTAGTTACCATGAAAGTCTGGATCATGAACTCAGAATGGGATGTTTTCCTCCATTCAAAGCATTTC
ATTTTGCAGTCATTCCATGGGAGCCTCATTTTCACACATCACCACAAAGAAATCTCTGATTTCAAAAATTTTATCC
AAACAGTTCACCCTTCCAAATACCCAGAAGATTTTTACCTTACACGGTTCTGGTTCTACTTTTTTAACTGCTCATT
TGCTGACGATGACTGTAATACACTGGAGAACTGTG
>cr65_PS .

GGGGGTGGTCCTCATAGATAACCATAAGGGACATGAGATTCTATCAGACATGAGAGGAAAGATGTATAGGAATAGA
GTGTGTGTAGCTTTTGTAAAAATGATGCCAGAGACCATTGATTTTTTTTTGGACCATTCTTTCCAGGACAGTAGAA
AGGTGATTGAGAAATCATTTGCAAATGTGGTTATCATTTATGATGACAGGGAATTTTTACATTGTATAATGCTAGC
TGGACTGTCTTCTGAAAAAACGTGGAAAATCTGGGTCATGAATTCAAAATTCAATGTCAATACCAGTTATGAACCT
TTTATTTTTTGATTCATTTCATGGGTCTCTCCTTTTTACATATCACCATGATGAGATTTCTGATTTTAGACAGTTT
ATTCAGACATATACCCCTTCTACATACCCAGACAATCATTATCTTGATATTTTTTGGAAAACTTATTTCAATTGCT
CTTTCTCTCTACATGATTGTAAAATATCTGGTAACTGCCTACCAAATGCTTCATTGGAAATGTTTCCTTTACATAT
ATGGGAAATGCACATGACTGAAGCATGCTACAATGTATGT
>cr66_PS .
GGGGTTGATCCTCATAGATAACCACAAAGGTATTCAGATTCTATCAGACTTTAGAGAAGAGTTTCACAAGAATAAA
ATATGTATAGCATTCATAAAAATGATGTCAGAAAATATGATTTACACAGACTATATAGAGGAAGCAACTCTTCGGC
TGATCCTGATGTCATCTGCAAATGTGGTTATCTTTTATGATGATAGTGAAAGTGCTCATGGTATATTTCTACATCC
AATGTATTTGTTAAAAACATGGAAAATCTGGGTCATGCATTTACAAATGGATTTTGGTGGCATTGAGGATCCTGTT
GTGTTTGATTCATTTCATGGAATTATCTATTTTGTCCACCATCATGATGAGATCTCTGATTTTAGGAAGTTTATGC
AGACATACACCCCTTCTAAATACCCAGAAGATCATTCTCTTGCTCATTTATGGAATTCATTCTTCAACTGCTCTTT
TTCTCTACCTGATTGTAAAATATCGGGTAACTGACTACCCAATGCTTCGTTGGAATTGTTACCTTCAAAAATGTGG
A
>cr67_PS .
GGGGTTGGTTCTCACAGATGACCACAAGGCATCTCAGATTCTATCTGATTTGAGAGAGAAATGGACAGAAATAAAG
TGTGCATAGCTTTTGCAAAAATGATTCCAGACACTCACTTTCCCACTCTTGACTCTATCATGAAAACTCGTGTGCA
GATCCTGGAATCATTGACAAATGTGATTATCATTTATGGTGACTGTGATTCCCTAAAATTTTTAATAGTGAGTTTA
TCACATCCTTTACTGACAATGAAAGTCTGGGTCTTGAAGTCACGATTACATACTTCTTCATATTTTCATTCACTTG
AGTTCCTGGCTGACTCATTCCACGCAGGTCTCATTTTTTCACATCACCTTGCAAAAATTCCTGGTTTTAAACATTT
CATGCAGACAGTTAATCCTTTCAAATATCCAGAAGACAATTTCCTAGCTATTTTGTGGCATAGACACTTCAATTGT
TCTTTTTCTCAACACGATTGTAAAATTCTGGGTATCTGTCAACCCAATGCTTCCCTGGAACAGTTGCCTATGAAAA
ATTGGGAAATGGGCATGACTGAAGAGAGTTATAATATATAT
>cr68_PS .
GGGACTGGTATTCACAGATGACCACAAAGGGGCTTACATTCTATCAGACTTGAGAACAGACATGGACAGGAACAAA
GTGTGTGTTGCTTTAGTGGAAATATTACAAGCATCTTGGTTTCCTTTGCCTACAATGCTCACTTGCAAATCATGAA
ATCATCTGCAAATGTGGTTATATTTTTGGTGAAGCTGAATCATTATATGGTATACTTATAAATATAGCAAATCACT
TATTGAATTGGAAAGTCTGAGTCATGACATCACAATTGGATGCAAATGCTGCTTCTAAATCTTTGATATTTGATCA
TTTCCATGGGAGTCTTACTTTTGCACATCAACGTCCTGAGATTTCTGATTTTAGGAAGTTTATCCAGACATACTAC
CCTTCCAAATACCCAAAAGACCATTTTCTTGCTCTGTTGTGGAACTCATACATCAATTGCTGTTTTTCCTCACCTG
ATTGTAAATTTTAGATAAATGTCTACCCAATGTTTCCTTGTAAATATTGCCTAGAAATGTTTAGGACATGGACATG
TCTGAAGATAGTTACAATCTATAT
>cr70_PS .
GAAGCTGCTCTTAGTAGACCATAGGGGTGCCCAGATTCTAGAAGATTTGAAAAGAGAAGTGGACAGAAACAGAGTC
TGCATTGCCTTTGTTACAATGATTCCAGCCAATGGAGGTTTATTTCTCAAAGAATCCTGGAAAAGTACAGGGCAGA
TGCGGGAATCATCAGGCAATGTGTTTGTCATTTGTGGTGGCGCTGATTATTAACTTTAAATATAAGTGTAAGGCAA
ACATTACATTGAGTTCTGGGTCATCAACTCACACTGGGATAATTGCAATGAAGAGAAATATTTTATGTTAGACTCA
TTGCCTCAGGCACTCACTTTTTCACACCACACATTTTATAAAGTCAGCTAACCCTTCCAAATATCCAGAAGACATT
CATCTTCAAGGATTGTGGTGTTCACCTTTCAACTGCCCATATTTGAGCAAGGATTATAGAATTTTAGATAATTGTT
TGCCCAATGCCTCATTGGGGTTCTTGCCTGGGAATATTTTGACAAGGCCATGAGTGAAGAGAGTTCCAGTGTGAGC
>cr72_PS .
GGGGCTCCTTGTGGCTGATGACCTGAAAGGAAAGGAGTTTCTCTCTGACCTGAAAGCAGAGATTGCATCAGAAGAT
ATCTGTGTGGCTTATACAGAAAAACTCCCAACTAATTGCAAAATTCAGTTAATGTCTGGATTTGGGTTGGTGAATT
TGAAACAACATATGCAAGTAAATTTGTATATATTCTGTGTTGATACAGATGACTTGCTGATTTGTTACCTGGACAA
TGAAATCATGTTAAGCAGAAGCAAGGTGTGATCATGGCAAAGCCAAAATTAGTTTACTTAGAGTCTATATTATATG
GGTGGAATGAATTGATGGGCTGTTTTAAAGGAAGCTTCTCATTTTCAAAGGAGAGAAATATCCCTGGCTTCAAATA
CTTTATTGAGGCACTTACTCCCTACCTGTACCCAGGTGACATTTACTTCCATAAATTCTGGCTGGACAATTTTTAA
TTGTTCACTTTCTCCTTTGCTATTTGGAAATCACAAACGCTGTCCATGAATATGTCACAGTCACTAACTCTTACCT
CTACATCAGACTGAAAGGTGATCCTGGCTCTACCAAACCT
>cr73_PS .
GGGGCTCATTGTGGCTGATGACCTGAAAGGAAAGGAGTTTCTCTCTGACCTGAAAGCAGAGATTGCATCGGAAGAT
ATCTGTGTGGCTTATACAGAAAAACTCCCAACTAATTGCAAAATTCAGTTAATGTCTGGATTTGGGTTGGTGAATT
TGAAACAACATATGCAAGTAAATGTGTTTATATTCTGTGTTGATACAGATGACTTGCTGATTTGCTACCTGGACTA
TGAAATCATGTAGCAGAAGCAAGGTATGGATCATGGCAAAGCCAAACTTAGTTTACTTAGAGCTGTATTATATGGG
TGGAATGAATTGATGGACTGTTTTAAAGGAAGCTTCTCATTTTCAAAGGAGAGAAATATCCCTGGCTTCAAACACT
TTATTGAGACACTTACTACCTACCTGTACCCAGGTGGCACTTACTTCCATAAATTCTGGCTGGACAATTTTAATTG
TTCATTTCCTCCTTTGCTATTTGGAAATCACAAACCCTGTCCATGAATATGTCACAGTCACTAACTCTTACCTCTA
CATCAGACTGAAAGGTGATCCTTGCTCTACCAAACCT
>cr74_PS .

GTGGCTCCTTGTGGCTGATGACCTGAAAGGAAAGGAGTTTCTCTCTGACCTGAAAGCAGAGATTGCATCAGAAGAT
ATCTGTGTGGCTTATACAGAAAAACTCCCAACTAATTGCAAAATTCAGTTAATGTCTGGAGTTGGGTTGGTGAATT
TGAAACAACATATGCAAGTAAATTTGTATATATTCTGTGTTGATACAGATGACTTGCTGATTTGCTACCTGGACAA
TAAAAATCATGTTAAGCAGAAGCAAGGTGTGGATCATGGCAAAGCCAAAATTAGTTAACTTAGAGTCTATATTATA
TGAGTGGAATGAATTGATGGTTTTAAAGGAAGCTTCTCATTTTCAAAGGAGAGAGATATCCCTGGCTTCAAACACT
TTATTGAGACACTTACTCCCTACCTGTACCCAGGTGACATTTACTTCCATAAATTCTGGCTGGACAATTTTTAATT
GTTCACTTCCTCCTTTGCTATTTGGAAATCACAAACGCTGTCCATGTATATGTCACAGTCACTAACTCTTACCTCT
ACATCAGACTGATAGGTGATCCTGGCTCTACCAAACCT
>cr83_PS .
AGGGCTGGTCATCTCAGACAATGATCAGGGCACCCAATTTCTCTCATATTTGAGAAGAGAGTTAGGAAAAAGTACA
GTCTGTCTTGCCTTTGTAAATATGATTCCAATCAACATGAAGTTATGCATGTCAAGAGCTGAATTGTATTATAACC
AAATAGAGACATCATCCACAAATGTCGTTATCATTTATGGTGACCCAGACAGTACTCTTGCTGTGAGCTTTCGAAT
GTGGCAATCCAGAGGTTTACAGAGATTATGGGTCATTGATTCACAATGGGATGTGATTACAAGTAAAAGGGACTTC
ATGCTTGACTCATCACATATGACTCTAGCCTTTGCCCACCATCATGGTGAGATTTCTGGCTTTAAAAATTTTGTCC
AGACAATGAACCCTCTCAAATACACAGATGAATACCTTGCAAGGCTGGAGTGGATGAACTTTAAATGTGAAGTCTC
CACATCCAACTGTAAGATGGAATGGCTAGTGATTCGGACTTTTGACATGGCCTTTAATGATGGCTTTTATGACATA
TACAATGCTGTGTATGCTCTGGCCCATGCAC
>cr84_PS .
GGGGCTGTATATCTCAGAAGATGAAAGAAGCATTCAGTTTTGGTATGACTTTAGAACACAAGAACAGTATCTGTGT
GGACTTAATGGAAATTGTGCCAACCAGCAAATTGTCAGCATCTCCAGATTTTAAATTGGGCCATTATCATCAGATC
TTGAAATCATTCGTTAATGTTATTATACTCTATTTTGACTCTGATTATCTGATCACCCTATTTTATGTAATAGACC
ATTCTCTAATGACACAAAAAGTCTGGCTCATGGCATCACCACATCATTCTTCCAGAATAAAACAGAGTATACTCTT
TGACCCATTTCATGATTCTCTCATGTTTTCACACCACCATAAGGAAATTTCAGGTTTTAGAAAATTTATCCATACT
GTCAATCCTTCTAAATACCCAAATGACATTTACTTAGCTATACTCTGGTCTTACTTTTTTGATTGTTCATTTTCTG
ATTCTGACTGTACAATACTGGAGAACTGTCCACTCAATGCTTCCTTGGAAATGTTACCTTATCCAGTTATTGACAT
GGCCATAAGTGAGGAGAGTTACAACCTATAC
>cr85_PS .
GGGGCTCATTGTGGCTGATGACATACATGGAAGGGAGTTCCTCTCTGACCTGACAGCAGAGCTTGCTTCAGAAGAT
ATCTGTGTGGCTTTTACAGAAAAAATACCAACTTTTATGAAAGTGGATTTTTACTTTGGGGTTGGGTTGGTGAATT
TGAGCCAACATACAAAAGTAAATGTGCGTGTGTTCTATGGGGATATAGATGATGTCCTGATTTTCTACATACAAAA
TGAAATTATATCAAAGAGAAAGAAGATGTGGATCATGGCAAAGCCCAACTTTGTTTACTTACAATCTGTATTTTAT
AAGTGGATTGGTTTGATGCCCAGCGTTTCCATATGGAATGCAGTGTATGCAGTGGCCCATGCCCTCCATGAAATGC
TTTTGAGTAAAACAGAAATAGATTCTCACAAAGACATAAACCAGGACAGGCTTCTACCTTGGCAGGTAAGTCTACC
TCATAAAGAAAGGAGACACTAGGAACTTACATCATTAGTAGGTACTTCACTGAGTTCAAACACACAACACACTTTC
ATATTGTAATGAAAAGAATAATGCAAAATGTTGCTTACTTCTAGGGT
>mr1 .
GGGAGTAATCATTTCAGATGATGACAATGGAATTCAATTTCTTTCTGAGTTGAGAGAAGAAATGCAAATATCTGTT
GTCTGTTTAGAATTTGTGTCTATTATCACAAACGATATTGACTTGCATTTAAAAATGTTTCCTAAGATTTTAAATA
CAATCTTATTGTCAACAGCAAAAGTTATATTTGTTTATGGAGACAAAGACGCTATCATACATCCACACTTTCTTGC
ATGGAATCATATTCTCTTCAGTAAGATATGGATCAGTATGTCACAATTTGATATAATAACAATAGATGGTGATTTC
TTGCTTAAACCTACCTATGGGACTCTAATTTTTTCACACCATCATTCTGAGATGTCAGGCTTTAAAAAATTTATGC
GGACAGTGCACCCTTCAAACTACAGTAATGATATCTCCTTTGCTAGACAATGGTGGATTTGTTTTAACTATTCTTT
GCCACCATTTAATTGTGAGAAACTGAAGAATTGTCCACTTGACACCCTATTTAAGTGGTTATTCAGGCCACCACTT
GAAATGTCGATGAGTGATACATCTTATAACTTGTAC
>mr2 .
TGGTCTAATCCTCCCAGATGACCACAGAGGAACTCAGATACTATCAGACTTGAGAGAAGATATGGAGAGAAATAGC
ATCTGCATAGGTTTTTTGAAAATGATCGCTGGCATGTGGAATTCATTTTCCAATGAGTTATGGAGAAATCTGGAGA
ATATTCAGGAATCATCAGCAAACGTGATTGTTATGTATGGGGACACTGTTTCTTTGCAAGGTTTAATGAGACACAT
TGCACAACTCTTAGTGACATGGAAAGTCTGGGTCTTGAATTCTCCATGGGATGCTGACCACTATTCTGATTATTTC
ATGGTAGAGTCATTTCATGGGAGTTTCATTTTTTCACATCACCATGAAGAGATGGTTGAGTATATGAATTTTATCC
AAACAGTTAATCCATACAAATACCCAGAAGACAACTATCTTCCTAAGTTTTGGTATTTGTTCTTCAAGTGCTCATT
CTCTGAGTCTGATCGTAGACTTTTAGAGAACTGCCAACCCAATGCTTCTTTGGACTTACTGCCCAGACACCTTTTT
GACCCAGCTTTGAGTAATGAGGGCTACAATATATAC
>mr3_PS .
TGGTCTAATCCTCCCAGATGACCACAGAGGAACTCAGATTTTATCAGACTTGAAAGAAGATATGGAGAGAAATAGC
ATCTGCATAGGTTTTTTGAAAATGATCCCTGGCATGTGGAATTCATTTTCCAATGAATTATGGAGAAACCTGGAGA
ATATTCAGGAATCATCAGCAAACATGATTGTTATGTATGGGGACACTGTTTCTTTGCAAGGTTTAATGAGACACAT
TGGACAACTCTTAGTGACATGGAAAGTCTGGGTCTTGAATTCTCCATGGGATGCTGACCACTATTCTGATTATTTC
ATGGTAGAGTAATTTCATGGGAGTTTCATTTTTTCACATCACCATGAAGAGATGGTTGAGTATATGAATTTTATCC
AAACAGTTAATCCATACAAATACCCAGAAGACAACTATCTTCCTAAGTTTTGGTATTTGTTCTTCAAGTGCTCATT
CTCTGAGTCTGATTGTAGACTTTTAGAGAACTGCCAACCCAATGCTTCTTTGGACTTACTGCCCAGACACCTTTTT
GACCCAGCTTTGAGTAATGAGGGCTACAATATATAC

>mr4_PS .
TGGTCTGATTCTCCCAGATGACCACAGAGGGAATCAGATGCTGTCAGACTTGAGAAAAGATACGGAGAGTAATGGC
ATCTGCATAGCCTTTATGAAATTGATCTCTGGCACCATGAATTCACTTTCCAATGAATTATGGAAAAATCTGGAGG
AGATTCAGGAATCCTCAGTAAATGTGATAGTTATATATGGGGACATTGGTTCTTTACAAGGTTTAATGTGACACAT
TGGGCACCTGTTTTTGACATGGAAAGTCTGGGTCTTAAACTCTCAGTGGGATGCTGTCAGCCATGCTGATTATTTC
ATGGTAGACTCATTTCATGGGAGTCTCCTTTTTACACACCATCATAAAGAGATGGTTGAGTTTACAAATTTTATTC
AGTTAATCCCTACAAATACCCTGAAGACACTTATCTTCCTAAGTTTTGGTATTTGTTCTTCAAGTGCTCATTTTCT
GAGCTTGACTGTCAACTTTTGGAAGACTGCCAACCCAATGCTTCTTTGGATTTCCTGACCAAAAATATTTTTGACG
CAGCCCTGAGTGAAGAGAGCTACAATATATAC
>mr6 .
TGGTCTTATCCTCCCAGATGACCACAGAGGGATCCAGATTCTGTCAGACTTGCAAGAAGATATGGAGAGTAACAAT
ATCTGCTTAGGCTTTTTGAAAATGATCCCTATTACCTGGAATGCACATTCCAGTGCATTATGGAAAGATCTGATAA
AGATTCAAGAATCATCAACTAATGTGGTAGTTATTTTTGGGGACCTTGTTTCTTTGCAAGGTTTAATGCGACTTAT
TGGGGAATTGTTAGTGACATGTAAAGTCTGGATCTTAAACTCTCAATGGGATGTTAGTTACAATTTTGATTATTTC
ATGTTAGAGTCATTCCATGGAAGCCTCATTTTTTCACACCACCATGAAGAGATGGTTGACTTTACAAATTTTGTTC
AAACAGTTAATCCCTACAAATACCCAGAAGACACTTATCTTCCTAAGTTTTGGTTTCTGTTTTTCAAGTGCTCATT
TTCTGAGTCTGATTGTCAACTTTTGGAAAACTGCCAACCCAATGCTTCTTTGGACTTACTGCCCAGACACCTTTTT
GACCCTGTCATAAGTGAAGAGAGCTACAATATATAC
>mr7 .
TGGTCTGATCCTCCCAGATGACCACAGAGGGATCCAGATTCTGTCAGACTTGCAAGAAGATATGGAGAGTAACAAC
ATCTGCTTAGGCTTTTTGGAAATGATCCCTAGTACCTGGAATGCATATTCCAGTGCATTATGGAAAGATCTGATAA
AGACTCAAGAATCATCAACTAATGTGGTAGTTAATTATGGGGACTTTGTTTCTTTGCAAGGTTTAATGAGACTTAT
TGGGGAATTGTTAGTGACATGGAAAGTCTGGATCCTAAACTCTCAATGGGATGTTAGTTACAATTTTGATTATTTC
ATGTTAGAGTCATTCCATGGGAGCCTCATTTTTTCACACCACCATGAAGAGATGGTTGACTTTACAAATTTTGTTC
AAACAGTTAATCCCTACAAATACTCAGAAGACACTTATCTTCCTAAGTTTTGGTTTCTGTTTTTCAAGTGCTCATT
TTCTGAGTCTGATTGTCAACTTTTGGAAAACTGCCAACCCAATGCTTCTTTGGACTTACTGCCCAGACACCTTTTT
GACCCTGTCATAAGTGAAGAGAGCTGCAATATATAC
>mr10_PS .
TGGGCTAGCCATCTCAGACAATGATCAAAGTATCCAATTCCTCTCATATTTGAGAACAGAAATGGAAAAAAATACA
GTCTGCATGGCCTTTGTGAACATGATCCCAGGCAACACACAGCTATACATGTCAAGAGCTGAAGTGTACTACAAAC
AAATCATGACATCATCTGCAAATGTGGTTATCATTTATGGTGACACAGATGGTACTCTAGCTGTGAGCTTCAGAAT
GTGGGAATCTCGAGGTATACAGAGAATATGGGTCACCACCTCACAGTGGGATGTCACTACAAGTCAGAGAGACTTC
ACACTTGACTCATTCCATGGGAATCTTGCTTTTGCACACCACCATGGGAAGATTTCTGGGTTTAAAAATTTTGTCC
AGACATTGAACCCTCTCAAATACTCAGACAAGTATCTGGCAAGGCTGGAGTGGATGTACTTTAACTGTGAAGACTC
AGCAGCTAACTGTAAGACACTGAAGAACTGCTCATCCAATGCCTCATGGGAAATGGGCTAATGGAACAGACTTTTG
ACATGGCCTTTAGTGATAGCAGTTATGACATATAT
>mr11 .
TGGGCTGGCCATCTCAGACAATGATCAAAGTATCCAATTCCTCTCATATTTGAGAACAGAGATGGAAAAAAGCACA
GTCTGCTTGGCCTTTGTGAACATGATCCCAGGCAACACACAGCTATACATGTCAAGAGCTGAAGTGTACTACAACC
AAATCATGACATCATCTGCAAATGTGGTTATCATTTATGGTGACACAGACGGTACTCTAGCTGTGAGCTTCAGTAT
GTGGGAATCTCGAGGTATACAGAGAATATGGGTCACCACCTCACAGTGGGATGTCACTACAGGTGAAAGAGACTTC
ACATTTGACACATTCCATGGGAATCTTGCTTTTGCACACCACCATGGGAAGATTTCTGGTTTTAAAAATTTTGTCC
AGACATTGAACCCTCTCAAATACTCAGACAAGTATCTGGCAAGGCTGGAGTGGATGTACTTTAACTGTGAAGACTC
AGCAGCTAACTGTAAGACACTGAAGAACTGCTCATCCAATGCCTCATTGGAATGGCTAATGGAACAGACTTTTGAC
ATGGCCTTTAGTGATAGCAGCTATGACATATAT
>mr13 .
TGGGCTAGCCATCTCAGACAATGATCAAAGTATCCAATTCCTCTCATATTTGAGAACAGAAATGGAAAAAAATACA
GTCTGCTTGGCCTTTGTGAACATGATCCCAGGCAACACACAGCTATACATGTCAAGAGCTGAAGTGTACTACAACC
AAACCATGACATCATCTGCAAATGTGGTTATCATTTATGGTGACACAGACGGTACTCTAGCTGTGAGCTTCAGTAT
GTGGGAATCTCGAGGTATACAGAGAATATGGGTCACCACCTCACAGTGGGATGTCACTACAAGTCAGAGAGACTTC
ACACTTGACTCATTCCATGGGAATCTTGCTTTTGCACACCACCATGGGAAGATTTCTGGGTTTAAAAATTTTGTCC
AGACATTGAACCCTCTCAAATACTCAGACAAATATCTGGCAAGGCTGGAGTGGATGCACTTCAACTGTGAAGACTC
AGCAGCTAACTGTAAGACACTGAGGAACTTCTCATCCAATGTCTCATTGGAATGGCTAATGGAACAGACATTTGAC
ATGGCCTTTAGTGATAGCAGTTATGACATATAC
>mr14_PS .
TGGTCTGATCCTCCCAGATGACCACAGAGGGATCCAGATTCTGTCAGACTTGTAAGAAGATATGGAGAGTAACAAC
ATCTGCTTAGGCTTTTTGGAAATGATCCCTCGTACCTGGAATGCATATTCCAGTGCATTATGGAAAGATCTGATAA
AGACTCAAGAATCATCAACTAATGTGGTAGTTATTTATGGGGACTTTGTTTCTTTGCAAGGTTTAATGAGACTTAT
TGGGGAATTGTTAGTGACATGGAAAGTCTGGATCCTAAACTCTCAATGGGATGTTAGTTACAATTTTGATTATTTC
ATGTTAGAGTCATTCCATGGGAGCCTCATTTTTTCACACCACCATGAAGAGATGGTTGACTTTACAAATTTTGTTC
AAACAGTTAATCCCTACAAATACTCAGAAGACACTTATCTTCCTAAGTTTTGGTTTCTGTTTTTCAAGTGCTCATT

TTCTGAGTCTGATTGTCAACTTTTGGAAAACTGCCAACCCAATGCTTCTTTGGACCTACTGCCCAGACACCTTTTT
GACCCTGTCATAAGTGAAGAGAGCTGCAATATATAC
>mr15 .
CGGGCTGGCCATCTCAGACAATGATCAAAGTATCCAATTCCTCTCATATTTGAGAACAGAGATGGAAAAAAGTACA
GTCTGCTTGGCCTTTGTGAACATGATCCCAGGCAACACACAGCTATACATGTCAAGAGCTGAAGTGTACTACAACC
AAATCATGACATCATCTGCAAATGTGGTTATCATTTATGGTGACACAGACGGTACTCTAGCTGTGAGCTTCAGAAT
GTGGGAATCTCGAGGTATACAGAGAATGTGGGTCACCACCTCACAATGGGATGTCACTACAAGTAAGAGAGACTTC
AGACTTGACTCATACCACGGGAATCTTGCTTTTGCACACCACCATGGGAAGATTTCTGGTTTTAAAAATTTTGTCC
AGACACTGAACTCTCTCAAATACTCAGACAAATATCTGGCAAGGCTGGAGTGGATGCACTTCAACTGTGAAGACTC
AGCAGCTAACTGTAAGACACTGAGGAACTTCTCATCCAATGTCTCATTGGAATGGCTAATGGAACAGACATTTGAC
ATGGCCTTTAGTGATAGCAGTTATGACATATAC
>mr16 .
TGGGCTGGCTATCTCAGACAATGATCAAAGTATCCAATTCCTCTCATATTTGAGAACAGAGATGGAAAAAAATACA
GTCTGCTTGGCCTTTGTGAACATGATCCCAGGCAACACACAGCTATACATGTCAAGAGCTGAAGTGTACTACAACC
AAATCATGACATCATCTGCAAATGTGGTTATCATTTATGGTGACACAGACGGTACTCTAGCTGTGAGCTTCAGTAT
GTGGGAATCTCGAGGTATACAGAGAATATGGGTCACCACCTCACAGTGGGATGTCACTACAGGTGAAAGAGACTTC
ACATTTGACACATTCCATGGGAATCTTGCTTTTGCACACCACCATGGGAAGATTTCTGGTTTTAAAAATTTTGTCC
AGACACTGAACTCTCTCAAATACTCAGACAAATATCTGGCAAGGCTGGAGTGGATGCACTTCAACTGTGAAGACTC
AGCAGCTAACTGTAAGACACTGAGGAACTTCTCATCCAATGTCTCATTGGAATGGCTAATGGAACAGACATTTGAC
ATGGCCTTTAGTGATAGCAGTTATGACATATAC
>mr17 .
TGGGCTGGCCATCTCAGATGATGACCAAGGTATCCAATTTCTCTCGTATTTGAGAACAGAGATGGAAAAAAATACA
GCCTGCTTGGCCTTTGTGAACATGATACCAGTCAACATGCAGTTATACATGTCAAGAGCTGAAGTGTACTACAACC
AAATCATGACATCATCTGCAAATGTTGTTATCATTTATGGTGACACAGACAGTACTCTAGCTGTGAGCTTCAGAAT
GTGGGAATCTCGAGGTATACAGAGAATATGGGTCACCACCTCACAGTGGGATGTCACTACAGGTGAAAGAGACTTC
ACATTTGACACATTCCATGGGAATCTTGCTTTTGCACACCACCATGGGAAGATTTCTGGTTTTAAAAATTTTGTCC
AGACACTGAACTCTCTCAAATACTCAGACAAGTATCTGGCAAGGCTGGAGTGGATGTACTTTAACTGTGAAGACTC
AGCAGCTAACTGTAAGACACTGAAGAACTGCTCATCCAATGCCTCATTGGAATGGCTAATGGAACAGACTTTTGAC
ATGGCCTTTAGTGATAGCAGTTATGACGTATAC
>mr18 .
TGGGCTGGCCATCTCAGACAATGATCAAAGTATCCAATTCCTCTCATATTTGAGAACAGAAATGGAAAAAAATACA
GTCTGCTTGGCCTTTGTGAACATGATCCCAGGCAACACACAGCTATACATGTCAAGAGCTGAAGTGTACTACAAAC
AAATCATGACATCATCTGCAAATGTGGTTATCATTTATGGTGACACAGACGGTACTCTAGCTGTGAGCTTCAGTAT
GTGGGAATCTCGAGGTATACAGAGAATATGGGTCACCACCTCACAGTGGGATGTCACTACAGGTGAAAGAGACTTC
ACATTTGACACATTCCATGGGAATCTTGCTTTTGCACACCACCATGGGAAGATTTCTGGTTTTAAAAATTTTGTCC
AGACATTGAACCCTCTCAAATACTCAGATAAGTATCTGGCAAGGCTGGAGTGGATGTACTTCAACTGTGAAGACTC
AGCAGCTAACTGTAAGACACTGAGGAACTGCTCATCCAATGCCTCATTGGAATGGCTAATGGAACAGACATTTGAC
ATGGCCTTTAGTGATAGCAGTTATGACTTATAT
>mr19 .
TGGGCTGGCCATCTCAGACAATGATCAAAGTATCCAATTCCTCTCATATTTGAGAACAGAGATGGAAAAAAGCACA
GTCTGCTTGGCCTTTGTGAACATGATCCCAGGCAACACACAGCTATACATGTCAAGAGCTGAAGTGTACTACAACC
AAATCATGACATCATCTGCAAATGTGGTTATCATTTATGGTGACACAGACGGTACTCTAGCTGTGAGCTTCAGTAT
GTGGGAATCTCGAGGTATACAGAGAATATGGGTCACCACCTCACAGTGGGATGTCACTACAGGTGAAAGAGACTTC
ACATTTGACACGTTCCATGGGAATCTTGCTTTTGCACACCACCATGGGAAGATTTCTGGTTTTAAAAATTTTGTCC
AGACATTGAACCCTCTCAAATACTCAGACAAGAACCTGGCAAGGCTGGAGTGGATGCACTTCAACTGTGAAGACTC
AGCAGCTAACTGTAAGACACTGAGGAACTGCTCATCCAATGCCTCATTGGAATGGCTAATGGAACAGACATTTGAC
ATGGCCTTTAGTGATAGCAGTTATGACTTATAC
>mr20 .
TGGTCTGATCCTCCCAGATGACCACAGAGGGATCCAGATTCTGTCAGACTTGCAAGAAGATATGGAGAGTAACAAC
ATCTGCTTAGGCTTTTTGGAAATGATCCCTCGTACCTGGAATGTATATTCCAGTGCATTATGGAAAGATCTGATAA
AGACTCAAGAATCATCAACTAATGTGGTAGTTATTTATGGGAACTTTGTTTCTTTGCAAGGTTTAATGAGACTTAT
TGGGGAATTGTTAGTGACATGGAAAGTCTGGATCCTAAACTCTCAATGGGATGTTAGTTACAATTTTGATTATTTC
ATGTTAGAGTCATTCCATGGGAGCCTCATTTTTTCACACCACCATGAAGAGATGGTTGACTTTACAAATTTTGTTC
AAACAGTTAATCCCTACAAATACTCAGAAGACACTTATCTTCCTAAGTTTTGGTTTCTGTTTTTCAAGTGCTCATT
TTCTGAGTCTGATTGTCAACTTTTGGAAAACTGCCAACCCAATGCTTCTTTGGACTTACTGCCCAGACACCTTTTT
GACCCTGTCATAAGTGAAGAGAGCTGCAATATATAC
>mr21 .
TGGTCTGATCCTCCCAGATGACCACAGAGGGATCCAGATTCTGTCAGACTTGCAAGAAGATATGGAGAGTAACAAC
ATCTGCTTAGGCTTTTTGGAAATGATCCCTCGTACCTGGAATGCATATTCCAGTGCATTATGGAAAGATCTGATAA
AGACTCAAGAATCATCAACTAATGTGGTAGTTATTTATGGGGACTTTGTTTCTTTGCAAGGTTTAATGAGACTTAT
TGGGGAATTGTTAGTGACATGGAAAGTCTGGATCCTAAACTCTCAATGGGGCGTTAGTTACAATTTTGATTATTTC
ATGTTAGAGTCATTCCATGGGAGCCTCATTTTTTCACACCACCATGAAGAGATGGTTGACTTTACAAATTTTGTTC

AAACAGTTAATCCCTACAAATACTCAGAAGACACTTATCTTCCTAAGTTTTGGTTTCTGTTTTTCAAGTGCTCATT
TTCTGAGTCTGATTGCCAACTTTTGGAAAACTGCCAACCCAATGCTTCTTTGGACTTACTGCCCAGACACCTTTTT
GACCCTGTCATAAGTGAAGAGAGCTGCAATATATAC
>mr22 .
TGGGCTGGCCATCTCAGACAATGATCAAAGTATCCAATTCCTCTCATATTTGAGAAAAGAAATGGAAAAAAATACA
CTCTGCTTGGCCTTTGTGAACATGATCCCAGGCAACACACAGCTATACATGTCAAGAGCTGAAGTGTACTACAACC
AAATCATGACATCATCTGCAAATGTGGTTATCATTTATGGTGACACAGACGGTCTGCTAACTGTGAGCTTCAGAAT
GTGGGAATCTCGAGGTATACAGAGAATATGGGTCACCACCTCACAGTGGGATATCACTACAGGTAAGAGAGACTTC
ACACTTGACACATTCCATGGGAATATTGCTTTTGCACACCACCATGGGAAGATTTCTGGTTTTAAAAATTTTGTCC
AGATATTGAACCCTCTCAAATACTCAGACAAGTATCTGGCAAGGCTGGAGTGGATGTACTTTAACTGTGAAGACTC
AGCAGCTAACTGTAAGACACTGAAGAACTGCTCATCCAATGCCTCATTGGAATGGCTAATGGAACAGACTTTTGAC
ATGGCCTTTAGTGATAGCAGTTATGACATATAC
>mr23 .
AGGACTAGTTGTTTCAGATGATGACCCAGGCATTCAATTTCTCTCTGAATTGAGAGGAGAGATGCACAGACACAGA
GTCTGTTTAGCCTTTGTGAATATGATCACAGAAAATATACAGTTATACCAGAAAAGAACTGCAAAGTATAATAACC
AGATCATGATGTCATCAGCAAAAGTTATTATCATTTATGGGGACACAGACACTCTAACGATACACTTTAGACTATG
GCAACATTTAGGCATTCAGAGACTCTGGATCACCACCTCACAGTGGGACATGACTACAAATAGAGGAGACTCCCCA
TTCAACTCCTTCCACGGGATTTTCATTTTTTCACATCGTCATTCTGAGATTTCTGGTTTTAAAAAATTCATCCAGA
CAGAGCACCCTTCCAACTACAATAAAGATACTTCCCTTGCCAGGTTATGGTGGATGTATTTTAACTGTTCATTGTC
AGCTCATTGTAAGACACTGAAAAATTGTTCAACCAAACTCCTACTAGAATGGTTATCTAGGCACCAGCTTGAAGTG
TCCATGAGAGAGACAAGTTACAACTTATAC
>mr24 .
TGGGCTGGCCATCTCAGACAATGATCAAAGTATCCAATTCCTCTCATATTTGAGAACAGAGATGGAAAAAAGCACA
GTCTGCTTGGCCTTTGTGAACATGATCCCAGGCAACACACAGCTATACATGTCAAGAGCTGAAGTGTACTACAACC
AAATCATGACATCATCTGCAAATGTGATTATCATTTATGGTGACACAGACGGTACTCTAGCTGTGAGCTTCAGTAT
GTGGGAATCTCGAGGTATACAGAGAATATGGGTCACCACCTCACAGTGGGATGTCACTACAGGTGAAAGAGACTTC
ACATTTGACACATTCCATGGGAATCTTGCTTTTGCACACCACCATGGGAAGATTTCTGGTTTTAAAAATTTTGTCC
AGACATTGAACCCTCTCAAATACTCAGACAAGTATCTGGCAAGGCTGGAGTGGATGTACTTCAACTGTGAAGACTC
AGCAGCTAACTGTAAGACACTGAGGAACTGCTCATCCAATGCCTCATTGGAATGGCTAATGGAACAGACATTTGAC
ATGGCCTTTAGTGATAGCAGTTATGACTTATAT
>sp1_PS .
GGGACTGGATGTCTCAGATGATGACCAAAGTGTTCAGCTTCTCTCAGACTTTAGGGGAGAGATGAAAGGAAACAGA
GTCTGCTTAGGCTTTGTGAACATTATTCCTCTAAGTACATGGTTATACATGATGAGAGCTCAAATATATTATGAAC
AAATCATGGAATCATCAGCAAATGTTGTGGTCATTTATGGTAATGTGAACTCTAGTTCAAAGCTATAGATGGTGGA
AATATTTAGGCATAAGGAGAATCTGGTTCAGCACCTCACAATGGGATAATATCATCAATCAGAGAGATTTCAACAT
TGACTCATCCCTTGGGACATTCACTTTTTCACATCATCATGGTGAAATTTCTGGGTTTAAAAATTTTATCCAGACG
TTGAAACTTTCTGACCGCTCTGAAAAAATTACTCTGACTAGCCTGGGGTGGATAGGTTTTAATTGTTCAGTGGCAT
CTAGTAACTTCAAGACACTGAAGGATTGGTCATCCAACACCTCATTTGAATCGTTAATGTGGCACAATTTTAACAT
GGCCATGAGTGATGAAAGCTACAATATATAC
>sp2_PS .
GGAACTGGTTGTCTCAGATGATAACAAAAGTGTTTAATTTCTCTCAGACTTTAGGGGACAGATGAAAGAAAAGTCT
GCTTAGCCTTTGTAAGCATGATTCTTCTAAGTACATGTTTATACATGACGAGAGCTCAAATATATTATAAACAAGT
CATGGAATCATCAGCACATATTCTTGTTATTTATGCTAATGTGAACTCTACTGTAGTCGAAAACTATAGATGGTGG
AAATATATAGGCATAAGGCGAATCTGGTTATTCCCCTCAGAATGGGATAATATCATGTATCAGAGAGATTTCAACC
TTAACTCATTCCCTGGGACATTCATATTTTCACTTGACCATGGTGAAATTTCTGGTTTTAAAACTTTTATCCAGAC
ATTGAGACTTTCTGAATACTCTGAAGAAATTACTCTGACAAGAATTGGGTGGATAGAATTTAATTTTTCAGTGGAA
TCCAGTAACTTCAAGACACTGAAGGATTGCTCATCCAGTACCTCATTTGAATTGTTAATGTGGCAGAATTTTAAAC
TGGCCATGAATGATGAAAGCTACAAGATATAC
>sp4_PS .
TGAAATGGTTGTCTCAGGTGATGACCAATGTGTTCAATTTATCTCAGACTTTAGGGGACATATGAAAGAAAACACT
TTGCTTAGCCTTTGTGAGCATGATTCTTCTAAGCACATGCTTATACATGATGAGAACTCAAATATATTATAAACAA
GTCATGGAATCATCAGCACATATTCTTGTCATTTATGCTAAGGTGAACTCTACTGTAGTCGAAAACTACAGATGGT
GGAACTATTTAGGCATAAGGTGAATCTGGTTCTCCCCCTCAGAATGGAATAATATCATAAATCAAAGAGATTTCAA
CCTTAACTCATTCCCTGTGACATTCATATTTTCACATGACCATGAATTTCTGGTTTTAAAAAATTTTTATCCAGAC
ATTGAGACTTTCTGAATACTCTGAAGAAATTACTCTGACAATAAATGGGTGGATAGACTTTAATTTTTCAGTGGAA
TCCACTAACTTCAAGATACTGAAGAATTGGTCATCCAATACTTCATTTGAATCGCTAATGTGGCAGAATTTTAAAG
TGTCGATGAGTAATGAAAGCTACAAGATGTAC
>sp5_PS .
AGGGCTGGTCATCTCAGGGAATGAAAAAGGAATTCAGTTCTTACCAGACTTGAGAGGAAAGATGGAGAGGAAGAGT
CTGTATAGCCTTTGTGGAGAGGCTCAGCACCAACCACATATACCCCTTGGATGATATGTACATCTATAGCAGTCCA
ATTGTGAGGGAATCAGCAAAAGCAATTACTGTTTTTGGTGACACTGACTCTACTGTAAGTGTGACATTTATGCTAT
GGCAACATGTGGTCCCATGAAGAGTGTGGGTCACCACGTCACAGTGGGACGTTGCTGGCAGTATGTGACATTTCAT

CCTGGACTCATTCCATGGAACTCTCATCTTCTCACAACACTACCATGAGCTTTCTATTTTAAGAATTTTGTTCAGA
CTGTAAACCCTTCCAAATACCCAGAAGATGTATTCCTTTCAACATTGTGGGAAATGTATTTTAATTACCCAGCCTC
AAAGACTAATTGTAAAACACTAAAGAACTGTTCATCCAAGGGATCCTTGGAATGGCTGCCTTGACATCGCTTTGAC
ACAGACACAAGCGTACCATGTCTAC
>sp6_PS .
GGAACTGGTTGTCTCAGATGATAACAAAAGTGTTTAATTTCTCTCAGACTTTAGGGGACAGATGAAAGAAAAGCCT
GCTTAGCCTTTGTAAGCATGATTCTTCTAAGTACATGTTTATACATGACGAGAGCTCAAATATATTATAAACAAGT
CATGGAATCATCAGCACATATTCTTGTTATTTATGCTAATGTGAACTCTACTGTAGTCGAAAACTATAGATGGTGG
AAATATATAGGCATAAGGCGAATCTGGTTATTCCCCTCAGAATGGGATAATATCATGTATCAGAGAGATTTCAACC
TTAACTCATTCCCTGGGACATTCATATTTTCACTTGACCATGGTGAAATTTCTGGTTTTAAAACTTTTATCCAGAC
ATTGAGACTTCCTGAATACTCTAAAGAAATTACCCTGACAAGAATTGGGTGGATAGACTTTAATTTTTCATTGGAA
TCCAGTAACTTCCAGACACTGAAGAATTGGTCATCCAATACCTCATTTGAATCGCTAATGTTACAGAATTTTAAAA
TGGCCATGAATGATGAAAGCTACAAGATATAC
>sp8_PS .
TGAAATGGTTGTCTCAGGTGATGACCAATGTGTTCAATTTATCTCAGACTTTAGGGGACATATGAAAGAAAACACT
TTGCTTAGCCTTTGTGAGCATGATTCTTCTAAGCACATGCTTATACATGATGAGAACTCAAATATATTATAAACAA
GTCATGGAATCATCAGCATATATTCTTGTCATTTATGCTAATGTGAACTCTACTGTAGTTGAAAACTATAGATGCT
GGAAATATTTAGGCATAAGGTGAATCTGGTTCCTCCCCTAAGAATGGGATAATGTCATAAATCAGAGAGATTTCAA
CCTTAACTCATTCCCTGGGACATTCATATTTTTACATGACCATGGTGAAATTTTTGGTTTTAAAATTTTTAGCCAG
ACATTGAGACTTTCTGAATACTCTAAAGAAATTACTCTGACAAGAATTGGGTGGATAGACTTTAATTTTTCATTGG
AATCCAGTAACTTCCAGACACTGAAGAATTGGTCATCCAATAAATCATTTGAATCGTTAATGTTACAGAATTTTAA
ACTGGCCATGAGTGATGAAAGCTACAAGATATAC
>sp12_PS .
GGGACTGGTTGTCTCAGATGATGAATAAGGTGTTCAGTTCCTCTCAGACTTTAGGGGACAAATGAAAGAAAACAGA
GTCTGCTTAGCTTTTGTGAACATGATTCCTCTAAGAATGCAGTTATACATGACAAGGGCTCACATATATTATGAAC
AAATCATGACATCATCAGCAAATGTTGTGGTCATTTATGGTAATGTGAACTCTACTCTAGCTGAAAGCTATAGATG
GTGGAAATATTTAGGCATAAGGAGAATCTGGTTCAGCACCTCACAATGGGATAATATCATCAATCAGAGAGATTTC
AACATTGACTCATCCCTTGGGACATTCACTTTTTCACATCATCATGGTGAAATTTCTGGGTTTAAAAATTTTATCC
ACACGTTGAAACTTTCTGACCGCTCTGAAAAATTTATCTCTGACTAGCCTGGGGTGGATAAGTTTTAATATTCCAG
GGGTCTCTGAGAGGATTACCTCCTAGAGACTCTTGAGAACTAGAGATATCTGAATAAGATATGATCAAAGATCTCG
ACAGGACCGCAACGGACTATCCTAAATGATTCCC
>sp13_PS .
GGAATTGGTTGTCTTAGATGATGACCAAAGTGTTCAATTTCTCTCAGACTTTATGGGACAGATGAAAGAAAATAGA
GTCTGCTTAGCCTTTGTGAGCATGATTCTTCTAAGTACATGTTTATACATGATGAGAATTCAAATATATTATAAAC
AAGTCATGGAATCATCAGCATATATTCTTGTCATTTATGCAAATGTGAACTCTACTGTAGGTGAAAACTAAAGATG
GTGGAAATATTTAGGCATAAGGCAAATCTGGTTCTTCCCCTCAGAATGGGATAATATCATAAATCAGAGAGATTTC
AACCTTAACTCATTCCCTGGGACATTCATATTTTTACATGACCATGTTGAAATTTTTGGTTTTAAAATTTTTAGCC
AGACATTGAGACTTTCTGAATACTCTAAAGAAATTACTCTGACAAGAATTGGGTGGATAGACTTTAATTTTTCATT
GGAATCCAGTAACTTCCAGACACTGAAGAATTGGTCATCCAATACCTCATTTGAATCGTTAAATGTTACAGAATTT
TAAAATGGCCATGAGTGATGAAAGCTACAAGATATAC
>sp18_PS .
AGGGCTGGTCATCTCAGAGAATGAAAAAGGAATTCAGTTCTTACCAGACTTGAGAGGAAAGATGGAGAGGAAGAGT
CTGTATAGCCTTTGTGGAGAGGCTCAGCACCAACCACATATACCCTTTGGATGATATGTACATCTATAGCAGTCCA
ATTGTGAGGGAATCAGCAAAAGCAATTACTGTTTTTGGTGACACTGACCCTACTGTAAGTGTGACATTTATGCTAT
GGCAACATGTGGTCCCATGAAGAGTGTGGGTCACCACGTCACAGTGGGACGTTGCTGGCAGTATGTGACATTTCAT
CCTGGACTCATCCCATGGAACTCTCATCTTCTCACAACACTACCATGAGCTTTCTATTTTAAGAATTTTGTTCAGA
CTGTAAACCCTTCCAAATACCCAGAAGATGTATTCCTTTCAACATTGTGGGAAATGTATTTTAATTACCCAGCCTC
AAAGACTAATTGTAAAGCACTAAAGAACTGTTCATCCAAGGGATCCTTGGAATGGCTGCCTTGACATCGCTTTGAC
ACAGACACAAGCGTACCATGTCTAC
>mcr1a .
TGGACTGGTCATCTCTGACAGTGATCTTGGCATTCAATTTCTCTCATATTTGAGAAGAGAGTTTGGAAAAAAATACA
GTCTGCTTTGCCTTTGTAAATATAATTCCAATCAGTATGCAATTATACATGTCAAGAGCTGAATTGTATTATAACA
AAATCAAGGCATCATCCACAAAAGTTGTTATCATTTATGGTGACACAGACAGTACTCTTGCTGTAAGCTTTCGAAT
GTGGGAATCTCGAGGTTTACAGAGAATATGGGTCATCACCTCACAGTGGGATGTGACTACCAGTAAAAGAGATTTC
ATGCTTGAATCACCACATATGACTATAGCTTTTGCACGCCAACATGGTGAGATTACTGGTTTTAAAAATTTTGTCC
AGACAATAAACCCTCTCAGGTACACAGATAAATACCTTGCAAATCTGGAGTGGATGAAGTTTAACTGTGAAGTCTC
CAGGTCCAACTGTAAGACACTGAAGAGATACTCATCAAATATCTCCATGGAATGGCTAGTGGTACAGACTTTTGAC
ATGGCCTTTAGTGGTGGCAGTTATGACATATAC
>sp20_PS .
GGAACTGGTTGTCTCAGATGATAACAAAAGTGTTTAATTTCTCTCAGACTTTAGGGGACAGATGAAAGAAAAGTCT
GCTTAGCCTTTGTAAGCATGATTCTTCTAAGTACATGTTTATACATGACGAGAGCTCAAATATATTATAAACAAGT
CATGGAATCATCAGCACATATTCTTGTTATTTATACTAATGTGAACTCTACTGTAGTCGAAAACTATAGATGGTGG

AAATATATAGGCATAAGGCGAATCTGGTTATTCCCCTCAGAATGGGATAATATCATGTATCAGAGAGATTTCAACC
TTAACTCATTCCCTGGGACATTCATATTTTCACTTGACCATGGTGAAATTTCTGGTTTTAAAACTTTTATCCAGAC
ATTGAGACTTTCTGAATACTCTGAAGAAATTACTCTGACAAGAATTGGGTGGATAGACTTTAATTTTTCAGTGGAA
TCCAGTAACTTCAAGACACTGAAGGATTGCTCATCCAGTACCTCATTTGAATTGTTAATGTGGCAGAACTTTAAAC
TGGCCATGAGTGATGAAAGCTACAGGATATAC
>sp21_PS .
GGAATTGGTTGTCTTAGATGATGACCAAAGTGTTCAATTTCTCTCAGACTTTATGGGACAGATGAAAGAAAATAGA
GTCTGCTTAGCCTTTGTGAGCATGATTCTTCTAAGTACATGTTTATACATGATGAGAATTCAAATATATTATAAAC
AAGTCATGGAATCATCAGCATATATTCTTGTCATTTATGCTAATGTGAACTCTACTGTAGTTGAAAACTATAGATG
CTGGAAATATTTAGGCATAAGGTGAATCTGGTTCTTCCCCTAAGAATGGGATAATGTCATAAATCAGAGAGATTTC
AACCTTAACTCATTCCCTGGGACATTCATATTTTTACATGACCATGGTGAAATTTTTGGTTTTAAAATTTTTAGCC
AGACATTGAGACTTTCTGAATACTCTAAAGAAATTACTCTGACAAGAATTGGGTGGATAGACTTTAATTTTTCATT
GGAATCCAGTAACTTCCAGACACTGAAGAATTGGTCATCCAATAAATCATTTGAATCGTTAATGTTACAGAATTTT
AAACTGGCCATGAGTGATGAAAGCTACAAGATATAC
>sp23_PS .
GGAATTGGTTGTCTTAGGTGATGACCAAAGTGTTCAATTTCTCTCAGACTTTATGGGACAGATGAAAGAAAATAGA
GTCTGCTTAGCCTTTGTGAGCATGATTCTTCTAAGTACATGTTTATACATGATGAGAATTCAAATATATTGTAAAC
AAGTCATGGAATCATCAGCATATATTCTTGTCATTTATGCAAGTGTGAACTCTACTGTAGGTGAAAACTAAAGATG
GTGGAAATATTTAGGCATAAGGCAAATCTGGTCCTTCCCCTCAGAATGGGATAATATCATAAATCAGAGAGATTTC
AACCTTAACTCATTCCCTGGGACATTCATATTTTTATATGACCATGTTGAAATTTTTGGTTTTAAAATTTTTAGCC
AGACATTGAGACTTTCTGAATACTCTAAAGAAATTACTCTGACAAGAATTGGGTGGATAGACTTTAATTTTTCATT
GGAATCCAGTAACTTCCAGACACTGAAGAATTGGTCATCCAATACCTCATTTGAATCGTTAATGTTACAGAATTTT
AAAATGGCCATGAGTGATGAAAGCTACAAGATATAC
>sp24_PS .
AGGGCTGATCATCTCAGAGAATGAAAAAGGAATTCAGTTCTTACCAGACTTGAGAGGAAAGATGGAGAGGAAGAGT
CTGTATAGCCTTTGTGGAGAGGCTCAGCACCAACCACATATACCCCTTGGATGATATGTACATCTATAGCAGTCCA
ATTGTGAGGGAATCAGCAAAAGCAATTACTGTTTTTGGTGACACTGACTCTACTGTAAGTGTGACATTTATGCTAT
GGCAACATGTGGTCCCATGAAGAGTGTGGGTCACCACGTCACAGTGGGACGTTGCTGGCAGTATGTAACATTTCAT
CCTGGACTCATCCCATGGAACTCTCATCTTCTCACAACACTACCATGAGCTTTCTATTTTAAGAATTTTGTTCAGA
CTGTAAACCCTTCCAAATACCCAGAAGATGTATTCCTTTCAACATTGTGGGAAATGTATTTTAATTACCCAGCCTC
AAAGACTAATTGTAAAACACTAAAGAACTGTTCATCCAAGGGATCCTTGGAATGGCTGCCTTGACATCGCTTTGAC
ACAGACACAAGCGTACCATGTCTAC
>sp25 .
GGGACTGGTTGTCTCAGATGATGAATAAGGTGTTCAGTTTCTCTCAGACTTTAGGGGACAAATGAAAGAAAACAGA
GTCTGCTTAGCTTTTGTGAACATGATTCCTCTAAGAATGCAGTTATACATGACAAGAGCTCACACATATTATGAAC
AAATCATGACATCATCAGCAAATGTTATGGTCATTTATGGTAATGTGAACTCTACTCTAGCTGAAAGCTATAGATG
GTGGAAATATTTAGGCATAAGGAGAATCTGGTTCAGCACCTCACAATGGGATAATATCATCAATCAGAGAGATTTC
AACATTGACTCATCCCTTGGGACATTCACTTTTTCACATCATCATGGTGAAATTTCTGGGTTTAAAAATTTTATCC
AGACGTTGAAACTTTCTGACTGCTCTGAAAAAATTACTCTGACTAGCCTGGGGTGGATAGGTTTTAATTGTTCAGT
GGCATCTAGTAACTTCAAGACACTGAAGGATTGGTCATCCAACACCTCATTTGAATCGTTAATGTGGCACAATTTT
AACATGGCCATGAGTGATGAAAGCTACAATATATAC
>sp26_PS .
GGAATTGGTTGTCTTAGATGATGACCAAGGTGTTCAATTTCTCTCAGACTTTATGGGACAGATGAAAGAAAATAGA
GTCTGCTTAGCCTTTGTGAGCATGATTCTTCTAAGTACATGTTTATACATGATGAGAATTCAAATATATTATAAAC
AAGTCATGGAATCATCAGCACATATTCTTGTCATTTATGCTAAGGTGAACTCTACTGTAGTCGAAAACTACAGATG
GTGGAACTATTTAGGCATAAGGTGAATCTGGTTCTTCCCCTCAGAATGGAATAATATCATAAATCAAAGAGATTTC
AACCTTAACTCATTCCCTGTGACATTCATATTTTCACATGACCATGAATTTCTGGTTTTAAAAAATTTTTATCCAG
ACATTGAGACTTTCTGAATACTCTGAAGAAATTACTCTGACAATAAATGGGTGGATAGACTTTAATTTTTCAGTGG
AATCCACTAACTTCAAGACACTGAAGAATTGGTCACCCAATACTTCATTTGAATCGCTAATGTGGCAGAATTTTAA
AGTGTCGATGAGTAATGAAAGCTACAAGATGTAC
>sp27_PS .
TGAAATGGTTGTCTCAGGTGATGACCAATGTGTTCAATTTATCTCAGACTTTAGGGGACATATGAAAGAAAACACT
TTGCTTAGCCTTTGTGAGCATGATTCTTCTAAGCACATGCTTATACACGATGAGAGCTCAAATATATTATAAACAA
GTCATGGAATCATCAGCACATATTCTTGTTATTTATGCTAATGTGAACTCTACTGTAGTCGAAAACTATAGATGGT
GGAAATATATAGGCATAAGGCAAATCTGGTTATTCCCCTCAGAATGGGACAATATCATGTATCAGAGAGATTTCAA
CCTTAACTCATTCCCTGGGACATTCTTATTTTCACATGACCATGGTGAAATTTCTGGTTTTAAAACTTTTATCCAG
ACATTGAGACTTTCTGAATACTCTGAAGAAATTACTCTGACAAGAATTGGGTGGATAGACTTTAATTTTTCAGTGG
AATCCAGTAACTTCAAGACACTGAAGGATTGCTCATCCAGTACCTCATTTGAATCGTTAATGTGGCAGAATTTTAA
ACTGGCCATGAGTGATGAAAGCTACAAGATATAC
>sp29_PS .
GGGACTGGTTGTCTCAGATGATGAATAAGGTGTTCAGTTTCTCTCAGACTTTAGGGGACAAATGAAAGAAAACAGA
GTCTGCTTAGCTTTTGTGAACATGATTCCTCTAAGAATGCAGTTATACATGACAAGAGCTCACATATATTATGAAC

AAATCATGACATCATCAGCAAATGTTGTGGTCATTTATGGTAATGTGAACTCTACTCTAGCTGAAAGCTATAGATG
GTGGAAATATTTAGGCATAAGGAGAATCTGGTTCAGCACCTCACAATGGGATAATATCATCAATCAGAGAGATTTC
AACATTGACTCATCCCTTGGGACATTCACTTTTTCACATCATCATGGTGAAATTTCTGGGTTTAAAAATTTTATCC
AGACGTTGAAACTTTCTGACTGCTCTGAAAAAATTACTCTGACTAGCCTGGGGTGGATAGGTTTTAATTGTTCAGT
GGCATCTAGTAACTTCAAGACACTGAAGGATTGGTCATCCAACACCTCATTTGAATCGTTAATGTGGCACAATTTT
AACATGGCCATGGGTGATGAAAGCCACAATATATAC
>an1 .
CGGGGTGATCATCTCAGAAGACCAACAAGGTATTCAGATGCTCTCAGACTTGAAAGAAGAAATGGACAGAAACAGT
GTTTGTGCAGGATTTGTGGCAAGGTTCCCAGTTTCTTATGCAGCACTTGCCTCAGTTTTCTGGTTACACGATAATT
TCATTTTGCAAACAAGGACAAATGTGATTATCACTTATGGTGACACTGAGTTTCTAAGAGGCTTCTTGATTTTCTT
AAAAGACACTTTAGTAACATGGAAAGTATGGGTCATGAACTCAGAATGGAATCCACTCTCCCTTCGCAGACATTTC
ATTTTATATTCATTGCATGGGGCTCTCATTTTTTCACACCACCATGAGGAAATCACTGGTTTCAGAGATTTTATCC
AGACAGCTAACCCTTCCAAATACCCAGAAGATGATTACCTTACTAAGTTGTGGGTTTTATATTTTAATTGCTCTTT
TTCTGAGGCTGACTCTAATAAAATGGAGAACTGTCCACCCAATGCTTCCTTGGAATGGTTGCCTGGGGATCTTATT
GATATGACCATCAGTGAATACAGTTATAATATATAC
>an2_PS .
CGGGGTGATCATGTCAGAAGACCAACAAGGTATCCAGATGCTCTCAGACTTGAAAGAAGAAATGGACAGAAACAGT
GTTTGTGCAGGATTTGTGGCAAGGTTCCCAGTTTCTTATGCAGCACTTGCCTCAGTTTTCTGGTTACATGATAATT
TCATTTTGCAAACAAGGACAAATGTGATTATCACTTATGGTGACACTGAGTTTCTAAGAAGCTTCTTGATTTTCTT
AAAACACACTTTAGTAACATGGAAAGTATGGGTCATGAACTCAGAATGGAATCCACTCTCCCTTCGCCGACATTTC
ATTTCACATTCATTGCATGGGGCTCTCATGTTTTCACACCACCATGAGGAAATCACTGGTTTCAGAGATTTTATCC
AGACAGCTAACCCTTCCAATTACCCGGAAGATGATGACCTTATAAAGTTGTGGGGTTTTATATTTTAATTGCTCTT
TTTTTGAGGCTGACTGTAATAAAATGGAAAACTGTCCACACATTGCTTCCTTGGAATGCTTGCCAGGGGATCTTAT
TGATACATCCATGAGTGAATGCAGTTATAATATATAC
>an3_PS .
GGGGCTGTTCATTTCAGAAGATCTGAGAGGTATTGAATTTCTCTCAGACTTGAGAAGAGAGATGGAAAGGCATAGA
GTCTGTGCAGCCTTTGTGAATATGATCCCAGACTCCATGATGTTCTATTTGAAAAGAGCTCGTGTGTACCCAGATT
GCGATTTCATCAGCAAATGTTATAATCATTTTTGGTGACAGCGACTCTTTTCTTGGCCTTCCATTAATAACATGGA
GTACTCTAATCACCAAGAAAGTCTGGGTCACTACTTCACAGTGGGATGTTACCACCAGTAAGAAACATTTCATTCT
TGACTCTTTCAATGGGACTCTTATTTTTTCACAGCTTCATGAAAATGTTTCTGCCTTTAAAGATTTTATCAGGAGA
GTGAATCCTTCCAAATAGCCAGAAGACATATTCCTGGCTAGGTTGTGGTTGATATCTTTTAATTGCTCAAGTCCAA
AATCTACCTGCAAAACACTGAAGAATTTCTCCTCCAAAGGCTCCTTGGAATGGTTACCATGGTACCATTTTGAAGT
GAGGGGAGTTAAAATATTGAC
>an4 .
CGGGGTGATCATCTCAGAAGACCAACAAGGTATTCAGATGCTCTCAGACTTGAAAGAAGGAATGGACAGAAACAGT
GTTTGTGCAGGATTTGTGGCAAGGTTCCCAGTTTCTTATGCAGCACTTGCCTCAGATTTCTGGTTACACGATAATT
TCATTTTGCAAACAAGGACAAATGTGATTATCACTTATGGTGACACTGAGTTTCTAAGAGGCTTCTTGATTTTCTT
AAAAGACACTTTAGTAACATGGAAAGTATGGGTCATGAACTCAGAATGGAATCCACTCTCCCTTCGCAGACATTTC
ATTTTACATTCATTGCATGGGGCTCTCATTTTTCCACACTACCATGAGGAAATCACTGGTTTCAGAGATTTTGTCC
AGACAGCTAACCCTTCCAAATACCCAGAAGATGATTACCTTACTAAGTTGTGGGTTTTATATTTTAATTGCTCTTT
TTCTGAGGCTGACTCTAATAAAATGGAGAACTGTCTACCCAATGCTTCCTTGGAATGGTTGCCTGGGGATCTTATT
GATATGGCCATCAGTGAATACAGTTATAATATATAC
>an5_PS .
GGGGGTGATCATCTCAGAAGACCAACAAGGTATTCAGATGCTCTCAGACTTGAAAGAAGAAATGGACAGAAACAGT
GTTTGTGCAGGATTTGCGGCAAGGTTCCCAGTTTCTTATGCAGCACTTGCCTCAGATTTCTGGTTACATGATAATT
TCATTTTGCAAACAAGGACAAATGTGATTATCACTTATGGTGACACTGAGTTTCTAAGAAGCTTCTTGATTTTCTT
AAAAGACACTTTAGTAACATCAAAAGTATAGGTCATGAACTCAGAATGGAATCCACTCTCCCTTCGCAGACATTTC
ATTTTACATTCATTGCATGGGGCTCTCATGTTTTCACACCACCATTAGGAAATCACTGGTTTCAGAGATTTTATCC
AGACAGCTAACCCTTCCAAATACCCAGAAGATGATTACCTTACTAAGTTGTGGGTTTTATATTTTAATTGCCCTTT
TTCTGAGGCTGACTGTAATAAAATGGAGAACTGTCCACCCAATGCTTCCTTGGAATGGTTGCCTGGGTATCTTATT
GATATGACCATGAGTGAATACGGTTATAATATATAC
>an10_PS .
GGGGCTGTTCATTTCAGAAGATCTGAGAGGTATTGAATTTCTCTCAGACTTGAGAAGAGAGATGGAAAGGCATAGA
GTCTGTGCAGCCTTTGTGAATATGATCCCAGACTCCATGATGTTCTATTTGAAAAGAGCTCGTGTGTACCCAGATT
GCGATTTCATCAGCAAATGTTATAATCATTTTTGGTGACAGCGACTCTTTTCTTGGCCTTCCATTAGTAACATGGA
GTACTCTAATCACCAAGAAAGTCTGGGTCACTACTTCACAGTGGGATGTTACCACCAGTAAGAAACATTTCATTCT
TGACTCTTTCAATGGGACTCTTATTTTTTCACAGCTTCATGAAGATGTTTCTGCCTTTAAAGATTTTATCAGGAGA
GTGAATCCTTCCAAATAGCCAGAAGACATATTCCTGGCTAGGTTGTGGTTGATATCTTTTAATTGCTCAAGTTCAA
AATCTACCTGCAAAACACTGAAGAATTTCTCCTCCAAAGGCTCCTTGGAATGGTTACCATGGTACCATTTTGAAGT
GAGGGGAGTTAAAATATTGAC
>an11 .

CGGGGTGATCATCTCAGAAGACCAACAAGGTATCCAGATGCTCTCAGACTTGAAAGAAGAAATGGACAGAAACAGT
GTTTGTGCAGGATTTGTGGCAAGGTTCCCAGTTTCTTATGCAGCACTTGCCTCAGTTTTCTGGTTACACGATAATT
TCATTTTGCAAACAAGGACAAATGTGATTATCACTTATGGTGACACTGAGTTTCTAAGAAGCTTCTTGATTTTCTT
AAAAAACACTTTAGTAACATCGAAAGTATGGATCATGAACTCAGAATGGAATCCACTCTCCCTTCGCAGACATTTC
ATTTTATATTCATTGCATGGGGCTCTCATTTTTTCACACCACCATGAGGAAATCACTGGTTTCAGAGATTTTATCC
ATACAGCTAACCCTTCCAAATACCCAGAAGATGATTACCTTACTAAGTTGTGGGTTTTATATTTTAATTGCTCTTT
TTCTGAGGCTGACTGTAATAAAATGGAGAACTGTCCACCAAATGCTTCCTTGGAATGGTTGCCTGGGTATCTTATT
GATATGACCATGAGTGAATACGGTTATAATATATAC
>an12_PS .
GGGGCTGTTCATTTCAGAAGATCTGAGAGGTATTGAATTTCTCTCAGACTTGAGAAGAGAGATGGAAAGGCATAGA
GTCTGTGCAGCCTTTGTGAATATGATCCCAGACTCCATGATGTTCTATTTGAAAAGAGCTCGTGTGTACCCAGATT
GCGATTCCATCAGCAAATGTTATAATCATTTTTGGTGACAGCGACTCTTTTCTTGGCCTTCCATTAATAACATGGA
GTACTCTAATCACCAAGAAAGTCTGGGTCACTACTTCACAGTGGGATGTTACCACCAGTAAGAAACATTTCATTCT
TGACTCTTTCAATGGGACTCTTATTTTTTCGCAGCTTCATGAAGATGTTTCTGCCTTTAAAGATTTTATCAGGAGA
GTGAATCCTTCCAAATAGCCAGAAGACATATTCCTGGCTAGGTTGTGGTTGATATCTTTTAATTGCTCAAGTTCAA
AATCTACCTGCAAAACACTGAAGAATTTCTCCTCCAAAGGCTCCTTGGAATGGTTACCATGGTACCATTTTGAAGT
GAGGGGAGTTAAAATATTGAC
>an13_PS .
CGGGGTGATCATCTCATAAGACCAACAAGGTATCCAGATGCTCTCAGACTTGAAAGAAGAAATGGACACAAACAGT
GTTTGTGCATGATTTGTGGCAAGGTTCCCAGTTTCTTATGCAGCACTTGCCTCGGTTTTCTGGTTACACGATAATT
TCATTTTGCAACCAAGGACAAATGTGATTATCACTTATGGTGACACTGAGTTTCTAAGAAGCTTCTTGATTTTCTT
AAAAAACACTTTAGTAACATCGAAAGTATGGGTCATGAACTCAGAATGCAATCCACTCTCCCTTCCCAGACATTTC
ATTTTGCATTCATTGCATGGGGCTCTCATGTTTTCACACCACCTTGATGAAATCACTGGTTTCAGAGATTTTATCC
TGATAGCTAACCCTTCCTATTACCCGGAAGATGATTACCTTACTAAGTTGTGGGTTTTATATTTTAATTGCTCTTT
TTCTGATGCTGACTGTCATAAACTGTGCAACTGTCCACCCAATGCTTCCTTGGAATGGTTGCCTGCGGATCTTATT
GATATGACCATGACTGAATACAGTTATATACAA
>dp2 .
GGGACTGTTCATCACTGAAGATGACTACAGTGTGTGGTTCCTCTCAGAATTGAGAGAAGAGATGGGCAAGAATGGA
ATCTGTTTGGCCTTTGAGAACATGATTACTTACATAGCCGTTTCAGATATCTCAAATGATCTATCAATGCATCGCC
AGCTAATGGAATCATCAACCAACGTGGTTATCATTTATGGGGACAGTAAATTTCTATTAAGCTTTATTCTTAAATT
TGGGCACATTCTAATTATAGGGAAAGTCTGGGTCATGAACTCACCCTATGAGGACATGATCATTGGTAAAAGATAT
TTTCTGATCAACTCCTTCCATGCACCTCTCATCTTCTCTCACCATCATAGAAATACTTCTGGGTTCACCAGTTTTA
TTCTGACAGATACCTTGTCCAAGAACTCAGGAGTAAATTCCCTTCCCATGTCATGGATTCAGTCTGTTTATTGCCT
AGTGATTAAGTCTGACTGCAGAGACTTGGGGCACTGTTCATACAATGCCACACTGGATTGGTTGCTGAGGCACATT
TTTGACATGACCATGTCAGACGAGAGTTACAACATCTA
>dp5 .
GGGACTATTCATCACCGAAGACAACTATGGTATGTGGTTCCTCTCAGAATTGAGAGAAGAGATGGGCAAGAGTAGA
GTCTGCTTTGCCTTTGAGAACATGATCACTTACAGTGTTCTTTCAGATTTCTCAAATGATCTATCGATGCAGCGCC
AGTTAATGGACTTATCAACCAATGTGATTATCATTTATGCGGACAGTAAATTTCTACTAGGCTTCATTCTTAAATT
TAGGCACTTTTTAATTACAGGCAAAGTCTGGGTCCTGAACTCACCCTATGATGATGTGACTATTACTAAAAGACAT
TTTCTGATTGACTCTTTCCATGCACCTCTTATCTTCTCTCACCATCATAGAAACACTGCTGGGTTCACCAATTTTA
TTCTGACAGAGACCTTCTCCAAGAACTCAGCAAATAATTCCCTTCCCAGGTCATGGATTCAGTCTGTTTATTGCCT
ACTGATTAAGTCTGACTGCAGAGACTTGGGGCACTGTTCATACAATGCCACACTGCATTGGTTGCTGAGGCACATT
TTTGACATGACCATGTCAGATGAGAGTTACAACATCTA
>dp6_ .
GGGACTGTTCATCACTGAAGACAACCATGGTGTGTGGTTCCTCTCAGAGTTGAGAGAAGAGATGGGCAAGAGTAGA
GTCTGTTTTGCCTTTGAGAACATGATCACTTACAATGCCTTTATAGATTACTCAGATGATCTATCCAAGTATCGCC
AGCTAATGGACTCATCAACCAATGTGATTATCATTTATGGGGACAGTAAATTTCTACTAAGCTTTATTTTTAAATT
TGGGCACATTTTAATTACAAGGAAAGTCTGGGTCATGAACTCACCCTATGACGATGCCACCATTGGTAAAAGATAT
TTTCTGATCCACTCCTTTCATGCATCTCTCACCTTCTCTCATCATCATAGAAACACTTCTCTGTTCACCAATTTCA
TTCTGACAGATACCTTGTCAAATAACTTAGGAAATAATTCCCTTCCTAGGTCATGGATTCAGTCTGTTTATAACCT
ACTGATTAAGTCTGACTGCAGAGACTTGGGGCACTGTTCATACAATGCCACACTCCATTGGTTGCTGAGGCACATT
TTTGACATGACCATGTCAGAAGAGAGTTACAACATCTT
>dp7_ .
GGGTCTGGTGATCTCAGAAGATGAGAATGGTTTGTGGATGCTGCCCCAATTGAGACAAGAGATGAACAAGCACAAA
GTCTGTACAGCTTTTGAACACATACTACCACTTTCTTTCTATCATGAAGCAACTGTATTTCGACCCTTTCATCGGA
AAATTAGAAAATCACTCACAACTGTGGTTGTCATTTACGGTAACAATGAATTCGTGTTCAATTTTTTAAACGCTTA
TTTCACAAGATCTTTAAGTCTAGGAACCATCTGGGTCATAAACTCCCTTAGTACATTTCTGTATGAGATGAAATAT
TTTTTGTTATACTCATTCCATGCACCTCTCATTTTCTCACACCACCATGGAGAGATTTCTGGTTTCAACAATTTTA
TCAAAACTGCTAACCCATCCAAATACCCAGAAGACTATTTCCTTGCTTTGGTGTGGATTCGCTTTTTTAATTGCAC
ATTTTCTGAGTCTAGTTGTGTCACTTGGAAGAACTGTCCAGCCAATGCGTCCTTGGAATGGTTGCCTAGGTACCTT
TTTGACATGGGCATGATGGAAGAGAGTTACTATATATA

>dp8 .
GGGTCTGTTGATCTCAGAAGATGAGAATGGTTTGTGGATGCTGCCCCAATTGAGAGAAGAGATGAACAAGCACAAA
ATCTGTACAGCTTTTGAACACATAATACCACTTTCTTTCTTTCGTGAAAGAACCGATTATGTATCTTTTCATCAGA
AAATTAGAAATTCATTCGCAAATGTGGTTCTCCTTTATGGTGACAATGAGTTCATGGTCAACTTGTTAAATGCTTA
TTTAACAAGTTTTTTAAGTCTAGGAACCGTCTGGGTCATAAACTCCCTTAGTACATTTCTGTACGTGATGAAATAT
TTTTTGTTACATTCACTCCATGCACCTCTCATTTTCTCACACCACCATGGAGAGATTTCTGGTTTCAACAATTTTA
TCAAAACTGCTAACCCATCCAAATACCCAGAAGACTATTTCCTTGCTTTGGTGTGGATTCGCTCTTTTAAGTGCAC
ATTTTCTGAGTCTAGCTGTGTCACTTGGAAGAACTGTCCAGCCAATGCGTCCTTGGAATGGTTGCCTAGGTACCTT
TTTGACATGGGCATGATGGAAGAGAGTTACTATATATA
>dp12 .
GGGATTATTGATCTCAATGGATGAGAAGGGTTTATGGATCCTTCCACAACTGAGGGATGAGATGTCCAAGAATGAA
GTCTGTGTAGCCTTTGAGGAAATGATCCCAGGCAGTGTCTTTACAGAAATGTCAAAAATCATAGGAATATACAACC
AAATTAATACATCATCAGCAAACGTCTTCATTATTTTTGGTGACACTGAAACCATTGCAGACTTGATGTATTCTGT
ACCACTTTTAATAAAAGGCAAAGTTTGTATCATGAATTCACAGTGGGATTTAACCAGAAAGAGAGATTTCGTGTTA
GGCTCATTCCATGTGCCTCTCATTTTTTCACACCACCATTCAGATATTTCTGGATTCCAAGATTTTTTAAGGACAG
TTAACCCTTCCAAATACCCAGGTGACTATTTTCTTGCTAGGTTGTGGCTCCTTCATTTTAATTGCTCAGATTCTGA
GATTGATTGCTTAACATGGGAGAACTGTCCACAGAATGCTTCCTTGGAATGGTTGCCTGGGCAGGTTTTTGACATG
GCCATGTCTGAAGAGAGTTACAATATATA
>dp14 .
GGGGCTATTGATCTCAGAAGATGAGAATGGTTTATGGATCCTTGCAGAACTGAGAGAAAAGATGTACAAGAGCAGA
ATTTGTATAGCCTTTGAGCATGTGATCCCAGGCAGTAGTATGACACAAGTATTTAATATGATGGCAATTCAGAACC
AGATTAATAAATCATCAGCAAATGTACTTATCATTTATGGTAATAAAGAATATCTGCTAAGTTTAATGATATATCT
TGGGCAATTTTTAATGACAAGCAAAGTTTGGCTCTTGAACTCAAAGTGTGATTTATCCATCGGAAGGAAGTATTTC
ATGTCAGGTTCATACCATGTACCTCTTATGTTTTCACATCACCATGGAGACATTTCTGGATTCACAGATTTTATCA
GGGAAGTTAACCCTTCCAAGTACCCAGAAGACTTTTATCTTGCTAGGCTATGGTTTCTCTCTTTTAATTGCTCCCA
TTCTGAGTCTGACTGTGTAATGTGGAAGAACTGTCCAGATGATACTTCCTTAGAATGGTTGCCTGGGCAAATTTTT
GACATGTCCATGTCTGAAGAGAGTTATAATACATA
>dp15 .
GGGACTGTTGAATTTACAAGATGAGAATGATTTGTGGGTTCTTGCTGAATTGAAAGAAGAGATGGCCAAGAACAGA
GTTTGTGTAGCCTATGAGCTAGTGATCTCAAACAAATCAATATCATATATAGGTAAAGCCATGGCAGGTTATAATG
AGGCAAATAAATCATCAAATGTCCTCATCATACATGGTAATAATGATTCACTAATAATTTTAATAATGGGCATTGA
GCAACTTTTAATAAAAGGCAAAGTTTGTATAATGAACGCACAGTGGGAATTTACCCTGAAAACGAGATATTTCATG
TTAGGCTCACTGCATGTTCCTCTTATTTTTTCACACCACCATGCAGATGTTTCTGGATTCACAGATTTTATCAAGG
CAGCTAACCCTTCCAAATACCCAGAAGACATTTTTCTTGCTAGGATGTGGTTTCACTTTTTTAATTGCTCAGATGC
TGAGTCTGACTGTGTGACATGGGACAACTGTCCTCATGACGCTTCTTTGGATTGGTTGCCTGGGCATGTTTTTGAC
ATGAATATGTCTGGACACAGTTACAATATATA
>dp17 .
AGGGCTTTTGATCTCACAAGATGAGAATGGCTTATCGATCCTTGCAGAACTGCGAGAAGTAATGGACAAGAACAAA
ATCTGTGTAGCCTTTGAGCATATGATCTCTGGCCGTACTCTGACACAAGTAATTAACATCATGACAATTCAGAACC
AGATTAATAAATCATCAGCAAATGTACTTATCATTTATGGTAATAAAGAATACCTGCTAGGCTTAATGGTATATCT
TGGGCAATTTTTAATAACAAGCAAAGTTTGGCTCTTGAACTCACAGTGTGATTTATGCATTGGAAAAAAGTATTTC
CTATTAGGCTCATTCCATGTACCTCTCATTTTTTCATATCACCATGAACACATAGCTGGATTCACAGATTTTATCA
GGGAAGTCAACCCTTCCAAATATCCAGAAGACTTTTACCTTGCTAAGTTGTGGTTTCTCTCTTTTAATTGCTCCCA
TTATGAGTCTGACTGTGTAATGTGGGAGAACTGTCCACATGATTCCTCCTTAGAATGGTTGCCTGTGCAAATTTTT
GACATGGCCATGTCTGAGGAGAGTTACAATATCTA
>dp18 .
GGGACTATTGACCTCGCAAGATGAGAATGGTGTGTGGATTCTTGTTGAATTGAAAGAAGAGATGGCCATGAACAGA
GTTTGTGTAGCCTATGAGCTAGTGATCTCATCCCAAGACACATCACATATATTCAAACTCATGGCAGTTCATAATG
AGATAAATCTATCTTCAGCAAGTGTTCTCATCATATATGGTGATAATGATTCAATGATAGGTTTAGTGGTATTTAT
CGAGCAAATTTTTATACATGGCAAAGTTTGCATTATGAACTCACAGTGGGATTTCACATTGAAAAGGAGATATTTC
ATGCTAGGATCATTGCATGTACCTCTTATTTTTACACACCATTATGTAGATGTTTCTGGATTCACAGATTTTATCA
AGGCAGCTAAGCCTTCAAAATACCCAGAAGACATTTTTCTTGCCAGACTGTGGTTTCTGTCTTTTAATTGCTCAGC
TTCTGAGTCTGACTGTGTGACTTGGGACAACTGTGTTCATGATGCCTCTTTGGATTTGTTGCCTGTACATATTTTT
GATATGACTATGTCTAGAGACAGTTACAATGTTTA
>dp19 .
GGGACTATTGACCTCGCAAGATGAGAATGGTGTGTGGATTCTTGTTGAATTGAAAGAAGAGATGGCCATGAACAGA
GTTTGTGTAGCCTATGAGCTAGTGATCTCATCCCAAGACACATCACATATATTCAAACTCATGGCAGTTCATAATG
AGATAAATCTATCTTCAGCAAGTGTTCTCATCATATATGGTGATAATGATTCACTGATAAGTTTAGTGGTATTTAT
CGAGCAAATTTTTATACATGGGAAAGTTTGCATTATGAACTCTCAGTGGGATTTCACATTGAAAAGGAGATATTTC
ATGCTAGGATCATTGCATGTACCTCTTATTTTTACACACCATTATGTAGATGTTTCTGGATTCACAGATTTTATCA
AGGCAGCTAAGCCTTCAAAATACCCAGAAGACATTTTTCTTGCCAGACTGTGGTTTCTGTCTTTTAATTGCTCAGC

TTCTGAGTCTGACTGTGTGACATGGGACAACTGTGTTCATGATGCCTCTTTGGATTTGTTGCCTGTACATATTTTT
GATATGACTATGTCTAGAGACAGTTACAATGTTTA
>dp20 .
GGGACTGTTGACCTCACAAGATGAGACTGGTCCATCCTTTCTTGCTGAATTGAAAGAAGAGATGGCCAAGAACAAA
ATATGTGTAGCCTATGAGCTAATGATCTCAACCCAAGATATGTCAAATGTATTCAGATTCATGTCAGTTCATAATG
AGATAAATCTATCTTCAGCAAGAGTACTCATCATATATGGTGATAATGATACACTGATAGCTTTAATGGTATTGAT
TGAGCAAATTTTTATACATGGAAAAATTTGTATTGTGAACTCTCAGTGGTATTTCACTCTGAAGAAGAAATATTTC
ATCCTGGGCTCCTTGTATGTACCTCTTATTTTTACACATCATCATGCAGAAGTTTCTGGATTCACAGATTTTGTCA
AGGTAGTTAAGCCTTCCAAATACCCAGAAGACATTTTTCTTGCGAGGCTGTGGTTTCTCTCTTTTAATTGCTCTGA
TTCTGAGTCTGACTGTGTAACATGGGACCATTGTTCTCATGATGCCTCTTTGGATTGGTTACCTGGGCATATTTTT
GAAATGACTATGTCTGGAGATAGTTACAATATATA
>dp21 .
GGGACTGTTGACCTCAGATGATGAGAATGGTTTGTGGGTTCTTGTTAAATGGAAAGAAGAAATGGCCAAGAACAAA
GTTTGTGTAGCCTATGAGCTAGTGATCTCAAGCAAAACCGTCTCACAGATGTTCAAATTAATGGCAGTTTATACTC
AGATGAATAAATCTTCAGCAAATGTCCTCATGATATATGGTGATAACGAGTCACTACTAGATTTAATTATGTGTAT
TGAGCAACTTTTACTAAAAGGAAAAGTTTGCATCATGAACTTACAGTGGGATTTAACCTTAAGAAAGAATCGTTTC
ATGCTAGGTTCATTCCATGTACCTCTCATTTTTTCACACCATCATGCAGATGTTTCTGGATTCACAGATTTTATCA
AGGCAGTTAATCCTTCCAAATACCCAGAAGACTTTCTTCTTGCTAAGCTGTGGTTTGTGTCTTTTAATTGTTCTGA
TTCTGAGTCTGACTGTGTAACATGGGACAATTGTCTTCATGATGCCTCGTTGGATTGGTTGCCTGGATATATTTTT
GACAGGACTCTGTCCGGAGAAAGTTATCATATATA
>dp22 .
AGGATTGTTAACCTTACAAGATGAAAATGGTTTGCGGAGTCTTCCTGCATTGAAAGAAGAGATGGCCAAGAATAAT
TTGATCTGGCAATAACAAACCATTTATGTACATTTTAAAAATCATGGCACCCTATAGCCATATAAGTAAATCATCA
GCCTATGTCCTCATCCTATACGTTCATAATGAATTCCTACTATATTTAATTGTGTGTGTTGAGCAAATTTTAATAA
AAGGAAAAGGTGTAGAATGGCATTTAACCATAAGAAAAATATTTCTTGCTTAGTTCATTACATGTACCTGTCATGT
TTTTACATTATCACTCAGATGTTTTTGGAATCACAAATGTTGTCAAAGTAGTTAACCCTTCCAAATACAGGAGACT
TTTTCCTTGGTGGGCTGTGGTTTCTCTTTTACTTGCTCAAGTTCTGATTCTGATTTGGTAATGGGGAGAACTATTC
TCATGATGCTTCTTTGGCTTGGTTGCCTATGTACATTTTTGACATGACTATTTCCGGAGACAGTTACAATATATA
>dp24 .
GGGACTGTTGACCTCACAAGTTGAGAATGGTGCATGGGTTCTTGCTGAATTGAAAGATGAGATGGCCAGAAACAGA
GTTTGTGTAGCCTATGAGCTAGTGATCTTAAGCCAAGACGTGTCATATATATTCAAACTCATGGCAGTTCATAGCG
AGGTAAATCTATCTTCAGCAAGTGTCCTCATCATATATGGTGATAATGATTCACTGATACTTTTAATGGTATTTAT
TGAGCAAATTTTTATGCATGGCAAAGTTTGTATCATGAACTCTCAATGGGATTTCACCATGAAGAGGAAATATTTC
ATGATAGGGTCATTGCATGTACCTCTGATTTTTGTACATCATCATGTAGAAGTTTCTGGATTCATAGATTTTGTCA
AGGCAGTTAAGCCTTCCAAATACCCAGAAGACATTTTTCTTGCCAGGCTGTGGTTTCTGTCATTTAATTGCTCATA
TTCTGAGTGTGACTGTGTGGCATGGGACAACTGTGCTCAGGATGCCTCTTTAGATTTGTTGCCTGGGCATATTTTT
GACACGAAAATGTCTGGAGACAGTTACAATGTATA
>dp25 .
GGGACTGCTGACCTCACAAGATGAGAATGCTTCATGGGTTCTTGCTGAATTAAAAGAAGAGATGGCCAAGAACAGA
GTTTGTATAGCCTATGAGCTAGTGATGTCAACCCAAGACGCATCACATATATTCAAACTCGTAGCAGTTCATAAAG
AGATAAATGTATCTTCAGCAAATGTCATCATCATATATGGTGATAATGATTCCCTGATAGCTTTAATGGTATTTAT
TGAGCAAATATTTATGCATGGTAAAGTTTGTATTGTGAACTCACAGTGGGATTTCACCCTGAAAAGGAGATATTTC
ATGCTAGGCTCATTGCATGTACCCTTTATTTTCACACATCATCATGTAGATGTTTCTGGATTCACAGATTTTGTCA
AGGCAGTTAAACCTTCCAAATACCCAGAAGACATTTTTCTTGCAAGGCTGTGGTTTCTCTCTTTTAATTGTTCAGA
TGTTGAGTCTGACTGTGTAACATGGGACAACTGTGCTCATGATACCTCTTTGGATTTGTTGCCTGCATGCACTTTT
GACATGACTATGTCTGTGGATAGTTACAATATATA
>dp26 .
GGGACTATTCATCACTGAAGACAACAATGGTATGTGGTTCCTCTCAGAATTGAGAGAAGAGATGAGCAAGAGTAGA
GTCTGCTTTGCCTTTGAGAACATGATCACTTACAGTGTCCTTTCAGATTACTCAAATGATCTATCAATGCAGCGCC
AGCTAATGGACTTATCAACCAATGTGATTATCATTTATGCGGACAGTAAATTTCTACTAGGCTTCATTCTTAAATT
TAGGCACTTTTTAATTACAGGCAAAGTCTGGGTCATGAACTCGCCCTATGATGATGTTACTATTGCTAAAAGACAT
TTTCTGATTGACTCTTTCCATGCACCTATTATCTTCTCTCACCATCATAGAAACACTGCTGGGTTCACCAATTTTA
TTCTGACAGAGACCTTCTCCAAGACCTCAGCAAATAACTCCCTTCTCAGGTCATGGATTCAGTCTGTTTATTGCCT
ACTGATTAAGTCTGACTGCAGAGACTTGGGGCACTGTTCATACAATGCCACACTGCATTGGTTGCTGAGGCACATT
TTTGACATGACCATGTCAGATGAGAGTTACAACATCTA
>dp29 .
GGGACTATTCATCACCGAAGAAAACTATGGTATGTGGTTCCTCTCAGAATTGAGAGAAGAGATGGGCAAGAGTAGA
GTCTGCTTTGCCTTTGAGAACATGATCACTTACAATGTCCTTGCAGATTTCTCAAATGATCTATCGATGCAGCGCC
AGCTAATGGACTTATCAACCAATGTGATTATCATTTATGGGGACAGTAAATTTCTACTAAGCTTTATTCTTAAATT
TAGGCACTTTTTAATTACAGGCAAAGTCTGGGTCCTGAACTCACCCTATGATGATGTGACTATTGCTAAAAGATAT
TTTCTGATTGACTCTTTCCATGCACCTCTTATTTTCTCTCACCATCATAGAAACACTGCTGGGTTTACCAATTTTA
TTCTGACAGAGACCTTCTCCAAGAACTCAGCTAATAACTCCCTTCCCAGGTCATGGATTCAGTCTGTTTATAGCCT

```
ACTGATTAAGTCTGACTGCAGAGACTTGGGGCATTGTTCATACAATGCCACACTGCATTGGTTGCTGAGGCACATT
TTTGACATGACCATGTCAGATGAGAGTTACAACATCTA
>dp31 .
GGGACTATTCATCACCGAAGATGACTATGGTGTGTGGTTCCTCTCAGAATTGAGAGAAGAGATGGACAAGAATGGA
GTTTGTTTGGCCTTTGAGAACATGATCACTTACAGTGCCTTTTTAGATTTCACAAATGATCTATGGATGCATCGCC
AGCTAATGGAATCATCAACCAATGTGATTATCATTTATGGGGACAGTAAATTTCTACTAAGCTTTATTCTTAAATT
TGGGTACTTTTTAATTACAGAGAAAGTCTGGGTCATGAACTCACCCTATGATGATGTGACTATTGCTAAAAGACAT
TTTCTCATTGACCCTTTCCATGCACCTCTCATCTTCTCTCACCACCATAGAAACACTTCTGGGTTCACCAATTTTA
TTCTGACAGAGATCTTCTCCAAGAACTCAAGAGATAATTCCCTTCCCATGTCATGGATTCAGTCTGTTTATTGCTT
ACTGGTTAAGTCTGACTGTAGAGACTTGGGGCACTGTTCATACATTGCCACACTGGATTGGATGCTGAGGCACATT
TTTGACATGACCATGTCAGACGAGAGTTACAACATCTA
>dp32 .
GGGACTTTTCATCACCGAAGAGGACTATGGTGTGTGGTTCCTCTCAGAATTGAGAGAAGAGATGGACAAGAATGGA
GTCTGTTTGGCCTTTGAGAACATGATCACTTACAATGCCCTTTCAGATTTCACAAATGATCTATGGATGCATCTCC
AGCTAATGGAATCATCAACCAATGTGATTATCATTTATGGGGACAGTAAATTTCTATTAAGCTTTATGCTTAAATT
AAGGGACTTTTTATTTACAGGGAAAGTCTGGGTCATGAACTCACCCTATGATGACGTGACCATTGGTAAAAGATAT
TTTCTGATTGACTCTTTCCATGCACCACTCATTTTCTCTCATCATCATAGAAATACTGCTGGGTTCACCAATTTTA
TTCTAAGCGAGACATTGTCGAAGAACTCAGGAGATAATTCCCTTCCCAGGTCATGGATTCAATCTGTTTATTGCCT
ACTGATTAAGTCTGACTGCAGAGACTTGGGGCACTGTTCGTACAATGCCACACTGGATTGGTTGCTGAGGCACATT
TTTGATATGACCATGTCAGACGAGAGTTACAACATCTA
>dp46 .
GGGACTGTTGACCTCACAAGATGAGAATGCTTCATGGGTTCTTGCTGAATTGAAAGAAGAGATGGCCAAGAACAGA
GTTTGTATAGCCTATGAGCTAGTGATGTCAACCCAAGACACATCACATATATTCAAACTCGTAGCAGTTCATAATG
AGATAAATATATCTTCAGCAAGTGTTCTCATCATATATGGTGATAATGATTCACTGATGGGTTTAGTGGTATTTAT
CGAGCAAATTTTTATACATGGCAAAGTTTGCATTATGAACTCACAGTGGGATTTCACACTGAAAAGGAGATATTTC
ATGCTAGGCTCATTGCATGTACCTCTTATTTTTACACACCATTATGTAGATGTTTCTGGATTCACAGATTTTATCA
AGGCAGCTAAGCCTTCAAAATACCCAGAAGACATTTTTCTTGCCAGACTGTGGTTTCTGTCTTTTAATTGCTCAGC
TTCTGAGTCTGACTGTGTGACATGGGACAACTGTGTTCATGATGCCTCTTTGGATTTGTTGCCTGTGCATATTTTT
GATATGACTATGTCTAGAGACAGTTACAATGTTTA
>dp33 .
GGGGCTGTTGATCTCACTAGATGAAAGTGGTTTGTGGATAATTCCAGAATTGAGGGAAGAGATGGCCAAGAACAGA
GTCTGTGTAGCCTTTGAGCATGTGGTTTCAAGCAATCTGCTGGCACAAGCACTAAACTTCATGATAATCCATAGCC
AGATTACTAGATCATCAGCAAATGTATTCATCATTTATGGTGATCATGAATACCTACTAAACTTAACACTGTATTT
TGGGCAATTTTTAATGACAGGCAAGGTTTGGCTTATGACTTCACAGAGTGATTTAACCATCAGTAGAAGATCTTTT
ATCTTAGGCTCATTCCATGTCCCTCTCATGTTCTCACACCACCATTCAGACATTTCTTGGTTTAAGAACTATGTCA
GGGAAGTTAATCCTTCCAAATACCCAGAAGACTTTTACCTTGCTAGGCTGTGGGTTCTCTCTTTTAATTGCTCCCA
TTCTGAGTCTGACTGTGTAACATGGGAGAACTGTCCCCAGGATGCCTCCTTGGAATGGCTGCCTGGACACATTTTT
GACATGGCCATGTCTGAAGAGAGTTATAATATATA
>dp34 .
AGGGCTATTGATCTCACTAGATGAAAATAGCTTGTGGATAATTTCAGAACTGAGAGAAGAGATGGCTAAGAACAGA
GTCTGTGTAGCCTTTGAGCATGTGATTTCAAGCAATATGTTCATGGAAGTAACAAATTACATAAGATTCCATCACC
AGATAAATAACTCATCAGCAAATGTATTTATCATTTATGGTGATAATGAACACCTACTAAACTTAACGCTGTATTT
TGGGCAATTTTTAATGACAGGCAAAGTTTGGCTCATGACTTCACAGAGTGAGTTATTCATCAGTAGAAGGTATTTC
ATGTTAGGCTCATTCCATGTCCATCTCATTTTTTCACACCACCATCCAGACATTTCTGGGTTTAAGAATTATGTCA
GGGAAGTTAATCCTTCCAAACACCCAGAAGACTTTTATCTTGCTCGGCTGTGGGTTCTTTCTTTTAATTGCTCATA
TTCTGAATCTGACTGTGTAACATGGGAGAACTGTCCCCAGGATGCCTCCTTGGAATGGCTGCCTGGACACATTTTT
GAAATGGCCATGTCTGAAGAGAGTTACAATATATA
>dp35 .
GGGGCTGTTGATCTCACAAGATGAGAATGGCTTGTGGATTCTTCTAGAATTGAGAGAAGAGATTTCCAAGAACAGA
ATCTGCATAGCCTTTGAGGAAGTGGTGCCAGTCAGCACTTATTCAGAGATATGGAGACTCATGGTAATATATAATG
AGATTAATAAGTCATCAGCAAATGTTTCCATCATTTATGGTGATAATGAATCCCTACTTGAGTTAATGTTGTATTT
TGAGCAATTTTTAATAACAGGAAAAGTTTGTATCATAAACTCCTCATGGGATTTAACTATGAGAAGCAGATACTTC
ATACTTGGCTCATTCCATGTACCTCTCATATTTTCACACCACTTTGCAGACATTGCTGGGTTCACGGATTTTATAA
GGGCAGTTAACCCTTCCAAATACCCAGAGGACTTTTACCTTGCTAGGGTTTGGCTTTTCTCTTTTAACTGTTCACA
GAATGAATCTGACTGTGTAACCTGGATGAACTGTCCATATGATACCTCCTTGGAATGGTTGCCTGGGCAGGTTTTT
GACATGACCATGTCTGAAGAGAGTTATAATATATA
>dp36 .
AGGGTTATTGATATCACAAGATGAGAATGGTTTGTCGATCCTTGCAGAACTGAGAGAAGCAATGGCCAAGAACAGA
ATCTGTGTAGCCTTTGAACATATGATCTCTGGCAGTACTGCAACACAAGTAGTTAACATCATGGCAATTCAGAACC
AGATTAATAAATCATCAGCAAATGTACTTATCATTTATGGTAATAAGGAATACCTGCTAGGATTAATGGTATATCT
TGGGCAATTTTTAATAACAAGCAAAGTTTGGCTCTTGAACTCACAGTGTGATTTATGCATTGGAAGAAATTATTTC
ATATTAGGCTCATTTCATGTGCCTCTCATTTTTTCATATCACCATGAAGACATAGCTGGATTCACAGATTTTATCA
```

GGGAAGTCAACCCTTCCAAATACCCAGAAGACTTTTACCTTGCTAAGTTGTGGTTTCTCTCTTTTAATTGCTCTCA
TTTTGAGTCTGACTGTGTAATGTGGGAGAACTGTCCACATGATGCCTCCTTGGAATGGTTGCCTGGGCAAATTTTT
GACATGGCCATGTCTGAAGAGAGTTACAATATCTA
>dp37 .
GGGGCTGTTGATCTCACTAGATGAAAGAGGTTTGTGGATAATTCCAGAATTGAGAGAAGAGATGGCCAAGAACAGA
GTCTGTGTAGCCTTTGAGCATGTGATTCCAAGCACTATCCTGACACAAGTATTAAACACAATGACAATCCATCACA
AGATTAGTAAATCATCAGCAAATGTGTTTATCATTTATGGTGATAATGAATACCTACTAGACTTAATGACTTATTT
TGGGAAGATTTTAATGTCAGACAAAGTTTGGCTTATGACTTCACAGAGTGATTTTTCCACCAGTAGAAGATACTTC
ATTTTAGGCTCATTCCATGTCCCTCTCATGTTCTCACACCACCATTCAGACATTTCTTGGTTTAAGAACTATGTCA
GGGAAGTTAATCCTTCCAAATACCCAGAAGACTTTTACCTTGCTAGTCTGTGGGTTCTCTCTTTTAATTGCTCCCA
TTCTGAGTCTGACTGTGGAACATGGGAGAACTGTCCCCAGGATGCCTCTTTGGAGTGGCTGCCTGGACACATTTTT
GACATGGCCATGTCTGAAGAGAGTTACAATATATA
>dp38 .
GGGACTGTTGAATTTACAAAATGAGAATGATTTGTGGGTTCTTGCTGAATTGAAAGAAGAGATGGCCAAGAACAGA
GTTTGTGTAGCCTATGAGCTAGTGATCTCAAGCCAATTAATATCACATATATTCAAAGCCATGGCAGGTTATAATG
AGATAAATAAATCAGCAAATGTGCTCATCATACTTGGTGATAATGATTCACTAATAATTTTAATAATAGGTATTGA
GCAACTTTTAACAAAAGGCAAAGTTTGTATCATGAACTCACAGTGGGAATTTGCCCTGAAAAGAAGATATTTCTTG
TTAGGTTCACCACATGTTCCTCTTATTTTTTCACACCATCATGCAGATGTTTCTGGATTCACAGATTTTGTCAAGG
CAGTTAACCCTTCCAAATACCCAGAAGATATTTTTCTTGCTAGGATGTGGTTTCTCTCTTTTAATTGCTCAGCTGC
TGTGTCTGACTGTGTAACATGGGACAATTGTCTTTGTAACGCGTCTTTGGATTGGTTGCCTGGGCATATTTTTGAC
ATGAATATGTCTGGACACAGTTACAATATATA
>dp39 .
AGGGTTATTGATATCACAAGATGAGAATGGTTTGTCGATCCTTGCAGAACTGAGAGAAGCAATGGCCAAGAACAGA
ATCTGTGTAGCCTTTGAGCATATGATCTCTGACAGTCCTACAACACAACTAATTAACATCATGGCAATTCAGAACC
AGATTAATAAATCATCAGCAAACGTACTTATCATTTACGGTAATAAGGAATACCTGCTAGGATTAATGGCATATTT
TGGACAATTCTTAATAACAAGCAAAGTTTGGCTCTTGAACTCACAGTGTGATTTATGCATTGGAAGAAATTATTTC
ATATTAGGCTCATTTCATGTGCCTCTCATTTTTTCATATCACCATGAAGACATAGCTGGATTCACAGATTTTATCA
GGGAAGTCAACCCTTCCAAATATCCAGAAGACTTTTACCTTGCTAAATTGTGGTTTCTCTCTTTTAATTGCTCCCA
TTTTGAGTCTGACTGTGTAATGTGGGAGAACTGTCCACATGATGCCTCTTTGGAATGGTTGCCTGGGCAAATTTTT
GACATGGCCATGTCTGAAGAAAGTTACAATATCTA
>dp40 .
TCATATTTTGAAATTCATGGCACATTATAACCAGATAAATAAATCTTCAGCTAAAGTCTTCATTCTATATGTTAAT
AATGAATTCCTAAACTATGTAATGATAGCTATTGAGCAATTTTTAATAAAAGGAAAAGTTTTTATCACAAACTCAC
AGTGGGATTTCATCATGAGGAAAAAATATTTCATTCTTGGTTCATTTCATGTACCTCTCATTTTTTCACACCATCA
CCCAGATATTTCTGCATTCATGGATTTTGTCAAGGCAATAAACCCTTCCAAATATCCAGAAGATTTTTTCCTTGCC
AGGCTGTGGTTTTGTTTTTTTAATTGTTCAGATTCTCAGTCTGACTGTGTAATGTGGGGGAACTGTCCACATGATG
CCTCCTTGGATTGGTTGCCTATGCACATTTTTGGTGTAACTATGTCTGGATACAGTTACAATATATA
>dp42 .
AGGGTTATTGATATCACAAGATGAAAATGGTTTGTCAATCCTTGCAGAACTGAAAGAAGCAATGGTCAAGAACAGA
ATCTGTGTAGCCTTTGAGCATATGATCTCTGACAGTCCTCAAACACAACTAATTAACATCATGGCAATTCAGAACC
AGATTAATAAGTCATCAGCAAATGTACTTATCATTTATGGCAATAAAGAATACCTGCTAGGATTAATGGTATATCT
TGGGCAATTTTTAATAACAAGCAAAGTTTGGCTCTTGAACTCACAGTGTGATTTATGCATTGGAAGAAATTATTTC
ATATTAGGCTCATTTCATGTGCCTCTCATATTTTCATATCACCATGAAGACATAGCTGGATTTACAGATTTTATCA
GGGAAGTCAACCCTTCCAAATACCCAGAAGACTTTTACCTTGCTAAATTGTGGTTTCTCTCTTTTAATTGCTCCCA
TTTTGAGTCTGACTGTGTAATGTGGGAGAACTGTCCACATGATGCCTCCTTGGAATGGTTGCCTGGGCAAATTTTT
GACATGGCCATGTCTGAAGAGAGTTACAATATCTA
>dp43 .
GGGACTGATAACCATACAAGATGAGAATGGTTTGTGGATTCTTCCTGCATTGAAAGAAGAGATGGGCAAGAACAGA
GTTTGTGTAGCCTTTGAGCTATCATTACCAAATAATTCCATGTCGCATATTTTCAAAATTATGGTACATTATAACC
AGATAAATAAATCTTCAGCAAATATCTTCATTGTGTATGCTGATAATGAGTTCCTACGACATTTATTTATATGTGT
TGAACAATTTTTAATACAAGGCAAAGTTTTTATTATGAACTCAGAGTGGGATGTAACCATGAGAAAGAGATATTTC
ATTCTAGGTTCATTCCTTGTACCTCTCGTTTTCTCACACCATCACCCAGATGTTTCTGGATTCACAGATTTTCTCA
AATCAGTTAACCCGTGCAAATACCCAGAAGACTTTTTTCTTGCTAGGTTATGGTTTCTCTCTTTTAATTGCTCAGA
TTCTGTGTCTGACTGTGTACAGTGGGAGAACTGTCCTCATGATGCCTCCTTGGATTGGTTGCCTAGGCATATATTT
GACATGAGTATGTCTGGAGACAGTTACAATATATA
>dp44 .
GGGTCTATTGACCTCACAGGATGAAAATGGTTCATGGGTTCTTGCTGAATTGAAAGAAGAGATGACCAAGAACAGA
GTTTGTGTGGCCTATGAGCTACTGATCTCAAGCAAAGGTGTGTCTTATCTATTCAAATTAATGGAAGTTTATAACA
AGATAAATAAATCTTCAGCAAGTGTCCTCATCATATATGGTGATAATGAATCACTACTAGATTTAATGGTGTGTAT
TGAGCAACTTTTAATCAAAGGCAAAGTTTGTATCATGAACTCACAGTGGGATTTAACCATGAGAAGGAAATATTTC
ATTCTAGGCTCATTGCATGTTCCTCTTATTTTTTCACATCATCATACAGATCTTTCTGGATTCACAGATTTTGTCA
AGGCAGTAAACCCTTCCAAATACCCAGAAGACATTTTTCTTGCTAGGATGTGGTTTCTGTCATTTAATTGCTCAGA

TGCTGACTCTGACTGTGTAACATGGGACAATTGTCCTCATGATGCCTCCTTGGATTGGTTGTCTGGGTATATTTCT
GTCATGACTATGTCTGGAGATAGTTACAATATATA
>dp45 .
GGGACTGTTAACATTACAAGATGACAGTGGTTTGTGGATTCTTCATGAATTGAAAGAAGAGATGGCCAAGAACAGA
GTTTGTGTAGCCTTTGAGCTAGCAATCCCAAACTATAGCCTGTCATTAAAAGTCATGGCACTTTATAACCAGATTA
ACAAATCTTCAGCAAATGTCCTCATCATATATGGTGATAATGAATCCTTACTAGATTTAATGATATGTATTGAGCA
GTATTTAATAAAAGGAAAAGTTTGTATCATGAACTCACAGTGGGATTTAACCACAAAGAGGACATATTTCATGCTA
GGTTCATCCCATGTACCATTAATTTTTTCACACCATCATCCAGATGTTTCTGGATTCACAGATTTTCTCAAGGCAG
TTAACCCTTCCAAACACCCAGAAGACTTTTTCCTTGCTAGGCTTTGGTTTCTCTCTTTTAATTGCTCAGATTCTGA
GTCTGACTGTATAACATGGGAGAATTGTCCTGATGATGCCTCCTTGGATTGGTTGCCTGGGCATATTTTTGACATG
ACTATGTCTGGACACAGTTACAATATATA
>dp48 .
GGGGCTGTTGATCTCACTAGATGAAAGTGGTTTGTCGATAATTCCAGAATTGAGGGAAGAGATGGCCAAGAACAGA
GTCTGTGTAGCCTTTGAGCATGTGATTCCAAGCACTATCCTGACACAAGTATTAAACATAATGACAATCCATCGCA
AGATTAGTAAATCATCAGCAAATGTGTTTATAATTTATGGTGATAATGAATACCTACTAGATTTAATGACTTATTT
TGGGAAGATTTTAATGTCAGGCAAAGTTTGGCTTATGACTTCACAGAGTGATTTATCCATGAGTAGAAGATATTTC
ATCTTAGGCTCATTCCATGTTCCTCTCATGTTTTCACACCACCATTCAGACATTTCTGGGTTTAAGAATTATGTCA
GGGAAGTCAATCCTTCCATATACCCAGAAGACTTTTACCTTGCTAGGCTGTGGGTTCTCTCTTTCAATTGCTCATA
TTCTGAGTCTGACTGTGTAACATGGGAGAACTGTCCCCAGGATGCCTCCTTGGAATGGCTGCCTGGACACATTTTT
GACATGGCCATGTTTGAAGAGAGTTATAATATATA
>dp49 .
AGGACTGTTGACCTCAGATGATGAGAATGGTTTGTGGATCCTTCCTGAATTGAAAGAAGAGATGGCCAAGAATACA
GTTTGTGTAGCCTTTGATCTAGTGATTCCAAATCATTCCATGTCACATATATTAAAAATCGTAGCAGTATATAACC
AGATTAACAAATCCGCAGCAAATGTCATCATCATCTATGGTGACAGTGAATCTCTACTAGATTTAATGATGTGTGT
TGAGCAAATTTTAATTACAGGCAAAGTTTGTATCATGAACTCACAGTGGCATTTAAAAATGAAAAGGAAATATTTC
ATGCTAGGTTCCTTCCATATACCTTTCATTTTTTCACATCACTATGCAGACAGTATTGGATTCACAAATTTTATAC
GGAAAGTTAACCCTTCCAAATACCCAGAAGACTTTTACCTTGCCAGGTTATGGTTTCTCTCTTTTAATTGTTCCCA
TTCTGAGTCTGACTGTGTGATATGGGAGAACTGTCCACAAGATGCCTCCTTGGAATGGTTGCCTGGACACATTTTC
GACATGGCCATGTCTGAAGAGAGTTACAATATATA
>dp52 .
AGGGCTATTGATCTCACAAGATGAGAATGGTTTATCAATCCTTTCAGAACTGAGAGAAGCAATGGACAAGAACAAA
ATCTGTGTAGCCTTTGAACATATGATTTCTGGCAGTACTCTGACACAAGTAATTAACATCATGACAATTCAGAACC
AGATCAATAAATCATCAGCAAATGTACTTATCATTTATGGTAAAAAGGAATACCTGCTAGACTTAATGGTATATCT
TGGGCAATATTTAATAACAAGCAAAGTTTGGCTCTTGAACTCACAGTGTGATTTATCCATTGGGAGGAAGTATTTC
ATGTTAGGCTCATTCCATGTACCTCTCATTTTTTCATATCACCATGAAGACATAGCTGGATTTACAGATTTTATCA
GGGAAGTCAACCCTTCCAAATACCCAGAAGACTTTTACCTTGCTAAGTTGTGGTTTTTCTCTTTTAATTGCTCCCA
TTTTGAGTCTGACTGTGTAATGTGGGAGAACTGTCCACATGCTGCCTCCTTGGAATGGTTGCCTGTGCAAATTTTT
GACATGGCCATGTCTGAAGAGAGTTACAATATCTA
>dp54 .
AGGACTATTGATCTCACAAGAGGAGAATGGTTTATTGATCCTTTCAGAACTGAGAGAAGCAATGGACAAGAACAGA
ATCTGTGTAGCCTTTGAGCATATGATCTCTGGCAGTACAAAGACACAATTATTTAACATTATGGCAATTCAGAACC
AGATTAATAAATCATCAGCAAATGTAGTTATCATTTACGGGAGTAAGGAATACCTGCTGGACATAATGGCATATCT
TGGGCACTATTTAATAAGAAGCAAAATTTGGCTCTTGAACTCACAGTGTGATTTATCCATTGGAAGGAAGTATTTC
ATGTTAGGCTCATTCCATGTACCTCTCATTTTTTCACATCACCATGCAGACATTTCTGGATTCACAGATTTTATCA
GGGAAGTCAACCCTTCCAAATACCCAGAAGACTTTTACCTTGCTAAGTTGTGGTTTCTCTCTTTTAATTGCTCCCA
TTATGAGTCTGACTGTGTAATATGGGAGAACTGTCCACATGATGCTTCCTTGGAATGGTTGCCTGTGCAAATTTTT
GACATGGCCATGTCTGAAGAGAGTTACAATATCTA
>dp55 .
GGGGCTATTGATCTCAGAAGGTGAGAATGGTTTATGGATCCTTGCAGAACTGAGAGAAAAGATGTACAAGAACAGA
ATCTGTATAGCCTTTGAGCATGTGATCCCAGGCAGTAGTCTGGCACAAGTATTTAATATGATGGGAATTCAGAACC
AGATTAATAAATCATCAGCAAATGTACTTATCATTTATGGTAATAAAGAATATCTGCTAAGCTTAATGATATTTCT
TGGGCAATATTTAATGACAAGCAAAGTTTGGCTCTTGAACTCAAAGTGTGATTTATCCATCGGAAGGAAGTATTTC
ATGTCAGGCTCATTCCATGTACCTCTTATGTTTTCACATCACCATGGAGACATTTCTGGATTCACAGATTTTATCA
GTAAAGTCAATCCTTCCAAATATCCAGAAGACTTTTATCTTGCTAGATTATGGTTTCTCTCTTTTAATTGCTCCCA
TTTTGAGTCTGACTGTGTAATGTGGAAGAACTGTCCAGATGATACTTCCTTGGAATTGTTGCCTGGGCAAATTTTT
GACATGTCCATGTCTGATGAGAGTTACAATACATA
>dp56 .
GGGACTATTGACATCAGACGATGACAATGGTTTGTGGATTCTTACTGAACTGAAAGAAGAGATGGCCAAGAACACA
GTTTGTGTAGCTTTTGAGCTAGTGATGCCGAACCATTCCCTGTCCCATGTGTTCAAACTTGTAAAAGTTTATAACA
AGATTAATAAATCTTTATCAAATGTCCTCATCATATATGGTGATAATGAATCCCTACTAGATTTAATGTCATGTGT
TGAGCAATATTTAATAACAGGCAAAGTTTGTATCATGAACTCACAGTGGCATTTAACCATGAAAAGGAAATATTTC
ATGCAAGGCTCCTTCCATATACCTCTCATTTTTTCACACTACCATGCAAAAAGTAGTGGATTCACAGATTTTATAA

GGGCAGTTAACCCTTACAAATACCCAGAAGACTTTTACCTTGCTTGGTTGTGGTTTCTGTCTTTCAATTGTTCAGT
TTCTGACTCTGACTGTGTGACATGGGAGAACTGTCCACATGATGCCTCCTTGGAATGGTTGCCTGGACACATTTTT
GACATTGCTATGTCTAAAGAGAGTTACAATATCTA
>dp57 .
GGGAATATTGACCTCACAAGATGAGAATGGTGCCTGGATTCTTGCTGAATTGAAAGAAGAGATGGCCAAAAACAGA
GTTTGTGTAGCCTATGAGCTAATGATCTCAACCCAAGACTTGTCATATATATTCAAACTCATGGCAGTTCATAATG
AGATAAATCTATCTTCAGCAAGTGTCCTCATCATATATGGTGATAATGATTCACTGATAGCTTTAATGATATTTAT
CGAGCAAATTTTTATACATGGCAAAGTTTGTATTGTGAACTGTCAGTGGGATTTCACCATGAAAAGGAAATATTTC
ATGCTAGGCTCATTGCATGTACCTCTTATTTTTACACACCATCATGTCGAAGTTTCTGGATTCACAGATTTTGTCA
AGGCAGTTAAACCTTCCAAGTACCCAGAAGACATTTTTCTTGCAAGGCTGTGGTTTCTCTCTTTTAATTGCTCAGA
TTCTGAGTCTGACTGTATAACATGGGAGAACTGTGTTCATGATGCCTCATTGGATTGGTTGCCTGTGCATATTTTT
GAAATGACTATGTCTGGAGACAGTTACAATATATA
>dp58 .
GGGGCTGTTGACCTCACAGGATGAGAATGGTTCATGGTTTCTGGCTGAATTGAAAGAAGAGATGGCCAAAAACAAA
GTTTGTGTAGCCTATGAGCTAGTTGTCTCAACCCAAGACCTGTCATATTTATTCAGAGTGATGGCAGTTTATAACA
AGATCAATAATTCTTCAGCAAGTGTTCTCATCATATATGGTGATAAAGATTCCCTAATATCTATAATAATGAGTCT
TGAGCAAATTTTAATACAAGGCAAAGTTTGTGTCATGAATTTACAGTGGGATTTCACCATGAAAACGAGATATTTC
ATGCTAGGCTCATTGCATGTACCTCTTGTTTTCACACAGCATCATGTAAATGTTTCTGGATTCACAGATTTTGTCA
AGGCAGTTAATCCTTCCAAATACCCAGAAGATATTTTCCTTGCTAGGCTGTGGTTTCTGTCATTTAATTGCTCAGA
TGCAGAGTCTGACTGTGTAATATGGGACAACTGTCCTCCTGACACCTCTTTGGATTGGTTGCCTGGACATATTTTT
GACATGACTATGTCTGGATACAGCTATAATATATA
>dp60 .
GGGTCTGTTGATCTCAGAAGATGAGAATGGTTTGTGGATGCTGCCCCAATTGAGACAAGAGATGAGCAAGCACAAA
GTCTGTACAGCTTTTGAACACATACTACCACTTTCTTTCTTTCGTGAATTAACCGATTATGTATCTTTTCATCAGA
AAATTAGAAATTCATTCGCAAATGTGGTTGTCATTTACGGTGACAATGAATTCATGTTCAATTTGTTAAAAACTTA
TTTCACAAAATCTTTAAGTCTAGGAACCGTCTGGGTCATAAACTCCCTTAGTACATTTCTGAATGTGATGAAATAT
TTTTTGTTACATTCACTCCATGCCCCTCTCATTTTCTCACACCACCATGGAGAGATTTCTGGTTTCAACAATTTTA
TCAAAACTGCTAACCCATCCAAATACCCAGAAGACTATTTCCTTGCTTTGGTGTGGATTCGCTTTTTTAAGTGCAC
ATTTTCTGAGTCTAGCTGTGTCACTTGGAAGAACTGTCCAGCCAATGCGTCCTTGGAATGGTTGCCTAGGTACCTT
TTTGACATGGGCATGATGGAAGAGAGTTACTATATATA
>dp61 .
GGGACTGTTAACCACACAAAATGAGCATGGTGCATGGGTTCTTGCTGAATTGAAGGAAGAGATGGCCAAGAACAGA
GTTTGTGTAGCCTATGAGCTAATGATCTCAACCCAAGACGCATCACATATATTCAAACTCATGGCAGTTCATAACG
AGATAAATCTATCTTCAGCAAATGTCCTCATCATATATGGTGATAATGATTCACAGACAGCTTTAATGGTATTGAT
TGAGCAAATCTTTATACATGGCAAAGTTTGTATCATGAACTCTCAGTGGGATTTCATTATGAAGAGGAAATATTTC
ATGCTAGGTTCATTGCATGTACCTCTGATTTTTACACACCATTATGTAGATGTTTCTGGATTCACAGATTTTGTCA
AGGCAGTTAAGCCTTCCAAATACCCAGAAGACATTTTTCTTGCCAGACTGTGGTTTCTCTCTTTTAATTGCTCAGA
TTCTGAGTCTGACTGTGTAACATGGGACAACTGTGCACATGATGCCTCTTTAGATTGGTTGCCTGGGCATATTTTT
GACATGACAATGTCTGAAGACAGTTACAATGTATA
>dp62 .
GGGACTGTTGACCTCACAAGATGAGAATGCTTCATGGGTTCTTGCTGAATTGAAAGAAGAGATGGCCAAGAACAGA
GTTTGTATAGCCTATGAGTTAGTGATGTCAACCCAAGACGTGTCACATATATTCAAACTGGTAGCAGTTCATAATG
AGATAAATGTATCTTCAGCAAGTGTTCTCATCATATATGGTGATAATGATTCACTGATAGGTTTAGTGGTATTTAT
CGAGCAAATTTTTATACATGGCAAAGTTTGCATTATGAACTCACAGTGGGATTTCACACTGAAAAGGAGATATTTC
ATGCTAGGCTCATTGCATGTACCTCTTATTTTTACACACCATTATGTAGATGTTTCTGGATTCACAGATTTTATCA
AGGCAGCTAAGCCTTCAAAATACCCAGAAGACATTTTTCTTGCCAGACTGTGGTTTCTGTCTTTTAATTGCTCAGC
TTCTGAGTCTGACTGTGTGACTTGGGACAACTGTGTTCATGATGCCTCTTTGGATTTGTTGCCTGTGCATATTTTT
GATATGACTATGTCTAGAGACAGTTACAATGTTTA
>dp63 .
GGGGTTGGTGATCTCACTAGATGAAAGTGGTTTGTGGATAATTCCAGAATTGAGGGAAGAGATGTCCAAGAACAGA
GTCTGTGTAGCCTTTGAGCATGTGATTCCAAGCACTATCCTTACACAGATATTTAGCAGCCTGATAATCCATCATG
AGATTAATAACTCATCAGCAAATGTATTTATCATTTATGGTGATAATGAATACCTTCTAAACTTAAGGCTGTATTT
TGGGCAGTTTTTAATGTCAAGCAAAGTTTGGCTTATGACTTCACAGAGTGATTTATCCATGAGTAGAAGGTATTTC
ATGTTAGGCTCATTCCATGTTCCTCTCATGTTTTCACACCACCATTCAGACATTTCTGGGTTTAAGAATTATGTCA
GGGAAATTAATCCTTCCAAATATCCAGAAGACTTTTACCTTGCTCGGCTGTGGTTTCTCTCTTTTAACTGCTCACA
TTCTGAGTCTGACTGTGTAACATGGGAGAACTGTCCCCAGGATGCCTCTTTGGAGTGGCTGCCTGGACACACTTTT
GACATGGCCATGTCTGAAGAGAGTTATAATATATA
>dp64 .
GGGTCTGTTGATCTCAGAAGATGAGAATGGTTTGTGGATGCTGCCCCAATTGAGAGAAGAGATGAACAACTACAAA
ATCTGTACAGCTTTTGAACACATACTACCACTTTCTTTCTTTCATGAAGTAACTGATTATGTATCCTTTCATCAGA
AAATTAGAAATTCACTCACAAATGTCGTTCTCCTTTATGGTGACAATGAGTTCATGGTCAACTTGTTAAATGTTCA
TTTCACAAGATTTTTAAGTCTAGGAACCGTCTGGGTCATAATCTCCCTTAGTACATTTCTGTATGTGATGAAATAT

TTTTTGTTACATTCACTCCATGCACCTCTCATTTTCTCACACCACCATGGAGAGATTTCTGGTTTCAACAATTTTA
TCAAAACTGCTAACCTATCCAAATACCCAAAAGACTATTTCCTTGATTTAGTGTGGATTCGCTTTTTTAAGTGCAC
ATTTTCTGAGTCTAGTCTTGTCACTTGGAAGAACTGTCCAGCCAATGCGTCCTTGGAATGGTTGCCTAGGTACCTT
TTTGACATGGGCATGATGGAAGAGAGTTACTATATATA
>dp65 .
GGGTCTGTTGATCTCAGAAGATGAGAATGGTTTGTGGATGCTGCCCCAATTGAGACAAGAGATGAACAAGCACAAA
GTCTGTACAGCTTTTGAACACATACTACCACTTTCTTTCTTTCATGAAGTAACTGAATATCTATCCTTTCATCAGA
AAATTAGAAATTCACTCACAACTGTGGTTCTCCTTTATGGTGACAATGAGTTCATGGTCAACTTGTTAAATGTTCA
TTTCACAAGATTTTTAAGTCTAGGAACCGTCTGGGTCATAAACTCCCTTAGTACATTTCTGTATGTGATGAAATAT
TTTTTGTTACATTCACTCCATGCCTCTCTCATTTTCTCACACCACCATGGAGAGATTTCTGGTTTCAACAATTTTA
TCAAAACTGCTAACCCATCCAAATACCCAAAAGACTATTTCCTTGCTTTGGTGTGGATTCGCTCTTTTAATTGCAC
ATTTTCTGAGTCTAGGTGTGTCACTTGGAAGAACTGTCCAGCCAATGCGTCCTTGGAATGGTTGCCTAGGTACCTT
TTTGACATGGGCATGATGGAAGAGAGTTACTATATATA
>dp66 .
GGGACTGTTGAACTTACGAGATGAGAATGATTTGTGGGTTCTGGCTGAATTAAAAGAAGAGATGGCCAAGAACAGA
GTTTGTGTAGCTTATGAGCTAGTTGTCTCAACCCAAGACCTGTCACATTTATTCAAATTAATGAGAGTTTATAACC
AGATAAATAAATCTTCAGCAAATGTCCTCATCATTTATGGTGATAATGAATCACTACTAGATTTAATTATATGTAT
TGAGCAACTTTTAATCAAAGGCAAAGTCTGTATCATGAACTCACAGTGGGATTCAACCATGAGAAAGAAGTATTTC
ATGCTAGGCTCACCACATGTTCTTCTTATTTTTTCACACCATCATGCAAATGTTTCTGGATTCATAGATTTTGTCA
AGACAGTTAACCCTTCCAAATACCCAGAAGACATTTTTCTTGCTAGGCTGTGGTTTCTGTTGTTTAATTGCTCAGA
TGCTGAGTCTGACTGTGCTACATGGAACAACTGTCCTCATGACGCCTCTTTGGATTGGTTGCCTGGGCATATTTTT
GACATGAATATGTCTGAAAACAGCTATAATATATA
>dp67 .
GGGACTGTTAAACTTACAGGATGAGAATGATTCGTGGATTCTTGCTGAATTGAAAGAAGAGATGGCCAAGAACAGA
GTTTGTGTAGCCTATGAGCAATTAATCTCAATCCAATCAATATCACATGTACTAGAAGCCATGGCAGTTTATAACG
AGATAAATAAATCTTCAGCAAATGTCTTCATCATACATGGTGATAATGGTAAACTAATAGAATTAATTGTGTGCAT
TGAGCAACTTTTAATAAAAGGCAGAGTTTGTATCATGAACTCACAGTGGGATTTCACCATGAAAAGGAAATATTTC
ATGCTAGGCTCATTGCATGTACCTCTCATTTTTTTACATCATCATGAAGATGTTTCTGGATTCACAGAATTTGTCA
AGACAGTTAATCCTTCCAAATACCCAGAAGACATTTTTCTTGCTAGGATATGGTTTCTGTCTTTTAATTGCTCAGA
TATTGAGTCTGACTGTGTGACATGGGACAACTGTGTTCATGAGGCCTCTTTGGATTGCTTGCCTGGGCATGTTTTA
GATATGACTATGTCTGGACACAGTTACGATATATA
>dp68_PS .
GGGTCTGTTGATCTCAGAAGATGAGAATGGTTTGTGGATGCTGCCCCAATTGAGACAAGAGATGAGCAAGCACAAA
GTCTGTACAGCTTTTGAACACATACTACCACTTTCTTTCTTTCGTGAAGTAACCGATTATGTATCTTTTCATCAGA
AAATTAGAAATTCATTCGCAAATGTGGTTGTCATTTACGGTGACAATGAATTCATGTTCAATTTGTTAAAAAATTA
TTTCACAAGATCTTTAAGTCTAGGAACCGTCTGGGTCATAAACTCCCTTAGTACATTTCTGTCTGTGATGAAATAT
TTTTTGTTACATTCACTCCATGCACCTCTCATTTTCTCACACCACCATGGAGAGATTTCTGGTTTCAACAATTTTA
TCAAAACTGCTAACCCATCCAAATACCCAGAAGACTATTTCCTTGCTTTGGTGTGGATTCGCTCTTTTAATTGCAC
ATTGTCTGACTGCAGAGACTTGGGGCACTGTTCATACAATGCCACACTCCATTGGTTGCTGAGACACATTTTTGAT
ATGACCATGATGGAAGAGAGTTACTATATATA
>dp69 .
GGGACTGTTCATCACCGAAGATGACTATGGTGTGTGGTTCCTCTTAGAATTGAGAGATGAGATGGACAAGAATGGA
GTCTGTTTGGCCTTTGAGAACATGATCACTTATCATACCATTTCAGATTTATCAAATGATCTATCCATACATCGCC
AGCTAATGGAATCATCAACCAATGTGATTATCATTTATGGGGACAGTAAACTTCTCTTAAGCTTTATTCTTAAATT
AGGGCACTTTTTAATTATAGGGAAAGTCTGGGTCATGAACTCACCCTATGATGACGTGACCATTGGTAAAAAATAT
TTTCTGCTTGATTCTTTCCATGCACCTCTCATCTTCTCTCACCATCATAGAAACACTTCTGGGTTCACCCATTTTA
TTCTGACAGATACCTTGTCCAAGAACTCAGGAGATAATTCCCTTCCCAGGTCATGGATTCAGTCTGTTTATTGCCT
ACTGATTAAGTCTGACTGCAGAGACTTGGGGCACTGCTCATACAACGCTACACTCCATTGGTTGCTGAGACACATT
TTTGATATGACCATGTCAGAAGAGAGTTACAACATCTA
>dp71 .
GGGACTGTTCATCACCGAAGATGACTATGGTGTGTGGTTCCTCTCAGAATTGAGAAAAGAGATGGGCAAGAATGGA
GTCTGTTTTGCCTTTGCAAACACAATCACATACAATGCCATGCCAAATTTTGCAAATGATGCATTGATACATGGCC
AGATTACAGAATCATCAACCAATGTGATTATCATTCATGGAGACAGTGAATTTCTACTAAGCTTTATTCTTAAATT
AGGGGATTTTTTAATTACAGGGAAAGTCTGGGTCATGAACTCACCCTATGATGATGTGACCCTCAGTAAAAGGTAT
TTCCTGCTAGACACCTTTCATGCACCTCTCATCTTTTCTCCCCACCATGGGAATATTTCTGGGTTCACCAGTTTGA
TCCCATCAGCTGAGTATTCCAAGAACTCAGAAGATATTTCCAAACCTAGCTTATGGATTCAGGCTGTTCAGTGCAT
ACTGATTAAGTCTGACTGTACAAAATTGGGGCACTGTTTGTATAATACCACACTGGATTGGTTGCTGAGGCACATT
TTTGACACGGCCGTGTCAGAAGAGAGTTACAACATTTA
>dp74 .
CTCCAACACACCTCATATATATTCAAACTCATGGCAGTTCATAATGAGATAAATCTATCTTCAGCAAGTGTCCTCA
TCATATATGGTGATAAAGATTCACTGATAGCTTTAATGATATTTATCGAGCAAATTTTTATACATGGCAAAGTTTG
TATTGTGAACTCTCAGTGGGATTTCACCATGAAGAGGAGATATTTCATGCTAGGCTCATTGCATGTACCTCTTATT

TTTACACACCATCATGTGGAAGTTTCTGGATTCACAGATTTTGTCAAGGCAGTTAAGCCTTCCAAATACCCAGAAG
ACATTTTTCTTGCGAGGCTGTGGTTTCTGTCTTTTAATTGCTCAGATTCTGAGTCTGACTGTGTAACATGGGACAA
CTGTCTTCATGATGCCTCTTTGGATTGGTTACATGGGCATATTTTTGAAATTACTATGTCTGGAGACAGTTATAAT
ATATA
>dp76 .
GGGACTGTTGAACTTAGAGGATGAGAATGGTTTGTGGTCTCTTGCTGAATTGAAAGAAGAGATGGCCAAGAACAGA
GTTTGTGTAGAATATGAGCTAGTGATCTCAAGCCAATCAGTATCACATATGCTCCAAGCCATGGCAGTTTATAATG
ACATAAATAAATCTTCAGCAAATGTCTACATCATACATGGTGATAATGGTTCACTATTACCTTTAATTGTATGCAT
TGAGCAACTTTTAATAAAAGGCAAGGTTTTCATCATGAACTCACAGTGGGATTTCACCATGAAAAGGAAATATTTC
ATGGTAGGCTCATTGCATATACCTCTCATTTTTATGCACCATCGTGAAGATGTTTCTGGATTCACAGATTTTGTCA
AGGCAGTTAATCCTTCCAAATACCCAGAAGACATTTTCCTTGCTAGGATATGGTTTCTGTCTTTTAATTGTTCAAC
TCGTCAGTCTGACTGTGTGACATGGGACAACTGTCCTCATGATGCCTCTTTGGATTGGTTGCCTGGGCGTGTTTTT
GACATGACTATGTCTGGACACAGTTACAATATATA
>dp77 .
GGGCCTGTTCATCACCGAAGACAACTATGGTGTGTGGTTCCTCTCAGATTTGAGAAAAGAGATGGGCAAGATAGGA
GTCTGTTTTGCCTTTGAGAACATGATCACTTACAATGCCTTGGCAGATTACTCAGATGATCTTTCCATGTATCGCC
AGCTAATGGACTCATCAACCAATGTGATTATCATTTATGGGGACAGTAAATTTCTTCTAAGCTTTATTGTTAAATT
TGGGCATATTTTAATTATAGGGAAAGTCTGGGTCATGAACTCACCCTATGATGATGTCACCATTGGTAAAAGGTAT
TTTCTGATTCACTCCTTCCATGCATCACTCACCTTCTCTCATCATCACAGAAACACTGCTGGGTTCACCAATTTTA
TTCTGACAGAGACCTTCTCCAAGAACTCAGGAGATAATTCCCTTCCCAGGTCATGGATTCAATCTGTTTACTGCAT
CCTGATTAAGTCTGACTGCAGAGACTTGGGGCACTGTTCATATAATGCCACACTGCATTGGTTGCTGAGGCATATT
TTTGACATGACCATGTCATACGAGAGTTACAACATCTA
>dp81 .
GGGACTGTTCATCACTGAAGATGACTATGGTGTGTGGTTCCTCTCAGAATTGAGAGAAGAGTTGTACAAAAATGGA
GTCTGTTTGTCCTTTGAGACCATGATCACTTACACTGAACTTGCAGATTTATCAAATGATTTATGGATGCATCGCC
AGCTAATGGAATCATCAACCAATGTGATTATCATTTATGGGGATAGTAAATTTCTACTAAGCGTTATTCTCAAATT
TGGGCACTTTTTAATTACAGGGAAAGTCTGGGTCATGAACTCACCCTATGATGACGTGACCATTGGTAGATATTTT
CTGCTTGATTCTTTCCATGCTCCTCTCATCTTCTCTCACCATCATAGAAACACGTATGGGTTCACCAATTATATTC
TGACAGATACCTTGTCCAAGAACTCAGGATATAACTCCCTTCCCAAGTCATGGATTCAGTCTGTTTATTGCCTACT
GATTAAGTCTGACTGCAGAGACTTGGGGCACTGTTCGTACAATGCCACACTGGATTGGTTGCTGAGGCACATTTTT
GACATGACCATGTCAGAAGAAAGTTACAACATCTA
>dp88 .
AGGGCTGTTGATCTCACTAGATGAAAATGGTTTGTGGATAATTCCAGAATTGAGAGAAGAGATGGCCAAGAACAGA
GTCTGTGTAGCCTTTGAACATGTGGTTTCAAGCAATCTGCTGACACAAGCACTAAACTTCATGATAATCCATAACC
AGATTACCAGATCATCAGCAAATGTATTCATCATTTATGGTGATAATGAATACCTTCTAAACTTAAGGCTGTATTT
TGGGCAATTTTTAATGACAGGCAAGGTTTGGCTTATGACTTCACAGAGTGATTTATCCTTCAGTAGGAGATATTTC
ATTTTAGGTTCATTCCATGTCCCTCTCATGTTCTCACACCACCATTCAGACATTTCTTGGTTTAAGAACTATGTCA
GGGAAGTTAATCCTTCCAAATACCCAGAAGACTTTTACCTTGCTAGGCTGTGGGTTCTCTCTTTTAATTGCTCACA
TTCTGAGTCTGACTGTGTAACATGGGAGAACTGTCCACAAGATGCCTCCTTAGAGTGGTTGCCTGGACACATTTTT
GACATGGCCATGTCTGAAGAGAGTTATAATATATA
>dp91 .
GGGACTGTTCACCTCACAAGATGAGAATGGCTTGTGGATTCTTCCTGAATTGAAAGAAGAACTGGCCAAGAACAGC
GTTTGTGTAGCCTATGAGCTACTGATATCAAGCAAAGGCATTGCACATATAATCAGAATCATGGCAGTTTATAACG
AGATAAATAAATCTTCAGCAAATGTCCTCATTATATATGGTGACAATGAATCACTACTAGATTTAATGCTATTTAT
TGAGCACATTTTAATGAAAGGCAAAGTTTGTATCATGAACTCACAATGGAATTTAAAAACAAGGAGATATTTCATG
TTAGGCTCATTGCATGTACCTCTTATTTTTACACACTATCATGCAGATGTTTCTGGATTCACAGATTTTGTCAAGA
CAGTTAATCCTTCCAAATACCCAGAAGACTTTTTTCTTGCAAAGTTGTGGTTTCTCTCTTTTAATTGCTCAGACAT
TGAGTCTGATTGTATAGCATGGGACAAATGTCCTCATGATGCCTCCTTGGATTGGTTGCCTAGGCATATTTTTGAC
ATTTCCATGTCTGGGGACAGTTACAACATATA
>dp92 .
GGGACTATTGACCACACAAGATGATAATGGGGCAAAGATTCTTGCTGAATTGAAAGTAGAGATGGCCAAGAACAGA
GTTTGTGTAGCCTATGAGCTAGTGATCGCCACCCAAGATGCATATAACCTCATAACAGTTAATCGTTATATTAATC
TATCTTCAGCAAGTGTCCTCATCATATATGGTGATAATAATTCACTGATGGTTTTTATGGTATTTGTTGAGCAAAT
TATTATACATGGGAAAGTTTGTATCATGAACTCTCAGTGGGATTTCATCCTGAAAAGGAGATATTTCATGTTAGGA
TCATTGCATGTACCTTTTATTTTTACACACCATCATGTAGATGTTTCTGGATTCACAGATTTTGTCAAGGCAGCTA
AGCCTTCCAAATACCCAGAAGACATTTTTCTTGCAAGGCTGTGGTTTCTCTCTTTTAATTGCTCTGATTCTGTATC
TGACTGTGTAACATGGGACAACTGTGCTCATGATGCCTCTTTGGATTGGTTACCTGGGCATATTTTAGACATGACT
ATGTCTGTAGATAGTTACAATATATA
>dp94 .
GGGACTGTTGACATCAGAGGATGAGAATAGTTTGTGGGTTCTTGTTAAATTGAAAGAAGAAATGGCTAAGAACAGA
CTTTGCGTAGCCTATGAGCTAGTGATGTCAAGCAAGTTCGTCTCGCAGATATATAAATTCATGACAATATATAGTC
AGATAAATAAATCTTCAGCAAATGTCCTCATGTTATACGGTGATAATGAGTCACTACTAGATTTAATGATGTGTTT

TGAGCAACTTTTAATAAAAGGAAAAGTTTGCATCATGAACTCACAGTGGGATTTAACCATGAGAAGGAACTATTTC
ATGCTAGGCTCATTCCATGTACCTCTCATTTTTTCACACCATCATGCAGATGTTTCTGGATTTACAGATTTTATCA
AGGCAGTTAATCCTTCCAAATACCCAGAAGACTTTGTTCTTGCTAGGCTGTGGTTTGTGTCTTTTAATTGTTCTGA
TTCTGAGACTGACTGTGTAACATGGGACAATTGTCTTCATGATGCCTCTTTGGATTGGTTGCCTGGGCATATTTTT
GACAGGACTCTGTCTGGAGACAGTTATAATATATA
>dp95 .
AGGACTGTTGACCTCACAGGATGAGAATGGTTCATGGGTTCTTGCTGAATTGAAAGAAGAGATGACCAAGAACAGA
GTTTGTGTGGCCTATGAGCTACTGATCTCAAGCAAAGACACATCACGTATATTCAAATTAATGGAAGTTTATAACA
AGATAAATAAATCTTCAGCAAGTGTCCTCATCATATATGGTGATAATGAATCACTACTAGATTTAATGGTGTGTAT
TGAGCAACTTTTAATCAAAGGCAAAGTTTGTATCATGAACTCACAGTGGGATTTAACCATGAGAAGGAAATATTTC
ATTCTAGGCTCATTGCATGTTCCTCTTATTTTTTCACATCATCATACAGATCTTTCTGGATTCACAGATTTTATCA
AGGCAGTAAACCCTTCCAAATACCCAGAAGACATTTTTCTTGCTAGGATGTGGTTTCTGTCATTTAATTGCTCAGA
TGCTGACTCTGACTGTGTAACATGGGACAATTGTCCTCATGATGCCTCTTTGGATTGGTTGTCTGGGTATATTTCT
GTCATGACTATGTCTGGAGATAGTTACCATATATA
>dp96 .
TTGATCTCACAAGATGAGAATGGCTTATCGATCCTTTCAGAACTGAGAGAAGCAATGGACAAGAACAGAATCTGTG
TAGCCTTTGAGCATGTGATCTCTGGCAGTACTGTGACACAAGTACTTAACATCATGGCAATTCAGAACCAGATTAA
TAAATCATCAGCAAATGTACTTATCATTTATGGGAGTAAGGAATACCTGTTAGACTTAATGGCATATTTTGGACAA
TTCTTAATAACAAGCAAAGTTTGGCTCTTGAATTCACAATGTGATTTATCCATTGGAAGGAAGTATTTCATGTTAG
GCTCATTCCATGTGCCTCTCATCTTTTCATATCACCATACAGACATTTCTGGATTCACAAATTTTATCAGGGAAGT
CAACCCTTCCAAATACCCAGAAGACTTTTACCTTGCTAAGTTGTGGTTTCTCTCTTTTAATTGCTCCCATTTTGAG
TCTGACTGTGTAATGTGGGAGAACTGTTCACATGATGCCTCCTTGGAATG
>dp99 .
GGGACTATTGACCACACAAGATGATAATGGGGCAAGGATTCTTGCTGAATTGGAAGTCGAGATGGCCAAGAACAGA
ATTTGTGTAGCCTATGAGCTAGTGGTCTCCACCCAAGATGCATATAACCACATAGCAGTTAATCGTTATATTAATC
TATCTTCAGCAAGTGTCCTCATCATATATGGTGATAATAATTCACTGATGATTTTTATGATATATATTGAGCAAAT
TATTACACATGGGAAAGTTTGTATCATGAACTCACAGTGGGATTTCTCCCTCAAAAGGAGATATTTCATGATAGGA
TCATTGCATGTACCTCTTATTTTTACACACCATCATGTAGATGTTTCTGGATTCACAGATTTTGTCAAGGCAGTTA
AGCCTTCCAAATACCCAGAAGACATTTTTCTTGCGAGGCTGTGGTTTCTCTCTTTTAATTGCTCTGATTCTGTGTC
GGACTGTGTAACATGGGACAACTGTGCTCATGATGCCTCTTTGTATTGGTTACCTGGGCATAGTTTTGATGTGAGT
ATGTCTGTAGACAGTTACAATATATA
>dp100 .
GGGTTCTTGTTAAATGGAAAGAAGAAATGGCCAAGAACAAAGTTTGTGTAGCCTATGAGCTAGTGATCTCAAGCAA
AACCGTCTCACAGATGTTCAAATTAATGGCAGTTTATACTCAGATGAATAAATCTTCAGCAAATGTCCTCATGATA
TATGGTGATAACGAGTCACTACTAGATTTAATTATGTGTATTGAGCAACTTTTACTAAAAGGAAAAGTTTGCATCA
TGAACTTACAGTGGGATTTAACCTTAAGAAAGAATCGTTTCATGCTAGGTTCATTCCATGTACCTCTCATTTTTTC
ACACCATCATGCAGATGTTTCTGGATTCACAGATTTTATCAAGGCAGTTAATCCTTCCAAATACCCAGAAGACTTT
CTTCTTGCTAAGCTGTGGTTTGTGTCTTTTAATTGTTCTGATTCTGAGTCTGACTGTGTAACATGGGACAATTGTC
TTCATGATGCCTCGTTGGATTGGTTGCCTGGATATATTTTTGACAGGACTCT
>dp101 .
GGGACTGTTCATCACTGAAGACAACTTTGGTGTGTGGTTCCTCTCAGATTTGAGAAAAGAGATGGGGAAGATAGGA
GTCTGTTTTGCCTTTGAGAACATGATCACTTACAATGCCTTGGCAGATTACTCAGATGATCTTTCCATGTATCGCC
AGCTAATGGACTCATCAACCAATGTGATTATCATTTATGGGGACAGCAAATTTCTTCTAAGCTTTATTGTTAAATT
TGGGCATATTTTAATTACAGGGAAAGTCTGGATCATGAACTCACCCTATGATGATATCACCATTGGTAAAAGATAT
TTTCTGATCCACTCCTTCCATGCATCTCTCACCTTCTCTCATCATCACAGAAACACTGCTGGGTTCACCAATTTTA
TTCTGACAGAGACCTTGTCCAAGAACTCAGAAAATAATTCCCTTCCTAGGTCATGGATTCAGTCTGTTTACTGCAT
CCTGATTAAGTCTGACTGCAGAGACTTGGGGCACTGTTCATACAATGCCACACTGCATTGGTTACTGAGGTATATT
TTTGACATGACCATGTCGGACGAGAGTTACAACATCTA
>dp102 .
GGGACTGCTAACCTCACAAGATGAGAATGGTATGTGGATTCTTCCTGAATTGAAAGAAGAGATGACCAAGAATAGC
GTTTGTGTAGCCTTTGAGCTAGTAATCCCAAACTATGCCCTGACATTAAAATTCATGACACTTTATGACCAGATTA
ATAAATCATCAGCCAATGTCCTCATCATATATGGTGATAATGAATTCATACTACACTTAATGATGTGTGTTGAGGA
ATATTTCATAAAAGGAAAAGTTTGCATCATGAACTCACACTGGGATTTAACCACAAGGAGGAGATATTTTATGCTA
GGTTCAATCCATGTTCCTATCATTTTTTCACCCCATCACCCAGATATTTTAGGTTTCACAGATTTTGTCAAGGCAG
TTAACCCTTCCAAATACCCAGAAGACTTTTTCCTAGCGAGGCTGTGGTTTCTGTCTTTTAATTGCTCAGATTCAGA
GTCTGACTGTGTATTGTGGGAGAATTGTCCTCATGAAGCCTCCTTCGACTGGCTGCCTGGACACATTTTTGACACG
ACTATGTCTGGAGACAGTTACCATATATA
>dp3_PS .
GGGACTGTTTATCTCAGAAGACGACTACAGTGTGTTGTTCCTCTCAGAATTGAGAGAAGAGATGGGCAAGAATGGT
ATCTGTTTGGCCTTTGAGAACATGATAACTTACAAAGCCATTTCAGATATCTCAAATGATCTGTCAATGCATCTCC
AGCTAATGGAATCATCAACCAATGTGATTATCATTTATGGGGACAGTAAATTTCTATTAAGCTTTATTATTAAATT
TGGGCACTTTTTAATTACAGGGAAAGTCTGGGTCATGAACTCACCCTATGAGGACGTGACCATTGGTAAAAGATAT

TTTCTGATCAACTCCTTCCATGCACCTCTCATCTTCTCTCACCATCTTAGAAACACTTCTGTGTTCACTAATTTTT
ATTCTGACAGATACATTGTCCAAGAACTCAGGAGTTAATTCCCTTTCCAGGGCATGGATTCAGTTTGTTTACTGCC
TACTGATTAAGTCTGAATTCAGAGACTTGGGGCACTGTTCATACAATGCCACACTGGATTGGTTGCTGAGGCACAT
TTTTGACATGACCATGTCAGACAAGAGTTAAAACATCTA
>dp4_PS .
GGGGCTGGTCATCTCAGCAGATGAGAATGGATTGTGGATGCTCCCAGCGCTGAGAGAAGAGATGGACAGAAATGGA
ATCTGCAGTGGTTTTGAAATGGTGTTGGCAGTCAACTCCATGAGATCGATGTCCAAGTCTTTACTACTCCTGTATG
AAATTAGAAAGTCAACGACAAATGTAATTGTTTTCTATGGTAACAGTGGTTTCCTGGAAACTGCAGTGAATGCAGA
AACTTTGGACAACTCTTTCCTGGCATGAAAAGTTTGGCTCATGAACTCACTCATCCTGGCGATCCTCATAGGGAGA
TATCACATGGCTGCACACTTCCACTCCCGCCTCCTTTTTATACATCACTACGGAGACGTTCCTGATTCACAACTTT
TATCCATGACAATTTACCCTTCCAAGTATCCTAAATACTATCTCCTCGCTAGGCTGTGGGTCATGGCTTTTAATTG
CTCCTTGTCCAAGTCTGACTGTGTGACTTGGCAGAACTGTCCACACAACGCCTCCTTTGAATGGCTGTCTGCACAC
TTTTTGGACATGACCATGTCAGAAAAGAGTCACCATGTCTA
>dp10_PS .
GGGTCTGTTGATCTCAGAAGATGAGAATGGTTTGTGGATGCTGCCCCAATTGAGACAAGAGATGAACAACTACAAA
ATCTGTACAGCTTTTGAACACATAATACCACTTTCTTTCTTTCGTGAAAGAACTGATTATGTATCTTTTCATCAGA
AAATTAGAAATTCATTCACAAATGTGGTTGTCATTACGGTGACAATGAATTCATGTTCAATTTGTTAAACACTTAT
TTCACAAGATCTTTAAGTCTAGGATTCGTCTGGGTCATAAACTCCCTTAGTACATTTCTGTCTGTGATGAAATATT
TTTTGTTACATTCACTCCATGCACCTCTCATTTTTTCACACCACCATGGAGAGATTTCTGGTTTCAACAATTTTAT
CAAAACTGCTAACCCATCCAAATACCCAAAAGACTATTTCCTTGCTTTGTTGTGGATTCGCTCTTTTAATTGCACA
TTTTCTGAGTCTAGCTGTGTCACTTGGAAGAACTGTCCAGCCAATGCGTCCTTGGAATGGTTGCCTAGGTACCTTT
TTGACATGGGCATGATGGAAGAGAGTTACTATATATA
>dp11_PS .
GGGACTGCTCATAACCAAAGACAACTATGGTGTGTGGTTCTTCTCAGAATTGAGAGAGGAGATGCAATAGAATGGA
GTCTGTCTTGCCTTTGAGAATATGATCCTGACAAACAGCCCATCAGATTTACCAACTGATCAACCAAGGTGTCGCC
AGATCATGGAATCCTCAACCAATGTGATAATCATTTATGGAGAAATTGAATTGCTACTAAGCTTTATTTTTAAATT
GGGAATTTTTTAAATACAGAGAAAGTCTGGGTGATGAACTCACCCTATGATGACATGACCCTTAGCAAAAAATACT
TCTTGCTAGACTCCTTCCATGCACCTCTCATCTTCTCTCACCACCATAAAGACATTTCTGGGTTCACCAATTGTGT
CCCAAGAGATAACGATTCCAAGGACTCATGAGATATTTCAGTTCCTAGGTTATGGATTTGGGCTGTTCAGTGAATA
AGTCTGATTATAGAAAATTGGGGTAGTCTTCATCCAATGCCACTGTGGCTTGGTGCAGGGACACACTTTTGATATG
GCCGTGTCACAAGAAAGTTACAATATCTA
>dp13_PS .
GGGGCTGGTCATCTCAGCAGGTGACAATGGACTGTGGATGCTTGCAGCGCTGAGAGAAGAGATGGACAGACATGGA
TCCACATATGTTTTGAAGCGGTGGCCTCTGTCCATTCATTAGATACTGAATCCCAATATCTACTACTCCAACATGA
AATGACAAAGTCATCCCCACACGTGATTGTCATCTACGGTAACATTTTCTTTCTGGAAATTGTTTGGAACGTTTAA
TATGTTGAGCAAAATTTTCATTGTAGGAAGAGCTTGGCTCACGACCTCTCTTGGCATCTGGAGCGTCAGAGGAGGC
ATTTCATATGAGACCAGTTGCACACCGACGCCCATCTCATCTTTTCTCATCACCGTGGAGACGTTCCTGAATTCAC
AACTTTTATCCAGACAATTTACCCTTCCAAATACCCAGAAGACTATCTCCTCACTGGGCTGTGGGTCATGGCTTTT
AATTGCTCCTCGTCCAAGTCTGACTGTGTGACTTGGCAGAACTGTCCACACAACGCCTCCTTTGAACGGCTGCCTG
CACACTTTTTGGACATGACCATGTCAGAAAAGAGTCACCATGTCTA
>dp16_PS .
GGGTCTGTTGATCTCAGAAGATGAGAATGGTTTGGGGATGCTGCCAAAATTGAGAGAAGAGATGAACAGGCACAAA
GTCGGTGTAGCTTTTGAACACATATTACTATATTCTTTCTTTGACAAAATATTTGAACTTGTATCCTTTCATGAGA
AAATTAGAAATTCATTCACAAATGTGGTTCTTATTTACAGTAACTATAAATTCTTGTTCAATTTGTTAAACATTTT
TTTAACAGATTATTTAACTCTAGGAACATCTAGGTCATAAACTGCCATGATACATTTATCTATGTGATGAAATATT
TTTTGTTACATTCACTCCATCCATCTCTCATTTTCTCACACTACCATGTAGAAATTTCTGGTTTCAACAATTTTAT
CAGAACAGCTAACCCATCCAAATACCCAGAAGACTATTACTTTGTTTTGGTGTGGATTTAATCTTTTAATTGCACA
TTTTCTGAGTCTAGCTATGTAACTTAGAAGAACTGTCTACCCAATACCTCCTTGGAATGGTTGCCTAGGTACCTTT
TTGACATGGAAGAGAGTTACAATATATA
>dp23_PS .
GGGACTATTAACCTTAGTAGATTTTGGGTTCATCCTGCATTGAAAGAAGAGATGGCCAAGAACAGAGTTTGTGCAG
TTTTTCAGGTAGTGATCCTAAATTTTATCTTGTCCTATATATTAAAATATATGGCACATTATAACCAGATTAATAA
ATCCTCAGCCAATGTCATCATCACATATGCTGAAAATGAATCCCTACTATAGATTATAATGTGTACTGATCAATTT
TTAATGCAAGGAAAAGTTTTTATCAGGAACTTAACTATGAAAAAAACATATTTTACTCTAGGTTCATTCCATGTAC
CTCTCATTTTTTCACATCATCACCCAGATGTTTCTGGATTCACAGATTTTGTCAAGGCAGTTAACCCTTCCAAATG
CACAGAAGACTTTTTCCTTGTTAGGCTGTGGTTTCTCTATTTCAATTGCTCAGATTCTAAGTCTGACTGTATAATA
TGGGAGAACTGTCCTCATGTTGCCTCCTTGGACTGGTTGCCTAGACACACATTTGACAAGACAATGTCTAGGGACA
GTTACACTATCTA
>dp27_PS .
AGGACTGCTGACCTCACAAGATGAGAATGCTTCATGGGTTCTTGCTGAATTGAAAGAAGAGATGGCCAAGAACAAA
ATTTGTGTAGCCTATGAGCTAATGATCTCAACCCAAGACGCATCACATATATACAAACTCATGGCAGTCTATAACC
AGATAAATCTATCTTCAGCAAGTGTCCTCATCATATATGGTGATAATGATTCCCTGATAGCTTTAATGGTATTTAT

TGAGCAAATATTTATGCATGGTAAAGTTTGTATCGTGAACTCACAGTGGGATTTCACCCTGAAAAGGAGATACTTC
ATGCTAGGCTCATTGCATGTACCTTTTATTTTCACACATCATCATGTAGATGTTTCTGGATTCACAGATTTTGTCA
AGGCAGTTAAACCTTCCAAATACCCAGAAGACATTTTTCTTGCAAGGCTGTGGTTTCACTCTTTTAATTGCTCAGA
TGCTGAGTCTGACTGCATAACATGGGACAATTGTGCTCATGATGCCTCTTTGGATTTGTTGCCTGCATGCATTTTT
GACATGACTATGTCTGTGGATAGTTACAATATATA
>dp28_PS .
GGGACTGCTGACCTCACAAGTTGAGAATGGTCCATGGGATCTTGCTGAATGGAAAGAAGAGTGACCAAGAACAGAG
TTTGTGCAGCCTATGAGCTAGTGATTTTAAGCCAAGACATGTCACATATATTCAAACTCATTGCAGTTCATAATCA
TAATGAGATAAATCTATCTTCAGCAAGTGTCCTTATCATATATGGTGATAATGATTCCCTGATAGCATTAATGATA
TTTATTGAGCAAATTTTTATACATGGCAAAGTTTGGATCATGAACTCTCAGTGGGATTTTACCAGGAGGAGGAAAT
ATTTCGTGTTAGGCTCATTGTATGTAACCTCTGATTTTTACACCCCGTCAGTTGAAGTTTCTGGATTCACAGATTT
TGTCAAGGCAGTTAAGCCTTCCAAACACCCAGAAGACATTTTTCTTGCCAGGCTGTGGTTTCTGTCATTTAATTGC
TCAGATTCTGTGTCTGACTATGTGACATGGGACAATTTTGCTCAGGATGCCTCTTTAGATTGGTTGCCTCGGCTCA
TTTTTGACATGACAATATCTGCAGACAGTTACAATATATA
>dp47_PS .
TGGACTGTTCACTTCACAAGATGAGAATGGCTTGTGGATGCTTCCTGAGCTTAAAGAAGAAATGGCCAAGAATAGA
GTTTGTGTAGCCTATGAGCTACCAATATCAAGCAAAGGCACTGAACATATAGTCAGAATTATGGCCGTTTATAATG
AGATAAATAAATCTTCAGCAAATGTCCTCATCATTTATGGTGATAATGAATCACTATTAGATTTAATGCTGTTTGT
TGAGCAATTTTTAATAAAAGGCAAAGTTTGTATCATGAACTCACAGTGGGATTTAATAACGAGGAGATATTTCATG
CTAGGCTCATTCCCTGTACCTCTTATTTTCTCACACCATCATGCAGATGTTTCTGGATTCACAGATTTTGTCAAAG
CAGTTCACCCTTCCAAATACCCAGAAGACTTTTTTCTTGCAAAGCTGTGGTTTCTCTCTTATAATTGCTCAGATGC
TGACTCTGACTGTGTATCATGGGACAGTTGTCCTCGTGATGCCTCCTTGGATTGGTTGCCTAGGCACATTTTTGAC
ATTACCATGTCTAGAGACAGTTACAATATATA
>dp50_PS .
GGGATTGTTGACCTCAGATGATGAGAATGGTTTGTGGTTTCTTCCTGAACTGAAAGAAGAGATGGCCAAGAACACA
GTTTGTGTAGCCTTTGAGCTAGTGATGCCAAACCATGCCCTGTCACATGTATTGAAACTCATAGCAGTTTATAATG
AGATTAATAAATCTTCAGCAAATGTCCTCATCATATATGGTGATAATGAATCCCTACTAGATTTAATGTTGTGTGT
TGAACAATTTTTAATTACAGGTAAAATTTGTATCATGAATTCACAGTGGCATTTAACCACAAAAAGGAAATATTTC
ATGCTAGGCTCTTTCCATGTACCTCTCATTTTTTCACACCATCATGCAGAAAGTATTGGATTCACAGATTTTATAA
GGGCAGTTAACCCTTCCAAATACCCAGAAGACTTTTACCTTGCTAGGTTGTGGTTTCTCTCTTTTAATTGTTCAGT
TTCTGACTCTGACTGTGTGACATGGGAGAACTGTCCACATGATGCCTCCTTGGAATGGTTGCCTGGACACACTTTT
GACATGGCTATGTCTGAAGAGAGTTACAATATCTA
>dp59_PS .
AGGACTGTTAACTACACAAGATGAGCATGGTACATGGGTTCTTGCTGAATTGAAAGAAGAGATGGCTAAGAATAGA
GTTTGTGTAGCCTATGAGCTAATGATTTCAACCCAAGATGCATCACATATATTCAAACTCATGGCAGTTCATAATG
AAATAAATCTACCTTCAGCAAGTGTCCTCATCATATATGGTGATAATGATTCACTGATATTTTTAATGGCATTTAT
TGAGCAAATTTTTATACATGGCAAAGTTTGTGTCATGAACTCACAGTGGGATTTCACCCTGAAAAGGAAATATTTC
ATGATAGGCTCATTGCATGTGACTCTTATTTTTACACACCACCATGTAGGTGTTTTTGGATTCACAGATTTTGTCA
AGACAGTTAAGCCTTCCAAATACCCAGAAGATATTTTTCTTGCCAGGCTGTGGTTTCTGTCATTTAATTGCTCTGA
TTCTGAATCTGACTGTGTGACATGGGACAATTGTCCTCATAGTGCCTCTTTAGATTGGTTGCCTGGACATATTTTT
GACATGACTATGTCTGGAGACAGTTACAATATATA
>dp70_PS .
GGGAGTATTGGCCACAGAGAATGAGCATAGTACATGATTTCTTGATGAATTGTAAGAAGAGATGGCCAAAAACAGA
GTTTGTGTAGCCTATGAGTTAATGATCTCAACCCAAGAGGTGGCATATGTATTCAAACTCATGGCAATTCATAAAG
AGATAAATCTATCTTCAGCAAGTGTCCTCATCATATATGGTGATAATGATTCACTGATAGCTTTAATGGTATTTAT
TGAGCAAATTTTTATACATGGCAAAGTTTGTATCATGAACTCTCAGTGGGATTTCACCATGAAGAGGAAATATTTC
ATGCTAGGCTCATTGCATGTACCTCTGATTTTTACACACCATCATGTAGATGTTTCTGGATTCACAGATTTTGTCA
AGGCAGTTAAGCCTTCCAAATACCCAGAAGACATTTTTCTTGCCAGGCTGTGGTTTCTTTCTTTTAATTGCTCAGA
TTCTGAGTCTGACTGTGTGACATGGGACAACTGTGCTCATGATGCCTCTTTAGATTGGTTGCCTGGGCATATTTTT
GAAATGACTATGTCTGGAGACAGTTACATATATA
>dp72_PS .
GGGACTGTTCATCACTGAAGATGACTATGGTGTGTGGTTCCTCTCAGAATTGAGAGAAGAGATGGACAAGAATGGA
GTTTGTTTGGCCTTTGAGAACATGATCACTTACAGTGCCTTTTTAGATTTCACAAATGATCTATGGATGCATCGCC
AGCTAATGGAATCATCAACCAATGTGATTATCATTTATGGGGACAGTAAATTTCTACTAAGCTTTATTCTTAAATT
TGGGTACTTTTTAATTACAGAGAAAGTCTGGGTCATGAACTCACCCTATGATGATGTGACTATTGCTAAAAGACAT
TTCTCATTGTGA
>dp73_PS .
GGGACTGTTCATCACCGAATACGACTATGGTGTGTGGTTCCTCTCAGAATTGAGAGAGGAGATGGGCAGGAATGGA
GTCTGTTTTGCCTTTGAGAACACAATCCAGACCAATGCAGTCTCCGATTTCTCAAGTGATCATCAGTGTATCGCCA
TAATTATAGATCCTCAACCAATGTGATTATCATTATGGAGACAGTGATTTTCTACTAGCTTTATTCTAATAAGGCC
ATTTTTAATACAGGGAAAGTCTGGGTCATGAACTCACCTTATGATGATGTGACCATCAGTAAAACCTATTTCCTGA
TAGATTCTTTCCATGCACCTCTCATCTTCTCTCACCATAGAAACAATTCTGGGTTCACCAATTTTATCCCAAGAGC

TAACTTTCCCAAGAACTCAGAAGATATTTCTCTTGCTAGGTTATGGATTCAGTCTATTCGTTGCATACTGATAAAA
TCTGACTGTACAAAATTGGGGCACTGTTCATACAATGCCACACTGCACTGGTTGCTGGGGCACATTTTTGACATGT
CCTTGTCAGAAGAGAGTCACAACATCTA
>dp78_PS .
GGGACTGTTCATAACCCAAGATGACTATGGTGTGTGGTTCCTCTCAGAATTGAGAGAAGAGATGTGTATAAATGGA
GTCTGTCTGGCTTTTCAGAACATGATCACTTACAGTGCTTTTTCAGATTTCTCAAATGATCTATGGATGCATTGCC
AGCTAATTGCCTAGACTCATCAACAAATGTGATTATCATTTATGGAGACAGTAAATTTCTACTAAGCTTTATTCTT
AAATTTGGGCACTTTTTAATTACAGGGAAAGCCTGGGTCATGAACTCACTATATGGTGATGTGACTATTGGTAAAA
GATATTTTCTGCTTGATTCTTTCCATGCTACTCTCATCTTCTCTTACCATCGTAGAAACAATTATGGGTTCACCAA
TTATATTCTGACAGATACCTTGTCCAAGAACTCAGGAGATAATTCCCTTCACAGCTCATAGATTCAATCTGTTTAT
TGCCTACTGATTAATTCTGACTGCAGAGATGTGGGACACTGTTCATACAATGCCACACTGCATTGGTTGCTGAAGC
ACATTTTTTACATGACCATGTAAGACGAGAGTTACAACATTTA
>dp80_PS .
GAGACTGTTTATAACCCAAGATGACTATGGTGTGTGGTTCCTCTCAGAATTGAGAGAAGAGATGTGTATAAATGGA
GTCTGTCTGGCTTTTCAGAACATGATCAAGAAATAATCTATGGATGCATTGCCAGCTAATTGCCTAGACTCATCAA
GTGTGATTATCATTTATGGAGACAGTAAATTTCTACTAAGCTTTATTCTTAAATTTGGGCACTTTATAATTACAGG
GAAAGTCTGGGTCATGAAATCACTCTATGATTATGTGACTATTGGTAAAAGATATTTTCTGCTTGATTCTTTCCAT
GCTACTATCATCTTCCCTTACCATCGTAGAAATACTTATGGGTTCAACAATTATATTCTGACAGATACCTTGTCCA
AGAACTCAGGAGATAATTCCCTTCACAGCTCATAGATTCAATCTGTTTATTGCCTACTGATTAATTCTGACTGCAG
AGACGTGGGGCACTGTTCATGCAATGCCACACTGGATTGGTTGCTGAGGCAGATTTTTGACATGAGCATGTCAGAC
GAGAGTTACAACATCTA
>dp83_PS .
GGGACTGTTGTCTACACAAGATGAGAATGGTGCATGGGTTCTTGCTGAATTGAAAGAAGTGATGGCCAAGAACAGA
GTTTGTGTAGATGAGATAAACCTAGCTTCAGCAAGTGTCCTCATCATAATAATTCACTGCTAGGTTTAATGATATT
GATGGAGCAAATTATTATACATAGGAAAGTGTGAATCATGAACTCACAGTGGGATTTCACCCTGAAAATGGGATAT
GTCATGTTAGGATCATTGCATGTACGTCTTATTTTTACACATCATCATGTAGATCTTTCTGGATTCACAGATTTTG
TCAAGACAGGTAAGCCTTCTAAATATCCAGAAGACATTTTACTTGGGAGGCTGTGGTTTCTCTCTTTTAATTGCTC
TGATTCTGAGTTTAACTGTGTAACATGGGACAATTGTTCTCTTGATTCCTCTTTGGATTGGCTACCTGGACACATA
TTTGAAATGACTATGTCTGTAGACAGTTACAACATATA
>dp84_PS .
GGGACTGTTCATCACCAAAGACAACTATGGTGTGTGGTTCCACTCAGATTTGAGAGAAGAGATGGGCAATAATGGA
GTCTGTTTTACCTTTGAAAACAAGATCACTTACAGTGCCCTTTCAGATTTCTCAAATGATCAGTTGATGCAATGCC
AGCTAATGGACTCATCCACCAATGTGATTATCATTTATGGGGGCAGTAAATTTCTACTAAGCTTTATTCTTAAATT
TAGGCAAATTTTAATTACAGAAGAGAGTTACAACATCTACAATGCTGTGTATGTTGTGGCCCAGGCCCTCCACCAG
ATGTTTCTTCATCACAAGTAAGAGCCACCAGTGCACAATGGGCAACCCAGGGCATTTCTCTCTTCACAGGTAATGG
CTTTGCTTTTGGGTGACCTACTCTAGGCATGCAGACTTTCTATAGAAGCTTTCATAGCAGATGGAACCAAGTTGTC
TGGCTCCATTGTGGAAAATACAAGACACTCTGGGCCTGTTGTGCTCTCCATGAAGTGATTGTAGAGCTGTGAAGAC
AATGTTCTGAAGAACACTGAATCGGTGACAAGGAAGTGCTGTGT
>dp85_PS .
GGGACTCCGCATAAGCAAAACGACTATGGTGTGTGGTTCCTCTCAGAATTGAGAGAGGAGATGGACAAGAATGGAA
TCTGTCTTGCCTGTGAGAACATGATCCTGACAAACATACTATAAGATTTATCAAGTGATTGACCAAGATATCGCTA
GATCATGGACTCTTCAAGCAATGTGATAATCACTTATGGAGAAATCGAATTGCTACTAAGCGTTATTCTTAAATTT
GGGGATTTATTAATTACAGAGAAAGTCTGGGTCATGAACTCACCCTATGATGACATTGACCCTTAGAAAAGATACT
TCCTGCTAGACTTCTTCCACGCACCTCTCATCTTCTCTCACCACCATACAGACATTTCTGGGTTCACCAATTTTGA
GATAACTATTCCAAGAATTCAGGAGATATTTCTCTTCCTAAGTTATGTATTTGGGCTGTTCAGTGCATACTGATTA
GGTCTGATTATAGAAAATTGGGGCAGTCTTCATCCAATGCCACTGTGGCTTGGTTACAGGGACACACTTTTAATAT
GGTCATGTCACAAGAGTTACAACATCTA
>dp87_PS .
GGGAATGTCCATCATGGAAGATGACTATGGTGTCTGGTTTCTCTCAAAATTGAGAGAAGAGATGGACAAGATTGGA
GTCTGCTTGCCTTTGAGAACACCATCCAGACAAATGGCACCTCAGATTTATCAAGTGATCGACCAAGATATCGCCA
GATCACGGAATCTTTAACCAATATGATTATCATTTATGGGAATATTAAATTGCTACTAAGCTTTATTCTGAAATTT
GGGGATTTTTAAATTACAGAGAAAGTCGGGCCATGAATTCACCCTATGATGACATGACCCTCAGCAAAATATATTT
CCTACTAGACTCCTTCCACAAACCTCTCATCTTCTCTCACCACTATAGAGACATTTCTGGGTTCACCAGTTTTGTC
TCAAGGGATAACTATTCCAAGGACTCAGAAGATATTTCCCTTCCTAAGTTATAAATTCAGGCTCTTTAGTGCATAC
TGATTAAGTCTCACTGTACAAATTTGGAGCACTGTTCGTACAATGCCACTGTGGATTGGTTGCAGGGACACCTTTT
TCACATGGCCATGTCAGAAGAGTTACAATCATCTA
>dp89_PS .
GGGTCTGTTGATCTCAGAAGATGAGAATGGTTTGTGGATGCTGCCCCAATTGAGAGAAGAGATGAACAAGCACAAA
ATCTGTACAGCTTTTGAACACATAATACCACTTTCTTTCTTTCATGAAGCAACTGTATTTCTACCCTTTCATCGGA
AAATTAGAAATTCACTCACAAATGTGGTTCTCCTTTATGGTGACAATGAGTTTATGGTCAACTTGTTAAATGTTTT
TTAAGGTTTTTAAGTCTAGGAACCGTCTGGGTCATAAACTCCCTTAGTACAATTCTGTACGTGATGAAATATTTTT
TGTTACATTCACTCCATGCCCCTCTCGTTTTCTCACACCACCATGGAGAGATTTCTGGTTTCAACAATTTTATCAA

AACTGCTAACCCATCCAAATACCCAGAAGACTATTTCCTTGCTTTGGTGTGGATTCACTCTTTTAAGTGCACATTT
TCTGAGTCTAGCTGTGTCACTTGGAAGAACTGTCCAGCCAATGCGTCCTTGGAATGGTTGCCTAGGTACCTTTTTG
ACATGGGCATGATGGAAGAGAGTTACTATATATA
>dp93_PS .
GGGACTGTTGACTTCAGAGGATGAGAATGGTTTGTTGGTTCTTGTTAAATTGAAAGAAGAAATGGCCAAGAACAGA
GTTTGCATATCTATGAGTTAGTGATCTCAGCAAAACCGTTTCACAGATGTTTAAATTCATGACAATTTATAGTCAG
ATAAATAAATCTTCAGCAAATGTCCTCGTGATATATGGTGATAATGAGTCATTACTAGATTTAATGATTTGTTTTG
AGCAACTTTTAATAAAAGGAAAAGTTTGCATCATGAACTCACAGTGGGATTTAACCACAAGAAAGAACTATTTCAT
GCTAGGCTCATTCCATGTACCTCTCATTTTTTCACAACATCATGCAGATGTTTCTGGATTCATAGATTTTATCAAG
GCAGTTAATCCTTCCAAATATCCAGAAGACTTTGTTCTTGCTAGGCTGTGGTTTGTGTCTTTTAATTGTTCAGATT
CCCCGTCTGACTGTATAACATGGGACAATTGTCTTCATGATTCCTCTTTGGATTGGTTGCCTGGGCATATTTTTGA
CAGGACTCTAGCTGGAGACAGTTACCATATATA
>dp97_PS .
TGGACTGTTGACCTCACAAGATGAGAATGGCTTGTGGGTTCTTACTGAATTGAAAGAAGAAGATGGCCAAGAACAG
AGTTTGTGTAGACTATGAGCTACTAATCTCAAGCAAAGGACTGTCACATATATTCAAATTAATGAGAGTTTATAAC
CAGATAAATAAATCTTCAGCAAATGTCCTCATCATTTATGGTGATAATGAATCACTACTAGATTTAATTATATGTA
TTGAGCAACTTTTAATCAAAGGCAAAGTCTGTATCATGAACTCACAGTGGGATTCAACCATGAGAAAGAAGTATTT
CATGCTAGGCTCATTGCACGTACCTCTCATTTTTTCACACCATCATGCAGATGTTTCTGGATTCACAGATTTTATC
AAGGCAGTTAAGCCTGCCAAATACCCAGAAGACTTTTTTCTTGCTAAGATGTGGTTTCTCTTTTTTAATTGCTCAG
ATTCAGAGTCTGAATGTGTAATGTGGGAGAGCTGTTCTCATGATGCATCTTTGGATTGGTTGCCTGGGCAGGTTTT
TGACATGACTATATCTGGAGACAGTTACAATATATA
>dp98_PS .
GGGACTGTTGACCTCACAAGATGAGAATGGTGCATGGGTTCTTGCTGAATTAAAAGAAGAGAAGGTCAAGAAAAGA
GTTTGTGTAGACTATGAGATAGTGATCTCAAGCCAATCCACTTCACATATATTCAAACTCATGGCAGTTTATAACA
AGATCAACAAATCTTCAGCAAATGTCCTCATCATATATGGTAACAATGGTTCATTAATAACTATAATAGTGTGTCT
TGAGCAAATTTTAATACAAGGCAAAGTTTGTGTCATGAATTCACAGTGGGATTTCACCATGAGAATGAGATATTTC
ACTCTAGGCTCATTGAATGTACCTCTTATTTTTACACAGCATCATGTATATGTTTCTAGATTCACAGATTTTATCA
AGGCAGTAAACCCTTCCAAATACCCAGAAGACATTTTTCTTGCTAGGCTGTGGTTTCTGTCTTTTAATTGTTCAGG
TCTGAGTCTGACTTTGTAACATGGGACAACTGTCCTCATGACGCCTCTTTGAATTGGTTGCCTAGGCATATTTTTG
ACATGACTATGTCTGGACACAGTTACAATATATA
>dp103_PS .
GGGACTGTTAATAATACAAGATGAGAATAGTTTGAGGAATCTTCATGAATCTTCATGAATTGAAAGAAGAGATGAC
TAAGCAATGAATTTGTGTAGCCTTTAACTCCCAAACAATGACATTTCATATATTTTCAAAATCATGGAGCGTTATA
TCCAGATTAATACATCGTCAGCAAACATCCTCATCATATATGGGGATAATGAATCCCTGTTGGAATTAACAGTGTG
TTTTGAGCAATTTTTAATAAAAGGCAAAGTGTGCATTGTGAACTCACATTGGGATTTAATGATGAGAAGGAAGTAT
TTCATGCTAGATTGATTTCATATACCTCATTTTTTTGCACGATCACCCAGATATTTCTGGATTCAGAGATTTTTTT
AAGACAGTTATTCCTTCCAAATACCCAGAAAATTTTTTCTTTGCTGAGATACAGTTTCTCTTTTTTGATTGTTCAG
ATTCTAATTCTGACTAATGTTTGAGCAATGTCTTCCTGATAACTCCTTGGACTGGTTACCTAGTATATTTTTGACA
TGACTATGTGTGGACATCGTTATAATATATA
>dp104_PS .
GGGACTGTTAATAATACAAGATGAGAATGGTTTGAGGAATCTTCATGAATTGAAAGAAGAGATGACTAAGCAATGA
ATTTGTGTAGCCTTTGAGATAGAAAGAACTCCCAAACAATGACATTTCATATATTTTCAAAATCATGGAGCGTTAT
ATCCAGATTAATACATCGTCAGCAAACATCCTCATCATATATGGGGATAATGAATCCCTGTTGGAGTTATCAGTGT
GTTTTGAGCAATTTTTAATAAAAGGCAAAGTGTGCATTGTGAACTCACATTGGGATTTATCGATGAGAAGGAAGTA
TTTCATGTTAGATTGATTTCATATACCTCATTTTTTTTGCACGATCACCCAGATATTTCTGGATTCAGAGATTTTT
TTAAGACAGTTATTCCTTCCAAATACCCAGAAAATTCTTTCTTTGCTGAGATACAGTTTCTCCTTTTTGATTGTTC
AGATTCTAAGTCTGACTAATGTTTGAGCAATGTCTTCCAGATAACTCCTTGGACTGGTTACCTAGGTATATTTTTG
ACATGACTATGTGTGGACATCGTTATAATATATA
>dp105_PS .
GGGACTATTGTAGAAGATGAGAATGGTTTGTGGATTCTTCCTGAATTGAAAGAAGAGGTGACTAAGAAATGAATTT
GTATAACCTTTGAGATAGAATTCCCAAACTCTGACATTTTATATATTTTCAAAATCATAGAATGTTGTAACTACAT
TAACACATCTTCAGCAAATATCCTCATCATATATGGGAATAATGAATCCCTGCAATATTTAATGGTGTGTTTTGAG
CAATTTTTAATAAAATGCAAAGTTTACATTGTGAACTCACATTGGGATTTAACGATGAGAAGGAAATATTTCATGC
TAGGATCATTGCATGTACCTCATTTTGTTTTTTGCACCATCGCCCAGATATTTCTGGATTCAGAGATTTTCTTAAG
GCAGTTATTCCTTCCAAATACCCAGGAATTTTTTTTCCTTTCTGGGCGATGGTTTCTCTTCTGATTATTCAGATTC
TAAGTCTGTTTAATGTTTGAGAAATGTCCTCCTGATAACTCCTTTGACATAACTATGTGTGGACACAGTTACAATA
TATA
>cv67 .
GGCCGTCTTTATATCTGATGATGTGAAAGGGGAGCAGTTCATGAGGGACCTGGAAGCAGAGATGCTAAAGAAAGGT
ATCTGTGTGGCCTTGACAGAAAAGCTCCCTGCCATTACCACAATGTTTGGATCAAGTCACACCACTTTTATGACTA
GGATCAGAGTTTCATCTGCCAATGTGCACATCCTGTATGGGGAGGTCAGGAGCCTCATCACTGTGGACATCGCAGC
TGAATTCTTTTTGACCACAGGAAAGGTGTGGATCATGACTGCAAAGTGGGACATGGTTGTGTATGAGACGGACCAC

```
ATGCTGCACTCTTTCCATGGAAGCTTCTCATTGTCACCTCATAAGAGGGAAATTCCTGGCTTCAAGCACTTTCTGA
AGACATCCAACCCTTCTCAATACCCACAAGATTTTTACTACAGTATATTGTGGACTTTCTTTTATGACTGCTCACT
TCCTGGGTCCTTGTGTGGGAAGATAGGAGTCTGCCTACCAAATATTTCCTTAGAGACTCTGCCTGGAAACTTGGAC
ATAATGACCATATCGGACTCAAGCTATTTCATCTAT
>cv68 .
GGCCGTCTTTATATCTGATGATGTGAAAGGGGAGCAGTTCATGAGGGACCTGGAAGCAGAGATGCTAAAGAAAGGT
ATCTGTGTGGCCTTGACAGAAAAGCTTCCTGCCATTACCACAATGTTTGGATCAAGTCACACCACTTTTATGACTA
GGATCAGAGTTTCATCTGCCAATGTGCACATCCTGTATGGGGAAGTCAGGAGCCTCATCACTGTGGACATCGCAGC
TGAATTCTTTTTGACCACAGGAAAGGTGTGGATCATGACTGCAAAGTGGGACATGGTTGTGTATGAGACGGACCAC
ATGCTGCACTCTTTCCATGGAAGCTTCTCATTGTCACCTCATAAGAGGGAAATTCCTGGCTTCAAGCACTTTCTGA
AGACATCCAACCCTTCTCAATACCCACAAGATTTTTACTTCAGTATATTGTGGATTGCCTTTTTAAATTGTTCGCC
TCCTGGGTCCTTGTGTGGGAAGATAGGAGTCTGCCTACCAAATATTTCCTTAGAGACTCTGCCTGGAAACTTTGAC
TTAATGACCGTATCGGACTCAAGCTATTTCATCTAT
>cv57 .
GGCTGTTTTTGTATCTGATGATTTGAAAGGGGAGCAGTTCCTGAGGGACCTGGAAGCAGAGATGCTAAAGAAAGGT
ATCTGTGTGGCCTTGACAGAAAAGCTCCCTGCCACTCAGATAATGTATGCCTCGAGTGACATCATTTTCATGAGCA
GGATCAGAGTTTCATCTGCCAATGTGCACATCCTGTATGGGGAAATCAGGAGCCTCATCACTGTGGACATTTCAGC
AGTATACTTTTTAACAACAGGAAAGGTGTGGATCATGACTGCAAAGTGGGACATCGTTGTGTATGAGACGGACCAC
ATGCTGCACTCCTTTCATGGAAGCTTCTCATTTTCTCCTCACAAGGGGGAAGTCCCTGGTTTCAGACACTTCCTCC
AAACAACCAACCCTTCCCAATTCCCTGAAGACTTTTATTTCAGTAAACTATGGCTTAGCATGTTTCATTGCTCACT
TCCTGGTTCACAATGTGGAGCAATTGATGTTTGCCCACCAAATACCTCCTTAGAGTTGTTGCCTGGAAACATTGAT
GTCATGACCATGTCAGACTCCAGCTATTTTATCTAC
>cv65 .
GGCCATCTTTGTATCTGATGATGTGAAAGGTGAGAAGTTCCTGGGGGACCTGAAAGCAGAGATGCTAAAGAAAGGT
ATCTGTGTGGCCTTGACAGAAAAGCTCCCTGCCACTAAGATAATGTATGCTTCAAGTGACATCACTTTCATGAGCA
AGATTAGAGTTTCATCTGCCAATGTGCATATCCTGTATGGGGAAGTCAGTAGCCTCATCACTGTGGACATTGCAGC
AGAATTCTTTTTGACTACAGGAAAAGTATGGATCATGACTGCAAAGTGGGACATTGTTGTGTATGAGGCGAACCAC
ATGCTACATTCCTTTCATGGGAGCTTCTCATTTTCTCCTCATAAGGGGGAAGTTCCTGGTTTCAGACACTTCCTCC
AAACAACCACCCCTTCCCAATACCCTGAAGACTTTTATTTCAGCAAATTATGGCTCAACTTTTTTGATTGCTCACT
TCCTGGTTCACAATGTGGGCGAATTGGCGTTTGCCCACCGAATACATCCTTAGAGTTTATGCCTGGAAACACTAAC
CTCATGACATTGTCTGACTCCAGCTATTTCATCTAC
>cv61 .
GACCATCTTTGTATCTGATGATGTGAAAGGTGAGCACTTCCTGGGGGACCTGAAAGCAGAGATGCTAAAGAAAGGT
ATCTGTGTGGCCTTGACAGAAAAGCTCCCTGCCACTAAGATAATGTATGCATCGAGTGACATCACTTTCATGAGCA
AGATCAGAGCTTCATCTGCCAATGTGCATATCCTGTATGGGGAAGTCGGGAGTCTCATCACTGTGGACATTGCAGC
AGAATTCTTTTTGACAACAGGAAAGGTGTGGATCATGACTGCAAAGTGGGACATTGTTGTGTATGAGACAAACCAC
ATGCTGCATTCCTTTCATGGAAGCTTCTCATTTTCTCCTCACAAGGGGGAAATTCCTGGTTTCAGACACTTCCTCC
AAACAATCAAGCCTTCCCAATACCCTGAAGACTTTTATTTCAGCAAATTGTGGCTCACTTTTTTTGATTGCTCACT
TCCTGGTTCACAATGTGGGAGAATTGGAGTTTGCCCACCAAATACATCCTTAGAGTTTATGCCTGGAAACATTGAC
CTCATGACATTGTCTGACTCCAGCTATTTCATCTAC
>cv60 .
GGCTATCTTTGTATCTAATGATGTGAAAGGTGAGCACTTCCTGGGGGACCTGAAAGCAGAGATGCTAAAGAAAGGT
ATCTGTGTGGCCTTGACAAAAAAGCTCCCTGCCACTAAGATAATGTATGCATCAAGTGACATCACTCTCATGAGCA
AGATCAGAGTTTCATCTGCCAATGTGCATATCCTGTATGGGGAAGTCGCGAGCCTCATCAATGTGGATATTGCAGC
AGAATTCTTTTTGACAACAGGAAAGGTGTGGATCATGACTGCAAAATGGGACATTGTTGTGTATGAGACGGACCAC
ATGCTGCATTCCTTTCATGGAAGCTTCTCATTTTCTCCTCACAAGGGGGAAGTTCCTGGTTTCAGACACTTCCTCC
AAACAACCAAGCCTTCCCAATACCCTGAAGACTTTTATTTCAGCAAATTATGGCTCAATTTTTTTGATTGCTCACT
TCCTGGTTCGCAATGTGGGAGAATTGGAGTTTGCCCCCCGAATACATCCTTAGAATTTATGCCTGGAAACATTGAC
CTCATGACATTGTCTGATTCCAGCTATTTCATCTAC
>cv63 .
GGCCATCTTTGTATCTAATGATGTGAAAGGTGAGCACTTCCTGGGGGACCTTAAAGCAGAGATACTAAAGAAAGGT
ATCTGTGTGGCCTTGACAGAAAAGCTCCCTGTCACTAAGATAATGTATGCATCAAGTGACATCACTTTCATGAGCA
AGATCAGAGTTTCATCTGCCAATGTGCATATCCTGTATGGGGAAGTCGCGAGCCTCATCAATGTGGATATTGCAGC
AGAATTCTTTTTGACAACAGGAAAGGTGTGGATCATGACTGCAAAGTGGGACATTGTTGTGTATGAGACAGACCAC
ATGCTACATTCCTTTCATGGAAGTTTCTCATTTTCTCCTCACAAGGGGGAAGTTCCTGGTTTCCGACAATTCCTCC
AAACAATCAAGCCTTCCCAATACCCTGAAGACTTTTTTTTCAGCAAATTATGGCTCAAATATTTTGATTGCTCACT
TCCTGGTTCACAATGTGGGAGAATTGGAGTTTGCCCACCGAATACATCCTTAGAGTTTATGCCTGGAAACATTGAC
CTCATGACATTGTCTGATTCCAGCTATTTCATCTAC
>cv56 .
GGCCATCTTTGTATCTGATGATGTGAAAGGTGAGCACTTCCTGGGGGACCTGAAAGCAGAGATGCTAAAGAAAGGT
ATCTGTGTGGCCTTGACAAAAAAGCTCCCTGCCACTAAGATAATGTATGCATCAAGTGACATCACTTTCATGAGCA
AGATCAGAGTTTCATCTGCCAATGTGCATATCCTGTATGGGGAAGTTGGAAGCCTGATCACTGTGGACATTGCAGC
```

```
AGAATTCTTTTTGACAACAGGAAAGGTGTGGATCATGACTGCAAAGTGGGACATTGTTGTGTATGAGACGGACCAC
ATGCTGAATTCCTTTCATGGAAGCTTCTCATTTTCTCCTCACAAGGGGGAAGTTCCTGGTTTCAGACACTTCCTCC
AAACAACCACCCCTTCCCAATACCCTGAAGACTTTTATTTCAGCAAATTATGGCTCAATTTTTTTGATTGTTCACT
TCCTGGTTCACAGTGTGGGAGAATTGGAGTTTGCCCACCGAATACATCCTTAGAGTTTATGCCTGGAAACATTGAC
CTCATGACATTGTCTGACTCCAGCTATTTCATCTAC
>cv1 .
GGGGCTGGTCATCACAGAAGGTCAGAAAGGTCATCAGTTTCTATCAGATGTGAAAACTGAGATGAACAGAAACAAA
GTCCGTGTGGCATTTGTGAAAATGATCTCGAATGCAGTCTTGTCGGTTCTTGCTCATGCACAGCAACATGATTTCC
TGACCAGGGAGACATCACCAGTAAACGTGGGTATCATTTACTATGACACCAAGAGCTTGAATGATGTGAACTATAA
TATTGGACAGTATCGAATGACATGGACTGTCTGGATCACAAACTCACAGTGGCATGCAGATATGCCTGGAAGAAAC
TTTATACTTGAGTCATTCCATGGGACTCTCATTTTTGCAAAGCATCACAAGGAGATTCGTGGTTTCCAAAAGTTTA
TTCAGGCTGTGAACCCTTCCATGTATCCAGAAGACAATTTCCTCACTCTATTTTGGTATGATCATTTTCATTGCTC
AGTGGCTGATTCTGACTGTAAGTTGAAGAACTGTACACCTAATGCATCTCTGGCATGGTTGCCTGGGAATCGTTTT
GATATGACCATGAATGATGACAGTTACAATATATAC
>cv2 .
GGGACTGATCATCACAGAAGGTCATAGAGGTGTTCAGTTTCTCTCAGATGTGAGAGCCGAAATGGAAAGGAATAGA
ATCTGTGTAGCATTTTTGAAAATGGTCTCAAGTGCTTTTGAATCATATTTCTTTAGGCCACTGCAACATGATATCA
TAGCCAGGGAAACATCCCCAGTGACTGTGGTTATCATATACTGTGATACTGAGACTCTCAATGATATAAACTACCT
TATAGGGCAAAATGTAGGCACATGGAGAGTCTGGGTCACAAACTCTCAATGGCATGCTGACATGTCTGGGGCTAAT
TTCATTCTTAACCCATTCCATGGGAGCTTCATTTTTTCTAACCATCATGAGGAGATTTCTGCTTTCAAAAATTTTG
TCCAAGAAGCCAATCCTTTCAGATACCCAGAAGACACTTACCTTACTATGTACTGGCTCAATCATTTCCGTTGCTC
AATCTCTGACTCTGACTGTGCACTGAGGAACTGTACACCTAATGCCTCTTTGGCACAGCTGCCCATGAATCGTTTT
GACACAGATATGACCGATGAGAGCTACAACATATAC
>cv5 .
GGGACTGCTCATCACAGAAGGTCATAAGGGTCTTCAGTTTCTCTCAGATATAAAAAAAGAGATGGACAGGAACAAA
ATTTGTGTAGCATTTGTGAAAAGGGTCTCAACCATTGCTGCATTTCATCTGTCAGCTTTCCAGACACAAGATATTC
TTACCACAGAAACATCCTCAATAAATGTGATTATTATTTACTATGATACTGATAGTCTAAATGATGTCAACTATAA
CATAGGGCAACACTTAGTGACATGGAAAGTCTGGGTCACAAATTCACAATGGCATCCTGACATGGCTGGGAGAAAT
TTCATACTTGACCCATTCCATGGAATTCTTGTCTTTGCACACCACAATGAGGAAATTTCAGGCTTCCGAAACTTTG
TCGAGGAAGCCACCCCTTTCAAATACCCAGAAGACACTTATCTCACTATGTATTGGTTCAAGAATTTCCATTGCTC
ATTCTCTGAATCTGACTGTGCATTGAAGAACTGTGTCCCTAATGCTTCTCTGGCTTGGTTGCCTCCAAATCGTTTT
GACGTGACCATGAGTGATGAGAGTTACAATATATAC
>cv7 .
GGGAGTGGCCATTACAGAAGGTCCAAAAGGACTTCGTTTCCTGGCAGATGTGACAAAAGAAATGGACAGGAACAGA
GTCTGCATGGCATTTGTGAAAATGATCCCAGTTGATCCTGTATCATACATGACACATACCGAACAACATGAATTGC
TGGTGGGAGAATCCTCTGCTGTAAATGTGGTTATCATTTACTATGATGCTGACAGTTTAAATGATATAAACTATAG
CATAAGTCTCCATTTAGTGACATGGAGGGTCTGGGTAACAAACTCGCAATGGCATGCAGATTTGACTGGGAAAAAG
TTCTTGCTTGACTCATTCCATGGGACAATCATTTTTTCCAATCACCATGAGGAAATTTCTGGTTTCAAACACTTTG
TTCAGACAGTGAATCTCTCCATGTACCCAGAAGATATTTTCCTTTATCAATTTTGGTACTGGAATTTCAATTGCTT
GATATCAGAGTCTGATTGTTCATTGAAGAACTGTACACCAAATGCCTCTTTGGCACAGTTGCCTGCAAATCGTTTT
GACCCTGCTATGAGTGATGTGAGTTACAACATATAC
>cv8 .
GGGACTGGTCATCACAGAGGGTCACAAAGGACTTCAGTTTATGTCTGATTTGACAGGAGAGATGGACAGGAACAAA
GTCTGTGTAGCATTTGTGAAAATAGCATCAACTCCTATTGTATCGTATATGGCACTATCAAAGGAATATGTTATTC
TGACCAAAGAAACATCACAAGTGAGTGTGGTGATCCTTTACTATGACACCGATATTTTAAGTGATGTAGACTATAA
CATAGAGCAACACTTACTTACTGGGAAAATCTGGGTGACAAACTCACAATGGCATGCTGACATGACAGGGAAAAAT
TTCATCCTTAATTCATTCCATGGGACTTTCATTTTTTCCAACCACCATGAAGAAATTTCTGGTTTCAAAACCTTTG
TCCAAGAAGTCAACCCTTCCAAATACCCAGAAGACTTTTATCTTACTATGTTCTGGTTCAATAATTTCCACTGTTC
ATTCTCTGATGCTGACTGTTCACTGAAAGACTGCACACCCAATGCCTCTCTGGCATGGCTGCCTGTGAATCACTTT
GACACAACTATGACTGATTGGAGTCACAATGTGTAC
>cv9 .
GGGAGTGGCCATTACAGAAGGTCCAAAAGGACTTCATTTCCTGGCAGATGTGACAAAAGAAATGGACAGGAACAGA
GTCTGCATGGCATTTGTGAAAATGATCCCAGTTGATCCTCTATCATACATGGCACATACACAACAACATGAATTCC
TGGTGGGAGAATCCTCTGCTGTAAATGTGGTTATCATTTACTATGATGCTGACAGTTTAAATGATATAAACTATAG
CATAGGTCTCCATTTAGTGACATGGAGGGTCTGGGTAACAAACTCGCAATGGCATGCAGATTTGACTGGGAAAAAG
TTCTTGCTTGACTCATTCCATGGGACAATCATTTTTTCCAATCACCATGAGGAAATTTCTGGTTTCAAACACTTTG
TTCAGACAGTGAATCTCTCCATGTACCCAGAAGATATTTTCCTTTATCAATTTTGGTACTGGAATTTCAATTGCTT
GATATCAGAGTCTGATTGTTCATTGAAGAACTGTACACCAAATGCCTCTTTGGCACAGTTGCCTGCAAATCGTTTT
GACCCTGCTATGAGTGATGTGAGTTACAACATATAC
>cv10 .
GGGACTGGTCATCACAGAAGGTCAGAAAGGTCTTCAGTTTCTCTCAGATGTGAGAGCAGAGATGGGCAGGAACAGA
ATCTGTGTAGCATTTGTAAAAATGATTGAAGTTACTCTTCGTGCTTTTTTTGGAAACTTTCAATACAATTTCCAGA
```

CCAGAGAAATATCACCAGCAAACGTAGCTATTATTTACTATGATAGTGAGAGTCTAAGTGATGTAAACTTTCATAT
AGGGGGCTATTTAGTGACATGGAGAGTCTGGGTGACAAACTCACAATGGCATGCTGACATGATGGGGAGAAATTTT
ATACTTGACTCATTCCACGGGACCCTCATTTTTTCAAATCACCATGAGGAGATTCCTGGTTTCAAAAATTTTGTTC
AAAGGGCTAATCCTTCCAGATACCCAGAAAATAATTTCCTTTCTCTGTATTGGTTCAAGAATTTCCATTGCTCATT
ATCTGATTCTGGCTGTTCATTGAGGAGCTGTCCACCCAATGCTTCTCTGGCTTGGCTGCCTGTGAATCACTTTGAC
ACAGCAATGAGTGACGTGAGTTACAATATATAC
>cv11 .
GGGACTGCTCATCACAGAAGGTCAGAAAGGTCTTGAGTTTCTCTCAGATGTGAGAGCAGAGATGGACAGGAACAGA
ATCTGTGTAGCATTTGTAAAAATGATTGAAGTTACTCTTCATGCTTTTTTTGGAAACTTTCCATATAATTTCCAGA
CCAGGGAAACATCACCAGCAAATGTAGTTATCATTTGCTATGATAGTGAGAGTCTATGTGATGTAAACTTTATTAT
AGGGGGCTATTTAGTGACATGGAGAGTCTGGGTGACAAACTCACAATGGAATGCTGACATGGTGGTGAGAAATTTT
ATACTTGACTCATTCCATGGGACTCTCACTTTTTCAAATCACCATGAGGAGATTCCTGGTTTAAAAATTTTGTTCA
AAGGGATAATCCCTTCCAGATACCCAGAAAATAATTTTTTTTCTCTGTATTGGTTCAAGAATTTCCATTGCTTGTT
ATCTAATTCTGACTGTTCATTGAGGAGCTGTCCACCCAATGCTTCTCTGGCTTGGCTGCCTGTGAATCACTTTGAC
ACAGCAATGAGTGATGTGAGTTACAATAAATAC
>cv12 .
GGGCCTGCTTATCACAGAAGGCCATAAGGGTCTTCAGTTTCTCTCAGATGTGCGAGCTGAGATGGACAGGAACAGA
GTCTGTGTAGCCTTTGTGAAAATGGTCTCCACTGTGACTGTGTCATATCTCTCAGCCACACAGAAACATCATATCC
TGACCGCAGATACATCATCAGTCAATGTTGTGGTTATGTACTATGATACAGATAGTCAAACTGATGCGAACTATAA
CATATGGCAACACTCTGTGACATGGAAAGTCTGGGTGACAAACTCACAGTGGCATCCTGATCTGGTAGGAAGAAAT
TTCATACTTGACCCATTCCATGGGACTCTAATTTTTTCACACAACCACAAAGAGATTTCAGGTTTCAAAAACTTTG
TCCAGGGAGCTACTCCTTTCAAATACCCAGAAGACATTTATCTAATTATGTATTGGTACAATAATTTCCATTGCTC
CTTCTCTGAGCCTGATTGTATACTGAAGAACTGTACACCTAATACATCTTTGGCTTGGTTGCCTCTGAATCGTTTT
GACACAGCCATGAGTGATGGGAGCTATCATATATAC
>cv13 .
AGGAGTGATTGTCACAGCAGGTGCAAAGGGTCTTCAGTTTCTCTCAGATGTGAGAGCAGAGATGGATGGGAACAGA
GTCTGCTTAGCATTTGTAAAAACAGTCACAAGTGATCCTCTGTTGTATGTTGCAAATACAGAACTACATGACTTCC
TGACCAAGGACACCATTGGAGTAAATGTGATTATCATTTACCACGATACTGAGAATGTCAATGACTTAAACCATAA
TGTAGGGTTACATTTAGGGACATGGAGAGTCTGGGTAACAAACTCACAATGGCAGGCTGACCTGACTGGGAAAAAT
TTCCTTCGTGACTCATTCCATGGAACTTTCATTTTTTCAAATCACCACAAGGAGATTTCTGGTTTTAAAAAGTTTA
TCCAGACAGTGAACCCTTCCATTTATCCAGAAGACATATTTATACAGCAGTTTTGGTACTCCCATTTCCAGTGCTC
ATTATCTGACTCCGGCTGTGCACTGGAGAACTGTATACCCAATGCCTCTCTGGAGTGGCTGCCTGTGAATCGTTTT
GACCCCACCATGAGTGATGCGAGTTACAACATATAC
>cv15 .
GGGACTGGTCATTATGGAAGGTCAGAAAGGATTTCAGTTTCTCTCAGATGTGAGAGTGGAGATGGACAGGAACAAA
ATCTGTGTAGCATTTGTGAAAATGGTCTCAACTCATCTTGTATCATATATGGCATCTGCAAAGGAATACATTATTT
TGACTAAGGAAACTTCACAAGTGAATGTGGTGATCATTTACTATGACACTGATTTCCTAAATGATGTGAACCATAA
CATAGAGCAGAACTTATTAACAGGAAAAGTCTGGGTGACCAACTCACAGTGGCATGCTGACATGACTGGGAAAAAC
TTCATCCTCAATTCATTCCATGGGACACTCATTTTTTCTAATCATCATGAAGAAATTTCTGATTTCAAAACTTTTG
TCCAAGAAGCCAACCCTTCCAAATACCCAGAAGACTTTTACCTCACTAGATTTTGGTTCAGCAATTTCCATTGCTC
ATTCTCTGATGCTGACTGTTCATTGAAAGACTGCACACCCAATGCCTCGCTGGTGCATCTGCCTGCAAATCATTTT
GACCCAACCATGACTGATTGGAGTCACAATGTACAC
>cv16 .
GGGGCTGCTCACCACGGAAGGCCATAAGGGTCTTCAGTTTCTCTCAAATGTGAGAACTGAGATGGACAGGAATAGA
ATCTGTGTAGCTTTTGTGAAAATTGTGTCAAGTGCCACTGTGTCATATCTCTCAGTGATGCAGAAACATGATATCT
GGACTGCAGATACATCATCAGTCAATGTGGTGGTTATTTACTATGATACAGATAGTCAAAATGATGTGAGTTACAA
CATAGTACAAAACTTAATGACATGGAAGGTCTGGATGACAAACTCACAATGGCAAGCTGTCCTAGATGGAAGAAAT
TTCATACTTGACTCATTCCATGGGGTTCTTGTTTTTTCACACCACCATGAAGAGATTTCAGGTTTTAAAACTTATG
TCCAGGAAGCTACCCCTTTCAAATACCCAGGTGACACTTATCTCTTTGTGTATTGGTACAAGAATTTCCACTGCTC
ATTCTCTGAGTCTGACTGTACACTGAAGAACTGCACACCTAATGTCTCTCTGGCTTGGCTGCCTCTGAATCATTTT
GACACAGCCATGAGTGATAGGAGTTACAACATATAC
>cv17 .
GGGACTGCTCATCACAGAAGGTCAGAAAGGTCTTGAGTTTCTCTCAGGTGTGAGAGCAGAGATGGACAGGAACAGA
ATCTGTGTAGCATTTGTAAAAATGATTGAAGTTACTCTTCGTGCTTTTTTGGGAAACTTTCAATATAATTTCCAGA
CCAGAGAAACATCACCAGCAAATGTAGCTATTGTTTACTATGATAGTGAGAGTCTAAGTGATGTAAACTTTATTAT
AGGGGGCTATTTAGTGACATGGAGAGTCTGGGTGACAAACTCACAATGGCATGCTGACATGGTGGTGAGAAATTTT
ATACTTGACTCATTCCATGGGACCCTCACTTTTTTAAATCACCATGAGGAGATTCCTGGTTTCAATAATTTTGTTC
AAAGGGATAATCCTTCCATATACCCAGAAAACAATTTCTTTTCTCTGTATTGGTTCAAGAATTTCCATTGCTTGTT
ATCTAATTCTGACTGTTCATTGAGGAGCTGTCCACCCAATGCTTCTCTGGCTTGGCTGCCTGTGAATCACTTTGAC
ACAGCAATGAGTGATGTGAGTTACAATAAATAC
>cv18 .

AGGACTGGTCATTATGGAAGGTCATAAAGGACTTCAGTTCCTCTCAGATGTGACAGGGGAAATGGACAGAAACAAA
ATCTGTGTAGCATTTGTGAAAATGGTCTCAACTCATCTTGTATCATATATGGCAGCTGCAAAGGAATACATTATTT
TGACCAAGGAAACATCACAAGTGAATGTGGTGATCATTTACTATGACACTGATTTCCTAAATGATGTGAACTATTA
CATAGAGCAGAACTTATTAACAGGAAAAGTCTGGGTGACAAACTCACAGTGGCATGCTGACATGACTGGGAAAAGT
TTCATCCTCAATTCATTCCATGGGACTCTCATTTTTTCTAACCATCATGAAGAAATTTCTGATTTCAAAATGTTTG
TGCAAGAAGTCAACCCTTCTAAATACCCAGATGACTTTTACCTCACTAGATTTTGGTTCAACAATTTCCACTGCTC
ATTCTCTGATGCCGACTGTTCCCTGAAGGACTGCTCACCCAATGCCTCACTGGTGCAGTTGCCTGCAAGTCATTTT
GACACAACTATGACCGATTGGAGCCACAATGTACAC
>cv19 .
AGAACTGGTCATTATGGAAGGTCATAAAGGACTTCAGTTCCTCTCAGATGTGACAGGGGAGATGGACAGAAACAAA
ATCTGTGTAGCATTTGTGAAAATGGTCTCAACTCATCTTGTATCATATATGGCAGCTGCAAAGGAACACATTATTT
TGACCAAGGAAACATCACAAGTGAATGTGGTGATCATTTACTATGACACTGATTTCCTAAATGATGTGAACCATTA
CATAGAGCAGAACTTATTAACAGAAAAAGTCTGGGTGACAAACTCACAGTGGCATGCTGGCATGACTGGGAAAAGT
TTCATCCTCAATTCATTCCATGGGACTCTCATTTTTTCTAACCATCGTGAAGAAATTTCTGATTTCAAAATGTTTG
TGCAAGAAGTCAACCCTTCTAAATACCCAGATGACTTTTACCTCACTAGATTTTGGTTCAACAATTTCCACTGCTC
ATTCTCTGATGCCGACTGTTCCCTGAAGGACTGCTCACCCAATGCCTCACTGGTGCAGTTGCCTGCAAGTCATTTT
GACACAACTATGACCGATTGGAGCCACAATGTACAC
>cv22 .
GGGAATAATCGTTGCCGAAGGTCATAAAGGCTTGCAATTTGTCTCAGATATCAGAGGAGAGGCAGAGAAAAGCAGA
GTCTGCATAGCATTTGTGAAAATGATCTCAAGTTCTTATGCATCCTATTTCTATCAAATAGAGAAAAGTATCATAG
CCCAGGAAGAGTCACCAGTAAATGTGGTTATCATTTATTATGATACAGACAGTCTAAATCATGTAAACCACTATAT
ACAGTTGCATTTAGTGACTTGGAAAGTCTGGGTGACAAATTCACAATGGCATGCTGATGTGGCTGGGAGAAACTTC
ATTCTTGATACATTCCATGGGCTTCTGATTTTTTCACATCACCATGAGAAGATTTCTGGTTTTAAGGACTTTATCC
AGAAAGCCACCCCATCCAAATATCCAGAAGACACTTTTCTCAGTCTGTTTTGGTTCCAGAATTTCCATTGCTCACT
GTCTGAATTCGACTGTTCCCTGAAGAACTGTACACCGAATGCCTCTCTGGCTTGGCTTCCTGAGAATATTTTTGAC
CCAGTCATGACTGATTGGAGCTACGACATATAC
>cv23 .
GGGCCTGCTCACCACAGAAGGCCATAAGGGTCTTCAGTTTCTCTCAGATGTGAGAACTGAGATGGACAGGAACAGA
GTCTGTGTAGCCTTTGTGAAAATTGTGTCAAGTATCACTGTGTCATATTTCTCAGCCATGCAGAAATATGATATCC
TGACTGCAGAAACTTCATCAGTCAATGTGGTGGTTATTTACTATGACACTGATAGTCTAAATGATGTGAGCTACAA
CATAGTACAAAACTTAGTGACATGGAAAGTCTGGATGACAAACTCACAATGGCAAGCTGTCCTAGATGGAAAAAAT
TTCATACTTGACTCATTCCATGGGGTTCTTGTTTTTTCACACCACTATGAAGAGATTTCAGGTTTCAAAAATTATG
TCCAGGAAGCCACTCCTTTCAAATACCCAGATGACACTTACCTCTTTATGTACTGGTACAAGAATTTCCACTGCTC
CTTCTCTGAGTCTGACTGTGCATTGGAGAACTGTACACCTAATGCCTCTCTAGCTTGGTTGCCTCCAAATCATTTT
GACACAGCCATGAATGACAGGAGCTACAATATATAC
>cv24 .
GGGAATAATCGTTGCTGAAGGTCATAAAGGCTTGCAGTTTGTCTCAGATTTCAGAGGTGAGGCAGAGAAAAGCAGA
GTCTGCATAGCATTTGTGAAAATGATCTCAAGTTCTTATGCATCCTATTTCTTACATATAGAGAAAAATGATATCA
TAGCCCAGGAAGCGTCACCAGTAAATGTGGTTATCATTTATTATGATACAGACAGTCTAAATCATGTAAACCACTA
TATACAGTTGCATTTAGTGACTTGGAAAGTCTGGGTGACAAATTCACAATGGCATGCTGATGTGGCTGGGAGAAAC
TTCATTCTTGATACATTCCATGGGCTTCTGATTTTTTCACATCACCATGAGAAGATTTCTGGTTTTAAGGACTTTA
TCCAGAAAGCCACCCCATCCAAATATCCAGAAGACACTTTTCTCAGTCTGTTTTGGTTCCAGAATTTCCATTGCTC
ACTGTCTGAATCCGACTGTTCCCCGAAGAACTGTACACCGAATGCCTCTCTGGCTTGGCTTCCTGAGAATATTTTT
GACCCAGTCATGAGTGACTGGAGCTACGACATATAC
>cv26 .
GGGCCTGCTCACCACAGAGGGCCATAAGGGTCTTCAGTTTCTCTCAGATGTGAGAACTGAGATGGACAGGAACAGA
GTCTGTGTAGCCTTTGTGAAAATTGTGTCAAGTATCACTGTGTCATATTTCTCAGTCATGCAGAAACATGATATCC
TGACTGCAGATACATCATCAGTGAATGTGGTGGTTATTTACTATGACACTGGTAGTCTAAATGATGTGAGCTACAA
CATAGTGCAAAACTTAATGACATGGAAAATCTGGGTGACAAACTCACAATGGCAAGCAGACCTGGATGGAAGAAAT
TTCATACTTGACTCTTTCCATGGGGTTCTTGTTTTTTCACACCACTTTCAAGAGATTTCTGGTTTTACAAATTATG
TCCAGGAAGCCACTCCTTTCAAATACCCAGAAGACACTTATCTCTTTATGTACTGGTACAAGAATTTCCACTGCTC
CTTCTCTGAGTCTGACTGTGCATTGGAGAACTGTACACCTAATGCCTCTCTAGCTTGGTTGCCTTCAAACAATTTT
GACACAGCCATGAATGACAGAAGCTACAATATATAC
>cv27 .
GGGCCTGATCATCACAGCAGACCATAAGGGTCTTCAGTTTCTCTCGGATGTGCGAGCTGAGATAGACAGGCATGGA
ATCTGTGTAGCTTTTGTGAAAATGGTCTCAACTGTGACTGTGTCATATCTCTCAGTCATACAGAAACATGATATTT
TTACTGCAGATACATCATCAGTCAATGTAGTGGTTATTTACTATGATAATGATAATCGAAATGATTTACACTACAA
CATAGTACAAAGCTCAGTGACTTGGAAAGTCTGGGTGACAAACTCACAATGGCATGCCGACCTGAGTGGGAGGAAT
TTTGTACTTGACACATTCCATGGGGTTCTTGTTTTTTCACACCACCATGAAGAGATTTCAGGTTTCAAAAATTTTG
TCAAGGAAGCCACTCCTTTCAAATATCCAGAAGACTCTTATCTCTTTATGTATTGGTACAAGAATTTCCACTGCTC
CTTCTCTGAACCTGACTGTGCATTAGAGAACTGTACAATTAATGCCTCTGTGGCTTGGTTGCCTCCAAATAGTTTT
GACACAGCCATGAGTGATGGGAGCTACAATATATAC

>cv28 .
GGGAATGTTTGTTGCTGAAGGTCATAAAGGCTTGCAATTTGTCTCTGATATCAGAGGACAGGCAGACAAAAGCAGA
GTCTGCATAGCATTTGTGAAAATGATCTCAAGTTCTTATGCATCCTATTTCTTCCATACAGAGAAAAATGATATCA
TAGCCAGGGAAGCGTCACCAGTAAATGTGGTTATCATTTATTATGATACAGACAGTCTAAATCATGTAAACCACTA
TATACGGTTGCATTTAGTGACTTGGAAAGTCTGGGTGACAAATTCACAATGGCATGCTGATGTGGCCGGGATAAAT
TTCATCCTTGATACTTTCCATGGGCTTCTGATTTTTTCACATCACCATGGGAAGATTTCTGGTTTTAACGAATTTA
TCCAGAAAGCCACCCCATCCAGATATCCAGAAGACACTATTCTCAGTCTGTTTTGGTTCCAGAATTTCCACTGCTC
AGTGTCTGAATTCGACTGTTCCCTGAAGAACTGTACACCGAACGCCTCTCTGGCATGGCTTCCTGAGAATATTTTT
GACCCAGTCATGAGTGACTGGAGCTACAACATATAC
>cv29 .
GGGCCTGCTCACCACAGAAGGCCATAAGGGTTTTCAGTTTCTCTCAGATGTGAGAACTGAGATGGACAGGAACAGA
GTCTGTGTAGCCTTTGTGAAAATTGTGGCAAGTATCACTGTGTCATATTTCTCAGTCATGCAGAAACATGATATCC
TGACTGCAGATACATCATCAGTGAATGTGGTGGTTATTTACTATGACACTGATAGTCTAAATGATGTGAGCTACAA
CATAGTGCAAAACTTAATGACATGGAAAATCTGGGTGACAAACTCACAATGGCAAGCAGACCTGGATGGAAGAAAT
TTCATACTTGACTCTTTCCATGGGGTCCTTGTTTTTTCACACCACTATGAAGAGATTTCAGGTTTCAAAAATTATG
TCCAGGAAGCCACTCCTTTCAAATACCCAGATGACACTTATCTCTTTATGTACTGGTACAAGAATTTCCACTGCTC
CTTCTCTGAGTCTGATTGTGCATTGGAGAACTGTATACCTAATGCCTCTCTAGCTTGGTTGCCTTCAAATCGTTTT
GACACAACCATGAGTGACAGGAGCTACAATATATAC
>cv34 .
GGGCGTGCTCACCACAGAAGGCCATAAGGGTCTTCAGTTTCTCTCAGATGTGAGAACTGAGATGGACAGGAACAGA
GTCTGTGTAGCCTTTGTGAAAATTGTGTCAAGTATCACTGTGTCATATTTCTCAGCCATGCAGAAACATGATATTC
TGACTGCAGATACGTCATCAGTCAATGTGGTGGTTATTTACTATGACACTGATAGTCTAAATGATGTGAGCTACAA
CATAGTACAAAACTTAATGACATGGAAAGTCTGGATGACAAACTCACAATGGCAACCTGTCCTGGATGGAAGAAAT
TTCATACTTGACTCATTCCATGGGGTTCTTGTTTTTTCACACCACTTTCAAGAGATTTCAGGTTTCAAAAATTATG
TCCAGGAAGCCACTCCTTTCAAATACCCAGAAGACACTTATCTCTTTTTGTACTGGTACAAGAATTTCCACTGCTC
CTTCTCTGAGTCTGACTGTGCATTGGAGAACTGTATACCTAATGCCTCTCTAGCTTGGTTGCCTCCAAATCATTTT
GACACAGCCATGAATGACAGGAGCTACAATATATAC
>cv36 .
GGGCCTGCTCACCACAGAAGGCCATAAGGGTTTTCAGTTTCTCTCAGATGTGAGAACTGAGATGGACAGGAACAGA
GTCTGTGTAGCCTTTGTGAAAATTGTGGCAAGTATCACTGTGTCATATTTCTCAGTCATGCAGAAACATGATATCC
TGACTGCAGATACATCATCAGTGAATGTGGTGGTTATTTACTATGACACTGATAGTCTAAATGATGTGAGCTACAA
CATAGTGCAAAACTTAATGACATGGAAAATCTGGGTGACAAACTCACAATGGCAAGCAGACCTGGATGGAAGAAAT
TTCATACTTGACTCTTTCCATGGGGTTCTTGTTTTTTCACACCACTATGAAGAGATTTCAGGTTTCAAAAATTATG
TCCAGGAAGCCACTCCTTTCAAGTACCCAGAAGACACTTATCTCTTTATGTACTGGTACAACAATTTCCACTGCTC
CTTCTCTGAGTCTGACTGTGCTTTGGAGAACTGTATACCTAATGCCTCTCTAGCTTGGTTGCCTTCAAATCGTTTT
GACACAACCATGAGTGACAGGAGCTACAATATATAC
>cv39 .
GGGCCTGCTCACCACAGAAGGCCATAAAGGTCTTCAGTTTATCTCAGATATGAGAACTGAAATGGACAGGGGCAGA
GTCTGTGTAGCCTTTGTGAAAATTGTGTCAAGTGTCACTGTGTCATATCTCTCAGCCATGCAGAAATATGATATCT
TGACTGCAGATACATCATCAGTCAATGTGGTGGTTATTTACTATGATACAGATAGTCAAAATGATGTGAGCTACAA
CATAGTTCAAAACTTAATGACATGGAAAGTCTGGATGACAAACTCACGATGGCAAGCTGACCTCGATGGAAGAAAT
TTCATACTTGACCCCTTCCATGGGGCTTTTGTTTTTACACAACACCATGAAGAGATTTCAGGTTTCAAAAATTATG
TCCAGGAAGCTACCCCTTTCAAATATCCAGATGACACTTACCTCTTTGTGTATTGGTACAAGAATTTCCACTGCTC
ATTCTTTGAGCCTGACTGTGCTTTGAAGAACTGTTCACCTAATAGCTCTCTGGCTTGGTTGCCCCCGAGTCATTTT
GACACAGCCATGAGTGACAGGAGTTACAATATATAC
>cv40 .
GGATGATTATTTCAGAAGATGAGAAATTTCTCTCAGCCTTGAGAGGGGAGAGAGAAACAAAGTGTGTTGCCTTTGT
GACTCTGATTCCAAGCTCCTTGCTGTTCTTTCTAAGGCGTGATATGTGTCACAATCAAATCAGAATATCATCAGCA
AACGTTGCTATCGTCTTTGGTGATGATGACTCCTATGTAGGTATGGCCTTGAAACGATGGGACTATTCGATCACAA
CAACTGTCTGGGTCACCACCTCACAATGGATGTCCCCACCGGCTGCAGACATTGCTTTGTTGACTCCTTCCACAAG
ACCTCATTTTCTCTCACTGCCACAGTGACGTTTCTGACTGCAAATTTCATCCGAACAGTGAAGCCTTCCCAATACC
CTGAAGACATTTTCCTTGCCAGATTGTGGCAGAGGTTTTTTAATTGCTCAGGCTCGGGGTCTGACTGCAGATCACT
GGAGAACAGTTCATCTTCTGGCTCCTTGGAAGGGTTACCTGGGCCCAGCACTGACACCGCCATGAGTGATAGGAGT
TATGATGTCTAC
>cv14_PS .
GGGCCTGCTCATCACAGAAGGCCATAAAGGTCTTCAGTTTCTCTCAGATATGAGAGCTGAGATGGACAGGAACAGA
GTCTGTGTAGCCTTTATGAAAATGGTTTCAACTGCAGCTGTGTCATATCTCTCAGCCTTGTATAAATATGATATCC
TGACTGCAGATACCTCATCAGTCAATGTGGTGGTCATTTATTATGATACTGATAGTATAAATGATGTGAACTACAA
CATAGTACAAAAGTTAGTGACATGGAAAGTCTGGGTGACAAACTCACACTGGCATGGTGAGCTGGATGGGAGAAAT
TTCATACTTGACCCATTCCATGGGACTCTTGCTTTTTCACACCATCATGAAGAGATTTCAGGTTTCAAACATTTTG
TCCAAGAAGCCACCCCCTTCAAATACCCAGAAGACACTTATCTCTTTATGTATTGGTACAAAAATTTCCACTGCTC

CTTCTCTGAGTCGAACTGTGCATTGGAGAACTGTATACCTAATGCCTCTCTAGCTTGGTTGCCTCTGAATTGTTTT
GACACAGCCATGAGTGACAGGAGCTACAATATATAC
>cv20_PS .
GGGCCTGCTCATCACAGAAGGCCACAAGGGTCTTCAGTTTCTCTCAGATGTGAGAACTGAGATGGACAGAAACAGA
GTCTGTGTGGCCTTTGTAAAAATGGTCCCAACTGTGGCTGTGTCATATCTCTCAGGCATGAAGAAATATGATAGTC
TGACTGCAGATACATCATCAGTAAGTGTGGTGGTCATTTACTATGATACTGATAGTCCAAATGATGTGAACTACAA
CATAGTACAAAAGTTAGTGACACGGAGAGTCTGGATGACAAACTCACAATGGCATGGTGACCTGGATAGGAGAAAC
TTCATACTTGACCCATTCCATGGGGTTCTTGTTTTTTCACACCACCATGAAGAGATTTCTGGTTTCAGAAATTTTG
TACAGGAAGCTACCCCTTTCAAATACTCGGAAGACACTTATCTGTTTACATATTGGTATAAGAATTTCCATTGCTC
ATTCTCTGAGTCAGACTGTGCATTGGAGAATTGTTCACTTAATGCCTCTGTAATTTGGTTGCAAATCATTTTGACA
CAGCCATGAGTGACAGGAGCTACAATTTATAC
>cv46_PS .
GGGGCTGCTCATTACAGAAGGCCATAAGGGTCTTCAGTTTCTCTCAGATGTGAGAACTGAGATTAATAAGAACCGA
ATTTGGATAGCCTTTGTAAAATGATCTCAACTATGACTGTGTCATATCTCTCAGGCATACAGAATCATAGTATCCT
GCCTGCAGATACATCTTCAGTCAATGTAGTGGTTATTTACTATGACAGTGATAATCCAAGTGATGTGAGATACATA
TAGTGCAACATTTAGAGTCATGGAAAATCTGGATAACAAACTCACAATGGCAAGCTGACATGGATGGATGAAATTT
CATACATGATCCATTCCATGTTCTTACTTTTTCACACCAGCATGAAGAGATTTTAAATTTTAAAACTTTTGTACAA
AAGCTACCACATTTAAATACTCTGAAGACAATTTTTTGTTATGTATCAATACAAGTATTTCTGTTGCTCCTTCTCT
GAATCTGACTGTGCACTGGAGAACTGTACACCTAATGCCTCTCTGGCTTGGCTGCCTCCAAATAGTTTTGACACAA
CCATGAGTGATAGAAGCTACAATATACAC
>cv48_PS .
GGGGCTGCTCATTACAGAAGGCCATAAGGGTCTTCAGTTTCTCTCAGATATGAGAACTGAGATTAATAAGAACAGA
ATTTGGATAGCCTTTGTAAAATGATCTCAACTATGGCTGTGTCATATCTCTCAGGCATACAGAATCATAGTATCCT
GCCTGCAGATACATCTTCAGTCAATGTAGTGGTTATTTACTATGACAGTGATAATCCAAGTGATGTGAGATACATA
TAGTGCAACATTTAGAGTCATGGAAAATCTGGATAACAAACTCACAATGGCAAGCTGACATGGATGGATGAAATTT
CATACACGATCCATTCCATGTTCTTACTTTTTCACACCAGCATGAAGAGATTTTAAATTTTAAAACTTTTGTACAA
AAGCTACCACATTTAAATACTCTGAAGACAATTTTTTGTTATGTATCAATACAAGTATTTCTGTTGCTCCTTCTCT
GAATCTGACCGTGCATTGGAGAACTGTACACCTAATGCCCCTCTGGCTTGGCTGCCTCCAAATAGTTTTGAGACAG
CCATGAGTGATAGAAGCTACAATATACAC
>cv55_PS .
GGGCCTGCTCATCACAGAAGACCATAAAGGTCTTCAGTTTCTCTCAGATATGAGAGCTGAAATGGACAGGAACAGA
GTCTGTGTAGCCTTTATGAAAATGGTGTCAACTGCAGCTGTATCATATCTCTCAGCTTGCATAAATATGATATCCT
GACCACAGATACATCATCAGTCAATGTGATGGTTATTTATTGTGATATTGATAGTATAAATAATGTGAACTACAAC
ATAGTACAAAAATTACTGACATGGAAAGTCTGGATGACAATCTCAAAATGGCATGATGACCTGGATGGAAGTAATT
TCATACTTGACCCATTCCATGGGGTTCTTGTTTTTTCACACAACCATGATGAGATTTCAGGTTTTTGAAATTTTTG
TACAGGAAGCTACTCCTTTCAAATAACCAGAAGATACTTATCTGTTTACATATTGGTATAAGAATTTCCATTGTTC
ATCCTCTGAGGGTGACTGCACATTAGTGAACTGTACACCCAATGCCTCTGTGATTTGGTTGCCTCCAAATCATTTT
GACACAGTGTTGCAGTCCTAGGTTGTACAGAGAACAA
>cv58_PS .
GGGTTTGCTCATCACAGAAAGCCATAAGGCTGTTCAGTTTCTCTCAGATGTGAGAGCTGAGATGGACAGGAACAGA
GTTTGTGTAGCCTTTGTGAAAATCATCCCAACTGTGGTTGTGTCCTATCTCTCAGGCATACACAATCATGATATTG
TAACTGCAGATACATCATCAGTCAATGTGGTGGTTATCTACTGTGGTACTGATAGTCCAAGTGATATGAGCTACAG
CATGATACAAGACCAGTAACTTGGAAAGTCTGGGCGACAAGCTCACAATTGCAAGCTGACCTGGATGGGAGAAATT
TCATACTTGACCCATGCCATGGGATTCTTCTTTTTTCTCACCACCATGAAGAGATTTCAGGTTTCAAAATTTTTTC
CCAGGAAGCTACCCTATTTAAATACGCAGAAGACACTTATTTGTTTAAGTATTGGCATGAGGATTTCCATTACCCC
TTCTCTGAATCTGATTGTGATTTGGAGAACTGTACACCTAATGCCTTTCTGGCTTGGTTGCTCTGTATCATTTTGA
ATCAGCCATGAGTGATAAGAGCTACAATATATGT
>cv64_PS .
GGGACTGCTCATCACAGAAGGCCCTAAGGGTCTTCAGTTTCTCTCAGATGTGAGTGCTGAGATGGACAGGAACAGA
GTCTGCATATCTTTTGTGAAAATCATCCTAACTGTGACTGTGTTACATTTGTCAGCCACACAGAAACATTATATCC
TGACTGCAGATACATCAGTCAATGTGGAGGTTATTTACTATGATACTGATAGTTGAAATGATGTGAATCAGAACAG
AGTGCAACATTAGTGTCACAGAAAGTTTGGATGACAAATTCATGATGGCAAGCTCAGCTGGAGGGCAGAAAATTCA
TACTTGATCCATTCCATGGGATTCTTATTTTTTCACACAAGCATGAAGAGATTTCAGGTTTCAAAAATTTTGTCAA
AGAAGCCACCCTTTTCAGGAACCCAGAAAACACTATCTCTTTATGTATTGGTACAAGAATTTCCATTGCTCCTTCT
CTGAGTCTGACTATGCATTAGAGAACTGTACACTTAATACTTCTATGGCTTGGTTGCCTCAAAATAGTCTTGATAC
AGCCACGAGTAATGGGAGCTACAGTACATAA
>cv66_PS .
GGGCCTGTTCATCACAGAATACCATAAAGGCCTTCAGTTTCTCTCAGATATGAGAGCTGAAATGGACAGGAACAGA
GTCTGTGTAGCCTTTATGAAAATGGTGTCAACTGCAGCTGTGTCATATCTCTCAGCTATGCATAAATATGATATCC
TGACCACAGATACATCATCAGTCAATGTGGTGGTTATTTATTATGATACTGATAGTATAAATGATATGAACTACAA
CATAGTACAAATATGGAAAGTCTGGATGACAAACTCAAAGTGGCATGGTGACCTGGATGGAAGAAATTTCATCTTG
ACCCATTCCATGGTGTTCTTGTTTTTTCACACAACCATGAAGAGATTTCAGGTTTTTGAAATTTTGTCCAGGAAGC

TACTCCTTTCAAATAACCAGAAGACACTTATCTGTTTACCTATTGGTATAAGAATTTACATTGTTCATCCTCTGAG
GGTGACTGCACATTAGTGAACTGTACGCCCAATGCTTCTGTGATTTGGTTGCCTCCAAATCATTTTGACACAGTGT
TGTAGTCAGGGTTGTGCAGAGAACAA
>cv69_PS .
AGGATTGCTCATTACAGAAGATCATAAAAGTCTTCAGTCTCTCTCAGACATAAAAGCAGAGATGGACAAGAAAGAC
ACTTGTGTTGAAAATCTAAAAATGACCTCAAATCCTTTGGTGTCATATGTCTCAAATATGCAGCAACAATATAGCC
TGACCAAGGAACCAACATCAGTAACATATTATTTACTATGACAGTGATGGTCTAAATGAAGTCAATATTGATATCC
GACAATATTTAGTGATTTGGACAGTCTGGGAGACAATCTCCCAATTGTATGTTGACATGCTTGGGAGAAATTTCAT
CCTTGACTTCATGGTACTTTCATTTTCTTCAAAACACCAGGAGACATCCAGTTTGAAAAATTTTGTCCAAACATCT
AATCCTTTCAAAAACCCAGAAGATACTTACCTTACTATGCTTTGGGGCCCAGAATTTCCATTGCTTTTTCTCAGAT
TCTGATTGTACACATGCAGAGAACATTTTGATATTGTATCTAAAATGTCAAGCACTAAATATTTCTGGTTCATAAA
AATATAATTCTCAATAATGTATAG
>cv70_PS .
AGGACTGCTCATTACAGAAGATCATAAAAGTCTTCAGTCTCTCTCAGACATAAAAGCAGAGATGGACAGGAAAGAC
GCTTGTGTTGAAAATCTAAAAATGACCTCAAATCCTTTGGTGTCATATGTCTCAAATATGCAGCAACAATGTATCC
TGACCAAGGAACCAACATCAGTAACATATTATTTACTATGACAGTGATGGTCTAAATGAAGTCAATATTGATATCC
CACAGTATTTAGTGATTTGGACAGTCTGAGAGACAATCTACCAATTGTATGTTGACATGCTTGGGAGAAATTTCAT
CCTTGACTTCATGGTACTTTCATTTTCTTCAAAAAAGCATGAGACATCCAGTTTGAAAAATTTTGTCCAAACATCT
AGTCCTTTCAAAAACCCAGAAGATACTTACCTTACTATGCATTGGGGCCCCAGAATTTCCATTGCTTTTTCTCAGA
TTCTGATTGTACACATGCAGAGAACATTTTGATATTGTATCTAAAATGTCAAGCACTAAATATTTCTGGTTCATAA
AAATATAATTCTCAATAATGTATTG
>cv73_PS .
GGGCCTGCTCATCACAGAAGAGGATAAGGATCTTTAAGTTTTTCTCAAATGTGAGAGCTGAGAGGGACAGAAACAG
TCTATGGAGCCTTTTTGAAGATTATCCCAACTCTTGTTGTGTCATATCTCTCAGAAACATGATATCCTGACTGCAG
ATGCATCATCATTCAATGTGGTGGTTATTTACTATGATACTGATAGTGAAAATTATGTGACCCACAACATAGTGCA
ACATTTAGTGTCATGGAAAGTCTGGATGACAAATTCATGATGCTAAGCTCAGCTAGAAGGTAGAAAATTTATATTT
GACCCATTATATGTGATTCTTATTTTTTCACACCTGTATGAAGAGATTTCAGGTTTCAAATATTTTGTCAAAGAAG
CCACGCTTTTCACATACCAAGAAGACATTATTTCTTTATGTATCAGTACAAGAATTCCCATTGCTCCTTCTTTGAG
TCTGACTGTGCATTGGAGAACTGTACACTTAATACCTCTGTGGCTTAGTTGCCACCAAATAGTTTTGGCACAGCAA
TGAGTGAAGGGAGTTACAATACATAA
>cv74_PS .
TGGTCTGCTCATCACAGAAGGCCATAATGGCTTTCAGTTTCCTTCAGAAGTGAGAGCTGAGATGGACAGGAACAGA
GTTTGTGTAGCCTTTGTGAAAATGGTCCAAACTGTGGCTGTGTCATATCTCTCAGTCATACACAACTATGATATCC
TGACTACAGATAAATCAGCAGTCAATATGGTGATTATTTACTATGATAGTCAAAGTGATGTGAGCTACAGCATAAT
ACAAGACCAGTGACATGGAAAGTCTGGGGAACAAACACAAACATAAGCTGACCTGGATGGGAGAATTTTCATACTT
GATCCATTCCATGGGATTATTGTTTTTTCTCACCACCATGAAGAGATTTAAGGTTTCAAATATTTTTCCCAAGAAG
CTACCCTGTTCAAATACCCCAAAGACACTTATGTGCTTAAGTATTGGCATAATAATTTCCATTGCCCCTTCTCTGA
ATCTGATTGTTAATTGGAGAACTATACCCCTAATGACTCTCTGGGTTGGTTGCTTTGTATCATTTTGAAACAGCCA
TGAGCAATAAGAGCTTCAGTATATAC
>cv76_PS .
GGGTCAATTCATCACAGGAGGCCATAACAGTCTTCTGCTTCTCAGATGTGAGTGCTAAGATGGAGAGGAACAAAGT
CTGTGTAGTGTTTGTGAAAATGGTCCCAACTCGGGTTGTGTCATTTGTCTGAACCATATAGAAGCCTGATGGAGAT
ACATCATCAGTCAGTGTGGTGGGCTGTTTATTATGATACTGATAGTCAAAATCACATGAGCCACAACATAGTGCAA
AACTTGGTGGCATGGAAATTCTGGATGAAAAACTCATAATGGCAAGCTCAACTGAATGGTAGAAATTTCATACTTG
ACACATTCCACAGGATTCTTGTTTTTTCACACCACCATGAAAATATTGTGGGTTTCAAAAACTTTGTCCAAGAAGT
GATCCCTTGAAAATGCCCAGAAGACACTTATCTCTTTATGTATTGATGCAAGCATTTCCATTGCTTCTTCTCTGAG
TCTGTGAATTGAAGAACTGTATCCTTAATGCCTCTGAATGATTTTGACACAGCCATGAGTGTCAGGAGCTACAATA
TATAC
>ht1 .
AGGACTTGTCATTACAGAAGGTCATAAAGGTCATCAATTTCTTTCCGATGTGCAAGCAGAGATGTCCAGGAACAGA
GTCTGTGCAGCATTTGTGAAAATGCTTCCAACTCCCCTTGCATCCTATATGCCAACTGCACATCAACATACTATCC
TGACTGGGGAAACATCACTGGTAACTGTGGTTGTAATTTACTATGATATTGATGCCCTAAATGATGTAAACTATGC
TATAGAGCCATACATAGCCACATGGAGAGTCTGGATCACAAACTCACAATGGCATGCTGACATGGCTGGGAAAAAT
TTTATACTTAACTCATTCCATGGGACTCTGATTTTTTCAAACCACCATGAGGAAATTTCGGGTTTCAAAAATTTTA
TCCAGGCAGTTAACCCTTTCAAATACCCAGAAGACACTTACCTCACTACTTACTGGTTCAATAATTTTCATTGTTC
ATTCTCTGATTCTGATTGCACATTGAAGGACTGTACACCCAATGCCTCTTTGGCATGGCTGCCTGTGAATCGTTTT
GACATGGCCATGAAGGATGGGAGTTACAATATATAC
>ht2 .
ATCACTGGTAACTGTGGTTGTAATTTACTATGATATTGATGGTCTAAATGATTTAAACTATGCTATAGAGTCATAT
ATAGCCACATGGAGAGTCTGGATCACAAATTCACAATGGCATGCTGACATGGCTGGGAAAAATTTTATACTTGACT
CATTCCATGGGACTCTGATTTTTTCAAACCGCCATGAGGAAATTTTGGGTTTCAAAAATTTTGTCCAGGCAGTTAA
CCCTTTCAAATACCCAGAAGACACTTACCTCACTACTTACTGGTTCAATAATTTTCATTGTTCATTCTCTGATTCT

GATTGCACATTGAAGGACTGTACACCCAATGCCTCTTTGGCATGGCTGCCTGTGAATCGTTTTGACATGGCCATGA
AGGATGGGAGTTACAATATATAC
>ht3 .
AGGACTTGTCATTACAGAAGGTCATAAAGGTCATCAATTTCTTTCCGATGTGCAAGCAGAGATGTCCAGGAACAGA
GTCTGTGCAGCATTTGTGAAAATGCTTCCAACTCCCCTTGCATCCTATATGCCAACTGCACATCAACATACTATCC
TGACTGGGGAAACATCACTGGTAACTGTGGTTGTAATTTACTATGATATTGATGGTCTAAATGATTTAAACTATGC
TATAGAGTCATATATAGCCACATGGAGAGTCTGGATCACAAATTCACAATGGCATGCTGACATGGCTGGGAAAAAT
TTTATACTTGACTCATTCCATGGGACTCTGATTTTTTCAAACCGCCATGAGGAAATTTTGGGTTTCAAAAATTTT
>ht4 .
GGGACTGGTCATCCCAGAAGGTCATAAAGGTCTTCAATTTCTCTCAGATGTGAGAGTGGAGATGGACAAGAGCAGA
ATCTGTGTAGCATTTGTGAAAACGATCTCAACTCCTCTTGTGTCCTTTGCCTCAGTTGTACAGCAAAAATTTATCC
TGACCACGGAAACATCATCAGTAAATGTGGTTATCATTTACTATGATACTGATGGTCTAGATGATGTAACCTATAA
TATAGGGCAATATGTAGTGACATGGCAAGTCTGGGTGACAAACTCACAATGGCATGCTGACATGGCTGGAAGAAAT
TTTATACTTGGCTCATTCCATGGGACTCTCATTTTTTCAATACATCATGAAGAGATTTCTGGTTTTAAAAATTTTC
TCCAGGCAGCCAGCCCTTCCAAATACCCAGAAGACACTTACCTCACTAAGTATTGGTTCCAGAATTTCCATTGCTC
TTTTTCTGATTCTGACTGTGCATTGAAGGACTGTACACCCAATGCCTCTTTGGCCTGGTTGCCTGTGAATCGTTTT
GACCCGGCTATGAGTGATGAGAGTTACAATATATAC
>ht7_PS .
GGGACTGGTTATTGGAGAAGGTCATAAAGGTCTTCAGTTTCTCTTAGACTTCAGAAGAGAGATGGACAGACAGAGA
GTCTATGTTGCATTTATTAAAATGGTCACAAATGATTTTAGGTCATGTTTGTCAAGTTCAGGGACTTATCATCTGT
GGAGCAGAGACACATCCCTAGGAAATGTTGTGATCATTTATCTTGATACAGATAGTACAATCAATATCAAGTTTCT
TGTATTGAAAAATTTAGTAACATGGACGATCTGGGTTACAAACTCACAGTGACAGGCTACAATCACCGGAAACAAT
TTCATACTTGACTCATTCCAAGGGACTCTCATTTCTACAAACCACCATGAGGGGATTTCTTGTTTTAAAATTTTTG
TACAGTCAACTAACCCCTCCAAATACCCAGAAGATACTCACCTCACTATATACTTGATGAAGTATTTAGATTGTTC
ATTCCCCCATTTCGACTGAGCACTGAAGAACTGTCCCCCCAGTGCCTCTCTGGCACAGTTGCCTGTGAATCTTATT
GACACAAGGATGACAGATTGGAGTTACAATATATAT
>ht8 .
GGGACTGGTCATTACGGAAGGTCAGAAAGGTCTTCAATTTCTCTCAGATGTGACAGGAGAGATGGACAGGAACAGA
GTGTGTGTAGCCTTTGTGAAAATGGTCTCAACTCCTCTTGTGTCATACATAGCAAGTGCAAATGAACACATTATCC
TGACCAAGGAAACATCACAAGTCAATGTGGTCATCATTTACTATGACACTGATATTCTAAATGATGTAAACTATAT
TATAGAGCAATATTTATTAACAGGCAAAGTCTGGGTCACAAACTCACAATGGCATGCGGACATGACTGGGAAAAAT
TTCATTCTCAATTCATTCCATGGGAGTCTCATTTTTTCAAACCACCCTGAAAACATTTCTGGTTTCAAAACTTTTG
TCCAAGAAGCCAAGCCTTCCAAATACCCAGAAGACTTTTACCTCACTATGTTTTGGTTCAATAATTTCTACTGCTC
ATTCTCTAACTCTGACTGTTCACTGAAGGACTGTACACCTAATGCCTCTCTGGCATGGCTGCCTGTGAATCGTTTT
GACACAGCCATGACCGACCAGAGTCACAATATCTAC
>ht9_PS .
GGGACTGGTCATTACGGAAGGTCAGAAAGGTCTTCAATTTCTCTCAGATGTGACAGGAGAAATGGACAGGAACAGA
GTGTGTGTAGCCTTTGTGAAAATGGTCTCAACTCCTCTTGTGTCATACATAGCAAGTGCAAATGAACACATTATCC
TGACCAAGGAAACATCACAAGTCAATGTGGTCATCATTTACTATGACACTGATATTCTAAATGATGTAAACTATAT
TATAGAGCAATATTTATTAACAGGCAAAGTCTGGGTCACAAACTCACAATGGCATGCGGACATGACTGGGAAAAAT
TTCATTCTCAATTCATTCCATGGGAGTCTCATTTTTTCAAACCACCCTGAAAACATTTCTGGTTTCAAAACTTTTG
TCCAAGAAGCCAAGCCTTCCAAATACCCAGAAGACTTTTACCTCACTATGTTTTGGTTCAATAATTTCTACTGCTC
ATTCTCTAACTCTGACTGTTCACTGAAGGACTGTACACCTAATGCCTCTCTGGCATGGCTGCCTGTGAATCGTTTT
GACACAGCCATGACCGACCAGAGTCACAATATCTAC
>ht12_PS .
GGGACTGGTCATTACGGAAGGTCAGAAAGGTCTTCAATTTCTCTCAGATGTGACAGGAGAGATGGACAGGAACAAA
GTCTGTGTAGCCTTTGTGAAAATGGTCCCAACTCCTCTTGTGTCATACATAGCAAGTGCAAATGAACACGTTATCA
TGACCAAGGAAACATCACAAGTCAATGTGGTCATCATTTACTATGACACTGATATTCTAAATGATGTAAACTATAT
TATAGTGCAATATTTATTAACAGGCAAAGTCTGGGTCACAAACTCACAGTGGCATGCTGACATGACTGGGAAAAAT
TTCATTCTCAATTCATTCCATGGGAGTCTCATTTTTTCAAACCACCGTGAAAACATTTCTGGTTTCAAAACTTTTG
TCCAAGAAGCCAACCCTTCCAAATACCCAGAAGACTTTTACCTCACTATGTTTTGGTTCAATAATTTCTACTGCTC
ATTCTCTAACTCTGACTGTTCACTGAAGGACTGTACACCTAATGCCTCTCTGGCATGGCTGCCTGTGAATCATTTT
GACACAACCATGACTGATCAGAGTCACAATATCTAA
>ht16 .
GGGACTGCTCATTATGGAAGGTCAGAAAGGTCTTAAATTTCTCTCAGATGTGACAGGAGAGATGGACAGGAACAAA
ATCTGTGTAGCATTTGTGAAAATGGTCTCAACCGTTCTTGTGTCCTATGTGACACTTTCAAATGAACGCATTATCC
TGACCAAGGAAGCATCACAAGTAAATGTGATCATCATTTACTATGACACTGATATTATAAATGATGTAAACTATAA
CATAGAGCAATACTTACTAACAGGCAATGTCTGGGTCACAAACTCACAATGGCACGCTGACATGACTGGGAAAAAA
TTCATACTTAGTTCATTCCATGGGACTTTCATTTTTTTAAACCACCGTGAGAAAATTTCTGGTTTCAAAACTTTTG
TCCAAGAAGCCAATCCTTCCAAATACCCAGAAGACTTGTATCTCACTGTGTTTTGGTTAGAGAATTTCCACTGCTC
ATTCTCTGTTTATGACTGCTCACTGAAAGACTGTACACCTAATGCCTCTCTGGCATGGCTGCCTGTGAATCAATTT
TACACAGCCATGACTGACTGGAGTCACAATATATAC

>ht18_PS .
GGGACTGATCATTATGGAAGGTCAGAAAGGTCTTCAATTTCTCTCAGATGTGATAGTAGAGATGGAAAGGAGCAAA
GTATGTGTAGCCTTTGTGAAAATGGTCTCAACTCCTGTTACATCATATGCGACAATTGCAAAAGAACACATTATCC
CAGCAAAGGAAATATCACATGTCATTGTGGTCATCATTTACTATGACACTGATACTCTCAATGATATAAACTATAG
TATAGAGCAAAATTCACTAACAGGCAAAGTCTGGGTCACAAACTCACAGTGGCATGCTGACATGATTGGGAAAAAT
TTCATACTTAATTCATTCCAGGGAACTCTCATTTTTTCAAATCACCATGAGAAAATTTCTGGTTTCAAAACCTTTG
TCCAAGAAGCTAAGCCTTCCAAATATCCAGAAGAATTTTACCTCACTATGTTTTGGTTCAATAATTTCCATTGCTC
ATTCTCCGATTCTGATTGTTTACTGAAGTACTGTACACCTAATGCCTCTCTGGCATGGCTGCCTATGAATCATTTT
GACACAGCCATGTCTGACTGGAGTCAAAATGTACAC
>ht20 .
GGAACTGGTCATTATGGAAGGTCAGAAGGGTCTTCAATTTCTCTCAGATGTCAGAGGAGAGATGGACAGAAACAAA
GTCTGTGTAGCCTTTGAGAAAATGGTCCTGACTCCTCTTGTGTCATATGTAGCATTTGCAAAGGAACACATTATCC
TGACCAAGAAAACATCACAAGTCAACGTGGTCATCATTTACTATGACACTGATATTCTAAATGATGTAAACTATAG
TATAGGGCAATATTTACTAACAGGCAATGTCTGGGTCACAAACTCACAATGGCATGCTGATATAACTGGGAAAAAT
TTCATACTTAATTCATTCCATGGAATTCTCATTCTTTCAAACCACCATGAGAACTTTTCTGGTTTCAAAACTTTTG
TTGAAGAGGCCAACCCCTCCAAATACCCAGAAGACTTTTACCTCACTGTGTTTTGGTTCAATAATTTCCACTGCTC
ATTTTCTGATTCTGACTGCTCACTAAAGGAATGTACACCCAGTGCCTCTGTGGCATGGCTGCCTGTGAATTGTTTT
GACACAGCCATGACTGACAGGAGTCACAATGTACAC
>ht24_PS .
GAGCTTGCTCATCACAGAGGGCCATAAGGGTGATCCATTTCAAATGGACAGGAACAGAGTCTGTGTAGCCTTTGTG
AAAATGGTATCAACAGTTCCTTTGTCATATCTCTCAGCCACACAGAAACATGATATTCTGACCAGGGAAACATCAT
CAGTCAATGTGGTGGTTATTTACTATGATACTGATGCTTGAAATGATGTAAACTACAACATATGGTGATACTTAGT
GATATGAAAAGTCTGGGTGACAAACTCACAGTGCCATGCTGACCTGGCTGGGAGAAATTTTATACTTGACCCATTC
CATGGGATTCTTGTTTTTTCACACCACCATGAATGAATTTCAGGTTTCAAACAATTTGTCCAGGCAGCTACCCTTT
TCAAATACCCAGAAAACACTTATATCACTATGTATTGGTCCAAGAATTTCCAGTGCTCTTTCTCTGACTCTGACTG
TGCATTGAAGAACTGTACACCTAATGCCTCTGTGGCTTGGTGGCCTCCAAATCATTTTGACACAGCTATGAGTGAT
GAGAGCTACAATATATAC
>ht26_PS .
GGGACTGGTTATCATGGAAGGTCAGAAAGGTCTTCAATTTCTCTCAGATGTGATAGTAGAGATGGACAGGAACAAA
GTATTTGTAGCCTTCATGAAAATGGACTCAACTCCTCTTATGTCATATGTGAAAATTGCAAAAGAACACATTATCA
TGACAAAAGAAATATCACGTCAATCTGGTCATCACTTACTATGACTCTGGTACTCCCAATTATATAAACTATAGTA
TAGAGCAATATTTACTAACAGGCAAAGTCTGGGTCACAAACTCACAATGGCATGCTGACATGACTGGGAAAAATTT
CATTCTCAATTCATTCCAGGGGACTCTCATTTTTTCAAACCACCATGAGAAAATTTCTGGTTTCAAAACCTTTGTC
CAAGAAGCCAAGCCCTCCAAATATCTAGAAGACTTTTATCTAACTATGTTCTGGTTCAATAATTTCCACTGCTCAT
TCTCTAACTCTGACTGTTCCCTGAAGGACTGTACACCTAATGCCTCTCTGGCATGGCTGCCTGTGAATCATTTTGA
CACAGCCATGACCAAACAGAGTCACAATGTACAC
>ht28 .
GAGACTGATCATTACAGAAAGCCAGAATGGTTTTTGGTTTCTCTCAGATGTGAGGGGAGAGATGGACAGGAATGGA
GTTTGTGTACCATTTGTAAAAATGATTGCAAATGCTTTTGTATCCTACCTCTCAGTTACAATGCTGCAAGATTTCC
TGACTAGGGAAATGCCACCAGTAAATGTGGTTATTATTTACTATGATACTGATAGTCTAAATCATATCAACTATCA
GATAGGGGTACATTTAGTGACATGGAGAGTCTGGGGGGTCACAAATCAATGGCATGCTGACATGGCTGGGAGAAAT
TTCGTCCTTGATTCATTCCATGGGACTCTCATTCTTTCACACCTCCATGATGAAATTTCTGGTTTTAATAAGTTTA
TCCAGATAGCTACCCCCTTCAAATACCCAGAAGACACTTTTCTCACTCTGTATTGGTTCCAGAATTTCCACTGCTC
ATTCTCTGATTCTGACTGTTCGCTGAAGGACTGTACACCTAATGCTTCTCTGGCATGGCTGTCTGTGAATCATTTT
GACACAGCCATGAGTGAAGGGAGCTACAACACATAC
>ht33_PS .
GGGACTGGTCATCACAGAAGGTCACAGAGGTCTTCAGTTCCTCTCAGATGTGAGAACAGAGATGGACAGGAGCAGA
GTCTGCATAGCATTTGTGAAAATGATCTCAAATTTTCTTGACTTGTATCTGTCAACTGCACAGAAACTAGATTTCC
TGACCAGAGAAACATCACCAGTAATTGTAGTTGTCATTTACTATGATCCTGAGAGTCTAAATGATATAAACTACCA
GATAGGGCAATATTTAGTGACCTGGAGGATCTGGGTCACAAACTCACAATGGCATGCTGATGTGGCTGGCAGAAAT
TTGATACGTGACTCATTCCATGGGACTCTCATTTTTTCACACCACCATAAGGAAATTCCAGATTTTAAAAATTTTA
TCTACACTGCTAACCCCTCCAAGTACCCACAAGACACTTTCCTCATTCTGTATTGGTTCCAGAATTTTCATTGCTC
AGTATCTGATTCTGATTGCTTGCTCACTGAGGAAATGTACACCTAATGCCTCTCTGGCAGGGCTGCCTGTGAATCA
TTTTTACCTGACAATGAGTGATGGGAGCTGCAACACACAT
>ht37_PS .
TGGCCTGGGCATCACAGTTGGGCCATAAGGGTTGTCAGTTTTTCTCAGACATAAGAGCTGAGATGGATAGGAACAG
AGTCTGTGTAGTCTGTGGGAAAATGGTCTCAACTGCTGCTGTCTCCTATCAGCCACACAGAAACATGAAATTCTGA
CCATGGAAACATCGACAGTCAGTGTGGTGGTTATTTGTTATGATACTGAAAGTTGAAATGATGTAACATATAGCAT
ATGGCAACACTTAGTGACATGGAAAGTCTGGGTGACAAACTCACAATGGCATGCTGACCCAGCTGGGAAAAATTTC
ATATTTGACCCATTCCATGGGATTCTTGTTTTTTCACACCACTATGAAGAAATTTCAGGTTTCAAACATTTTGTCC
AGGCAGCGACCTCTTTCAAATACCCAGAAGACACTTAACCTTGTTATGTGTTGCCCCAAGAATTTCCAGTGCTCAT

TCTCTGACTCTGACTGTGCATTGAAAAACTGTACACCTAATGCCTCTGTGGCTTGGTTGCCTCTGAATCGTTTTGA
CACAGCCATGAGTGATGGGAGTTGTCATATATAC
>ht39_PS .
GGGACTGATCATCACAGAAGGTCAGAAAAGTCTTCAATTCCTTTCAGATTTGAGAGGAGAGATGGACAAGAACAGA
GTCTGTGTAGCATTTGTGAAAGTGATCTCAAATGCTCCTGTTTCATATGTCTCAACTGCTAAGCCACGTGATTTCC
TAAGCAAAGAAATATTACCAGTAAATGCAGTTATCATTTACTTTGATTCTGTGAGTCTAAATGATATAAACTATCA
TGTAAGGCTATATTTATTGACATGCAGAGTGGATCACAAACTCAGAATGGCATGCTGATATGGCTGGGAGAAACTT
GACCTTTGATTCTTTCCATCCATGGGACTCTGATTTTTTCACAACACCATGAGATTTCTTGATTTAAAAATTTCAT
CCAAACTATTAACCCCTAAACATAACCAGAAGATACTTTCCTCACTCTGTATTGGTTCCAGAATTTCCATTGTTTG
TTTTCTGATTCTGTCTGTTCTTTCAAGGACTGTACACCTAATGCCTCTTTGGCATGGTTGCCTATGAATCATTTTG
ACTTGGCCATGAGTGATAGGAGCTACAACATATAC
>ht40_PS .
GGGACTGGTCATCACAGAAGGTCAGAAAGGTCTTCAATTCCTTTCAGATTTGAGAGGAGAGATGGACAAGAACAGA
GTCTGTGTAGCATTTGTGAAAGTGATCTCAAATGCTCCTGTTTCATATGTCTCAACTGCTAAGCAACATGATTTCC
TAAGCAAAAAAACATTACCAGTAAATGCAGTTATCATTTACTTTGATTCTGTGAGTCTAAATGATATAAACTATCA
TTTAAGGCTATATTTATTGACATGCAGAGTGGATCACAAACTCAGAATGGCATGCTGATATGGCTGGGAGAAACTT
GACCTTTGATTCTTTCCATCCATGGGACTCTGATTTTTTCACAACACCATGAGATTTCTTGACTTAAAAATTTCAT
CCGAACAATTAACCCCTACACATAACCAGAAGATGCTTTCCTCACTCTGTATTGGTTCCAGAATTTCCACTGTTTG
TTCTCTGATTCTGTCTGTTCTTTCAAGGACTGTACACCTAATGCCTCTTTGGCATGGTTGCCTATGAATCATTTTG
ACTTGGCCATGAGTGATAGGAGCTACACCATATAC
>ht41_PS .
GGAACTGGTCATTATGGAAAGCCAGAAAGGTCTTCCATTTGCTTCAATGTGACAGGAGTGATGGACAGGAACAAAG
TCTATGTAGCCTTTTGTGAAAATGATCTTAAGATTTCTTACATTGTTTGTGGTATTTGTAAAGGAACATATTATCC
TGACTAAGGTATCATCAAAAATAAATGTGGTCATTTATCAGTGTCATTAATTATGACACTGATACTTGATATGATG
TAAAGTATAGCATAGAGCAGTCTTTACTAAGAGGAAAATTCTGGGTCACAAACTGAGAATGGCGTGCTGACATGGC
TGGGAAAAATTTCATGCTTAATTAATTCCATGGGACTCATTTTTTCAAACCACTATGAGAAAATTTCTGGTTTCAA
AGCTTTTACACAAGAAGCTAACCCTTCCAAATACACAGAAGAAACTTACCTCACTGTTTTGGTTCCATAATTTCCA
CTGCTCATTCTCTGATGCTGACTGTTCACTGAAAGACTGTACACCTAATACCTCTCTGGCATAGCTGCCTGTGAGT
CATTTTGACACAGCCATGATTGACTGGAGTCACAATATACAC
>ht42_PS .
GGGACTGGTCATTATGGAATGTCCAAAAGGTCTTCAATTTCTCTCAGGTGTGGCAAGAGAGATGGACAGGAACAAA
ATCTGTGTGGTGTTTGTGAAAATGGTCTCAAGATTTTGTATATATTCTGTGGCACTCACAAAGGAACACACTATCC
TGACTAACGAATCATCAAAAGTAAATGTGGTCATCATTTACTATGACACTGAAATTCGATATGACACAAAGTATAG
TATAGAGTGCTATTTACTAAGAGGCAAAGTTTTGTTCACAAACTCACAATGGCATGCTGACATGACTGGGAAAAAA
AATCATACTTAATTAATTCTATGTTACTCTCAATTTTTCAAATCACCATGAGAAAATTTCTGGTTTCCAGACTTCG
GTCGAAGAAGCCAACCCTTCCAAATACCCAGAAGACACTTACTTCACTATGTTTTGGTTCAATAATTTCCACTGCT
CATTCTCTGACTCTGACTGTTCACTGAAGGACTGTACACCTAATACCTCTCTGGCATGGCTGCCTGTGAATCATTT
TGACACAACCATGACTGATCAGAGTCACAATATCTAA
>ht44_PS .
AGGGCTGATCATCACAGAAAGTCCAAAAAGTCTTCAATTTCTCTCAGATATAAAACCAGAGATGGACAGGAATGGA
GTCTGTGTTGGATATGTAAAAACAGTCTTAACTCCTCTTGTGTCATAGGTCTCAAAAGTGCAGCAAAACATACCCT
GACCAGGGAGCAAACATTAGACGTGGTTGTTATTTACTATGATAGCGATGGTCTAAATGAAGTCAGTGTTGACATT
TGGCAATATTTAATGATGTGGAGACTCTTGGTGACAAAATTCGCAGTGGCATGCTGACATGGCTGGGAGAAATTTC
AAACTTGACTCATTCCATGGTATTCTCATTTTCTCAAAATATCGTGATGAGATTTCTGTTTAAAAACTTTGTTCAG
ACAGCTAATCCTTCCAAATACCCAGAAGACACTTACCTCACTCTCTATTGGTCCCAGAATTTCCATTGCTCATTCT
CTGATTCTGATGATTGTGAACTAAAGGACTCTACAACCAAGGCTTCTTTGGTACAGTTGTCTGTGAGTCATTTTGA
CACAGCCATGACTGATGGGAGTTACAATATATAC
>ht46 .
GAGACTGATCATTACAGAAAGCCAGAATGGTTTTTGGTTTCTCTCAGATGTGAGGGGAGAGATGGACAGGAATGGA
GTTTGTGTACCATTTGTAAAAATGATTGCAAATGCTTTTGTATCCTACCTCTCAGTTACAATGCTGCAAGATTTCC
TGACTAGGGAAATGCCACCAGTAAATGTGGTTATTATTTACTATGATACTGATAGTCTAAATCATATCAACTATCA
GATAGGGGTACATTTAGTGACATGGAGAGTCTGGGTCACAAACTCTCAATGGCATGCTGACATGGCTGGGAGAAAT
TTCATCCTTGATTCATTCCATGGGACTCTCATTCTTTCACACCACCATGATGAAATGTCTGGTTTTAATAAGTTTA
TCCAGATAGCTACCCCCTTCAAATACCCAGAAGACACTTTTCTCACTCTGTATTGGTTCCAGAATTTCCACTGCTC
ATTCTCTGATTCTGACTGTTCACTGAAGGACTGTACACCTAATGCTTCTCTGGCATGGCTCCCTGTAAAT
>ht48_PS .
GGGACTGGTCATCACAGAAGGCCAGAATGTTTTTCAGTTTCTCTCAGATGTGAAGGGTGAGATGGACAGGAATGGA
GTCTGTGTAGCATTAGTAAAAATTATCTCAAATACTCTTGTATCACCCTTCTGGAGTCCAAAGCTACAAGATTTCC
TGAGCATAGAAACATCACCCGTAAATGTGGTTATCATTATGATGATACTGACAATCTAAATGATATAAACTATCAG
GTAGTGCCACATTTAGTGACATGGAGAGTTTGGGTCACAAAGTCAGATTGGCATGCTGACCTGGCTGGGATAAATT
TAATCCTTGACTCATTCCATGGGACTCTCATTTTTTCACAACACCATGGGGAGATTTCTGGTTTTAATGAGTTTAT
CAAAACTACCCCCTTCAAATACCTAGAAGATACTTTCTTCACTTTCCTCAGAATTTCCACTGCTCGTTCTCTGATT

GTGACTGTTCACTGAAGGACTGTACACCTAATGCCTCTCTGTCATGGCTGCTTTCAAATCATTTTGACACACCCAT
GAGTGAGGGAGCTACAACACATAC
>ht50_PS .
GGGACTGGTCATCACAGAAGGCCAGAATGTTTTTCAGTTTCTCTCAGATGTGAAGGGTGAGATGGACAGGAATGGA
GTCTGTGTAGCATTAGTAAAAATTATCTCAAATACTCTTGTATCACCCTTCTGGAGTCCAAAGCTACAAGATTTCC
TGAGCATAGAAACATCACCCGTAAATGTGGTTATCATTATGATGATACTGACAATCTAAATGATATAAACTATCAG
GTAGTGCCACATTTAGTGACATGGAGAGTTTGGGTCACAAAGTCAGAATGGCATGCTGACCTGGCTGGGATAAATT
TAATCCTTGACTCATTCCATGGGACTCTCATTTTTTCACAACACCATGGGGAGATTTCTGGTTTTAATGAGTTTAT
CAAAACTACCCCCTTCAAATACCTAGAAGATACTTTCTTCACTTTCCTCAGAATTTCCACTGCTCATTCTCTGATT
GTGACTGTTCACTGAAGGACTGTACACCTAATGCCTCTCTGTCATGGCTGCTTTCAAATCATTTTGACACACCCAT
GAGTGAGGGAGCTACAACACATAC
>ht53_ .
GGGACTGGTCATTGCAGAGTGTCAGAAAGGTCTTCAATTTCTCTCAGACTTGCGGGCAGAGATGGACAGGAACAGA
GTCTGTGTAGCATTTGTAAAAATGATCTTAAACAGTTACATCCAAAACGAATCAAATAGACTAACATATGATTTAT
GGACCAGAGAAACATCACCAGCAAATGTTGTTATTATCTACTTTGATACTAATACTGCACATGATGCTGGATTTTA
CACTGGGAAATATTTAGTGACATGGAGAGTCTGGGTCACAAACTCACAATGGCATCCTGACATCTTTGGGAAAAAT
TTCATAATTGACTCTTTCCATGGAACACTCATTTTCTCACTCCAGCATGAGGAGATTTCTGGTTTTAAAACTTTTA
TCCAGACAGCTACCCCCTCAAAATATCCAGAAGACATTTACTTCACTATATTTTGGTCCATGAACTTTGACTGCAC
TTTATCTAATTTTGATTGTACATTGAGGAACTGCCCAGCTAATGCTTCTCTCATGTATCTTCCTGTAAGTCGTTTT
GACCCTGCCATGAGTGATGAGAGTTATAATATCTAC
>ht54 .
GGGACTGGTCATTGCAGAGTGTCAGAAAGGTCTTCAATTTCTCTCAGACTTGCGGGCAGAGATGGACAGGAACAGA
GTCTGTGTAGCATTTGTAAAAATGATCTTAAACAGTTACATCCAAAACGAATCAAATAGACTAACATATGATTTAT
GGACCAGAGAAACATCACCAGCAAATGTTGTTATTATCTACTTTGATACTAATACTGCACATGATGCTGGATTTTA
CACTGGGAAATATTTAGTGACATGGAGAGTCTGGGTCACAAACTCACAATGGCATCCTGACATCTTTGGGAAAAAT
TTCATAATTGACTCTTTCCATGGAACACTCATTTTCTCACTCCAGCATGAGGAGATTTCTGGTTTTAAAACTTTTA
TCCAGACAGCTACCCCCTCAAAATATCCAGAAGACATTTACTTCACTATATTTTGGTCCATGAACTTTGACTGCAC
TTTATCTAATTTTGATTGTACATTGAGGAACTGCCCAGCTAATGCTTCTCTCATGTATCTTCCTGTAAGTCGTTTT
GACCCTGCCATGAGTGATGAGAGTTATAATATCTAC
>ht55_PS .
GGGGCTGGTCATCATGGAAGTCAGCAAGGTCTTTAATTTCTCTCAGATGTGATAGTAGAGATGGACAGGAACAAAG
TATGTGTAGCCTTCCTGAAAATAGACTCAACTCCTCTTATGTCATATGTGAAAATTGCAAAGGAACACATTATCCT
GAAAAAACAAATATCACATCAATGTGGTCATCATTCACTATGACCCCGGTACTCCCAATTATATAAACTGTAGTAG
AGAGCAATATTTACTAACAGGCAAAATCTGGGCCACAAACTCATGCTGACATGACTGGGAAAAAATTTCATTCTCAA
TTCATTCCAGGGGACTCTCATTTTTTCAAACCACCATGAGAAAATTTCTGGTTTCAAAACTTTTGTCCAAGAAGCC
AAGCCCTCCAAATATCTAGAAGACTTTTATCTAACTATGTCACTGTGTTCTGGTTCAATAATTCCTACTGCTCATT
CTCTAACTCTGACTGTTCCCTGAAGGACTGTACACCTAATGCTTCTCTGGCATGGCTGCCTGTGAATCATTTTGAC
ACAGCCATAACTGAACAGAGTCACAATGTACAC
>ht61_PS .
GGGACTGGTCATTGCAGAGAGTGAGAAAGGTCTTCCATTTCTCTCAGACTTAAGGGAAGACATGGACAGGAGTAGA
ATCTGTGTAGCATTTGTAAAAATGATCTCAAACAGTGCCATGAGATATTTATGAAATCGACCAACATATGATTTAT
GGACCAGAGAAACATCACCAGCAAGTGTTATTGTCATCTACTTTGATACTAACAAAGTGATGTTAGATTTTACACT
GGATAATATTTAGTGACATGGAGAGTCTGGGTCACAAACTCACAATGACATTTTGCCACCTTTGAGGAAAATTTCA
TAATTGACCCTTTCCATGGTACACTAATTTTCTCACACCAGCATGAGGAGATTTCTGGTTTTAAAGCTTTCATCCA
GACAGCTACCCCTTCAAATACCCAGAAGATATTTACCTGTCTATATTTTGGTCCATGAACTTTGACTGCACTTTAT
CTAATTTTGATTGTACATTGAAGAAATGCCCACCCAATGCTTCTCTCATGTATCTTCCTGTGAGTTGTTTTGACCC
TGCCATGAGCGATGAGAGTTATGATATCTAC
>ht64_PS .
GGGATTGATAATTGCAGAGGGTCAGGAAGATCTTCAGTTTCTCTCAGACTTGTGGAAAGAGATGTACAGGAACAGA
ATCTGTGTAGTATTTGTGAAAATGATCTCAATCAGTGCCATACTACACTCATCAAATAGACCAACATATGACTTAT
GGACCACACAAAGAAACATTACCAGAAAATGGTGTTATTATATACTTTGATACTAATAGTGTAAGTGCTGGAGCAT
TTTACACTAGGCAGTATTTAGTGATGCGGAGAGTCTTGGTCACAAACTCACAATGGCATTCTGCAACCTTTGGGGA
AAATTTCATAATTGACTCATTCCACTCATTTTTTCAAACCAATATGAGGAGATTTATGGTTTCAAAACTTTTATCC
AGACAGCTACCCCTTCAAAATACCCAGAAGATATTTACCTCATTGTATTTTGGTCCATGAAATTTTACTGCACTTT
ATCTAGTTTTGATTGTGCATTGAAGAACTTCCAATCCAATGTTTCTTTCACATGTTTGCCTGCGTTGTTTTTATCC
AGCCATGTGTGACAAGAGTTACAATACCTAC
>ht68_PS .
GGGACTGGTCATCACAGCAGGTCAGAATGGTTTTCAATTTCTCTCAGATGTGAGGGGAGAGATGGACAAGAATGGA
GTCTGTGTAGCATTTATAAAAATGATCAAAAATTATATTGTGTCATACCTCTGGATTCCAAAGCTGCAAGATTTCC
TCAGCAGAGAAACATCACCTGTAAATGTGGTTATCATTTATTATGATACTGACAGTCTAAATGATATAAACTATCA
GGTTGGGGTACATCTAATGACATGGAGAGTCTGGGTGACAAACACTCAGTGGCATGCTGAACTGTCTGGGAGAAAT
TTTATCCTTGATTCATTCCATGAGAGTCTCGTTTTTACACACCACTATGAGGAGATTTCTGGTTTTGATGAGTTTA

TCCAGACAGCTACCCCCTTCAAATACCCAGAAGACACTTTCCTCACTCTGTATGGGTTCCGGAATTTCCACTGCTC
TGGAATGGCTGCCTGCAAATCGTTTCGACACTGCCATGAGTGACAGGAGCTACAACGCATAC
>ht72_PS .
GGGAGTGGTCATTTCAGAGAGTGAGAAAGGTCTTCCATTTCTCTCAGACTTAAGGGAAGACATGGACAGGAGTAGA
ATCTGTGTAGCATTTGTAAAAATGATCTCAAACAGTGCCATGTGATATTTATGAAATCAACCAACATGTGATTTAT
GGACCAGAGAAACATCACCAGCAAGGGTTATTATCATCTACTTTGATACTAACAGAGTAAGTGATGTTAGATTTTA
CACTGGGTAATATTTAGTGACATGGAGAGTCTGGGTCACAAACTCACAATGGCATTTTGCCACCTTTGAGGAAAAT
TTCATAATTGACCCTTTCCATGGTACACTAATTTTCTCACACCAGCTTGAGGAGATTTCTGGTTTTAAAGCTTTCA
TCCAGACAGCTTCCCCTTCTAAATACCCAGAAGATATTTACCTGTCTATATTTTGGTCCATGAACTTTGACTGAAC
TTTGTCTAATTTTGATTGTACACTGAAGAAACGCCCACCCAATGCTTCTCTCTTGTATCTTCCTGTGAGTCGTTTT
GACCCTGCCATGAGCAATGAGAATTATGATATCTAC
>ht74_PS .
GGGATTGATCATCTCAGAAGACCAGGAAGGCATTCAGATTCTCTCAGATTTGAGAAGACAAATGGACATAACCAGA
GTGTGTAGAGCTTTTGTGGAAATGATTCCAAGTAACATAGTCCCCAGAGTCCTTATTAAAATTTCACTAGATCATA
AACTCCTCTACAAATGTAATTAGCATTTAAGGCGGTAGTGATTATCTGCAAATTTTGATGCATAAATTAGGTTAAA
TGTTAATAACATGGAAAGTCTGGATCATGAACTCACATTGGGATCTTTCCATCACTCCCAGATATTTTATGTTAGA
CTCATTTCATGGAACTCTCATTTTTTCACAACACCATGATGAAATTCAAAAATTTTATCCAGACAGCTAACCCTTC
TAAATACCCAGAAGACATTT
>ht1bis_PS .
GGGACTGGTCATCACAGAAGGCCAGCAAGGTCTTCAGTTTCTCTCAGATGTGAGAGCAGAGATGGTGGGGAACAGA
GTCTGTGCAGCACTGGTGAAAATGATCTCAAGTCCTCTCGTGACAATTCTCCCATTTCCACAGCGACATGATTTTC
TGACAAGGGAATTGTCAGCAGTAAATGTGGTCATTGTTCACTATGATATTGATAATCTAAACGATGTGAACTATAT
GATAGGTTAAAAGTTAGTGACACGGAGAGTCTGGGTGACAAACTCACAATGGAATGTCAACATGGCTGGGAGACAG
TTCATACTTGACTCTTCCAAAGGACTCTCATGTTTTCAAACTACCATGCAGAGCTTTCTGGTTTCAAAAACTTTAC
CCAGGCTGGGGATTTAGCTCAGTGGTTCCGGCTCCTGCCTTGCAAGCTCAAGGTCCCGAGTTCGATTCCCTGTACC
AAAAAAAACTTTACCCCAAATGCTGACCCTTTCAAATACCCAGAAGACAACTTTCTTGCAGTGTATTAGTGTATTG
GTTCAAGATTTCCATTGTTCATTACCTGATTCTG
>ht4bis_PS .
GGGACTGGTTATTGGAGAAGGTCAAAAAGGTCTTCAGTTTCTCTTAGACTTCAGAAGAGAGATGGACAGACAGACA
GTCTATGTTGCATTTATTAAAATGGTCACAAATGATTTTAGGACATGTTTGTCAAGTTCAGGGACTTATCATCTGT
GGAGCAGAGACACATCCCTAGGAAATGTTGTGATCATTAATTTTGATACAGATAGTACAATCAATATCAAGTTTCT
TGTATTGAAAAATTTAGTAACATGGACGATCTGGGTTACGAACTCACAGTGACAGGCTACAATCACCGTAAACAAT
TTCATACTTGACCTATTCCAAGGGACTTTCATTTCTACAAACCACCATGAGATTTCTTGTTTTAAAAATTTTGTAC
AGTCAACTAACCCCTCCAAATATCCAGAAGATACTCACCTCGCTATATACTTGATGAAGTATTTAGATTGTTCATT
CCCCAATTTCGACTGAGCACTGAAGAACTGTCCCCCCTCAGTGCCTCTCTGGCACAGTTGCCTGTGAATCTCATTG
ACACAAGGATGACAGATTGGAGTTACAATATATAT
>ht5bis_PS .
GGGACTGATTACTGCAGAAGGTCAAAAAGGTCTTCAGTTTCTCTCCAACTTGAGAAGAGAGATGAACAAGAAAAAA
GTCTGTGTAGCACTTATTAAAATGATATCAAAAGATATTAGGATATGTTTGTCAAGTACAGGGTCTTATCATCTAT
AGACCAGAGACACATCCCTAGGAAATGTTGTTATTTATTTTGTACAGATAGTACAATAGATATCAAGTCTCTTACA
TGGAAATATACCTTGGAGAATCTGGGTTACAAACTCAGAGTAACAGGCTACAATCAGTGGAAATAGTTTTATACTT
GACTCATTCCAAGAAACTCTCATTTTTACAAACCAACATGAGGAAATTTCTGGTTTTAAGAATTTT
>ht7bis_PS .
GGGACTGGTCATCACAGAAGGTCAGAAAGGTCTTCAATTCCTTTCAGATTTGAGAGGAGAGATGGACAAGAACAGA
GTCTGTGTAGCATTTGTGAAAGTGATCTCAAATGCTCCTGTTTCATATGTCTCAACTGCTAAGCCACGTGATTTCC
TAAGCAAAGAAATATTACCAGTAAATGCAGTTATCATTTACTTTGATTCTGTGAGTCTAAATGATATAAACTATCA
TGTAAGGCTATATTTATTGACATGCAGAGTGGATCACAAACTCAGAATGGCATGCTGATATGGCTGGGAGAAACTT
GACCTTTGATTCTTTCCATCCATGGGACTCTGATTTTTTCACAACACCATGAGATTTCTTGATTTAAAAATTTCAT
CCAAACTATTAACCCCTAAACATAACCAGAAGATACTTTCCTCACTCTGTATTGGTTCCAGAATTTCCATTGTTTG
TTTTCTGATTCTGTCTGTTCTTTCAAGGACTGTACACCTAATGCCTCTTTGGCATGGTTGCCTAT
>ht8bis_PS .
GGGACTGATTACTGCAGAAGGTCAAAAAGGTCTTCAGTTTCTTTCAGACTTGAGAAGAGAGATGAACAAGAAAAAA
GTCTGTGTAGCACTTATTAAAATGATATTAAAGATATTAGGATATGTTTGTCAAGTACAGGGTCTTATCATCTATA
GAGCAGAGACACATCCCTAGGAAATGTTATTATTTATTTTGTACAGACAGTACAATAGATATCAAGTCTCTTACAT
GGAAAAATACCTTGGAGAATCTGGGTTACAAACTCAGAGTAACAGGCTACAATCAGTGGAAACAGTTTTATACTTG
ACACATTCCAAGAAACTCTCATTTTTACAAACCAACATGAGGAAATTTCTGGTTTTAAGAATTTT
>scv1_PS .
AGGGCTGGTGATCTCAGAAGATGAGAAAGGCATTCGATTTCTCTCAGAGTTGAGAGGAGAAATGGACAGGAATGGA
GTCTGTGCAGCCTTTATTAATATGATCCCAGTCACTGTGCTTTATTTCAAAAGAGCTCACATACAATACAGCCAGA
TCTGAAATCATTAGCAGGGGTTGTGATGATTTTTGGAGACACAGAGTCTCTTCTGGCTGTGAGTTTTAAAAGAGGG
GACAATTTAGTCACTCGAAGAATTTGGGTCACCACCTCTCAGTGGGATGTTACCACCAGTAAGAGACATTTCATCC
CTGACTCATGCCATGGTGCTCTCCTCTTTTCACACTACCATGGGGAGATTTCAGGTTTTGAACTTTTTATCCAGAC

AGCTAATCCATCCAAATACCCAGAGGACACATACCTTGCGAGGTTGTGGTGGATGCATTTTAACTGCTCAGTCTCA
AAATCTGACTGTACAACATTGAGGAACTGTTCTTCCAAAGGCTCCTTGGCATGGTTACCATGGCACCACTTTGACA
TGGCCATGAGTGACGGGAGTTACAATATATAC
>scv2_PS .
TGGGCTGGTGATCTCAGAAGATGAGAAAGGCATTTGATTTCTCTCAGAGTTGAGAGGAGAAATGGACAGGAATGGA
GTCTGTGCAGCCTTTATTAATATGATCCCAGTCACTGTGCTTTAGTATTTCAAAAGAGCTCACATACAATACAGCC
AGATCTGAAATCATCAGCAAGGGTTGTGATCATTTTTGGAGACACAGAGTCTCTTCTGGCTGTGAGTTTTAAAAGA
TGGGACAATTTAGTCACACGAAGAATTTGGGTCACCACCTCTCAGTGGGATGTTACCACCAGTAAGAGACATTTCA
TCCTTGACCCATTCCATGGTGCTCTCCTCTTTTCACACTACCATGGGGAGATTTCTGGTTTTAAACATTTTATCCA
GACAGCTAATCCATCCAAATACCCAGAGGACACATACCTTGCGAGGTTGTGGTGGATGCATTTTAACTGCTCAGTC
TCAAAATCTGACTGTACAACATTGAGGAACTGTTCTTCCAAAGGCTCCTTGGCATGATTACCATGGCACCACTTTG
ACATGGCCATGAGTGACGGGAGTTACAATATATAC
>scv3 .
TGGGCTGGTGATCTCAGAAGATGAGAAAGGCATTCGATTTCTCTCAGAGTTGAGAGGAGAAATGGACAGGAATGGA
GTCTGTGCAGCCTTTATTAATATGATCCCAGTCACTGTGCTTTTGTATTTCAAAAGAGCTCACATACAATACAGCC
AGATCGTGAAATCATCAGCAAGGGTTGTGATCATTTTTGGAGACACAGAGTCTCTTCTAGCTGTGAGTTTTAAAAG
ATGGGACAATTTAGTCACACGAAGAATTTGGGTCACCACCTCTCAGTGGGATGTTACCACCAGTAAGAGACATTTC
ATCCTTGACCCATTCCATGGTGCTCTCCTCTTTTCACACTACCATGGGGAGATTTCTGGTTTTAAACATTTTGTCC
AGACAGCTAATCCATCCAAATACCCAGAGGACACATACCTTGCAAGGTTGTGGTGGATGCATTTTAACTGCTCAGT
CTCAAAATCTGACTGTACAACATTGAGGAACTGTTCTTCCAAAGGCTCCTTGGCATGGTTACCATGGCACCACTTT
GACATGGCCACGAGTGATGGGAGTTACAATATATAC
>scv5_PS .
GGGGCTGGTGATCTCAGAAGATGAGAAAGGCATTTGATTTCTCTCAGAGTTGAGAGGAGAAATGGACAGGAATGGA
GTCTGTGCAGCCTTTATTAATATGATCCCAGTCACTGTGCTTTAGTATTTCAAAAGAGCTCACATACAATACAGCC
AGATCTGAAATCATCAGCAAGGGTTGTGATCATTTTTGGAGACACAGAGTCTCTTCTAGCTGTGAGTTTTAAAAGA
TGGGACAATTTAGTCACACGAAGAATTTGGGTCACCACCTCTCAGTGGGATGTTACCACCAGTAAGAGACATTTCA
TCCTTGACCCATTCCATGGTGCTCTCCTCTTTTCACACTACCATGGGGAGATTTCTGGTTTTAAACATTTTATCCA
GACAGCTAATCCATCCAAATACCCAGAGGACACATACCTTGCAAGGTTGTGGTGGATGCATTTTAACTGCTCAGTC
TCAAAATCTGACTGTACAACATTGAGGAACTGTTCTTCCAAAGGCTCCTTGGCATGGTTACCATGGCACCACTTTG
ACATGGCCATGAGTGACGGGAGTTACAATATATAC
>scv7_PS .
GGGGCTGGTGATCTCAGAAGATGAGAAAGGCATTCGATTTCTCTCAGAGTTGAGAGGAGAAATGGACAGGAATGGA
GTCTGTGCAGCCTTTATTAAAATGATCCCAGTCACTGTGCTTTATTTCAAAGGAGCTCACATACAATACAGCCAGA
TCTGAAATCATTAGCAAGGGTTGTGATGATTTTTGGAGACACAGAGTCTCTTCTGGCTGTGAGTTTTAAAAGAGGG
GACAATTTAGTCACTCGAAGAATTTGGGTCACCACCTCTCAGTGGGATGTTACCACCAGTAAGAGCATTTCATCCT
TGACCCATTCCATGGTGCTCTCCTCTTTTCACACTACCATGGGGAGATTTCTGGTTTTAAACATTTTATCCAGACA
GCTAATCCATCCAAATACCCAGAGGACACATACCTTGCAAGGTTGTGGTGGATGCATTTTAACTGCTCAGTCTCAA
AATCTGACTGTACAACATTGAGGAACTGTTCTTCCAAAGGCTCCTTGGCATGGTTACCATGGCACCACTTTGACAT
GGCCATGAGTGACGGGAGTTACAATATATAC
>scv8_PS .
GGGGCTGGTGATCTCAGAAGATGAGAAAGGCATTTGATTTCTCTCAGAGTTGAGAGGAGAAATGGACAGGAATGGA
GTCTGTGCAGCCTTTATTAATATGATCCCAGTCACTGTGCTTTTGTATTTCAAAAGAGCTCACATACAATACAGCC
AGATCGTGAAATCATCAGCAAGGGTTGTGATCATTTTTGGAGACACAGAGTCTCTTCTAGCTGTGAGTTTTAAAAG
ATGGGACAATTTAGTCACACGAAGAATTTGGGTCACCACCTCTCAGTGGGATGTTACCACCAGTAAGAGACATTTC
ATCCTTGACCCATTCCATGGTGCTCTCCTCTTTTCACACTACCATGGGGAGATTTCTGGTTTTAAACATTTTATCC
AGACAGCTAATCCATCCAAATACCCAGAGGACACATACCTTGCGAGGTTGTGATGGATGCATTTTAACTGCTCAGT
CTCAAAGTCTGACTGTAAATCATTGAGTAACTGTTCTTCCAAAGGCTCCTTGGCATGGTTACCATGGCACCATTTT
GACATGGCCACGAGTGATGGGAGTTACAATATATAC
>scv9_PS .
GGGGCTGGTGATCTCAGAAGATGAGAAAGGCATTTGATTTCTCTCAGAGTTGAGAGGAGAAATGGACAGGAATGGA
GTCTGTGCAGCCTTTATTAATATGATCCCAGTCACTGTGCTTTAGTATTTCAAAAGAGCTCACATACAATACAGCC
AGATCTGAAATCATCAGCAAGGGTTGTGATCATTTTTGGAGACACAGAGTCTCTTCTGGCTGTGAGTTTTAAAAGA
TGGGACAATTTAGTCACACGAAGAATTTGGGTCACCACCTCTCAGTGGGATGTTACCACCAGTAAGAGACATTTCA
TCCTTGACCCATGCCATGGTGCTCTCCTCTTTTCACACTACCATGGGGAGATTTCTGGTTTTAAACTTTTTATCCA
GACAGCTAATCCATCCAAATACCCAGAGGACACATACCTTGCGAGGTTGTGATGGATGCATTTTAACTGCTCAGTC
TCAAAGTCTGACTGTAAATCATTGAGTAACTGTTCTTCCAAAGGCTCCTTGGCATGGTTACCATGGCACCATTTTG
ACATGGCCACGAGTGATGGGAGTTACAATATATAC
>sq1_PS .
GGGGCTGATCATTTCAGAAGATGAGAAAGGCATTCGATTTCTCTCAGATGTGAGAGGCGAGATGGACAGGAACGGA
GTCTGCGTGGCCTTTGTGAATATGATCCCAGTCACCATGCTGCTGTATTTCACAAGAGCTCACCAACATTACAACC
AGATCATGAAATCCTCGGCACGCATGGTTATCATTTTTGGTGACACAGAGTCTCTTCTGGCTGTGAGTTTTAATAG
ATGGGACAATGTAGTCACACAGAGAGTTTGGGTCACCACCTCACAATGGGATGTTACCAATAGTAAGAAACCTTTC

ATCCTGGACCCATTCCATGGCGCACTCATTTTTTCTCACCACCATGGGGAGATTTCTGGACTGAAACATTTTATCC
AGACAGCAAACCCTGCCAAATACCCAGAGGACACATACCTTGCTAGGTTGTGGTGGATGCATTTTAACTGCTCAGT
CTCAGAGTCTGAATGTAAAACACTGGAGAGCTGTTCTTCCACAGGCTCCTTGGCATGGTTACCACGGCACCATTTT
GACATGGCCGTGAGGGACGGGAGCTACAACATATAC
>sq2_PS .
GGGGCTGATCATCTCAGAGGATGAGAAAGGCATCCGTTTTCTCTCAGACTTGAGAGGCGAGATGGACAGGAACGGA
GTCTGCATGGCCTTTGTGAATATGATCCCATCCAGTGTGTCATTATACCTAAAAAGGCCTTACTTGTATTACAACA
GGTTAATGACTTCAACAGCAAATGTTGTGATCATTTTTGGTGATATGGACTCTTCCCTGAGCATGAGCTTTAGAAC
ATGGGACTATCTGGACACTTGGAAAATCTGGGTTACCACCTCCCAATTGGATGTAACGTCAAGTGAGAGCCATTTC
ATCCTGGACCCATTCCATGGCGCACTCATTTTTTCTCACCACCATGGGGAGATTTCTGGACTGAAACATTTTATCC
AGACAGCAAACCCTGCCAAATACCCAGAGGACACATACCTTGCTAGGTTGTGGTGGATGCATTTTAACTGCTCAGT
CTCAGAGTCTGAATGTAAAACACTGGAGAGCTGTTCTTCCACAGGCTCCTTGGCATGGTTACCACGGCACCATTTT
GACATGGCCGTGAGGGACGGGAGCTACAACATATAC
>sq3_PS .
GGCGCTCTTCCTGTCTGATGATTTGAAGGGAGAGCAGTTCCTGAGGGATCTGAAAGCAGAGATGGTAAAGAATGGT
ATCTGTGTGGCCTTAACAGAAAAGCTCCCTGTCACTAAGATAATGTATGGGACAGGGGACATCACCTTCCTGAGTA
GGATCAGAGTCTCCTCTGCAAACGTGCACATACTCCACGGTGAAGCAGGGAGCCTCGTCACCGTGGACATGGCCGC
AGACTTCTTTTTGACCACAGGGAAGGTGTGGATCATGGCAGCGAAGCAGGAGATCGTCCTGCACGAGATGAACCAC
ATGCTGCACTCTTTCCACGGGGGCTTCTCCTTCTCGCCTCACAGGGGGGAAATCCCTGGCTTCAGACACTTCCTCC
AGACAGTGACCCCTTCCCACTACCCGGAGGACTTTTACTTCTCTAAATTGTGGTTGCACCTCTTTCATTGTTCACC
GGCTGGGTCACTATGTGGACAAATTCATAATTGCCCTCCAAATGCATCCTTCGAGTTTGTGTCGGGAGTAATTGAC
ATGATGACCCTGTCTGACTCCAGCTACTTCATCTAT
>pr2nr .
GGATTGGCCATCTCTGACAGTGATCAGGGTACCCAATTTCTCTCATATTTGAGAACAGAGATTGAAAGAAATTCAG
TCTGCTTTGCTTTTGTGAGCATAATCCCAGTCAATATGAATTTATACATGTCAAGAACTGAAGTGTATTATAACCA
AATCATAACATCATCTGCAAAAGTAGTTATCATTTATGGTGACAAAGACAGTACTCTAGCTGTGAGCTTTAGAATG
TGGGAATCACTAGGTATACAGAGAATATGGGTGACTACCTCACAGCTGGATGTCACTACAAGTAAGAGAGTCTCCA
CAGTTGGCTCATCCCAAGGGACACTAGCTTTTGCACAGCATCATTCTGAGATTTCTGGTTTTAAAACTTTTGTCCA
GACATTGAACCCTCTGAAATACACAGATGAATACCTTGCAAGGCTGGGATGGATGAACTTTAACTGTAAAGTCTCA
GCTTCCAAGTGTAAGATACTGAAGAATTGTTCATTCAATGCCTCATTACAATGGCTAATGGGACAGACTTTTGACA
TGGCCTTTGGTGATGAAAGTTATGACATATAT
>pr4nr .
GGGCTGACCATCTCAGATGATGACCAGGGTCTCCAATTTCTTTCAGATCTGAGAGGAGAAAGTAGAGTCCAAAGAG
TCTGTTTAGCCTTTGTGATGGTGATCCCATTCAACATTGAGTTATACATGTCAAGAGCTAAAGTGTATAAAAACCA
AATTGAGACATCATCCACAAATGTTGTTATCATTTATGGTGATACAGACAGTACTCTAGCTGTGTGCTTTAGAATG
TGGATATCACAGGATATACAGAGAATATGGGTCACCACCTCACAGTGGGATTTCATTACAAGTAAGAGAAACTTCA
CCCTTGACTCATTCTATGGGACTCTTGCTTTTGCACACCACCATGCTGAGATTTCTGGTTTTAAAAATTTTGTTAA
GACAATGAACCCAGCCAAATACACAGACATGTTCCTGGCAGGACTGGGGTGGATGTACTTTAGCTGTGAAGCCTCA
GCATCTAACTGTAAGACATTGGAAAATTGTTCATCTAATGTCTCACTGGAATGGTTAATGCTACAGAGTTTTGACA
CGGCCTTTAGTGATGATACTTATGATATATAC
>pr7nr .
GGACTGGCCATCTCTGACAATGACCAGGGTACACAATTTCTCTCACATTTGAGAACTGAGATGGAGAAAAATATAG
TCTGCTTTGCCTTTGTAAGCATGATCCCAGCCAACATGCATTTATATATGTCAAGAGCTGAAGTGTATTATAACCA
AATCATAACATCATCTGCAAATGTAGTTATCATTTATGGTGACATAGACAGTACTCTAGCTGTGAGCTTTAAAATG
TGGGAATCTCTAGGTATACACAGAATATTTTTGACCACATCACAGTGGGATGTCACTACAAGTAAGAGAGACTCTA
CAATTGGCTCATCCCATGGGATACTAGCTTTTACACACCACCATTCTGAGATTTCTGGTTTCAAAACATTTGTCCA
GACATTGAACCCTCTGAAATACACAGATGAATACCTTTCAAAGCTGGGATGGATGAACTTTAACTGTGAGGTCTCA
GCTTCTAAGTGTAAGACACTGAAGAATTGTTCATCCAATGCCTCATTACAATTTCTAATGGGACAGACTATTGACA
TGGCCTTTAGTGATGACAGTTATGACATGTAT
>pr9nr .
GGGCTGGCCATCTCAGATGATGACCAGGGTCTCCAATTTCTCTCAGATCTGAGGGGAGAAACTGAAGTCCAAAGAG
TCTGCTTAGCCTTTGTGATCATGATCCCAGTCAACATGGAGTTATACATGTCAAGAGCTGAAATGTATAACAACCA
AATTGAGACATCATCCACAAATGTTGTTATCATTTATGGTGACACAGACAGTACTCTAGCTGTGTGCTTTAGAATG
TGGATATTACAGGGTATACAGAGAATATGGGTCACCACCTCACAGTGGGATGTCATTACAACCATGAGAGACTTCA
CCCTTGACTCATTCCATGGGACTCTTGCCTTTGCACACCAACATGCTGAGATTTCTGGTTTTAAAAATTATGTTCA
GACATTGAACCCTTCCAAACTCACAGACATGTTCCTGGCAGGACTGGGGTGGATGTACATTAGCGGTGAAGCCTCA
ACATCTAACTGTAAGACACTGGAGTACTGCTCATCTAATGTCTCACTGGAATGGTTAATGCTACAGAGTTTTGACA
TGGCCTTTAGTGATGATGCTTATGATATATAC
>pr12nr .
GGGATGATCATTTCAGATGATGACCATGGAATTCAATTTCTTTCTGAATGGAGAGGAGAGATGCAAAGAAACATTG
TCTGTTTAGCATTTGTGACTATGATCTCAACAGATTCAATGTTATACTTTAAAATGTTAAATAAGTATTATAACCA
GATCATGACATCATCAGCAAAAGTTGTGATCGTTAATGGAGACAAAGAATCTCATCTAAAGTGGAACTTTATCCTA

TGGCAATCTCTAGACATTCGGAGAATCTGGGTCAGTGTGTCACAATTTGATATGATCACAGTAAGAGGAGATTTCT
TGCTTAAATCCCCACATGGGACTCTCATTTTCTCACATCAGCATTCTGAGGTATCTGGTTTTAAACAATTTCTGCA
GACAGTGCACCCTTCAAACTACAGTAATGAAATTTCTCTTGCAAAACTGTGGTGGACTTATTTTAAGTGTTCTTTA
CCATCATCTAATTGTAACAAACTGAAGTATTGTTCAACAGAAACCCTACTGAAATGGTTATTTAGGACACCATCTG
GAATGGCCATGAGTGATACAACTTATAACTTATAT
>pr14nr .
GGACTAGTAATTCCAGATGATGACCCAGGCATTCAATTTCTCTCTGAATTGAGAGAAGAGATGCACAAACACAGAG
TCTGTTTGGCCTTTGTGAATGTGATCACAGAAAATAGAGACCAGAAAAGAACTGAAACATACTATAACCAGATCAT
GACATCATCAGCAAAAGTAATTATCATCTATGGGGACACAGACTCTGCTCTAACTCTACACTTTAGACTATGGCAA
CATTTAGGCACTCAGAGACTCTGGATCACCACCTCATTGTGGGATATGACCACAAGTAAAGGGGAGTTCCCCAAAA
AATACTTCCATGGGACTCTCATTTTATCACATCACCATTCTGAGATTTCTGGTTTTAAAAAATTCATTCAGACTGT
GCAACCTTCCAACTACAGTAGAAATATTTCCCTTGGTAGGTTGTGGTGGATGTATTTTAACTGTTCATTGCCATCT
CATTGTAACACATTGAAAAACTGTTCAACCAAAATCCTACTAGAATGGTTATCCAGGAACCAATTTGAAGTGTCCA
TGAGAGAGACAAGTTACAACCTATAC
>pr15nr .
GGAGTAATCATTTCAGATGAAGACCATGGACTTCAGTTTCTTTCTGAATTGAGAGAGGAGATGCAAAGGAACATTG
TCTGTTTAGCATTTGTGAGTATTTTCATCCATGGGAATTTGTCATTCCTTAAAATTGCTGAGAAGTACTTTAACCA
GATCATGATATCTTCAGCAAAGGTTGTGATTGTTTATGGAGATAAAGACTCTACTGTACAATTGAACTTTATACTA
TGGAAATCTGTAACATATAACAGAATCTGGGTCAGTATGTCACAATTTGATAGAATCACAATTAAAGGAGATTTCT
TGCTTAACTCACCCCATGGAACTCTCATTTATTCACATCAGAATTCTGAGATATCTGGTTTTAAACAATTTATGCA
GAGAGTGCACCCTTCAAATTACAGTAATGAAATTTCATTGCTTAGACTATGGTGGATTTATTTTAATTGTTCTTTG
TCATCATCTGATTGTAAGAAACTGAGAAATTGTTCTACCACAAATATATTAAACTGGTTGTTTAGGCACCAGTTTG
GAATGGCCATGAGTGACTCAAGTTATAACTTATAC
>pr16nr .
GGAGTGATCATTTCAGATGATGATTATGGAATTCAATTTCTTTCTGAACTGAGAGAGAAGATGCAAAAAAACATTG
TCTGTTTAGCATTTGTGAGTATTATTGCCCATCAGAATTTATCATTCCTCAAAATGGCTGATAAATACTTTAATCA
GATCATGATGTCATCAGCAAAAGTTGTGATTGTTTATGGAGACAAAGACTCTACTGTACAAGTGAACTTTATACTA
TGGAAATCTGTAACAGTTAACAGAATCTGGGTCAGCATGTCACAATTTGATAGAATCACAATTAAAGGAAATTTCT
TGCTTAACTCCAACTATGGGACTCTCATGTTTTCACATCAGAATTCTGAGATGCCTGATTTTAAACAATTTATGCA
GAGAGTGCACCCTTCAAATTACAGTAATGAAATTTCATTTGTTAGACTATGGTGGATTTATTTTAATTGTTCTTTG
TCATCATGTAGTTGTAAGAAACTGAAAAATTGTTCTACCACAAATATATTCAAGTGGTTGTTTAGGCACCGGTTTG
GAATGTCCATGAGTGACTCAAGTTATAACTTATAC
>pr17nr .
GGTGTAATCATTTCAGATGATGATCTAGGACTTCAATTTCTCTCAGATTTGAGAAGAGATATGCAAAAAAACAGTG
CATGCTTAGCATTTGTGCATATTATCATGGAAGATAAAATATTATTCCAGAAAAATATGAATGTCTATTATAATGA
GATCACAACATCATCAGCCAAAGTTGTTATCATTTATGGAGACAAAGACTCTCATCTGCAACTTGGCAGATTATGG
TGGCTTTATTTTAATTGTTCTCTGACTTCATTTAATTCTATGAATCTTAAGAATTGTTCAATAGAAAAACGATATC
GTTGGTTATTAAGGCACCAATTAGAAATATCTGTGAGTGGTACAGGTTATGTCCTATAC
>pr18nr .
GGAATGATCATTTCAGATGATGATGATGGAATTCAATTTCTTTCTGAATGGAGAAAAGAGGTGCAAAGAAACATTG
TCTGTTTAGCATTTGTGACTATAATCTCAACAGATACAATAATATACTTTAAAATGTTAAATAAGTATTATAACCA
AATCATGACATCATCAGCAAAAGCTGTGGTTGTTAATGGAAACAAAGAATCTCATCTAAAGTGGAACTTTATCCTA
TGGCAATCTCTAGACATTTGGAGAATCTGGGTCAGTGTGTCACAATTTGATATGATCACAGTAGGAGGAGATTTCT
TGCTTAAATCCCCTCATGGGACTCTCATTTTCTCACATCAGCATTCTGAGGTATCTGGTTTTAAACAATTTCTGCA
GAGAGTGCATCCTTCAAACTACAGTACTGAAATTTCTCTTGCAAGCCTGTGGTGGACTTATTTTAAGTGTTCTTTA
CCATCATCTAATTGTAACAAACTGAAGTATTGTTCAACAGAAACCCTACTGAAATGGTTATTTAGGACACCATCTG
GAATGGCCATGAGTGATACAACTTATAACTTATAT
>pr19nr .
GGAGTGATCATTTCAGATGAAGACCGTGGAATTCATTTTCTTTCTGAATTGAGAGAGGAGATGCAAAGGAACATTG
TCTGTTTAGCATTTGTGAGTATTATCATCCATGGGAATTTGTCATTCCTTAAAATGGCTGAGAAGTACTTTAACCA
GATCATGATGTCTTCAGCAAGAGTTGTGATTGTTTATGGAGACAAAGACTCTACTGTTCAATTGAACTTTATACTA
AGGAAATCTGTAACGTTTAACAGAATCTGGGTCAGTATGTCACAATTTGATAGAATCACAATTAAAGGAGAGTTCT
TGCTTAACTCACCCCATGGGACTCTCATTTATTCACATCATATTTCTGAGATATCTGGTTTTAAACAATTTATTCA
GAGAGTGCACCCTTCAAATTACAGTAATGAAATTTCATTGGTTAGACTATGGTGGATTTATTTTAATTGTTCTTTG
TCATCATCTAATTGTAAGAAACTGAAAAATTGTTCTACCACAAATATATTCAAGTGGTTGTTTAGGCACCAGTTTG
GAATGTCCATGAGTGACTCAAGTTATAACTTATAC
>pr21nr .
GGAGCAATCATTTCAGATGATGACCTAGGACTTGAGTTTCTCCTGGAATTGAGAAAAGAGATGCAAAGAAATAGTG
TGTGCTTAGCCTTTGTGCATATTATTGTGGAGGACAAAATATTATTCCAGAAAAATGCAAACATGTATTATAATGA
GATCACAATGTCATCAGCAAAAGTTGTTATCATTTATGGAGACAAAGACTCTTATCTACAATTCAATCTTAGACTT
TACAAATTAGCATACATTCAGAGAATATGGGTTACTACCCGACAGTGGGACTGGATCACACAAAATGACAGATTCC
TTATTGATTCCTTCTATGGGACCTTTACTTTTTTAAATCACCATCATGAATTATCTGGTTTTAATACATTTATCAA

AACAGCAGTCCCTTTCAAATACAATGGTATTCACCTTTCTAAACTATCTTGGCTATATTTTAATTGTTCTTTGTCA
TCATTTGGTTGTAAGAATCTGAAGAATTGTTCAATGCGAACACGATTGCATTGGTTATTCAAACATTATTTTGAAA
TTTCTGTGAGTGGTACAGGTTATGTCCTTTAC
>pr22nr.
 .
GGAGCAATCATTTCAGATGATGACCTAGGACTTGAATTTCTCTCAGAATTGAGAAGAGAGATGCAAAGAAAAAGTG
CATGCTTAGCCTTTGTGCATATTATTATGGAAGATAAAATATTATTCCAGAAAAATGTAAATATTTATTATAATGA
GATCATAACCTCATCAGCCAAAGTTGTTATCATTTATGGAGACAAAGACTCTCATCTGCAACTTAACCTTAGATTC
TACAGACTAGCATACCTTCAGAGGATCTGGGTCACTACTTCACAGTGGGATATGATCGTACATAATGAGAGACTCC
TTCTTGATTCCTTCTATGGGACTTTTATATTTTTACTTCACTCTTCAGAATTATCTGGTTTTAAAAAATTTATTGA
GACAGTAGATCCTTCCAAGTATAGTAAATCAATTTCTCTTGCTGAATTATGGTGGATTTATTTTAATTGTTCTCTG
ACTTCATTTAATTCTATGAACCTTAAGAATTGCTCAATAGAAAAACGAATGCAGTGGTTATTCCAGCACCATTTTG
AAACATCTGTTAGTGGTACAGGTTATGTTGTATAT
>pr24nr .
GGAGCAATCATTTCAGATGATGACCTAGGACTTCAATTTCTCTCAGAATTGAGAAGAGAGATGCAAAGAAACAGTG
TGTGCTTAGCGTTTGTGCATATTATCATGGAAGATAAAATATTATTCCAGAAAAATGTAAATATCTATTATAATGA
GATCACAACGTCATCAGCCAAAGTTGTTATCATTTATGGAGACAAAGACTCTCATCTGCAACTTAACCTTAGACTC
TACAGATTATCAAACGTTCAGAGAATCTGGGTCACTACTTCACAGTGGGATATGATCACACATAATGACAAATTAC
TTCTTGATTCTTTCTACGGGATGTTTACTTTTTTACTCCACTTTTCTGAATTATCTGGTTTTAGAACATTTATTGA
GACAGTAGTTCCTTCCAAGTATAGTACGCCAATTTCCCTTGGTAAATTATGGTGGATTTATTTTAATTGTTCTCTG
ACTTCATTTAATTCTATGAATCTTAAGAATTGCTCAATAGAAAAACGGTATCATTGGTTATTCCAGCACCATTTTG
AAATGTCTGTGAGTGGTACAGGTTATGTCCTATAC
>pr25nr .
GGAGCAATCATTTCAGATGATGATCTAGGACTTGAATTTCTCTCAGAATTGAGAAGAGAGATGCAAAGAAACAGTG
TGTGCTTAGGCTTTGTGCATATTATTATGGAAGATAAAATATTATTCCAGAAAAATGTAAATATTTATTATAATGA
GATTACCACGTCATCAGCTAAAGTTGTTATCATTTATGGAGACAAAGATTCTCATCTGCAACTTAACCTTAAATTC
TATAGACTAGTATACCTTCAGAGGATCTGGGTCACTACTTCGCAGTGGGATATGATCACACATAATGACAGATTCC
TTCTTGATTCCTTCTATGGGACCTTTACATTTTTAATTCACTCTTCAGAATTACCTGGTTTTAAAAAATTTATTGA
GACAGCAGATCCTTCCAAGTATAGTAATAAATATTCTCTGGCTAAAATGTGGTGGATTTATTTTAATTGTTCTCTG
ACTTCATTTAATTCTATGAACCTTAAGAATTGCTCAATAGAGAAACGAATGAAGTGGTTATTCCAGCACCGTTTTG
AAACGTCTGTGAGTGGTACAGGTTATGTTGTATAC
>pr26nr .
GGTCTGATTCTCCCAGATGACCATAAAGGGGTTCATTTTGTGTCAGAATTGAGAAGAGAATTGGAGAGTAATAGAG
TCTGCATAGCTTTTGTGGAAATGATTCTAGGCACCTGGAGTTCATTTTCCAATAAATTCTGGAAAAATCTGGGGAA
GATCCAAAATTCATCAGCAAATGTGATAATCATTTATGGAGATACTTATTCTCTACAAGGTTTAATGAGAAACATT
GGGCAACTGTTAATGACAGGGAAAGTCTGGATCTTGAATGCTCAATGGGATTATACCAACCATGCTGATTATTTTC
TTTTGGACTCATTCCATGGCAGTCTTATTTTTTCACACCACCTTGAGGAGAGTTCTGAGTTTACAAATTTTATCAG
AACAGTTAATCCTTACAAATACCCAGAAGACAATTACCTCCCTAAGCTATGGTTTTTGTTCTTCAAATGCTCATTT
TCTGAGATTGATTGCCATCTTTTGGAGAACTGCCATCCCAATGCTTCCTTGGAATTATTGCCTAGACACATTTTTG
ACATTGTAATGAGTGAAGAGAGCTACAATATATAC
>pr30nr .
GGTCTTATTGTCCCAGATGACCACAGAGGAACTCAATTTCTATCAGATATCAGAGAAAATATGGAGAAAAATCAAA
TCTGCATAGCTTTTGTTGAAATGATCCAAAGTACCTGGAATTCATTTCCATACAAATACTGGAAAAGTTTTGTCAC
TGTCCAGGAATCATTGGCCAACATCATCATCATTTATGGTGACAATGATTCACTACAAGGTTTAATGAGAAATCTG
GGGCAACAGATTTTGACAAGTAAAGTCTGGGTCATGACCTCTCAGTGGGATGTTACTAATATGGCTGATTATTTCT
TGCTAGATTCTTTCCATGGAAGTCTTATTTTTTCACACCACCATGAGCAGATTATTGAATTTAAAAATTTTATCCA
AACAGTTAATCCTTATAAATACCCAGAAGACAATTACCTTCCTAAATTGTGGTTTTTGTTCTTCAAATGCCCATTT
TCAGAGCTGGATTGTCATCTTTTGGAGAACTGCCAACCCAATGCTTCCTTGGAATTACTGCCTAGACACATTTTTG
ACATGGCAATGAATGAAGACAGCTACAATACATAC
>pr31nr .
GGGCTGCTTATCACAGATGATCACAGAGGGACTCATTTTCTAGCAGATATCAGAAAAAAGATGGAGAAAAATCAAG
TCTGCATAGCTTTTGTTGAAATGATCCAAAGCACCTGGAATTCATTTTCATATAAGTACTGGAAAAGTCTTGGCAT
TATCCAGGAAACATTGGCCAATATTATCATCATTTATGGTGACAATGATTCACTACAAGGTTTAGTGAGCAATCTG
GGGCAACAGATTTTGACCAGTAAAGTCTGGGTCATGACCTCTGAATGGGATGTTACTAATATTGCTGATTATTTCT
TACTGGATTCATTCCATGGAAGTCTCATTTTTTCACAACACCATGAGCAGGTGTCTGAATTTAAAAATTTTATCCA
AACAGTTAATCCTTATAAATACCCAGAAGACAATTACCTTCCTAAATTGTGGTTTTTGTTCTTCAAGTGCTCATTT
TCAGAGCTTGATTGTCATCTTTTGGAGAACTGCCAACCCAATGCTTCCTTGGAATTACTGCCTAGACATATTTTTG
ACATGGTAATGAATGAAGACAGCTACAATACATAC
>pr35nr .
GGGCTGCTTATCCCAGATGATCACAGAGGGACTCAGTTTCTAGCAGATATCAGAGAAAAGATGGAGAAAAATCAAG
TCTGCATAGCTTTTGTTGAAATGATCCAAAGCACCTGGAATTCATTTTCATACAAATACTGGAAAAGCCTTGGGAT
CATCCAGGAAACATTGGCCAATATTATCATCATTTATGGTGACAATGATTCACTACAGGGTTTAATACGAAATCTA

GGGCAACAGATTTTGACAAGTAAAGTCTGGGTCATGACCTCTGAGTGGGATGTTACTAATATTGCTGATTATTTCT
TGCTGGATTCATTCCATGGAAGTCTCATTTTTTCACAACACCATGAGCAGGTGGCTGAATTTAAAAATTTTATCCA
AACAGTTAATCCTTATAAATACCCAGAAGACAATTACCTTCCTAAATTGTGGTTTTTGTTCTTCAAGTGCTCATTT
TCAGAGCTTGATTGTCATCTTTTGGAGAACTGCCAACCCAATGTTTCCTTGGAATTACTGCCTAGACATATTTTTG
ACACGGCAATGAATGAAGACAGCTACAATACATAC
>pr36nr .
GGTCTTGTTGTCCCAGATGACCACAGAGGAACTCAGTTTCTAGCAGATATAAGAGGAAATATGGAGAAAAATCAAG
TCTGCATAGCTTTTGTTGAAATGATCCAAAGCACCTTTAATTCATTTTCTTACAAATACTGGAAAAGTCTTGGCAT
GGTCCAGGAATCATTGGCCAATATCATCATCATTTATGGTGACACTGATTCACTACAAGGTTTAATGAGACATCTA
GGGCAACAGATTTTGACAACTAAAGTCTGGGTCATGACCTCCCAGTGGGATGTTACTAATATGGCTGATTATTTCT
TGTTAGATTCATTCCATGGAAGTCTCATTTTTTCACAACACCATGAGCAGGTGGCTGAATTTAAAAATTTTATCCA
AACAGTTAATCCTTATAAATACCCAGAAGACAATTACCTTCCTAAATTGTGGTTTTTGTTCTTCAAGTGCTCATTT
TCAGAGCTTGATTGTCATCTTTTGGAGAACTGCCAACCCAATGCTTCCTTGGAATTACTGCCTAGACATATTTTTG
ACACGGCAATGAATGAAGACAGCTACAACACATAC
>pr38nr .
GGGCTGCTTATCACAGATGATCACAGAGGGACTCATTTTCTAGCAGATGTCAGAGAAAAGATGGAGAAAAATCAAG
TCTGCATAGCTTTTGTTGAAATGATCCAAAGCACCTTTAATTCATTTTCATACAAGTACTGGAAAAGTCTTAGCAT
TATCCAGGAAACATTGGCCAATATTATCATCATTTATGGTGACAATGATTCACTACAAGGTTTAATGAGCAATCTA
GGGCAACAGATTTTGACAAGTAAAGTCTGGGTTATGACATCTGAGTGGGATGTTACTAATAATGCTGATTATTTCT
TACTGGATTCATTCCATGGAAGTCTCATTTTTTCACAACACCATGAACAGGGGACTGAATTTATAAATTTTCTCCA
AACAGTTAATCCTTATAAATACCCAGAAGACAATTACCTTCCTAAATTGTGGTTTTTGTTCTTCAAGTGCTCATTT
TCAGAGCTTGATTGTCATCTTTTGGAGAACTGCCAACCCAATGCTTCCTTGGAATTCCTGCCTAGACATATTTTTG
ACATGGTAATGAATGAAGACAGCTACAGTACATAC
>pr40nr .
GGATTGCTCCTCACAGATGACCACAGAGGAATTCAAATTTTATTTGACTTGAAAGAAGAGATGAGGAAAAATATAG
TCTGCATGGCTTTTGTGGAAATGATTTCAGGTACATTGGCTTCATTTTCTGACAATTTCTGGAAAAGTGTGGGAAG
GATCCAAGACTCATCTGCAAATGTGATTATCATTTATGGAGACACTGATTCTCTACATGGCTTAGTGAAAAATATA
GGACAGAAGTTAACTACAGGGAAAGTCTGGGTCATGAACTCACCTTGGGATGTTACCAACCACTTTGATTATTTTA
TGTTAGATTCTTTCCATGGGAGTCTCATTTTTTCACACCACTATGAGGAGATGGTTGATTTTACACATTTTATCCA
AACAGTTCGTCCTTCCAAATACCCAGAAGATACATATCTTCCTAAATTGTGGCATTTGTTCTTCAAATGCCCATTC
TCCAGGATTGATTGTAAACTCTTGGAGAACTGTCAACCCAATGCTTCCTTGGATTTATTGCCTAAGCAAAATTTTG
ACACGGCCATGAGTGAAGAGAGTTACTACATATAC
>pr41nr .
GGATTGCTCCTCAGAGATGACCACAGAGGAAGTCAAATTTTATCTCATTTGAAAGACGAGATGAAGAAAAATGGAG
TCTGTATGGCTTTTGTGGAAATGATTTCAGGTACATTGGCTTCATTTTCTGACAGTTTCTGGAAAAGTGTGGGAAG
GATTCAAGACTCATCTGCAAATGTGATTATCATTTATGGAGACACTGATTCTCTACAAGGCTTAATGAAAAATATA
GGGCAGAAGCTAACTACAGGGAAAGTCTGGGTCATGAACTCACTTTGGGATGTTACCAACCACTTGGATTATTTTA
TGTTAGATTCTTTCCATGGGAGTCTCATTTTTTCACACCACTATGAGGAGATGGTTGATTTTACACATTTTATCCA
AAAAGTTCATCCTTCCAAATACCCAGAAGATACATATCTTCCAAAATTGTGGCATTTATTCTTCAAATGTCCATTC
TCTAGAACTGATTGTAAACTCTTGGAGAACTGTCAGCCCAATGCTTCCTTGGATTTATTGCCTAAGCAAAATTTTG
ACACGGCCATGAGTGAAGAGAGTTACTACATATAT
>pr42nr.
.
GGGCTGTTCATCATAGATGATCACAAGGGTGCCCAGATTCTGTCAGATTTGAGAAGTGAGCTGGAGAAAAATGGAG
TCTGTATAGCATTTGTGGAAATGATCCTAGCCATCAGGGGTTCATTTCTGACCACTTCCTGGAAAAATCAGGTGCA
GATTCTGGATTCATCAGCAAATGTGATTATTATTTATGGAGATACTGATTCTCTCTTAAGCTTAATAGTAAATATA
AAGCAGAAGTTAGTCACATGGAAAGTCTGGGTCCTGAACTCACACTGGGATCACTCCAAATTTGATAACTATTTCC
TGTTAGATGCATTGCATGGAACCCTAATTTTTTCACACCATAATGAGGAGATTAATAATTTTACAAATTTTATTCA
GACAGCAAATCCTTCCAAATACCCAGAAGACATTTATCTTCATGTGTTGTGGCACTCATTCTTTAATTGCTCATTT
TTGTGGAAAAACTGTAAAATCATCGATAACTGTCTGCCTAATGCCTCCTTGGGATTCTTGCCAGGGAACATGTTTG
ACATGGCCATGAGTGAAGAGAGGTACAATGTGTAC
>pr46nr .
GGGCTGTTCATCATAGATGATCACAAGGGTGCCCAGATTCTGTCAGATTTGAGAAGTGAACTGGAGAAAAATGGAG
TCTGTATAGCATTTGTGGAAATGATCCTAGCTATCAGGGGTTCATTTCTCTCCACATCTTGGAAAAATCAGGTGAA
AATCCTGGAATCATCAGCAAATGTGATTATTATTTATGGGGACACTGATTCTCTATTGAGTTTAATATCAAATATA
AAGCAGAAGTTAGTCACATGGAAAGTCTGGGTCCTGAACTCACACTGGGATCATTCCACATTTGATAACTATTTCC
TGTTAGATGCATTACATGGGACCCTTATTTTTTCACACCATAAGGAGGAGATTATTAATTTTACGGATTTTGTTCA
GACAGCCAATCCTTACAAATACCCAGAAGACATTTATCTTCATGTATTGTGGCACTCATTCTTCAATTGCTCATTT
TTGAGGAAAGACTGTAAAATTGTGGGTAACTGTCTGCCTAATGCATCCTTGGGATTCTTGCCAGGGAACATATTTG
ACATGGCCATGAGTGAAGAGAGTTACAATGTGTAC
>pr47nr .

```
GGGCTGTTCATCATAGATGATGACAAAGGTGCCCAGATTCTGTCAGATTTGAGAAGTGAGATGGAGAAAAATGGAG
TCTGCATAGCATTTGTGGAAATGATCCTAGCTATCAGGGGTTCGTTTATGACCACATCCTGGAAAAATCAGGTGCA
GATCCTGGAATCATCAGCAAATGTGATTATTATTTATGGGGACACTGATTCCTTATTAAGTTTAATAGTAAATATT
AAGCAGAAATTACTTACATGGAAAGTCTGGGTCCTGAGCTCATACTGGGATCATTCCAAATTTGATAACTATTTGC
TGTTAGATTCATTGCATGGGGCTCTTATTTTTTCACACCATAAGGAGGACATCTATAATTTTACAGATTTCATTCA
GACAGCCAATCCTTTCAAATACCCACAAGACATTATTCTTCATGTATTGTGGCACTCATTCTTCAATTGCTCATTT
TTGAGAAAAGATTGTAAAATTGTGGATAACTGTCTGCCTAATGCCTCCTTGGGATTCTTGCCAGGGAACATATTTG
ACATGACCATGAGTGAAGAGAGTTACAATGTGTAC
>pr48nr.
 .
GGGCTGTTCATCATAGATGATGACAAAGGTGCCCAGATTCTGTCAGATTTGAGAAGTGAGATGGAGAAAAATGGAG
TCTGCATAGCATTTGTGGAAATGATCTTAACTGTCAGGGGTTCATTCATGACAACATCCTGGAAAAATCAGGTGCA
GATCCTGGAATCATCAGCAAATGTGATTATTATTTATGGGGACACTGATTCCTTATTAAGCTTAATAGTAAATATT
AAAGAGAAGTTACTCACATGGAAAGTCTGGGTCATGAACTCACACTGGGATAATTCCAAATTTGATAACTATTTCC
TGTTAGATTCATTGCATGGAGCCCTTATTTTTTCACACCACAAGGAGGAGATCATTAATTTTACAGATTTTATTCA
GCTAGCCAATCCTTTCAAATACCCACAAGACATTATTCTTCATGTATTGTGGCACTCATTCTTCAATTGCTCATTT
TTGAGAAAAGATTGTAAAATTGTGGATAACTGTCTGCCTAATGCCTCCTTGGGATTTTTGCCAGGGAACATGTTTG
ACATGACCATGAGTGAAGAGAGTTACAATGTGTAC
>pr52nr .
GGACTGGTTCTCATGGATGACCACACAGGGGCTGAGAGTCTATCAGACTTGAGAAGAGAAATGGACAGGTACAGAG
TGTGTTTAGCTTTTGTAGAAATGATTCCAGACATCCCAATTATCTCCTACTATGAACCTTCCATAAAAACTCACCT
GCAGATACTGAAATCATCTGCAAATGTGGTTATCCTTTATGGTGACCATGAATCATTACATGGTGCAATTATGATT
ATAGCAGCACATGTTTTGAACAGAAAAGTCTGGGTCTTGAAGTCACAATGGGATGAAACTACTCTTACTAAATCTT
TCATATTTGATGCATTTCACGGGAGTCTCATTTTTGCACATCACCACACTGAGGTTTCTGATTTTAGGAAGTTTAT
CCAGACATACAATCCTTCCAAATACCCAGAGGACTATTTTCTTGCTCAGTTCTGGAACACATACTTCAACTGCTCT
TTTTCTGGACCAGATTGTAAAATTTTGGGTAACTGTCTACCCAATGCTTCTTTGGATATGTTGCCAAAAAATGTTT
GGGAAATGGATATGACTGAAGAGAGTTACAATTTATAT
>pr53nr .
GGACTGGTTCTCATAGATGACCACAATGCAGCTGAGATTCTATCCAACTTGAGAGGAGAGATGGATAGAAACAGAG
TGTGTGTAGCTTTTGTAGAAATGATTCCATTCAACTGGATGAACACTGTTTTTAAATCCAGAACAATGCATCCACA
GATCCTGAAATCATCTGCAAATGTGGTTATCATTTACGATGTCACTAAATCTTTATATGCTCTAATAATATATATA
AGTATACATTCAAGTGCTTGGAAAGTCTGGATCATTAAGTCACAATGGGATGTAACTACTGATGTTCCTTATTTCA
TATTTGATCCATTCCATGGTAGTCTCATTTTTGCACAACACCATGCTGAGATTTCTGATTTTAGGAAGTTTATCCA
GACATACAACCCTTCCAAATATCCAGGAGATCATATCCTTGCTCTTCTTTGGAACACATATTTCAATTGCTCTTTT
TCTGGACCTGATTGTAAAAGTTTGGTTAACTGTCTACCCAATGCTTCTTTGGAAATGTTGCCTGGAAATGCTTTGG
AAATGGACATGAGTGAAGAGAGTTACAATGTATAT
>pr55nr .
GGGCTCATCGTGTCCGATGATCTGAAAGGAAAGGAGTTCCTCTCTGACCTGAAAGCAGAGATGGTATCAGAAGATA
TCTGTGTGGCTTTTACAGAAAAACTCCAGGCTAATTGGAAGTATCAGTTAACATCTGATGTTGGGTTGGTGAATTT
GATCCAACATACACAAGCAAATGTGCATGTATTCTATGGTGATATAGATGACTTAGTATTTTTCTTCATGAAAACT
AAAATCATGAAAGCCATAAGGAAGGTGTGGATCATGGCAAAGTCCCACTTAGTTTACTTAGAGTCTGTATTCTATG
AAAAAAATTATTTGAAGGACTTTTTTGAAGGTAGCTTCTCATTTTCAAAGAAGAGAGATATCCCTGGCTTCAAGCC
CTTTCTAGAGGCACTTACACCCTCCCAGTACCCAGGTGAGCTTTACTTTAATAAATTCTGGATTGACAATTTTTAT
TGCTCACCTCCTCCTCTGCTATGTGGAAATCATAAACCCTGCCCAGTGAATACATCCCTAAAGACAAAGCAAGAAA
TAAATGTGATGATCACTTCTGAGCCCAGTTTTTCCATATGG
>pr65nr .
GGCCTTGTGGTCCCAGATGATATGAGGGGAGAACGATTCCTTAGGGACATCACAGAAGAGATGACCATCCATGGAC
TTTGTGTTGCATTTGCAGAAAAAATTCCAGAATTTCCTGCTAAGGACACAACAAACAGGCTACGGTTCACAGAAAG
ATTCACATTGACAAATGTCGTTGTTGTATTTGGAGACACATATTCTCTTCTGAGGCTTGTCTATAACATCTTTTGC
AATATTCCTCTTGGTAATGTCTGGGTTACAACTTTGGACTGGGATATCACCACATTACCATTTGAACAAAGTCTAA
GTTATACATACTTCGGTGGAGGATTATCATTTTCTGTTTACATGGATGAAATTCTGGGATTCAAGGATTTTCTCAG
GAGTGTTCAACCTGGGAAATACCCTCGTGATATCTTTATCCAGGATGTGTGGTCAGTATTATTTGAATGTCCATAT
TCAGATCGGGACTTTATCAGGGAATTGAGTCAGTGTGAGCAAAATGGCACCCTGGGCACACGAGCTCTGCATGCTT
GGGATATGAATACCTCCCCCCCAAGCTACAGAGTCCAT
>pr13 .
GGGCTCATAGTGTCTGATGATCTGAAAGGAAAGGAGTTCCTCTCTGACCTGAAAGCAGAGATGGTATCAGAAGATA
TCTGTGTGGCTTTTACTGAAAAACTCCAGGTTAATTGGAAGTTTCAGTTAAGATCTAATGTTGGGTTGGTGAATTT
GATCCAACATACACAAGCAAATGTGCATGTATTCTATGGTGATATAGATGACTTAGTATTTTTCTGCATGGAAAAT
GAAATCATGAAAGCCAAAAGGAAGATGTGGATCATGGCAAAGTCCCACTTAGTTTACTTAGAGTCTGTATTCTATG
AAAACAATTATTTGAAGGACTTTTTTGAAGGAAGCTTCTCATTTTCAAAGAAGAGAGATATCCCTGGCTTCAAGCC
CTTTCTAGAGGCACTTACACCCTCCAGGTACCCAGGAGAGCTTTACTTCCATAAATTCTGGATTGACAATTTTTAT
```

```
TGCTCACCTCCTCCTCTGCTATGTGGAAATCATAAACCCTGCCCACTGAATACATCCCTAAAGACAAAACAAGAAA
TAAATGTGATGATCACCTCTGAGCCCAGTTTTTCCATATGG
>pr14 .
GGGCTCATCGTGTCTGATGATCTGAAAGGAAAGGAGTTCCTCTCTGACCTGAAAGCAGAGATGGTATCAGAAGACA
TCTGTGTGGCTTTTACAGAAAAACTCCAGTCTAATTGGAAGTTTCAGATCATATCTGATGTTGGGTTGGTGAATTT
GATCAAACATACACAAGCAAATGTGCATGTATTCTATGGTGATATAGATGCCTTAGTATTTTTCTGCATGCAAAAT
GAAATCATGAAATCCATAAGGAAGGTGTGGATCATGGCAAAGTCCCACTTAGTTTACTTAGAGTCTGTATTCTATG
AAAACAATTATTTGAAGGACTTTTTTGAAGGAAGCTTCTCATTTTCAAAGAAGAGAGATATCCCTGGCTTCAAGCC
CTTTCTAGAGGCACTTACACCCTCCCAGTACCCAGGTGAGCTTTACTTTAATAAATTCTGGATTGACAATTTTTAT
TGCTCACCTCCTCCTCTGCTATGTGTACATCATAAACCCTGCCCACTGAATACATCCCTAAAGAGAAAGCAAGAAA
TAAATGTGATGACCACTTCTGAGCCTAGCGTTTCCATATGG
>pr15 .
GGGCTCATCGTGTCTGATGATCTGAAAGGAAAGGAGTTCCTCTCTGACCTGAAAGCAGAGATGGTATCAGAAGATA
TCTGTGTGGCTTTTACTGAAAAACTCCAGGCTAATTGGAAATTTCAGTTAACATATGATGTTGGGTTGGTAAATTT
GATCCAACATACACAAGCAAATGTGCATGTATTCTATGGTGATATAGATGACTTAGTATTTTTCTTCATGAAAACT
AAAATGAAAGCCAGATGGATGGTGTGGATCTTGGCAAAGTCCCACTTAGTTTACTTAGAGTCTGTATTCAATGAGC
AAAATGATTTGATGAACATTTTTGAAGGAAGCTTCTCATTTTCAAAGAAGAGAGATATCCCTGGCTTCAACCACTT
TCTAGAAGCACTTACACCCTCCCATTACCCAGGTGAGCTTTACTTTAATAAATTCTGGATTGACAATTTTTATTGC
TCACCTCCGCCTCTGCTATGTGGAAATCATAAACCCTGCCCACTGAATATATCCCTAAAGACAAAGCAAGAAAAAA
ATATGATGGTCACGTCTGAGCCCAGTTTTTCCATATGG
>pr16 .
GGGCTCATCGTGTCTGATGATCTGAAAGGAAAGGAGTTCCTCTCTGACCTGAAAGCAGAGATGGTATCAGAAGATA
TCTGTGTGGCTTTTACAGAAAAACTCCAGGCTAATTGGAAGTTTCAGTTAACATATGATGTTGGGTTGGTGAATTT
GATCCAAAATACACAAGCAAATGTGCATGTATTCTATGGTGATATAGATGACTTAGTATTTTTCTGTGCACAAAAT
GAAATCATGAAAGCCGGAAGGAAGGTGTGGATCATGGCAAAGTCCCACTTAGTTTACTTAGAGTCTGTATTCTATG
AAAATAATTATTTTAAGGACTTTTTTGAAGGAAGCTTCTCATTTTCAAAGAAGAGAGATATCCCTGGCTTCAAGCC
CTTTCTAGAGGCACTTACACCCTCCCGGTACCCAGGAGATCTTTATTTCTATAAATTCTGGATTGACAATTTTTAT
TGCTCACCTCCTCCTCTGCTTTGTGAAAATAAAAAACCCTGCCCACTGAATACATCCCTAAAGACAAAGCAAGAAA
TAAATGTGATGATCACTTCTGAGCCTAGCTTTTCCATATGG
>pr17 .
GGGCTCATCGTGTCTGATGATCTGAAAGGAAAGGAGTTCCTCTCTGACCTGAAAGCAGAGATGGTATCTGAAGATA
TCTGTGTGGCTTTTACAGAAAAACTCCAGGCTAATGGGAATGTTCAGTTAACATCTGAGTTTGGGAAGGTGAATTT
GATTCAACATACAAAAGCAAATGTGCATGTATTCTATGGTGATACAGATGACTTAGTATTTTTCTGCGTGCAAAAT
GAAATCATGAAAGCCAGAAGGAAGGTGTGGATCATGGCAAAGTCCCACTTAGTTTACTTAGTGTCTGTATTCAAGA
ATCAAAATGATTTGATGGACTTTTTTGAAGGAAGCTTCTCATTTTCAAAGAAGAAAGAGATCCCTGGCTTCAAGCC
CTTTCTAGAGGCACTTACACCCTCCTGGTACCCAGGAGATCCTTATTTCTATAAATTCTGGATTGACAATTTTTAT
TGCTCACCCCCTCCTCTGCTATGTGAAAATAAAAAACCCTGCCCACTGAATACATCCCTAAAGACAAAGCAAGAAA
TAAATGTGATGATCACTTCTGAGCCTAGCTTTTCCATATGG
>pr18 .
GGGCTCATCGTGTCTGATGATCTGAAAGGAAAGGAGTTCCTCTCTGACCTGAAAGCAGAGATGGTATCTGAAGATA
TCTGTGTGGCTTTTACAGAAAAACTCCAGGCTAATGGGAATTTTCAGTTAACATCTGAGTTTGGGAAGGTGAATTT
GATTCAACATACAAAAGCAAATGTGCATGTATTCTATGGTGATACAGATGACTTAGTATTTTTCTGCGCGCAAAAT
GAAATCATGAAAGCCAGAAGGAAGGTGTGGATCATGGCAAAGTCCCACTTAGTTTACTTAGAGTCTGTATTCAAGA
ATCAAAATGATTTGATGGACTTTTTTGAAGGAAGCTTCTCATTTTCAAAGAAGAAAGACATCCCTGGCTTCAAGCC
CTTTCTAGAGGCACTTACACCCTCCTGGTACCCAGGAGATCCTTATTTCTATAAATTCTGGATTGACAATTTTTAT
TGCTCACCCCCTCCTCTGCTATGTGAAAATAAAAATTCCTGCCCACTGAATACATCCCTAAAGACAAAGCAAGAAA
AAAATATGATGGTCATATCTGAGCCCAGTTTTTCCATATGG
>pr19 .
GGGCTCATCGTGTCTGATGATCTGAAAGGAAAGGAGTTCCTCTCTGACCTGAAAGCAGAGATGGTATCAAAAGATA
TCTGTGTGGCTTATACAGAAAAATTCCAGGCTAAATGGCATTATCAGTTAACATTTGAGGTTGGGTTGATGAACTT
GATCCAACATACACAAGCAAATGTGCATGTATTCTATGGTGATACAGATGACTTAGGACTTTTCTTCATGAAAATT
AAATTCATGAAAGCCAGAAGGAAGGTGTGGATCATGGCAAAGTCCCACTTTGTTTACTTAGAGTCTGCATTCAATA
AGCTAAATGATTTGATGGACCTTTTAGAAGGAAGCTTTTCATTTTCAAAGAAGAGAGATATCCCTGGCTTCAACCA
CTTTCTAGAAGGTCTTACACCCTCCCAGTACCCAGGTGAACTTTACTTTAATAAATTCTGGATTGATACTTTTTAT
TGCTCACCTCCTCCTGTGCTATGTAAAATTCA
>pr22 .
GGTCTGCTTATCACAGATGATCGCAGAGGGACTCAGTTTCTAGCAGATATCAGAAAAAATATGGAGAAAAATCAAG
TCTGCATAGCTTTTGTTGAAATGATCCAAAGCACCTGGAATTCATTTTCATACAAATACTGGAAAAGTCTTGGCAT
CATCCAGGAAACATTGGCCAATATTATCATCATTTATGGTGACAATGATTCACTGCAAGGTTTAATGAGCAATCTA
GGGCAACAGATTTTGACCAGTAAAGTATGGGTCATGACCTCTGAGTGTGATGTTACTAATAAGGCTGACTATTTCT
TACTGGATTCATTCCATGGAAGTCTCATTTTTTCACAACACCATGAGCAGGGGACTGAATTTAAAAAAATTACCCA
AAGAGTTAATCCTTATACATACCCAGAGGACAATTACCTTCCTAAATTGTGGTTTTTGTTCTTCAAGTGCTCATTT
```

TCAGAGCTTGATTGTCATCTTTTGGAGAACTGCCAACCCAATGCTTCCTTGGAATTACTGCCTAGACATATGTTTG
AAATGATAATGAATGAAGACAGCTACAATACATAC
>pr27 .
GGTTTACTTATTTCAGATGACCACAGAGGGATTCAGTTTCTTTCAGATATCAGAGAGAAGATGGAGAAAAATCAAG
TCTGCATAGCTTTTATTGAAATGATCCACAGCACCTGGAATTCATTTTCATACAAATACTGGAAAAGCCTTGGGAT
CATCCAGGAAACATTGGCCAATATTATCATCATTTATGGTGACAATGATTCACTGCAAGGTTTAATGAGCAATATA
GGGCAACAGATTTTGACAAGTAAAGTCTGGGTCATGACCTCTGAGTGGGATGTTACTAATAATGCTGATTATTTCT
TGCTAGATTCATTCCATGGAAGTCTCATTTTTTCACAACACCATGAGCAGGTGGCTGAATTTAAAAATTTTCTCCA
GACAGTTAATCCTTATAAATACCCAGAAGACAATTACCTCCCTAAATTGTGGTTTTTGTTCTTCAAGTGCTCATTT
TCAGAGCTTGATTGTCATCTTTTGGAGAACTGCCAACCCAATGCTTCCTTGGAATTACTGCCTAGACATATTTTTG
ACATGGTAATGAATGAAGATAGCTACAATACATAC
>pr28 .
GGTTTACTTATTCCAGGTGACCACAGAGGGATTCAGTTTCTTTCAGATATCAGAGAAAAGATGGAGAAAAATCAAG
TCTGTATAGCTTTTGTTGAAATGATTCAAAGCACCTGGAATTCATTTTCTTACAAATACTGGAAAAGTCTTGGCAT
CATCCAGGAAACATTGGCCAATATTATCATCATTTATGGTGACAATGATTCACTACAAGGTTTAATGAGCAATCTA
GGGCAACAGATTTTGACCAGTAAAGTCTGGGTCATGACCTCTGATTGGGATGTTACTAATATTGGTGATTATTTCT
TGCTAGATTCATTCCATGGAAGTCTCATTTTTTCACAACACCATGAGCAGGTGTCTGAATTTAAAAATTTTATCCA
AACAGTTAATCCTTATAAATACCCAGAAGACAATTACCTTCCTAAATTGTGGTTTTTGTTCTTCAAGTGCTCATTT
TCAGAGCATGATTGTCATTTTTTGGAGAACTGCCAACCCAATGCTTCCTTGGAATTACTGCCTAGACATATTTTTG
ACACGGCAATGAATGAAGACAGCTACAATACATAC
>pr32 .
GGTCTGCTTATCCCAGATGATCACAGAGGAACTCAGTTTCTATCAGATATCAGAGAAAACATGGAGAAAAATCAAG
TCTGCATAGCTTTTGTTGAAATGATGCAAAGTTCCTGGACTTCATTTCCTGAAAAATACTGGAAAATTATTGGCAT
CATCCAGGAATCATTGGCCAATATCATCATCATTTACGGTGACAATGATTCACTACAAGGTTTAATGAGAAATCTA
GGGCAACAGATTTTGACAAGGAAAGTGTGGGTCATGACCTCTCAATGGGATGTTACTAATATCGCTGATTATTTCT
TGCTAGATTCCTTCCATGGAAGTCTCATTTTTTCACAACACCATGAGCAGGTCATTGAATTTAAAAATTTTATCCA
AACAGTAAATCCTTATAAATACCCAGAAGACAATTACCTTCCTAAATTGTGGTTTTTGTTCTTTGAGTGCTCATTT
TCAGAGCTTGATTGTCATCTTTTGGAGAACTGCCAAGCCAATGCTTCCTTGGAATTCCTACCTAGACACATTTTTG
ATATGGCAATGAACGAAGACAGCTACAACACATAC
>pr33_PS .
GGTTTACTTATTCCAGATGATCACAGAGGGACTCAGTTTCTATCAGATATCAGAGAAAAGATGGAGAATAATCAAG
TCTGCATAGCTTTTGTTAAAATGATCCAAAGTACCTGGAATTCATTTTCATACAAATACTGGAAAAGTCTTGGCAT
GGTCCAGGAATCATCTGCCAATATCATCATCATTTATGGTGACAATGATTCACTACAAGGTTTAATGCGAAATCTA
GGGCAACAGATTTTGACAATGAAAGTTTGGCTCATGACCTCTGAATGGGACCTTACTAATATCGCTGATTTTTTCT
TGCTAGATTGATTCCATGGAAGTCTCATTTTTTCACAACACCATGAGCAGGTCATTGAATTTAAAAAATTTATACA
AACAGTTAATCCTTATAAATACCCAGAAGACAATTACCTTCCTAAATTGTGGATGATGTTCTTCAAGTGCTCATTT
TCTGAGCTTGATTGTCATCTTTTGGAGAACTGCCGACCCAATGCTTCCTTGGAATTCCTGCCTAGATACATTTTTG
ATTTGGCAATGAATGAAGACAGCTACAATACATAC
>pr34 .
GGTCTGCTTATCCCAGATGACCACAGAGGGACTCAGTTTCTAGCAGATATCAGAGAAAAGATGGAGACTAATCAAA
TCTGCTTGGCTTTTGTCGAAATGATCCAAAGTACCTGGAATTCATTTCCATACAAATACTGGAAAAGTCTTGGCAT
GGTCCAGGAATCATTGGCCAATATCATCATCATTTATGGTGACAACGATTCATTACAAGGTTTAATGCGAAATCTA
GGGCAACAGATTTTGACAGTGAAAGTCTGGGTCATGACCTCTGAATGGGATGTTAGTAATATCATTGATTTTTTCT
TGCTAGATTCATTCCATGGAAGTCTCATTTTTTCACAGCACCATGAACAGGTCATTGAATTTAAAAATTTTATCCA
AACAGTTAATCCTTATAAATACCCAAAAGACAATTACCTTCCTAAATTGTGGAATTTGTTCTTCAAGTGCTCATTT
TCAGAGCTGGATTGTCATCTTTTGGAGAACTGCCAACCCAATGCTTCCTTGGAATTACTACCTAGACACATTTTTG
ACATGGCAATGAATGAAGACAGCTACAATACATAC
>pr35 .
GGTTTACTTATTCCAGATGACCACAGAGGGACTCAGTTTCTTTCAGATATCAGAGAAAAGATGGAGAATAATCAAG
TCTGCGTAGCTTTTGTTGAAATGATTCAAAGCTCCTGGAATTCATTTTCATACAAATACTGGAAAAGTCTTGGCAT
GGTCCAGGAATCATCTGCCAATATCATCATCATTTATGGTGACAATGATTCACTACAAGGTTTAATGCGAAATCTA
GGGCAACAGATTTTGACAATGAAAGTTTGGCTCATGACCTCTGAATGGGACCTTACTAATATTGCTGATTTTTTCT
TGCTAGATTCATTCCATGGAAGTCTCATTTTTTCACAACACCATGAGCAGGTCATTGAATTTAAAAATTTTATACA
AACAGTTAATCCTTATAAATACCCAGAAGACAATTACCTTCCTAAATTGTGGATTATGTTCTTCAAGTGCTCATTT
TCAGAGCTGGATTGTCATCTTTTAGAGAACTGCCAACCCAATGCCTCCTTGGAATTCCTGCCTAGATACATTTTTG
ACACTGCAATGAATGAAGACAGCTACAACACATAC
>pr36 .
GGTCTAATTGTCCCAGATGACCACAGAGGAACTCAGTTTTTATCAAATATAAGAGAAAATATGGAGAATAATCAAG
TCTGCATAGATTTTGCTGAAATGATCCAAAGCACCTGGAATTCATTTCCATACAAATACTGGAAAAGTTTTGTCAC
GGTCCAGGAAACATTGGCCAATATTATCATCATTTATGGTGACAATGATTCACTGCAAGGTTTAATGAGAAACCTA
GGGCAACAGATTTTGACAACGAAAGTCTGGGTCGTGACCTCTCAGTGGAATGTTACTAATATAGCTGATTATTTCT
TGCTAGATTCTTTCCATGGAAGTCCCATATTTTCACACCACCATGAGCAGATTATTGAATTTAAAAATTTTATCCA

AACAGTTAATCCTTATAAATACCCAGAAGACAATTTCCTTCCTAAATTGTGTTTTTTGTTCTTCAAGTGCTCATTT
TCAGAGCTTGATTGTCAATTTCTGGAGAACTGCCAACCCAATGCTTCCCTGGAATTACTGCCTAGACACATTTTTG
ATATAACAATGAATGAAGACAGCTACAATACATAC
>pr37_PS .
GGTCTGCTTATCCCAGATGACCACAGAGGGACTCAGTTTCTAGCAGATATCAGAGAAAAGATGGAGACTAATCAAG
TATGCATAGCTTTTGTTGAAATGATCCAAAGTACCTGGAATTCATTTTCATACCAATACTGGAAAAGTCTTGGCAT
GGTCCAGGAATCATCAGCCAATATCATCATCATTTATGGTGACACTGACTCACTACAAGGTTTAATACGAAATCTA
GGGCAACAGATATTGACAAGGAAAGTTTGGGTCATGACCTCTGAGTGGGATGTTACAAATATTGCTGATTTTTTCT
TGCTAGATTCTTTCCACGGAAGTCTCATTTTTTCACGACAGCATGAGCAGGTCAATGAATTTAAAAATTTTATCCA
AACAGTTAATCCTTATAAATACCTAGACGACAATTACCTCCCTAAATTGTGGTTTTTGTTCTTCAAGTGCCCATTT
TCAGAGTTTGATTGCCAACTTTTGGAGAACTGCCAATCCAATGCTTCCTTGGAATTCCTGCCTAGACACATTTTTG
ACACGGCAATGAACGAAGACAGCTACAATACATAC
>pr38 .
GGTCTTATTGTCCCACATGACCACAGGGGAACTCAATTTCTATCAGATATCAGAGAAAATATGGAGAAAAATCAAG
TCTGCATAGCTTTTGTTGAAATGATCCAAAGTACCTGGAATTCGTTTCCGTACAAATACTGGAAAAGTTTTATCAC
AGTCCAGGAATCATTGGCCAACATCATCATCATTTATGGTGACAATGATTCACTACAAGGTTTAATGAGAAATCTG
GGGCAACAGATTTTGACAAGTAAAGTCTGGGTCATGACCTCTCAGTGGGATGTTACTAATATGGCTGATTATTTCT
TGCTAGATTCTTTCCATGGAAGTCTCATTTTTTCACACCACCATGAGCAGATTATTGAATTTAAAAATTTTATCCA
AACAGTTAATCCTTATAAATACCCAGAAGACAATTACCTTCCTAAATTGTGGTTTTTGTTCTTCAA
>pr41 .
GGTTTACTACTCCCAGATGACCACAGAGGGACAGAGATGCTATCAGATTTAAGAGAAAAGATGGAGAGTAGTAGAA
TATGCATAGCTTTTGCAGAAATGATCCCAGGCACCTGGACTTCATTTTCCAATAAATTTTGGAAAAGTCTAGGAAA
GATCCAGGAATCACTGGCCAATGTCATCATTATTTATGGCGATATTGATTCTCTACAAGGTTTAATGCGAAATCTA
GGTCAACAGTTTTTGACACAGAAAGTCTGGGTTACAAACTCTCAATGGGATGTTACCAATCACGCTGATTATTTCA
TGATAGACACTTTCCATGGCAGTCTCATTTTTGAACACCATCATGAGGAGATGGTTGAGTTTACCAATTTTGTTAG
AACCTTTAATCCATATAAATACCCAGAAGACAATTACCTTCCTAAATTATGGTTTTTGTTCTTCAAATGCCCATTT
TCTGAGCTTGATTGTCAACTTGTAGAGACCTGCCAACCCAACGCTTCATTGGAATTCTTGCCTAGGTACATTTTTA
ACATGGCCATGAGTGAAGAGAGTTACAACATATAC
>pr42_PS .
GGTGTACTACTCCCAGATGACCACAGAGGGACACAGATGCTATCAGATTTAAGTGAAAAAATGGAAAGTAGTAGCA
TATGCATAGCTTTTGTAGAAATGATCCCAGGCACCTGGACTTCATTTTCTAACAAATTTTGGAAAAATCTGGGAAA
GATCCAGGCATCACTAGCCAATGTCATCATTATTTATGGTGACATTGGTTCTCTACAAGGTTTAATGCGAAATCTA
GGGCAAGAGCTTCTGACACAGAAAGTCTGGGTTTAAACTCTCAATAGGATGTTACCAACCATGCTGATTATTTCA
TGATAGACTCTTTCCATGGGAGTCTCATTTTTGAGCACCACCATGAGGAAATGGTTGAGTTTACCAATTTTATTAG
AACAGTTAATCCATATAAATACCCAGAAGACAATTACCTTCCAAAATTATGGTTTTTGTTCTTCAAATGCCCATTT
TCTGAGCTTGATTGTCAACTTTTAGAGAACTGCCAACCCAATGCTTCATTGGAATTCTTGCCTAGGCACATTTTTA
ACATGGCCATGAGTGAAAACAGTTACAACATATAC
>pr43 .
GGTCTGATTCTCCCAGATGACCATAAAGGGATTCATATTGTGTCAGAATTGAGAAGAGAATTGGAGAGTAATAGAG
TCTGCATAGCTTTTGTGGAAATGATTCCAGGCACCTGGACTTCATTTTCCAATAAATTCTGGAAAAATCTGGGGAA
GATCCAAAATTCATCAGCAAATGTGATAATCATTTATGGAGATACTTATTCTCTACAAGGTTTAATGAGAAACATT
GGGCAACTGTTAATGACAGGGAAAGTGTGGGTCTTGAATGCTCAGTGGGATTATACCAACCATGCTGATTATTTCC
TTTTAGATTCATTCCATGGCAGTCTTGTTTTTTCACATCACCTTGAGGAGAGTTCTGAGTTTACAAATTTTATCAG
AACAGTTAATCCTTACAAATACCCAGAAGACAATTACCTTCCTAAGCTATGGTTTTTGTTCTTTAAATGTGCATTT
TCTGAGCTTGATTGTCATCTTTTGGAGAACTGCCATCCCAATGCTTCCTTGGAATTATTGCCTAGACACATTTTTG
ACATTGTAATGAGTGAAGAGAGCTACAATATATAC
>pr44 .
GGTTTACTACTCCCAGATGACCACAGAGGGACAGAGATGCTATCAGATTTAAGAGAAAAGATGGAGAGTAGTAGAA
TATGCATAGCTTTTGCACAAATGATCCCAGGCACGTGGACTTCATTTTCCAACAAATTTTGGAAAAGTCTAGGAAA
GGTCCAGGAATCACCGGCCAATGTCATCATTATTTATGGTGATATTGATTCTCTACAAGGTTTAATGAGAAATCTA
GGTCAACAGTTTTTGACACAGAAAGTCTGGGTTACAAACTCTCAATGGGATGTTACCAATCACGGTGATTATTTCA
TGATAGACTCTTTCCATGGCAGTCTCATTTTTGAACACCACCATGAGGAAATGGTTGAGTTTACCAATTTTGTTAG
AACCCTTAATCCATATAAATACCCAGAAGACAGTTACCTTCCTAAATTATGGTTTTTGTTCTTCAAATGCCCATTT
TCTGAGCTTGATTGTCAACTTGTAGAGACCTGCCAACCCAACGCTTCATTGGAATTCTTGCCTAGGCACATTTTTA
ACATGGCCATGAGTGAAGAGAGTTACAACATATAC
>pr50_PS .
GGGCTGTTGATCATAAATGATCACAAAGGTTCCCAGATCCTGTCAGATTTGAGAATAAAGATGGAGAAAAATGGAG
TCTCCATAGCATTTGTGGAAATGATCCTAGTCATCAGGTGTTCATTTCTGGCCACTTCCTGGAAAAATCAGGTGAA
AATCCTGGAATCATCAGCAAATGTGATTATTATTTATGGGGACACTGATTATCTATTAAGCTTAATAGTAAATATT
AAGCAGATGTTACCCACATGGAAAGTCTGCGTCCTGAACTCACAATGGGATCATTCCACATTTGATAACTATTTCC
TGTTAGATGCATTGCATGGGACCCTTATTTTTTCACAACATAATGAGGAGATCAATAATTTTACAGATTTTATCCA
GACAGCCAATCCTTCCAAATACCCAGAAGACATTTATCTTCCCATATTATGGCACTCATTCTTCAACTGTTCATTT

TTGAGGAAAAACTGTAAAATTGTGGATAACTGTCTGTCTAACACCTCCTTGAGATTCTTACCAGGGAACATGTTTG
ACATTGCCATGAATGAAGAGAGTTATAATGTGTAC
>pr52 .
GGTCTGTTCATCATAGATGATCACAAGGGTGCCCAGATTCTGTCAGATTTGAGAAGCGAGCTGGAGAAAAATGGAG
TCTGTATAGCATTTGTGGAAATGATCCTAGCCATCAGGGGTTCATTTTTGACCACATCTTGGAAAAATCAGGTGAA
AATCCTGGAATCATCAGCAAATGTGATTATTATTTATGGGGACACTGATTCTCTATTGAGTTTAATATTAAATATA
AAGCAGAAGTTAGTCACATGGAAAGTCTGGGTCCTGAACTCACACTGGGATCATTCCACATTTGATAACTATTTCC
TGTTAGATGCATTACATGGGACCCTTATTTTTTCACACCATAAGGAGGAGATTATTAATTTTACGGATTTTGTTCA
GACAGCCAATCCTTACAAATACCCAGAAGACATTTATCTTCATGTATTGTGGCACTCATTCTTCAATTGCTCATTT
TTGAGGAAAGACTGTAAAATTGTGGGTAACTGTCTGCCTAATGCATCCTTGGGATTCTTGCCAGGGAACATATTTG
ACATGGCCATGAGTGAAGAGAGTTACAATGTGTAC
>pr55 .
GGGCTGTTCATCATAGATGATCACAAAGGTGCCCAGATTCTGTCAGATTTGAGAAATGAGATGGAGAAAAATGGAG
TCTGCATAGCATTTGTGGAAATGATCCTAGTCATCAGGGGTTCATCTCTGACCACACCCTTAAAAAATCAGGTGCA
AATCCTGGAATCATCAGCAAATGTGATTATTATTTATGGGGACACTGATTCTCTATTAAGCTTAATAGTAAATATT
AAGCAGATGTTACTCACTTGGAAAGTCTGGGTCCTCACCTCACACTGGGATGATTCCAAATTTTTTAACTATTTTC
TGTTGGATTCATTGCATGGGGCCCTTGTTTTTTCACACCATAAGGAGGACATGATCAATTTTACAGATTTTATTCA
GACAGCCAGTCCTTTCAAATACCCACAAGACACTTATCTTCATGTATTGTGGCACTTAATCTTTAATTGCTCATTT
TTGAGGAAAGACTGTAAAATTGTGGATAACTGTCTGCCTAATGCCTCCTTGGAATTGTTGCCACATAACATATTTG
ACATGGCCATGAGTGAAGAGAGTTATAATGTGTAC
>pr56_PS .
GGGCTGTTCATCATAGATGATCACAAAGGTTCCCAGATCCTGTCAGATTTGAGAATCAAGATGGAGAAAAATGGAG
TCTGCATAGCATTTGTGGAAATGATCCTAGTCATCAGGTGTTCATTTCTGGCCACTTCCTGGAAAAATCAGGTGAA
AATCCTGGAATCATTACCAAATGTGATTATTATTTTTGGTGACACTGATTCTCTATTAAGCTTAATAGTAAATATT
AAGCAGATGTTACCCACATGGAAAGTCTGGGTCCTGAACTCACAATGGGATCATTCCACATTTGATAACTATTTCC
TATTAGATGCATTGCATGGGACCCTTACTTTTTCACAACATAATGAGGAGATCAATAATTTTACAGATTTTATCCA
GTCAATCCTTCCAAATACCCAAAAGACATTTATCTTCCCGTATTATGGCACTCATTCTTCAACTGTTCTCTTTTGA
GGAAAAACTGTAAAATTGTGGATAACTGTCTGTCTAACACCTCCTTGAGATTCTTACCAGGGAACATGTTTGACAT
TGTCATGAATGAAGAGAGTTACAATGTGTAC
>pr61 .
GGGCTGTTCATCATAGATGATGACAAGGGTGCCCATATTCTGTCAGATTTGAGAAGTGAGCTGGAGAAATATGGAG
TCTGTATAGCATTTGTGGAAATGATCCAAGTAATCAGGGGTTCATTTCTGACTACTTCCTGGAAAAATCAGGTGCA
GATCATGGATTCATCAGCAAATGTGATTATTATTTATGGGGACACTGATTCTCTCTTAAGCTTAATAGTAAATATA
AAGCAGAAATTAGTCACATGGAAAGTCTGGGTCCTGAGCTCACAATGGGATCATTCAGAAATTGATGACTATTTCC
TGTTAGATGCACTGCATGGGACCCTTATTTTTTCACACCATAATGAGGAGATTAAAAATTTTACAGATTTTATCCA
GACAGCCAATCCTTCTAAATATCCAGAAGATGTTTATCTTCATGTATTATGGCACTCATTCTTCAATTGCTCTTTT
TTGAGGAAAGACTGTAAAATTGTGGATAACTGTTTGCCTAATGCCTCCTTGCGATTTTTGCCAGGGAACATATTTG
ATATAGCCATGAGTGAAGAAAGTTACAATGTGTAC
>pr66 .
GGACTGGTTATTATGGATGACCACACAGGGGCTGAGAGTCTATCAGACTTGAGAGAAGAGATGGACAGGAACAGAG
TGTGTTTAGCTTTTGTAGAAATGATTCCAGACATCCCGATTGTCTCCTACTATGACCCTTCCATAAAAACTCACCT
GCGTATTGAGAAATCACCTGCAAATGTGGTTATCATTTATGGTGACCATGAATCCTTATATGGTGCAATTATGATT
ATAGCAGCACATTTATTGAACTGGAAAGTCTGGGTCCTGAAGTCACAATGGGATGAAAGTTCTCTTATTAAATCTT
TGATAATTGATACATTCCATGGGAGTCTCATTTTTGCACATCACCACACTGAGGTTTCTGATTTTAGGAAATTTAT
CCAGACATATAACCCTTCCAAATACCCAGAGGACTATTTTCTTGCTCTGTTCTGGAACTCATACTTCAAGTGCTCT
TTTCTGGACCTGATTGTAAAATTTTGCCTAACTGTCTACCCAATGCTTCTTTGGAAATGTTGCCTAAAAATGTTT
GGGAAATGGATATGACTGAAGAGAGTTACAATTTATAT
>pr67 .
GGACTGGTTCTCGTAGATGACCACAATGGGGCTGAGATTCTCTCAGACCTGAGAGGAGAGATGGACAGGAACAGAG
TGTGCATAGCTTTTGTAGAAATGATTCAGGACAACTTGATTTCCTCCAACGACTTTTCCAGAACAACTTACCTGAA
GATCCTGAAATCATCTGCAAATGTGGTTTTCATTTATGGTGACACAGAATCTTCACATGGTATAATCATAAATACA
GGGATACGTTTAATGAATGGGAAAGTTTGGATCATGAGGTCACAATGGGATTCAACTACTGATTTTGAGTATTTCA
TATTTGATTCATTCCATGGGAGTCTCATTTTTGCACACCACCGTGCTGAGATTTCTGATTTTAGGAAGTTTGTCCA
GACATACAACCCTTCCAAATACCCAGAAGATGGTTATCTTGCTGTTTTGTGGAACACATACTTCAATTGTCCCTTT
TCTGGACCTGATTGTAAAATTTTGAGTAGCTGTCTACCCAACGTTTCCTTGGAAATGTTGCCTAAAAATGTTTGGG
AAATGGATATGACTGAAGAGAGTTACAATATATAT
>pr68 .
GGACTGGTTCTCATGGATGACCACACAGGGGCTGACAGTCTATCAGACTTGAGAAGAGAGATGGACAGGTACAGAG
TGTGTTTAGCTTTTGTAGAAATGATTCCAGAAATCCCAGTTTCCTCCTACTCCCAACCTTCCATAAAAACTCACAT
GCAGATACTGAAATCATCTGCAAATGTGGTTATCATTTATGGTGACCATGAACCATTACATGGTGCAATTATGATT
ATAGAAGCACATGTTTTGAACATGAAAGTGTGGGTATTGAAGTCACAATGGGATGAAACTTATCTTACTAAATCTT
TGATATTTGATACATTTCACGGGAGTCTCATATTTGCACATCACCACACTGAGGTTTCTGATTTTAGGAAGTTTAT

CCAGACATACAATCCTTCCAAATACCCAGAGGACTATTTTCTTGCTAAGTTCTGGAATGCATACTTCAACTGCTCT
TTTTCTGGACCTGATTGTAAAATTTTGGGTAACTGTCTACCCAATGCTTCTTTGGATATGTTGCCAAAAAATGTTT
GGGAAATGGATATGACTGAAGAGAGTTACAATTTATAT
>pr70_PS .
GGACTGGTTCTCTTGGATGACCATAAAGGGACTGAGAGTCTATCAGACTTGAAAAGAGAGATGGACAGGTACAGAG
TGTGTTTAGCTTTTGTAGAATTGATTCCAGACATCCCAGTTTCCTCCTACTATGAACCTTCAATAAAAACTCACCT
GCAGATACAGAAGTCATCTGCAAATGTGGTTATCATTTATGGTGACCATGAATCATTACATGGTGCAATTATGGTT
ATAGCAGCACATGTATTGAACAGGAAAGTCTGGGTCTTGAAGTCACAGTGGGATGAAAATTCTCTTACTAAATCTC
TTGTATTTGATACATTCCATGGGAGTCTCATTTTTGCACATCACCACAATGAGGTTTCTGATTTTAGGAAGTTTAT
CCAGACATACAATCCTTCCAAATACCCAGAGGACTATTTTCTTGCTAAGTTCTGGAACACATACTTCAACTGCTCT
TTTTCTGGACCTGATTGTAAAATTTTGGATAATTGTTTACCCAATGCATCTTTGGATATGTTGCCTAAAAATGATT
GGGAGATGGATATGACTGAAGAGAGTTACAATTTATAT
>pr72_PS .
GGATTGGTTCTCACAGATGACCACAATGGGGCTGAGATTCTATCAAACTTGAGAAGAGAGATGGACAGGAACAGAG
TGTGTGTAGCTTTTGTAGAAATGATTCCGGACAGCCAAGTTTACTCGGGCTATGAATCCAGAACAACTCATCGAAA
CATCTTGAAATCATCTACAAATGTTGTTTTCCTTTATGATGTCACTCAATATTTATATGATCTAATAATATATATA
GGAATACATTCAATTACTTGGAAAGTCTGGATCACTAAGTCACAATGGGATGTAACTACTGATGTTCCTTATTTTG
TATCTGATCCATTCCATGGTAGTCTCATTTTTGCACAACACCATGCTGATATTTCTGATTTTAGGAAGTTTATCCA
GACATACAAGCCTTCCAAATATCCAGAAGACTATATCCTTGCTCTTTTGTGGAACAGATATTTCAATTGCTCTTTT
TCTGGACCCAGTTGTAAAATATTAGGTAACTGTCAACCCAATGCTTCCTTGGAAATGTTGCCTCGAAATGCTTTGG
AAATGGATATGACTGAAGAGAGTTATAATGTATAT
>pr73_PS .
GGACTGGTTCTCATAGATGACCACAATGGGGCTGAGATTCTATCACACGTGAGAGGAGAGATGGATAGGAACAGAG
TGTGTATAGCTTTTGTAGAAATGATTTCTAACAACTGGATTGACTCTGGCTACAAATCCAGAACAAGTCATCGGCA
GATCTGAAATCATCTGCAAATGTGGTTTTCATTTATGATGTCACTATATCTTTATATGGTATAATACTATACATAA
GTATACATTCAATTACTTGGAAAGTCTGGATCATTAAGTCACAGTGGGCTGTATCTACTGATGTTCCTTATTTTGT
ATTTGATCCATTCCATGGGAGTCTCATTTTTGCACCACACCATTCTGAAATTTCTGATTTTAGGAAGTTTATCCAG
ACATACAAGCCTTCCAAATACTCAGAAGCCCATATCCTTGCTCTTCTGTGGAACAGATATTTCAATTGCTCTTTTT
CTGGACCTAACTGTGAAATTTTGGTTAACTGTCTACCCAATGCTTCCTTGGAAATGTTGCCTGGAAATGCTTTGGA
AATTCACATGACTGAAGACAGTTACAACATATAT
>pr74_PS .
GGACTGCTCATCACAAGTGACCGCAGAAGTGCTCAGATTGTATCAGGCTTGAGAAGGGAGCTGGACAGGAATGGAG
TCTGCATAGCTTTTGTGGAAACAGTACCAGTTCTAGGGGAATCATTATATTCAATTCCTCTGCATACTCTGGTGCA
TATTTTGAAATCATCAGCAAACGTGATTATAATTTATGGTGACAGCACTTCTTTATTAACTGTAATAGTAAATACA
CGGGGAAAGTTCATATCTAGGAATGTTTGGGTCATGAACTCAAAATGGGGTGGTCACAGGTTTTATGGGTATACCA
TGTTAGATTCATTCCATAGGAGTCTCATTTTTTCATACCATCATGGAAAGATTTGGGGTTTCACAAAATTTATGCA
GGAAGTCACCCTATAAAGTACCCAGAAGACATTTATCTTCATATCTTTTGGAACTTGTATTTCAACTACTCACCTT
TGCACTCCAATTGTCAGATCTTTCAAAACTGTCTCCCAATGCCTCTTTGGAATTGTTGCTAGGGAACAATATTGAC
ATGGCCATGAGATTTACAATGAAGAGAGTTACAATGCATAC
>pr75_PS .
GGGCTATTCATCATAGATGATCACAAAGGTGCCAAGATTCTGTCAGATTTGAGAAGTTAGCTGGAGAAAAATGGAA
TCTGCATAGCATTTGTGGAAATGATCCTAGCCATCAGGGGTTCATTTCTGACCACATCCTCGAAAAATCAGGTGCA
GATTCTGGAATCATCAGCAAATATGATTATTATATATGGGGACACTGATTCTCTATTGAGCTTAATAATGAATATA
AGGCAGAAATTAGTCACATGGAAAGTCTAGGTCCTGAATTCACAATGGGGTCATTCCAAATTTGATGACTATTTCT
TGTTAGATGTGCTGCATGGGACCCTTATTTTTTCACACCATAAGAAGGAAATTATTAATTTTACAGATTTTATCCA
GACAGCCAATCCTTACAAATACCCACAAGACATTTATCTTCATGTATTGTGGCACTCATTTTTCAATTGCTCATTT
T
>pr76 .
AATCATCAGCAAATGTGATCATCACTTATGGTGACAGTGACTTTCTGAGAGGCTTCCTGTTTTATTTAAGACAAAC
TTTAGTTACAAGGAAAGTTTGGATCATGAACTCAGAATGGGATGTTATCCTCCATTCAAAGCATTTCATTTTGCAG
TCATTTCATGGAAGCCTCATTTTCTCACATCACCACAAAGAAATCTCTAGTTTCAGAAATTTTATCCAAACAGTTC
ACCCTTCCAAATACCCAGAAGATTTTTACCTTACAAAGTTGTGGTTCTACTTTTTTAACTGTTCATTTGCTGATGA
TGACTGCAATACACTGGAGAACTGTCTGCCTAACGTTTCCTTGCATCAATCACCTGGAAATCGTTTTGATACGGTC
ATGACTGAATATGCTTATAATACATAC
>pr77_PS .
AGATTGGTCCTTATAGATAACCATAAAGGAATTCAGATTCTATCAGACTGGAGAGGAGAGATGCACAGAAATAAAG
TGTGTGTAGCTTTCATGAAAATGATGCCTGAAAACTTGAGTTACTTGGACTATTCATTCAAAGGAACTATTGAGCT
GCTCCTGATATCATCGACAAACGTGGTTATCATTTATGATGACAACAAATCCTTACTTTTCATAATCCTATGTCAA
ATGCATTTGTTAATAGCATGGAAAGTCTGGGTCATAAATTCACAATTGGATGTTGGTACCATCAGAGAACCTTTTC
TTTTTGATTCATTTCATGGGACTCTCCTTTTTGCAAACCACCATGATGAGATTTCTGATTTTAGAAAGTTTATGCA
CAAATATACTCCTTCTAATTACCCAGAAGACCATTATCTTGCTCTTATTTGGAATATATTCTTCAATTGTTCTTTG

TCTGTACCTGATTGTAAAATATCAGGTAACTGTCTGTCCAATGCTTCATTGGAATTATTCCCTTCAAATCTGTGAA
ACATGGACACGACTGAAGAAGGTTACAATATCTAT
>pr79 .
GGGCTGGCCATCTCAGATGATGACCAGGGTCTCCAATTTCTCTCAGATCTGAGAGGAGAAAATGAAGTCCAAAGAG
TCTGCTTAGCCTTTGTGATCATGATCCCAGTCAACATGGAATTATACATGTCAAGACCTGAAGTGTATAACAACCA
AATTGAGACATCATCCACAAATGTTGTTATCATTTATGGTGACACAGGCAGTATTCTAGCTGTGTGCTTTAGAATG
TGGGTATCACAGGGTATACAGAGAATATGGGTCACCACCTCACAGTGGGATGTCACTACAAGTAAGAGAGACTTCA
CCCTTGACTCATTCCATGGGACTCTTGCTTTTCAGCACCACCATGCTGAGATTTCTGGTCTTAAAAATTTTGTTCA
GACACTGAACCCTTCTAAATACACAGACAAGTTCCTGGCAGGACTGAGATGGATGTACTTTAGCTGTGAAGCCTCA
ACATCTAACTGCCAGACACTGGAGAACTGCTCTTTGAACGTCTCACTGGAATGGTTAATGCTACAGAGTTTTGACA
TGGCCTTTAGTGATGATGCCTATGATATATAC
>pr80_PS .
GGGCTGGCCATCTCAGATGATGACCAGGGTCTCCAATTTCTCTCAGATCTGAGAGGAGTAGGTGAAGTCTAAAGAG
TCTGCTTAGCCTTTGTGATTGTGATCCCAGTCAACATGGAGTTATTCATGTCAAGAGCTGAAGTGTTTAACAACCA
AATTGAGACATCATCCACAAATGTTATCATTTATGGTGACACAGACAGTACTCTAGCTGGGCTTTAGAATGTGGGA
ATCACAGGTTATACAGTGAATATGGGTCACCACCTCACAGTGGGATGTCACTACAAGTAAGAGAGACTTCACCCTT
GATTCATTCCATGGGACTCTTGCTTTTCAACAACACCATGCTGAGATTTCTGGTTTTAAAAGTTTTGTTCAGACAC
TGAACCCTTGCAAATACACAGA
>pr82 .
GGTCTGGCCATCTCAGACGATGATCAGGGTACCCAATTTCTCTCACATTTGAGGAGAGAGATGGAAAAAAATGCAG
TCTGCTTTGCCTTTGTGAACACGATCCCAGCCAACATGAAGTTATACATGCCAAGAGCTGAACTGTATTATAACCA
AATAATGACATCATCCACAAATGTTGTTATCATTTATGGTGACACAGACAGTACTCTAGCTGTGAGCTTTAGAATG
TGGGAATCTCTAGGTATACAGAGAATATGGGTGACTACCTCACAGTGGGATGTCACTACAAGTAAGAGAGACTTTA
CCCTTGACTCATCCCTTGGGAAACTAGCTTTTGCACACCATCATGGTGAGATTTCTGGTTTTAAAAATTTTGTTCA
GACATTGAACCCTCTAAAATACACAGATGAATATCTGGCAAGTCTGGAGTGGATGAACTTTAACTGTGAAGTCTCT
GCACCCAACTGTAAGACCCTGAGGAACTTCTCATCGAATACCTCAGTGGATTGTCTAGTGGTAGAGACTTTTGACA
TGGCCTTTATTGATAGCTGTTATGATGTATAC
>pr83 .
GGGCTGGCCATCTCAGACAATGATCAAGGTATTCAATTTCTCTCATATTTGAGAACAGAGATGGAAAAAAATACAG
TCTGCTTTGCCTTTGTAAACATGATCCCGGTTAACATGGAGTTATACATGTCAAGAGCTGAAGTGTATTATAACCA
AATCATGACATCATCCACAAATGTAGTTATCATTTATGGAGACACAGACAGTACTCTAGCTGTGAGCTTTAGAATG
TGGGAATCTCTAGGTATACAGAAAATATGGGTCACCACTTCACAGTGGGATGATATTACAAGTAATAGAGACTTCA
CACTTACCTCATCTCTTGGAACAATAACTTTTGCTCATCACCATACTGAGATTTCTGGTTTTAAAAATTTTGTTCA
GACATTGACCCATGTCAGATACTCAGATGAATACCTGGCAAAGTTGGAGTGGATGAAATTTAACTGTGAAGTAGCA
GAATCTAAGTGGAAGACACTGAAGAATTGTTCAACCAATGCCTCACTGGAATGGCTGATGGTACAAACTTTTGACA
TGGCCTTTAATG
>pr86 .
GGACTGGCCATCTTAGACAATGACCATGGTACCCAATTTCTATCACATTTGAGAAGAGAGATGGAAAAAAATACAG
TCTGCTTTGCCTTTGTGAGTATGATCCCAGTCAACATGCACATATACATGTCAAGAGCAGAAGTGTATTATAACCA
AATCATGACATCATCCACAAATATAGTTATCATTTATGGTGACACAGACAGTACTCTAGCTGTGAGCTTTAGAATG
TGGGAATCTCTAGGTATAAAGAGAATATGGATGACCACTTCACAGTGGGATGTCACTACAAGTAAGAGAGACTTAA
CACTTGACACATACTATGGGACACTAGCTTTTGCACAACACCATTCTGAGATTTCTGGTTTTAAAAATTTTGTCCA
GACACTGAATCCTCTCAAATACTCAGATGAATACCTCACAAGGCTGGGATGGATGAATTTTAACTGTGAGGTCTCA
GCTTCTAAGTGTGAGACACTGAAGAATTGTTCATCCAATGCTTCATTGCAATGGCTAATGAGACAGCCTTTCGACA
TGGCTTTTAGTGATGACAGTTATGACATATAT
>pr87_PS .
GGACTGGCCACCTCTGACAATGACCAGGGTACCCAATTTCTCTCACATTTGAGAACTGAGATGGAGAAAAATACAG
TCTGCTTTGCCTTTGTAAGCATGATTCCAGCCAACATGCATTTATACATGTCAAGAGCTGAAGTGTATTGTAACCA
AATCATAACATCATCTGCAAATATAATTATCATTTATGGTGACATAGACAGTACTCTAGCTGTGAGCTTTAGAATG
TGGGAATCTCTAGGTATACACAGAATATTTTTTACCACATCACAGTGGGATGTCACTACAAGTAAAAGAGACTTCA
CAGTTGGCTCATCCCTTGGGATACTAGCTTTTACACACCACCATTCTGAGATTTCTGGTTTTAAAACATTTGTCCA
GACTCTGAACCCTCTGAAATACACAGATGAATACCTTGCAAGGCTGGGATGGATGAACTTTAACTGTGAGGTCTCA
GCTTCCAAGTGTAAGACAGTGAAGAATTGTTCATCCAATGCCTCATTACAATATCTAGTGGGACAGACTATTGACA
TGGCCTTTAGTGATGACAGTTATGACATGTAT
>pr88 .
GGACTGGCCATCTTAGACAATGATCAGGGTACCCAATTTCTATCACATTTGAGAAGAGAGATGGAAAAAAATACAG
TCTGCTTTGCCTTTGTGAGTATGATCTTAGTCAACATGCATATATACATGTCAAGAGCAGAAGTGTATTATAACCA
AATCATGACATCATCCACAAATATAGTTATCATTAATGGTGACACAAACAGTACTCTTGCTTTGAGCTTTAGAATG
TGGGAATCTCTAGGCATAAAGAAAATATGGGTGACCACTTCACAGTGGAATGTCACTACAAGTAAGAGAGACTTCG
CCATTGACTCATCGCATGGGACACTAGCTTTTGCACAACACCATTCTGAGATTTCTGGTTTTAAAAAATTTCTCCA
GACACTGAACCCTCTCAAATACTCAGATGAATACCTCACAAGGCTGGGGTGGATGAACTTTAACTGTGAGGTCTCA

GATTCTAAGTGTGAGACACTGAAGAATTATTCATCCAATGCTTCATTGCAATGGCTAATGACAGAGATGTTTGATA
TGGCTTTTAGTGATGAAGGTTATGACATATAT
>pr91 .
GGACTGGCCATTTCAGATAATGATCAGGGCACCCAGTTTCTCTCAGATTTGAGAAGAGAGATGGAACAAAATACAA
TCTGCTTTGCCTTTGTGAACATGATTCTGGTCAACATGCATTTATACATGTCAAGAGCTGAAGTGTATTGTAACCA
AATAATGACATCATCCGCAAATGTTGTTATCATTTATGGTGACACAGACAGTACTCTAGCTGTGAGCTTTAGAATG
TGGGAATCTCTAGGTATACAGAGAATATGGATCACCACCTCACAGTGGGATGTCATTCCAAGCAAGAGCGACTTCA
CACTTGACTCATCGTACAGGACTCTAGCTTTTGCACACCATCATGCTGAGATTTCTGGTTTTAAAAATTTCCTCCA
GACATTGAGCCCTCTCACCCCAAATGAAATCCTGGCAAGTCTGGAGTGGATGAAATTTAACTGTGAAGTTTCAGAT
TCTAAGTGTCAGACATTGATGAATTGCTCATCCAATGCCTCATTGGAATGGCTAAATTTAGAAACTTTTGATATGG
CATTTAATGATGGCAATTATGATATATAT
>pr94 .
GGACTGGCCATCTTAGACAATGACCAGGGTACCCAATTTCTATCACATTTGAGAAGAGAGATGGAAAAAAATACAG
TCTGCTTTGCCTTTGTGAGTATGATCCCAGTCAACATGCACGTATACATGTCAAGAGCAGAAGTGTATTATAACCA
AATCATGACATCATCCACAAATATAGTTATCATTTATGGTGACACAGACAGTACTCTAGCTGTGAGCTTTAGAATG
TGGGAATCTCTAGGTATAAAGAGAATATGGGTGACCACTTCACAGTGGGATGTCACTACAAGTAAGAGAGACTTCG
CCATTGACTCATCGCATGGGACACTATCTTTTATACACCACCATTCTGAGATTTTTGGTTTTAAAAAATTTGTCCA
GACACTGAACCCTCTCAAATACTCAGATGAATACCTCACAAGGCTGGGGTGGATGAACTTTAACTGTGAGGTCTCA
GATTCTAAGTGTGAGACACTGAAGAATTGTTCATCCAATGCTTCATTGCAATGGCTAATGAGACAGCCTTTCGACA
TGGCTTTTAGTGATGACGGTTATGA
>pr95 .
GGACTGGCCATCTCTGACAATGACCAGGGTACCCAATTTCTCTCACATTTGAGAACCGAGAATGAGAAAAATACAT
TCTGCTTTGCCTATGTAAGCATGATCCCAGCCAACTTCCATTTATACATGTCAAGACCTGAAGTGTATTATAACCA
AATCATAACATCATCTTCAAATGTAGTTATCATTTATGGTGACATTGACAGTACTCTAGCTGTGAGCTTTAGAATG
TGGGAATCTCTAGGTATACACAGAATATTTTTGACCACATCACAGTGGGATGTCACTACAAGGAAGAGAGACTCTA
CAGTTGGCTCAGCCCATGGGACACTATCTTTTTCACACCATCATCCTGAGATTTCTAGTTTTAAAACCTTTGTCCA
GACATTGAACCCTCTGAAATACACAGATAAATACCTTGCAAGGCTGGGATGGATGAACTTTAACTGTGAAGTCTCA
GCTTCTAAGTGTAAGACACTGAAGAATTGTTCATTCAATGCCTCATTACAATTTCTAATGGGACAGACTATTGACA
TGGACTTTAGAGATGACAGTTATGACATATAT
>pr96_PS .
GGGCTGGCCATCTCAGATGATGACCAAGGTCTCCAATTTCTCTCAGATCTGAGAGGAGAATGTGAAGTCCAAAGAG
TCTGCTTAGCCTTTGTGATCATGATCCCAGTCAACATGGAGTTATACATTTCAAGAGCTGAAGTGTATAACAACCA
AATTGAGACATCATCCACAAATGTTGTTATCATTTATGGTGACATAGACAGTACTCTAGCTGTGTGCTTTAGAATG
TAGAAATCACAGGGTATACAGAGAATATGGGTCACCACCTCACAGTGGGATACCACTACAAGTAAGAGAGACTTCA
CCCTTGACTCATTCCATGGAACTCTTGCTTTTGCACACCACCATGCTGAGATTTCTGGTCTTAAAAATTTTGTTCA
GACACTGAACCCTTCCAAATACACAGACAAGTTCCTGGCAGGACTGGGGTGGATGTACTTTAGCTGTGAAGCCTCA
ACATCTAACTGTAAGACACTGGAGAACTGCTCTTTGAACGTCTTACTGGAATGGTTAATGCTACAGAGTTTTGACA
TGGCTTTCAGTGATGATGCTTATGATATATAC
>pr97_PS .
GGGCTGGCCATCTCAGATGATGACCAGGGTCTCCAATTTCTTTCAGATCTGAGAGGAGAAATTGAAGTCCAAAGAG
TCTGCTTAGCCTTTGTGATCATGATCCCAGTCAACATGGAGTTATTCATGTAAAGAGCTGAAGTGTTTAACAACCA
AATTGAGACATCATCCACAAATATTGTTATCATTAATTGTGACACAGACAGTACTCTGGCTGTGTGCTTTATAATG
TGGGTATCACAGGGTATACAGAGAATATGGGTCACCACCTCACAGTGGGATGTCACTACAAGTAAGAGAGACTTTA
TCCTTGACTCATTCCTTGGGACTCTTTCTTTTGAACACAACCATGCTGAGATTTCTGGTTTTAAAAATTTTGTTCA
GACACTGAACCCTTCCAAATACACAGACATGTTCCTGGCAGGACTGGAGTGGATGTACTTTAGCTGTGAAGCCTCA
GCATCCAACTGTAAGACACTGGAGAACTGCTCTTTGAACATCTCACTGGAATGGTTAATGCTACAGAGTTTTGACA
TGGCCTTTAGTGATGATGCCTATGATATATAC
>pr98 .
GGGCTGGCCATCTCAGATGATGACCAGGGTCTCCAATTTCTCTCAGATCTGAGAGGAGAAACTGAAGTCCAAAGAG
TCTGCTTAGCCTTTGTGATCATGATCCCAGTCAACATGGAGTTATACATGTCAAGAGCTGAAGTGTATAACAACCA
AATTGAGACATCATCCACAAATGTTGTTATCATTTATGGTGACACAGACAGTACTCTAGCTGTGTGCTTTAGAATG
TGGGTATCACGTGGTATACAGAGAATATGGGTTACCACCTCACAGTGGGATGTCATTACAAGTAAGAGAGACTTCA
CAGTTGATTCATTCCATGGGACTTTTGCTTTTGCACACCACCATGCTGAGATTTCTGGTCTTAAAAATTTTGTTCA
GACATTGAACCATTCCAAATACACAGACATGTTCCTGGCCCGACTGGGGTGGATGTACTTTAGCTGTGAAGCCTCC
ACATCTAACTGTAAGACACTGGAGAACTGCTCATTGAACGTCTCACTGGAATGGTTAATGCTACAGAGTTTTGATA
TGGCCTTTAGTGATGGTGGTTATGATATATAC
>pr110 .
GGGCTGGCCATCTCAGATGATGACCAAGGTCTCCAATTTCTCTCAGATCTGAGAGGAGAAAGTGAAGTCCAAAGAA
TCTGCTTATCCTTTGTGATCATGATCCCAGTCAACATGGAGTTATACATGTCAAGAGCTGAAGTGTATAACAACCA
AATTGAGACATCATCCACAAATGTTGTTATCATTTATGGTGACACAGACAGTACTCTAGCTGTGTGCTTTAGAATG
TGGGTATCACATGGGATACAGAGAATATGGGTCACCACCTCACAGTGGGATGTCATTACAAGTAAGAGAGACTTCA
CCCTTGACTCATTCCATGGGACTCTTGCTTTTGCACACCACCATGCTGAGATTTCTGGTTTTAAAAATTTTGTTCA

GACAATGAACTCTTCCAAATACACAGACCTATTCCTGGCCCAACTGGGGTGGATGTTCTTTAGCTGTGAAGCCTCA
ACATCTAACTGTAAGACACTGGAGAACTGCTCTTTCAATGCCTCACTAGAATGGTTAATGGAACAGAGTTTTGACA
TGGCCTTTAGTGATGATGCTTATGATATATAC
>pr112 .
GGGCTGGCCATCCCAGACAATGATCATGGTATCCAGTTTCTCTCATATTTGAGAAGAGAAATGGAAGAAAATATAA
TCTGCTTTGCCTTTGTGAACATGATTTCAGTCAACATGCACTTATACATGTCAAGAGCTGAAGTGTATTATAGACA
AATCATGACATCATCCTCAAATGTTGTTATCATTTATGGTTATACAGACAGCACTCTAGCTTTGAGCTATAGAATG
TGGGAATCTCTAGGTATACAGAAAATATGGGTCACCACCTCACAGTGGGATGTCACTACAAGTATGAGAGACTTAA
CACTTAACTCATCTCAAAAGACTCTAACTTTTGCACACCATCATGCTGAGATTTCTGGATTTAAAAATTTCCTCCA
TACCTTGAGCCCTCTCACTCCAAATGAATATCTGGCAAGTCTGGACTGGATGAACTTTAACTGTGAAGTCTCAGCT
TCTAATTGTAAGACACTGAAGAATTGCTCATCCAATGCCTCACTGGAATGGCTAATATTGCACACTTTTGACATGA
CTTTTAGTGATGAGAATTATGATATATAT
>pr116 .
GGGCTGGCCATCTCAGATGATGACCAGGGTCTCCAATTTCTCTCAGATCTGAGAGGAGAAATTGAAGTCCAAAGAG
TCTGCTTAGCCTTTGTGATCGTGATCCCAGTCAATATGGAATTATACATGTCAAGAGCTGAAGTGTATAACAACCA
AATTGAGACATCATCCACAAATGTTGTTATCATTTCTGGTGACACAGGAAGTACTCTAGCTGTGTGCTTTAGAATG
TGGGTATCACAGGGTATGCAGAGAATATGGGTCACTACCTCACAGTGGGATGTCACTACAAGTAAGAGAGACTTCA
CCCTTGACTCATTCCATGGGACTCTTGCTTTTGAACACCACCATGCTGAGATTTCTGGTTTCAAAAATTTTGTTCA
GACACTGAACCCGTCCAAATACACAGACATGTTCCTGGCAGGACTGGGGTGGATGTACTTTAGCTGTGAAGCCTCC
ACTTCTAACTGTAAGATACTGGAGAACTGCTTATTGAACATCTCACATGAACGGTTAATGCTACAGAGTTTTGACA
TGGCCTTTAGTTATGATGCTTATGATATATAC
>pr117 .
GGGCTGGCCATCTCAGATGATGACCAGGGTCTCCAATTTCTCTCAGATCTGAGGGGAGAAACTGAAGTCCAAAGAG
TCTGCTTAGCCTTTGTGATCATGATCCCAGTCAACATGGAGTTATACATGTCAAGAGCTGAAATGTATAACAACCA
AATTGAGACATCATCCACAAATGTTGTTATCATTTATGGTGACACAGACAGTACTCTAGCTGTGTGCTTTAGAATG
TGGATATTACAGGGTATACAGAGACTATGGGTCACCACCTCACACTGGGATGTCATTACAACCATGAGAGACTTCA
CCCTTGACTCATTCCATGGGACTCTTGCCTTTGCACACCACCATGCTGAGATTTCTGGTTTTAAAAATTATGTTCA
GACATTGAACCCTTCCAAATACACAGACATGTTCCTGGCAGGACTGGGGTGGATGTACATTAGCGGTGAAGCCTCA
ACATCTAGCTGTAAGACACTGGAGTACTGCTCATCTAACGTCTCACTGGAATGGTTAATGCTACAGAGTTTTGACA
TGGCCTTTAGTGATGATGCCTATGGTATATAC
>pr118_PS .
GGGCTGGCCATCTCAGATGATGACCAGGGTCTCCAATTTCTCTCAGATCTGAGGGGAGAAACTGAAGTCCAAAGAG
TCTGCTTAGCCTTTGTGATCATGATCCCAGTCAACATGGAGTTATACATGTCAAGAGCTGAAATGTATAACAACCA
AATTGAGACATCATCCACAAATGTTATCATTTATGGTGACACAGACAGTACTCTAGCTGTGTGCTTTAGAATGTGG
ATATTACAGGGTATACAGAGAATATGGGTCACCACCTCACACTGGGATATCATTACAACCATGAGAGACTTCACCC
TTGACTCATTCCATGGGACTCTTGCTTTTGCACACCACCATGCTGAGATTTCTGGTTTTAAAAATTATGTTCAGAC
ATTGAACCCTTCCAAATACACAGACAAGTTCCTGGCAGGACTGGGGTGGATGTACATTAGCGGTGAAGCCTCGACA
TCTAACTGTAAGACACTGGAGTACTGCTCATCTAATGTCTCACTGGAATGGTTAATGCTACAGAGTTTTGACATGG
CCTTTAGTGATGATGCTTACAATATATAC
>pr122 .
GGGCTGACCATCTCAGACAATGATCATGGTATCCAGTTCCTCTCATATTTGAGAAGAGAAATGGAAGAAAAAACAG
TCTGCTTTGCCTTTGTGAACATGATTCCAGTCAACATGCATTTATATGTGTTAAGAGCTGAAACATATTATAAGCA
AATCATGACATCATCCTCAAATGTTGTTATCATTTATGGTGATACAGACAGTACTCTAGCTTTGAGCTATAGAATG
TGGGAATCTTTAGGTATACAGAAAATATGGGTCACCACCTCACAGTGGGATGTCACCACAAGAATGAGAGACTTTA
CATTTGACGCACCTCAAAAGACTCTAACTTTTGCACACCATCATGCTGAGATTTCTGGTTTTAAACTTTTTCTCCA
GACTTTGAGCCCTCTCACCACAAACACATACCTGGCAAGTCTGGATTGGATGAACTTTAACTGTGAAGTCTCAGCT
TCTAATTGTAAGACACTGAAGAATTGCTTATCCAATGCCTCATTGGAATGGCTAGGATTACAGACTTTTGACATGG
CTTTTAATGATGCCAACTATGATATATAT
>pr123 .
GGACTGACCATCTCAGACAATGATCATGGTATCCAGTTTCTCTCATATTTGAGAAGAGAAATGGAAGAAAAAACAG
TCTGCTTTGCCTTTGTGAACATGATTCCCATCAACATGCACTTATATGTGTCAAGAGCTGAAACATATTATAAGCA
AATCATGACATCATCCTCAAATGTTGTTATCATTTATGGTGATACAGACAGTACTCAAGCTTTGAGCTATAGAATG
TGGGAATCTTTAGGTATACAGAAAATATGGGTCACCACCTCACAGTGGGATGTCACCACAAGAATGAGAGACTTTA
CATTTGATGCACCTCAAAAGACTCTAACTTTTGCACACCATCATGCTGAGATTTCTGGTTTTAAAATTTTTCTCCA
GACGTTGAGCCCTCTCACCACAAACACATACCTGGCAAGTCTGGAATGGATGAACTTTAACTGTGAAGTCTCAGCT
TCTAATTGTAAGACACTGAAGAATTGCTTATCCAATGCCTCATTGGAATGGCTAGGATTACAGACTTTTGACATGG
CTTTTTATGATGCCAACTATGATATATAT
>pr126_PS .
GGGCTGGCCATCTCAGATGATGACCAGGGTCTCCAATTTCTCTCAGATCTGAGAGGAGAAACTGAAGTCAAAGGAG
TCTGCTTAGCCTTTGTGATCATGATCCCAGTCAACATGGAGTTATACATGTCAAGAGCTGAAGTGTATAACAACCA
AATTGAGACATCATCCACAAATGTTGTTATCATCTATGGTGACACACACAGCACTCTGTGTGCTTTAGAATGTGGA
AATCACGAGGTATACAGAGAATATGGGTCACAACCTCACAGTGGGATGTCACTACAAGTAAGAGAGATTTCACACT

TGACTCATTCCATGGGACTCTTGCTGAGATTTCTGGTCTTAAAAATTATGTTCAGACATTGAACCCTTCCAAAATA
CACAGACAAATTCCTGGCAGGACTAGGGTGGATTACTTTAGATGTGAAGCCTCAACATCTAACTGTAAGATACTGG
AGAACTGCTCATCTAATGTCTCACTGGAATGGTTAATGCTACAGACTTTTGACATGGCCTTTAGTGTTGATGGTTA
TGATGTATAC
>pr129_PS .
GGGCTGGCCATCTCAGATGATGACCAGGGTCTCCAATTTCTCTCAGATCTGAGAGGAGTAGGTGAAGTCTAAAGAG
TCTGCTTAGCCTTTGTGATTGTGATCCCAGTCAACATGGAGTTATTCATGTCAAGAGCTGAAGTGTTTAACAACCA
AATTGAGACATCATCCACAAATGTTATCATTTATGGTGACACAGACAGTACTCTAGCTGGGCTTTAGAATGTGGGA
ATCACAGGTTATACAGTGAATATGGGTCACCACCTCACAGTGGGATGTCACTACAAGTAAGAGAGACTTCACCCTT
GATTCATTCCATGGGACTCTTGCTTTTCAACAACACCATGCTGAGATTTCTGGTTTTAAAAGTTTTGTTCAGACAC
TGAACCCTTGCAAATACACAGACATGTTCCCAGCAGGACTGGGGTGGATGTCCATTAGCTGTGAAGTCTCAACATC
TAACTGAAAGACACTGGAGAACTGCTTATTGAACGTCTCACTTGAACAGTTAATGCTACAGAGTTTTGACATGGCC
TTTAGTGATGAGGCTTATGATATATAC
>pr131 .
GGGCTGGCCATCTCAGATGATGACCAGGGTCTCCAATTTCTCTCAGATCTGAGAGGAGAAAGTGAAGTCCAAAGAA
TCTGCTTATCCTTTGTGATCATGATCCCAGTCAACATGGAGTTATACATGTCAAGAGCTGAAGTGTATAAAAACCA
AATTGAGACATCATCCACAAATGTTGTTATCATTTATGGTGACACAGACAGTACTCTAGCTGTGTGCTTTAGAATG
TGGGTATCACATGGGATACAGAGAATATGGGTCACCACCTCACAGTGGGATGTCATTACAAGTAAGAGAGACTTCA
TCCTTGACTCATTACATGGGACTCTTGCTTTTGAACACCACCATGCTGAGATTTCTGGTTTTAAAAATTTTGTTCA
GACATTGAACTCTTCCAAATACACAGACCTATTCCTGGCCCAACTGGGGTGGATGTACTTTAGCTGTGAAGCCTC
>pr135_PS .
GGGCTGTCTATCTTAGACAATGATCAGGCTATACAATTTCTTTCACATTTGAGAAGAGACAATGGAAAAAATACAG
TCTGCTTCACCTTTGTAAGCATGATGTCAGGCAGCATATCAAAAGCTGAAGTGTATTATAACAAAATCATGCCACC
ATCCATGAATGTAGTAATTATTTATGGAGACACAAACAGAACTCTAGCTTTGGGCTTTTGGCTGTGGGACTCTCTA
GATATACAGAGAATATGAGTCATCACCTCACAGTGGGATGTCAATACAATTAAGAGAAGATTCACACTTGACTCAA
CCCTTGGGACTCTAGCTTTTGCACAAACCATCCTTCAATTTCTGGTTTCAATTTTTTGTCTGGACATTGACCCATC
TCAAATACTCAGATGAATACCTGGCAAGGATGGGGAAAAAAGTTTAACTGTGAACTGTTGGCTTCTAAGTGTAAGA
CACTGAAAAACTGCTTATCCAAAGCCGTGTTGGAATGGATAATGTTCCAGACTATTGAAATGGCCATTAGTGATGA
AAGTTATGATGTATAC
>pr137_PS .
GGGCTGTCTATCTTAGAAAATGACCATGCTATACAATTTCTTTCACATTTGAGAAGAGACATGGAAAAAAGTCTGC
TTCACCTTTGTGAGCATGATCTCAGGCATCATGTCTAAAGCTGAATTGTATTATACCATATCATGACACAATCCAT
GAATGTAGTAATTATTTATGGCGACACAAATAGAATTCTAGTTTTGAGCTTTTGTATGTGGGGCTTTCTAGGTATA
CAGAGAATATGAGTCACCACCTCACAGTGGGATGTCACTACAAGTAAGAGAAGATTCACGCTTGACTCATCCCTTG
GGGCTCTAGCATTTGCACAAAACCATCCTTCGATTTCTGGTTTTATTTTTTTGATCTGGACATTGATCCATCTCAA
ATACTCAGGTGAATACCTGGCAAGGATGGGGAATAAAATTTAACTGTGAACTGTTGGCATCTAAGTGTAAGACACT
GAAAAACTGCTCATCCAATGCATCGTTGGAATGGCAAATATTCCAGACTATTGACATGGCCATTGGTGATGGACGT
TATGATGTATAC
>pr138_PS .
GGGCTGTCTATCTTAGACAATGATCAGGCTATACAATTTCTTTCACATTTGAGAAGAGACATGGAAAAAATACAGT
CTGCTTCACCTTTGTGAGCATGATCCCGAGCTGCATATTAAAAGCTGAAGTGTGTTATAACCAAATCATGACACCA
TCCATGAATGTAGTAATTTTTTATGGTGACACAAACAGAATTCTAGCTTTGAGCTTTTGTATGTGGGACTCTCTAG
GTATACAGAGAATATGAGTCACCACCTCACAGTGGGATGTCACTACAAGTAAGAGAAGATTCATGCTTAACTCATC
CCTTGGGACTCTAGCTTTTGCATAAAACCATCATTTGATTTCTGGTTTCAATTTTTTGTCTGGATATTGACCCACC
TCAAATACTCAGCTGAATACCTGGCAAGACTGGGGAATAAAACTTAACTGTGAACTGTTGGCATCTAAGTGTAAGA
CACTGAAAAACTGCAAATCCAATGCCTTGTTGGAATGGATAATGTTCCAGACTATTGAAATGGCCATTAGTGATAG
AAGTTACGATGTATAC
>pr139_PS .
TGTGAGCTTGATCCCAGTCAAGATGGATTTATACATGTCAAGAGCTGAAGTGTATTATAACCAAATCATAACATCA
TCTACTAATGTAGTTATCATTTATGGTGACACAGACAGCACTCTAGCTGTGAGCTTTAGAATGTGGGAATCTCTAG
GTATACAGAGAATATGGGTTACCACCTCACAGTGGGATGTCACTACAAGTAAGAGAGACTTAACACTTGACACATC
CCATAGGACACTAGCTTTTGCACAACACCATTTGAGATTTCTGTTTTAAAACTTTTGTCCAGACACTGAACCCTCT
CAAATGCTCAAATGAATACCTTGCAAGGCTGGGATGGATGAGCTTTAACTGTGAAGTCTCAGCTTCTAAGTGTGAG
ACACTGAAGAATTGCTCATCCAATGCTTCGTTGGAATAACTAATGAGAGAGATGTTCGACATGACTTTTAGTGATG
CCAGTTATGACATATAT
>pr140_PS .
GGGTTGTCTATCTTAGACAATGATCAGGCTATACAATTTCTTTCACATTTGAGAAGAGACATGGAAAAAATACAGT
CTGCTTCATCTTTGTGAGCATGATCCCGGGCAGCATGTCAAAAGCTGAAGTGTATTATACCAAATCATGACACCAT
CCATGAATGTAGTAATTATTTATGGTGATACAAACAGTATTCTAGCTTTGAGCTTTTGAATGTGAGGCTATCTAGG
TATACAGAGAATATGAGTCACCACCTCACAGTGGGATGTCACTACAAGTAAGAGAAGATTCACACTTGACTCATCC
CTTGGGACTCTAATTATTTGCACAAAACCATCCTTCGATTTCTGGTTTCAATTTTTTTTTGTCGTCTGGACATTGA
CCCATCTCAAATACTCAGATGAATACCTGGCAAAGCTCGGGAATAAAATTTAACAGTGAACTGTTGGCATCTAAGT

GTAAGACACTGAAAACTGCTCATCCAATTCCTCATTGGGATGGCCAATGTTCCAGACTATTGACATGGCCATTGGT
GATGGAAGTTACGATGTATAC
>pr141_PS .
GGGCCGTCTCTCTTAGACAATGATCAGGCTTTACAATTTCTTTCACATTTGAGAAGACACATGGAAAAAATACAGT
CTGCTTCACCTTTGTGAGCATGATCCTGGGCAGCATGTCAAAAGCTGAAGTGTATTATACCAAATCATGACACCAT
CCATGAATGTAGTAATTATTTATGGTGACACAAACAGTATTTTAGCTTTGAGCTTTTGTATGTGGGACTCTCTAGG
TATACAGAGAATATGAGTCAGCACCTCACATTGGATTGTCACTACAAGTAAGAGAAGATTCACACTTGACTCATCC
CTTGGGACTCTAGCTTTTACACAAAACCATCCTTGGATTTCTGATTTCAATTTTTTTCTCTGGACATTGACCCAAC
TCAAATACTAAGATGAATACCTGGCAAGACTGGGGAATAAAATTTAACTGTGAACTGTTGGCATCTAAGTGTAAGA
CACTGAAAAACTGTTTATCCAATGCCTAGTTGGAAATGGCTAATGTTCTAGACTATTGACATGGCCCTTAGTGACG
GAAGTTATGATGTATAC
>pr143_PS .
GGACTGTCCATCTTAGAAAATGATCAGACCACTTTATTTATTTCACATTTGAGAACAGACATGAAAAAAATATTGT
TTTACCTTTGGGATCATGATCCTAGGCAAGAAGTATTTTTACATGTCAAGAGCAGATGTGTATTATAACCAAATCA
TGACATATCCATAAATATAGTTGTGATTTATAGTGACACAAACAGTATTCAAGCTGTGAGCCTTAGAATGTTGGAT
CTCTAAGTATACAAAGAATATGGGCTACCACTTCACAGTGGGATGTCACTACAATTAAGAGAGGATTCACACAACT
CATCTATCAGGACTCTAGCTTTTGCACCCCTCTATACTGAGATTTCTGGTTTTAAAATTTAGTCAAGACATTAATC
CATCTCAAATACTCAGATGATTACCTGGAATGGCTGGGTGGATTAATTTTAACTGTGAGGTCTTGGAATCTAATTG
TAAGACACTGAAAAACTGCTCATCCAATGCCTTGTTGGAATGGCTAATGGTCAGGACTATTGACATGGCCTTTAGT
TGTGGCAGTCACACTGTATAC
>pr145 .
GGGCTGGTTGTCTCAGATGATGACCAGGGAATTCAGTTTCTCTCAGACTTGAGAGAAGAAATTCAAAGAAATGGAG
TCTGTTTAGCTTTTGTGAATGTGATTCCAGATAGTATGCAATTATATACAGCAAGGGCTGGAATATATGACAAACA
AATAATGACATCATCAGCAAAGGTTGTTATTATTTATGGTGAAATGAACTCTACCCTAGAAGTCAGCTTCAGAAGA
TGGGGATGTTTAGGTATAAAGAGAATCTGGGTCACCACCTCACAATGGGATGTCACCACAAGTAACAGAGATTTCA
GCCTTGACCCCTTCCAAGGGACTGTCACTTTTGCACACCACCATGGCAAGGTTTCTAAATTTAGGAATTTTATGCA
AACAATGAACACTTCCAAATATCCAGTAGACATTTCTCAGATGAGAACAAAGTGGAATTATTATAATTGTTCAGTC
TCTGAGACCAACTATAGTTCAATGAATCATTGTTCATTCAACACTACATTGGAATGGTTTTCACAACACAGATTTG
ACATGGTCCTGAGTGAAGAAGGTTACAATTTATAC
>pr147 .
GGGCTGGTTGTCTCAGATGATGACCAGGGAATTCAGTTTCTCTCAGACTTGAGAGAAGAGATGCAAAGAAAAGGAG
TCTGTTTAGCTTTTGTGAATGTGGTCCCAGAAAACATGCAGTTATACATGACAATGGCTGGAATATATGAAAAACA
AATCATGACATCATCAGCAAAGGTTGTTATCATTTATGGTGAGATGAACTCTAACCTTGAAGTCAGTTTTAGAAGA
TGGGAATATTTAGGTATACACAGAATTTGGGTCACCACCTCAAGATGGGATGTCACCACAAGTAAGAGAAATTTCA
GCCTTGACCCTTTCTATGGGACTGTCACTTTTGCACATCACCATGATAAGGTTTCTAAATTTTCGAACTTTATGCA
AACAATGAACACTTCCAAATATCCAATAGACATTTCTCAGATGAGAACAAAGTGGAATTATTTTAATTGTTCAATC
TATAAGACCAACTACAGCTCAATGAATCATTGCTCATTCAACACTACATTGGAATGGTTATCACAACACAAATTTG
ACATGGTCCTGAGTGAAGAAGGTTACAATTTATAC
>pr153_PS .
GGAGCAATCATTTCAGATGATGACCTAGGACTTGAATTTCTCTCAGAATTGAGAAGAGAGATGCAAAGAAACAGTG
TGTGCTTAGCCTTTGTGCATATTATTATGGAAGATAAAATATTATTCCAGAAAAATGTAAATATTTATTATAATGA
GATCACAGCATCATCAGCCAAAGTTGTTATAATTTATGGAGACAAAGACTCTCATCTGCAACTTAACCTTAGATTC
TACAGACTAGCATACCTTCAGAGGATCTGGGTCACTACTTCACAGTGGGATATGATCACACATAATGACAGACTCC
TTCTTGATTCCTTCTATGGGACCTTTGTATTTTTACCTCACTTTTCTGAATTATCTGGTTTTAAAAAATTTATTGA
GACA
>pr154_PS .
GGAGCAATCATTTCAGATGATGACCTAGGACTGGAATTTCTCTCAGAATTAAGAAGAGAGATGCAAAGAAACAGTG
TGTGCTTAGCCTTTGTGCATATTATTATGGAAGATAAAATGCTATTCCGGAAAAATGTAAATATTTATTATAATGA
GATCACAACGTCATCAGCCAAAGTTGTTGTCATTTATGGAGACAAAGACTCTCATCTGCAACTTAACCTTAGACTC
TACAGATTATCAAACCTTAAGAGGATCTGGGTCACTACTTTACAGTGGGATATGATAACACATAATGGCAGATTAC
TTCTTGATTCATTCTATGGGACATTTACTTTTTTACTTCACTTTTCTGAATTACCTGGTTTAAAAAAATTTATTGA
GACA
>pr6 .
ATCATTCTGGCCCATGATGATGATTTTGGGCAGCAGGCCAGCTCTCTGGCCATTCAGGAGCTGAGCACAGCTGGTG
TGTGCATTGAGTACACCCTCCATGTTCCTTCTCATGAGTCCTTGGGGAAGATTGAAGAGATTGTCCAGAAGATGCA
AAAATCCACAGCCAGGGTTGTTCTGGTTTTCCTAAGCAATGCAAATTTCCAGCTTATCCTGTATGGCTTACTGGAT
GTCCCTGTCTCAGGCCAGGTCTGGGTCAGCAAGGACACTCTGCACATGGCACTCGCCCTGACCATTCCAGGCATTT
CCCAGGTATTGCACAGCACATTTGGCCTTCTGTATCACAGCAGCAGGGCAACTGGCTTCCCTGAGTTCCTTGCTTA
CCTGAGGCCCAGCCAGACCCCAGAAGACATGTTTATAAAGAAGTTTTGGGAGTTCACCTTTGATTGTACATGGCCC
CACCAGAGCAGCACAGTGACAGAGGGTGTCCAGTTGTGCTCAGGGAATGAGAGTCTGAAAAACCAGCAGTATGCTC
TCCCAGAAGTGAGTAAAATTGATGCTGCTTACACAGCTGT
>pr7 .

ATCATTCTGGCACATGATGATGAATATGGGCAGCAGGCCAGCTCTCTGGCCACTCAGGAGCTTAGCCCAGCTGGTG
TGTGCATTGAGTTCACTTTCCATGTTCCTTCCCATGAGTCCATGGGGAAAATTAAAGAGATTGTCCAGAAGATGCA
AAAATCCACAGCCAGGGTTGTTCTGGTTTTCCTAAGCAATGCAAATTTCCAGCTCATCCTGTATGGCTTACTGGAT
GTCCCTGTCTCAGGCCAGGTCTGGGTCAGCAAGGACACTCTGCACATGGCACTCGCCCTGACCATTCCAGGCATTT
CCCAGGTATTGCACAGCACATTTGGCCTTCTGTATCACAGCAGCAGGGCAATTGGCTTCCCTGAATTCCTTGCTAA
CCTGCAGCCCAGCCAGATCCCAGAAGACATGTTTATAAAGAAGTTTTGGGAGTTTACCTTTGATTGTACATGGCCC
CACCAGAGCAGCACAATGACAGATGCTATCCAGTTGTGCTCAGGGAATGAGAGTCTGAAAAACAAGCAGTACCCTT
TTGCAGAAGTGAGTAAAATTGATGCTGCTTACACAGCTGT
>pr8 .
ATCATTCTGGCCTATGATGATGACTTTGGGCAGCAGGCCAGCTCTCTGGCCACTCAGGAGCTGAGTACAGCTCGTG
TGTGCATTGAGTACACCCTCCATGTTCCTTCCCACGAGTCCCTGGAGAAGATTGAGCAGATTGTCCAGAAGATGCA
AAAATCCACAGCCAGGGTTGTTCTGGTTTTCCTAAGCAGTTCTAATTTCGAGCTCATCCTGTATGGCTTACTGGAT
GTCCCTGTCTCAGGCCAGGTCTGGGTCAGCAAGAACATTCTTCACATGGTGCTCACCATCACCGTTCCAGGCATTT
CCCAGGTATTACACAGCACATTTGGCCTTCTGTATCACAGCAGCAGGGCAATTGGCTTCTCTGAGTTCCTTGCTAA
CCTGCGACCCAGCCAGACCCCAGAAGACATGTTTATAAAGAAGTTCTGGGAGTTCACCTTTGATTGTACATGGCCC
CACCAGAGCAGCACAATGACAGAGGGTGTCCAGTTGTGCTCAGGGAATGAGAGTCTGAAAAACAAGCAGTACCCTT
TCCCAGAAGTGAGTAAAATTGATGCTGCTTACACAGCTGT
>pr9 .
ATCATTCTGGCCCATGATGATGATTTTGGGCAGCAGGCCAGCTCTCTGGCCACTCAGGAGCTGAGCACAGCTGGTG
TGTGCATTGAGTACACCATCCATGTTCCTTCTCATGAGTCCTTGGGGAAAATTAAAGAGATTGTCCAGAAGATGCA
AAAATCCACAGCCAGGGTTGTTCTGGTTTTCCTAAGCAATGCAAATTTCCAGCTCATCCTGTATGGCTTACTGGAT
GTCCCTGTCTCAGGCCAGGTCTGGGTCAGCAAGGACACTCTGCACATGGCACTCGCCCTGACCATTCCAGGCATTT
CCCAGGTATTTCACAGCACATTTGGCCTTCTGCCTCACAGCAGCAGGGCAACTGGCTTCCCTGAGTTCCTTGCTCA
CCTGCGGCCCAGCCAGACCCCAGAAGACATGTTTATAAAAAAGTTTTGGGAGTTTACCTTTGATTGTACATGGCTC
CACCAGAGCAGCACAGTGACAGAGGGTGTCCAGTTGTGCTCTGGGAATGAGAGTCTGAAAAACCAGCAGTATGCTC
TCCCAGAAGTGAGTAAAATTGATGCTGCTTACACAGCTGT
>pr10 .
ATCATTCTGGCCCATGATGATGACTTTGGGCAGCAGGCCAGCTCTCTGGCCACTCAGGAGCTGAGCACAGCTGGTG
TGTGCATTGAGTACACCCTCCATGTTCCTTCTCATGAGTCCTTGGGGAAGACTGAAGAGATTGTCCAGAAGATGCA
AAAATCCACTGCCAGTATTGTTCTGCTTTTCCTAAGCAATTCTAATTTCGAGCTCATCCTGTATGGCTTACTGGAT
GTGCCTGTCTCAGGCCAGGTCTGGGTCAGCAAGAACATTCTTCACATGGTGCTCACTGTGACCATTCCAGGCATTT
CCCGAGTATTACACAGCACCTTTGGACTTCTGCATCACAGCAGCACTGCAATTGGCTTCCCTGAGTTCCTTGCTCA
CCTGCGGCCCAGCCAGACCCCAGAAGACATGTTTATAAAGAAGTTCTGGGAGTTCACCTTTGATTGTACATGGCCC
CACCAGAGCAGCACAGTGACAGAGGGTGTCCAGTTGTGCTCAGGGAATGAGAGTCTGAAAAACAAGCAGTACGCTT
TCCCAGAAGTGAGTAAAATTGATGTTGCTTATACATCTGT
>pr2 .
GGCGCTATTGCAGCTGATGACGATTATGGAAAATATGGAGTAAAAACTTTTAAGGAAGAAATGGAGACTGCTAACC
TCTGTGTTGCTTTCTCTGAAACCATTCCCAAAGTCTACTCCAATGAGAAAATGAAAAAAGCTGTTGATGCAGTAAA
GGGTTCCACGGCCAGAGTCGTTGTGCTTTATACCTCTGACATTGACCTCAGCCCCTTTGTACTGGAAATGATTCAT
CACAACATAACAGACAGGACATGGATAGCCAGCGAAGCCTGGATTACCTCAGCCCTCATTGCAAAGCCTGAGTACT
TTCCATATTTTGGAGGAAGTATTGGATTTGCAGTCCCAAGAGCCGTTATACCAGGACTAAAAGAATTTCTTTATGA
TATACACCCTAGCAAGGATCCAAATGATGTCTTGACCATTGAATTCTGGCAAACTGCTTTTAACTGTACCTGGCCC
AACAGCAGTGTGCCTTACAATGTGGACCACAGAGTGAACATGACTGGCAAAGAAGACAGACTGTATGACATGTCTG
ATCAGCTCTGCACTGGAGAGGAGAGGCTGGAAGACCTGAAAAATACCTA
>pr3_PS .
GGGCCAGGTACAGTTAAAAGCAGTTTGCCAGAATTCAATGGTCAAGACATCATTTGGATCCTTGCTAGGGTGTATA
TCATAAAGAAATTCTTTTAGTCCTGGTATAACGGCTCTTGGGACTGCAAATCCAATACTTCCTCCAAAATATGGAA
AGTACTCAGGCTTTGCAATGAGGGCTGAGGTAATCCAGGCTTCGCTGGCTATCCATGTCCTGTCTGTTATGTTGTG
ATGAATCATTTCCAGTACAAATGGGCTGAGGTCAATGTCAGTTGTAAAAAGCACAACGACTCTGGCCGTGGAACCC
TTTACTGCATCAACAGCTTTTTTCATTTTCTCATTGGAGTAGACTTTGGGAATGGTTTCAGAGAAA
>ns8_PS .
GGGACTGGATGTCTCAGATGATGACCAAAGTGTTCAGTTTCTCTCAGACTTTAGGGGAGAGATGAAAGGAAACAGA
GTCTGCTTAGGCTTTGTGAACATTATTCCTCTAAGTACACAGCTATACATGATGAGAGCTCAAATATATTATGAAC
AAATAATGGAATAATCAGCAAATGTTGTGGTCATTTATGGTAATGTGAACTCTAGTTCAAAGCTATAGATGGTGGA
AATATTTAGACATAAGGAGAATCTGGTTCACTACTTCATAATGGGATAATATCATCAATCAGAGAGATTTCAACCT
TGACTCATCCCTTGTGACTTTTAATTTTTCACATCACCATGGTGAAGTTTCTGGTTTTAAAATTTTTTATCCAGAC
ATTGAGACTTTCTGAATACTCTGAATTTCTGAATTTACTCTGACAAGACCAGGGTGGATAGGTTTTAATTGTTCAG
TGGCATCTATTAACTTCAAGACACTGGAGGATTGGTCACCCAACACCTCATGTGAATCATTAATGTTACAGAATTT
TAACATGGCCATGAGTGATGAAAGCTATAGTTTGTAC
>ns4_PS .
GGGACTGGTTGTCTCAGATGATAACCAAGGTGTTCAGTTTCTTTCAGACTTTAGGGGAGAGATGAAAGAAAACAGA
GTCTGCTTAGCCTTTGTGAACATGATTCCTCTAAGTACACAGCTATACATGATGAGAGCTCAAATATATTATGAAC

AAATCATGGAATCAACAACCACTGCTGTGGTCATTTATGGTAATGTGAACTCTAGTTCAAAGCTATAGATGGTGGA
AATATTTAGGCATAAGGAGAATCTGGTTTACCACTTCACAATGGGATAATATCATAAATCAGGGAGATTTCAGCCT
TGATACATTCCTCGGGACATTCACTTTTTCGCATCACCATGGTGAAATTTCTGGTTTTAAAAATTTTATCCAGGCA
TTGAGACTTTCTGAATACTCTGAAGAAATTTCTCTGACAAGACTGGGGCGGATAGGTTTTAATTGTTCAGTGGCAT
CTAGTAACTTCAAGACACTGAAGGATTGGTCATCCAACAGCTCATTTGAGTGGTTAATGTGGCAGAATTGTAACAT
GGCCATGAATGATGAAAGCTACAATCTACAC
>ns14_PS .
TCTCTCAGACTTGAGAGGAGAGATGAAAGAAAAGAGTCTGCCTAGCCTTTGTGAACATGATTCCTCCAAGAATGCA
GTTATACATGAGGAGAGATCAAATATATTATAAACAAATCATTACTTCATCATCAAATGTCATTTATGATAATATG
AACTCAATTCTAATTGAAAGCTATAGATGGTGGAAATATGTAGGCATAAGGAGAATCTGATTCACCACCTCACAAT
GGGTTAATATCATAAATCAGAGAGATTTCAACCTTGACTCATTCCCTGGGACATTCATTTTTTCACATCACAATGG
TGAGATTTTGGGTTTTAAAATTTTTATCTTGACATTGAGACTTTCTGAATAATCTGAAGAAATTACTCTGGCAAGA
CTGGGGTGGATAGGTTTTAATTGTTCACTGGATCTAGTAACTTCAAGACACTGAAGGATTGGTCATCCAACACCTC
ATTTGAGTGGTTAATGTGGCACAATTTTAATATGGCCATGAGTGATGAAAGCTACAATATATAC
>ns10_PS .
GGGGCTGGTCACCTCAGATGGTTACCAGGAGGTCCATTTTCTTTCAGAATGAGGGGAGAGATGAAAAGTCACTGAG
TCTGTTTAGCTTTTGTGAATTTGATCCCAGAAAACATGGAATTATACAGATCAAGGACTGAAATACATTACAAACA
AATTATGACTTCCTCAGCTAATGTCATCATTTATGGAAGTGAGCATTAGGTATTAGGAGGATTGGGACATATGGAA
AATCTGGGTCATGACCTCACAATGGGATGTTATCTCAAAGACCAGAGAGTTCCATGGTGAGTCATTCCATGGGACC
TTGACTTTTTCACATCAACATGGTGAGATTTCTAATCTCAAAAATTTATCCAAGGCATCGAACTTTCCCTAATACT
CAGATGGCATTGCTGTGGTGAAATTGGGGTGGATGTATTTGAATTTTTCAATCTTGAAATATAACTGTAAAATATT
AAATAATTGTTCATCCAACCCATCCTTGGGATGGTTACCATGGCACAGTTTTGACATGACTATGAATGATGAGTGT
TACAATCTATGC
>ns7_PS .
TGGACTGGTCATGTCAGATAATGACCAGTGTATTCAATATCTCCCAGACTTTAGGGATGAGATACAAAGAAATGGA
ATCTAGTCAGCTTTTGTGATTGTAATCCCAATAAATTTTGATTTATATATGACAAGAGCTCATCTATATTATAACC
AAATTGTGATGTCATCGGCAAAATTTCTTATTATTTATGGTGATTTCAGCTCTGCTCTGGATTGGAGCTTTAGAAT
GTGGGAACGTTTATGTACAGGGAGAGTCTGGTCACCACCTCACAATGAGATGTTATCACAAGTCAAAGAGTCTTAA
CACGACTCATTTCATGACACTTTCACATTTTCACAACACTATCATGAGATTTTAGGTTTTAGAAACATTATAGAAT
CCTTAAACATGTCTGCATATTCAGAAGACATTTTGCAAGCCATATTGGGTTTGAACGAATTCAAATGTTCAGTGTC
AGAATCCCTCTGTGAAAGACTAAATAATTTTTCATCCAACACCTCATTGGAATGGTTATCATGGCACACTTTGGAA
ATTACCATGAGCAATGAGATTTATAATATATAC
>ns13_PS .
GGGAGTGCTCATTTTAGAAGACGAGCAAGGTATTCAATTTCTCTCAGACTTGAGAAGAGAGATTGAAAAGAATAGC
ATCTGTATAGCCTTTGTGAACATGATTCCTGTCTCTCTGGTATTATACTTGAAAAGTGCTCAGATATACTCTGATC
AGATCATGTTATCATCAGCAAATGTTTTAATCATTTTTGGAGATAGTGACTCTTCTCTTGGCTTGGCATCAAAAAT
ATCAGGCCATAGGGTCACACAAAGAGCCTGGGTCTCCACTTCACAATGGGTTGTTACCACAAATAAGGAAAATCTC
ATCCTTTATTCTTTCAATGGGACTATTATTTTCAAACATCAGCAGGAGGAGATTCCTGTTTTTAAAAAATTTGTCC
AGGCAACAAACCCTTTCAAATATTCAAAAGACACTTACCTTGACAAGTTGTGGTGGATGTATTTTAATTGCTCAGT
TTCAACATCTACCTGTAAATCACCGAGGAACTGTTCCTCAAAAGGCTCTTTGGAGTGGTTATCTTGGACAGGTTTT
GATATGGCCATGAGTGAAGGAAGTTACAATATGTAT
>ns11_PS .
AGGACTTTTGATTTCAGATGATGATCAAGGAATTCAATTTTTTACAGAGTTGAAGGAGCAGATGAAAATAAATGGA
GCCTTCTCAGCTTTTGTGAATGTAATCCCAGTGACTAGGCAGTTATACATGACAAGAGCTGACATATACTATAACC
AGATTGTGACATCATCAGCAAAAGTTTTTGTCATCTATGGTGACTTGAAATATAGCAACATGGAAAAAAAATTTTT
TTCACTTTGCTGTGAATATAACAACATGGAAAATTTTAAGCACATGCAGAATCTGGGTCAGCACCTCACAATAGGA
TGTTGTCATGAATAGGAGAAATTTAATCCTAGATCCATTCCATGGGACTTTTACTTTTTCACACCACCATGGTGAA
ATT
>jc_LOC101611716 .
GGGGCTGGTCATCCTAGATGATGACCAAGGTATTCAGTTTCTCTCAGACATGAGAGAAGAGATGCAAAGACAACAA
GTCTGTTCATCCTTTGTTAATATACTTCCAGTGAGCATGCATTTATTCCTCACAAGTGCTGAAGTATATGATAAGA
GAATTGTGACATCCACGGCAAATGTTGTTATCATTTATGGTGACATGAACTCGACTCTAGGTTTGAGCTTTAAAAG
ATGGGAATATTTAGTTATGGGAAGAATCTGGGTCACCTTCTCACAATGGGATATTATGGGAAACCGTGATTTCACT
CAAGACTTACCCCATCCGACCATCACTTTTTCACATCACCATCGTGACATTTCTGATTTTAAAGAATTTATTTGGA
CAGTTAACACTTCCAAACACATAGAAGATGTTTTAATGCCCAGATTTGGGTGGATGCATTATAACTGTGCTTCCTC
AGCATCCCACTGTAAAACACTGAAGAACTGTTCATCCAATGGCTCTTTGGAATGGTTGCCATGGCATCATTTTGAC
ATGGCTATGAGTAGCTGGAGTTACAACCTATAC
>jc_LOC101612014 .
GGGGCTGGTCATCACTGATGATGACCAAGGTGTTCAGTTTTTCTCAAACTTGAGACAAGAGATGCATAGAAATGAA
ATCTGTTTAGCCTTTGTGAATGTGATCCCAGTTAACATGCAGGTATACATAATGAGGGCTGAAACATATTATAACC
AAATTGTGACATCATCAGCAAAAGTTGTTATTATTTATGGTGAGAAGAATTCTACTCTAGAAGTGAGCTTTAGAAG
ATGGGAATGTTTAGGCACACAGAGAATCTGGGTCACCACCTCAAAATGGAATACTTTAACAAGGAAGAGAGATTTT

AACCTAGACTTATACCTCAATACTTTCATTTTTTCACACCACCATCATGAAATTTCTAGTTTTAAAAATTTCATAC
AGACAGTGAACTTTTCCACATACCCAAATGGCATCACTTTGAAGCAACTGAGTTGGATGTATTTTAATTGTTCAAC
CTCTATATCTTACTGTAAAACAATGAATAATTGTTCATCAAACACTATATTGGAATGGTTACTACAGCACAGTTTT
GACATGTCAATGAATGATGACTGCTACAATATTTAC
>jc7 .
GGGGCTGGTCATCTCCGATGATGACCAAGGTATTCAATTTCTCTCAGACTTCAGAGAAAAGATGCAAGGAAATGAA
CTCTGTTTAGCCTTTGTGAATGTGATCCCATTAAACATGAACATATACAATGCAAGAGCCGAGAAATATTATGAAC
AAATTGTGACATCATTGGCAAATGTTGTGGTCATTTATGGTGAAATAAATTCTACACTGGAATTGAGCTTTAGAAG
ATGGGCATATTTAGGCATCCGTAGGATCTGGGTCACCACCTCACAGTGGGATGTCATCACAAATACAGGAAGACAC
TTCATTCTTGACTCATTCTATGGTGCTTTCACATTTTCTAATCACCATCATGAGATTCCCAATTTTAAAAAATTTA
TTCAGTCAATGAACACTTCTCAATATCCAATAGACTTTTTGGTTATGACATCAGAATGGATGTACTTTAATTGTTT
GGACATTGAATCTAAATGTAGATCACAGAGTAAATGTGCACTCAATAATTCATTAAAATGGTTAGCAAGTCAAAGA
TTTGACATAGCCATGAATGACGAGAATTATAATCTATAC
>jc14_PS .
TTGGTCATCTCCGATGATGACCAAGGTATTCAATTTCTCTCAGACTTCAGGGAAAAGATGCAAGGAAATGAACTCT
GTTTAGCCTTTGTGAATGTGATCCCATTAAACATGGACTTATACAATACAAGGGCTGAGAAATATTATAAACAAAT
TGTGACATCATTGGCAAATGTTGTGGTCATTTATGGTGAAATAAATTCTACACTGGAATTGAGCTTTAGAAGATGG
GCATATTTAGGCATCCGTAGGATCTGGGTCACCACCTCACAGTGGGATGTCATCACAAATACAGGAAGACACTTCA
TTCTTGACTCATTCTATGGTGCTTTCACATTTTCTAATCACCATCATGAGATTCCCAATTTTAAAAAATTTTATTC
AGTCAATGAACACTTCTCAATATCCAATAGACTTTTTGGTTATGACATCAGAATGGATGTACTTTAATTGTTTGGA
CATTGAATCTAAATGTAGATCACAGAGTAAATGTGCACTCAATAATTCATTAAAATGGTTAGCAAGTCAAAGATTT
GACATAGCCATGAATGACGAGAATTATAATCTAT
>jc2_PS .
GGGGCTGGTCATCTCCGATGATGATCAAGGTATTCAATTTCTCTCAGACTTCAGAGAAAAGATGCAAGGAAATGAT
CTCTGTTTAGCCTTTGTGAATGTGATTCCATTAAACATGAACATATACAATACAAGAGCCGAGAAATATTATGAAC
AAATTGTGACATCATTGGCAAATGTTGTGGTCATTTATGGTGAATTAAATTCCACACTGGAAGTGAGCTGTAGAAG
ATGGGCAAATTTAGGCATCCGTAGGATCTGGGTCACCACCTCACAGTGAGATGTCATCACAAATACAGGAAGACAC
TTCATTCTTGACTCATTCTATGGTGCTTTCACATTTCCTAATCACCATCATGAGATTCCCAATTTTAAAAAATTTA
TTCAGTCAATGAACACTTCTCAATATCCAATAGACTTTTTGGTTATGACATCAGAATGGATGTACTTTAATTGTTT
GGACATTGAATCTAAATGTAGATCACAGAGTAAATGTGCACTCAATAATTCATTAAAATGGTTAGCAAGTCAAAGA
TTTGACATAGCCATGAATGATGAGAATTATAATCTATAC
>jc20 .
GGGGCTGGTCATCTCTGATGATGACCAAGGTATTCAATTTCTCTCAGACTTCAGAGAAAAGATGCAAGGAAATGAT
CTCTGTTTAGCCTTTGTGAATGTGATTCCATTAAACATGGACATATACAATACAAGAGCCGAGAAATATTATGAAC
AAATTGTGACATCATTGGCAAATGTTGTGGTCATTTATGGTGAAATAAATTCTACACTGGAATTGAGCTTTAGAAG
ATGGGCATATTTAGGCATCCATAGGATCTGGGTCACCACCTCACAGTGGGATGTCATCACAAATACAGGAAGACAC
TTCATTCTTGACTCATTCTATGGTGTTTTCACATTTTCTAATCACCATCATGAGATTCCCAATTTTAAAAAATTTA
TTCAGTCAATGAACACTTCTCAATATCCAATAGACTTTTTGGTTATGACATCAGAATGGATGTACTTTAATTGTTT
GGACATTGAATCTAAATGTAGAACACAGAGTAAATGTGCACTCAATAATTCATTAAAATGGATTGCAAGTCAAGGA
TTTCACATAGCCATGAATGATGAGAATTATAATC
>jc23 .
GGGGCTGGTCATTTCCGATGATGATCAAGGTATTCAATTTCTCTCAGACTTCAGAGAAAAGATGCATGGAAATGAT
CTCTGTTTAGCCTTTGTGAATGTGATTCCATTAAACATGAACATATACAATACAAGAGCCGAGAAATATTATGAAC
AAATTGTGACATCATTGGCAAATGTTGTGGTCATTTATGGTGAATTAAATTCCACACTGGAATTGAGCTTTAGAAG
ATGGGCATATTTAGGCATCCGTAGGATCTGGGTCACCACCTCACAGTGGGATGTCATCACAAATACAGGAAGACAC
TTCATTCTTGACTCATTCTATGGTGCTTTCACATTTTCTAATCACCATCATGAGATTCCCAATTTTAAAAAATTTA
TTCAGTCAATGAACACTTCTCAATATCCAATAGACTTTTTGGTTATGACATCAGAATGGATGTACTTTAATTGTTT
GGACATTGAATCTAAATGTAGATCACAGAGTAAATGTGCGCTCAATAATTCATTAAAATGGTTTGTAAATCAAGGA
TTTCACATAGTCATGAATGACGAGAATTATAATCTATAC
>jc15 .
GGGTCTGGTCATCTCTGATGATGACCAAGGTATTCAATTTCTCTCAGACTTCAGAGAAAAGATGCAAGGAAATGAT
CTCTGTTTAGCCTTTGTGAATGTAATTCCATTAAACATGGACTTATACTATACAAGGGCCAAGAAATATTATGAAC
AAATTGTGACATCATTGGCAAATGTTGTGGTCATTTATGGTGAAATAAATTCCACACTGAAAGTGAGCTTTAGAAG
ATGGGCAAATTTAGGCATCCGAAGGATCTGGGTCACCACCTCACAGTGGGATGTCATCACAAATACAGGAAGAGAC
ATCAATTTTGACTCATTCTATGGTGCTTTCACATTTTCTAATCACCATCATGAGATTCCCAATTTTAAAAAATTTA
TTCAATCAATGAACACTTCTCAATATCCAATAGACTTTTCGGTTCTGAGATCAGAATGGATGTACTTTCATTGTTT
GGACATTGAATCTAAATGTAGAACACAGAGTAAATGTGCACTCAATAATTCATTAAAATGGTTTGCAAGTCAAGGA
TTTGACATAGCCATGAATGATGAGAGTTATAATCTATAT
>jc11 .
GGGTTTGGTCATCTCCGATGATGACCAAGGTATTCAATTTCTCTCAGACTTCAGAGAAAAGATGCAAGGAAATGAA
CTCTGTTTAGCCTTTGTGAATGTGATCCCATTAAACATGGACTTATACAATACAAGGGCTGAGAAATATTATAAAC
AAATTGTGACATCATTGGCAAATGTTGTGGTCATTTATGGTGAATTAAATTCCACACTGGGAGTGAGCTTTAGAAG

ATGGGTATATTTAGGCACCCAGAGGATCTGGGTCACCACCTCACAGTGGGATGTCATTGCAAATACAAGAAGAGAC
TACATTCTTGACTCATTCTATGATGCTTTCACATTTTCTAAACACCATCACAAGATCCCTAATTTTAAAAAATTTA
TTCAGACAATGAACACTTCCCAATACTCAATAGACTTTTCTGTTATGAGATCAGAATGGATGTACTTTAATTGTCT
GGAGATTGAATCTAAATGTAGAACACAGAATAAATGTGCCCTCAATAATTCATTAAAATGGTTTGTAAATCAAGGA
TTTCACATAGTCATAAATGACGAGAATTATAATCTATAC
>jc10 .
TCATGTATAGTGAATTAAATTCCACACTGGGAGTGAGCTTTAGAAGATGGGTATATTTAGGCACCCAGAGGATCTG
GGTCACCACCTCACAGTGGGATGTCATTGCAAATACAAGAAGAGACTACATTCTTGACTCATTCTATGATGCTTTC
ACATTTTCTAAACACCATCACAAGATCCCTAATTTTAAAAAATTTATTCAGACAATGAACACTTCCCAATACTCAA
TAGACTTTTCTGTTATGAGATCAGAATGGATGTACTTTAATTGTCTGGAGATTGAATCTAAATGTAGAACACAGAA
TAAATGTGCCCTCAATAATTCATTAAAATGGTTTGTAAATCAAGGATTTCACATAGTCATAAATGACGAGAATTAT
AATCTATAC
>jc_LOC101596453 .
GGGGCTGGTCATCTCTGATGATGACCAAGGTATTCAATTTCTCTCAGACCTCAGAGAAAAGATGCAAGGAAATGAA
CTCTGCTTAGCCTTTGTGAATGTGATCCCGTTAAACATGCAGTTATATAATACAAGGGCTGAGAAATATTATCAAC
AAATTGTGACATCATTGGCAAATGTTGTGATCATTTATGGTGAAATGAATTCTACACTGGAAGTGAGCTTTAGAAG
ATGGGCATATTTAGGCATCCATAGGATCTGGGTCACCACCTCACAGTGGGATGTCATCACAAATACAGGAAGAGAC
TTCATTCGTGACTCATTCCATGGTGCTTTCACATTTTCTAATCACTATCAAGAGATTCCTAATTTTAAAAAATTTA
TTCAGACAGTGAACACTTCCCAATATCCAATAAACTTTTCTGTTATGCAATCAGAATGGATGTGCTTTAATTGTTT
GGACATTGAATCTAAATGTGAAACACAGAGTAAGTGTTCACTCAATACTTTATTAAAATGGTATGCAAGTCAAGGA
CTTGACATAGCCATGAATGATGAGAGTTATAATCTATAC
>jc_LOC101599746 .
GGGGCTGGTTATCTCTGATGATGACCAAGGTATTCAGTTTCTCTCAGATTTCAGAGAAAAGATACAAGGAAATGAA
ATCTGTTTAGCCTTTTTGAATGTGATCCCAGTAAACATAAAGTTATACAATACAAAGGCTGAAAAATATTATAAGC
AAATTATGACATCATTGGCAAATGTTGTGATAATTTATGGTGAAATGAATTCTACACTGGAAGTGAGCTTTAGAAG
ATGGGTATATTTAGGCATCCGTAGGATCTGGGTCACCACATCACAGTGGGATGTCATTGCAAAAACAGGGAGAGAC
TACATTCTTGACTCATTCTATGATGCTTTTACATTTTCTAAACACCATCACGAGATCCCTAATTTTAAAAAATTTA
TTCAGACAATGAACACTTCCCAATACTCAATAGACTTTTCTGTTATGAGATCAGAATGGATGTACTTTGATTGTCT
GGTGATTGAATCTAAATGTAGAACAAAGAGCAAATGTTCACCCAACACTTCATTAGAATGGTATGCAAGTCAAGGA
TTTGACATAGCCATGAATGATGAGAATTACAATCTATAC
>jc_LOC101596509 .
GGGGCTGGTCATCTCTGATGATGACCAAGGTATTCAATTTCTCTCAGACTTGAGAGAAGAGATGCAAGGAAATGAG
GTGTGTTTAGCCTTTGTGAATGTGATCCCACTAAACATGGAGTTGTACATTACAAGGGCTGAGACATATTATAACC
AAATTGTGATATCACTGGCAAATGTTGTGATCATTTATGGTGAAATGAATTCTACTTTAGAAGTGAGCTTTAGACA
ATGGGAATATTTAGGTATCCGGAGGATCTGGGTCACCACATCACAGTGGGATGGCGTCACAAATACAAGAAGAGAC
TTCATTCTTCATGCATGCTGTGGGACTTTCTCTTTTTCTAGCCACCATCATGAGATTCCCAACTTTAAAGAAATTA
TTCGCACATTTAACACTTCCCAATATCCAATAGACACTTCTCTGATGAGATCAGAATGGATGTACTTTAATTGTTC
AAACATTGATTCTCAATGTAAAACCCAGAGTAAGTGTTCACCCAACACTTCATTGGAATGGTATGCAGGGCAAGGA
TTTGACATGGCCATGAATGATGAGAATTATAATCTATAC
>jc1_PS .
GGGGCTGGTCATCTCTGATGATGACCAAGGTATTCAATTTCTCTCAGACTTCAGAGAAAAGATGCAAGGAAATAAA
CTGTGCTTAGACTTTGTGAATGTGATCCCAATAAACATGAAGTTATACAATACAAGGGCTGAGAAATATTATGAAC
AAATTGTGACATCATTGACAAATGTTGTGATCATTTATGGTGAAACGAATTCTACACTGGAAGTTGGCTTTAGAAG
ATGGACATATTTAGGCATCTGGAGGATCTGGGTCATTACTTTACAGTGGGATGTCATCTCAACTATGAGAAGAGAC
TTCATTCTTGCCTCATTCCATGGGACTTTCATTTTTTCTAACCACTATCACCAGATTCCCAATTTTAAAAAATTAT
TCAGACAATGAACACTTCCAAATATCCAATAGACATTTCTCTGATGAGGTCATAATGGATGTACTTTAACTGTTGA
ATCTAAATGTAGAACACAGAATAAGTCTTCACCCAACACCTCTTTAGAGTGGTATGCAGGTCAAGGATTTGACATG
GCCATGAATGATGAGACTTATAATCTATAC
>jc_LOC101594644 .
GGGGCTGGTCATCTCTGATGATGACCAAGGTATTCAATTTCTCTCAGACTTCAGAGAAAAGATGCAAGGAAATGAA
CTGTGCTTAGACTTTGTGAATGTGATCCCAATAAACATGAAGTTATACAATACAAGGGCTGAGAAATATTATGAAC
AAATTATGACATCATTGACAAATGTTGTGATCATTTATGGTGAAATGAATTCTACACTGGAAGTGGGCTTTAAAAG
ATGGACATATTTAGGCATCCATAGGATCTGGGTCACCACCTCACAGTGGGATGTCATCACAAATACAAGAAGAGAC
TTCATTCATGACTCATTCTATGGAGCTTTTACATTTTCTCATCACCATCACGAGATTCCCAATCTAAAAAAATTTA
TTGAGACAATGAACACTTCCCAATATCCAATAGACATCTCTGTGATGAGATCAGAATGGATGCACTTTAATTGTTT
GTACATTGAATCTCAATGTAGAAGACAGAGAAAGTGTTCACCTAACATCTCATTAGAATGGTATGCAAGTCAAGAA
TTTGACATAGCTATGAATGATGAGAATTATAATCTATAT
>jc9 .
AGGGCTTATCATCTCTGATGATGACCAAGGTATTCAATTTCTCTCAGATTTGAGAGAAAAGATGCAAGGAAGTGAA
GTCTGTTTAGCCTTTGTGAATGAGATACCATTAAACATGGAGTTATACAATACAAGGGCTGAGATATATTATAACC
AAATTGTGACGTCATTGGCAAATGTTGTGATGATTTATGGTGAAATGAATTCCACAGTAGAAGTGAGCTTCAGAAG
ATGGGCCTATTTAGGCATCCAGAGGATCTGGGTCACCACTTCACAGTGGGATGTTGTCACCAAACATACAAGCAGA

GACGTTAGTTTTGACTCATTTCATGGGACTTTCATTTTTTCTAACCACCATCAGGAGATTCCCAATTTCAAAAAAA
ATATTCAGACAATGAACACTTCAAAATATCCAATAGACCTTTCTCTGATGAAGTCAGAATGGATGTACTTTAATTG
TTCAAATATTGAATCTAAATGTAGAACACAGAATAAGTCTTCACCCAACACCTCTTTAGAGTGGTATGCAGGCCAA
GGATTTGACATGGCCATGAATGATGAGACTTATAATCTTTAC
>jc8 .
GGGGCTTATCATCTCTGATGATTTCCAAGGTATTCAATTTCTCTCAGACTTGAGAGAAAAGATGCAAGGAAGTGAA
GTCTGTTTAGCCTTTGTGAATGAAATTCCATTAAACATGGAGTTATACAATACAAGGGCTGAGACATATTATAACC
AAATTGTGACGTCATTGGCAAATGTTGTGATGATTTATGGTGAAATGAATTCCACAGTAGAAGTGAGCTTCAGAAG
GTGGGCCTATTTAGGCATCCAGAGGGTCTGGGTCACTACTTCACAGTGGGATGTTGTCACCAAACATACAAGCAGA
GACATTAGTTTTGACTCATTTCATGGGACTTTCATTTTTTCTAACCACCATCAGGAGATTCCCAATTTCAAGAAAA
ATATTCAGACAATGAACACTTCAAAATATCCAATAGATCTTTCTCTGTTGAAGTCAGAATGGATGCACTTTAATTG
TTCAAATATTGAATCTAAATGTAGAACACAGAATAAGTCTTCACCCAACACCTCTTTAGAGTGGTATGCAGGTCAA
GGATTTGACATGGCCATGAATGATGAGACTTATAATCTTTAC
>jc16 .
GGGGCTTATCATCTCTGATGATGACCAAGGTATTCAATTTCTCTCAGATTTGAGAGAAAAGATGCAAGGAAGTGAA
GTCTGTTTAGCCTTTGTGAATGAGATACCATTAAACATGGAGTTATACAATACAAGGGCTGAGATATATTATAAAC
AAATTGTGACATCATTGGCAAATGTTGTGGTCATTTATGGTGAATTAAATTCCACACTGGAAGTGAGCTTTAGAAG
ATGGGCAAATTTAGGCATCCATAGGATCTGGGTCACCACCTCACAGTGGGATGTCATCACAAATACAGGAAGAGAC
ATCAATTTTGACTCATTCTATGGTGCTTTCACATTTTCCCATCATCATCCTGAAATTCACAATTTAAAAAACAATA
TGCAGACAATGAACATTTCTCAATATCCAATAGACATTTCTGTGATGAAATCAGAATGGATGTACTTTAATTGCTT
GGACGTTGAATCTACATGTATAACACAGAGTAAGTGTTCACCCAACACCTCATTAGAATGGTATGCAAGTCAACAA
TTTGATACAGCCATGGATGATGAGAATTATAACCTATAC
>jc26 .
GGGTTTGGTCATCTCTGATGATGACCAAGGTATTCAATTTCTCTCAGACTTGAGAGAAAAGATGCAAGGAAATCAA
GTCTGTTTAGCCTTTGTGAATGTGATCCCATTAAACATGGAGTTATACAATTCAAGGGCTGAGACATATTACAAAC
AAATTGTGACACATTTGACAAATGTTGTGATCATTTATGGTGAAGGTAATTCCACGCTAGAGGTGAGCTTCAGGAG
ATGGGCACATTTAGGCATCCAGAGGATCTGGGTCACTACTTCACAGTGGGATGTCATCACCAATATGAGAAGAGAC
TTCATTCCTGACTCATTCCATGGGGGTTTCACTTTTTCTAACCACCATCATGAGATTCCCAATTTTAAAAAATTTA
TCCAGACACTGAACACTACCACATATCCAATACACATGTCTTTGATGATGTCAGAATGGATGTACTTTAATTGCTC
AGACTTAGAATCTAAACATAGAATACAGAGTATGAATTCACCCAACACCTCATTAGAATGGTATGCAGGTCAAGGA
TTTGAGATGGCCATGAATGATGACAATTATAATCTATAC
>jc_LOC101600514 .
GGGTTTGGTCATCTCTGATGATGACCAAGGTATTCAATTTCTCTCAGACTTGAGAGAAAAGATGCAAGGAAATCAA
GTCTGTTTAGCCTTTGTGAATGTGATCCCATTAAACATGGAATTATACAATTCAAGGGCTGAGATATATTACAAAG
AAATTGTGACACATTTGACAAATGTTGTGATCATTTATGGTGAAGGTAATTCCACACTAGAGGTGAGCTTCAGGAG
ATGGGCACATTTAGGCATCCAGAGGATCTGGGTCACTACTTCACAGTGGGATGTCATCACCAATATGAGAAGAGAC
TTCATTCCTGACTCATTCCATGGGGGTTTCACTTTTTCTAACCACCATCATGAGATTCCCAATTTTAAAAAATTTA
TCCAGACACTGAACACTACCACATATCCAATAGACATGTCTTTGATGATGTCAGAATGGATGTACTTTAATTGCTC
AGACTTAGAATCTAAACATAGAATACAGAGTATGTATTCACCCAATACCTCATTAGAATGGTATGCAGGTCAAGGA
TTTGAGATGGCCATGAATGATGACAATTATAATCTATAC
>jc_LOC101600515 .
GGGTTTGGTCATCTCTGATGATGACCAAGGTATTCAATTTCTCTCAGACTTGAGAGAAAAGATGCAAGGAAATCAA
ATCTGTTTAGCCTTTGTGAATGTGATCCCATTAAACATGGAGTTATACAATTCAAGGGCTGAGACATATTACAAAC
AAATTGTGACACATTTGACAAATGTTGTGATCATTTATGGTGAAGGTAATTCCACACTAGAGGTGAGCTTCAGGAG
ATGGGCACATTTAGGCATCCAGAGGATCTGGGTCACTACTTCACAGTGGGATGTCGTTGCCCATATGAGAAGAGAC
ATCATTCTTGACTCATTCCATGGGGGTTTCACTTTTTCTAACTACCATCATGAGATTCCCAATTTTAAAAAATTTA
TTCAGACAATGAACACTTCCCAATATCCAATAGACCTTTCTGTTATGAGATCAGAATGGATGTACTTTAATTGTTC
AGACTTTGGATCGAAGTGTGGAATACACAGTAAGTGTTCACCCACCTCATTAGAATGGTATGCAGATCCAGGATTT
GACATGGCCATGAATGATGACAATTACAATCTATAC
>jc_LOC101595386 .
GGGGCTGGTCATCTCTGATGATGACCAAGGTATTGAATTTCTCTCAGACTTGAGAGAAAACTTACAACAAAATGAA
GTCTGTTTAGCCTTTGTGAATGTGATCCCATTAAACATGGAGTTAAGCACTAAAATGGTTGAGATACATTATAAAC
AAATTGCGACATCATTGGCAAAGGTTGTGATCATTTATGGTCAAATGGATTCTACACTAGAAATAAACTTTAAATT
ATGGGCATATTTAGGCATCTGGAGGATCTGGGTCACCACCTCACAGTGGGATGTTATCACAAATACCAGAGAAGAA
TTCATTTTTTACTCATTCCGTGGGGCTTTCACTTTTTCTAACCACCATCATCAGATTCCCAATTTTAAAACAATGA
TTCAGACAATAAACATTTCTAAATATCCAATAGACCTCTCTCTGATGAGATCAGAATGGATGTACTTTAATTGCTC
AGACATTGAATCTAAATGTAGAACACAGAGTAAGTGTTCACCCAGTACCTCGTTAGAGTGGTATGCAGGTCAAGGA
TTTGACATGGCCATGAATGATGAGAATTACAATCTGTAT
>jc3 .
GGAGCTGTTCATCTCAGATGATGACCAAGGCTTTCAATTTCTCTCTAACTTGAGAGTAGAAATGCAAAGATATGGC
ATCTGTTTAGCATATGTGAATATGTTCCCTGTAAACACAGATATATTATTGACAGCTCTTATAAATTTGCGTTATC
CAATCTTCACATCTTCGGCAAATGCTGTTGTCATTTATGGTAACATTAACTCTATTCTGGAAATGAGCTTTAGAAA

```
ATGGGAATGTTTAGGAGCATGGAGAATCTGGGTCACCACCTCACAGTGGGATGTCTTCCCATGTGAAGGAAACTTC
ATCCCTTACTCATACCGTTGGTCTTTCACTTTCGTACACCACCATGGAGAGATTTCTAGTTTAAAAACTTTTATCC
AGACAATGAACTTTTCCAAATATACAGAAAACTTTTATTCTGTAAAAACAAGATGGGTGGAATTTAATTGTTCTCT
CTCCAAATCATACTATGAAACACTGAACAACTATTCTTACAATCCTTCACTGGAGTGGTTATCAAGGCACAGCTTT
GACGTGGCCATGGATGAAGAGTTTTACAACATCTAC
>jc21 .
GGAGCTGTTCATCTCAGATGATGACCAAGGCTTTCAATTTCTCTCTAACCTGAGATTAGAAATGCAAAGACATGGC
ATATGTTTAGCATATGTGAATATGATCCCTGTAAACACAAATATATTATTGACAGCTCTTATAAATTTGCGTTATC
CAATCTTCACATCATCAGCAAATGCTGTTGTCATTTATGGTAACATTAACTCTATTCTGGAAATGAGCTTTAGAAA
ATGGGAATGTTTAGGAGCATGGAGAATCTGGGTCACCACCTCACAGTGGGATGTCTTTCCATGTGAAGGAAACTTC
ATCCCTTACTCATTACGTTGGTCTTTCACTTTCGTACACCACCATGGAGAGATTTCTAGTTTAAAAACTTTTATCC
AGACAATGAACTTTTCCAAATATATAGAAAACTTTTATTCTGTAAAAACAAGATGGGTGGAATTTAATTGTTCTCT
CTACAAATCATACTATGAAACACTGAACAACTATTCTTACAATCCTTTACTGGAGTGGTTTTCAAGGCACAGCTTT
GACATGGGCATGGATGAAGAGTTTTACAACATCTAC
>jc6 .
GGAGCTGTTCATCTCAGATGATGACGTAGGCTTTCAATTCCTCTCTAAATTGAGAGTAGAAATGCAAAGATATGGC
ATCTGTCTAGCATATGTGAATATGATCCCTATGAACGCAGATATAGCACTGACATTTTTTATATATATGCGTTATC
CAATCTTCACATCGTCGGCAAATGCTGTTGTCATTTATGGTAACATTAACCCTATTATGGAAATGAGCTTTAGAAA
ATGGGAATGTTTAGGAGCATGGAGAATCTGAGTCACCACCTCACAGTGGGATGTCTTTCCATGTGAAGGAAACTTC
ATCCCTTACTCATTACGTTGGTCTTTCACTTTCGTACACCACCATGGAGAGATTTCTCGTTTAAAAACTTTTATCC
AGACAATGAACTTTTCCAAATATACAGAAAACTTTTATTCTGTAAAAACAAGGTGGGTGGAATTTAATTGTTCTCT
CTACAAATCATACTATGAAACACTGAACAACTATTCTTACAATCCTTTACTGGAGTGGTTTTCAAGGCACAGCTTT
GACATGGGCATGGATGAAGAGTTTTACAACATCTAC
>jc19 .
GGAGCTGTTCATCTCAGATGATGACGTAGGCTTTCAATTCCTCTCTAAATTGAGAGTAGAAATGCAAAGATATGGC
ATCTGTCTAGCATATGTGAATATGATCCCTATGAACGCAGATATAACATTGACATTTTTTATATATATGCGTTATC
CAATCTTCACATCGTCGGCAAATGCTGTTGTCATTTATGGTAACATTAACCCTATTCTGGAAATGAGCTTTAGAAA
ATGGGAATGTTTAGGAGCATGGAGAATCTGGGTCACCACCTCACAGTGGGATGTCTTTCCATGTGAAGGAAACTTC
ATCCCTTACTCATTACGTTGGTCTTTCACTTTCGTACACCACCATGGAGAGATTTCTAGTTTAAAAACTTTTATCC
AGACAATGAACTTTTCCAAATATACAGAAAACTTTTATTCTGTAAAAACAAGATGGGTGGAATTTAATTGTTCTCT
CTACAAATCATACTATGAAACACTGAACAACTATTCTTACAATCCTTTACTGGAGTGGTTTTCAAGGCACAGCTTT
GACATGGGCATGGATGAAGAGTTTTACAACATCTAC
>jc17_PS .
GGAGCTGTTCATCTCAGATGATGACTAAGGCTTTCAATTTCTCTCTAACCTGAGAGTAGAAATGCAAAGACATGGC
ATATGTTTAGCATATGTGAATATGATCCCTGTAAACACAAATATATTATTGACAGCTCTTATAAATTTGCGTTATC
CAATCTTCACATCATCAGCAAATGCTGTTGTCATTTATGGTAACATTAACTCTATTCTGGAAATGAGCTTTAGAAA
ATGAGAATGTTTAGGCACACGGAGAATCTGGGTCACCACCTCACAGTGGGATCTCCTCCCATGTGAGGGAGATTTC
GTCTCTTATCCATTCCGTTGGTCTTTCACTTTCATACACCACCATGGTGAGATTTCTAGTTTAAAACCCTTTATCC
AGACAATGAACTTTTCCAAATATACAGAAAACATTAATTCTGTAAGATCAAGGTGGATGGGTTTTAATTGTTCTCT
CTCCAAATCATACTATGAAACACTGAACAACTATCTTTCCAACCCTTCACTGGAGTGGTTTTCAAGGCACAGCTTT
GACATGGACATGGATGAAGAATTTTACAACA
>jc4 .
GGAGCTGTTCATCTCAGATGATGACCAAGGCTTTCAATTTCTCTCTAACCTGAGAGTAGAAATGCAAAGACATGGC
ATATGTTTAGCATATGTGAATATGATCCCTGTAAACACAAATATATTATTGACAGCTCTTATAAATTTGCGTTATC
CAATCTTCACATCATCAGCAAATGCTGTTGTCATTTATGGTAACATTAACTCTATTCTGGAAATGAGCTTTAGAAA
ATGAGAATGTTTAGGCACACGGAGAATCTGGGTCACCACCTCACAGTGGGATCTCCTCCCATGTGAAGGAAACTTC
ATCCGTTTCTCATTCCGTTGGTCTTTCACTTTCGTACACCACCATGGTGAGATTTCTAGTTTAAAACCTTTTATCC
AGACAATGAACTTTTCCAAATATACAGAAAACATTAATTCTGTAGGATCAAGGTGGATAGGTTTTAATTGTTCTCT
CTACAAATCGTACTATGAAAAACTGAACAACTATTCTTCCAACCCTTCACTGGAGTGGTTTTCAAGGCACAGCTTT
GACATGGACATGGATGAAGAATTTTACAACATCTAC
>jc18 .
GGAGCTGTTCATCTCAGATGATGACCAAGGCCTTCAATTTCTCTCTAACTTGAGAGTAGAAATGCAAAGATATGGC
ATCTGTTTAGCATATGTGAATATGTTCCCTGTAAACACAGATATATTATTGACAGCTCTTATAAATTTGCGTTATC
CAATCTTCACATCTTCGGCAAATGCTGTTGTCATTTATGGTAACATTAACTCTATTCTGGAAATGAGCTTTAGAAA
ATGGGAATGTTTAGGCACACGGAGAATCTGGGTCACCACCTCACAGTGGGATCTCCTCCCATGTGAGGGAGATTTC
ATCCCTTATCCATTCCGTTGGTCTTTCACTTTCGTACACCACCATGGTGAGATTTCTAGTTTAAAACCTTTTATCC
AGACAATGAACTTTTCCAAATATACAGAAAACATTAATTCTGTAAGATCAAGCTGGATGGGTTTTAATTGTTCTCT
CTCCAAATCATACTATGAAACACTGAACAACTATTCTTCCAACCCTTCACTGGAGTGGTTTTCAAGGCACAGCTTT
GACATGGACATGGATGAAGAGTTTTACAACA
>jc22 .
GGAGCTGTTCATCTCAGATGATGACCAAGGCTTTCAATTTCTCTCTAACTTGAGAGTAGAAATGCAAAGATATGGC
ATCTGTTTAGCATATGTGAATATGTTCCCTGTAAACACAGATATATTATTGACAGCTCTTATAAATTTGCGTTATC
```

CAATCTTCACATCTTCGGCAAATGCTGTTGTCATTTATGGTAACATTAACTCTATTCTGGAAATGAGCTTTAGAAA
ATGGGAATGTCTAGGCACACGGAGAATCTGGGTCACCACCTCACAGTGGGATCTCCTCCCATGTGAGGGAGATTTC
ATCCCTTATCCATTCCGTTGGTCTTTCACTTTCGTACACCACCATGGTGAGATTTCTAGTTTAAAAACTTTTATCC
AGACAATGAACTTTTCCAAATATACAGAAAACATTAATTCTGTAAGATCAAGGTGGATGGGATTTAATTGTTCTCT
CTCCAAATCATTCTATGAAACACTGAACAACTATTCTTCCAACCCTTCACTGGAGTGGTTTTCAAGGCACAGCTTT
GACATGGACATGGATGAAGAGTTTTATAACA
>jc_LOC101617402 .
GGAGCTGTTCATTTCAGATGATGACCAAGGCTTTCAATTTCTCTCTAACTTGAGAGTAGAAATGCAAAGATATGGC
ATCTGTCTAGCATATGTGAATATGATCCCTGTAAACACAGATATAGCATTGACATTTTTTATTAATATGCATTATC
CAATCTTCACATCATCGGCAAATGCTGTTGTCATTTATGGTAACATTAACCCTATTATGGAAATGAGCTTTAGAAA
ATGGGAATGTTTAGGCACATGGAGAATCTGGGTCACCACCTCACAGTGGGATCTCCTCCCATGTGAGGGAGATTTC
ATCCCTTACTCATTCCGTTGGTCTTTCACTTTCGTACACCGCCATGGTGAGATTTCTAGTTTAAAACCTTTTATCC
AGACAATGAACTTTTCCAAATATACAGAAAACATTAATTCTGTAAGATCAAGCTGGATGGGATTTAATTGTTCTCT
CTACAAATCATACTATGAAACACTGAACAACTATTCTTACAATCCTTCACTGGAGTGGTTTTCAAGGCACAGCTCT
GAAATGGCCATGGATGAAGAGTTTTACAACATCTAC
>jc_LOC101598138 .
GGGACTGGTCATCTCAGATGATGACCAGGGTATTCAGTTTCTGTCAGACTTGAGAGAAGAGATGCAAAGAAATGGA
GTCTGCTCTGCTTTTGTGAATGTTGTCCCAGTAAACAACCAGTTGTACTTGAGAAAATATGGAATATATGATAAGC
ACATTATGACATCATCAGCAAATGTTATCATCATGTATGGTGACACTAGCTCTACTCTAGATGAAACCATCAGAAT
GTTGCAATATTTAGGCACAGGAAAAATCTGGGTCACAACCACACAGTGGCATGAGATCTCAAGTAAGAGATATTTA
AGCCTTGACCCATTCCGTAGCATTTTCACTCTTTCTCCACACCATGGTGATATTTATGGATTTAAGAAATTTATCC
AAACACTGAAATCTTCCAAGTACTTAGAAGACATTTCTTTGGAAAAGTTGGGGTGGATGTATTTTAATTGTTCTGT
GTTAGAGTCTAAATGGAAAACCGAGTATAATTTTTCAGCAAACATCTCGTTGGAATTGTTCACACAGCACACATTT
GATGTGGCCATGAGTGAGGAGAGTCACCATATATAC
>jc_LOC101609526 .
GGGACTGGTCATCTCAGATGATGACCAAGGTATTCAATTTCTGGCAGACTTGAGAGAAGAGATGCAAAGAAATGGA
ATCTGCTCTGCTTTTGTGAATGTTGTCCCAGTGAACAACCAGCTGTACTTGAGAAGACATGGAATATATGATAAGC
ACATTATGACATCATCAGCAAATGTTATCATCATGTATGGTGACACTAGCTCTACTCTAGATGAAACCATCAGAAT
GTTGCAATATTTAGGCACGGGAAAGATCTGGGTCACAACCACACAATGGTATGATATCTCAAGTAAGAGATACATA
AGCCTTGACTCATTCCATAGGACTTTCACTCTTTCTCAACACCATGGTGATATTTATGGATTTAAGAAATTTATCC
AAACACTGAAATCTTCCAAGTACTTAGAAGATATTTCTCTGGAAAATTTGGGGTGGATGTATTTTAATTGTTCTGT
ATCAAAGTCTAAATGGAATACCTTGTATAATTTTTCATCAAACATATCATTGGAATTGTTCACAAAGCACACATTT
GATGTAGCCTTGAGTGAGGAGAGTTACCATATATAC
>jc_LOC101607531 .
GGGGCTGGCCATCCCTGATGATGACCAAGGTACCCAATTTCTCTCAGACTTGAGAGAAAACATACAAGGAAATGAA
CTCTGCTTAGCCTTTGTGAATGCGATCTCATTAAACATGGAGTTATACAATACAAGGGCTGAGATATATTATAAAC
AGATTGTGACATCATTGGCAAATGTTGTTATCATTTATGGTGAAATGAATTCTACACTAGAATTGAGTTTTATAAG
ATGGGCTTATCTACGCATCCAGAGGATCTGGGTTACCACCTTACAGTTAGATGTCATCACAAATATGAAAGGAGAT
TTCATGCTTGACTCATTCCTTGGGACTATTGTTTTTTCAACCCACCATCATGAGATTCCCAATTTCAAAAAATTTA
TCCAAACAATGAGCACTTCCACATATCCAATAGATATTTCACTGCTGAAATCAGAATGGATGAACTTTAATTGTTC
AGACAATGAATCTAAATGCCAATCACAGAATGAACTTCCTACCTGTTACAAACACATTTCTGGTTTTCCTCAGATC
CCCTCCTCTGTGTGCAATGAGACTTGCAGGCCTGGT
>mcr22 .
TGGACTGGTCATCTCTGACAGTGATCTTGGCATTCAATTTCTCTCATATTTGAGAAGAGAGTTTGGAAAAAATACA
GTCTGCTTTGCCTTTGTAAATATAATTCCAATCAGTATGCAATTATACATGTCAAGAGCTGAATTGTATTATAACA
AAATCAAGGCATCATCCACAAAAGTTGTTATCATTTATGGTGACACAGACAGTACTCTTGCTGTAAGCTTTCGAAT
GTGGGAATCTCGAGGTTTACAGAGAATATGGGTCATCACCTCACAGTGGGATGTGACTACCAGTAAAAGAGATTTC
ATGCTTGAATCACCACATATGACTATAGCTTTTGCACGCCAACATGGTGAGATTACTGGTTTTAAAAATTTTGTCC
AGACAATAAACCCTCTCAGGTACACAGATAAATACCTTGCAAATCTGGAGTGGATGAAGTTTAACTGTGAAGTCTC
CAGGTCCAACTGTAAGACACTGAAGAGATACTCATCAAATATCTCCATGGAATGGCTAGTGGTACAGACTTTTGAC
ATGGCCTTTAGTGGTGGCAGTTATGACATATAC
