# Supplementary material for: Evolution of Spatially Coexpressed Families of Type-2 Vomeronasal Receptors in Rodents
Source: Genome Biol Evol. 2014 Dec 23;7(1):272–85. doi: 10.1093/gbe/evu283 (PMC4316634; doi:10.1093/gbe/evu283)
Supplement: Supplementary Data [file supp_evu283_Supplementary_file_S4.pdf]

Reconstructed family-C genes.

>dpVmn2r1a-like **[Dipodomys ordii], kangaroo rat** vomeronasal type 2 receptor (V2R), Vmn2r1-like
ATGGCCAACAGAAGGACATACGTGGTTCTGGGAGTTCTGCTGTTTGTATGGGCCAAGTTGGGCACTAAAGGCCAAGAAGA
AGAAGAGGAAGTGACCTGCAGGTTGCTGGGCAAGTTTGATTTGAATGGATATGTGGATGCCAAAAACCATTCCGTTGTTA
TTGGAGGTCTGTTTCCTATTCACTCCAGGACCATCCCAGCAAATGAGTCTATTTTGGAGCCAGTGTCAGCCAAATGTGAA
GGGTTCAACTTTCGAGGATTCCGCTGGATGAAGACTATGATCCACACCATCCAGGAGATCAATGAGAGGAAGGACATTTT
GCCCAAGCACACTCTGGGCTATCAGATCTTTGATTCCTGTTTCACTGTGTCCAAAGCGATGGAGTCAGCTTTGGTATTCC
TCACAGGGCAGGAAGAAAACAAACCCAACATGAGAAACAGCACCGGCGCCTATCTGGCGGGAATTGTTGGATCAGGAGGC
TCAGCCCTATCGATTGCTGCGTCAAGACTTCTAGGGCTATATTATGTACCTCAGGTGGGTTATGCCTCTACCAGCTCAGT
TCTTAGTGACAAATACCAGTTTCCAAGTTATTTTCGCATAGTACCTAGTGATAATATCCAATCCCAGGCCATGGTGAAAC
TTATCCAGCATTTTGGTTGGGTATGGGTGGGCACTATCGCATCTGATGATGACTATGGAAAATATGGTGTTAAAGTTTTT
AAGGAAAACGTGGAGAGTGCCAACCTCTGTGTTGCATTCTCTGAAACCATCCCCAAAGTATATTCCAATGAGAAAATGCA
AAAGGCTGTCAATGCTATTAAAGGCTCCTCTGCCAAAGTCATCGTGCTGTATGCCTCTGATCTGGACCTCAGTCCCTTCG
TGCTGGAGATGGTGTACCACAACATAACGGACAGGACATGGATAGCGAGCGAAGCATGGATCACCTCGGCCCTCTTGGCA
AAACCTGAATACTTCCCCTATTTTGGTGGAAGTATTGGGTTTGCAGTGCCAAGAAGTGACATCCCGGGGTTGAAAGAATT
TCTTTATGACGTGCATCCCAGCAAGGATCCAAATGATGTCCTGACCATCGAATTCTGGCAGACAGCATTTAACTGTACTT
GGCCCAATAGCAGTGTTCCTTACAATCTGGACCACCGAGTGAATATGACCGGGAAAGCGGACCGACTGGAGGCCACATCT
GATCGGTTGTGTACTGGAGAGGAGAGGCTGGAAGATCTTAAAAATACCTACCTGGATGTGTCTCAGCTGAGAATCACAAA
CAATGTCAGACAAGCTGTGTATACTCTGGCTTATGCCCTGGATGAGCTCAGCAGATGTGACAAAGAGAATCCAAGATGGC
ACTGTGCCTACATACCTGCATTTGAGCCCTGGCAGCTATCCTACTATATGAGGAGAGTTAAGTTTACAGCTCATGATGGA
AAGAAAATAGAGCTTAATGATGATGGAGATTTGGATGCTGGGCACTATGATATTCTAAACTGGCAAACAAATGATAATGG
AGAAGTTGACTTTGTTAAGATTGGAGAATACAATTTCAGAACCTCTAAATTTGAAATTGTTTTTGCAAAGAAGTCAACAA
TATTTTGGAACACTGAATCATCCAAGCTTCCCCATTCAGTGTGTACCGATGTGTGTCTTCCTGGGACCCGGAAAGGGATT
CGTCAAGGGGAGCCGATCTGCTGCTTTGATTGCATCCCATGTGCTGATGGACATGTGTCAGAGAAACCAGATAAGAGGGA
GTGCACGAAGTGTGGTGAGGACTACTGGTCTAATGAAGACAGGAGCAAGTGTGTGCTCAAAGAGGTGGAATTCCTGGCCT
ATGACGAGGCCCTGGGCTTCACCCTGGTCATCCTGTCCATCCTCGGGGCCCTGGTGGCGCTGGCGGTCACCGTGGTCTAT
GTGCTCTACAGGAGGACTCCCCTGGTGCAGGCCAATGACCGGGAGCTGAGCTTCCTCATCCAGGTGGCGCTCCTCATCAT
CATGCTGTCCTCTCTGATCTTCATCGGCAAGCCACACACCTGGTCCTGCAGGGCCCGCCAGGTCACTCTGGCCCTGGGCT
TTGCTCTTTGCCTCTCCTCCATTCTGGGAAAGACCATTTCACTCTTCCTGGCCTACAGAATTTCTGCCTCCAAAACTCGG
TTCCTCTCCATGCGCCCCGTGTTTCGCAAGCTCATCGTGTTGCTCTCCGTGCTGGTGGAGGCTGGCATATGCATCGCCTA
CTTGCTGATGCGCCCCCCGAGGGTGTACAAGAACATGGAGTCTCAGGTGATCAAGATCATTCTGGAGTGCAACGAGGGTT
CTATCGAGTTTTTGTGCGTCGTGTTTGGGGTCGACGTCTTGCTGGCCTTCCTGTGCTTTCTGAGCAGCTTCGTGGCTCGC
CAGCTGCCAGATAATTATTACGAAGGCAAATGCATCACTTTTGGCATGCTGGCCTTCTTCATCATCTGGATCTCTTTTGT
GCCCGCCTACTTGAGCACCAAAGGCAAATTCAAGGTGGCCGTGGAGATCTTTGCCATCCTGGCATCCAGCTACGGTTTGT
TGGGCTGTATATTTGCTCCCAAGTGCTTCATCATTTTGCTGAGGCCGAAGAGGAACACGGCTGAGACAGTTGGGGGAAGA
GTGCCCACTGCAGATAAGAGCATCCAGCTGACATCTGCATCTGTGAGCAGTGAGCGGAATGAGACTGCCATGTTCTCAGG
TCTGGATGAC

>dpVmn2r1b-like **[Dipodomys ordii], kangaroo rat** vomeronasal type 2 receptor (V2R), Vmn2r1-like,
ATGGCCAGCAGAAGGACATACCTGGTTCTGGGAGTTCTGCTGTTTGTATGGGCCGAGTTGGGCACTAAAGGCCAAGAAGA
AGAAGAGGAAGAGACCTGCAGATTGATGCGAAAGTTTGATTTGAATGGTTATGTGGATGCCAAAAACCATTCCATTATTA
TTGGAGGTCTGTTTCCTATTCACTCCAGGACCATCCCAGAAAACGAGTCTAGTTTCGAGCCAGTGTCAGCCCAATGTGAA
GGGTTCAACTTTCGAGGATTCCGCTGGATGAAGACCATGATCCACACCATCCAGGAGATCAATGAGAGGAAGGACATTTT
GCCCCAGCACACTCTGGGCTATCAGATCTTTGATACATGTTGCACCGTGTCCAAAGCTATGGAGTCAGCTGTGGTCATGC
TCATGGGGCAGGAAGAAAACAAACCCAACATAAGAAACAGCACCGGGGCCTATCTGCTAGGAGTTATTGGATCAGGAGGC
TCAGCCTTATCGATTGCCACTTCAAGACTCCTGAGCTTGTACAGATTACCTCAGGTGGGTTATGCTTCTACCTGCTCACT
TCTCAGTGACGAATTCCAGTTTCCAAGTTATTTTCGCACAATAGCCAGTGATACTATCCAGTCCCAGGCCATGGTGAAAC
TTATCCTGCACTTTGGCTGGGTGTGGGTGGGCACGATTGCAACTGATGACGACTATGGAAAATATGGTGTAAAAGTTTTT
AAAGAAAACTTGGAGAGTGCCAACCTCTGCGTTGCATTCTCTGAAACCATCCCCAAAGTCTATTCCAATGAGAAAATGCA
AAAGGCTGTGAATGCTATTAAAGGCTCCTCCGCCAAAGTCATCGTGCTGTATGCCTCTGATCTGGACCTCAGTCCCTTCG
TGCTGGAGATGGTGTACCACAACATAACGGACAGGACATGGATAGCGAGCGAAGCATGGATCACCTCGGCCCTCTTGGCA
AAGCCTGAATACTTCCCCTATTTTGGTGGAAGTATTGGGTTTGCAGTGCCAAGAAGTGACATCCCGGGGTTGAAAGAATT
TCTTTATGACGTGCATCCCAGCAAGGATCCAAATGATGTCCTGACCATCGAATTCTGGCAGACAGCATTTAACTGTACTT
GGCCCAATAGCAGTGTTCCTTACAATCTGGACCACCGAGTGAATATGACTGGGAAAGAGGACCGACTGGAGGCCACATCT
GATCGGTTGTGTACTGGAGAGGAGAGGCTGGAAGATCTTAAAAATACCTACCTGGACGTGTCTCAGCTGAGAATCACAAA
CAATGTCAGACAAGCTGTGTATACTCTGGCTTATGCCCTGGATGAGCTCAGCAGATGTGAGGAACTTAAAGGGCCAGACC
AGTTCTGTCCATATATGCCTACTTTTCAACCACAGGAACTGACGGAATATGTAAGTAGTCTTAGATTTACAACACATGAT
GGAAGGAAAGTAGAGTTTGATGAAAGTGGAGATATAAAAGAAGGAAACTATGATATTCTAAACTGGCAATTAGATGATAA

TGGAGAAGTTGCCTTCATAAAGCTTGGAGAATATACTTTCAAAGAATCTAAGTATGAACTCCGCTTCAAAAAGGAGTCAC
CAATATTTTGGAACACAGAATCATCAAAGGTTCCCTATTCGGTGTGCACCGACGTGTGTCCTCCCGGGACCCGCAAGGGG
ATTCGTCATGGTAAACCCATCTGCTGCTTTGATTGCATCCCATGTGCTGATGGACATGTGTCAGAAAAACCAGGTGAGAG
GGAGTGCACGCAGTGTGGTGAGGACTACTGGTCTAATGAAGACAGGAGCAAGTGTGTGCTCAAAGAGGTGGAATTCCTGG
CCTATGACGAGGCCCTGGGCTTCACCCTGGTCATCCTGTCCATCCTCGGGGCCCTGGTGGCGCTGGCGGTCACCGTGGTC
TATGTGCTCTACAGGAGGACTCCCCTGGTGCAGGCCAATGACCGGGAGCTGAGCTTCCTCATCCAGGTGGCGCTCCTCAT
CATCATGCTGTCCTCTCTGATCTTCATCGGCAAGCCACACACCTGGTCCTGCAGGGCCCGCCAGGTCACTCTGGCCCTGG
GCTTTACTCTTTGCCTCTCCTCCATTCTGGGAAAGACCATTTCACTCTTCCTGGCCTACAGAATTTCTGCCTCCAAAACT
CGGTTCCTCTCCATGCGCCCCGTGTTTCGCAAGCTCATCGTGTTGCTCTCCGTGCTGGTGGAGGCTGGCATATGCATCGC
CTACTTGCTGATGCGCCCCCCGAGGGTGTACAAGAACATGGAGTCTCAGGTGATCAAGATCATTCTGGAGTGCAACGAGG
GTTCTATCGAGTTTTTGTGCGTCGTGTTTGGGGTCGACGTCTTGCTGGCCTTCCTGTGCTTTCTGAGCAGCTTCGTGGCT
CGCCAGCTGCCAGATAATTATTACGAAGGCAAATGCATCACTTTTGGCATGCTGGCCTTCTTCATCATCTGGATCTCTTT
TGTGCCCGCCTACTTGAGCACCAAAGGCAAATTCAAGGTGGCCGTGGAGATCTTTGCCATCCTGGCATCCAGCTACGGTT
TGTTGGGCTGTATATTTGCTCCCAAGTGCTTCATCATTTTGCTGAGGCCGAAGAGGAACACGGCTGAGACAGTTGGGGGA
AGAGTGCCCACTGCAGATAAGAGCATCCAGCTGACATCTGCATCTGTGAGCAGTGAGCGGAATGAGACCGCCGTGTCCAC
AGTTCTGGATGAC

>crVmn2r1-like **[Cricetulus griseus], chinese hamster** vomeronasal type 2 receptor (V2R), Vmn2r1-like
ATGGCCAGCAGAATGATATGCTTGGCTCTAGGATTTCTTGCATTTTTATGGGCTGAATTGGGAGCTCAAGACAAAACAAC
GGAGGAACACCAATGCAGGTTGATGGCCAAGTTTAATTTGTCTGGATATGTAGATGCCAAAAACCATTCACTTGTTATTG
CAGGACTGTTTCCTATTCACTCCAGGATCATCCCTGTAGATGAATCTATTTTGGAGCCAGTATCACCCATGTGTGAAGGG
TTTAACTTCCGGGGATTCCGGTGGATGAAAACCATGATCCACACTATCAAGGAGATTAATGAGAGGAACGACATTTTGCC
CAACCATACTCTGGGCTATCAGATCTTTGACACCTGCTATACCATCACTAAAGCAATGGAGTCAGCTTTAGTGTTTCTTA
CAGGGCAAGAAGAATTCCAGCCCAATTTTAGAAACAGCACTGGATCAATTTTGGCAGCAATGGTTGGAGCGGGGGGATCG
TCCTTGTCAGTTGCAGCTTCAAGAATTCTTGGGTTGTATTACATGCCTCAGGTGGGCTATACTTCTTCCTGCTCAATTCT
TAGTGACAAATTCCAGTTTCCTTCTTATCTTCGTGTAATACCCAGTGATAAGATCCAGTCTGAGGCCATGGTGAATCTTA
TCAAACACTTTGGTTGGGTCTGGGTAGGCGCTATTGCAGCTGATGATGATTATGGAAAATATGGAGTAAAGACATTTAAG
GAAAAAATGGAGACTGCCAACCTCTGTGTTGCTTTCTCTGAAACCATTCCCAAGATCTACTCCAATGAGAAAATGCAAAA
GGCTGTCGATGCAGTAAAGACTTCCACTGCCAGAGTCATTGTGCTTTTTACCTCTGACATTGATCTCAGCCCCTTTGTGC
TGGAAATGGTTCATCATAACATAACTGACAGAACCTGGATAGCCAGTGAAGCCTGGATTACCTCAGCCCTCATTGCAAAG
CCCGAGTACTTTCCATATTTTGGTGGAACTATTGGATTTGCAATACCAAGAACCATTATACCAGGATTGAAAGAATTTCT
TTATGATATACAACCTAGAAAGGATCCAAATGATGTCTTGACAATTGAATTCTGGCAAACTGCTTTTAACTGTACCTGGC
CCAATAGCAGTGTGCCTTACAATGTGGATCACAGAGTGAATATGACAGGCAAAGAAGACAGATTGTATGACATGTCTGAT
CAGCTCTGCACTGGGGAGGAGAAGCTGGAAGACCTGAAAAATACCTATCTGGATGTGTCTCAGCTCAGAACCACAAACAA
TGTCAAACAAGCTGTGTATGCTATCGCTTATGCCCTGGATGGCCTCAGCAGATGTCAAGAGGGACATGGGCCATATGATC
CAAATAACACTTGTGCGTATATACCTACCTTTGAGTTCTGGCAGCTGATGTACTATATGAAGGGAATTAAATTTAAAACG
CATGATGGAAAATGGATAGTTCTGGATGAAAATGGAGATGTACTACATGGACACTATGATATCTTAAACTGGCAATTAGA
CGACAATGGAGAAATTTCCTTTGTGACAGTTGGGAAATTCAACTTTAGAGCTACTAAGTTTGAGCTCTTTATTCCAAATA
ATTCTTCAATATTTTGGAACACTGAGTCATCAAGGCTTCCCTACTCAGTCTGCACACATGTGTGTCCTCCAGGGACCCGG
AAGGGGATTCGCCAGGGGGAGCCCATATGCTGCTTTGATTGCATCCCGTGTGCTGATGGATATGTGTCAGAGAAACCAGN
NNNNNNNNNNNNNNNNNNNNNNNNNNNNNNNNNNNNNNNNNNNNNNNNNNNNNNNNNNNNNNNNNNNNNNNNNNNNNNNN
NNNNNNNNNNNNNNNNNNNNNNNNNNNNNNNNNNNNNNNNNNNNNNNNNNNNNNNNNNNNNNNNNNNNNNNNNNNNNNNN
NNNNNNNNNNNNNNNNACAGGCACACTCCCTTGGTGAAGGCCAATGACCGGGAGCTGAGCTTCCTCATTCAGATGTCTCT
GGTCATCACAGTGCTCTCATCCATGCTCTTCATAGGCAAGCCATTGAACTGGTCCTGCATGGCCCGCCAGGTCACTCTGG
CACTAGGCTTTTGCCTTTGTCTGTCTTCCATTCTCGGAAAGACTATCTCACTCTTTTTTGCCTACAGGATTTCCAAATCC
AAAACCAGACTTATATCCATGCACCCCATTATTCGAAAACTCATAGTGCTGATCTGTGTTTTAGGGGAGATTGGTGTATG
CTCAGCTTACTTGCTGTTGGATCCTCCGAGAATGTACAAGAACATTGAACCTCAAAATGTAAAGATCATCTTTGAATGCA
ATGAAGGCTCTATAGAGTTTCTGTGTTCCATATTTGGGTTTGATGTCCTTCTGGCCTTACTGTGTTTTCTTACAACCTTT
GTGGCTCGCCAGCTGCCAGATAACTACTATGAAGGGAAATGCATCACTTTTGCAATGCTGGTCTTTTTCATTGTCTGGAT
CTCTTTTGTCCCTGCTTACTTGAGCACCAAAGGCAAATTCAAAGTGGCCGTGGAAATATTTGCCATTTTGGCATCCAGCT
ATGGCTTGTTAGGCTGCATATTTCTTCCCAAGTGCTTCATTATTTTGCTGAGGCCAAAGAGGAATACTGAGGAGACTGTG
GGTGGGAGAGTCCCCACTGTAGACAGGAGCATCCAGTTGACCTCAGCTTCTGTGAGCAGTGAGCTTAACAACACCACAGT
GTCCACTGTTCTGGATGAG

>crVmn2r2-like **[Cricetulus griseus], chinese hamster** vomeronasal type 2 receptor (V2R), Vmn2r2-like
ATGGTTAGCAGGAAGAGGTGCTTGGTTCTGGGATTAACTGCATTTTTATGGGTTGCACATGCACAACAGGAAGACCAGAA
ATGCAGGTTGCTGCGCAAGTTTAATTTGACTGGATATGTAGAAGCCAAAAACCATTCAGTTGTTATTGGAGGACTATTTC
CTATCCACTCTAGGACAATCCCAACAGATGACCCTGATGGAGAACCCGTATCAGCCATGTGTGAAGGGTTTAACTTCCGG
GGATTCCGCTGGATGAAAACCATGATCCACACCATCAAGGAGATTAATGAGCGGAAGGACATTTTGCCCAACCACACTCT
GGGCTATCAGATATTTGACACCTGCTATACCATCGCCAAAACAATGGAGACAGCTTTGGCATTTCTTACAGGACAAGAAG

AATTCCAGCCCAATTTTAGAAACAGCACTGGAAAATATCTAGTAGGAATTATTGGAGCTGGGGGATCATCCTTGTCAGTT
GCTGCTTCAAGAATTCTGGGGTTGTATTACATGCCTCAGGTGGGCTATACTTCTTCCTGCCCAATTCTTAGTGACAAATT
CCAGTTTCCATCTTATCTTCGCACAATACCCAGTGATAAGATCCAGTCTGAGGCCATGGTGACTCTCATCAAACACTTTG
GTTGGGTCTGGGTAGGCGCTATTGCAGCTGATGATGATTATGGAAAATATGGAGTAAAATCCTTTAGGGAAAAAATGGAA
AGCTCCAACCTCTGTGTTGCTTTCTCTGAAACCATTCCCAAAGTCTACTCCAATGAGAAAATGCAAAATGCTGTGACTGC
AGTGAAGAAGTCTACTGCCAGAGTCATTGTGCTTTTTACAACTGACATTGACCTCAGCCCCTTTGTGCTGGAAGTGGTTC
ATCATAACATAACTGACAGGACCTGGATAGCCAGTGAAGCCTGGATTACCTCAGCCCTCATTGCAAAGCCCGAGTACTTT
CCATATTTTGGTGGAACTATTGGATTTGCAGTACCAAGAACCATTATACCAGGATTGAAAGAATTTCTTTATGATATACA
CCCTAGCAAGGATCCAAATGATGTCTTGACCATTGAATTCTGGCAAACTGCTTTTAACTGTACCTGGCCCAACAGCAGTG
TGCCTTACAATGTGGACCACAGAGTGAATATGACTGGCAAAGAAGACAGATTATATGACATGTCTGATCAGCTCTGCACT
GGGGAGGAGAAGCTGGAAGACCTGAAAAATACCTATCTGGATGTATCTCAGCTCAGAATCACAAACAATGTCAAACAAGC
TGTGTATGCTATGGCTTATGGTCTGGATCGTCTCAGCAGATGTGACTTACCGAAAAGAGAAAATGCAGATGCAAAATGTT
CACACATGCCTGACTTTGAGCCCAGGGAGCTATTGGCTTACTTAAAGGAATTGACATTTACTACTCATGATGGAAGAAAT
ATACACTTTGACTTGAATGGAGATCTGGAAAGTGGATATTATGATATCCTCAATTGGCAAATAGATAATGCTGGAGAAAT
CGCCTTTGTCAAAGTTGGAGAATACAAATTCCAACAATCAAATTTTGAACTTGTTCTTCTGAAGAATGCAACATTATTTT
GGAACACTGAATCTTCAGGGCTTCCAGATTCAGTCTGCACAAAACTGTGTCCTCCAGGGACCCGGAAGGGGATTCGGCAG
GGGCAACCCATATGCTGCTTTGATTGCATCCCCTGTGCTGATGGATATGTGTCAGAGAAACCAGGTCAAAGAGAATGTGA
TGCATGTGGGGAAGACGACTGGTCCAATGCACAGAAGAGCAAGTGTGTGCCAAAAGAGGTGGAGTTCCTTGCTTATGAGG
AGGCCCTGGGATTCACCCTTGTCATCCTCTCCATCTTTGGGGCACTTGTGGTCTTGGCAGTCACTGTGGTGTATGTGATC
CACAGGCACACTCCCTTGGTGAAGGCCAATGACCGGGAGCTGAGCTTCCTCATTCAGATGTCTCTGGTCATCACAGTGCT
CTCATCCATGCTCTTCATAGGCAAGCCATTGAACTGGTCCTGCATGGCCCGCCAGGTCACTCTGGCACTAGGCTTTTGCC
TTTGTCTGTCTTCCATTCTCGGAAAGACTATCTCACTCTTTTTTGCCTACAGGATTTCCAAATCCAAAACCAGACTTATA
TCCATGCACCCCATTATTCGAAAACTCATAGTGCTGATCTGTGTTTTAGGGGAGATTGGTGTATGCTCAGCTTACTTGCT
GTTGGATCCTCCGAGAATGTACAAGAACATTGAACCTCAAAATGTAAAGATCATCTTTGAATGCAATGAAGGCTCTATAG
AGTTTCTGTGCTCCATATTTGGTTTTGATGTCCTTCTGGCCTTACTGTGTTTTCTTACAACCTTTGTGGCTCGCCAGCTG
CCAGATAACTACTATGAAGGGAAATGCATCACTTTTGCAATGCTGGTCTTTTTCATTGTCTGGATCTCTTTTGTCCCTGC
TTACTTGAGCACCAAAGGCAAATTCAAAGTGGCCGTGGAAATATTTGCCATTTTGGCATCCAGCTATGGCTTGTTAGGCT
GCATATTTCTTCCCAAGTGCTTCATTATTTTGCTGAGGCCAAAGAGGAATACTGAGGAGACTGTGGGTGGGAGAGTCCCC
ACTGTAGACAGGAGCATCCAGTTGACCTCAGCTTCTGTGAGCAGTGAGCTTAACAACACCACAGTGTCCACTGTTCTGGA
TGAG

>nsVmn2r1-like [Nannospalax galili] Nannospalax galili vomeronasal type 2 receptor (V2R), Vmn2r1-like
ATGGCCAGTAGAGAGATATGCTTGGTTCTAGGATTTCTGACGTTTTTGTGGGCTGAATTGGGTGGTCAAAGCAGGCAACA
GGAGGAACCAAAATGCAGGTTGATGGGCAAGTTTAATTTGAGTGGATATGTAGATGCCAAAAACCATTCACTTGTTATTG
GAGGACTATTTCCCATTCACTCCAGGATCATCCCGGCCGATGAATCTGTCTTGGAGCCAGTATCTGCCATGTGTGAAGGG
TTTAACTTTCGGGGTTTCCGCTGGATGAAAACCATGATCCACACGCTCAAGGAGATTAACGAGAGGAAGGATATCTTGCC
AAACCACACTCTGGGCTATCAGATCTTTGATTCCTGTTACACCATCTCCAAAGCAGTGGAGTCAGCTTTGGTGTTCCTTA
CAGGGCAGGAAGAATACCAGCCCAATTTCAGAAACAGCACTGGATCAATTCTGGCAGGAATTGTTGGATCAGGGGGATCG
TCCTTATCAGTCGCTGCTTCAAGAATTCTAGGGTTGTATTATTTACCTCAGGTGGGCTATACATCTTCCTGTTCAATTCT
CAGTGACAAATTCCAGTTTCCATCTTTTATTCGCATAGTACCTACTGATAAGATCCAGTCCGAGGCCATGGTGAATCTTA
TAAAACACTTTGGATGGGTCTGGGTAGGTGCTATTGCAGCTGATGATGATTATGGAAAATATGGTGTAAAAACTTTTAGG
GAAAAAATGGAGAGTGCCAACCTCTGTGTCGCTTTCTCTGAAACCATTCCCAAAGTCTACTCCAATGAGAAAATGCAGAA
GGCTGTTGATGCAGTAAAGACGTCCACTGCCAAAGTCATTGTGCTTTTTTCATCTGACATTGACCTCAGCCCTTTTGTGC
TGGAAATGATTCATCATAACATTACTGACAGGACATGGATAGCGAGCGAAGCATGGATTACCTCAGCTCTCATTGCAAAG
CCTGAGTACTTTCCATATTTTGGTGGAAGTATTGGATTTGCAATACCAAGAACTGATATACCAGGATTAAAAGAATTTCT
TTATGATGTACATCCCAGCAAGGATCCAAATGATGTCTTGACCATTGAATTCTGGCAAACTGCTTTTAACTGTACTTGGC
CTAATAGTAGTGTTCCATACAATGTTGACCACAGAGTGAATATGACTGGTAAAAAAGACAGACTATATGACATGTCAGAT
AAGCTCTGTACTGGAGAGGAGAAGTTGGAAGACCTTAAAAACACCTACCTAGATGTGTCTCAGCTAAGAATCACAAACAA
TGTCAAACAAGCTGTGTATGCTATGGCTTATGCTCTGGATCATCTAAGTAAATGTGAAGATGGACAGGGACCATTTACTG
ACAATAAGATTTGTGCAAAGATACCTACCTTTGAGCTCTGGGAGCTGATGTTCTACATGAAGTCACTCAAGTTTGAAACA
CATGATGGAAAGAAAATAGAAATAGATGATAATGGAGATGTGAAAAGTGGACATTATGATATTCTAAATTGGCAATTAGA
TGACAATGGAGAAATTTCCTTTGCAACGGTTGGAAAATATACTTTCCGAAATTCTATGTTTGAGCTCATTCTCCCAAAGA
ATTCTACAATATTTTGGAACACTGAATCATCAAGGCTTCCCCATTCAGTCTGTACGGATGTGTGTCCTCCAGGGACCAGG
AAGGGGATTCGTCAGGGGGAACCAATATGCTGCTTTGACTGCATCCCATGTGCTGATGGCTATGTGTCGGAGACACCAGG
CCAAAGGGAATGTGATAAATGTGGTGAAGACTTCTGGTCCAATGCACAGAAGAACAAATGTGTGCTGAAGGAAGTGGAAT
TCCTTGCTTACGAGGAGGCCCTGGGATTCACCCTCGTCATCCTCTCCGTCTTTGGGGCACTTGTGGTCTTGGCAGTGACA
GTTGTCTATGTGATCTACAGGCACACTCCGCTGGTGAAGGCCAACGACCGCGAGCTGAGCTTTCTCATTCAGCTGTCTCT
TGTCACCACGGTGCTGTCGTCCATGCTTTTCATCGGCAAGCCGTGCGACTGGTCCTGCATGGCCCGCCAGGTCACCCTGG
CGCTGGGCTTTTGCCTTTGCCTATCTTCTATTCTTGGAAAGACTGTCTCACTGTTCTTTGCCTACAGAATCTCCGTATCC
AAAACCCGGCTTACATCCATGTACCCCATTTTTCGAAAGCTCATTGTGCTGATCTCGGTTCTAAGTGAGACTGGTGTGTG
TATAGCTTACTTGGTGCTGGAGCCCCCAAAGTTATACAAGAACATGGAATCTCAAAATATAAAGATCATCTTTGAATGCA

ATGAGGGTTCCATAGAATTTTTGTGCTCCATATTTGGGTTCGACGTCCTTCTGGCCTTTCTGTGTTTTCTTACAACCTTT
GTGGCCCGCCAGTTGCCAGATAATTACTACGAAGGCAAATGCATCACTTTTGGGATGCTGGTCTTTTTCATTGTCTGGAT
CTCTTTTGTCCCTGCTTACCTGAGCACCAAAGGCAAATTCAAAGCGGCTGTGGAAATCTTTGCCATTTTGGCATCCAGCT
ATGGCTTGTTAGGCTGTATATTTGCTCCCAAGTGCTTCATTATTTTGCTGAGACCAAAGAGGAACACGGATGAAACTGTG
GGTGGAAGGGTCCCCACTGTGGACAGGAGCATCCAGCTGACTTCAGCGTCTATGAGCAGTGAGCTTAACATCACCACAGT
GTCCACTGTTCTGGATGGG
