# Supplementary material for: Evolution of Spatially Coexpressed Families of Type-2 Vomeronasal Receptors in Rodents
Source: Genome Biol Evol. 2014 Dec 23;7(1):272–85. doi: 10.1093/gbe/evu283 (PMC4316634; doi:10.1093/gbe/evu283)
Supplement: Supplementary Data [file supp_evu283_Supplementary_file_S5.pdf]

Spalax MHC sequences (Suppl. Fig. S10)

>SpH2_1 [organism=Spalax leucodon] Spalax leucodon histocompatibility 2, clone 1, partial sequence.
GGAGATGGGCAGGACCCGATCCAGGACATGGACCTTGTGGAGACCAGGCCTGCAGGG
GATGGAACCTTC
>SpH2_2 [organism=Spalax leucodon] Spalax leucodon histocompatibility 2, clone 2, partial sequence.
GGAGATGGCCAGGACCCGATCCAGGACATGGATCTTGTGGAACCATGCCTGCAGGG
GATGGAACCTTC
>SpH2_3 [organism=Spalax leucodon] Spalax leucodon histocompatibility 2, clone 3, partial sequence.
AGAGATGGGGAGGACCCGACCCAGGACATGGAGCTTGTGGAGACCTGGCCTTCAGGG
GATGGAAACTTC
>SpH2_4 [organism=Spalax leucodon] Spalax leucodon histocompatibility 2, clone 4, partial sequence.
AGGGATGGGGATGACTTGACCTAGGATATGGATGTTGTGGAGACCAGGCCTGCAAGG
GATGGAACCTTC
>SpH2_5 [organism=Spalax leucodon] Spalax leucodon histocompatibility 2, clone 5, partial sequence.
GGAGAGGGGAAGGACCTGACCCAGGACATGGAGCTTGTGGAGACCAGGCCTGCAGGG
GATGGAACCTTC
>SpH2_6 [organism=Spalax leucodon] Spalax leucodon histocompatibility 2, clone 6, partial sequence.
GGAGATGGGGAGGACCTGGCCAAGGACATGGAGCTTGTGGAGACCAGGCCTTCAGGG
GATGGAAACTTC
>SpH2_7 [organism=Spalax leucodon] Spalax leucodon histocompatibility 2, clone 7, partial sequence.
AGAGATGGGGAGGACCCGACCCAGGACATGGAGCTTGTGGAGACCAGGCCTGCAGGG
GATGGAACCTTC
>SpH2_8 [organism=Spalax leucodon] Spalax leucodon histocompatibility 2, clone 8, partial sequence.
AAGGATGGAGCAAACCAGACCCAGGACATGGAGCTTGTGGAGACCAGGCCTGCAGGG
GATGGAAACTTC
>SpH2_9 [organism=Spalax leucodon] Spalax leucodon histocompatibility 2, clone 9, partial sequence.
AGGGATGGAGCAAACCAGACCCAGGACATGGAGCTTGTGGAGACCAGGCCTGCAGGG
GATGGAAACTTC
>SpH2_10 [organism=Spalax leucodon] Spalax leucodon histocompatibility 2, clone 10, partial sequence.
AGAGATGGGGAGGACCCGACCCAGGACATGGAGCTTGTGGAGACCAGGCCTTCAAGG
GATGGAAACTTC
>SpH2_11 [organism=Spalax leucodon] Spalax leucodon histocompatibility 2, clone 11, partial sequence.
AGGGATGGGGAGGATCTGACCCAGGACATGGAGCTTGTGGAGACCAGGACTGCAGGG
GATGGAACCTTC
>SpH21_12 [organism=Spalax leucodon] Spalax leucodon histocompatibility 2, clone 12, partial sequence.
AGAGATGGGGAGGACCTGACCAAGGACATGGAGCTTGTGGAGACCAGGCCTGCAGGG
GATGGAACCTTC
>SpH21_13 [organism=Spalax leucodon] Spalax leucodon histocompatibility 2, clone 13, partial sequence.
GCAGATGGAGAGGACCAGACCCAGGAAATGGGGTTTGTGGAGACCAGGCCTGCAGGG
GATGGAAACTTC
>SpH21_14 [organism=Spalax leucodon] Spalax leucodon histocompatibility 2, clone 14, partial sequence.
GGGAATGGGGAGGACCTGACCCAGGACATGGAGCTTGTGGAGCCCAGGCCTGCAGGG
AATGGAAACTTC

>SpH2_15 [organism=Spalax leucodon] Spalax leucodon histocompatibility 2, clone 15, partial sequence.
AGAGATGGCATGGACCTGACCCAGGACATGGACCTTGTGGAGACCAGTCCTGCAGTG
GATGGAAACTTC
>SpH2_16 [organism=Spalax leucodon] Spalax leucodon histocompatibility 2, clone 16, partial sequence.
GGGGATGGGGATGACTTGACCTAGGATATGGATGTTGTGGAGACCAGGCCTGCAGGG
GATGGAACCTTC
