# Supplementary material for: Evolution of Spatially Coexpressed Families of Type-2 Vomeronasal Receptors in Rodents
Source: Genome Biol Evol. 2014 Dec 23;7(1):272–85. doi: 10.1093/gbe/evu283 (PMC4316634; doi:10.1093/gbe/evu283)
Supplement: Supplementary Data [file supp_evu283_Supplementary_Tables.pdf]

Supplementary Table S1: Primers used in this study.

Target	Name	Sequence	Length (bp)
Rodent family-A V2Rs	A207[a]	F1: 5’-TRVTTCAYTTYAGHTGGAMYTGGRT-3’<br>F2: 5’-TTCAYTTYAGHTGGAMYTGGRTDGG-3’<br>R: 5’- GCMAVAGCATRCACASCATT-3’	563 (F1); 560 (F2)
	A239[b]	F: 5’-TTAGCCTTTGTRARYATGATYCY-3’<br>R: 5’-TCAYTMAWGGCCAKKTYAAA-3’	590
Rodent family-C V2Rs	C224[c]	F: 5’-CTTCCMBAYTCAKTBTGYAC-3’<br>R: 5’-GGGATRCAATCAAAGCAGCAY-3’	92
Rodent H2-Mv	H255[d]	F: 5’- CTGAVATYACCCTRACCTGGMAG -3'<br>R: 5’- ACCAYCACWGCTGCCCAYTTCTG -3'	113
	H256[e]	F: 5’- GYTCACACTGGCTGMAGWYTTTCR -3'<br>R: 5’- CCAATAYTCAGGYRGCTCCTGCT -3'	124
	H257[f]	F: 5’-GTTCACACWCYCTGAGMTWTKT-3'<br>R: 5’- CTDTRRKTGTASATGTGRAGCA -3'	218
Mouse family-C V2Rs	mC117[g]	F: 5’- AGATGTCAAGAAGGGCAGGGA-3’<br>R: 5’-CTAATCATCCAGAATTACCCA-3’	140
	mC118[h]	F: 5’-ACTTGTGACTTATTGGAAGAA-3’<br>R: 5’CAAATCAAGTTCTATTTCTGC-3’	137
	mC120[i]	F: 5’-AATCAGAGTTTTAATAAAAGA-3’<br>R: 5’-AACATTAAGCTCTATTTTTCT-3’	138
	mC250[l]	F: 5’- GCGATGGATATTTTGAGACAAGATG -3’<br>R: 5’- AAATTCGTTACCCATGATGGAA -3’	136
	mC171[m]	F: 5’-ATAGATCGTTTTAGCCAAGCT-3’<br>R: 5’-TTCCATCATGGGTAATGAATTTTA-3’	130
Squirrel family-B V2Rs	sB223[n]	F: 5’-ATCAGCWATGGTCCATTTGACYAC-3'<br>R: 5’-GTATTCATATCCCAAACATGCAGA-3'	680

NOTE.- Primers specific for: [a]family-A of all rodent species; [b]*Anomalurus* family-A sequences; [c]exon 5 of both subfamily C1 and C2 V2Rs; [d]exon 4 of H2-Mv genes; [e]exon 2 of H2-Mv genes (M10 group); [f]exon 2 of H2-Mv sequences (M1-9-11 group); [g]Vmn2r1.[h]Vmn2R2/5; [i]Vmn2r3; [l]Vmn2r4; [m]Vmn2r5/6; [n]exon 3 of the Sciuridae family-B genes.

Length of the predicted amplified sequence is indicated.

Supplementary Table S2. Primer specificity for family-A V2Rs

**Species**	**Sequences**	**A207_F1**[a]			**A207_F2**[a]			**A207_R**[a]		
		**m0**	**m1**	**m2**	**m0**	**m1**	**m2**	**m0**	**m1**	**m2**
Mus musculus (ms)	97	20%	49%	69%	29%	61%	74%	28%	52%	70%
Rattus norvegicus (rt)	66	12%	27%	64%	45%	67%	91%	12%	39%	76%
Cricetulus griseus (cr)	80	14%	30%	44%	21%	41%	63%	14%	30%	46%
Jaculus jaculus (jc)	21	0%	48%	81%	10%	38%	57%	0%	52%	76%
Dipodomys ordii (dp)	97	35%	72%	91%	40%	61%	76%	41%	75%	91%
Cavia porcellus (cv)	85	11%	35%	58%	21%	52%	65%	11%	33%	55%

NOTE.- [a]Percentage of rodent V2RA sequences matching to the designed primers with 0, 1 and 2 mismatches.

Supplementary Table S3. Primer specificity of VNO (H2-Mv) and non-VNO expressing Mhc.

	**M10**[a] **(n=11)**	**M1-9-11**[b] **(n=13)**	**MHC**[c] **(n=107)**
**H255_F**	91%	100%	65%
**H255_R**	100%	100%	68%
**H256_F**	100%	0%	0%
**H256_R**	91%	0%	0%
**H257_F**	0%	62%	0%
**H257_R**	0%	92%	0%

NOTE.- percentage of: [a]*M. musculus*, *R. norvegicus* and *C. griseus* H2-Mv sequences (M10 group)matching the designed primers; [b]*M. musculus*, *R. norvegicus* and *C. griseus* H2-Mv sequences (M1-9-11 group) matching the designed primers; [c]*M. musculus*, *R. norvegicus* and *C. griseus* non-VNO expressing Mhc matching the designed primers. Sequences were retrieved by blastn search against *M. musculus*, *R. norvegicus* and *C. griseus* WGS databases using *M. musculus* H2-Mv genes as queries.

Supplementary Table S4. Average sequence identity of mouse V2RA subfamilies.

	[a]A	B	D	C
**A10**	39%	35%	32%	25%
**A9**	42%	35%	31%	25%
**A8**	44%	34%	32%	24%
**A6**	45%	38%	34%	26%
**A5**	45%	31%	31%	23%
**A4**	46%	32%	30%	24%
**A3**	45%	32%	30%	25%
**A2**	46%	33%	31%	24%
**A1**	45%	32%	31%	24%

NOTE.- [a]Average identity with respect to the other A-subfamilies

Supplementary Table S5. Subfamily A1-5 and ESP genes in rodent species

	A1-2	A5	A3	A4	ESPs[b]
Mus musculus	18	15	7	24	37
Rattus norvegicus	16	6	10	8	10
C. griseus	5	14	6	–	5
P. maniculatus	1	12	12	–	3
M. ochrogaster	–	–	1	–	1
N. galili[a]	7	–	–	–	–
S. leucodon[a]	17	–	–	–	–
J. jaculus[a]	14	–	–	–	–
Anomalurus[a]	–	–	–	–	–

NOTE.- Subfamily distribution of family-A putatively intact genes (non-redundant sequence database) or [a]both gene and pseudogenes based on exon-3 sequences (see Suppl. Figs. S5 and S6). The symbol "–" refers to sequences not identified in both the WGS and non-redundant sequence databases by iterated tblast and blastn search.

[b]Search for ESP genes against the *C. griseus* WGS and the non-redundant sequence databases was performed using the ESP sequences (exon 2) of *Mus musculus* (mouse) and *Rattus norvegicus* (rat) as inputs of tblastn and blastn queries. Mouse and *Cricetulus* sequences were employed as tblastn and blastn queries against the WGS and non-redundant sequence databases of *P. maniculatus* and *M. ochrogaster*, to obtain ESP sequences. No significant hits were retrieved from the databases of other rodent species using ESP sequences of mouse, rat and Cricetidae as input of blastn, tblastn, blastp and PSI blast queries.
